# A sequence-anchored genetic linkage map for the moss, *Physcomitrella patens*

**DOI:** 10.1111/j.1365-313X.2008.03637.x

**Published:** 2008

**Authors:** Yasuko Kamisugi, Mark von Stackelberg, Daniel Lang, Matthew Care, Ralf Reski, Stefan A Rensing, Andrew C Cuming

**Affiliations:** 1 Centre for Plant Sciences, Faculty of Biological Sciences, Leeds UniversityLeeds LS2 9JT, UK; 2 Plant Biotechnology, Faculty of Biology, University of Freiburg, Schaenzlestrasse 1D-79104 Freiburg, Germany; 3 Freiburg Initiative for Systems Biology (FRISYS), Faculty of Biology, Schaenzlestrasse 1D-79104 Freiburg, Germany

**Keywords:** *Physcomitrella patens*, linkage map, AFLPs, SSRs, genome sequence

## Abstract

The moss *Physcomitrella patens* is a model for the study of plant cell biology and, by virtue of its basal position in land plant phylogeny, for comparative analysis of the evolution of plant gene function and development. It is ideally suited for ‘reverse genetic’ analysis by virtue of its outstanding ability to undertake targeted transgene integration by homologous recombination. However, gene identification through mutagenesis and map-based cloning has hitherto not been possible, due to the lack of a genetic linkage map. Using molecular markers [amplified fragment length polymorphisms (AFLP) and simple sequence repeats (SSR)] we have generated genetic linkage maps for *Physcomitrella.* One hundred and seventy-nine gene-specific SSR markers were mapped in 46 linkage groups, and 1574 polymorphic AFLP markers were identified. Integrating the SSR- and AFLP-based maps generated 31 linkage groups comprising 1420 markers. Anchorage of the integrated linkage map with gene-specific SSR markers coupled with computational prediction of AFLP loci has enabled its correspondence with the newly sequenced *Physcomitrella* genome. The generation of a linkage map densely populated with molecular markers and anchored to the genome sequence now provides a resource for forward genetic interrogation of the organism and for the development of a pipeline for the map-based cloning of *Physcomitrella* genes. This will radically enhance the potential of *Physcomitrella* for determining how gene function has evolved for the acquisition of complex developmental strategies within the plant kingdom.

## Introduction

The moss *Physcomitrella patens* is the first non-flowering land plant for which an annotated genome sequence has been determined (Rensing *et al.*, 2008, http://genome.jgi-psf.org/Phypa1_1/Phypa1_1.home.html). A member of the bryophytes, the first group of plants to diverge from the modern land plant lineage, *Physcomitrella* retains many features typical of ancestral land plants (Kenrick and Crane, 1997; Rensing *et al.*, 2008). As such, it represents a fruitful source for the comparative analysis of gene function within the plant kingdom. An anatomically simple plant, *Physcomitrella* is used as a model organism for the study of plant cell biology, particularly differentiation at the cellular level and growth responses to environmental stimuli by single cells (Cove, 2005; Reski, 1998). A remarkable feature of *Physcomitrella* is its ability to incorporate transforming DNA at targeted sites within the genome by homologous recombination (Hofmann *et al.*, 1999; Schaefer and Zryd, 1997; Strepp *et al.*, 1998). Targeting vectors containing sequences as short as a few hundred base pairs are preferentially integrated at the cognate loci with frequencies similar to those observed in yeast (Kamisugi *et al.*, 2005, 2006). This has enabled the rapid construction and analysis of mutant strains containing precisely engineered allele replacements and gene knockouts for the functional analysis of candidate genes identified in the genome sequence by ‘reverse genetics’.

Reverse genetics permits the analysis of genes whose function may be postulated by virtue of their homology with genes previously identified in other organisms. However, the majority (63%) of predicted *P. patens* genes currently lack evidence of their function and homology (Rensing *et al.*, 2008). Therefore, the full potential of *Physcomitrella* will only be realised once a ‘forward genetic’ analysis of the organism is possible. Forward genetic approaches – the mutagenic interrogation of an organism to uncover genes responsible for specific phenotypes – enables novel genetic functions to be identified without *a priori* assumptions. It is an approach that is both powerful and philosophically satisfying, and in *Physcomitrella* mutagenesis is facilitated by the haploid nature of the dominant (gametophyte) stage of the life cycle.

Numerous *Physcomitrella* mutants have been isolated and described, with phenotypes associated with metabolism, cellular differentiation and hormone responses, and cellular-level growth responses such as gravi- and phototropisms (Abel *et al.*, 1989; Ashton and Cove, 1977; Ashton *et al.*, 1979a,b; Engel, 1968; Jenkins and Cove, 1983; Jenkins *et al.*, 1986; Knight *et al.*, 1991; Wang *et al.*, 1981). However, successful identification of the genes identified through a random mutagenic approach requires that a means exists whereby a researcher may move rapidly from an identified mutant phenotype to the underlying mutated DNA sequence. In most widely adopted model organisms, this requirement is satisfied by the existence of a completed genome sequence underpinned by a well-marked genetic linkage map populated by sequence-anchored genetic markers. This enables the rapid map-based cloning of genes responsible for mutant phenotypes (Jander *et al.*, 2002; Lukowitz *et al.*, 2000).

Remarkably, although genetic analysis of *Physcomitrella* mutants was established 40 years ago (Engel, 1968), no genetic linkage map for this species has previously been established. Moreover, cytogenetic studies have historically shown disagreement as to the number of chromosomes (which are small and difficult to visualise), but the current consensus is that for the internationally used laboratory strain ‘Gransden2004’*n* = 1*x* = 27 (Bryan, 1957; Reski *et al.*, 1994). In order to enable the integration of forward genetic approaches with the unrivalled capacity for reverse genetics that characterises *Physcomitrella*, we have constructed a high-density linkage map populated with molecular markers that can be directly integrated with the underlying sequence of the genome.

## Results

### Establishment of mapping populations

Our laboratories embarked on the identification of two classes of molecular genetic marker: amplified fragment length polymorphisms (AFLPs) (Vos *et al.*, 1995) and expressed sequence tag (EST)-derived simple sequence repeats (SSRs) (Tautz and Renz, 1984). The former provide a means of simultaneously determining the allelic status of hundreds of independent genetic loci without any prior knowledge of their DNA sequence, and are thus well suited to the rapid acquisition of mapped loci (Van Os *et al.*, 2006). The latter generate a framework of gene sequence-linked markers that provides a means by which the AFLP-based linkage map can be anchored to the genome sequence.

A number of independently isolated and geographically diverse *Physcomitrella* accessions were first screened for their relative genetic diversity, using gene-linked SSR markers identified following a computational analysis of the large *Physcomitrella* EST database, as well as by analysis of ribosomal DNA internal transcribed spacer sequences (von Stackelberg *et al.*, 2006). The genetic diversity of a collection of 21 worldwide *Physcomitrella* accessions and two related species in the Funariaceae was evaluated by testing 64 informative SSR markers, as well as by comparing ribosomal DNA internal transcribed spacer sequences (von Stackelberg *et al.*, 2006, and manuscript in preparation; http://www.cosmoss.org/ecomap.content). This analysis identified strains that could contribute the genetically distinct parental genotypes necessary for the generation of a mapping population. Because the object of the programme was to provide linkage between the genetic and physical maps, we chose the sequenced genotype (Gransden2004).

The French accession Villersexel K3 exhibited the greatest genetic distance from Gransden among all tested European lines. The ability to hybridise and produce viable progeny with a *nicB5*/*ylo6* male-sterile Gransden mutant (Ashton and Cove, 1977) was evaluated for the Villersexel strain and also for two genetically divergent Japanese and Australian accessions. However, crossing experiments with the accessions from Japan (*P. patens* ssp. *californica*) and Australia (*P. patens* ssp. *readeri*) failed to produce reasonable numbers of viable progeny. Consequently the Villersexel K3 strain was selected as the most suitable choice for the second parent.

Mapping populations were established in both Leeds and Freiburg. In Leeds, we used a Gransden2004 parent that had been transgenically marked by the targeted incorporation of a single copy of an *nptII* selection cassette to the *PpLea-1* (AY870926) locus (Kamisugi and Cuming, 2005). When crossed with the Villersexel accession, hybrid sporophytes were identified through the 1:1 segregation of the G418 resistance phenotype in their resulting spores. In Freiburg, the Villersexel parent was crossed as father with the *nicB5*/*ylo6* male-sterile Gransden mutant as mother. Successful crosses were identified by the appearance of segregating parental SSR alleles in F_1_ individuals.

Like all bryophytes, the dominant vegetative form of *Physcomitrella* is the haploid gametophyte. Gametes are produced by mitosis, and their fusion (the outcome of fertilization of a single egg cell within an archegonium by a spermatozoid) results in the formation of a diploid sporophyte that remains dependent on the gametophyte for its nourishment and development. Within the sporophyte, meiotic spore mother cells generate the haploid spores (typically 2000–5000 per spore capsule) (Reski, 1998). Recombination during these meiotic divisions therefore generates a population of segregating, recombinant haploid F_1_ progeny. The mapping lines used corresponded to the individual F_1_ spore-derived plants that were subsequently propagated vegetatively. Being haploid and self-fertile, these lines represent a population of plants in which the original recombination junctions are fixed in a single generation: equivalent to recombinant inbred lines in a diploid species.

### SSR analysis

**Figure 1:**
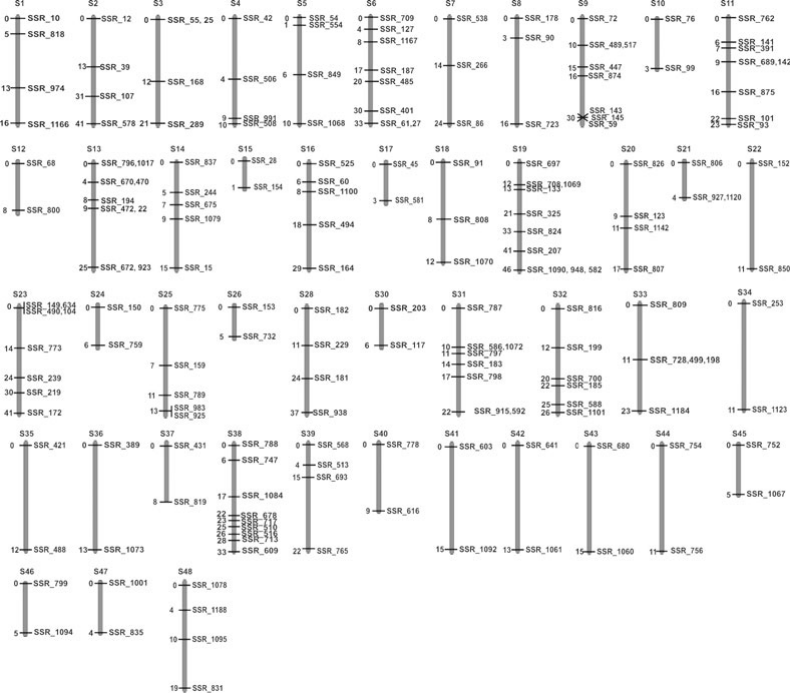
The simple sequence repeat (SSR)-based *Physcomitrella* linkage map. Forty-eight linkage groups were identified following genotyping of a population of 94 F_1_ recombinants. Linkage groups were determined by MapMaker using a LOD threshold of 6, a recombination threshold of 0.3 and Kosambi's mapping function. Linkage groups S27 and S29 are not shown, as the markers on these groups showed non-Mendelian segregation. Each linkage group is identified by a number (S1–S48), above each diagram. Linkage distance from the topmost marker is given in cM to the left of each linkage group, and the name of each marker to the right.

The SSR identification (Freiburg) was carried out as previously described (von Stackelberg *et al.*, 2006). In total, 3723 microsatellites were identified in a non-redundant *Physcomitrella* EST database and their utility as molecular markers was tested. F_1_ progeny selected from six independent spore capsules resulting from Villersexel K3 × Gransden crosses were chosen as the mapping population. Initially, 1238 microsatellite loci were examined for polymorphisms by PCR. Subsequently, the allelic status of 256 SSR markers was scored in a mapping population of 94 F_1_ recombinant haploid lines. Computational linkage analysis and map construction was performed with MapMaker (Lander *et al.*, 1987). We identified 46 linkage groups (S1–S46) containing two or more SSR markers at a logarithm of the odds (LOD) threshold of 6 (Figure 1). The largest number of markers per linkage group was 10, with a map length of 46.4 cM, while there were 20 linkage groups containing only two SSR markers with an average length of 8.2 cM. In total 179 markers were mapped to a total map length (McDaniel *et al.*, 2007) of 1168–1401 cM and an average map length of 25–30 cM per linkage group. Details of these SSRs are listed in (Appendix S1). The average marker density of the maps is one every 4.3 cM. However, these values must be treated cautiously, due to the relatively small number of markers mapped, the calculated map length representing a 50% underestimate relative to the theoretical expectation of 2700 cM (based on an assumption of 27 chromosomes (Bryan, 1957; Reski *et al.*, 1994) with one crossover per chromosome arm).

### AFLP analysis

We determined the utility of AFLP markers (Leeds) to discriminate between the Gransden and Villersexel genotypes. We used genomic DNA digested with *Eco*RI and *Mse*I and *Eco*RI and *Taq*I, respectively, for adapter ligation and amplification with primers containing two selective nucleotides each. In total, we tested 224 primer combinations, before selecting a subset of 39 primer pairs for genotyping a mapping population. High-resolution electrophoresis of amplicons on a capillary sequencer enabled the identification of approximately 10 000 amplified loci (about 250 amplicons per primer pair), of which 1574 were polymorphic (Table 1).

**Table 1:** Primers used to identify AFLP loci

Primers (E-FAM)	Loci	Primers (E-HEX)	Loci	Primers (E-NED)	Loci
EGA_MGA	48	ECA_MCG	40	ECG_MCG	12
EGA_MGC	38	ECA_MCC	39	ECG_MCC	16
EAC_MCC	47	ECA_MGG	56	ECG_MTA	38
EAC_MGA	55	EAG_MTA	36	ECG_MGA	20
EAC_MTC	89	ECA_MCT	59	ECG_MTT	32
EAC_MCG	38	EAG_MGG	40	ECG_MGC	26
EAC_MGG	67	EAG_MCT	47	ECG_MGT	21
EAC_MGC	66	EAG_MCA	46	ECG_MCA	21
EAC_MGT	43	EAG_MTT	46	ECG_MGG	26
EAC_TCC	40	EAG_TTA	22	ECG_TGT	46
EAC_TAG	61	EAG_TCA	39	ECG_TAC	25
EAC_TGA	39	EAG_TGC	32	ECG_TCA	19
EAC_TTC	60	EAG_TAA	32	ECG_TGA	47

Primer combinations are indicated by restriction enzyme site (E = *Eco*RI, M = *Mse*I, T = *Taq*I) and selective dinucleotide. The Eco-primers were labelled with FAM, HEX and NED as indicated. The number of polymorphic loci identified with each primer combination is indicated.

The allelic status of each polymorphic locus was scored in a mapping population of 188 F_1_ recombinant haploid lines and entered into JoinMap3.0 (Van Ooijen and Voorrips, 2001) for map construction. We initially obtained 26 linkage groups containing 20 or more markers at a LOD threshold of 10, comprising a total of 1220 mapped loci in a total map length of 4401 cM (not shown). This map was then integrated with the SSR-based map to enable anchorage with the genome sequence.

## Map integration

**Figure 2:**
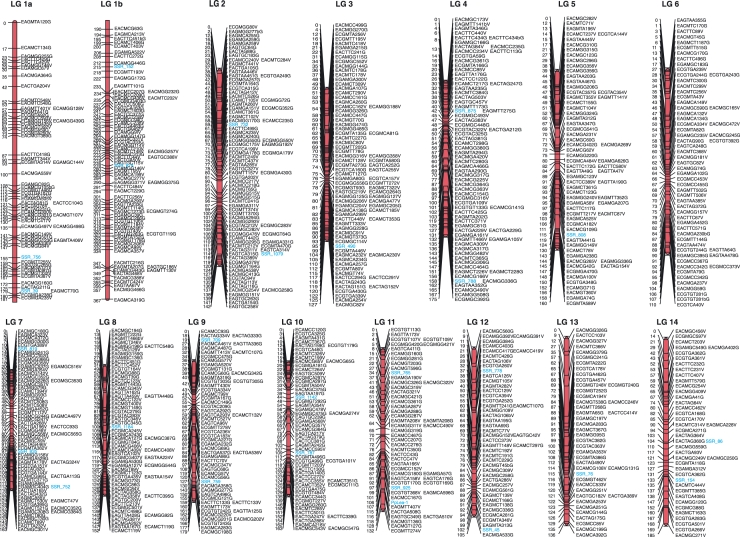

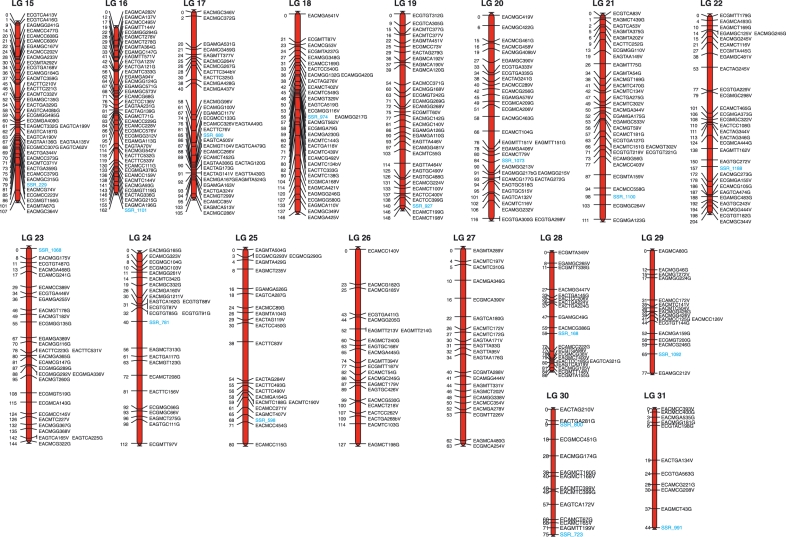
The integrated *Physcomitrella* linkage map Thirty-one linkage groups comprising 42 simple sequence repeat (SSR) markers and 1378 amplified fragment length polymorphism (AFLP) markers were obtained following genotyping of a population of 188 F_1_ recombinants. Data were analysed using JoinMap3.0. Linkage groups were determined using a LOD threshold of 8–10. Mapping parameters included a recombination threshold of 0.3, a ripple value of 1, a jump threshold of 5, and Kosambi's mapping function. Each linkage group is identified by a number (LG1-LG31), above each diagram. Linkage distance from the topmost marker is given in cM to the left of each linkage group, and the name of each marker to the right. The AFLP markers are systematically named according to the restriction sites used to generate fragments (E = *Eco*RI, M = *Mse*I, T = *Taq*I), the selective nucleotides, the length of the amplified fragment and the genotype from which the fragment was amplified (G = Gransden2004; V = Villersexel K3). Thus the first marker on LG1 (EAGMTA120G) corresponds to a 120-bp fragment amplified from the Gransden genotype, using primers recognising the *Eco*RI adapter with a selective AG dinucleotide and the *Mse*I adapter with a selective TA dinucleotide. The locations of SSR markers are indicated in blue, as is the *PpLEA-1* locus on LG11.

The AFLP loci are anonymous. However, because each SSR is derived from an expressed gene, each SSR marker can be identified within the genomic sequence scaffolds. The mapped SSR loci were found on 94 sequence scaffolds. These provided a starting point for integration between the two linkage maps and anchorage to the annotated genome sequence. For map integration we used primers corresponding to a single ‘signature’ SSR within each SSR linkage group to genotype the respective loci within the Leeds mapping lines, thereby placing these SSR loci within the AFLP linkage map. This resulted in an integrated map comprising 31 linkage groups (LG1–LG31), with an overall map length of 4410–4418 cM. Three pairs of linkage groups that were separate at LOD = 10 coalesced at lower LOD thresholds. (LG5 and LG16 coalesced at LOD = 7, but with insufficient linkage between the loci on the two groups to calculate a combined map. LG7 and LG30 and LG20 and LG23 coalesced at LOD = 8. The combined maps for these LGs are appended at the end of Figure S1. This would generate 28 linkage groups more closely approaching *n* = 27. The integrated map includes three linkage groups that contain fewer than 20 loci, but that include at least one signature SSR locus (Figure 2). We placed 1420 markers in this map, including 42 SSR markers. The largest linkage group comprised 143 markers with an aggregate linkage score of 367 cM. The estimated genome coverage is high, with 99.8% of the genome predicted to be within 10 cM of a mapped marker.

### Anchorage with the genome sequence

Integration of the maps provided the opportunity to convert AFLP markers into sequence characterised amplified length polymorphisms (SCALPs). The AFLPs comprise sequence-uncharacterised bands on gels. To be useful, it is essential to identify the underlying polymorphic DNA sequence. Typically, this is achieved by physical excision of a band from a gel, reamplification and sequence analysis (Dussle *et al.*, 2002). However, this is not easily achieved when bands are resolved by capillary sequencers. Alternatively, the sequences may be predicted by computational analysis of the sequenced genome. As polymorphic fragments, AFLPs occur (by definition) once in the genome sequence. They have ends defined by restriction sites plus the selective nucleotides, and are of a defined length. It is theoretically possible to identify such fragments within a well-characterised genome sequence, with a high level of confidence (Peters *et al.*, 2001). However, the first-draft *Physcomitrella* sequence assembly is still incomplete. Currently comprising 2106 scaffolds, additional ambiguity is introduced by stretches of undetermined sequences, of indeterminate length, within some contigs, represented as lengths of ‘NNNNNNNNNNN’ comprising about 5% of the genome sequence assembly. This does not permit unambiguous identification of AFLP marker sequences within the whole genome sequence assembly. Nevertheless, definitive association of an AFLP with a sequence scaffold provides a much improved likelihood of correct assignation. Integration of the AFLP-based map with a sequence-anchored SSR-based map enables such associations to be made.

We developed a Perl program, ‘SCALPHunter’, to propose candidate sequences within the *Physcomitrella* genome assembly based on these criteria. Because accurate determination of AFLP fragment lengths carries with it uncertainties (variable accuracy of fragment length determination in different sequencing eletropherograms, addition of terminal bases to some PCR products by Taq polymerase), SCALPHunter identifies all possible length variants within the range ±4 bp. The sequence scaffolds containing mapped SSRs were therefore interrogated by SCALPHunter to identify candidate AFLP sequences corresponding to the Gransden-specific markers with which the mapped SSRs co-segregated. In many cases, this identified several candidate genomic loci corresponding to a cluster of AFLP markers, linked to the SSR locus, enabling an initial integration between genetic linkage and underlying DNA sequence. The candidate AFLP sequences identified are listed in Appendix S2, and their map locations are illustrated in Figure S1.

### Verification of a SCALPHunter prediction

**Figure 3:**
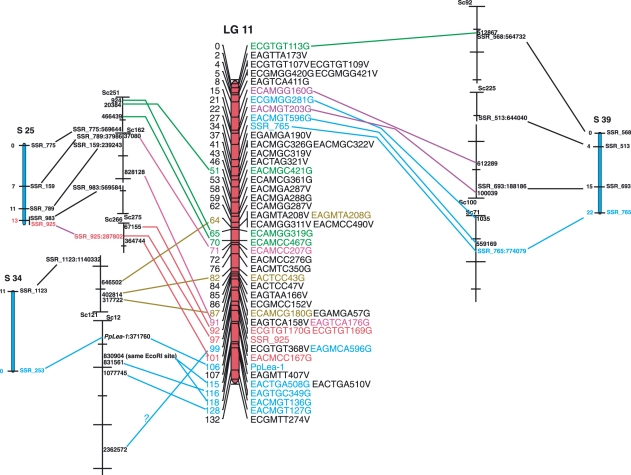
Anchoring the linkage map to the genome sequence: linkage group (LG) 11 Three simple sequence repeat (SSR) loci were mapped in LG11: SSR_925, SSR_253 (in the 5′-untranslated region of the *PpLEA-1* gene) and SSR_765. These loci lie in SSR linkage groups S25, S34 and S39, respectively. The ten genome sequence scaffolds containing the SSR markers comprising these linkage groups were searched for candidate amplified fragment length polymorphism loci using SCALPHunter, and the positions of these candidate sequences were identified in each scaffold by BlastN search. The sequence coordinates of the *Eco*RI site defining each candidate on the plus-strand of each sequence scaffold are shown. Note that only Gransden-specific alleles are identifiable.

The confidence with which SCALPs may be identified requires experimental verification. The predicted anchorage between the sequence and AFLP markers was therefore tested. As a proof of concept, we undertook the mapping of a *Physcomitrella* gene. The G418-resistant phenotype in the Gransden parent used to construct the Leeds mapping lines results from the targeted integration of a selection cassette into the *PpLEA-1* locus. This locus is present in sequence scaffold 12 of the v1.1 genome assembly. Segregation of the G418-resistant phenotype indicated that the gene was located at 106 cM on linkage group 11 of the integrated map (Figure 2). Coincidentally, SSR_253 defines a GT-dinucleotide repeat polymorphism within the 5′-untranslated region (UTR) of this gene, mapped in SSR-linkage group S34 (Figure 1). Linkage group 11 contains two additional signature SSR markers, SSR_765 and SSR_925, in S39 and S25, respectively, and 50 mapped AFLP markers. Of these, 29 derive from the Gransden genotype and are thus potentially identifiable by SCALPHunter. The three SSR-linkage groups contain 11 SSR markers, which correspond to sequences located on ten genome sequence scaffolds (scaffolds 12, 71, 92, 100, 121, 162, 225, 251, 266 and 275, respectively). Interrogation of these sequence scaffolds by SCALPHunter proposed candidate sequences for 21 of the 29 the Gransden-specific AFLP loci within the integrated LG11 (Figure 3).

The predicted sequences of candidate AFLP loci can be used to design primers with an extended selective sequence to amplify fragments from the parental genotypes. These fragments can be resolved electrophoretically to ascertain their genotype-specific nature, and sequenced to confirm their identity. We selected the locus EACTGA508G/EACTGA510V, at 115 cM on linkage group 11 (Figure 3), since this was represented by an allelic pair of fragments that differ by two base pairs and we anticipated that both the Gransden and Villersexel alleles would be amplifiable using AFLP-specific primers.

**Figure 4:**
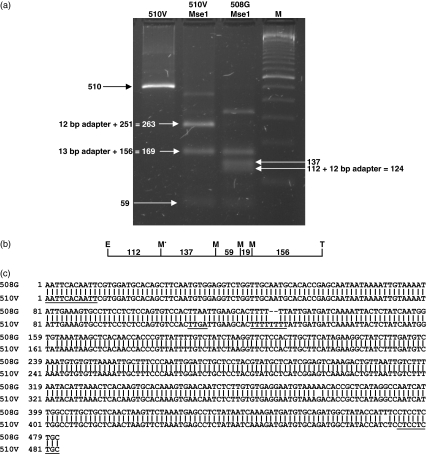
Verification of an amplified fragment length polymorphism (AFLP) locus identified using SCALPHunter Selective primers Eco-ACAATT and Taq-GAGGAG were used to amplify the allelic fragments EACTAG508G/EACTAG510V from adapter-ligated Gransden and Villersexel K3 genomic DNA. (a) Analysis of fragments by restriction enzyme digestion: Tracks from left show the ∼500-bp fragment amplified from the Villersexel genotype (510V); this fragment digested with *Mse*I (*Mse*I 510V); the corresponding Gransden fragment, digested with *Mse*I (*Mse*I 508G) and a ladder of size markers (M). The fragment amplified from the Gransden genotype contains an additional *Mse*I site, generating the genotype-specific AFLP marker ‘EACMGT136G’. (b) A restriction map of the amplified fragment. E = *Eco*RI, M = *Mse*I and T = *Taq*I. The Gransden-specific *Mse*I site is indicated as ‘M^*^’. (c) Sequence alignment of the fragments amplified from the Gransden genotype (‘508G’) and the Villersexel genotype (‘510V’). The sequences corresponding to the primer tails, the *Mse*I polymorphism and the 508/510 indel are underlined.

SCALPHunter identifies this locus as commencing at an *Eco*RI site that is the same as that identified for the closely linked locus EACMGT136G (118 cM). We designed +6 primers (Eco-ACAATT/Taq-GAGGAG) to amplify the allelic pair. Amplification generated a fragment of about 500 bp from each parental genotype (Figure 4a). Digestion with *Mse*I revealed an additional *Mse*I site in the fragment amplified from the Gransden genotype, the existence of which generates the Gransden-specific AFLP, EACMGT136G (Figure 4a,b). Sequence analysis of the genotype-specific amplified fragments revealed the causes of these polymorphisms (Figure 4c). A mutation A113G/G113V abolishes the *Mse*I site in the Villersexel genotype, whilst a 2-bp indel in the stretch of T-residues commencing at position 124 accounts for the 2-bp difference in fragment length between the Villersexel and Gransden alleles. Three additional SNPs were also observed in this fragment: G160G/A162V, A377G/G379V and T470G/C472V.

## Discussion

Genetic linkage maps are inevitably ‘works in progress’, their accuracy dependent on the resolution with which alleles may be scored and by the sizes of mapping populations. Their reliability increases as more loci are added, and only a comprehensive integration with a completely determined genome sequence will provide a true relationship between genetic and physical distance. We have initiated such an integration for *P. patens*. The incorporation of further sequence-anchored loci will refine this.

The overall integrated map length of 4410–4418 cM is almost certainly an overestimate. The molecular characterisation of two loci on LG11 (Figure 4) supports this view: the AFLP loci, EACTGA508G and EACMGT136G, share a common *Eco*RI site but are separated by 3 cM in the derived map. Because AFLPs are dominant markers, the allelic status of a locus being characterised by either the presence or absence of a band, map inflation is an inherent problem due to the difficulties of unambiguously scoring each locus (van Os *et al.*, 2005). ‘Allele drop-out’– the failure to amplify a single fragment – in a small mapping population can introduce significant error: increasing the number of co-dominant markers in future iterations of the map should resolve this.

In many cases, the order in which candidate AFLP sequences occur in the genome sequence scaffolds is consistent with the order of these markers in the linkage map, but there are also several instances where marker position appears to be inverted, or intercalated markers are observed. This might arise either through incorrect assembly of the sequence scaffolds, through the identification of an incorrect sequence by SCALPHunter or through incorrect scoring of segregation. This last cause most likely results in ambiguities in marker order over short linkage and sequence distances [for example, the intercalation of EAGTGC349G between EACTGA508G and EACMGT136G in LG11 (Figure 4)]. Where a marker appears to be inverted relative to others by a long interval (e.g. EAGMCA596G in LG11 relative to other candidate loci identified in sequence scaffold 12, or EACMGC421G in the same linkage group, relative to the other candidate loci in sequence scaffold 251 (Figure 4)], then the cause is most likely a misidentification by SCALPHunter. Such misidentifications will be inevitable, due to the length variation (±4 bp) built in to SCALPHunter predictions.

The addition of further sequence-anchored markers will also be necessary to refine the accuracy of prediction of AFLP loci by SCALPHunter. Currently, only 94 of the 2106 sequence scaffolds comprising the *Physcomitrella* version 1.1 sequence assembly are represented by mapped markers. These comprise about 143 Mbp, approximately 30% of the estimated complete genome sequence (Rensing *et al.*, 2008). Future development of the map will be based on additional SSR loci and single nucleotide polymorphisms (SNPs) located on unanchored sequence scaffolds. The ESTs generated in support of the first-draft genome sequence assembly include a catalogue of about 120 000 ESTs derived from the Villersexel parental genotype to provide a resource for the rapid identification of suitable SNP loci for future genotyping.

At present, the ease with which determination of gene function can be undertaken through homologous-recombination-mediated gene targeting recommends *Physcomitrella* as a powerful tool for ‘reverse genetic’ analysis of plant gene function. However, in order to identify novel genes essential for the normal development of *Physcomitrella*, which may be characteristic of the earliest land plants, and which may be expected to inform our understanding of land plant evolution, it is essential to be able to undertake ‘forward’ genetic analysis: the identification of genes from their mutant phenotypes. The availability of a well-marked genetic linkage map directly related to the underlying genome sequence will provide a platform from which the map-based cloning of genes identified through mutagenesis can be undertaken.

## Experimental procedures

### Plant material

*Physcomitrella patens* (Hedw.) B.S.G. plants were vegetatively propagated on agar medium as previously described [Knight *et al.,* 2002; Bierfreund *et al.,* 2003]. The ‘Gransden’ isolate was derived from a single spore collected by H. L. K. Whitehouse near Gransden Wood, Cambridgeshire, UK in 1962. The ‘Villersexel K3’ strain was collected from Villersexel, Villers la Ville, Haute Saône, France. Crosses between the two genotypes used the Villersexel strain as the male parent and defined mutant strains of the Gransden strain as the female parent. In Leeds, a transgenic strain of the Gransden isolate was constructed by transformation with an *nptII* cassette targeted to the *PpLEA-1* locus (Kamisugi and Cuming, 2005), conferring resistance to G418. This strain was confirmed as containing a single copy of the selectable marker at this locus by Southern blot analysis (Kamisugi *et al.*, 2005). In Freiburg, the Gransden parent was a self-sterile nicotinic acid-requiring auxotrophic mutant *nicB5*/*ylo6* (Ashton and Cove, 1977). Four to five plantlets of each parent were inoculated in a mixed stand adjacent to one another on solid medium and grown under inductive conditions for sporophyte development (Ashton and Cove, 1977). Mature spore capsules from the Gransden parent were surface-sterilised and the spores were liberated by gently crushing in sterile water. Spores were germinated and the progeny screened for G-418 resistance (Leeds) or SSR segregation (Freiburg). A 1:1 segregation indicated that the progeny derived from a hybrid sporophyte. Individual plants (188 Leeds; 94 Freiburg) were maintained as mapping populations by vegetative propagation.

### DNA isolation and analysis

Genomic DNA was isolated as described previously for AFLP and SSR analysis (AFLP, Knight *et al.*, 2002; SSR, von Stackelberg *et al.*, 2006). The SSR analysis was performed as previously described (von Stackelberg *et al.*, 2006). The primers used and the designation of the individual SSRs are listed in Table S1. For AFLP analysis, we followed the protocols established by Myburg *et al.* (2001). DNA (100 ng) was digested with *Eco*RI and *Mse*I, or with *Eco*RI and *Taq*I, prior to ligation with adapter oligonucleotides (Myburg *et al.*, 2001). DNA was pre-amplified using primers containing an additional selective nucleotide (+1 primers: *Eco*RI, GACTGCGTACCAATTCN; *Mse*I, GATGAGTCCTGAGTAAN; *Taq*I, GATGAGTCCTGAGCGAN). The PCR comprised 28 cycles of 15 sec at 94°C, 30 sec at 60°C, 1 min + 1 sec per cycle at 72°C followed by 1 cycle of 2 min at 72°C. Pre-amplified DNA (diluted 50-fold) was selectively amplified using the same primer sequences but containing two selective nucleotides (+2 primers). The *Eco*RI primer was labelled with one of three fluorescent dyes: FAM, HEX (Operon Biotechnologies, https://www.operon.com/) and NED (Applied Biosystems, http://www.appliedbiosystems.com/). The PCR was performed with 13 cycles of 10 sec at 94°C, 30 sec at 65 −0.7°C per cycle, 1 min at 72°C followed by 25 cycles of 10 sec at 94°C, 30 sec at 56°C, 1 min + 1 sec per cycle at 72°C. Following amplification, DNA was recovered by ethanol precipitation and redissolved in water. For electrophoretic analysis, three differently labelled sets of amplification products were multiplexed by mixing in the ratio FAM:HEX:NED = 1:2:2 supplemented with 0.5 μl of ROX-labelled size markers (MapMarker500, Cambio, http://www.cambio.co.uk/) and applied to an ABI 3130 Genetic Analyzer (Applied Biosystems). Chromatograms were analysed using the Applied Biosystems ‘Genemapper’ software, with the individual polymorphic peaks being scored manually and recorded in an Excel spreadsheet.

### Linkage analysis

The allelic status of 256 SSR markers was scored in a mapping population of 94 F_1_ recombinant haploid lines (1 × 96-well plates, including DNA from the parental genotypes in two wells). Computational linkage analysis and map construction was performed with MapMaker (Lander *et al.*,1987) using Kosambi's mapping function for distance estimation, a LOD threshold of 6 and a maximal distance of 30 cM. The AFLPs were scored in a mapping population of 188 F_1_ recombinant haploid lines (2 × 96-well plates, each including DNA from the parental genotypes in two wells). Linkage analysis for the ALFPs was performed using the mapping software JoinMap 3.0 (Van Ooijen and Voorrips, 2001) with a LOD threshold of 8–10, a recombination threshold of 0.3, a ripple value of 1, a jump threshold of 5 and Kosambi's mapping function. Markers exhibiting segregation significantly different from a 1:1 ratio were excluded following χ^2^ testing. The same mapping parameters were used following scoring of signature SSR loci within the AFLP linkage mapping population, to generate an integrated map.

### Map length calculation

We followed the approach of McDaniel *et al.* (2007) in calculating map lengths using two methods: that of Fishman *et al.* (2001) in which total map length *L* = Σ[(linkage group length) + 2(linkage group length/no. markers)] and Chakravarti *et al.* (1991), in which *L* = Σ[(linkage group length) × (no. markers + 1)/(no. markers − 1)]. Similarly, genome coverage (*c*) was calculated as *c* = 1 − exp(2*dn*/*L*) where *d* is distance in cM, *n* is the number of markers and *L* is map length.

### SCALPHunter

SCALPHunter is a Perl script that, given a list of AFLPs, identifies the possible candidate sequences within a given set of scaffolds. It can run 800 AFLP sequences across the whole *Physcomitrella* genome in about 30 min (AMD dual core 4400+ 2 GB RAM). The basic process is as follows:

- SCALPHunter reads in a list of codes describing AFLPs (length after subtraction of primers and end sequence), which provides the information for pattern matching against the scaffolds. The forward- and reverse-complement regular expressions are precompiled and placed upon a hash (using precompiled regular expressions greatly speeds up the process of searching for the existence of a list of AFLPs amongst a set of scaffolds). In addition, the regular expressions include a user-defined length variation (e.g. ±4 bp) to allow for the variable accuracy of fragment length determination.
- Each scaffold is searched in turn using all of the precompiled AFLP regular expressions and all matches are stored.
- The complete set of matches is sorted by size (allowing for variation of ±4 bp) and output in three different forms. Firstly, a set of matches-per-scaffold, showing all of the AFLPs found to match a scaffold given the pattern/length-variation criteria. In addition, a set of matches-per-AFLP, showing all of the scaffolds that an AFLP is found to match, and the corresponding sequences within those scaffolds. Finally, a list of the matches-per-length-variation, showing all of the AFLPs that matched scaffolds for a given sequence-length variation.

To identify candidate AFLPs within a scaffold identified as carrying an SSR, the matches-per-scaffold file was interrogated with each AFLP within the corresponding linkage group. The sequence of each candidate was obtained from the matches-per-AFLP file and its position was located within the scaffold by Blast search of the *Physcomitrella* genome.

## Supporting Information

**Figure S1**. The anchored Physcomitrella patens linkage map.

**Appendix S1**. Candidate amplified fragment length polymorphism sequences.

**Appendix S2**. Segregation data.

**Table S1**. Simple sequence repeat marker information.
