# Supplementary material for: A sequence-anchored genetic linkage map for the moss, Physcomitrella patens
Source: Plant J. 2008;56(5):855–66. doi: 10.1111/j.1365-313X.2008.03637.x (PMC2667646; doi:10.1111/j.1365-313X.2008.03637.x)
Supplement: Supplementary file 1 [file tpj0056-0855-SD1.pdf]

Supplementary Figure 1: The anchored *Physcomitrella patens* linkage map

The SSR-linkage groups ("S 1" *etc.*) are shown as blue bars, with each "signature" SSR locus labelled in colour. The integrated AFLP+SSR linkage groups ("LG 1" *etc.*) are shown as red bars. Genetic distances are in Cm. The sequence scaffolds are indicated as scaled lines ("ScN", with 0.5Mbp intervals) between the SSR and integrated linkage groups. The sequence coordinates of the remaining SSR markers, and of each candidate AFLP locus proposed by SCALPHunter (where the sequence coordinate corresponds to the first base of the *Eco*RI site), are indicated on the sequence scaffold. In each linkage group, candidate AFLP loci identified in the same sequence scaffold are highlighted in identical colours. Note that individual diagrams are not provided for LG 6, LG 26 and LG 27, since these linkage groups do not contain an SSR marker and thus cannot be anchored to the sequence scaffolds. At the end of the figure are two additional maps, based on coalescence of LG 7 and LG30, and of LG 20 and LG 23 at LOD = 8.

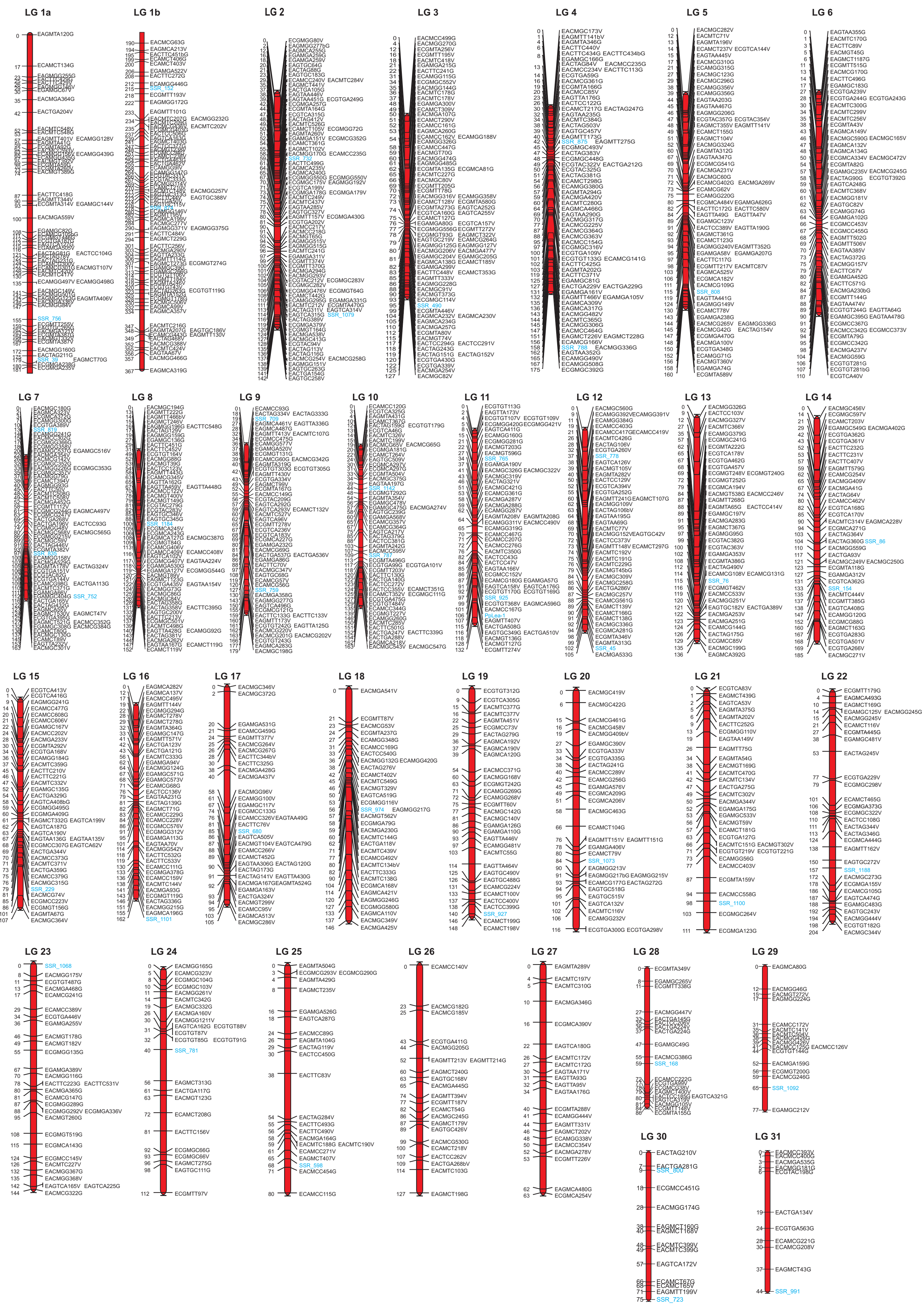

LG 1a
LG 1b
LG 2
LG 3
LG 4
LG 5
LG 6
LG 7
LG 8
LG 9
LG 10
LG 11
LG 12
LG 13
LG 14
LG 15
LG 16
LG 17
LG 18
LG 19
LG 20
LG 21
LG 22
LG 23
LG 24
LG 25
LG 26
LG 27
LG 28
LG 29
LG 30
LG 31

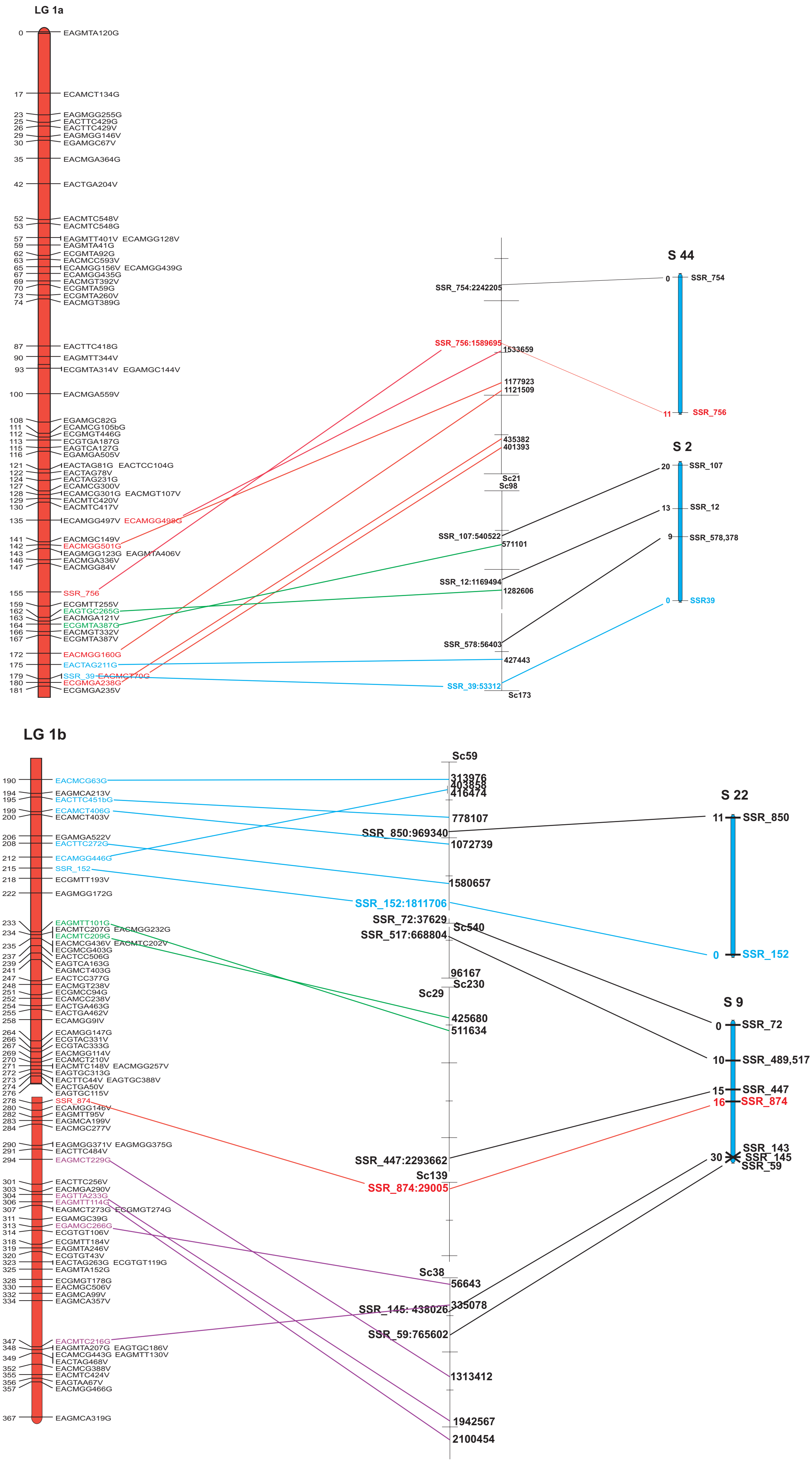

LG 1a
0 EAGMTA120G
17 ECAMCT134G
23 EAGMGG255G
25 EACTTC429G
26 EACTTC429V
29 EAGMGG146V
30 EGAMGC67V
35 EACMGA364G
42 EACTGA204V
52 EACMTC548V
53 EACMTC548G
57 EAGMTT401V ECAMGG128V
59 EAGMTA41G
62 ECGMTA92G
63 EACMCC593V
65 ECAMGG156V ECAMGG439G
67 ECAMGG435G
69 EACMGT392V
70 ECGMTA59G
73 ECGMTA260V
74 EACMGT389G
87 EACTTC418G
90 EAGMTT344V
93 ECGMTA314V EAGMGC144V
100 EACMGA559V
108 EGAMGC82G
111 ECAMCG105bG
112 ECGMGT446G
113 ECGTGA187G
115 EAGTCA127G
116 EGAMGA505V
121 EACTAG81G EACTCC104G
122 EACTAG78V
124 EACTAG231G
127 ECAMCG300V
128 ECAMCG301G EACMGT107V
129 EACMTC420V
130 EACMTC417V
135 ECAMGG497V ECAMGG498G
141 EACMGC149V
142 EACMGG501G
143 EAGMGG123G EAGMTA406V
146 EACMGA336V
147 EACMGG84V
155 SSR_756
159 ECGMTT255V
162 EAGTGC265G
163 EACMGA121V
164 ECGMTA387G
166 EACMGT332V
167 ECGMTA387V
172 EACMGG160G
175 EACTAG211G
179 SSR_39 EAGMCT70G
180 ECGMGA238G
181 ECGMGA235V
SSR_754:2242205
SSR_756:1589695
1533659
1177923
1121509
435382
401393
Sc21
Sc98
SSR_107:540522
571101
SSR_12:1169494
1282606
SSR_578:56403
427443
SSR_39:53312
Sc173
S 44
0 SSR_754
11 SSR_756
S 2
20 SSR_107
13 SSR_12
9 SSR_578,378
0 SSR39
LG 1b
190 EACMCG63G
194 EAGMCA213V
195 EACTTC451bG
199 ECAMCT406G
200 ECAMCT403V
206 EGAMGA522V
208 EACTTC272G
212 ECAMGG446G
215 SSR_152
218 ECGMTT193V
222 EAGMGG172G
233 EAGMTT101G
234 EACMTC207G EACMGG232G
EACMTC209G
235 EACMCG436V EACMTC202V
ECGMCG403G
237 EACTCC506G
239 EAGTCA163G
241 EAGMCT403G
247 EACTCC377G
248 EACMGT238V
251 ECGMCC94G
252 ECAMCC238V
254 EACTGA463G
255 EACTGA462V
258 ECAMGG9IV
264 ECAMGG147G
266 ECGTAC331V
267 ECGTAC333G
269 EACMGG114V
270 ECAMCT210V
271 EACMTC148V EACMGG257V
272 EAGTGC313G
273 EACTTC44V EAGTGC388V
274 EACTGA50V
276 EAGTGC115V
278 SSR_874
280 ECAMGG146V
282 EAGMTT95V
283 EAGMCA199V
284 EACMGC277V
290 EAGMGG371V EAGMGG375G
291 EACTTC484V
294 EAGMCT229G
301 EACTTC256V
303 EACMGA290V
304 EAGTTA233G
306 EAGMTT114G
307 EAGMCT273G ECGMGT274G
311 EGAMGC39G
313 EGAMGC266G
314 ECGTGT106V
318 ECGMTT184V
319 EAGMTA246V
320 ECGTGT43V
323 EACTAG263G ECGTGT119G
325 EAGMTA152G
328 ECGMGT178G
330 EACMGC506V
332 EAGMCA99V
334 EAGMCA357V
347 EACMTC216G
348 EAGMTA207G EAGTGC186V
349 ECAMCG443G EAGMTT130V
EACTAG468V
352 EACMCG388V
355 EACMTC424V
356 EAGTAA67V
357 EACMGG466G
367 EAGMCA319G
Sc59
313976
403858
416474
778107
SSR_850:969340
1072739
1580657
SSR_152:1811706
SSR_72:37629
SSR_517:668804
Sc540
96167
Sc230
Sc29
425680
511634
SSR_447:2293662
Sc139
SSR_874:29005
Sc38
56643
SSR_145: 438026
335078
SSR_59:765602
1313412
1942567
2100454
S 22
11 SSR_850
0 SSR_152
S 9
0 SSR_72
10 SSR_489,517
15 SSR_447
16 SSR_874
SSR_143
30 SSR_145
SSR_59

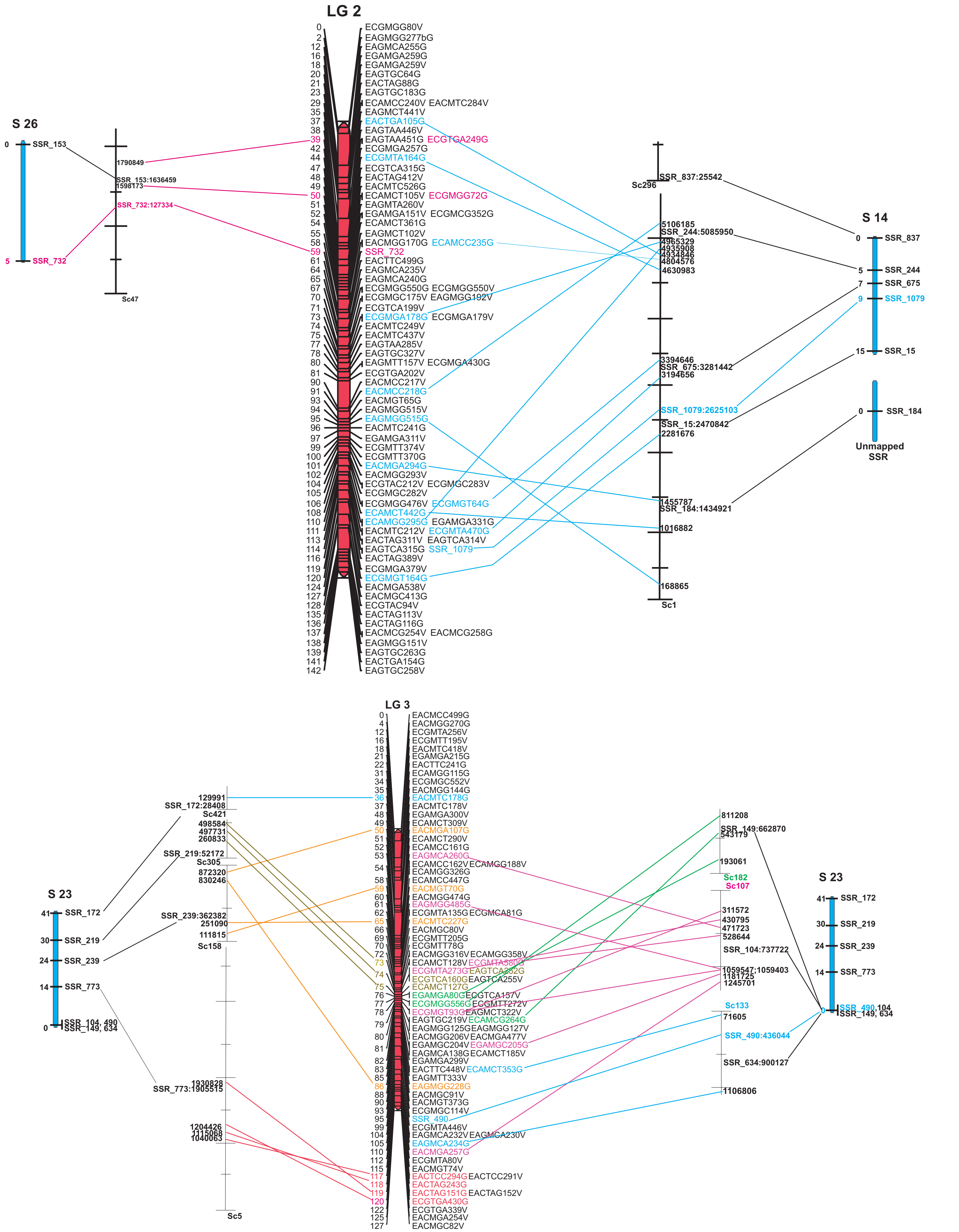
LG 2
S 26
SSR_153
SSR_732
Sc47
Sc296
Sc1
S 14
SSR_837
SSR_244
SSR_675
SSR_1079
SSR_15
SSR_184
Unmapped SSR
LG 3
S 23
SSR_172
SSR_219
SSR_239
SSR_773
Sc421
Sc305
Sc158
Sc5
Sc182
Sc107
Sc133

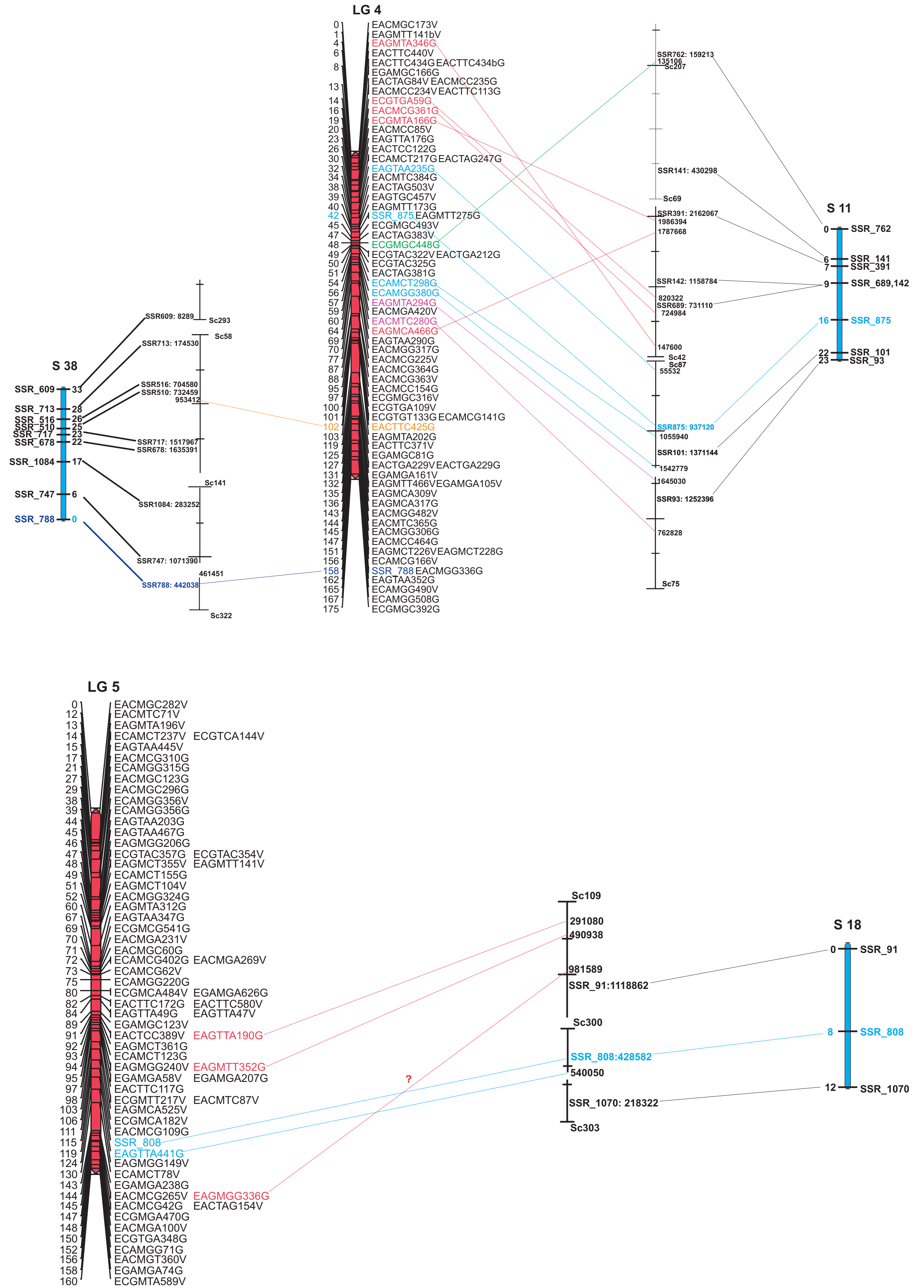

LG 4
0 EACMGC173V
1 EAGMTT141bV
4 EAGMTA346G
6 EACTTC440V
8 EACTTC434G EACTTC434bG
EGAMGC166G
EACTAG84V EACMCC235G
13 EACMCC234V EACTTC113G
14 ECGTGA59G
16 EACMCG361G
19 ECGMTA166G
20 EACMCC85V
23 EAGTTA176G
26 EACTCC122G
30 ECAMCT217G EACTAG247G
32 EAGTAA235G
34 EACMTC384G
38 EACTAG503V
39 EAGTGC457V
40 EAGMTT173G
42 SSR_875 EAGMTT275G
45 ECGMGC493V
47 EACTAG383V
48 ECGMGC448G
49 ECGTAC322V EACTGA212G
50 ECGTAC325G
51 EACTAG381G
54 ECAMCT298G
56 ECAMGG380G
57 EAGMTA294G
59 EACMGA420V
60 EACMTC280G
64 EAGMCA466G
69 EAGTAA290G
70 EACMGG317G
77 EACMCG225V
87 EACMCG364G
88 EACMCG363V
95 EACMCC154G
97 ECGMGC316V
100 ECGTGA109V
101 ECGTGT133G ECAMCG141G
102 EACTTC425G
103 EAGMTA202G
119 EACTTC371V
125 EGAMGC81G
127 EACTGA229V EACTGA229G
131 EGAMGA161V
132 EAGMTT466V EGAMGA105V
135 EAGMCA309V
136 EAGMCA317G
143 EACMGG482V
144 EACMTC365G
145 EACMGG306G
147 EACMCC464G
151 EAGMCT226V EAGMCT228G
156 ECAMCG166V
158 SSR_788 EACMGG336G
162 EAGTAA352G
165 ECAMGG490V
167 ECAMGG508G
175 ECGMGC392G
S 38
SSR_609 33
SSR_713 28
SSR_516 26
SSR_510 25
SSR_717 23
SSR_678 22
SSR_1084 17
SSR_747 6
SSR_788 0
SSR609: 8289
Sc293
SSR713: 174530
Sc58
SSR516: 704580
SSR510: 732459
953412
SSR717: 1517967
SSR678: 1635391
Sc141
SSR1084: 283252
SSR747: 1071390
461451
SSR788: 442038
Sc322
SSR762: 159213
135106
Sc207
SSR141: 430298
Sc69
SSR391: 2162067
1986394
1787668
SSR142: 1158784
820322
SSR689: 731110
724984
147600
Sc42
Sc87
55532
SSR875: 937120
1055940
SSR101: 1371144
1542779
1645030
SSR93: 1252396
762828
Sc75
S 11
0 SSR_762
6 SSR_141
7 SSR_391
9 SSR_689,142
16 SSR_875
22 SSR_101
23 SSR_93
LG 5
0 EACMGC282V
12 EACMTC71V
13 EAGMTA196V
14 ECAMCT237V ECGTCA144V
15 EAGTAA445V
17 EACMCG310G
21 ECAMGG315G
27 EACMGC123G
29 EACMGC296G
38 ECAMGG356V
39 ECAMGG356G
44 EAGTAA203G
45 EAGTAA467G
46 EAGMGG206G
47 ECGTAC357G ECGTAC354V
48 EAGMCT355V EAGMTT141V
49 ECAMCT155G
51 EAGMCT104V
52 EACMGG324G
60 EAGMTA312G
67 EAGTAA347G
69 ECGMCG541G
70 EACMGA231V
71 EACMGC60G
72 ECAMCG402G EACMGA269V
73 ECAMCG62V
75 ECAMGG220G
80 ECGMCA484V EGAMGA626G
82 EACTTC172G EACTTC580V
84 EAGTTA49G EAGTTA47V
89 EGAMGC123V
91 EACTCC389V EAGTTA190G
92 EAGMCT361G
93 ECAMCT123G
94 EAGMGG240V EAGMTT352G
95 EGAMGA58V EGAMGA207G
97 EACTTC117G
98 ECGMTT217V EACMTC87V
103 EAGMCA525V
106 ECGMCA182V
111 EACMCG109G
115 SSR_808
119 EAGTTA441G
124 EAGMGG149V
130 ECAMCT78V
143 EGAMGA238G
144 EACMCG265V EAGMGG336G
145 EACMCG42G EACTAG154V
147 ECGMGA470G
148 EACMGA100V
150 ECGTGA348G
152 ECAMGG71G
156 EACMGT360V
158 EGAMGA74G
160 ECGMTA589V
?
Sc109
291080
490938
981589
SSR_91:1118862
Sc300
SSR_808:428582
540050
SSR_1070: 218322
Sc303
S 18
0 SSR_91
8 SSR_808
12 SSR_1070

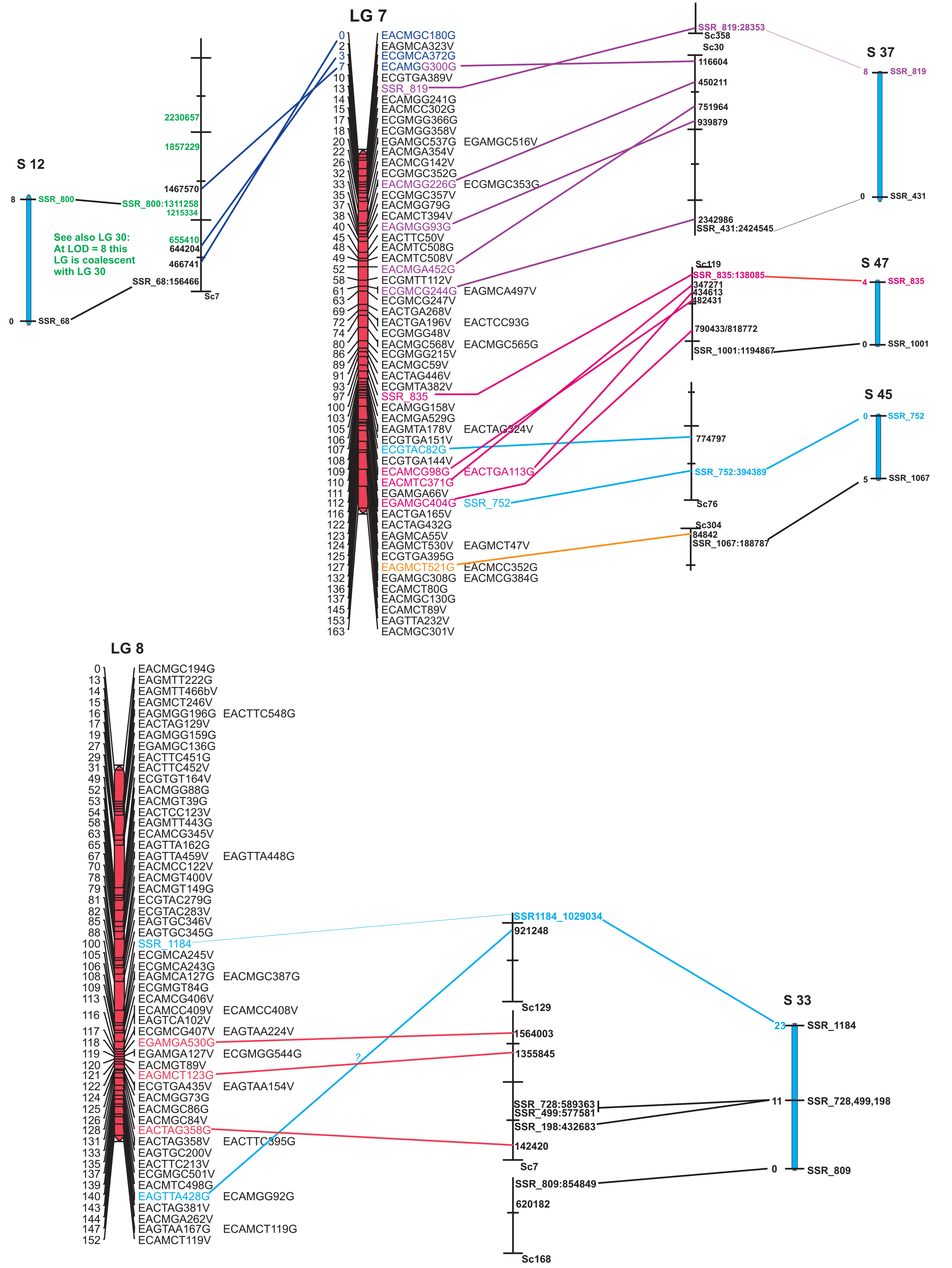
LG 7
LG 8
S 12
S 37
S 47
S 45
S 33
See also LG 30:
At LOD = 8 this
LG is coalescent
with LG 30
SSR_800
SSR_68
SSR_800:1311258
SSR_68:156466
Sc7
Sc358
Sc30
Sc119
Sc76
Sc304
Sc129
Sc168
SSR_819:28353
SSR_431:2424545
SSR_835:138085
SSR_1001:1194867
SSR_752:394389
SSR_1067:188787
SSR1184_1029034
SSR_728:589363
SSR_499:577581
SSR_198:432683
SSR_809:854849
SSR_819
SSR_431
SSR_835
SSR_1001
SSR_752
SSR_1067
SSR_1184
SSR_728,499,198
SSR_809

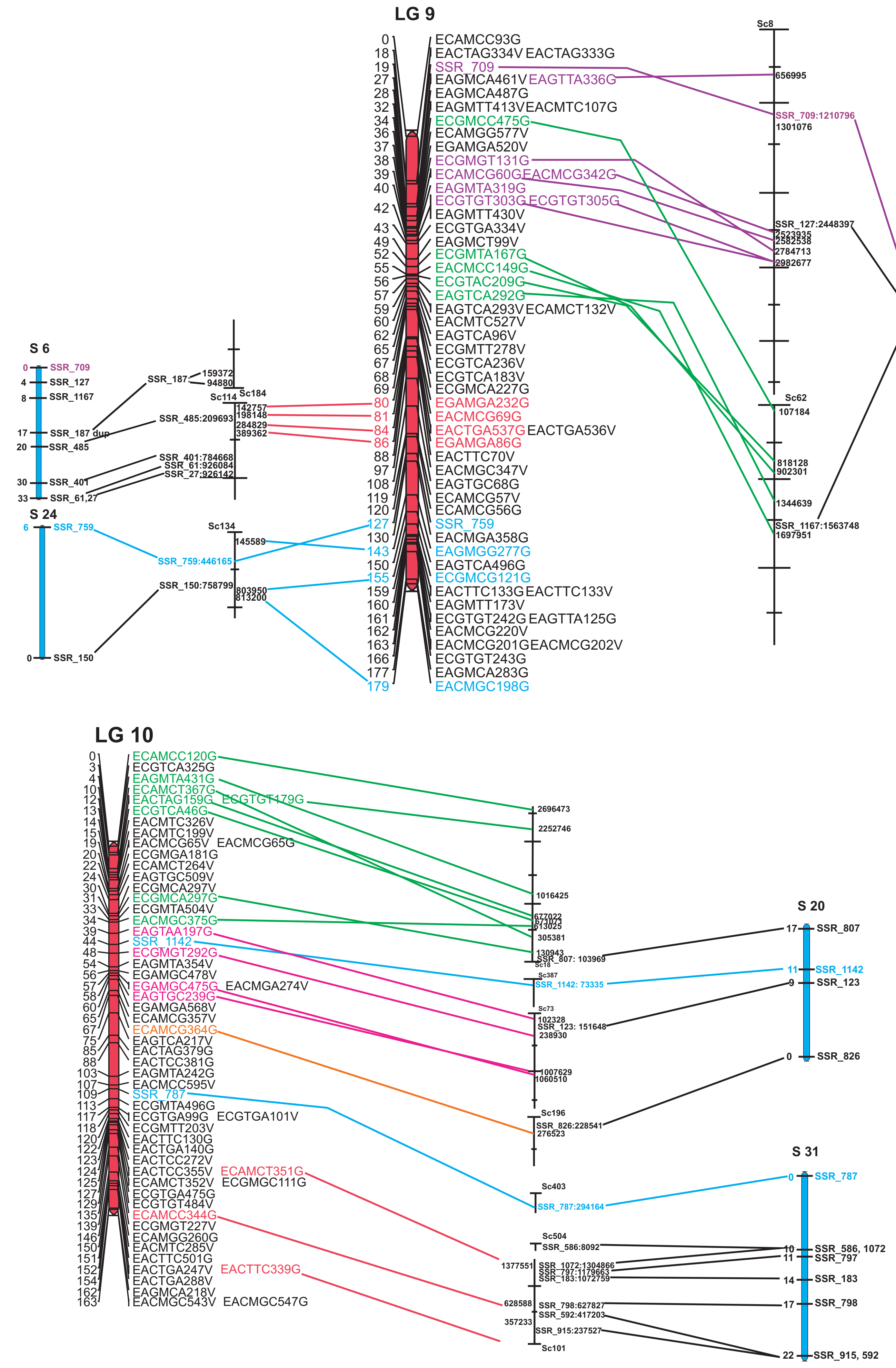

LG 9
0 ECAMCC93G
18 EACTAG334V EACTAG333G
19 SSR_709
27 EAGMCA461V EAGTTA336G
28 EAGMCA487G
32 EAGMTT413V EACMTC107G
34 ECGMCC475G
36 ECAMGG577V
37 EGAMGA520V
38 ECGMGT131G
39 ECAMCG60G EACMCG342G
40 EAGMTA319G
42 ECGTGT303G ECGTGT305G
EAGMTT430V
43 ECGTGA334V
49 EAGMCT99V
52 ECGMTA167G
55 EACMCC149G
56 ECGTAC209G
57 EAGTCA292G
59 EAGTCA293V ECAMCT132V
60 EACMTC527V
62 EAGTCA96V
65 ECGMTT278V
67 ECGTCA236V
68 ECGTCA183V
69 ECGMCA227G
80 EGAMGA232G
81 EACMCG69G
84 EACTGA537G EACTGA536V
86 EGAMGA86G
88 EACTTC70V
97 EACMGC347V
108 EAGTGC68G
119 ECAMCG57V
120 ECAMCG56G
127 SSR_759
130 EACMGA358G
143 EAGMGG277G
150 EAGTCA496G
155 ECGMCG121G
159 EACTTC133G EACTTC133V
160 EAGMTT173V
161 ECGTGT242G EAGTTA125G
162 EACMCG220V
163 EACMCG201G EACMCG202V
166 ECGTGT243G
177 EAGMCA283G
179 EACMGC198G
Sc8
656995
SSR_709:1210796
1301076
SSR_127:2448397
2523935
2582538
2784713
2982677
Sc62
107184
818128
902301
1344639
SSR_1167:1563748
1697951
S 6
0 SSR_709
4 SSR_127
8 SSR_1167
17 SSR_187 dup
20 SSR_485
30 SSR_401
33 SSR_61,27
S 6
0 SSR_709
4 SSR_127
8 SSR_1167
17 SSR_187 dup
20 SSR_485
30 SSR_401
33 SSR_61,27
SSR_187:
159372
94880
Sc184
Sc114
142757
198148
284829
389362
SSR_485:209693
SSR_401:784668
SSR_61:926084
SSR_27:926142
S 24
6 SSR_759
0 SSR_150
Sc134
145589
SSR_759:446165
SSR_150:758799
803950
813200
LG 10
0 ECAMCC120G
3 ECGTCA325G
4 EAGMTA431G
10 ECAMCT367G
12 EACTAG159G ECGTGT179G
13 ECGTCA46G
14 EACMTC326V
15 EACMTC199V
19 EACMCG65V EACMCG65G
20 ECGMGA181G
22 ECAMCT264V
24 EAGTGC509V
30 ECGMCA297V
31 ECGMCA297G
33 ECGMTA504V
34 EACMGC375G
39 EAGTAA197G
44 SSR_1142
48 ECGMGT292G
54 EAGMTA354V
56 EGAMGC478V
57 EGAMGC475G EACMGA274V
58 EAGTGC239G
60 EGAMGA568V
65 ECAMCG357V
67 ECAMCG364G
75 EAGTCA217V
85 EACTAG379G
88 EACTCC381G
103 EAGMTA242G
107 EACMCC595V
109 SSR_787
113 ECGMTA496G
117 ECGTGA99G ECGTGA101V
118 ECGMTT203V
120 EACTTC130G
122 EACTGA140G
123 EACTCC272V
124 EACTCC355V ECAMCT351G
125 ECAMCT352V ECGMGC111G
127 ECGTGA475G
129 ECGTGT484V
135 ECAMCC344G
139 ECGMGT227V
146 ECAMGG260G
150 EACMTC285V
151 EACTTC501G
152 EACTGA247V EACTTC339G
154 EACTGA288V
162 EAGMCA218V
163 EACMGC543V EACMGC547G
2696473
2252746
1016425
677022
671071
613025
305381
130943
SSR_807: 103969
Sc18
Sc387
SSR_1142: 73335
Sc73
102328
SSR_123: 151648
238930
1007629
1060510
Sc196
SSR_826:228541
276523
Sc403
SSR_787:294164
Sc504
SSR_586:8092
1377551
SSR_1072:1304866
SSR_797:1179663
SSR_183:1072759
628588
SSR_798:627827
357233
SSR_592:417203
SSR_915:237527
Sc101
S 20
17 SSR_807
11 SSR_1142
9 SSR_123
0 SSR_826
S 31
0 SSR_787
10 SSR_586, 1072
11 SSR_797
14 SSR_183
17 SSR_798
22 SSR_915, 592

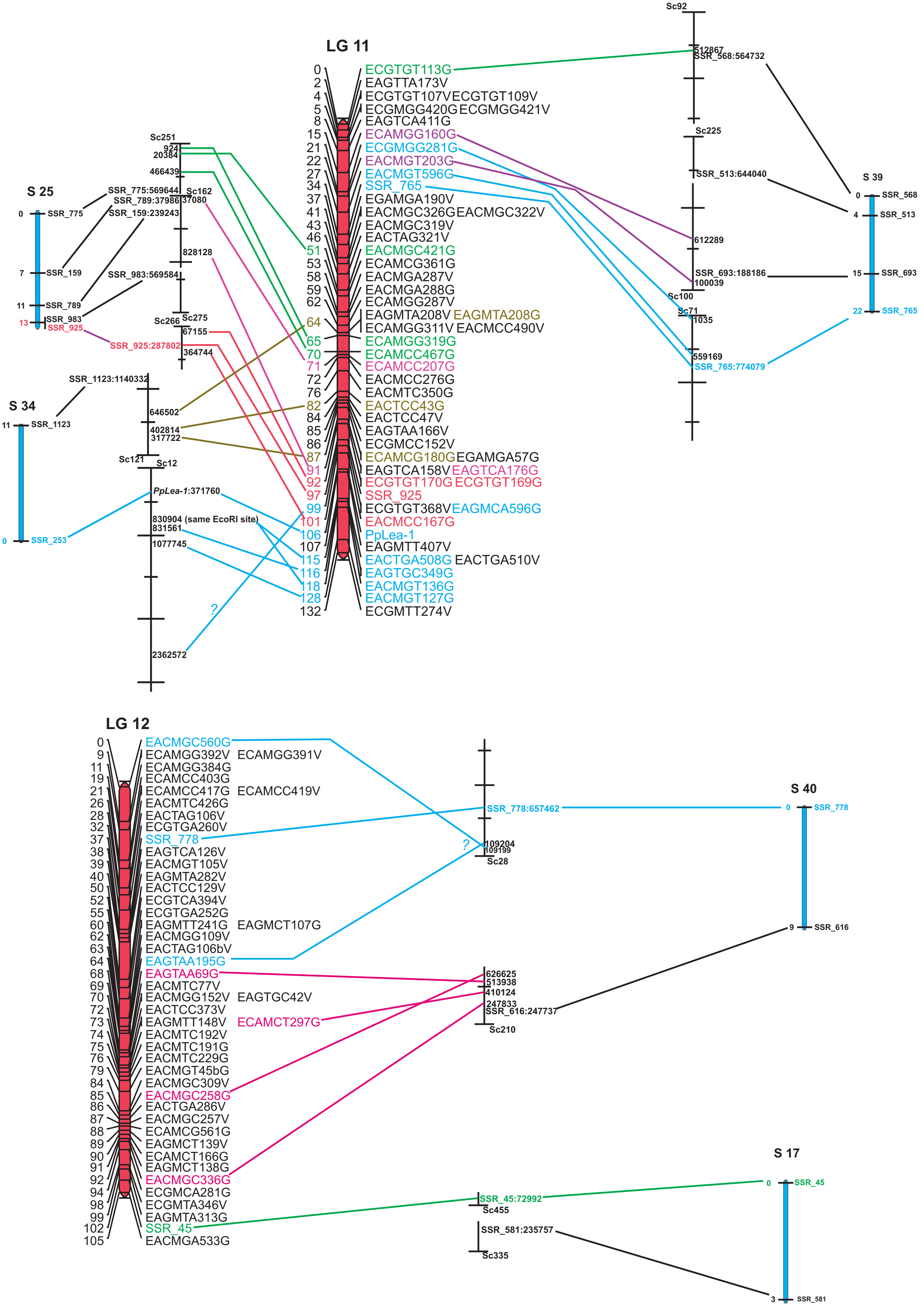

LG 11
0 ECGTGT113G
2 EAGTTA173V
4 ECGTGT107VECGTGT109V
5 ECGMGG420G ECGMGG421V
8 EAGTCA411G
15 ECAMGG160G
21 ECGMGG281G
22 EACMGT203G
27 EACMGT596G
34 SSR_765
37 EGAMGA190V
41 EACMGC326GEACMGC322V
43 EACMGC319V
46 EACTAG321V
51 EACMGC421G
53 ECAMCG361G
58 EACMGA287V
59 EACMGA288G
62 ECAMGG287V
64 EAGMTA208V EAGMTA208G
65 ECAMGG311V EACMCC490V
70 ECAMGG319G
71 ECAMCC467G
72 ECAMCC207G
76 EACMCC276G
82 EACMTC350G
84 EACTCC43G
85 EACTCC47V
86 EAGTAA166V
87 ECGMCC152V
91 ECAMCG180GEGAMGA57G
92 EAGTCA158VEAGTCA176G
97 ECGTGT170G ECGTGT169G
99 SSR_925
101 ECGTGT368VEAGMCA596G
106 EACMCC167G
107 PpLea-1
115 EAGMTT407V
116 EACTGA508G EACTGA510V
118 EAGTGC349G
128 EACMGT136G
132 EACMGT127G
ECGMTT274V
S 25
0 SSR_775
7 SSR_159
11 SSR_789
13 SSR_983
SSR_925
Sc251
924
20384
466439
SSR_775:569644
SSR_789:37986
SSR_159:239243
Sc162
37080
828128
SSR_983:569584
Sc266
Sc275
67155
SSR_925:287802
364744
S 34
11 SSR_1123
0 SSR_253
SSR_1123:1140332
646502
402814
317722
Sc121
Sc12
PpLea-1:371760
830904 (same EcoRI site)
831561
1077745
?
2362572
Sc92
512867
SSR_568:564732
Sc225
SSR_513:644040
612289
SSR_693:188186
100039
Sc100
Sc71
1035
559169
SSR_765:774079
S 39
0 SSR_568
4 SSR_513
15 SSR_693
22 SSR_765
LG 12
0 EACMGC560G
9 ECAMGG392V ECAMGG391V
11 ECAMGG384G
19 ECAMCC403G
21 ECAMCC417G ECAMCC419V
26 EACMTC426G
28 EACTAG106V
32 ECGTGA260V
37 SSR_778
38 EAGTCA126V
39 EACMGT105V
40 EAGMTA282V
50 EACTCC129V
52 ECGTCA394V
55 ECGTGA252G
60 EAGMTT241G EAGMCT107G
62 EACMGG109V
63 EACTAG106bV
64 EAGTAA195G
68 EAGTAA69G
69 EACMTC77V
70 EACMGG152V EAGTGC42V
72 EACTCC373V
73 EAGMTT148V ECAMCT297G
74 EACMTC192V
75 EACMTC191G
76 EACMTC229G
79 EACMGT45bG
84 EACMGC309V
85 EACMGC258G
86 EACTGA286V
87 EACMGC257V
88 ECAMCG561G
89 EAGMCT139V
90 ECAMCT166G
91 EAGMCT138G
92 EACMGC336G
94 ECGMCA281G
98 ECGMTA346V
99 EAGMTA313G
102 SSR_45
105 EACMGA533G
SSR_778:657462
?
109204
109199
Sc28
S 40
0 SSR_778
9 SSR_616
626625
513938
410124
247833
SSR_616:247737
Sc210
S 17
0 SSR_45
3 SSR_581
SSR_45:72992
Sc455
SSR_581:235757
Sc335

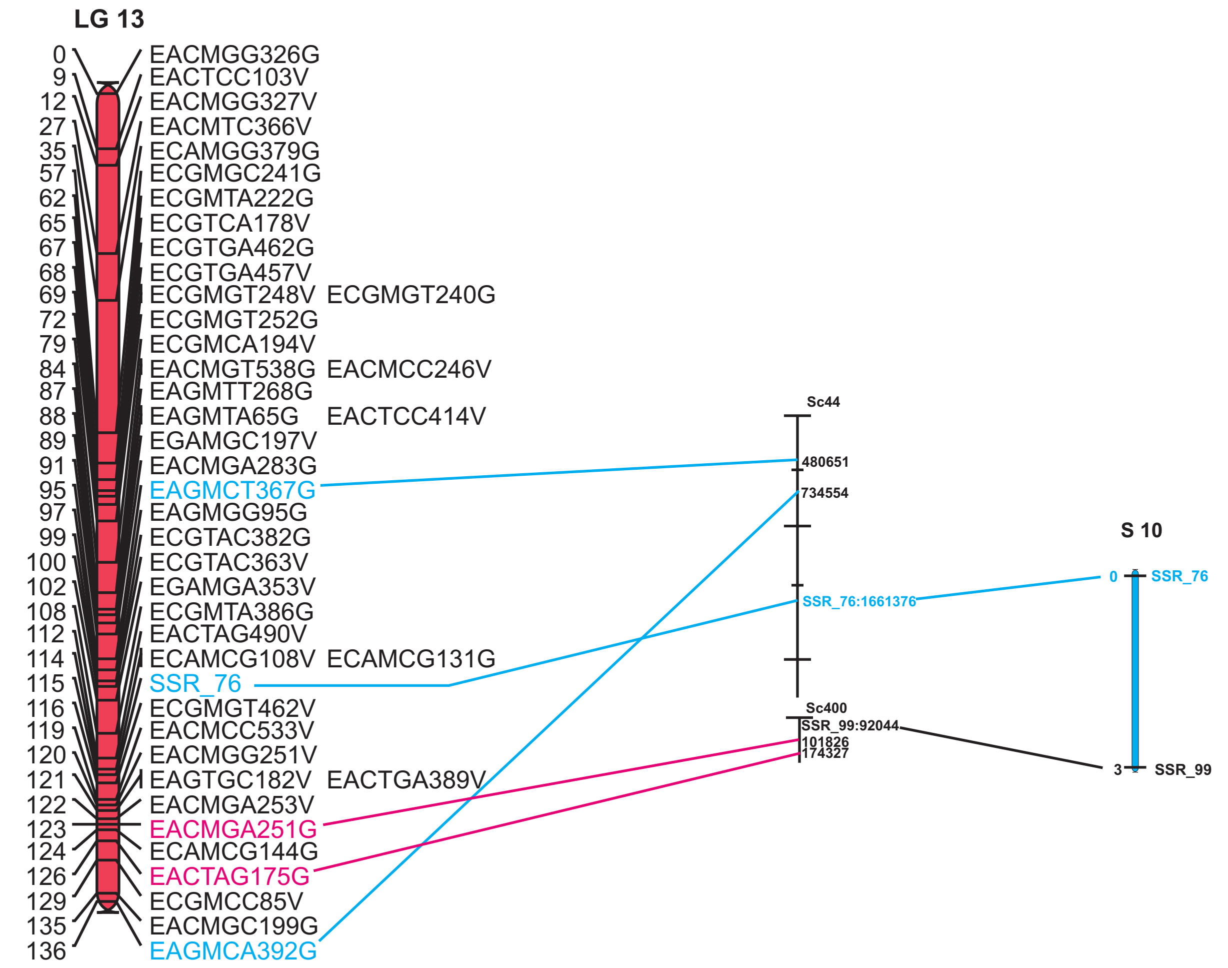
LG 13
0 EACMGG326G
9 EACTCC103V
12 EACMGG327V
27 EACMTC366V
35 ECAMGG379G
57 ECGMGC241G
62 ECGMTA222G
65 ECGTCA178V
67 ECGTGA462G
68 ECGTGA457V
69 ECGMGT248V ECGMGT240G
72 ECGMGT252G
79 ECGMCA194V
84 EACMGT538G EACMCC246V
87 EAGMTT268G
88 EAGMTA65G EACTCC414V
89 EGAMGC197V
91 EACMGA283G
95 EAGMCT367G
97 EAGMGG95G
99 ECGTAC382G
100 ECGTAC363V
102 EGAMGA353V
108 ECGMTA386G
112 EACTAG490V
114 ECAMCG108V ECAMCG131G
115 SSR_76
116 ECGMGT462V
119 EACMCC533V
120 EACMGG251V
121 EAGTGC182V EACTGA389V
122 EACMGA253V
123 EACMGA251G
124 ECAMCG144G
126 EACTAG175G
129 ECGMCC85V
135 EACMGC199G
136 EAGMCA392G
Sc44
480651
734554
SSR_76:1661376
Sc400
SSR_99:92044
101826
174327
S 10
0 SSR_76
3 SSR_99

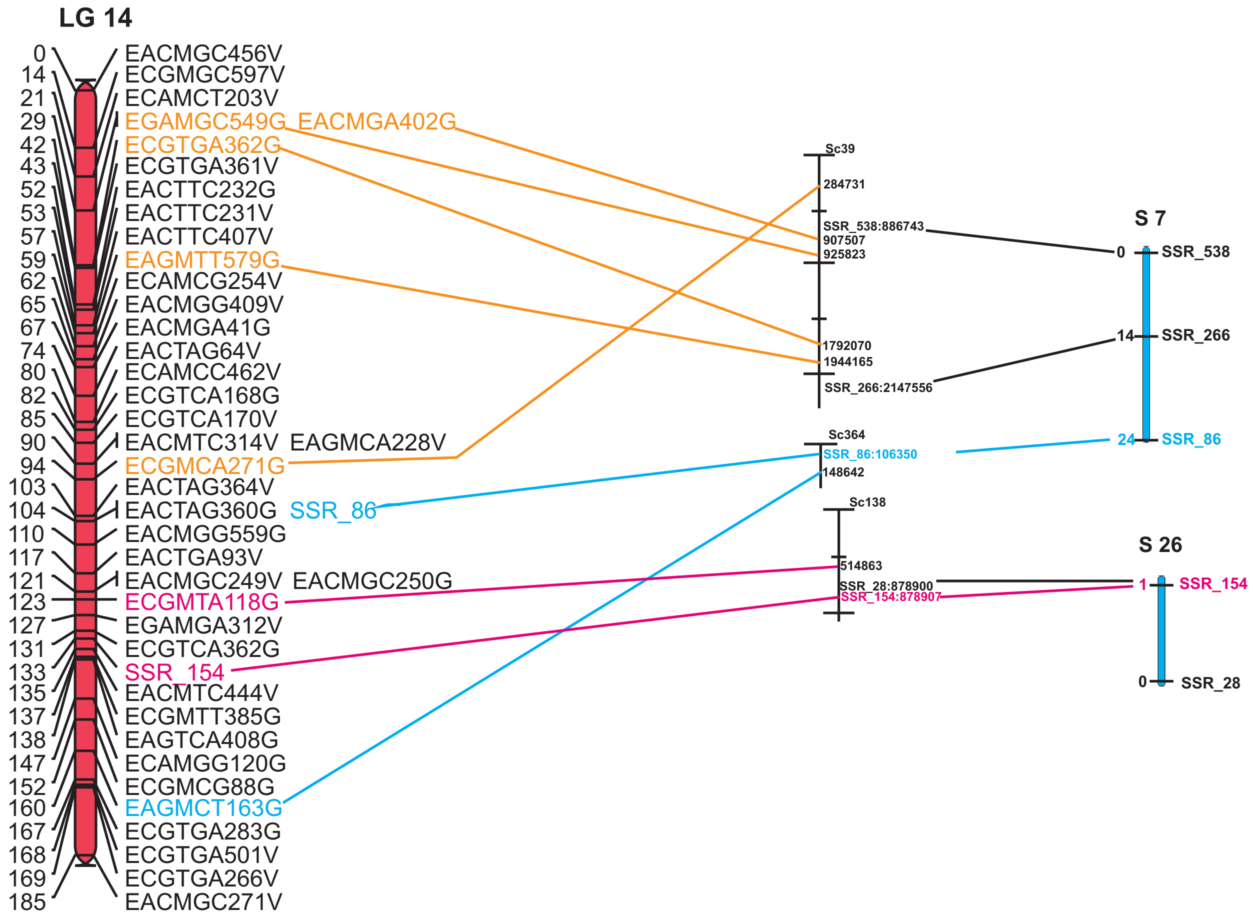
LG 14
0 EACMGC456V
14 ECGMGC597V
21 ECAMCT203V
29 EGAMGC549G EACMGA402G
42 ECGTGA362G
43 ECGTGA361V
52 EACTTC232G
53 EACTTC231V
57 EACTTC407V
59 EAGMTT579G
62 ECAMCG254V
65 EACMGG409V
67 EACMGA41G
74 EACTAG64V
80 ECAMCC462V
82 ECGTCA168G
85 ECGTCA170V
90 EACMTC314V EAGMCA228V
94 ECGMCA271G
103 EACTAG364V
104 EACTAG360G SSR_86
110 EACMGG559G
117 EACTGA93V
121 EACMGC249V EACMGC250G
123 ECGMTA118G
127 EGAMGA312V
131 ECGTCA362G
133 SSR_154
135 EACMTC444V
137 ECGMTT385G
138 EAGTCA408G
147 ECAMGG120G
152 ECGMCG88G
160 EAGMCT163G
167 ECGTGA283G
168 ECGTGA501V
169 ECGTGA266V
185 EACMGC271V
Sc39
284731
SSR_538:886743
907507
925823
1792070
1944165
SSR_266:2147556
Sc364
SSR_86:106350
148642
Sc138
514863
SSR_28:878900
SSR_154:878907
S 7
0 SSR_538
14 SSR_266
24 SSR_86
S 26
1 SSR_154
0 SSR_28

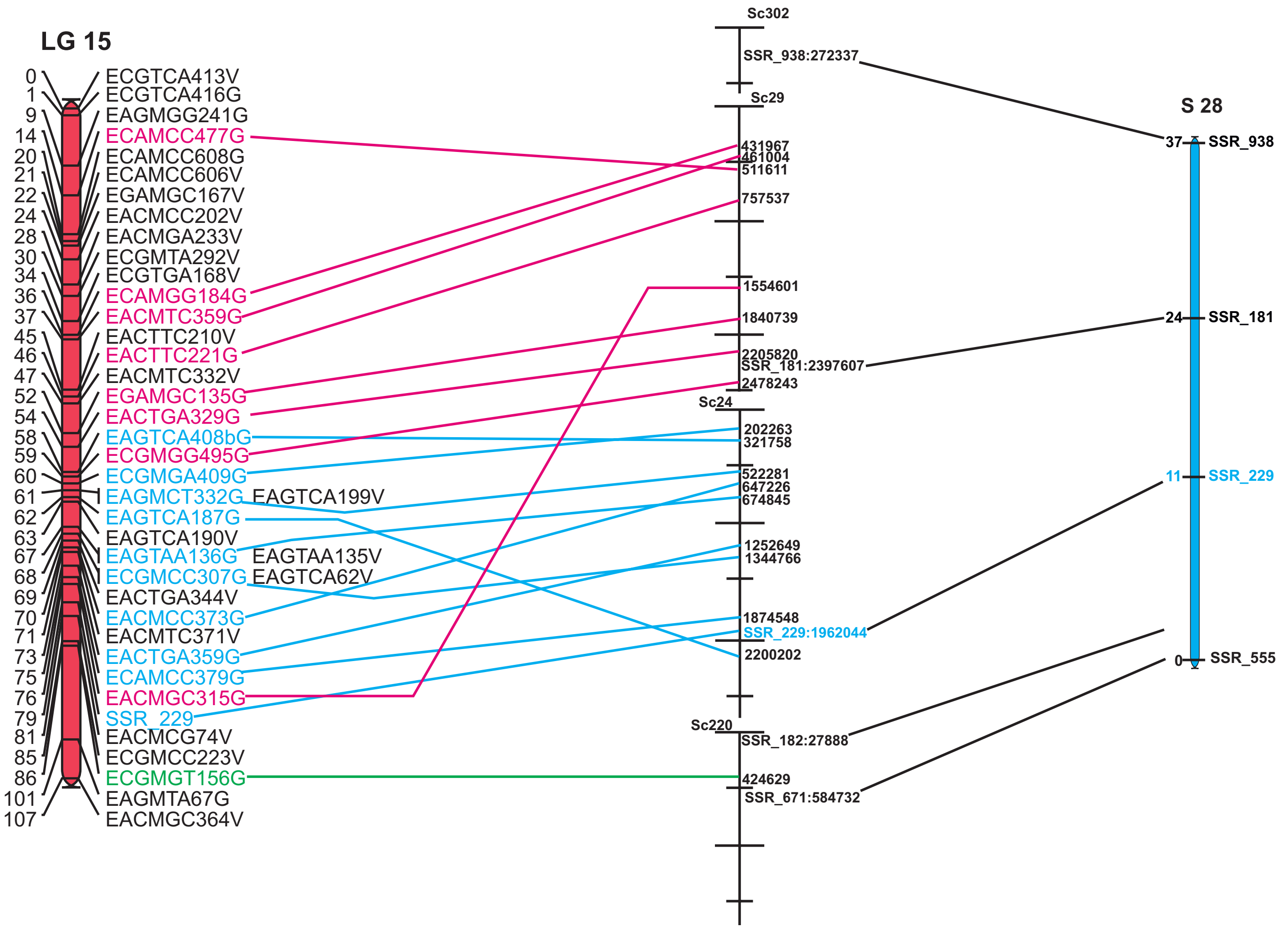
LG 15
0 ECGTCA413V
1 ECGTCA416G
9 EAGMGG241G
14 ECAMCC477G
20 ECAMCC608G
21 ECAMCC606V
22 EGAMGC167V
24 EACMCC202V
28 EACMGA233V
30 ECGMTA292V
34 ECGTGA168V
36 ECAMGG184G
37 EACMTC359G
45 EACTTC210V
46 EACTTC221G
47 EACMTC332V
52 EGAMGC135G
54 EACTGA329G
58 EAGTCA408bG
59 ECGMGG495G
60 ECGMGA409G
61 EAGMCT332G EAGTCA199V
62 EAGTCA187G
63 EAGTCA190V
67 EAGTAA136G EAGTAA135V
68 ECGMCC307G EAGTCA62V
69 EACTGA344V
70 EACMCC373G
71 EAMTC371V
73 EACTGA359G
75 ECAMCC379G
76 EACMGC315G
79 SSR_229
81 EACMCG74V
85 ECGMCC223V
86 ECGMGT156G
101 EAGMTA67G
107 EACMGC364V
Sc302
SSR_938:272337
Sc29
431967
461004
511611
757537
1554601
1840739
2205820
SSR_181:2397607
2478243
Sc24
202263
321758
522281
647226
674845
1252649
1344766
1874548
SSR_229:1962044
2200202
Sc220
SSR_182:27888
424629
SSR_671:584732
S 28
37 SSR_938
24 SSR_181
11 SSR_229
0 SSR_555

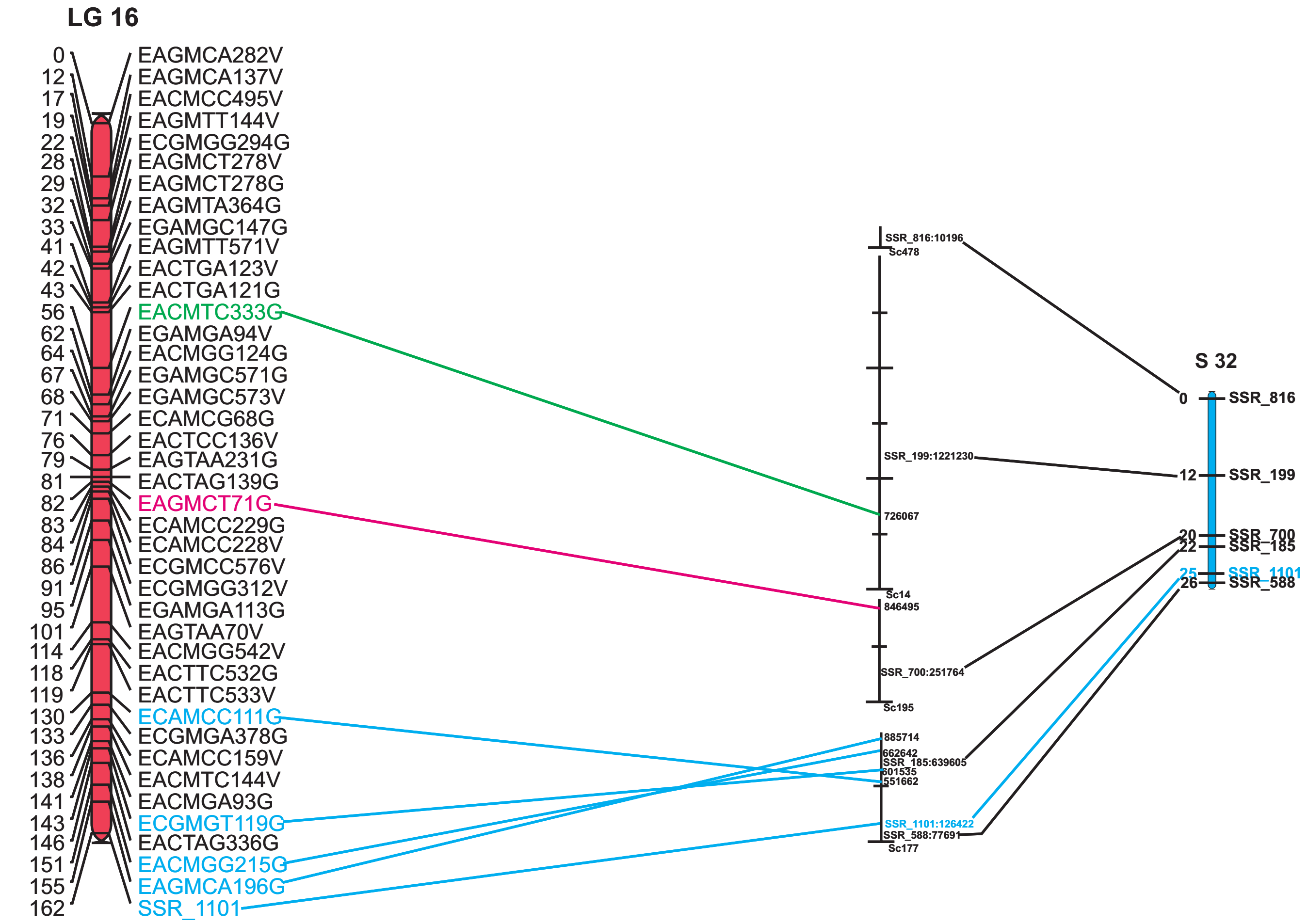
LG 16
0 EAGMCA282V
12 EAGMCA137V
17 EACMCC495V
19 EAGMTT144V
22 ECGMGG294G
28 EAGMCT278V
29 EAGMCT278G
32 EAGMTA364G
33 EGAMGC147G
41 EAGMTT571V
42 EACTGA123V
43 EACTGA121G
56 EACMTC333G
62 EGAMGA94V
64 EACMGG124G
67 EGAMGC571G
68 EGAMGC573V
71 ECAMCG68G
76 EACTCC136V
79 EAGTAA231G
81 EACTAG139G
82 EAGMCT71G
83 ECAMCC229G
84 ECAMCC228V
86 ECGMCC576V
91 ECGMGG312V
95 EGAMGA113G
101 EAGTAA70V
114 EACMGG542V
118 EACTTC532G
119 EACTTC533V
130 ECAMCC111G
133 ECGMGA378G
136 ECAMCC159V
138 EACMTC144V
141 EACMGA93G
143 ECGMGT119G
146 EACTAG336G
151 EACMGG215G
155 EAGMCA196G
162 SSR_1101
SSR_816:10196
Sc478
SSR_199:1221230
726067
Sc14
846495
SSR_700:251764
Sc195
885714
662642
SSR_185:639605
601535
551662
SSR_1101:126422
SSR_588:77691
Sc177
S 32
0 SSR_816
12 SSR_199
20 SSR_700
22 SSR_185
25 SSR_1101
26 SSR_588

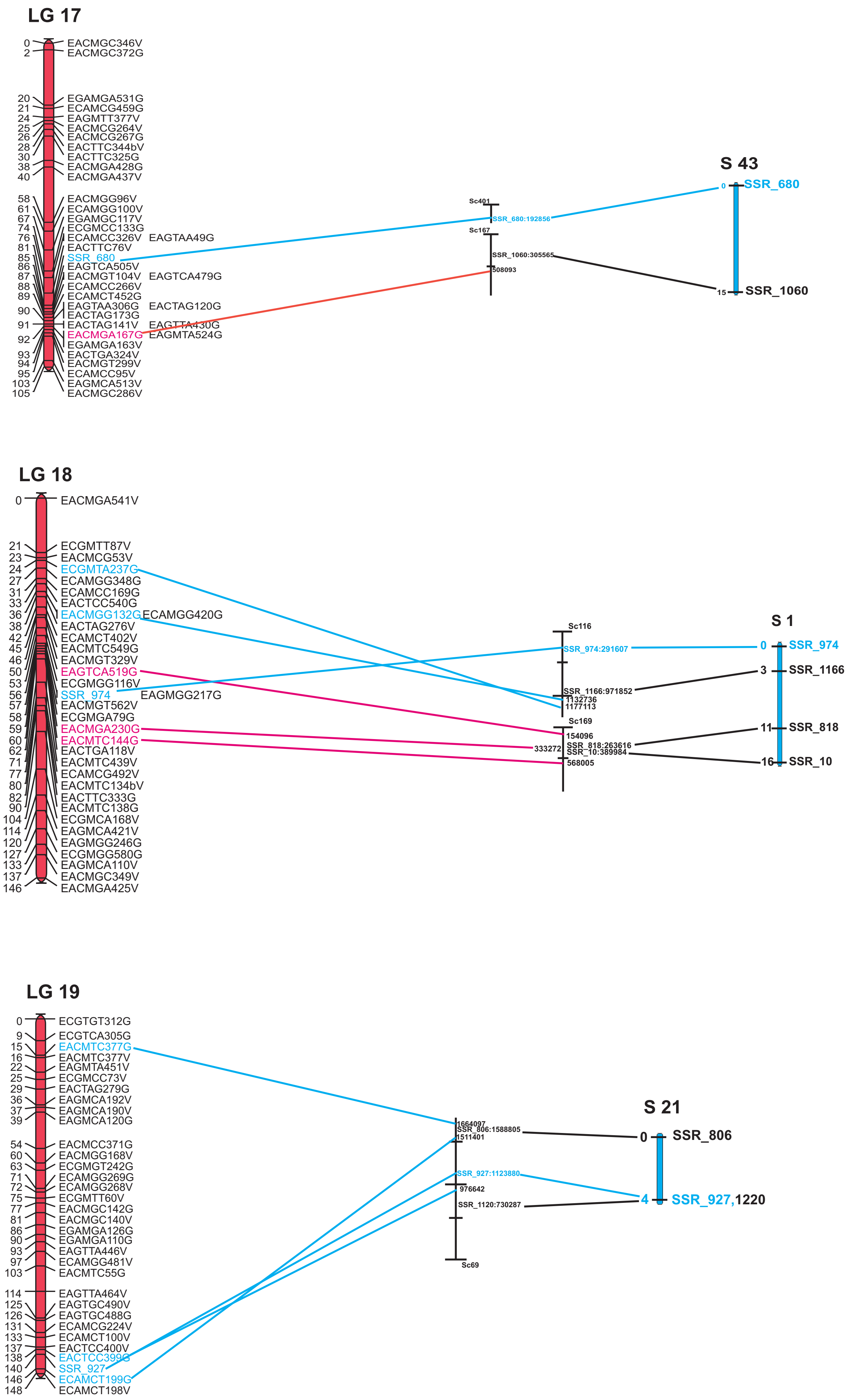
LG 17
0 EACMGC346V
2 EACMGC372G
20 EGAMGA531G
21 ECAMCG459G
24 EAGMTT377V
25 EACMCG264V
26 EACMCG267G
28 EACTTC344bV
30 EACTTC325G
38 EACMGA428G
40 EACMGA437V
58 EACMGG96V
61 ECAMGG100V
67 EGAMGC117V
74 ECGMCC133G
76 ECAMCC326V EAGTAA49G
81 EACTTC76V
85 SSR_680
86 EAGTCA505V
87 EACMGT104V EAGTCA479G
88 ECAMCC266V
89 ECAMCT452G
90 EAGTAA306G EACTAG120G
EACTAG173G
91 EACTAG141V EAGTTA430G
92 EACMGA167G EAGMTA524G
EGAMGA163V
93 EACTGA324V
94 EACMGT299V
95 ECAMCC95V
103 EAGMCA513V
105 EACMGC286V
Sc401
SSR_680:192856
Sc167
SSR_1060:305565
508093
S 43
0 SSR_680
15 SSR_1060
LG 18
0 EACMGA541V
21 ECGMTT87V
23 EACMCG53V
24 ECGMTA237G
27 ECAMGG348G
31 ECAMCC169G
33 EACTCC540G
36 EACMGG132G ECAMGG420G
38 EACTAG276V
42 ECAMCT402V
45 EACMTC549G
46 EACMGT329V
50 EAGTCA519G
53 ECGMGG116V
56 SSR_974 EAGMGG217G
57 EACMGT562V
58 ECGMGA79G
59 EACMGA230G
60 EACMTC144G
62 EACTGA118V
71 EACMTC439V
77 ECAMCG492V
80 EACMTC134bV
82 EACTTC333G
90 EACMTC138G
104 ECGMCA168V
114 EAGMCA421V
120 EAGMGG246G
127 ECGMGG580G
133 EAGMCA110V
137 EACMGC349V
146 EACMGA425V
Sc116
SSR_974:291607
SSR_1166:971852
1132736
1177113
Sc169
154096
333272
SSR_818:263616
SSR_10:389984
568005
S 1
0 SSR_974
3 SSR_1166
11 SSR_818
16 SSR_10
LG 19
0 ECGTGT312G
9 ECGTCA305G
15 EACMTC377G
16 EACMTC377V
22 EAGMTA451V
25 ECGMCC73V
29 EACTAG279G
36 EAGMCA192V
37 EAGMCA190V
39 EAGMCA120G
54 EACMCC371G
60 EACMGG168V
63 ECGMGT242G
71 ECAMGG269G
72 ECAMGG268V
75 ECGMTT60V
77 EACMGC142G
81 EACMGC140V
86 EGAMGA126G
90 EGAMGA110G
93 EAGTTA446V
97 ECAMGG481V
103 EACMTC55G
114 EAGTTA464V
125 EAGTGC490V
126 EAGTGC488G
131 ECAMCG224V
133 ECAMCT100V
137 EACTCC400V
138 EACTCC399G
140 SSR_927
146 ECAMCT199G
148 ECAMCT198V
1664097
SSR_806:1588805
1511401
SSR_927:1123880
976642
SSR_1120:730287
Sc69
S 21
0 SSR_806
4 SSR_927,1220

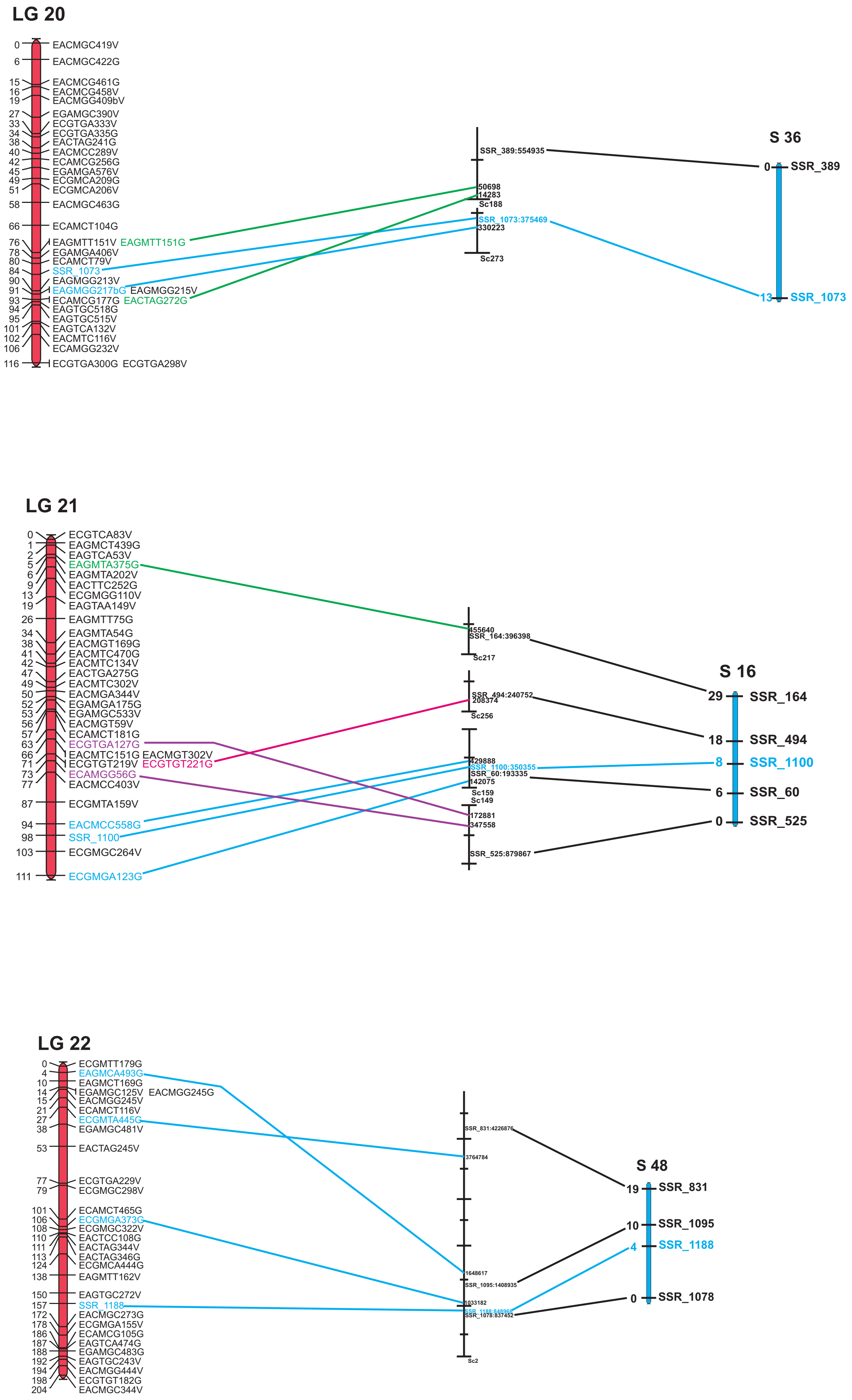

LG 20
0 EACMGC419V
6 EACMGC422G
15 EACMCG461G
16 EACMCG458V
19 EACMGG409bV
27 EGAMGC390V
33 ECGTGA333V
34 ECGTGA335G
38 EACTAG241G
40 EACMCC289V
42 ECAMCG256G
45 EGAMGA576V
49 ECGMCA209G
51 ECGMCA206V
58 EACMGC463G
66 ECAMCT104G
76 EAGMTT151V EAGMTT151G
78 EGAMGA406V
80 ECAMCT79V
84 SSR_1073
90 EAGMGG213V
91 EAGMGG217bG EAGMGG215V
93 ECAMCG177G EACTAG272G
94 EAGTGC518G
95 EAGTGC515V
101 EAGTCA132V
102 EACMTC116V
106 ECAMGG232V
116 ECGTGA300G ECGTGA298V
SSR_389:554935
50698
14283
Sc188
SSR_1073:375469
330223
Sc273
S 36
0 SSR_389
13 SSR_1073
LG 21
0 ECGTCA83V
1 EAGMCT439G
2 EAGTCA53V
5 EAGMTA375G
6 EAGMTA202V
9 EACTTC252G
13 ECGMGG110V
19 EAGTAA149V
26 EAGMTT75G
34 EAGMTA54G
38 EACMGT169G
41 EACMTC470G
42 EACMTC134V
47 EACTGA275G
49 EACMTC302V
50 ECAMGA344V
52 EGAMGA175G
53 EGAMGC533V
56 EACMGT59V
57 ECAMCT181G
63 ECGTGA127G
66 EACMTC151G EACMGT302V
71 ECGTGT219V ECGTGT221G
73 ECAMGG56G
77 EACMCC403V
87 ECGMTA159V
94 EACMCC558G
98 SSR_1100
103 ECGMGC264V
111 ECGMGA123G
455640
SSR_164:396398
Sc217
SSR_494:240752
208374
Sc256
429888
SSR_1100:350355
SSR_60:193335
142075
Sc159
Sc149
172881
347558
SSR_525:879867
S 16
29 SSR_164
18 SSR_494
8 SSR_1100
6 SSR_60
0 SSR_525
LG 22
0 ECGMTT179G
4 EAGMCA493G
10 EAGMCT169G
14 EGAMGC125V EACMGG245G
15 EACMGG245V
21 ECAMCT116V
27 ECGMTA445G
38 EGAMGC481V
53 EACTAG245V
77 ECGTGA229V
79 ECGMGC298V
101 ECAMCT465G
106 ECGMGA373G
108 ECGMGC322V
110 EACTCC108G
111 EACTAG344V
113 EACTAG346G
124 ECGMCA444G
138 EAGMTT162V
150 EAGTGC272V
157 SSR_1188
172 EACMGC273G
178 ECGMGA155V
186 ECAMCG105G
187 EAGTCA474G
188 EGAMGC483G
192 EAGTGC243V
194 EACMGG444V
198 ECGTGT182G
204 EACMGC344V
SSR_831:4226876
3764784
1648617
SSR_1095:1408935
1033182
SSR_1078:837452
Sc2
S 48
19 SSR_831
10 SSR_1095
4 SSR_1188
0 SSR_1078

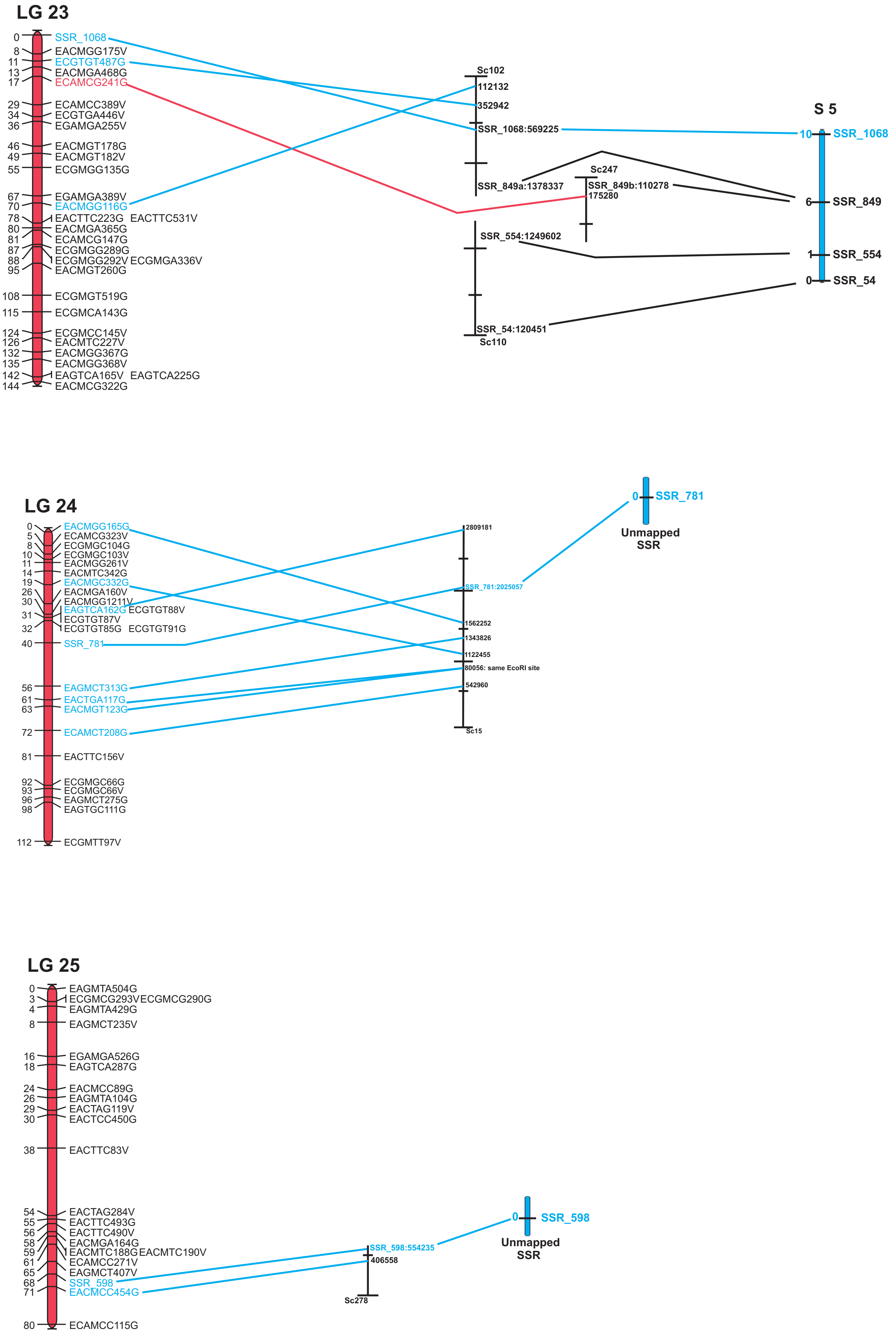
LG 23
0 SSR_1068
8 EACMGG175V
11 ECGTGT487G
13 EACMGA468G
17 ECAMCG241G
29 ECAMCC389V
34 ECGTGA446V
36 EGAMGA255V
46 EACMGT178G
49 EACMGT182V
55 ECGMGG135G
67 EGAMGA389V
70 EACMGG116G
78 EACTTC223G EACTTC531V
80 EACMGA365G
81 ECAMCG147G
87 ECGMGG289G
88 ECGMGG292V ECGMGA336V
95 EACMGT260G
108 ECGMGT519G
115 ECGMCA143G
124 ECGMCC145V
126 EACMTC227V
132 EACMGG367G
135 EACMGG368V
142 EAGTCA165V EAGTCA225G
144 EACMCG322G
Sc102
112132
352942
SSR_1068:569225
SSR_849a:1378337
Sc247
SSR_849b:110278
175280
SSR_554:1249602
SSR_54:120451
Sc110
S 5
10 SSR_1068
6 SSR_849
1 SSR_554
0 SSR_54
LG 24
0 EACMGG165G
5 ECAMCG323V
8 ECGMGC104G
10 ECGMGC103V
11 EACMGG261V
14 EACMTC342G
19 EACMGC332G
26 ECMGA160V
30 EACMGG1211V
31 EAGTCA162G ECGTGT88V
ECGTGT87V
32 ECGTGT85G ECGTGT91G
40 SSR_781
56 EAGMCT313G
61 EACTGA117G
63 EACMGT123G
72 ECAMCT208G
81 EACTTC156V
92 ECGMGC66G
93 ECGMGC66V
96 EAGMCT275G
98 EAGTGC111G
112 ECGMTT97V
2809181
SSR_781:2025057
1562252
1343826
1122455
80056: same EcoRI site
542960
Sc15
0 SSR_781
Unmapped SSR
LG 25
0 EAGMTA504G
3 ECGMCG293V ECGMCG290G
4 EAGMTA429G
8 EAGMCT235V
16 EGAMGA526G
18 EAGTCA287G
24 EACMCC89G
26 EAGMTA104G
29 EACTAG119V
30 EACTCC450G
38 EACTTC83V
54 EACTAG284V
55 EACTTC493G
56 EACTTC490V
58 EACMGA164G
59 EACMTC188G EACMTC190V
61 ECAMCC271V
65 EAGMCT407V
68 SSR_598
71 EACMCC454G
80 ECAMCC115G
SSR_598:554235
406558
Sc278
0 SSR_598
Unmapped SSR

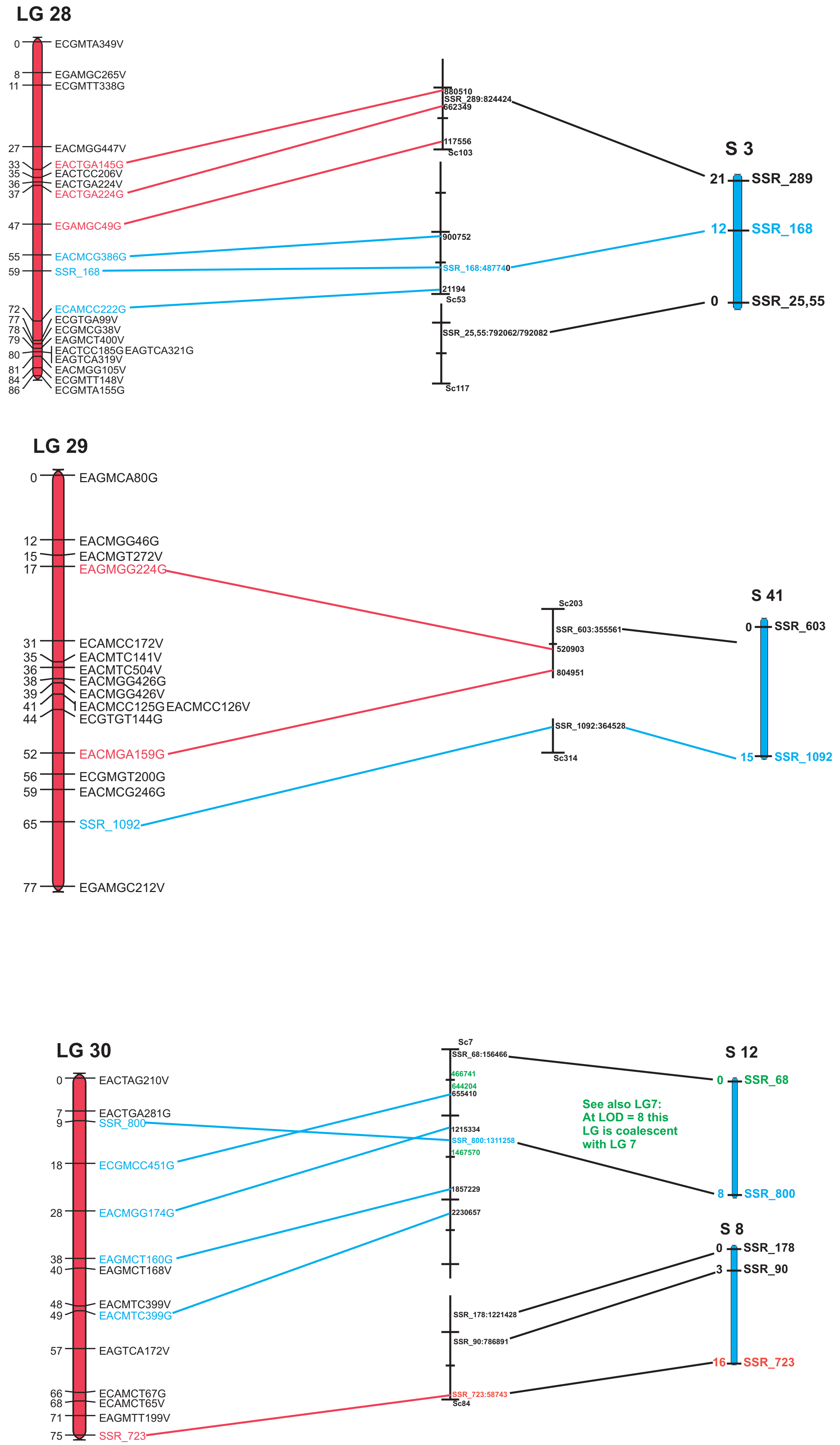
LG 28
0 ECGMTA349V
8 EGAMGC265V
11 ECGMTT338G
27 EACMGG447V
33 EACTGA145G
35 EACTCC206V
36 EACTGA224V
37 EACTGA224G
47 EGAMGC49G
55 EACMCG386G
59 SSR_168
72 ECAMCC222G
77 ECGTGA99V
78 ECGMCG38V
79 EAGMCT400V
80 EACTCC185G EAGTCA321G
EAGTCA319V
81 EACMGG105V
84 ECGMTT148V
86 ECGMTA155G
880510
SSR_289:824424
662349
117556
Sc103
900752
SSR_168:487740
21194
Sc53
SSR_25,55:792062/792082
Sc117
S 3
21 SSR_289
12 SSR_168
0 SSR_25,55
LG 29
0 EAGMCA80G
12 EACMGG46G
15 EACMGT272V
17 EAGMGG224G
31 ECAMCC172V
35 EACMTC141V
36 EACMTC504V
38 EACMGG426G
39 EACMGG426V
41 EACMCC125G EACMCC126V
44 ECGTGT144G
52 EACMGA159G
56 ECGMGT200G
59 EACMCG246G
65 SSR_1092
77 EGAMGC212V
Sc203
SSR_603:355561
520903
804951
SSR_1092:364528
Sc314
S 41
0 SSR_603
15 SSR_1092
LG 30
0 EACTAG210V
7 EACTGA281G
9 SSR_800
18 ECGMCC451G
28 EACMGG174G
38 EAGMCT160G
40 EAGMCT168V
48 EACMTC399V
49 EACMTC399G
57 EAGTCA172V
66 ECAMCT67G
68 ECAMCT65V
71 EAGMTT199V
75 SSR_723
Sc7
SSR_68:156466
466741
644204
655410
1215334
SSR_800:1311258
1467570
1857229
2230657
SSR_178:1221428
SSR_90:786891
SSR_723:58743
Sc84
S 12
0 SSR_68
8 SSR_800
See also LG7:
At LOD = 8 this
LG is coalescent
with LG 7
S 8
0 SSR_178
3 SSR_90
16 SSR_723

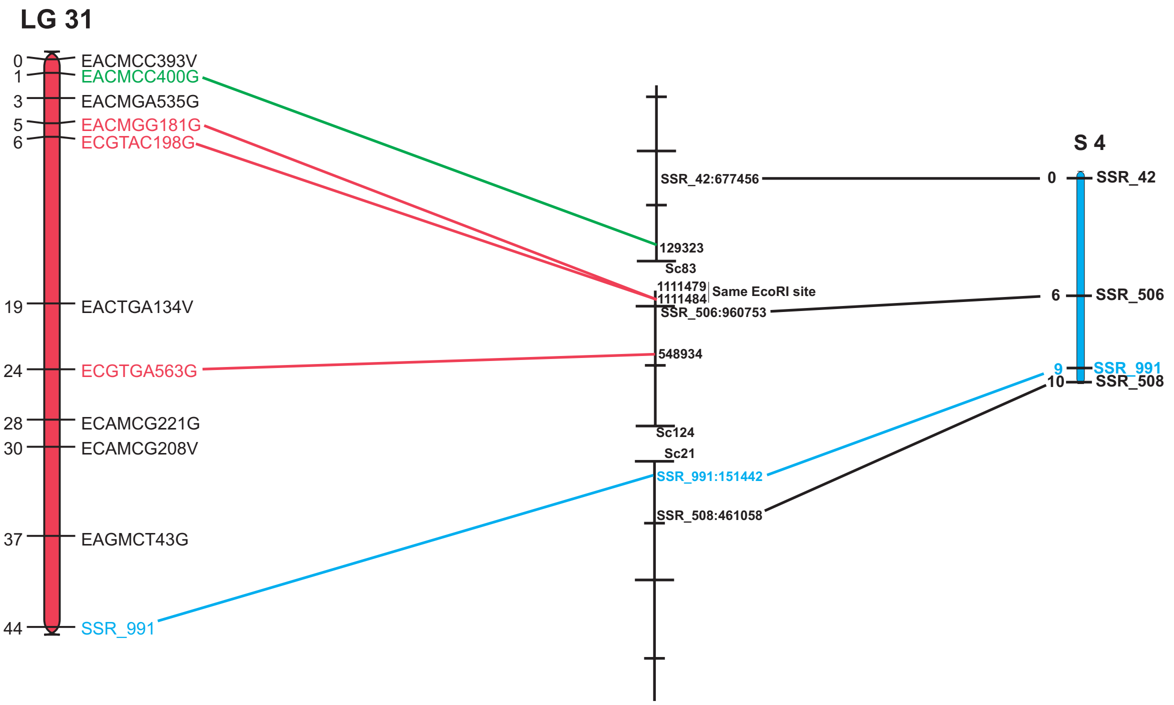
LG 31
0 EACMCC393V
1 EACMCC400G
3 EACMGA535G
5 EACMGG181G
6 ECGTAC198G
19 EACTGA134V
24 ECGTGA563G
28 ECAMCG221G
30 ECAMCG208V
37 EAGMCT43G
44 SSR_991
SSR_42:677456
129323
Sc83
1111479
1111484
Same EcoRI site
SSR_506:960753
548934
Sc124
Sc21
SSR_991:151442
SSR_508:461058
S 4
0 SSR_42
6 SSR_506
9 SSR_991
10 SSR_508

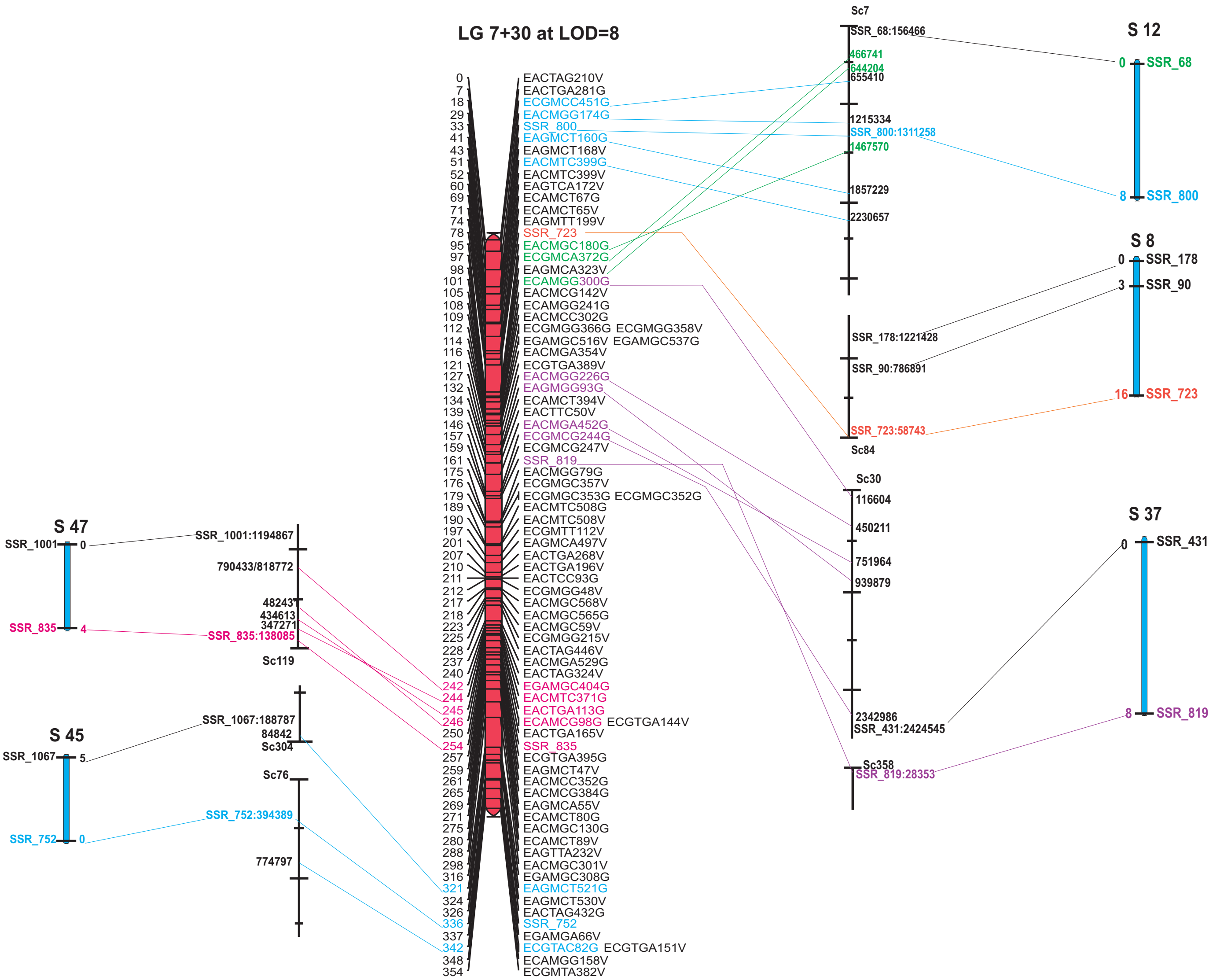
LG 7+30 at LOD=8
0 EACTAG210V
7 EACTGA281G
18 ECGMCC451G
29 EACMGG174G
33 SSR_800
41 EAGMCT160G
43 EAGMCT168V
51 EACMTC399G
52 EACMTC399V
60 EAGTCA172V
69 ECAMCT67G
71 ECAMCT65V
74 EAGMTT199V
78 SSR_723
95 EACMGC180G
97 ECGMCA372G
98 EAGMCA323V
101 ECAMGG300G
105 EACMCG142V
108 ECAMGG241G
109 EACMCC302G
112 ECGMGG366G ECGMGG358V
114 EGAMGC516V EGAMGC537G
116 EACMGA354V
121 ECGTGA389V
127 EACMGG226G
132 EAGMGG93G
134 ECAMCT394V
139 EACTTC50V
146 EACMGA452G
157 ECGMCG244G
159 ECGMCG247V
161 SSR_819
175 EACMGG79G
176 ECGMGC357V
179 ECGMGC353G ECGMGC352G
189 EACMTC508G
190 EACMTC508V
197 ECGMTT112V
201 EAGMCA497V
207 EACTGA268V
210 EACTGA196V
211 EACTCC93G
212 ECGMGG48V
217 EACMGC568V
218 EACMGC565G
223 EACMGC59V
225 ECGMGG215V
228 EACTAG446V
237 EACMGA529G
240 EACTAG324V
242 EGAMGC404G
244 EACMTC371G
245 EACTGA113G
246 ECAMCG98G ECGTGA144V
250 EACTGA165V
254 SSR_835
257 ECGTGA395G
259 EAGMCT47V
261 EACMCC352G
265 EACMCG384G
269 EAGMCA55V
271 ECAMCT80G
275 EACMGC130G
280 ECAMCT89V
288 EAGTTA232V
298 EACMGC301V
316 EGAMGC308G
321 EAGMCT521G
324 EAGMCT530V
326 EACTAG432G
336 SSR_752
337 EGAMGA66V
342 ECGTAC82G ECGTGA151V
348 ECAMGG158V
354 ECGMTA382V
Sc7
SSR_68:156466
466741
644204
655410
1215334
SSR_800:1311258
1467570
1857229
2230657
SSR_178:1221428
SSR_90:786891
SSR_723:58743
Sc84
Sc30
116604
450211
751964
939879
2342986
SSR_431:2424545
Sc358
SSR_819:28353
S 12
0 SSR_68
8 SSR_800
S 8
0 SSR_178
3 SSR_90
16 SSR_723
S 37
0 SSR_431
8 SSR_819
S 47
SSR_1001 0
SSR_835 4
SSR_1001:1194867
790433/818772
48243
434613
347271
SSR_835:138085
Sc119
S 45
SSR_1067 5
SSR_752 0
SSR_1067:188787
84842
Sc304
Sc76
SSR_752:394389
774797

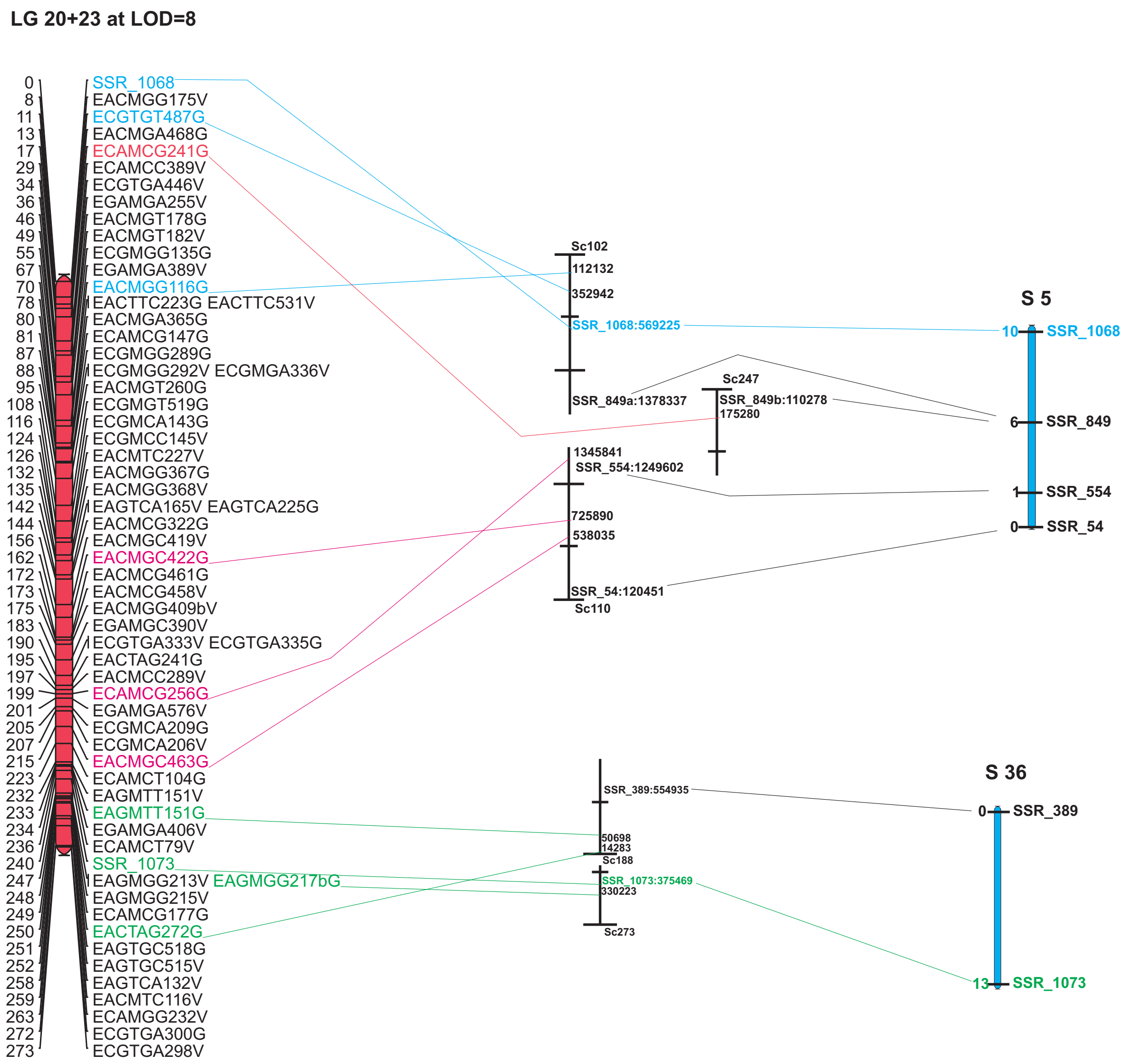

LG 20+23 at LOD=8
0 SSR_1068
8 EACMGG175V
11 ECGTGT487G
13 EACMGA468G
17 ECAMCG241G
29 ECAMCC389V
34 ECGTGA446V
36 EGAMGA255V
46 EACMGT178G
49 EACMGT182V
55 ECGMGG135G
67 EGAMGA389V
70 EACMGG116G
78 EACTTC223G EACTTC531V
80 EACMGA365G
81 ECAMCG147G
87 ECGMGG289G
88 ECGMGG292V ECGMGA336V
95 EACMGT260G
108 ECGMGT519G
116 ECGMCA143G
124 ECGMCC145V
126 EACMTC227V
132 EACMGG367G
135 EACMGG368V
142 EAGTCA165V EAGTCA225G
144 EACMCG322G
156 EACMGC419V
162 EACMGC422G
172 EACMCG461G
173 EACMCG458V
175 EACMGG409bV
183 EGAMGC390V
190 ECGTGA333V ECGTGA335G
195 EACTAG241G
197 EACMCC289V
199 ECAMCG256G
201 EGAMGA576V
205 ECGMCA209G
207 ECGMCA206V
215 EACMGC463G
223 ECAMCT104G
232 EAGMTT151V
233 EAGMTT151G
234 EGAMGA406V
236 ECAMCT79V
240 SSR_1073
247 EAGMGG213V EAGMGG217bG
248 EAGMGG215V
249 ECAMCG177G
250 EACTAG272G
251 EAGTGC518G
252 EAGTGC515V
258 EAGTCA132V
259 EACMTC116V
263 ECAMGG232V
272 ECGTGA300G
273 ECGTGA298V
Sc102
112132
352942
SSR_1068:569225
SSR_849a:1378337
Sc247
SSR_849b:110278
175280
1345841
SSR_554:1249602
725890
538035
SSR_54:120451
Sc110
S 5
10 SSR_1068
6 SSR_849
1 SSR_554
0 SSR_54
SSR_389:554935
50698
14283
Sc188
SSR_1073:375469
330223
Sc273
S 36
0 SSR_389
13 SSR_1073
