# Supplementary material for: A sequence-anchored genetic linkage map for the moss, Physcomitrella patens
Source: Plant J. 2008;56(5):855–66. doi: 10.1111/j.1365-313X.2008.03637.x (PMC2667646; doi:10.1111/j.1365-313X.2008.03637.x)
Supplement: Supplementary file 2 [file tpj0056-0855-SD2.pdf]

**Supplementary Appendix 1: Candidate AFLP Sequences**

The candidate sequences were retrieved from the *Physcomitrella* repeat-unmasked sequence scaffolds ("plus-strand" sequence) using SCALPHunter. Sequence coordinates were confirmed by a BLASTN search of the genome sequence. Sequence co-ordinates correspond to the first base (5'-G) of the EcoRI site in each sequence.

**Linkage Group 1 Scaffold 21**

```
>EACMGG160G_1121559
TTAAGGGCCAAGACCCTTGATCACATCTCAAAGAGAGGTTCTTCTCAAAGGTGTGCAACTATCTTGTAATC
CTAGATTGGCTACAAATAGAGGGCAATAAGTACCAGAAATATTGACATCAATTTATAGTTGTGAATTC
>EACMGG501G_1177923
GAATTCACCCTTTAATTTTACCAAAAAAAAAAACCAACTGTAAATTCACTCTCGATCTTCTGACATCTAAA
AAACCCTAGCAGGCACTCTAAACCCTAATTTATAAGCACCCGAAAAAATATTTTCCTTATATCTCAAAAAT
CCAAACTCCAAATGTCATAAAACTTATGCATAAAGTTTACTAATATGAAATTACACATTCCCTGAAATTTT
GAGCCAAAACCTTATCAAATTGCTCAAGATATAGCTCAAGAGACAACCACTAGTAAATCGGTTTACTTACT
ATCCTTAGATTGCATACGCCGACTTTAAAATCCAATAACTCCTTTATGCCAACTTGAAATCCAATAAGCAG
TATGTAAAAAGTATAGCCACAAAAGTGTTCTCTCTAGTTGCGGTCAGCGATCCTCAATTGCTGTCCGTATG
CATGTGTTATGCTCAAAAGAGTGACAGTAGGTCCAAAACTAACACCTTAA
>ECAMGG498G_1533659
GAATTCCAGCTTGAATATTAGGACCTATTTACATCCTCCGCAGACGTGCCGAATCTAATCACCTGCTCTGA
CTATTACACAAAACCACGTTCAGAAAATGTGCAATACATTCTCTCCTTGTAGTGACAAGAAGCACATACGG
AGATCCTATTTAGGAGAAATTGGCACATGATATCTGAGGGTTGTAACGTAAATAACGGATAGACATCTAAA
CATATTCCTGGAGATGTGCTACGGAGTCGTCTACTTTGGCACAAAATTACCTCATGAAATAAAATAAAATA
AAAAAACAAACTATGATGTGGGTTTCTCTTCTCTTTCTTGTCTTGGAAAAACCTGCTCCTCAGTCTTCTCT
TCACCCCTCGTCGGGGTTGCTTGTTCTTCTACTGTCTCTTTATCATCTGAGTCTGCGACAGGTTCATCAGT
CTCCTGGTTCTTGTTTGATGGCGTAGGAACCTCTTCAGAGGCCTTAA
>EAGMCT70G_401393
TTAACTTCGTGGGGATAATACACACCCGCCTTATTTTTCACCGCCTGAATTC
>ECGMGA238G_435382
TTAAGACAGCACACAATGCAAAAGGGGGGGGGGGTCTTATATCCTCGAGTGATGGACTGCAGGAGTTGCAA
CCATTTGGAACAAAATGATGGTCGGGTTGGTCACGCAAATGGTGTGCTGCCCCCGCCCTCACCCAACCACC
GACTTATTCTTGATGACGGAGAAGCGAGCGAAGGTGATGCGATGCCCATGATTTCATTGTTTGACCGGAAT
TC
```

**Linkage Group 1 Scaffold 29**

```
>EAGMTT101G_511634
TTAATTTCTCTTCTAACCTTGAAAGGTGATTGGTTTAATTATGTTATCTTTTGAATTTGAATTCCATCTTA
TACCTGAATTC
>EACMTC207G_425680
GAATTCACAATAAGTCAAGTAAAAATTAAAAAATAAAGATATAAAAAAACTTATATATATATATATATATA
TATATATATATATATATATCCAATTCTAAATTATATATTCATATACTTCTTATGCCAACAATAAAAATCCT
AAAATTAAAGTCAAAACGGCTATAACTTCCAAAGTTGATTAA
>EACMTC209G_425680_Variation
GAATTCACAATAAGTCAAGTAAAAATTAAAAAATAAAGATATAAAAAAACTTATATATATATATATATATA
TATATATATATATATATATCCAATTCTAAATTATATATTCATATACTTCTTATGCCAACAATAAAAATCCT
AAAATTAAAGTCAAAACGGCTATAACTTCCAAAGTTGATTAA
```

**Linkage Group 1 Sc38**

```
>EACMTC216G_335078
GAATTCACAAATATTTTCTAAAATAAATATACTTATAGTTATCTTTACTTCTAAGATTAAGGTTTAAAATA
AACAATAAAGTTATTTCATAATTTAATAAAAGATAAGTTTTGTCATATGTTATTTGTAAGAAATTTCTAAT
CCATGTTGTTGTGAGTCCTACTAATAAATCTTAATTAGAAGATTGATTAA
>EGAMGC266G_56643
```

TTAAGCATAATAACTTTGTCTGAAGTATCTGTCTAACACTGTATACAATGTGAACCGAAACATGCACCTCC
TGTATCATGACTTACTTGGGGTTTTGAAAGAGAGGCCCCTGAAGGAGCGGTAGAATCCACGCTAACTTTGT
CTGCAAGATCTGTTGCACCTGCATTTTTCTGGGCCCGTATTATAGGCAGCTTTCGGAGCCTAGAAGTTGAG
GACAATGTCACTCTCTGCAGCCCAACGTCGAATTC
>EAGMTT114G_2100454
TTAATTTGGAGAAAGTAATCAAAAAAGAATAGTATAGAATAAGTTTTTATCTTTAGAATAAGGATTACAGT
TAGCCATTATATATGCTGAATTC
>EAGTTA233G_1942567
TCGATAAGGATCCAACAGAGAAACATGAAAGACTGGATGTACCTTCATAGAAGCCGGTAACTCCAGCCGGT
ATGCCACTGGATTAATTTGCTTCTGGATGATAAATGGTCCCAATCTTTGATAGTCCAACTTATCACATGGT
CGCTTGGTCTTGATATTTCGTTGAAGAAGCCATGCTTTGTCTCCAACTTTGAAAGTAGGTGCTGAATTC
>EAGMCT229G_1313412
GAATTCAGCGGAGTCTTCAGAATTGTTTTAGCTCTGTGCACTTTTCCCTTATTGTTATATGAGATTCTCTA
TTTGGAAATGTCTTATTCTGGGTCATTCTCTGAATACTTGAAGTCGACAGGGTTCTTTGTAGACGATGTGA
ATCCTCTCTCTGGTATGTGGTGCAGAGTTATGAGAGAAGCTGATGCTAACAAACTGAAGTTAA

**Linkage Group1: Scaffold 59**
>ECAMGG446G_403858
TTAAGGGACGCCGTGAAGATCATTCGAGTGGTGTGGGTCATAAGGGTTTGCAGTTTTCGGTGGCGTCCATG
GCAGGCAGCTGCTCGGATGCGCCGTCGTGCATATTTGTTGGGCCAATTGAGACTGCGGAGAAAACACGCTT
GGAAGCTTTGTACCAGCAAGCGCGAGATTCGTATTACAGTGGGCAGCCGTTAGTTGTGGACGACATGTTTG
ACAAGGTGGAGTTGCAGCTGCGATGGCATGGGTCGAAGTTGGTGTTGAAGTATCCCCGGTGTAGCTTGAGG
CGTTTCACGGCCTATGCGGACGCTGAAGTGGATCCGTCCCAGATGCGAGCCTTGGCTACGGTGTGGAGCTT
GCTTCTCGCAGTGGGATTGGGGGTTGCTGTGGGCCTGCCAGTTTTAATTGCAACCTACATGGAATTC
>EACTTC272G_1580617
GAATTCACGTCGCTGATGTTTCAGAGCTGCGCGGCGCTGCGGCCGTACATAGCTCACATTGACAACATATA
TAAGGATTTCACGACGTACAAGACGAGTGTGCCCGTGGCGGGCGCGATCATGCTGGACGAAACCCTGGAGA
GATGTTTGCTTGTGAAGGGTTGGAAGGCCGGTGCAAGTTGGGGCTTTCCGCGAGGGAAGAAGAACAAGGAC
GAGGAGGATTCTATCTGTGCTGTGCGGGAAGTGATCGA
>ECAMCT406G_1072739
TTAACTCCACCATCTGCTGGGGCTTGCAAGTCTAACACGTTTATTTGATCACCGCAAAATCATCAATCAAG
GAGGCTTGCAAGTATATGTATATATATATATGTATATCCTAAGAAGCTGGTGCACAGAAGCATTTGAGACA
TCTAAAACGAACCCCAAATTATCGTCAATCTTCATGCGAAATGCTGATCCAGTGTTTGCAATTGTGGGAAC
AGGTGTCCATAATGAAGCGGTTGGATCATCCCAACATTGTGAAACTTTATGAGGTCATTGATGATCCGCAG
AGCGATAAAATGTATCTGGGTACCTGATGATCACCTTGGTAAAAGTAGCAGTGATTTCATATGACCTATTG
CTGCAGAATGTCCTACTTTTGGAATTC
>EACTTC451bG_778107
GAATTCACAAAAAAACATTATTCATAAAATATTGACAAGCTGTTGATATGTTTGTGAATCCAAAAGGCATA
AGATTATATTCAAAATATCCATACTTAATTTAAAAAACAATCTTTAATTCATTTCTTTTTTAAATGAGAAT
CAAGTTATATATTCTTCATAGATCTATTTTTATATAGATTTTTGTTTGATTGAATTGGCCAAGAAGTATTA
AAAATAAAGATAGGAAATACTTATTATTGATGGTGATCTTGTTAAATCTTTGATAATCTATACACATCAAC
AAAGATCCATTATTTTTCTTTAAAAATAAGATATGAGCTATTGTTGGAAGTTTTCAATAGTATATGAAGTT
TTTAGCCAAGTTCTCATTAATATATTTATTTGATACAATCAATTCATTTTACAATAAGTTGAAGATTGATC
GA
>EACMCG63G_313976
TTAACGAGGAAGAAACTAACTCTGATATCTGAGGTGAGTGAATTC
>EACMTC207G_416474
GAATTCACCATTAGTCTGTATAATATGTTACCATATTACATGTTATTATGGATAACAGTAAGATATGGCAT
TCAAAGAAGCAAAGTATTCATATGTTACTCATGTCAGAGTTGTTGAATAAAGAGATTCATTACTTATCCAA
TCTATTGTCTATTTCAATTCAAACTTCATTTTTAAACCATGATTAA
>EACMTC209G_416474_variation
GAATTCACCATTAGTCTGTATAATATGTTACCATATTACATGTTATTATGGATAACAGTAAGATATGGCAT
TCAAAGAAGCAAAGTATTCATATGTTACTCATGTCAGAGTTGTTGAATAAAGAGATTCATTACTTATCCAA
TCTATTGTCTATTTCAATTCAAACTTCATTTTTAAACCATGATTAA

**Linkage Group 1 Scaffold 98**
>ECAMCT406G_45993

GAATTCCAATGCCATCTATTTTTGTAAAATTTATTTGGACTAGAATTTGGTAGACATTGACAATTGTGCTT
CAATGACAGTCAAGAATTTTTAGTCTTCCCAAATTGTATTCATTGTATATCCTTCAATTCACAAAAATCAA
CAGTCTAATTCTGCTTGTTGTTTAGCTGCCTGAGTAGTCCAGTAAGTGTGAGCCCGTGTCACCCTTTCTAA
TAACTCTTGTGGAACTGGCTCTCCTCTCCTTTGGTGAGGGGCAATACGTGCCGTATGGACCAAAGTTTTCC
ATTTATCCTTTTCAATGGACCAAATTTTTTAAAAAATAAATAAATAAAAATAAATAATCTTTCATGTATAA
CTATTTTGATGTGGAAACTATCAATAAGTTAA
>EAGTGC265G_1282606
GAATTCAGGAATCAACCCATAAGAGATCAAATCACTGCTCTCTACCTGCACGAATTAGTCAAAATTCACTT
GTCAAGTAATCTGTAACTTCAAAACAGGCATGTGCTCAATTTCCTACTGAAAAGGTTCAACAAAGCAAATC
TACATACCTTCATCAGACCTAGAACATGCCTAAACCATGGAAAGAAACCTGAAATTGAAGGATGTTGACAA
GAAATTTCTGCAGGCATGAAAAAATGCTCGA

**Linkage Group 1 Scaffold 173**
>EACTAG211G_427443
TCGAAGCAACGAAGCCTGGGTGCTCTTTACGACTGGGCTTGTTGTAGAGAAGAGGGCGTGAGATTTTGGAC
GAGTATTTTGGATTGAGCATGTGGGTCTTGTTGCAGAGGTTGTATATCTCTTCGCAATCCTGTTCTCAGTA
TCCATCCGGGGGTTGATGGAATGTTGCACACTTTGGAATTGGTGAATTC
>ECGMTA387G_571101
TTAATACTCACAGTATCTGAATTTTCCTCAGAGCTAGCTCGAATTAGTCGGTACCGCCGGTTGTCCAAACC
CACAAGTCTGTTCGCATTCCCCGAGAGAACCACACTGTGAAGCGGGGATGTTCTCCTACCCACATTGCCCG
CAGCAAATCCATCTCTCTGGACAACCGAGCTGGTTGTCGCAGGCACGAGCGGCGGTGAAAGCATAACCACT
CCGCAAGGCGCCATTCCTGAACTGTGAAGCCTAATTAATCGGCGCGACCCAAGTGCAGTTCCAGCACTAAT
TTCCCCAATCTGCAAATACCAGACAACACAGCCAGACTTCGTGCTCTATGCACCCAACATTCGAGTCGCAA
CAACGGAATTC

**Linkage group 1 Scaffold 230**
>EACMTC207G_96196
GAATTCACTCTTGCCTCTATATATACTATCTACCTAGGAACTGCATGGCAACATCATTTAGGACCTCCAAA
ATATATTGACTTTGTACAACATGTATCTTAATTTATAAAGCAAGTTTTTTATATGAGTACTATATATTTAG
CTTATTTCATAAGCTATCAATACATGTAATATGTGATTAA

**Linkage Group 2 Scaffold 1**
>ECAMCT442G_1016882
TTAACTCAAAAGCCATCATTTGATGACTTATCCGATAAGACATTGTAATTATCACAACCTTTAAGATGACA
TAATAGGCATGAAGACCTACAAGAATAACATGTTTTTTTATAAAATAGATGGTGAAATACAACAAGTTTGG
TATCACGACTTGGAAAAAGATTCTTGATGTTTGGTGATTTTATTGGAGGTAGGCATGACCTTGATAGTGTT
ACAAATGTTTTATTTGAAGGAAATTATGAAGGTTTTAACAATGATCAAATCACAAGGTGGTCTCTTTGTCT
TCGAATTGGACATTACTAAGAGTTGAGAGGACCTATCAACTATCTTTAAAGAGATCAAAGATCAAAGTACG
TGGCAAATACAATTTGTTGCATCTTAGACTTTCTCAACAAATGATCACAATTCCTTGTGGAATTC
>EACMGA294G_1455787
TTAAGATATGTCCCCTACAGACTTTACTCCTCCCTAAAGAGTAATACAAAAACATAACCTCCAAATCCTGA
TCATCACCAGGGCTCAGTGCAGGCGGATGCAAGCTGGAAAGCCCTCTGTGTATGTGTGTGTTTCTTTTCTT
CTTTTTTCTTTTTTGGAGAAAATGTCGATAATTCAGCTGGGATATGTTGGTGAAAGAACTTACACACTGGA
AGACTTCCTTCTATGTTTTTTACTAGAGAAGTTGTAGAAAAGTCCCACTGGCGTGAATTC
>EAGMGG515G_168865
GAATTCAGATTGATTGGAGTGAAGTTTGTTATTCCTAGTGTTGGATGGAATTTATTGAGTTTTGAGCAGTC
TTTTGAGAGTGCAGGGTTGCACATTGTGGTTATCAGTTGATGAAGGAGTATTCTTATTGGAAGGAAGCATT
GTAGTATTTCTTAATATTTTAGTAGTTCAGAATACAGTTAATTGTGTGACCCTAACAAGTGAAACTCATGC
GCTTAGTCTTTCCTTGTGTTTGAATTTCCTTATACAAGTTTATTCTGGAAGGAATTATAGAATAAGTAAAA
TGGCAATCATAATATCAATAGAGGTTTCAATGTCCAATTGAAGCTTATAAGAAGGACCTTTATTTTTAGGT
TTGTTTGAACCTTCCTTTTTTTTTGTCTTTTGTTCTATTTTCTTAAATTCTAACTCCACTTTTTAGTTGACA
GAAAAGCATTGCTTGAGCTTTTGAGAAAGAAAGCTCTTATCTTCTTTGAGTAGACTCTTTAATCCTTAA
>ECGMGT164G_2281676
TTAAGTTCTCTAAATATAAAGAGTTGTCTGACATCTAAGCAAAAACGTTTGCTAGATTCTTTGATAATTGC
TTACGAAAAGAATTGGCCAACAAAATACTAACTCTGACTGTGCTCACCGTTGCCAAGATCTGTTCGGAATT
C
>ECGMTA470G_3194656

GAATTCCGGGTACTCTTGGTCGGTGGGAACATACTCCTGGTTGTCGAAATCTACCGGCATTGACCTCGCGT
GGGGAGACTTCCACTGCGATGCAAGCACTATAAGCACCGTCAGCAAACTCGCCACCCGCCCGGACACCATT
CTTGTGGTCGATTTTGAAGAAGGCTCGTCTTGGATCCTTTGCTGGCACTACTGGGAACCTGAAGTCTAAAC
AAGCACAACTTCTCTGTAAGTTGAAAGTCTTTTGAAATGCCTTTGCGCAGCAACCAACAATCCCAATAGGA
TCCGCAACTCATAATTCACAACGCAACGAAGAGTTGGAAGAATCACCCCATCACATTCTGGTGAGGAGAGA
TAGCGAAGTATGCATCATGTCCGTGGACATAAGCAGGATTAGAGCCTACTCACGTGGTGAACAAGCCGATG
GAAGTAGCACACTTCCTATTAA
>ECAMGG295G_4935908
GAATTCCAAGATGTTTCTGAGAAGAAGAATGCAGATATTCTTCCAGACATCGACCTTATGATTGTTCAATT
GATCTTCAAGAAGGAGCTCAACGGCCATTTAGACCAATCTATAACTTATCACAAAATAAATTGGTTGCACT
AAAAGAGTATATTGACAAGAACTTGGCTAAAAACTTCATATGCCATTCAAAATCTCCAACAGGAGCGTCGA
CCTTATTTGTGAAGAAAAAAGATGGATCTCTATAGATGTGCGTAGACTATTGGGGCCTTAA
>ECGMGT64G_3394646
GAATTCCGATCTGAGAACCATACAAGTGGACCTCAACGTAACTTAA
>EACMCC218G_5106185
GAATTCACATCTGTTCTCCCTTTTCGAGGGAAAAAAAAAAAAAACAAGACAATCCGACGTGGACTATGAATT
GAAGATCTCTTTCACCACGTGGCACTATCGCATAGGTCACGATCCTCTTTTCTTTTTCATGGTTCATTTTG
TAGAGAGGAGAATGACCGGGAGTAGCAGGTGAGGGTGGTGTCTTAGGGTTAA
>ECGMGA178G_4965329
GAATTCCGATCATCTGTCCGCGTACAACCACAATGGAATGCCACTAAACTTCGACTGCCAAATCCGTCACG
ATGTAAACGTTTCCCGACATCAACCAACCAGTTAACAGATGCACTACAAGTGAACACAAAAGACGTCAACA
GATCGTCTTAA
>ECAMCC235G_4804576
TTAACCTATTGTTATCTATTTTTATTTAATCATATAGATAATTATTGGTTTACATGTATTTGTCCATGATA
TCTTCGTATTTAATTCTAAGATTTTTTTTAAGCTTAAATTTCATGCCATAATTTAAAATGAATTAAAAATA
TAAAGTTTAATTGTTAAATTAATGATGATTTCTAGAGAATAGAGGTGAACTAATTATTTTAGTGGAATTC
>ECGMTA164G_4630983
GAATTCCGCATCTCTTAATTTTTGTAACACACGACAAATATGAAGTTCATGTTTATCTATGTTCTTAGAAT
AGATCAAGATGTCGTCCAAAATACAGACAATAAAATCACCTAAAAACTCTCAAAAAATATCATTTATTAA
>EACTGA105G_4934846
TCGAGAAAGGGTAGCAAAGAACAAGCTTGTATCACTATGAGAAAGATCACGTTCAGCCACCACGTATGCTG
GTGAATTC

**Linkage Group 2 Scaffold 47**
>ECGTGA249G_1790849
TCGAGATGCTTCCGTTGCAGTTGAACTCAGCCTGCCGTTCAAAGTTCTCTCCAGCATTACATACCGCTTTC
TACATTTACCACCTACAACTTTCGCATTAGATTTTCCCATTCCTCTTGAAGGGCCATTCATGAATTTTGAA
CAGTTCCTTATTTCTATGATTTGAAAATGGGTTTGAATTGCGAGGTCTGCAAGACAGCATAATAAATAACG
ATTCTACGGAATTC
>ECGMGG72G_1598173
TTAAGGAGACAGCATCTTGATGCTTTCATTCGAAATATATAACAATCGGAATTC

**Linkage Group 3 Scaffold 5**
>EACTAG243G_1115068
TCGAAGTCCCTCCCATTTCATAAAACCCCAGTAGAGGCAGTATTGGTGTGAGAGTGTCCCTTCTTTAAAAC
CATCATTTTGATGATGACTATGTAGTAATTGACCTAATTTTTTTTTTTGGGTTTAACTAGAAATTCAGGAA
GAAAAAAAAAATCAAAAATCAAATTATGTGCATAAAAAAATAAATATAGATGGTGTTAGTGATGCTCTTTT
GTGAATTC
>EACTAG151G_1930828
TCGAAGAAATTTGTTGCAGCTAAATGGCCCTTTGTTTCACAGGAAAGTTGCGCTAGAACCTCACTGGTTTT
GCACTGGAATTGTGAGTGGTGGCTTCAAGGCTGGCAGGTCGGATCAACCCTCGTGAATTC
>EACTCC294G_1040063
TCGACCCGTCTGGGCCCCGCGGCTGCTTCTTCGGCGCCTTCCCCAGTCGGTGCAGCTTTTTCATTCATCCA
TCTATCCATTCACGGGCTTCGTTCGTCTCATCCCTCATCTGTTCCATTTTCCGTCCATCAACTTCACGGCC
TCGTTCATTTGTTCCCTCCTTCATTCATCCATTGGAGATCAACCTCGTGCTGGTTTATTAGTGTCGGGTTT
CGCTGAATTAGTAAGGATTTAATTAGTATATTATTGCGGGTTAGATCATGGGGTGAATTC

>ECGTGA430G_1204426
GAATTCCGATCTGAGAACCAACTTCAACGTTACTATAATTCAATGGCAAATCCAACACCTCTTCAACACAT
ACATTCGGAAGTAGAGGATATGCAAATTGATGCAGTATGTTTCAAACCTCTTACTGAGCAAGAAAAGACTC
GTCGTCGTCAAGAAGGATTATATTTATATTGTGGAGAACCAAAACACATTGCTCAACATTGTCTAAAGAAA
CGACGTAATCATAAAATGAGGAGTATGGCTGTGAAAGAAGACAATATGTTAGAAAATGAGTTTGTCCAGCT
ACAATAGGGACCGTGCGGCTAGAACCTTGCACAACATCTAGTAAAGGTTCTATTCTCTCCTTAGAGCCCTC
TCCTTGTTTTACATTGAATATTATTTTAGGACCTAAATATTACAAAGTCTCGA

**Linkage Group 3 Scaffold 107**
>EACMGA257G_1181725
GAATTCACCTTTAATTTTTATCACAAAAAAAACCAAGTGCAAATTCTTTTCCAATCTTGTGATATCTAAGA
AATTCTAGCAGATGCTCTAAATCCTAATGTACAAGAACTTGACCACTATTTTCTTGATATCTCAAGAATCC
AATATTCAAATATCATGAAACTTATGTAAAAAGTTTACTAACATGAAATTACACATTTTTTTAAATTTTGA
GCCAAAACCTTATCAAATTTCTTAA
>EGAMGC205G_1245704
TTAAGCCTAATGCCTTAGTCAACATGTAGTGTCTATCCTTCACTAATGCCTTTGTCAACAAATCAGCAAGT
ATGTCCTCCATAGCACAATACTTCATATCAATCACTCCCATTTCAATCTTCTCTCTTATAAAGTGATATTG
AATGTCAATGTACTTGGTTCTTGAATGATGCTTCGAATTC
>EAGMGG125G_1059547
GAATTCAGCAATCTCAAATCTTTGACTATCTTTGATATATCAGATTGCAATAGTTTTGCATCATTGCCAAA
TGAATTAAACAACCTCACATCTTTGACTACCTTAA
>ECGMGT93G_311572
TTAAGTTCAGTCACCCCGTTATAGTCAGCCGGGGCATACTGAAATGCTCCATTCGATTCCAATAACGGAAT
TC
>ECGMTA273G_430795
TTAATATTCCCACGCAATCTTTGCACAGTCGACATGCGAATGTTATTTTATGAGTGAGCGCTGAAAAAAAT
TGTGTTCAAATCATTGAACATTCCAAGACCTGGTGGTCTGTTATCATTTCGAAGCAGCGCTTCATCGTTCA
TCATGACGAATTGAAACTTAGAATGCACTGAGATTGTAAGCGGTTTGACTCTCACCGCAGCTATGATTTTG
GACTACGCATTGTGCAATTTGAAATACTGACGGAATTC
>ECGMTA580G_471723
GAATTCCGATGCATAGACTGATGTAGATCGCATTCCTTATCGTAGTGATCGGATCTTTGTTGTAGTCCATC
AAACCTTGTCGTATTCATCAAATGCTTCTGCAAACGCCTCCAAAAAGGTTTCAAAGTTGTTCAATACGGAC
GATCATTTCTCAAACAAAGGAGCAAACCATGATAGAGCTTGTCCTGTAAGTAAGGTCCCAACAAGCCCAAC
ACAAGACTCCTCCGTTGGATAGCATTCGGGCTGCAAAACTGTTATCAACCTGATTTGGTTGACGAATCCTC
GAAACTTGGAATGCGTACCATCGAACTTGTCCGGAAGGCTAACTTGGAGCTCCTTATGTTGTGGAACCAGC
GGTGCTTGTCGAACCTGAGGCAAAGTTGCTTCTCCAAGTGTAGAAGTTTGAGGCTGGTGCAACCTAGATTG
TGCCATCCCAGATGCTCCATCATTATGAAGCCTTGCCACGACTCCTTCAATCTTTGCAGTTCTTTGCGATA
ACGTAGCGAGGGCATTCATGATAGCATCCAATTTGGACTTCGAACTTTTCGTCATATTAA
>EAGMCA260G_528644
GAATTCAGGTACCAGTTTATTCCACAGCTGTTGTTTCTCAATGCTCTCTTCGGCTATCTATCATTTCTAAT
AGTTCTGAAGTGGTGCCAAGGAAGCAAGCCCGATCTGTACCATGTAATGATTTACATGTTTCTAAGTCCTA
CTGAAGACCTTGGTGAGAATCAGCTCTTCATGGGTCAAACTTTTGTTCAGGTACGAAAATCTTACCTCTTT
CTGGGGTAAGAAATGTTCTATGTTAA
>EAGMGG485G_1059403
GAATTCAGAAACTTTACATCTTTAACTATCTTAAATATGGGAGGGTGCAAAAACTCGATACCAATGCCAAA
AGAATTCAGAGACCTCACATCTTTAACTATCTTAAATATGGAAGGGTACAAAAATTTGATATTAATGCCAA
AGGAATTCAGCAATCTCAAATCTTTGACTATCTTTGATATATCAGATTGCAATAGTTTTGCATCATTGCCA
AATGAATTAAACAACCTCACATCTTTGACTACCTTAAATATAAGAGGGTGCAAAAACTTGATATTATTGGC
AAATGAACTGGGCAACCTCACATCTTTGACTACCTTAAATATAAATGGATGCTCCATATTGATATCATTAC
CAAATGATTTGGGCAACCTCATATCTTTGACTACTTTATATACAAATGGATGCTCTTGATTGACATCATTG
CCAAATAAATTGGACAACTTCACATCTTTGACTACCTTAA

**Linkage Group 3 Scaffold 133**
>EAGMCA234G_1106806
GAATTCAGCACCATATGTTGCCACCCCAAGTGCACCATTCAAACCCTAAAACCTCCAACCCTAAAAAACCC
CTAAATAAACCCTAATGAACCTAAAGGGGGTTCGCAGGGGAGCCCAGCATTCTTCCTTCCCCCACGCCGCT
TGCTGTGCGATGCTGAGCTTTCGATTGCGATTCTACGGCGACGCCAAGGGCCAACTTACGAAGGTGTTAA

>ECAMCT353G_71605
GAATTCCAAAGAAAGATAATCTCAATACACTCTTGTGTCACTCAAAAATACTTAGAAATCACTCAAAGGTG
ATAATATATTGAACTAGAATGGCTATTGCCAAAGATTAGAAAACATGATGACTCTTCAAACTAGATTGAAA
ATAAGAAAGGAGTTGACAAGAATGAAGAAGGCTTCACTCTTGTCTCTATATATGCTATCTAGTTAGGAGCT
ACGTGGCGACGTTCTATAGGACCTCCAATATATGTTGAATTTGTAAGACACTTGCCTTGAATTACAAGGTA
AGTTTTTCTACATATGGACCACATAATTAGGTTGATCTTCAAGTTAA

**Linkage Group 3 Scaffold 158**
>EACMGT70G_111815
TTAAGTTTATTCATTTGAGATAACCTTGGAGTTCAATATCTCGTGAATTC
>EACMGA107G_251090
TTAAGAGTGGTTCTTAAATTTTCACAAACATTAATGATATGGATGCCATTGTGGCCTTTGCAAAAGTTGGA
ACAAGATATAGTGAATTC
>EAGMGG228G_830246
GAATTCAGATAAGTAGAGGCGAAAGAGGGATGTGTTGACACCTGTGTCACTAGACTATGAGTTGGATCGCC
CAAAGTATCGCGTTGAGCAGAAGCCGCGATATGCATCCGAAGTTTGTGACACTCCAAAGTGAGGTGAGTTA
GCATGGAATGGAAGTATTCATTTTCTTCAGCAAGGCGTTTGATCTCCAGGCGTGCTTCCCTTAA
>EACMTC227G_872320
TTAATCTTCATGGATGAATTATTAGTTGAATGACAATATTTAGATGGATGTATATATCAATACCTTCCTTT
CAAACTTTATTTGGTGTAAGTATATAATCAATGTTTTGCAAATTATTTATCATTTCATTTGCTTGTATTAT
CTAACTCAATAGTTTTGGTAGATGTTGGATTTGTTTTATGAATAGATAGTGTAGTATTAGTGAATTC

**Linkage Group 3 Scaffold 182**
>ECAMCG264G_543197
TTAACGTTAGGGTCACACAATCAATTACCTCCTGAATGTATCGTCTTTATTTCTAATATCTGAACACACAA
GGAAACTATAATCTCTTCCTTCACCTTATTGGTCTCTTCCTTATAACAATTCAGTCATACAAATAACTGCT
ATTTCCACCAATATATTCACCCTCATAATTTCCAACTGTAATACTGTGCCTTCACAGCTTTCAGCACACAA
TTACCCTTGTTCCAATCATTGGAATTC
>EGAMGA80G_193061
TTAAGACGACATTTACTATGGCATTTATTATGGCTCATTATGGACCTTTCGCCATCGAATTC
>ECGMGG556G_811208
TTAAGGAGAAGCGTTCAAAGGTTGAAAGGGAAAAAGAAAAAGAAAAAGAGAAGGAAAGAGAGAAAGAGCGA
GAGAAGGAACGTGAAAGAGAGAGGGAGAGGGAAAGGGAAAGGGAGCGACAAGAACGCGAGAAGGAGAGGGA
GCGAGAAAGGGAGCGGGTAGAACGGGAAAGAGACCGCGAGAGGGAGAAGGAACGAGAGAAAGAGCGGGAAC
GTGACAGGTCAGAGCGTGAGAAGGAACGGGATGAGAAAACTAAAATCAAAGTTGAAGAGCCTCGAGGAGCG
CCTGTTGGAGGTGCGCACATATTTATCCATCTGCAAACTCAGTAGTCGCCAATGACTAAAAGGATCTTGTT
GTAGGGGAAATTGTGGTATGTTTCAGAAGAGTAATAATTCTGTTCACGATGGAACTTAGGGTGCTCATGTG
ATGTTGGAAGTGGCTTTAGCTGATAGTTACTAAGAATATGAATAGAAAGGTTCGGGTTCAACCTCAATGGA
AGACCTAGGGTTTAGTCTAGAAAGTCGGAATTC

**Linkage Group 3 Scaffold 305**
>ECGTCA160G_497731
GAATTCCGGTTCTTCGCGTGAATCGCGACTGAGGTGTGATCGTGTCTAGCTGTTAATAACTCACGCTCTTA
TTGACAGCCACTGGTGACCCCTGCATGCTTCTTGGAGTTTTGTCACAACAGTTATGAGAATGTCGA
>EAGTCA252G_498584
GAATTCAGGTGGCGGAGGAGGCTGCGGATTCCTTCGCATGAGAACCCATATTATCGCAGAGTGCTCAAAGA
GAGAGAAAGTGCGCCGTGCACCTCGCCACCAATGGGAGGCAGGGAGATACTATGAATCTGGGCGAGAGGAA
CAGGTAACATCCAAAGATTACCCCAACGGTTTGTTCAGGGAAAAGATAAGATCTCCGTGACAAGCATGCCC
CGACCTCAAACATGTCGA
>ECAMCT127G_260833
GAATTCCAAAGATTGAAACAAGGATAATTATGTGCTGAAAGCTGTGAGGGCACAGTATTACAGTTGGAGAT
TATGAGGGTAAATATATTGGTAGAAATAGCAGTTAA

**Linkage Group 3 Scaffold 421**
>EACMTC178G_129991

TTAATCTAGTTCTTGCTAGTTCTTCTTGAACTTCTTGTTAGATCTTGAGCTTGTATTTCTGTGTCCGTAAC
ACAAAGGCTACTTTCAAAAAATAGTATGTGCAAAGTTATATTTGGTGTGATGATGATTCTTATTCTCGAAA
AGATTGTGGTGAATTC

**Linkage Group 4 Scaffold 42**
>ECGTGA59G_820322
GAATTCCGTAAGGTCAATGATGAGGAGTTTTTGATCTCGA
>EACMCG361G_724948
TTAACGGGTTTCCTACAGCAGCAGCAAATCCGAAGCTAAAGTGCTGATCGCTGAATTACATTTCTGCAATC
CCTGGTGAGTATGATTTGGGCTGTAGCGAAGACATTGCTACTAGCACCCTTCAAAATACAAAAATCACATG
ATAAGTAGGTAATAGATGATGCTCCTTGCATACACCTTATATCTTTGAAGACTGTTGAAGGAACCCCGAAT
GGATTCAAGTCCATGAATGCTAATGGCGACAAGGCATGAGGTCCGGAAAACTGCGAGCCACTAGTGAAAGA
AGCTCTGAAATTTATGCCATGGCTTCCGAAGATGGCAAGGGAAGCAGTGAATTC
>EAGMTA346G_147600
GAATTCAGCGGCATACATGGTGGCTGAATGTGATCCTCGTTATAGTGATACAAGCTTAGTCTCTGCTATCC
TTTTTCAATCACTTTCTCCAAATATAGTCATAAATACTTCCAAGAATGAGTTCAAATCATAGAATAGTCAT
GAATCTTTTTCCATCAAAGGTGCAAACCAAGAGAGAGCACTTCCAGATAGAAGTGTACCTAAGAATCCAAC
TTGAGTAAATCCATAAGGGTATTGGAAAAGATGCAACCTTAAGTAGAATTTAACTTGTTACAAAAACCCTC
GAAATCTTTATCTTGTTCCGTCAAACTTTTCAGGCATATTAA
>ECGMTA166G_1986394
GAATTCCGAGATGTTTTTGAAAAGAAAAATGTAAATACTCTCCCCGAACATCAATCTTATGATTGTTCAAT
TGATCTACAAGAAAGAGCTCAACCACCGTTCAGACCAATCTATAGCTTATCGCAGAATGAGTTGGATGTAT
TAA
>EAGMCA466G_1787668
TTAACAAGCAATTTTCCAAATAAACTGCAGCTCTTTGCAGACAGAGCACTGAAGGCATCTCCTCCTCTCAG
TATCTGCGTGGCAAGATGCAACTCCAAAGCATCATTTAGAGCATGCCTAATCACAAGGGTGTTGAGCAAGT
CAAAGGATCTCTCATGTAGTTGACCCAACAAGTCAGAGGCAATATCTTCAGAGTCCACCACATCGAAACCA
AGTGCGTCGCTGTTCATTAGACTAGATTGGGATAGATAAATCTGCAGGAAAACATCCAGATTTTCCTTCGA
CAGCATCTTACAAGAGACATCGAACGTCGGTTGATGCCACTCGGAACAGAACAGACAATTTTTCAGAAAGC
TTAATAACAAATTGGCTACTTGGAGAGGAGTACTGTGAAGGGCATCCAACTTCTTTCTTGAGGTACAGGTT
GACGCAAACCCCTCTGAATTC

**Linkage Group 4 Scaffold 58**
>EACTTC425G_953412
GAATTCACATTGATAGATGAATTTTACTTCATACATGGTTATTTTAAATATCAATACTTGTTTATATGAGT
TGGAGGCAATGAATATGTGCTAAGTGAAATTCTTATGACATAGTAGGTATTGAGTTTGAATCTTGTGTAAG
AGCCATCGTTAATGAGTTCTTTATCCCGAAGGTCATGGGTTGGCGAGTAATGCAAGCCAAAACATTTCAGA
CCTGCCCGAATTCTTCAACTGAATGGACCTTTGTTACGGAAATTTACCCAAAGCACATAGTTTTAGGGCTG
TTTCTCTCTGGTCGTCATAGAAAAGTGGATGAAAGGGCCCCAGCCATGAGATTCATGCGGTGAAGGGAGCC
GATGTCACAACAAATTAATCAATGTCACAGCCAAACTGGCTTTCGATCGA

**Linkage Group 4 Scaffold 75**
>EAGMTA294G_1645030
GAATTCAGAGGACAATAAGAAGGTGATATCAGAGCAAAACCGAAAAATTGAAGAGCAAGAATGAACGATAA
ATGAAATTGGAGAATCCAAGTTGGTATTGGAACAAAAATGGATTCACTAATATGAGTCTTAGACGAAAGAA
AAAAAAATATATCTTATGGATTGTGGAAGGACAACGGGATGAATTGATTCAGAATTACAAGATATGAGATT
TGGAATGAAACAAACTCAAATGCCAGTTGCAGGTGGAAGGAGACAAGGCAGAGTTATTAA
>EACMTC280G_762828
TTAATCATTTGTTGCATTATCTAATGTAGGTTTCACTGAATCGGGTTCTAAATTCAAATCTTCTTCTTCCT
CCAATCCGTCCATTGACTCCTTCTTCAACTAGTTTGATAGATCTTCATTACACTCACCCAATTCACGGACT
TGAGCCTCCAATTTCAAGATCTGGGCATCACGGAGTTCCAATTGATGCTAGTGCTGACTCATCTATTCTGG
AATACCTTCAATTGACACTTGTGACTGGCTTCTCATTGTGAATTC

**Linkage Group 4 Scaffold 87**
>ECAMCT298G_1055940

GAATTCCATGATGTTATTATTGACAGTATTAAATGAAAGAGGCAATTAATAGAAGAAGCTAGATTAAATCA
TAGTATTGATGCACACATATATGAAGGTGAAGAAGATATATTTGATAATTATTACAAACAATCAAAAAGTG
AAGGGAGGAGTATAGAATAGGTAAATGAACTTTGTAATAAGTTACTGGTTGGTTATTGATAACCTTGAGAA
TTAGAGTGATAGTTAGACAGTTTAGTTGATAATAATAGGTTGTTAGTGGAATAAATAGTTAA
>ECAMGG380G_1542779
TTAAGGGCCATAGTAGTCATATTATTTTCAATATTATTAGAACCCCATCAAATCTTGTTATTCTTGGTCTT
TCATGGTTGGAAAAGCATAACCCATCCATAGATTGGAAGTTACGGAAGATAACCTTTTCCACAAAACCACC
CAAGAGCAAGCCTAGTAGAAAATCCCAAATAGAAAAATCCTTATTTATTGGAGGGAGAGCGTTTATAAGAT
CGTCCAAGGAAGGCATATTTACAAGACAATTTTACAATGAAGACACGAAAGTTCATGCTTGGTTAGAAGTT
GAAGGAGTGGGCCACTACCGTTGGCTTCAAGGGCCGATGAACCACTTACCTCGTGAACAACAGTTGGAATT
C
>EAGTAA235G_55532
GAATTCAGCAAAAGGAGAAAGGACCAATTCAGAATATAGGTGAGCCTGATGCGAAGTGCCTATTTCATCCA
ATTATGGAGACATCTAATCGAAGTCCTATTACGGGCCTCTTATCCACTATGAGACCTGAATCAGTTGGGCA
ACCCAATGAGGTCTCGATTACAGAGTCTTCGATTCCATTTCAAGCTGTTCCATTCTCTATATAGTTTCGA

**Linkage Group 4 Scaffold 207**
>ECGMGC448_135106
TTAAGCCAAAAGCTTGCAGAGATTGCCAATCCCAATCTCAGCTACCAAAACCAAGTGCAACACCTTCGCTA
ACCTAATGCCGTAACTACCCTCTCCCTGTCTTCCCCAATTTCAGAGCGCACTAAACCTACACGACTTTCAA
CCGCAGTCAATGCGGTTCACACACTCTCCTCCCAGGTACACCATTTCCTGGGTCATTCAACTTCGAAGCTC
TCACATGAAACCCTACTGCAATCCCTCGCCCAATCTCCACACAAAAGCCTGCAACCTGCTTCGTCTTCCTC
TCCTTCTTCCTCTTCTCCTTCTTCCTCTTCTCCTTCTTCTTCTTCTTCGTTCTTTCTTTCTTTCTTTCTTT
CTTTCACCTGTCCTTGCCAAAGGCAGTTCCACTTCTCCACTTCCTCCGCGCAACCGATCCTCTCGGAATTC

**Linkage Group 5 Scaffold 109**
>EAGMTT352G_981589
TTAATTATTAATATCACTATAAATCTAATTAATTAAGAATATTCATCATCTCTATGTACCAACAAAATTGA
TGAAATCAATTTTGATTACTCTTTTCTTCATCCTCTTATTACTCAAAGATGATGACAACTCCTTAAATGCT
TTAGTTACTTAACTAGAGATTTGCAATATAGTTTTGCCAACTATGTCATGATTTTATCAGCTTCAATTATT
TTACAAAATTTTATCTAATATTCTTGCTTGTACTTCGAAAATTCCACAATCTTATGATAAAGCTCTTTGAT
TACTTTATTCCATATTATAGTAGAAAGCAATACAAGCTGAATTC
>EAGTTA190G_291080
GAATTCAGAAAAAATATAAATTTGAGGTACCCTCAAGAAAATTATTGGGTCATATCGTATTGGAAACAGGTA
TCGCCACCAATCCGGATAAAATAAAAATTATACTAGAACTCCAAATACCCGATACAGTGACAGGTGTTCGC
ACCTTTATTGGGCATGTTAGCTATTATCGA
>EAGMGG336G_490938
GAATTCAGAAATGAGAAACGGCTGCGAATTATTCGACACTTCAGGCGGTCACAAAGGAAAGCAGGCTCCAA
CGAGAGTTCGACTACGAACAGAGTGTCATTCGCCAGAAGGACGACGGATGGAAGGAAGCCCAGCAGAGCAG
ATAGCGAGAGGCGAGGAAGCGGAGGTCAAGAGATTTTCATTGAGAAAAATCGACTCTCTGCTCTCAATTAT
CCTAGCAATCTCCTACGAAGCGGCCAAGATTGCAAGGAAACAGATCTGAAAAGGCAAGAAGTGACAATTGG
ACGCCTCGTGATGACTTAGAAGAAACCCTTAA

**Linkage Group 5 Scaffold 300**
>EAGTTA441G_540050
TCGATAAGAGATTGGTACTCTTCTTCCATAGCCTTCTCCTACTTGATTGCATCTTCACACTTCATAGCATC
ACACAAAGACAATGGATCAGCAAGAAGAGCAACATTTATATTTTCTTCACTTACTTGAGAGAAGATGTGAT
TCTTCCACCATTCGCCACGTTGCCTTCTTTCTCTCAAAAGATATCTCCTTTCATTGATTGAAGAACATTCA
CCTTATTCTTTTCCAACTCTTAAACCTTGTCATTTCTTACTCAATGGTTGGAAGTCTTCATCATTGTCATC
ATCCCAATCTTCAACATCTTCATTTCTTTCATTGCTGGTCCTATCAATTATAAGAGCCTTCATCTTTTCAA
CCTTCTTTGGCTTTTCATCTTCATCATCTTCATTGTCTTCATCTTCTTGTTTCTTTGCTGAATTC

**Linkage Group 5 Scaffold 303**
>EAGTTA190G_48362

GAATTCAGAAAAATATAAATTTGGGGTACCCTCAGGAAAATTATTGGGTCATATCGTATCGGAAACAGGTA
TCGCCACCGATCCGGATAAAATAAAAATTATACTAGAACTCCAAATACCCGATACAGTGACAGGTGTTCGC
ACCTTTATTGGGCATGTTAGCTATTATCGA

**Linkage Group 7 Scaffold 7: see also LG30**
>EACMGC180G_466741
TTAAGCTACATACCAATTTTAAATTGAAGTTAATAGTTGAAGAAGGAGAAATTACTACTAGAACATTTGCA
TCAACTTTTAAATTGATAGCCAAGTAACATTTGTGTGGTTTTTGGGTGTTTTCTCACAATCTGTAAGTTGG
ATTGGAGTGAATTC
>ECAMGG300G_1467570
TTAAGGCCCTGATAGTCTACACACATCCATAGAGATCCTTTTTTTTCTTCACAAATAAGATGGGTGCTCCT
GTTGGAGATTTTGAATGTCGTATGAAGTTTTTAGCTAAGTTCTCGTCAATATATTTCTTTAGTGCAACTAA
TTCGTTTTGTAATAAGCTATAGATTGATCCAAATGGCGGTTGAGCTCCTTCTTGAAGATCAATTGAATAAT
CATAAGGTCGATGTTCTGGAAGAATATCTGCATTCTTCTTTTCAAAAACATCTTGGAATTC
>ECAMGG241G_644204
GAATTCCACACCAATGACAAGCCCCCTACAAATCTCACTATAAAAAGAATTTGTCATGAATTTGATTCTTT
GATTAGCTGTACTATTTTCTTACAACTCTAATGACTCCCATATTTTTTGAAAAATAAAATAAAATAAATAA
ATAAATAAAATAAAAAATCTCCCTCTAAAAAGGTGTAAATTAACCCATACATGTGATAATATCTTTACAAC
CTTAA

**Linkage Group 7 Scaffold 30**
>ECGMCG244G_2342986
GAATTCCGCCCGCGTGTGGTCGCCAATGCCGAACGGCATCTACTTCTCTACGGAAGTGAATGAGAGTGGAG
GGTGGGCGGGGCAAGATCTCTCAAGTTATGGCCCGATTGACAATCCCGCCGGCTACATCGAGGTCGACTAC
TGCGGCTTCTGGCAGCTCAGTGATTATTCTTCTTCTTCTTCTACTACCACTACTTCTACTACTTCTACTTC
CCCGTTAA
>EACMGA452G_751964
GAATTCACAGTGTGGTACTTGCCTTGCCTACTTGGACGAGGCTTGCCTCGTGACACTGTGACGACCAGTGC
TACATCGAAACGTAGCTTCTTTCAGTAGAATGTTGGCATGCCTTCAAGGGTAACGGATAGTAAGGCTAAGG
AGATACCACATTATCATACGACGTCACTTGTCTCTCGTTAGGATCCTTTTTCTCAGAAATATGGACGAGTA
TACCGCGTGATCTGTAAAATAGCTCTTCTGCTGCACTGCTCTGTATAACCCGGACGCTTATTCCGTGAATT
CTGAAGTCACTGTACGAATCAAGTAAAAAATGTATAATTCTTATAGCAATGTCTCCTGGTCTTTGTAAAGC
AAGAGACGTTGAGTTTGGTCTTAGCTTGCCAACAGCATACTGAAGCAGTCTTGCAAGTAGTGTGATATAAT
CTTAA
>EAGMGG93G_939879
GAATTCAGCAAGAGAATAAGCAGTATTTTTCCGGTGTTGAAAAAACAGATAAGAGAGGGTCCGCGTCACCC
TTAA
>EACMGG226G_450200
GAATTCACGCCTCTTAGAGGATCTTTCAACAGAAAGGACGTTTCTTGAAACTATATAGAAAACGGAACAAC
TTGAAACGAAATCAAAGCCTCCGTAATTGGTACCTCATTAGGATACACAATCGATTCAAGTCTTATACTGA
GTAAGGAGTCCATGTCAGGACTTGGATTGGATGGTCCCATAATTGGGTCCAATTGGCCCTTAA
>ECAMGG241G_116604
TTAAGGGACATATGATTGAAAGTGTTGGTTTTGTGGAAGAATAGAATTACAAAATTGAGCCAAATGCCCTA
ATTGTTCACATTGAATACACCATAATCCTACTGTATATCTTTTTGATTCTATAGAAGATAGGCAAGGGTTG
GGATTAGAATTTCTATCTAGTTCAATTAGTTTTTTTTGTCATCAACTTTATTTGATGAGAGATTTGGATTT
GGAATTC

**Linkage Group 7 Scaffold 76**
>ECGTAC82G_774797
TCGAACTGGCATAACACCCTCCTCTAAAATAATCTGGTGCTCACACACATCAACCGGAATTC

**Linkage Group 7 Scaffold 119**
>ECAMCG98G_482431
GAATTCCAAAGAACCAATATCAACCAAATTAGGAGAATAATCTTACCTATTGAAAGAACCAATTTTACAAG
CTCCGTTAA
>EACTGA113G_434613

GAATTCACTACGGATGAGCCTTGCGATGACTTCGCTGAATTTGCTTGCATTTTCAATGATCCATTTAGATG
GAAAGAGTAGGAATGTCTCGA
>EACMTC371G_347271
TTAATCTAATAATACTAAGGTTTTTAGTTTTTGAGGGCCAAAAGATAGAAGAATGATAAAACAATGGATAG
GCTGAGCAGAAAGAAAAGAACCTGATTGTTGTTGTGGTATTGAATTGTCCAACCGCATGGTCCTTATTGTG
GCTGGGCATCTTTTTTTTTGTGAACAAATGCACCTCTAATCTTGACAATGGGTCGATTTTGCTTCTTGGGG
CAGTTTCTAGCTTTGTGGCCTTCTTTACCGCAATATAAGCATAGTCTTTTTTCCATACATTGCTTTTTTTC
TTCTAGAGTTAGGAATTTGATATGTTTGACATCAATTTGTATGTCTTCCATCTTTGAATGTGAATTC
>EGAMGC404G_818772_+_790433_inverted repeat
GAATTCGACGTGAGCCTCTCCCATGACTAGTGATCAAAAATCAAAATGTGGCACTAGATTATATCAAAGCT
TGCACTGCTCTTTTTTTCAGTGAGAAAAGATAAAGCTATTGGGAGCAAGATTGTGGGTTTCTAGAGGTACA
ATGATCATGATAGACGATTCTATTACTACGAGCAAGGGTCAGGTTCTCATGTTGGCCATAAGTCCTCTAGC
ACCAGACCCCTCTTGGTCCTCGAGTATGAAGCAATTGAGATGCTCTAAATCGAGTAAAAAGTAGATATCAT
GACTTGGTTACCATGCCCTTCTTAGCCCATTCCACGTAATTATCCAAAAGCAAAGGCCATGCTTGACTTTC
GTCGTAGTTTCTGAGGTCGGGCTTAA

**Linkage Group 7 Scaffold 304**
>EAGMCT521G_84842
GAATTCAGCACTATAGCACACATGTTTCACGTTATTACCTGCCATCTGGCACCGAAAACCTCTTCCAAACC
ACACCAGTTCTCGTATTAGTTCGTTCAGTGATTCTGGACAGCCAGTGAATCTCCAAAGGGCCAATTCATGT
CACATGGGAATAAAATACTGCAGAGAGGCGACAGAGAGGCTTACCAATTGCAGCACATCGCCTCTGGTACA
CACCCTCCAATCCTCCTGCATCGAATTGTGCAGTCTATTGGGGGACTCTCTCCCACCACCGCGCAGGCTCA
CCCTTGGATCCCGCAACTCCGTGGATGACATGTGCTCTTTTGGGTTCCGTTCATTCGGTTGTGCATGGGCT
GTTTTTGAGTGGATGGCGTTCATTAGTGGCTGAGATTGTAGATACCTTCACGTGTTTTCTTTTTTTCTTTC
TTTTGGTACAAATGCAGAACTTTTCATTGCTGGGAGTAACCTACACAGATCCAGGCTGAGCTAGAGTTAA

**Linkage Group 8 Scaffold 57**
>EGAMGA530G_1564003
TTAAGAGTTGTTCCTACCTCTAATATAATCACCAAACATTGGAGTCTCTTCTAAGGTTGTAATACCAAATA
TGTTGTATCTTGCCATCTATTCGAGGAAGGACATGTTGTTCTTGAAGATCTTCATCTCAATTTTGTCATCT
TGAAGGTTGTGACAATTGCAATGTTTCATTTGGTAAATCCACAAATGAAAGCTCTTCAGTTGAATATGCTG
ATTATTAGTTTTCCATCTCCTTAGTATCACAAATATACACAAGCTACAAAGAAAGATAAAAAATAAATTAT
TATCTTCCTTTGATCTTGTTTCTTTTATAGTAAAGATTTGTTATTCTTCATCTTAGTTTTGATGGATTTTG
TTGATGAAAAGCTTCAACTTTCTTTGCTGTATTTACAAGATTCTTTGATGGCTCCCATGTGCATTCACTAA
TATCATATCTTTGCTAATGAACAAGATATTTCAACCTATTTTGGCATTACCTTGAGTCTAAGACTTCTTCC
ACCTCGAATTC
>EAGMCT123G_1355845
TTAACTGCTGCATGCATCGTTCCTTGTTACACACAAAAACAACAACAAACAAGTGCTTGCTGTCTCTCCGT
CTCTATTGTCTGTGCATCTTACACTGAATTC
>EACTAG358G_142420
TCGAAGGGATAGTAAGGTAAACTGTGGCATAACTGACTCACTTTCCTTCCGAGTAATAAAAACTTGCAAAA
TTTCTATCGCACCTGCGGACACTTTGTAGACGAACATCACATGTCCCGTCAATATGTAATGTGTTTACACA
CTGACGTGAAGAATAAGTACCAGACAATGTTCTTGTGCCGAGAAACGTATCACTTCTCACGTGACTCAATA
GAAGGTGCATAGCGTCAACAAAATAGTTGCAGAACTTGAAACGAAAAAAGTCTGGATCAAATGGGTCTTCC
CTCTGCCATGTGGAATCCCGACCAGACAGTTGAAAACAGGGTTGTGAATTC

**Linkage Group 8 Scaffold 129**
>EAGTTA428G_921248
GAATTCAGATCATAAAAGAGTCAAGATTCTTTTTCCAACAAAGATGCAAATCAAGAAAAAAAACACTCAAA
TAGAAGTACACCTATAAATCCAACTTGAGTGCATCTATAAAGATATTGAGAATGATTCAACTTTAAATAGA
ATTTTATTTATTGTAAAAATCTTTGAAGCTTTGATCTTGTTCCATCAAAATTATGTATTACAAAAACTTAG
AGATGCAAGATTGTACGCCAAGATGGAAAAGTGTGTATTTCACACTACTCAAGTTGACTTTTTAGGATACA
TCATTTCAAATGATGGTCTCATGATGGATTCTAAGAAAGTCCAAACAATCATGGATTGGAAGATTCCAAGG
ACAGTAAGAGATGTCCAATGTTTCTTGGGATTTGCCAATTACTATCGA

**Linkage group 9 Scaffold 8**
>ECGTGT303G_2982677
TCGAGTGGAGAGAGACTGTTATGAGCACCAACGCCCTCGGCAGGATGGAATGTGCCCGGAGATGATTTGCC
TATCAAGCGCCTACAGTGGTTATGTTTCGCACTGCATCGCACGTCCAAGGCCAGTGCTACCCCATTGTCAC
AGATGGACGCAGTGAAGTAGTTTGCGAAAAGCGGCTGAGGTCTTACGGCAACTGATTTCCTAGTAAATCCT
TTCGTGTCTTTGCGATTGCCAATATATTTGTCGTCACCGTCATCTTGGAGGAGGCATCTTCGGAATTC
>ECGTGT305G_2982677_Variation
TCGAGTGGAGAGAGACTGTTATGAGCACCAACGCCCTCGGCAGGATGGAATGTGCCCGGAGATGATTTGCC
TATCAAGCGCCTACAGTGGTTATGTTTCGCACTGCATCGCACGTCCAAGGCCAGTGCTACCCCATTGTCAC
AGATGGACGCAGTGAAGTAGTTTGCGAAAAGCGGCTGAGGTCTTACGGCAACTGATTTCCTAGTAAATCCT
TTCGTGTCTTTGCGATTGCCAATATATTTGTCGTCACCGTCATCTTGGAGGAGGCATCTTCGGAATTC
>ECAMCG60G_2582538
TTAACGTTGAGGTCTACTTGTATGGTTCTCAGATTGGAATTC
>EAGMTA319G_1301076
GAATTCAGAAGAGGTTCTACAAAAACAGGAGGGTGTACTGAGGACCAATTGCATCGACTGTCTAGACCGCA
CAAACGTCACTCAGGTATACGATTCTGATTTAACTGAATCCATATATTTTTGCCTGTTCATTAATTCAACA
CTGTTATTTTTTTTTTTTTGTGGAGGCATGCTCGTTTTGTACTTTGGAATGGAAAAGTCCGTCCACTTAGG
AATGAGTAATCCCAGGTTAAGAAAGACTTGCTTGTTCGTGTATCCTCAACGTGGAATTAGAGACTTCAGTT
GGTTATTAA
>EACMCG342G_2523935
GAATTCACCTTGTAAAGATCCATGATGACAGAAGCTCTCGCTTCTAAAAAGCATTCATAGCTCAAACTCCT
GAATGAGCACAATGTCTCACAAAATCAGCTCCTCAATTCCACAAAACTCCACAATTCAACATCTGGCACCA
CTACGACACCTCACAAGCAACACAAAACTCTCCAGCAACCTAAACAATCAACATAAGAGAACACAATTATC
GCTCCACAGCTTCCATCAAACCATGCAAATAGCTCAAAAAACCCTAAGACAAATATTCACAGGACCCCCCC
CCCCCAACTAACAGTAGAAGCAGAAGATTCACTCCGTTAA
>ECGMGT131G_2784713
TTAAGTTATGGCTCCAACCGTTACTATCAATAAAATGACTCATTTACTACCTTTGAATTACATATGTTGTC
TTCAATGCCCTATAACTCCTTCATCTAAACTCGGAATTC
>EAGTTA336G_653995
GAATTCAGCAACATATATAATAACTGACTGTGATCCTTATTGTAAAGATAAGAGCTTAGTTTCTACTATTC
TTTTTTGATCACTATCTCTAAATATAGCCATAAATACTTTTAAGAATTTAAATCATAAAAGAGTGATGAAT
CTTTTTCTATTAATGGTGTAAACCAAGAGAAAGCACTTCCAGATAGTAGTTCCCTTATAAATCCAACTTGA
ATAAATTCATTAAGATATTGAAAAAGATGCAAGCTTAGATAAAGCTTGACTTGTTGCAGAAATCTTTGAAA
TTTTGATCTTGTTCTATTAAACATATCGA

**Linkage group 9 Scaffold 62**
>EACMCC149G_1697951
TTAACCAAATTAGGCTAATAACCTTATGATTATACAGCTCTCAGAAACTGTCAAGCACAAAAATAAAATAA
AATAAAATAAAATAAAAGTCGGATGCGCAGATGTGTTTACCTTCGTCAGTGAATTC
>EAGTCA292G_1344639
GAATTCAGCAACATAACTCAACTCTGACACGCCTCTCTGGGGTAACTGTGGCACAAGTTTGGGGGAAATCA
CACACTGGATCTGACAATGTGAACACATTAGTTCACGGAACCCAATGGATATGGGACTGAGTTTGCTGGAG
GACCGGATCAACGTTCCTGTTGAGAAGGAAAACTGGGCGTAGTTGTGGAGTGATAGCTGTGATCCACAAGC
ATCCTGGAATATCACAGCGCACTCAGCGGTGTTGGCTGAGGTTGTGTGGTTGTCGA
>ECGTAC209G_818128
TCGAACATTTTGCCCATGTTTTCACACCTCAGCAGGACTAGAAGGTGGAAGGATTGTGCGCGGTTAGCTCA
ATTTGACTTTGGGTCTGTAAAAATAACTTAATGAGAGAATTACTGGATGGATTGTAGCAACTTGGCTGTTC
AGCCTCAAAACTTTCCAGACTCTGCTCTAAAATAGGTCGGAATTC
>ECGMTA167G_902301
GAATTCCGAGATATTTTTGAAAAAAAAGAATAGAGATATTCTACTAAAGTATTGACCTTATGATTATTCAA
TTGATCTGCAAGAAGGAGCTCAACTACCATTCAGATCAATCTATAGTTTATTGCAGAATAAGTTGGTTGTA
TTAA
>ECGMCC475G_107184
TTAACCAATACTCTAAAGATGATGGATTGGTTGAAGGCAAAGTTAGAAAAAGAAGATAAGATGAGTGATTT
GGAAGAGCTTCACTATTGCTTAGGAGTGGAATTTGTAAGAGATCGTGCTAACAAGACCATCACAATGAGCC
AAAGGAAGTACATTGAAGGAGTGTTGAAACGATTCAACATGGAGGATTGCAAGCCCATTGGAACACCTTTG
GAGTCCAATTTGAAGTTGATGAAGCTCACGGATGAAGAGTTTGCACAAGTTGAAGGCCAAATGTAAGGTGT

TTCATACAAGGTAGCAATTGGATCACTCATGTATGCAATGGTTGGCACAAGGGTGGATCTTGCTTATGCGG
TGAGTGTCGTTAGTCAATACATGACAAAAGCAGGACCACTTCATTGGGCAATCATCAAGAGGATCATGAGA
TACTTGAAAGGTACTTCGGAATTC

**Linkage group 9 Scaffold 114**
>EACTGA537G_284829
TCGAGAGCTTGATTGTCTTTACACCTTGAGAAGCTGTTAGGGTGCCTCCTAGTGTATTGGGGGTGATTCCA
CATGCCTCTATTAGGGTTTTTGCATTGGGTTTCAGCTTATTGACTGTAGCTCTCAGTTCCTCATCTACCGT
AGCCTTGTGTGTTAAGTCCAAGTGCAGGTTTTGTTTAATGTAGTCATCGGTTTCTGTCAAGCGCTTTTGTA
GTAGCTGTTTGATGTTTTGTAGTAGTTGTGTTGTAAATAGTTGTGCCGTCTTCTCTCGGTTGAGATCAAGG
TCTTCTAGGGCCTCAGTAATAGGAGTGAGGGATGGATTGTGGTTTAGGGTTGGTGTGGTGAGACTCTGTTG
TTCTCGGTCTTTCATCTATGATTTCGTTCCCTCAGGCTAGTGGGTGGGAGACTAGAGCTGGTCCACTATCA
GATTGTCGTCTATGGTGTATCGGGGACTGTCCTCTAGGTTCCTTGCTGTATGTGTACTACCTTAAGCTGTA
ATTTGCGCTGGTTGTGAATTC
>EGAMGA86G_389362
GAATTCGACTTGAGTAGTATAGAATACATACTTTTTTTATCTTAGCATAGAGTCCCGTATCTCTTAA
>EACMCG69G_198148
GAATTCACTGAGCAATTTTTGTGTCCGAATTGACTTGCCGACTGCGTTAA
>EGAMGA232G_142757
TTAAGAGTTGTTCCTACTTCTAATAAAATCACCAAACAACGGAAGTCCCTTTCAAGGTCATGATACCAAAC
GCGTTGTATCTTGCCATCTATTCGAGGATGAATATGTCATTCTTGAAGATCTTTATTTCGATTGCATTGTC
TTGAAGGTTGTGACAATCATAATGTCTCGTCCAACAGGTCAGCAAACGAAGGCTCTTGAGTCGAATTC

**Linkage group 9 Scaffold 134**
>EACMGC198G_813200
TTAAGCCCAAAGAAAAATAGGAATATATCCACCAAAGAATATAGCATGAATTCAATTGAAAACAAAGTGAT
ATTTTCTCTCAAAGAACTTGTTTTGTTGGGTATTTGGACAATACTCAATTGTTGTAGGATTTTATGTATTA
GAAGATATCAATCAAATTCTATAGAAGTGAATTC
>ECGMCG121G_803950
GAATTCCGGAACATTGCTTTACGGTGCCCAGAATTTCAATCTATTTTTAGATTTGAAAATACCACGGTATT
TTTTCGAGGGTATAGAAAACGATCGTTAA
>EAGMGG277G_145589
TTAAGGTTGCAACCTTTCCAATACCCTGATGGATTTTTCAAGTTGGATTCATAAGTGCACTTCTATCTAGA
AGTGCTCTCTCTTAGTTTGCACCTTTGATGGAAAAAGATTCACGACTACTCTATGATTTGAACTCATTCTT
GGAAGTATTTATGGCTACATTTGGAGAAAGTGATCAAGAAAGGATAGTAGAGACCAAGCTTGTATCACCAT
GACAAGGATCACGTTTAGCCACCATGTATGCCGCTGAATTC

**Linkage Group 10 Scaffold 18**
>ECAMCC120G_2696473
TTAACCCTAATTCTCTCCTCTTTCAAACCCTGGCCTCTGAATTTCTTCAATCTTCCGTCTTGGATCTGGAA
TTGGCTCCTTCCGTCTTGGATCTGGAATTC
>EAGMTA431G_1016425
GAATTCAGATTAGTTAGAGTGAAGTCTGTTATTTCTAGTGTTGGATGGAAATTATTATGTTTTTAGTGGTA
TTTTGAGAGTGCAGGGTTACACACTGTTGTTATTAGTTGATGAAGGAGTATTGTTACTAGGAGAAAGTATC
ATAGTATTTTTTAGTATTTTAGTAGTTCGGAATATAGTTAATTATATGACCCTAACAAGTAAAATTCATGC
ACTTAGTCTTTCCTTATATTTGAATTTTCCTACACAAGTTTATTTCTGAAAGAAATTGTAGAATAATTAAA
ATGACAATCACAGTTATACTTTCAGAAATGTCAAGACTACAAAAAGTCCAATCTATTTTAGGGTTATATTT
TTCTAGCCATGATAAACCTAAAATGATTGGATTAGAACGAGGTCTAATGATATTA
>ECAMCT367G_305381
TTAACTTACACATACGATTGTCAGGACCTTTTTCTTAACTATTTACGTACTTTTTACTCTGAAAGTAGCGT
CTAAGCGGTGCTTTCATTTTGTTTCTTTGTGTACAGGAGCTGAAATTCAGAATTGAGACAGTCAAGAAAGA
ACAAGCAGCCAACACCGGATGTGCCAGCGAGAACGCCAGAAATGAGGAGCTTCTGAAGATTTTGAGAGATA
TTGTGACTTTTCATGGAGAAATGGTTCTCTTGGAGAACTATAGCTCGTTGAATTATATAGGTAAATCCTTA
ATGCTATAAAATAACATGCCCCCCCCGGTTTAAACGTAGGCGGTGTTTTTTGGAATTC
>EACTAG159G_677022

GAATTCACCACACAACGGCACCAAGAGTATTGTTCAATCGATCAGTAGGTCTCGAACACATACATCCTAAT
AGCATCAGCGGAATCCGAACCTGCGGCACGAGCCCACAGAGCGATTCCTCGTGATCGAACCCCCTTCGA
>ECGTGT179G_2252746
TCGAGTTCTTATCCTTGACAGCTAGCTCTTTGACCCCCAAAATCCTGATCTCCGAATGCTCCTTTCTTTAT
CTTTTTCTCTCTTTTAACTCCGTGTGGTCCCTTTTTTTCCCGCCTTGGACTCCGAATTGGAACAGCCGATG
GTGCTTCCTCGGAATTC
>ECGTCA46G_671071
GAATTCCGTGATCAGTTTGTGTGTCGA
>ECGMCA297G_130943
TTAACAGAGTGGAGGATATGCTTTTCTTGGGTGTTTACCGCAAGAATCTCGGCATGAAAAAAACGAAACCC
GTCGCTCTGCCAGTTTTACTTCTTCATCCGAGACTCTGCGGTACGCGGTGTTGCCCTGGGGATGGAGCGTT
CCTCCAACTCCCAGGTCACTGCCTGTTGGTAGAAAGCATACGGTCTCGATGAACGGTTCATGAATCTCGGA
TAATCAATTGCAAACCCCAACCCAGACACGCAATTCTCACACGCATTGCCAAAACGGAATTC
>EACMGC375G_613025
GAATTCACTCCTCTCGAAGCGACACAAACATAAAGCGTTAGTAGTCATTGGAAAGCATATTCCTAACGAAC
ACCAGTTGTAGCACTTGCTCAAGTTTTCATAATCGTGTTCAGTGAAAATGGCTTTTACCTCGTTTCATTCT
CAGTGCGTGTGCGTACTAACACAATTTTCTTCATCTTTCAGACTTGACGAACGGAAACGTAGGAAGAACTT
CATACTAGAAAGAGGTCTCTTGAACATCAAGCGACAGCAGGTCCTAGATAGGAAGCGGAGTAAAGAAGAAA
GAGAATTATACAATCGCAGCCGAGTCTTCATGCGCTACCATTCAGCGGAAGAACATGAAGCATTGCTTAA

**Linkage group 10 Scaffold 73**
>EAGTAA197G_102328
TCGAAATGTAGACAGTTTTATGAGTTTTCCTAATTTTTATGAGTGGCTTCTCCCGCTATACGCGTCTAGAC
TTATGTTTTTGAAAGATTATTGACTTTGATTTCATTCCGAGCGGGTGATGGTTGGACTATAGATTTTGGTT
TTCTTAATGAACACAGGTGATGTATTGCTGAATTC
>ECGMGT292G_238930
TTAAGTGATACCGCCGCTGGGTTCCTCTCGTTTCGGTGTGTGTGCTCACTGGGCACGGTTTACCGGATTGT
AGTCGTGCAATGAAGCTCGCTTGAGTTTTTTGAGGTGAGGATCCGGGGTACATTGGCGGTTTATGTGTATG
TTTTTGTTTTTTTTGCTGTTTTGGTAGCGGCATCGTGGTTGCTGGAGGTGTATGTGGGGGCGTGTGGGAGA
TTGCCGGTAATGTGGTTTGGTGATGGTGATCGCGTAAGGGATGAATTAGAGTCGGAATTC
>EGAMGC475G_1060510
GAATTCGAAGAACTCTCAAGCACGAGATAATATCCAGGATTGCGTGAAAGATCATGGGCGTTGACTATCGA
CCAATAAGTATCGCAATAAACACTGAACAGATCACTGCAACAAGACTGAGGACGTCACAAAGTTCGCGAAC
AGAAAAATACTTGAGCACGAACAAATCCGCACAAGGCGAATGTGGTAAACTGCCCAGTGTAGACGCCATTT
TCGTCAACATGACCCACGTTCAACTGAACAGAGGCAACGTCCTTGGAAGTGATCAACCTGTTGGTTGAAGA
ACTGCGAGCACAAACACGACGCCCATTCCAAATTAGTCTACCCGAACCCAAGCACACTACCTTGAAATTAG
AAGAACTAACCACAGACCTTGGCATCCACTCACTTCCTCGAGAAAAACCACATTACGCGCAGTAATCACAC
CACGCAATTCTAAAACCCTCTAAGCTTAA
>EAGTGC239G_1007629
GAATTCAGTTCCAATTAATCAAGATAAAGCAGACCACAGATCGTCTTCACGAATTTCTTACAGGAGAATAG
TCATGTGAACGAAAATTTGGTGAAATCTGTTGGGTGCATGGCTTGAGGCGAAAGCAAGTACTGGGCATGAC
TTCACTCTGCAGAGATGTCAATGAGCAATCACCTCACACTAGTTTCAAATCGTTTTGTCGGCGCACTCTGC
TCGA

**Linkage group 10 Scaffold 101**
>ECAMCT351G_357233
TTAACTCTCCACCAAGATTATCCTGGGCTTCTTCAACTGTGCCAACAATTTCGCGTCATGTGTCTCAACGG
AGAAAATGTAACCCCACCTTCAGCACAATAAATACGTTGATGAGGTAGCTACCAAGCGAAATGAAATGCCC
TAAGTGTAAGCTATGTAGTTTACTTTTCCGATCTCTCGCTACGGCTAAGAATAGCCTTCGCTAATCAATGT
GCTACTTCTAATTCATAAGAAAGTTATCCTCATCCCAGAGCGAAGTGCTTGAACTTGACCGGCTCCCTGTG
CCATACTTGTCGCAGCGGCAGAAATTACAGATTTAGTGGAATTC
>ECAMCC344G_628588
GAATTCCAGTTTCTCCTGCAGTAACTTCTATTGAAGGCTCCTGAGATATTGCTGGTTCCTAATTGCAACTC
GCTTCATTGTCTCTTTATGCATTTTCACTTGTACACTTAGTAGAAATTGTGCGAAGCGATTAGTTCCAACA
CGATTCTCACTGGATTCGATCTCTCTCTCTCTCTCTCTCTCTCTCTCTCTCTTCACGCCATTCCTTTCTGT
CGCGATTCCCTTTGTTATCAATGTCCACGAGCTGGACTGAGTGATTTTCCCAAGGTACTTTGTTATGTATA
TCTTTCTCAAAAGACGTGTTGTCGGACCTTGGTTAA

>EACTTC339G_1377551
TCGATCCGAGAGTCGTACACGGGAACCTCAACATTATTACTCAATGGCAACATCAGCATCTTCGTCAAACG
TACATTCAGAAGTAGAAGACATGTAAATTGACGCGGACTCCTTGACCAGTTGAATCAAGCTAAGATTTATA
CAAAAATAGATCTAAGAGGAGCATATAATCTAGTACGAATCAAAGAAGGAGATGAATGGAAGACTGCGTTT
TGCACCAGGTACGGACATTTTGAATATAATGTCATGCCTTTTGGACTCACAAATGCACTAGCTGCTTTCCA
ACATCTCATGAATGATGTATTTCGTGAATTC

**Linkage group 10 Scaffold 196**
>ECAMCG364G_276523
TTAACGGGTGGAAGGTGTGGAGAAGGAAGGGAGTTTCATTTGTGCGGTGAAAGGATTGGAGGTGAAGAACG
GAGACCTAGTTGGTGGCGAAGGAGGAAGGGGGGCGGTTTAGGTGCCTGTAGAGGAGCGGTGAGTTATGTTT
TTGAGGTGTGGGATGCGGGTGGCGGTGATTGGTTTTTTGAGCTTGAGGTGGGTTTGGCGGCTGGTAGAGGG
TGGTGGCGATGAACGCATTGTTATGTGGAGTTTGGTTGGGTTATTGGAGTTGGTGGGGTGGGTGTTATGGA
GGGGCTTTTGGAATTAGGGGGCGGTGAGGGAAGCATTGGACAGGCTCGGGCGTTTGGAATTC

**Linkage Group 11 Scaffold 12**
>EAGMCA596G_1077745
GAATTCAGACTGTCTCCCGAAGGCTTCCTCTGATGACTTGGAGCTGCCCTTCCTGTGATAACCACCGGAAG
TTCACTCAGTGACCACCACGGACGGTAACAACCTCCAATCATGTGAAGATCGCATCACATGTAAGCTGAAA
CCGCTGAACTATGCTACTCCAGATCCTAAGGTCATCCGTTGCTGGACACCATCATTCAACCGTAACACACG
GTAGTGAGGAGGCTATAAGTAATGTACCTGTAATGAAAGGCAGCTTCGTCATGTTTGCTTGGCCAGTCTGC
GGCTTATCCGCGGTGGTGGAGAGGTTTTTCTTCGAAGCGAGGGGAGCAGCGTATCGAGATGGCACAGCTGA
GAACTGCTTCAGTAGGCGCGTTACATTTCGACCATCCGCGGTGCTCTTTGCTGTTGAGAGGTGATCCTGGC
TGAAACGCACCAGAACCGAGGTTGAGGCTGAAACTAGGAGCAATTCGTAACAAATCGAAATGTAACTCCAC
ATACCTCTGCACACTCAGACGGATTGCGCATCTACCTGGAATGAAACTGTTTGGATTGAGAGAGTACCTCT
TGTTAA
>EACMGT127G_2362572
TTAAGTGTGTATTTGATGGCAAAGTAGCAAAATATTTGAAAAGAATGAAAAAAATTCAAGATCTATTATAG
GTTGTCTTTCTTTAGTACTTAGAGAATGTGAATTC
>EAGTGC349G_831561
GAATTCAGAAAAAATCACTACCCCAGCACATCTTCAATGACACCTTCCAACTCAACGTGTCATTTGAATAG
AAACAAGAAGCGCTAGAATTGCATTCAAAAAAAAAAACAAAAGAAGATGCTTCTGCCATGTTAGATTATCA
CCACCAACCCCAGTTGACCACCGCAACTTTAGCTACTCCAATGCGTCTCCGCAATATACCTAGAAAGAAAA
ATTTGAATGAAAGGAAATTAATATTGTAGTATTGTTGCTGCATCGCAACGTTAGAAATACTAACCACTTGT
TGTATCATAGCAAAAGAAGAGAAGGATTACAAATTCAGAGCTCGA
>EACTGA508G_830904
GAATTCACAATTCGTGGATGCACAGCTTCAATGTGGAGGTCTGGTTGCAATGCACACCGAGCAATAATAAA
ATTGTAAAATATTGAAAGTGCCTTCCTCTCCAGTGTCCACTTAATTGAAGCACTTTTTTATTGATGATCAA
AATTACTCTATCAATGGTGTAAATAAGCTCACAACCACCCGTTATTTTGTCTATCTAAGGTTCTCCACTTG
CTTCATAGAAGCCTATCTTTGATGTCAAATGTGTGTTAAAATTGCTTTCCCAATTGGATCTGCTCCTACGT
ATGCTCATCGGAGTCAAAGACTGTTAATTGTCTTTCAATACATTAAACTCACAAGTGCACAAAGTGAACAA
TCTCTTGTGTGAGGAATGTAAAAACACCGCTCATAGGCCAATCATGGCCTTGCTGCTCAACTAAGTTCTAA
ATGAGCCTCTATAATCAAAGATGATGTGCAGATGGCTATACCATTTCCTCCTCTCGA
>EACMGT136G_830904
GAATTCACAATTCGTGGATGCACAGCTTCAATGTGGAGGTCTGGTTGCAATGCACACCGAGCAATAATAAA
ATTGTAAAATATTGAAAGTGCCTTCCTCTCCAGTGTCCACTTAA

**Linkage Group 11 Scaffold 71**
>EACMGT596G_559169
TTAAGTGGCATTCCAAGTCAATTTCAGGAGTATCAAGACGTTTTTGAAAAAAAGAATGCAGATATTCTTCC
TGGAATGATATGAGTCCTAAAATTGTCCATATGATTGTTCAATCGACCTCAAGGAAGGAACTCAGCCACCA
TTTGGACCAATTTACAGTTTATCGCAAGATGACTTGGCTGAACTCAAGACATATATTGACGAAAATTTAGC
GAAGAATTTCATACGCTATTCAAAATCTCCAGCAAGATCTCCAATTCTATTCGTAAAGAAAAAAGATGAAT
CTTTACGAATGTGTGTTGATTATCGAGGACTCAACAAGATCACAATCAAGAACAAGTATCCATTACCTTTG
ATATCACGACTCCTTGACCAGTTGAATCAAGCTAAGATCTACACAAAAATAGATCTAAGAGGAGCATATAA
TCTAGTACGGATCAAAGAAGGAAATGAATGGAAGACTGCATTTCAAACTAGGTATGGACATTTCGAATATA

ATGTCATGCCTTTTGGACTCACGAATGCACCAGCTGCTTTTCAACATCTCATGAATGATGTATTTCGTGAA
TTC
>ECGMGG281G_1035
TTAAGGGACTTGAGGGTGCCAATGCCAACCTGAATGTGATTTTCACTTGGATCATCGTCGACGTAACTCTT
ACACGTATCGATCGCTACTAGAAAGTGTGTACCCCTGGAACAAACTCGTTGTTTCGTACCGGATGACGGAT
CCGTTACGTCAAGGCTGAGTCGATCACGGTACAAACTGGAGATGTTTTCTGACGCAAAGGAGAAGACGAAG
CCACTACCATCCTTCTGCCAGCCACTGCTGCCGATTGCGGAATTC

**Linkage Group 11 Scaffold 92**
>ECGTGT113G_512867
GAATTCCGCAACCCAGACTCACGACACTAACTTACTGTACTCTCGGGGTCGTCGGGGTCTCCAGAACCCTT
CCGAATCACCCGGGACTCGA

**Linkage Group 11 Scaffold 100**
>EACMGT203G_100039
TTAAGTCGATGTTGTCTTTGGCGTATTGGTGTAGCATTTTCTTCCAATATAATTTGATGCTCACAAACTTC
AGCTTTATTGGGGTGATCATGGTTTTATTGGGGCGATCATGGTTGTATGAATTGGGTCAGGTGCACCAATT
TCTTCTTTTGTACACTCATCTTTATAATTCGATGTGAATTC
>ECAMGG160G_612289
GAATTCCAAGTTCGAATCTACGTAAGTGTTTGAACTTCCGAGATGACGACACTTCACAACGTTTACGTCGA
AACTTTTCTATTTCTCTCCCCCTTTCTCTTTTTTTCAACTTTTTTCTTTTTCTTTTGTTGTCCTTAA

**Linkage Group 11 Scaffold 121**
>EACTCC43G_646502
TCGACCTGCACGTGGATGTGAATTC
>ECAMCG180G_317722
TTAACGTGAGTTGAGTGCTGAATATTCACTTTGAGGCTCCTTTTCTTTCTTCTTGGTTATGAATGAAGACA
TTGTTGATATAGTTTATTATTGATCTATAGTTTGTAAACTTATACAATTAGGTCAGCCTTGATCCAATATT
TCATAATTTATTGGAATTC
>EAGMTA208G_402814
TTAATAATCTATTTAATCCAAGAAAAATAATACTAAAAGTTAAGAGTGAAATTAATACTAACTACAAGATT
TTTAGGAGCAATTTAAAGACCCTTTAGCACTATTCCTTCAAATCTTGATAAAATATTAAACTAGCCAAAAT
TACACATAGACAAGCACTTGCCTATAGAGTAAGAACTCTGAATTC

**Linkage Group 11 Scaffold 162**
>ECAMCC207G_828128
GAATTCCAAAGCTAATTTAGAGCTTCTTTTAGAAATGCAAGGCTTTTCTGTATAATATTTGTTCAAATTTA
GGGATTGTATTGGCAAATTATGATTTATGCAATACAATTAGGGCTACCACTCTTGGCTAGTTTATAAAATT
GGTGTCACTTTTATGGTATAAAATTTGATAATATTTGGTTAA
>EAGTCA176G_37080
GAATTCAGCATTACAATGCAATCTGTTGCAGTGATGTTGATTTATCAAGATCGGCAACAAGGACCCAGCAG
TTACTCATAGATCCCATCGCTGCTGTGAATGTTTGCTAGACATCTGATTCAAGCTTCCTAAATGCTCATTT
GGTATGTGTCGA

**Linkage Group 11 Scaffold 251**
>EACMGC421G_20384
TTAAGCATGAGTGGAGGGACTAGGGTTTTTTGAGTTCAAGTTAGGCATATAGGTATGTATGCTATTTGATT
ATCATAGCACAGTTTTCTGCACTGTTGGCCACTGAAAATATTTTTCCAGTTGTGTTATTGTCTCATCATTA
TAAATATTGTTGTTTAGGATGAAGTGATATTTAGTAGCTCCAATTGAGAGAATTGATTTCACACAAAACAT
GGAACATGGAGAGAAGAGACCTTGCTATAAGCTTGAGTTGGATGGACCCAGTTGGCAAGTTACAGTCGATT
CTTGGACTCACGTTTGGCGTTGGTGGAGCACTAGTATGGGTGTTGAGTAGAATCTTATCAATTTGTAGATA
TATAGCCAAATTAGACTTATCTTAGAATACATTTGGGTGAATTC
>ECAMCC467G_949
TTAACCCTAAACCCTAAACCCTAAACCCTAAACCCTAAACCCTAAACCCCTAAACCCTAAAACCCTAAAAC
CCTAAAACCCTAAACCTGGGGATAGGATTTAGGGTTGCACGCATTCAACAATTTCTTTTGGAGCCCGCCGA
GGTCGTTGATGTCCAAGGGTGAGTTTTCGGGGTTTTCGATTGCAAATGAGGAGTCGAGGATCGAGGCGGGG
AGCAGTGAAGGAGGCTTTTATGAGGAGGCAGGAAGAGAGCAAGTCGAACGGACATGCGAGGGTTGAAGTTG

GGATCGGCGTCACTGTTGGGGGCCGAGGGACCTTGTCGCTAGGATTACAAACATTTTTCTATGCATCGTTC
AATAATTTTCTGTCATGGAACAACGAAGACGAGGAGGCGAGGCCAGTGATAGATATGTATGGGTGCAATGA
GTTCCACGTGTTGGAATTC
>ECAMGG319G_466439
TTAAGGCCAATGCCACAATTCGTGCCAGCGCTGCGGAGGTAACTCCAATCTCCTCATGCATTGCATTGCAG
CTTGGTTTTGATGGTGCTCGTAGGAGTTTTTACATAGGTTGGTGATATGAGTTACGTGGAAGCGAAACAGA
GTGCACCTTGTGCAATTTTGTTGGTGTATGAAGCTCTTTGGGGAGGAATGCCATTCAGATACACCGAATCA
TGATGTGGGTAATGTGTATTTTTTTTTATTTATTTATTTTTTTTTTGCAGGAATCTAAGGCAGTCAGGCAA
GCCATGGTTGGAATTC

**Linkage Group 11 Scaffold 266**
>EACMCC167G_364774
GAATTCACCGCATATTAGATTTTCTCAATATATAATCACCGCTCCTCGTGGAACTCGTCCCTAGAAGGGGA
GGTAATGTCACGGACATACATATAATTACCTATATGAATACATCAAAGTATGTAAAAGTAGATAAAAGTAG
GTTAA
>ECGTGT169G_67155
GAATTCCGAAAAGAGTGAATCTACACATGTGATAGATTACAACTCATGATAAGTAATCAATTACAACTCAT
GATAAGTAATCTCATGAGAAACTGGAATGGCTATTGCCAAAAGACAAAGAAACTAGAGATCCTTGAAACTA
GACTCGA
>ECGTGT170G_67155_Variation
GAATTCCGAAAAGAGTGAATCTACACATGTGATAGATTACAACTCATGATAAGTAATCAATTACAACTCAT
GATAAGTAATCTCATGAGAAACTGGAATGGCTATTGCCAAAAGACAAAGAAACTAGAGATCCTTGAAACTA
GACTCGA
>ECAMGG319G_267777
GAATTCCAATCAACTCTATATCTTCACTTCCAACCAATTGTATTATTTCTTGTTATAGTAAATTGAATTGA
TGCATTATACATTATCATATGGTACAATTTTCTATCTGGGAATACACCGAGTACTTAGTTGTAGAGAGTTC
AGCTTTAACTTATTGGATAAGTTCTAATAAATGATCTATTTTTATTATAGAAGCCAAATAAATATAATAAT
ATTAAGAGATATTTTATTAATAAAATCTTTTTATCTTTAAGAAGCTTTATACGTAAGGCTTTTAAAGCATC
TAAAAATCCCTTAA

**Linkage Group 12 Scaffold 28**
>EAGTAA195G_109204
TCGAAACCACTAAAATACCAAAAAATACTACAATGCTTCCTCCTAGTAACAATACTCCTTCATCAACTGAT
AACAATAGTGTGCAACCTTGCACTCTTAAAAGACTGCTTAAATCTCATAAATTCCATTCAATACCAGGGGT
AATGGACTTCTCTCCAACCAATCTGAATTC
>EACMGC560G_109199
GAATTCACAGAATTTCTGTAATCACAGACTTCCTTTGTCGAAACACACATACAACTCTCAATAAACAACAA
TCATAATCTTCTATCCATAATCAATCTTCCAATAACTTCTCATATAATTCAATCACATAATACTCCAATTA
CATAATCAATAACTCATAAATCAATCTTTGATTTATTCCACAGACTCATTTCTCATTATTCAAAAACTATC
CATTACTCTTCAACAACAAAAAAAATAAAGAAACAAAGAGGTAGTACCAAGCTTAACCCTGGCTCATGACT
ATCACAATCTAGCCCTCCAACAAACTTCTACAACTCTTATATAATTGCTACTGATTTCTAGATTCTAATCG
TAATCCTATAATAACTGCTTTTAATAACTAATTTAACCACCCATCCTCATGAACTGACTATTCTCTGGACT
ACTAATCTATTTTACTAATATCTTATAAAAACAAACTAATATCTATTTTATAGTTTATTAAAGCTCATTGA
TTAGAATTGGAACTTACAAGAAAAATCTAAATTGCTTAA

**Linkage Group 12 Scaffold 210**
>EACMGC336G_247833
GAATTCACTTGCCCGACTTGCACTGCAGCAGCAAGAGGAGCAGAAGAAGGACGAGGAGGAGGAGGAGTTGG
AGGAGATGGAGGATCGCCGTTCCTCTTCCTTCGTTCCTTCCTTCATTCCTCCCTCCCCCCGATGAGGCTCA
GCCAGCTTTTGGGTTGCCCTGGTTCTTGCCAACACAGACTGACAGGCGGGCATTGTTTATTCCCCCTTCCA
TGCCCTTTCCGGACTCAATCCCAACATTCCATGTGTGCACACGCTTATGCTTGCATTTTTATTACTGAGTC
CCAAAATACACCTTTCTTTCGAAGGCTTAA
>EACMGC258G_626625
GAATTCACCATAAGGATCATCAATAACGCCCTCATAAACCCACCTATCATGCACCAAAAACATGACGTAAG
AAAATCAATACTCAGACATAGGAAGCAAGCACATTAGTTTTTTTGTTTACATGACCAGATGGAACACAAAA

AAATCCTTTTCTGACTCATGCCTACATAAGCTGTTGTAACATAACCCAGCAGAAAGTCCTAGTAAGAAAAT
CTACCTTGCTAATTGCTTCGCTTAA
>ECAMCT297G_410124
GAATTCCAACAATTTGCTGTTAGGTACTTTCCTCCGCAAAATGTACATTGATCCATTATTTGTGAGGGTGA
GGGGCATTCTGTTACAGAATGACCATGGCCCTTACAATTACTGCACCATACATTGGTCTTAGTATTGGTAG
GCTTTTGCATCTTTTCCTTATCTTTGACCATATTGACAGCTATTTCCTCCATTTTCTTCGTCAAATTCAAT
AACAAGGTTTCATTGGAATCTTTTAGGGCTGGCATTATTGGTATCTTTGCCATAGTTAA
>EAGTAA69G_513938
TCGAAAAAAAACAAAGGACGCTCTTCCGTGACCAATGCATGGCTGAATTC

**Linkage Group 13 Scaffold 44**
>EAGMCA392G_480651
GAATTCAGAACGGGCAACACATTTGGAGCCACTGCGTTTACCTCATACGGAGCCTTCTGGCTTTCCTACGC
TGCGATTCTCATCCCCTGGTTCGGCGTGCACGACTCTTACCTAGGCGGCTACGAGCGCGACGTGGCGCCCG
CGATCGCCATCTTTTTACTCGGTTGGACGATCTTCACTTTCATGATGTGGTTTGGGACGTTGCGGGCCAAC
GTCGTCATCAGCGCCTTGTTCGCTTTTTTGACAATCACTTTTCTACTGCTCACCATCGCCGAATACAAAGG
CGCAGAAGGTCATCGCATCAAGCGGGTGAGCACCAAATCTCTCTCTCTCTCACACATACACCTCTTTTCAG
CCCATATTTGTTAA
>EACMGA251G_605264
TTAAGAAAGAAGGATGGAGGCATATTAATGCTGCAGCATGTAAAACTGCATGACTCCACATACATGTAGGT
AAATTTGTATGAAGGAGGAGTGGTTGGATAATGAATTGTATATGCTTTATAAATGACTCTACAAGTCCATT
TTGAGTATCTTCATATGGAAAACAATATGTCAACTCAATTCCTATTGTAGTACAATAGTCTTCAAAAGACT
TTGTTGTGAATTC
>EAGMCT367G_734554
GAATTCAGAAAAAAATCCAAAAGATAATATATTGGTGTATTATAAAAATTAAAAGACAAATTAAAAATAAA
AGATGTAGATATAAAATAAAGATAAAATAAAGTGGCTATTGCCAAATACATGAAACTAAAGAGACTTGAAT
TAAAAGAAGAATATTAAAAAGCCATTGATAAGACTAAAGATAGCTTCATTTTCACTTTATATACTACATAC
TAAAAAGATATTTTAGAAGCTTTCTAAGAACCATGGGCTTTGTGGCCAAAGGTCAATTTATAGTTTATTTT
GAGTGTTACTTTCTAAAAGTCAAAAACTATCAAGGCCATGAATCACTAAGAACCAAAGAGTTAA

**Linkage Group 13 Scaffold 400**
>EACMGA251G_101826
TTAAGAACCGGACGTAGAATCAATATTTCCGGTCATTCAGAATGGATCCCTTTCATACTTCCAACCCAAGT
ACTTCACAAATGGACTGGAGTAGATGTGCATTCAACCTCATGGCCAAGCGTTGTTTCACACACGGGTTTTC
ATGCACTATATATCGGAGAATCCGTGTCATAAAACTAGTTGTGATCACGTTAGGACATCGTTGACACTCAA
ACCACATGTGAATTC
>EACTAG175G_174327
TCGAAGAAGAATAAGACTCCAATATTGTAACAAAAGAAGTATCAGTTTAGAGGAGGGTAATCCGCAACCTC
TTAAATGCTAGTGCACCTTCAAGCAGAGGGGTAATCCACACTCTAACGAGGAAGAAACTAGCTCTGATATC
TGAGGTGAATTC

**Linkage Group 14 Scaffold 31**
>EACTAG360G_843454
GAATTCACAACACTGACTGAACAACCTCTCAAAAGAAAAATCAGAAGTAAAACAAAACAAAACAAAACAAA
CAAGACGCCTACACGACAAAACCCCTAGCGAACTCACGCCCACGCAAACTCACGCCCACATGGCACCGCAC
AAGACCGACTCAAATCCAAACTCCGCCAACCTGAGCGAGTACACAGCGACCCAGCGTCTCTCTCTCTCTCC
CACCCAAACCTTCCAAATAGATCCAGGACATCAACGAAGCAGTCACCTGGAATTAGTAGCTTGCCTCTAGT
TGATCGGTGCTAGTAGCAGTTCCTTCTCATCCTTCCAAAACACCCTCAACACTTCGA

**Linkage Group 14 Scaffold 39**
>EGAMGC549G_925823
GAATTCGAGCCTGCTTACGATGGTCAACCAAAAGGCGTGTTTGTAGGACAATTTCACAATGAAGACACAGA
AATTCATGCTTGGTTAGAAGTCGAAGGCGTGGGCCACTACCGTTGGCTTCAAGGGCCAATGAACCACTTGC
CTTCTGAACAATAGTTGGAGTTCATAGTAGACGTTATATGATAGATCTTCATTTTCTTATGTTGGTACTGA

GTAACAACAACATTACTTTTCAGAGATCTCGAAGCTAAAGGAGGGGGTGTATTAGGGACATAGTCCTTAGA
ATCTTCTAGAGACAAGTTTCTAGAACCTTCTAGAAGCAAGTCTCTGGAACCTTCTAGAAGCTTCTTAGTTG
ATAGAAATTACTTAGGAAGCTTCTTAGTTGGTAGAAGATGTTAGGGACATAGTCCTTAGAACCTTCTAGAG
ACAAGTTTCTAGAACCTTCTAGAGACAAGTTTCTAGAACCTTCTAGAAGCAAGTCTCTAGAATCTTTTAGA
AGCTTCCTAGTTGGTAGAAGTTGCTTAA
>EACMGA402G_907507
TTAAGAGTGCAAATTAGGAATAATTACCTCAAGAAGACGAAAGTTGATACCTGGAAAGGACCGGTTATGAA
CCAGGGTCGAAATCAGACCCTGAGGCACAAAACCGAGTCTCGATGACGACTCCGGGATTCTTTGGCAGATC
GGAGAAAAAAGTTTGATAGAAGATTGAGTAGGGTTTCTATAGGTGTTGATTTTGGGTGATTTGGATTTGTA
TTGACTTTGAAATAGCTTTGCAAAGTAGAGGTTTGTTTTGGATGACTTGGGTTTTATGAAATAAAATAGGG
TTTATGTTTTGAGTGATTTTGAGAGTTTTGTTGCAGATCTACGGGTGAATTTTTTTTGTTTTATGATTATA
GTTTTTTGCAATGCATATTGTGAATTC
>ECGTGA362G_1792070
TCGAGACAGATGATACTGACATCAATTCGTTTTTTGTTTTTATCACAACCCTTATTTTTACAATAATAATA
ATAAAAAAAATTGTAAATCAAAATGTTTTTTTAATTATTTTATTGTTGTCAAGCAAAAGAAGAATAAAAAT
TGAAGTATTTCATGAGCACCTAGAATTAGAATTACAAATTCTAATTTTCATGTCCAGATAGAATCTCCATT
ACGGTCAGAGATTCATTTCGCCGACGTGGCATGACACGTGGCTGCCCAGAAACATAGAAAACTTGTCACAC
CAAAACTCACCGGTCGGAAGTATACGTATATGAGATGAAAAAAAATACGGAATTC
>EAGMTT579G_1944165
TTAATTCTTTGATAATCCTTCCCTCCATGACTATTATTCATAATCTTCCATTCTTCTTTACAAAATCAATA
CTGTCTTGAATTATAACTTACATATTTGTGATCTCAAAACTAGTTCTTTTCACGTTCTCATTCAACATATA
ATTCCATCTCCCATACAATCAATTTCTAACATTTCATATTGTTATTATTTCCTCAGCAGCGACGCCAAAAT
GTTATTTTCATTGTATTCAAGCTTCAATATACCAAAGCAATTGAACTAATAGAGGTGGATTCGAACACGGA
GAAAGATTGGTTGTATAAATTGGGTTTGTTAGTATCACAATTGACTATATTCTGGCTATCTAAAATCAAGT
ATCAAACTATAAAACACCGGGGCTTACAACTTCCATCTTAATTCAACCCATTATCCATTCTACGATTATCA
ATTCAACAATACTTCTTTATTCTTATTCGTTCATCCTTCACTTACTCTCTTTGTGGTTTATATATATTCAA
TTACTGATCTTCCGCTTACTTATTATATTCAATTTCTCAATTTACACCCTGAATTC
>ECGMCA271G_284731
TTAACAAAGAAGCACCTGAAGAAGCATAACGTGAGAGATTGGCTTCGCGTGATAGCGTCCAACAAGGACAG
AAATGTGTATGAATTGCGGTACTTCAACATCGCGGACCAGGATGCCGAGGAGGAGGAGTGAGGCCCAATTT
TTCGAGTCCTTCGATATCAAGAGTGCGACACTGAGTTTCTGTGGCCATTTCTTTTACGTTATCCACAAGGA
TCCTTGACTTCGAGTTTATTTTTTCAGCTGTGCGGAATTC

**Linkage Group 14 Scaffold 138**
>ECGMTA118G_514863
TTAATATACCCGAAGGATTCGGAAAACTCTGCTTATGTTAGTATTGACTGGAAATCTCCGCACGATGTGCA
CCATGTTAGTGCCCTCGAGACGGAATTC
>EACMGA402G_196702
GAATTCACATATTTTCTAGTCATGGATTCAATTACATTTTAATATATTTTTGGAATGGCAATTCATGCTAC
TATTGAGATGTGTCTCATAGATGTGATAAGTATATATTTATATGATTCTCTTGATGTAGATATTTATATCA
AAGTTCCACCTAGTTTGGAAATAAAAACTCCATATGCACCCACTCCAAGTTAATATCATGGTATTAAATTA
CACAAGGCCCTATATGATCTAAAACAATCTAAACATATGTGGTATCAACATCTCTAAGATTATTTAGTTAC
AAATAAGTTTATAAGTGATCCACTAATTCCTTATTTCTTCATCAAATGAAATAAAATTGATTTTGTGATCA
TCATTATAGATGTTGATGATCTTAA

**Linkage Group 14 Scaffold 364**
>EAGMCT163G_148642
GAATTCAGAAACGAAGCCATCGAGTAGTTGTAAAATGCTGATAATTAACGTTAGCCGACATGACACCCACC
CCACAGAATTATCCTCCGCGAAATTGATCGTTCGGCGGTAGCAATGACCTAGGATATATAATGCTAGTTAA

**Linkage Group 15 Scaffold 24**
>ECAMCC379G_1874548
TTAACCAGCGGCAATTCATCACGAAGCGGCAACTGAAGGGATTGGAAGATTGCACTGACATTTAGAATGAA
TGGCATCAATGATGTGCCACGTAGGATCGGAATAATTGGTATGGAATCCAGTTCTGACGAGAGAAAGGTTC
ATTGGCGGAGAAGTTTGATTGGATGTTTGTGTTTTATTTGACGTTGCCTGGCTCAATAATAAGTTCAGGGC
CCCCTTTTTGTTCCTCCACTGCCAATCAACCACATTGTATGTTGCTTCCAGCTGGCGTGGCTTCCCTTGCA

CTGAAGCATTTCCAGATTGTCAGCAGAGCATGCGTTCGTTCATTCCTTTCCTTTCTCAGCATTGCTGGAAT
TC
>EACMCC373G647226
GAATTCACCATAGTGAAGGAGTTGCAGAGCCCTTGGAAATCCTCTAAGGAGATGAAGTTCAACCTTATATC
AAACCCATGAGATGAGAACTCCAACCAAGTTCTATTGACACAATGCTTGTTTTGTGAGCAGCTTGGTTGTA
AAGAGGTCAATCTCACAAGGCATAAGCAAAGTAGATGGTGAATGTGAAAACATTTTCAATTTTTTTTGTGT
AAACATTATTATAGTGCATATGAAGTTATAGCATACCAAAGCATGGATAGAATATAATGGCCTTCCAAATG
CCTCAAAGAAACAATTCTTTGATCAAGTGAATTTCAAAAACATCCTTATAATTCATTTTGAGGTTAA
>EAGTAA136G_674845
TCGAAATGTGACTGCAGGTCTATGAGTAGGAAAAAAATAGCAAGCCTCCAGCCTTGCACATGCTATGTCAT
GAAGGTCCGACAGTGCTATAATTAAACAATGAATCCTGAATTC
>EAGMCT332G_522281
GAATTCAGCATTCTGTCCAAACAGATTAGAATGATTATCGCAGACAACAAGACACCTAAGATGCATATGCG
AAGGTCAGGTTGTAAACTCATCAGGGATGTGTACATCTCAGCTCCAACCTACTACCGTGGTTGCAGAATCT
CACATGTCCACAACAAACTGTCGACTAACTCACCACGTGCCTTGCAGCGGCCCGAGGATAAGAAAATATAC
CACTGTAGCTGAGTTTTCAACGACTAAATAAGTTTACCTAAAACTGGACCTAAGGATCGAGCTCTATCCCC
ACTTCCGTATGGACCATGCCCAGTTAA
>ECGMCC307G_1344766
GAATTCCGACTAAGCATAAACTTCCATCTCTTCATTGTGAAAATGCCTTGCAAACACTCCTTCGGGAAGAT
CATTGTAGGCAGGTTCTACCTCAAACACAATCACTAGCGACTTATTACTAGGTATTCTCCGTCGTCTTTGT
GGTACAGGCGGTTTTTCATTGGGGTCGTTTTCGTGAGATACTCGGTTGAGAAGACATGACTGGTATGGTAT
CTATTTCTTGGCTATCTATATCAAACCAAAAAATATAAGCACTAAATATGAAAGAATGGACTCAGGTTAA
>EACTGA359G_1252649
TCGAGATTCTAAGATTCTTCTAATCCACAACAAAATAAAATTTCATCACAAAGTAGAATAAAATAGACCAT
AACAAAACTAAATAGAACTAAACAGAATATGACACAAAAGATAAAGTTATGCAAATGAAAATAGAACAAAA
CAAAATTGAATAAGAAGATACAAAACCAAACTTTATTAAGAGAGAATATTCAAAGTCATATTTGACCAGAT
GCCTTGGCTAGTCTTATTTCTACTTTGTTTTATCACCATGGACTTTTTAGAATGTTCTTAGATGATGGAGT
CCATTTAAATAACCACCCTAAATTCTTTTTGGATGTTTTTTGGTGAATTC
>ECGMGA409G_202263
GAATTCCGGCTATTAGCTGGTGGTCCAATCTCAACCTGAGATTGCTAGGTTTGCGATTCGATTGTTAGGGG
TTTGCATTGGAAACTGCAAAGAATTAGAGACCCTCAGTGTCGTTTCATGAGGCATGTGCTTTCTCGAGTGG
GAAAGATTGATTGAAGCAAGTGAATCGATGTTTTTCTGATACTATGTGACAGAGAAACTCTGAACAGACTT
CTTATTGCTTAGTCTTGAGCGGATGTATGACTTGGAACTCCCGCGTTCGATTGCTTTCTTGAAAGCAATTT
GTGCCGAGCCCTGAACACTCTCTGGATGGTTGAATTTTTGTCTCGTCAATTTCCTGGATAAGTCCGAAACA
CCTCCCTGAGACTCAGCTACGTTCTTAA
>EAGTCA408bG_321758
TCGACAATTTTGAACAACTTCAAAATTTTTTTAGAGGCGTTTGTAGAAGCATTTGGCGAACATGATAAGGC
GTGATGGGCTACAACAAAGATTTGATCACTACAACAAGGGACATGCTCTATGTTAATCTATGCATCAGATT
TTAGACAAATAGCATGCAACATCAATTGGAACGAGGAAGTATTGATGAGTCAATTTTATTGGAACTTGAAG
GACGACGTAAAGGATTTGTTATTATCTCTTTTCAATCTGCAAATGCTCAATGAAGCCATTAGCCAAGCTAT
GAAGTGCCATAAACACTTGTTCCAACATCGCCAAGACCAATACTCAAGACAACATATGGCTCACTACGCTC
TAACTATGTCAGCAAACAACCTGAATTC
>EAGTCA187G_2200202
TCGACATCCCAGTTAGCACGAAGAAGGTGGTGCAAGACCTCAAGGAGGTGGTCGGGAACAGCGAGGAGGAG
ATTTATGCCATGCTCAAAGAGTGTAACATGGACCCAAACGAAACCGTGCAACGGCTGTTGAGTCAAGGTAC
GCTCCGAATGCGCGCCTGAATTC

**Linkage Group 15 Scaffold 29**
>ECGMGG495G_2478243
GAATTCCGCCTCATCACCTTGATCACCAATTACTCCGAACTGAAACAGCTGGCTTGCTTCAGTAACAGATC
ATTTGTAGCACGAGAAACAACCCAAAACTGCAGTTATCACATGAACAAGACTCCTGCACATTCAGAGTCTA
GGTCACCCTCGTTTCATCCAAATAGCCGAGAAGAATCGGGTGCAGATTTTACAACGAATATATCAGCTGTT
GTTACAAGTCCACTTCATTCAGCACGCTACTACCACAGAATCCAGCATGTGATAGAAACGTCAACATTCCG
ACTAGGAAGATTGTCTGGCTCGGAACAAGGAAGAACAAGCACACTGGTGCAAAACACAAGTGGTCGAGGCA
TGTAACGCCGAACTCAATCAAGTTCGCAGTATCAACCATCGCCTGAGGGCCCTCAGGCGACCAAGTCGACG
GAGTAGGGCGAGCCACTGTTGCTTTCGTCAGGATTCCCTCGTCCTTAA
>EACTGA329G_2205820

TCGAGATCAAAAACTCCTCATCATCAACCTTACAGACTTCTTCAGCCATTATCCATTCCGAAACAACCATG
GTCTTCTATTTCTTTGGATTTCATCATGAATCTTCCAATCTCACATTCTTATGACTCTGTATTCGTGATAG
TAGATCATTTTATCAAGATGGCACATTTTGTACCATGTACAAAATCTATCACAGTTGAAGAAATAGCTAAG
CTCTTTCTTTACAATATCTATTGCTATCATGGATTATCCAAGGATATTATATCACATAGGGGAATATAATT
TGTATTAAGATTTTGGTGAATTC
>EGAMGC135G_1840739
TTAAGCTAGGTACGTGAAGTTTTATTGATTACTAGGATTTTGAACTTACTGCTTCACGCCTGTGTTGTTTA
CTACGGAAGTTGCAGAAATCTTTCCCCCCAACATTATCGAATTC
>EACMTC359G_461004
TTAATCTAACAAGACAGTCAGTGGATACAAGATCGTAAGTACAAAGCGGTATCGGAGATTAGCAGATTGGA
TTGTTCGGGGTTCTTTGTAACGTCTAGAGACCTACCGGCTGGGACAAAAAGGACTTCGACAAGTAGTCGAA
GGGCATCCGTATAGATTTACGATGTATTCTTGCAGTCGTTTATATTCCAGCAGTCAAAAATTGATAGACTG
CTTGACTATTGGCCAATCTCTATACTACATTCTACAGCCAGACGCTCGGCGCGTTCTCGAGCAGGAAAGCA
TGTAATACGACACCGCATCAATTTGCATGTCTTCTACCTCTGAATGTGAATTC
>ECAMGG184G_431967
TTAAGGCATTGTTGATACAGACAGAGTGACATAGAAAAAACAAATCCATGAGACGAAATACCAATTGCGAA
GGCATCCGGACACTATTGCAGGGTGTAGTGATAGAGAACGACCCAACAAAGGTGGTGCCGCGATTCTGCGC
CATGGATGTATTGGAATTC
>EACMGC315G_1840739
TTAAGCAGTGTGCCAATGATGACAAGTTCAAGGTGGAGGGAGATCTTTTATACTTTGAGGAGCGGTTGTAT
ATCCCAAAAGGACCAGCACAACTCTGAGTTCTTCAATCTCGCCACGATTTTCCAACTGCGAGATATTTTGG
ATTCAACAAGACCTTGGAATTTATTTCTTGAGATTTTTGGTGGCCCCAAATGTGGAAGGATGTGAAAGATT
TTGTCATCTCCTGTGACATCTGTTCAAGATTCAAGAATCCTCCGCATCGCCCTTATAGACTCCTATAGCCA
TTGTGAATTC
>ECAMCC477G_511611
GAATTCCATCTTATACCTGAATTCTCTAAGTAATAATATCCATTGAGTTATATGACCATTCAAATCTGCCT
TGTTGACTAAGTATTTGATGGCCATATAATCAACAAAAAAAATACCACCAAATTTAATAAAAGTTAATGTT
AGAATTTCTTCATAGAAAAGACTATGGCTAAGTTTTTTTTTTTTGTGGTATTATAATTTTATTTTGCTATA
TTTAACTTTTTGCTAGAAAATATAATAGGATAATCTTCATGCCCACCTTTCATTAGTTGACATAATATACT
ACCTATTACTATGGATGAAGCATCACAATAGATATAGAAGGATAGATCTCAATTAGGCAATTTTAAAATAA
AGTAGGAACTTAATGTTGTTTTTATCCAATCAAAGGTTTCTTGACAAGAGAATGAATCTCTTGTCTATTTA
ATCTTAACCAACTTCTTTTTGAGGTTAA

**Linkage Group 15 Scaffold 220**
>ECGMGT156G_424629
GAATTCCGGCATTGATCATATCCTCGGCAATTTGTTGAGAAAGCTCCCATTGCTGCCCGCGCCGTAGCACC
TTCAGCATCACATTATAGGTGTAGATGTTGAGATTGAAGTTCTGCTCGGCTTGCAACCACTTAA

**Linkage Group 16 Scaffold 14**
>EACMTC333G_726067
GAATTCACAACTTGTATCATATTACGTGCTTTCCTGTTTAAGAACGTGTCGGGCATCTAGCTGTAGAAGGT
AGTATGGAGATTCGCTAATATTTAAGCAGTCTATCGGTTTTTGACTGTTGGAATAAAAACAATTACAAGAA
TATACTATAAATCTATACGGATGTCCTTCGACTGCTTGTTGAAGTCCTTTTTGCCCCAACCGGTAGGTCTC
TAAACATTACAGAGAACTCAAAACAATCCAATCTGTTAATCTCCGATACCACTTTATACTCATGACCTTGT
ATCCACTGACTGTCTTGTTAGATTAA

**Linkage Group 16 Scaffold 177**
>EAGMCA196G_885714
TTAACAGATGCTAGTCATGAGCTGCATAATTCCTCAGTTGTATGTACAACTTTAGGTTCCTATCAAATATA
TAAATTCTGTTAGATTCACAACCCTTCAATCTAAACTAACGCTTTCCGAACTGCAACTCAGAAGCCACAAC
CAGAGCTTGAAAGCTTTGGAAACCTGAATTC
>ECAMCC111G_551662
GAATTCCAAGAGACAACCAACAATTATAAGGTCAACAATGGAGGCGGAGTATATGGCAGCAAGCCATGGTG
CAATGGAGGCAATATGGTTAA
>ECGMGT119G_601535

GAATTCCGAAGCCATCAAACCTGACTCACTCAACAAGCTCCAAACCCATATGGAAATGGGAACATGCATGA
AGAAGGGAAACAGAGAGATTGCCACTTAA
>EACMGG215G_642662
GAATTCACTTGCTTTTATATCAAGAAGTCCCTTTCTTTAAATATTTGTAAAAAAAAAAAAAAATAAAAAAAA
AAATACAAGAGAGCCAAAATTCTCCTAAAGAATCTTTATATCCCCTTCCTAAGATGGTTGCTCTTCTTTTC
TCTAAAATTTTCCACCATAAATCCTACCTCTAAATCTTAAATCCTAAACCTTAA

**Linkage Group 16 Scaffold 195**
>EAGMCT95G_846495
TTAACTTTACAAAAAGAATCACAATCAGCCACTATATATGCTACTGAATTC
>EAGMCA196G_817844
TTAACAGTATTCTGAACTACTAAAATATTAAAAAATACTACAATGCTTCTTCCCAATAACAATACTCCTTC
ATCAATTGATAACCACAATGTGCAACCCTGCACTCTCAAAAGACCACTCAAAATTCATCCAACACTAAGAA
TAACAAACTTCACTCCAAGCAATCTGAATTC

**Linkage Group 17 Scaffold 167**
>EACMGA167G_508093
TTAAGATAAGTCAATAGAAAATTTTAGAGGATAACACTATTTATTCTATATAGAAAAAGTGCACTCCATC
TTATTTTTTCTACTTAAATCCTTTGCATGTACTTTTAAAATTTTAAAGTCAACTTAAAAAGACCTTTGTGA
ATTC

**Linkage Group 18 Scaffold 116**
>EACMGG132G_1132736
TTAAGGATCAAGTTTTGCTGCCTCTGCAATGTGCTCCACTAGCAGATAGTACATGCACGCGGATATATACT
CTTTGAGAAAATTTCTACAGGGCATCATACATGTGAATTC
>ECGMTA237G_1177113
GAATTCCGATCTGAGAACCGTACAAGTGCACCTCAACGTTGTTATAATTCAATGGCAACACCTACACCTCT
TCAACACATACATTCAAAAGTAGAGGATATGCAAATTGATGCAGTACGTTTCAAACCTCTTATTGAGCAAG
AAAAGACTCGTCGTCGTCAAGAAGGATTATGTTTATATTGTGGAGAACCAAAGCACACTACTCAATATTAT
TAA
>EACMGA230G_91605
GAATTCACTTAGTAATTTTTATATTGAATCTACTATTCTAGAGTGGAATACTATATATTAAATTAATACTA
TATTATAAGATAAGAATATAAAAGACAAGATTAATTGTAGTAGTTAATTTTCTAATTTGACTGAACCATGG
TAGGGATTGGTTCAAGCCTTGTTGAAATCTAAGATATCTAAGTTTATAAAAAAGTCTCTTAA
>EACMTC144G_1109153
GAATTCACTAATTGTGAAAAAATAACTCTTAAAACATTTAGATGTTGACTCCTTTTCTTCTTGTCGTAAAT
ATATATCTACTATAATCATTACCTAGGTAAGTAGAATTAGGTAAAAGAAGATTAA

**Linkage Group 18 Scaffold 169**
>EAGTCA519G_154496
TCGACAACACATCTCTTGGAGTTCTAGCTCACCTTTCTGGTGTTGTTCTTGTATTCATCTGTTTAATCTAG
TTCTGTCACTTCTTCTTAAACTTCTTGTTAGATTTCAAGTCTGTATTTTGTGTCCGTGATAGCATCACACA
ATGACAATGGATCAGCAATAAGAGCAACGTTTGCATATTCTTCACTTACTTGAGGGAAGATGTGATCCTTC
CACCATTCACCATGTTGCCTTCTTTCTTTCAAGGGATATCTCCTTTCACTTATAAAAGAACATTCACCTTG
TTGCACTTTATCAATCTTCTTCTCTCTTAAACCTTGTCGTTTTTCACCCACTGGTTGGTACTCTTCATCGT
TATCATCATCCCAATCTTCAACATCTTCATTTCTTTCATTGTTGGTCCTTTCAATTCTAGGAGCCTTCATC
TTTTCAACCTTCTTTGGCTTTTCATCTTCATCATCTTCATTTTCTTCATCTTCTTGTGTCTTTGCTGAATT
C
>EACMGA230G_333272
GAATTCACATCAAGAGATAAACATCTTAAATTAAGTCGATTGAAAAGCAGTACTTCACTGTATGGCTTGCA
TAAATCTATTATCAATAACAAACACAATAAAGAATTGCAAATACAAAGTGACAGTCATTTGAACTATTGTC
TTCAATGTGGCAACCCTGGACATACTATTGATAAATGCAACCAAATTCGTGAATATTGCAAGTGTCTTAA
>EACMTC144G_568005

GAATTCACAAATTTCGAGATTGCAATATTTTATGTGATGTATTTGGCGATGCTCGTTTGGATGAGAAAGTC
CTTCTTTTGCCTTGATTGGTAAAATCTTTGCTTTTTTGGGGGGGATTAA
>EACMTC138G_568005_Variation
GAATTCACAAATTTCGAGATTGCAATATTTTATGTGATGTATTTGGCGATGCTCGTTTGGATGAGAAAGTC
CTTCTTTTGCCTTGATTGGTAAAATCTTTGCTTTTTTGGGGGGGATTAA

**Linkage Group 19 Scaffold 69**
>ECAMCT199G_1511401
TTAACTACAAAATAAAGAGGTAGATTGTAGGCTTCAAATCCAAATTCTAATTTAGCACGTCTTTATTTGAC
AAAGAAGGAATGCATGAAAAATTTACAAAAATGGTAGAATTTGTTGGTTATTTACTCAACCTTTTGTAATA
ACTTTGATACAAAAACTTCTTGTTCCTTGGAATTC
>EACTCC399G_976642
GAATTCACATTCAGAGGTGGAAGACATGCAAATTGATGCAATACGTGTCAAATCCATAATTCTAGAAAAAA
AAAAGCGGCTTATGGAGGAAGGACTATGCTTGTATTGTGGTGAAGAAGGCCACAGAGCAAGGAATTGCCCC
AAGAAGCAAAATTGACGCATTGTCAAAACCAGAGGCATATTGTCATGTTGTAACATGACTACATGGTGAAA
GATGATAATATGGATCTTATTATAGAAGAGCCAAGTTAAAAAAAAGGTAGAAAAATATGGATAAAAGATAG
GAAGATAGTAAAACTTTTAGTAGTAATGTTGCCCTTTTAGAAAAAATAGCTTCTACTTACTTTGTAGGACC
AAAAATAAAGCAAAGTAAGGTCGA
>EACMTC377G_1664097
TTAATCAAAATTTAACCAAATCCAAAAGAAGTTTGAAAGAAATTATGAAAGAAATATAATAAATCATAAAT
TAATCTTAAAAAAAAATTCTAAAGAAATACTAAAAACAAAAAAACCTTAAAGAAATAATAAAGAATATTTT
TAAAAAACTAAAAATAGAAATAATAAAGAAATAGAAATTCTAAAAAAATACTAAAAATATTAAAAAATAAT
AAAGAATCTTTAAAAAATAATAAAGAATCTTGAAAAAATAATAAAAATAGAAACCTTAAATTAATCCTAAA
TAATTCCTAATCATAATTTAATGCAGTCCTATAAAAATCCTAAAAAAATCATAAATAATCCATGTGAATTC

**Linkage Group 20 Scaffold 188**
>EAGMTT151G_50698
TTAATTTGGAAAAAATCTAATTCAACAAACATGGAGAGTGACATTCACATTCAAGGAATACCTGAATTGTA
ACTCATGTAAAAGTAGATGAATACAAACACTACATGACACCTCGTGTACTCTGAATTC
>EACTAG272G_14283
TCGAAGAGTTAGCTTCAAGCTCTTTTTAAGGTTACTAGTTCGGTTTCATTGGAGTTTTTTGAAGATCAAGA
GGATTGGAGAGGAGCTTCATGCTTCGGGTCGAGGAAGAAAAGATGGAGAATTGGATTTTTCTTCTTCATAG
AATTTTGGAAAGACTAAGGCAATCGTTGCTAAAGTCAGTGTTGTAATAGTGAATCGGGATTTTTTAATGAA
GTAGGTTTGATTACTCTTGACTGTTTTGTGAATTC

**Linkage Group 20 Scaffold 273**
>EAGMGG217bG_330223
TTAAGGTCACACAATTAACCGTATTTCAAACCACTAAAATACCAAGAAACACTATGATGCTTTCTCCCAAT
AAGAATACTCCTTCATAAATTGATAACAATAATGTGCAACTCTACACTCTCAAAATACTGCTCAAAATACA
ATAATTTCCATCCAATACCAAAAATAATACACTTCACTCTAACAATCTGAATTC

**Linkage Group 21 Scaffold 149**
>ECGTGA127G_172881
GAATTCCGTATTGTCTCCTCGTTTTGACTGTCGTGGTGACGTTTGGGTGTTAAAAAGTTCAACAAGGCGCA
CTCTTTGTTCCACGATAATGTGCGCAATTCTCGA
>ECAMGG56G_347558
TTAAGGGGGTGGCCAAATTGAGAGACTCAATGGAATTC

**Linkage Group 21 Scaffold 159**
>ECGMGA123G_142075
GAATTCCGAACCACTAAAACACCAAGAAATACTACAATGCTTCCTCCCAGTAACAATACTCCTTCATCAAC
TGATAACAACAGTGTGCAAGCTTGTACTCTTAA
>EACMCC558G_429888

TTAACCACCTGTGCTAACCTCAGCACAACTCAGTGCTCCGGTCAGCTCCACAACAGAGCCAAATACAATAA
TGCATTATCTCATGGGCAGGAGAAGACCCAAATACCATCCTCTCGACAGATGAGCGAGATCCACGAGAACC
CGCAGCCAACACAGGATTTCGGTGAGGCACCTGCTTTCAGCCTTTATGGAGGACTCCGCGCAACAATGCGA
ACAAGCAAACCCTAATTCCTGCACGAAGGATGATACCCGAGGTAGATACCATGGTAAAAAAAACACAGAAT
GCGATAATTTGGTAATGAAGTCCTGGATATACCCGCCATGGTATCTGCTTGGCTTGAAAAGACAGCTCGCT
GCTGAATCCTCCGCCCACTGTTTGGTTGTGTTTTGTGTGGGATGTGAGGGGCATGGTGATGTCGATTTGTG
CCTCTGAATTGGCTTTCTGCTGTTCGAAGTGCCCGGTGGCCATGACGTCTGATAGCGTGCTTGCTCCTGGA
CAGCCACGCATCAAATCACAAGTCCAACGGTGAATTC

**Linkage Group 21 Scaffold 217**
>EAGMTA375G_455640
TTAATAATTTTTTAAAGTAATCTTTTTTCCTGGTTTCTTTGATTAATTCATCTATATCAATTTTGAATATA
TTAATAAAAATATCTAGGATCAAGAATTGCTCCAAAAATACCTATATATATATATATATATATGTTATCAT
ATATTTATGTATATTTATGTATATTTTTTCATAAGTATTGTCACTATAAAAAAATGAATGACAAATCTTGA
TCCTTGATGTTCTCTTCAATGCACAACATCTACAAAAAAAAACTAACAAATCAAAAAAAAATCAAAAACCA
TGTAGCAATTTATGGACCAATCAAAATAATTACGTACCATCACCAAAAAAAGTCGTTCAATTAACTGAATT
C

**Linkage Group 21 Scaffold 256**
>ECGTGT221G_208374
TCGAGTAAAAGAAGACTAGAGGAGAGCTGAATGTTCTGGTTACAGGCTTCCTGCTTTTTCCTGACCAAGGA
CGATGAGTGGCAAACATTCATTGCCAGTAGCCACAAGAGTCTCCAGAAGAAATATTCTTACAGACAGAAAC
TTTGTTAAGGGGTTAATCGCCACTCTAATGTTAGAAAACTTTCAGCATCGGAATTC

**Linkage Group 22 Scaffold 2**
>ECGMGA373G_1033182
GAATTCCGGCAAATGGAGGAGAACCCTGAGAAGATTCGGGAGAGGTGCGTGTTGCAGATGGTAGCAAATCT
CGGGTGCCAGTTCGCAAAGCCTGCACTCTCCCCTCCATTCGGTCAGCTTTTTTGTGCTCATCTCCTCCCTT
CTTTCACTATCAGGGATGGTATCTAATTAAGCACCTCGTGCTTTGTATTCTGACGCCCTGCAAGTTGCATT
TCCCTGCCTTCGAGGTCCACAATCATCGTCTTCAAACTGGGTTCACTAGTTCACCTCCTCGTCGCCCAGAA
CGCGTCATCACGACATTCCCCAGGCACCCATCACCCCTGCCACTCCTATAACCTCCCCCTTCTTAA
>ECGMTA445G_3764784
GAATTCCGTAAGTTTGGTAGTAGATCTACAAATAAAATTTTAATATTTTGTGGGTTTATATGTATTTAATA
TATGTTGTAAGTTTGTGTTTGAACGAAGGGTTGAATTGAGGATTATTGAGGTTTAAAATGGTCAAAGTATT
GGAAAGTAGAGTTTTAGGTGTAGTAAGGGTTTCTAAGCTTTCTTGCAATCTACTAATTGGATTGAAGTGGA
AATTAAGTGTGTGGGTAGTAGACTTGTTTTTCTACTTATACAAGAAATTTGAGATTAATTGAATGGTTTGA
TAAGGAGATATTGGCACTGGAAATTTAACAATTAGATTGAAGAAGTAGGCCGGCCAACATCCAAGCAAATA
TTAGAGTTGATCTTCTTTTTTTGTGAATTCAATCTTCAAAAATTTTGTACTTATTGCCAACTTAATTATTAA
>EAGMCA493G_1648617
TTAACATTGTGGATGGAGAACTAGCCATTTGTCTCGAAACTGCAGCCGCAGCTGCTAAGGCTGCTCCTGGT
CTCGACATTATTCGAACTCCTGCAACTTGACCTTCGGACTCCAACACCAGTTTTGGTGACCTAGACATTTC
GTCACTGTCTAAAAGCACATGTCCTCTTTCAACAGCGACAGACTCCAAACTTGAGTAATCCTTCGATCGGG
TCTCCACGAAGTTAGCACTGCACGAATCCTCTACAAGACTAGACATCTCAAGGGAATGCAAATGCGGCGAT
GCATCCAGTCCAGAAATGCCTCCATTTGTAACACTTTCATGTCCAAAAGAAGCTTGACTCCCAAGCAATGA
GGCATTTAAGCCCTCAAAAGACGCTAAGATTGTCGAAAATGGAACATCATTATTAAGATCCGTTGAATCGA
TTATTTGATCAAGAATATTGCGTTCAAGCTCGGATATATCTGAATTC

**Linkage Group 23 Scaffold 102**
>ECGTGT487G_352942
GAATTCCGATAACAATCCGTAATCAATGTAATATGGTCTCTTATCCCCGATATCATGGCAAGTCCGAATAA
TTCAACCATTTTCTTCTCGGAAATCCGATTTAATTCCATTTCATGAACATAACATCTTAAATACCTTGAAA
TATTCCTTCCATTAAATTGATCAATTTTATTGACCATTGCTTGCAATGCTTTGGTTGTGAAAGCTTCCTTT
TGATCATTGCTTTGCTGTTACTGCATTGATAATGAAAAGACTTGAAACATACTAGCAATAGACGGATCCAT
GTGCATACCTTATCCTTTATAAGAACCCCCACCAGAAACTCCTCCCAAAGATCCTTCAAAATTTTTATTTT
CATTCTCCATAAAAATAAAAATAAAATAAAACAAAATCCAATACCTTCCACCTCAAGCTTTCACGTCGCAT
TTACCACTTGTATCGTATTATGTGCTTTCCTACTCGA

>EACMGG116G_112132
TTAAGGAGCCTTTTTTGGCACTTCTTGGCACATGGAATTGTTGTTGGGCAGCACTAGCCATGTCAAGCTTA
CTTCCCAAAATTTAGTGAATTC

**Linkage Group 23 Scaffold 247 (SSR_849 identifies 2 possible scaffolds)**
>ECAMCG241G_175280
GAATTCCATGAGGAGTGGTCTTCATATATTGAAAAACTTTATGATGGATAAATTATATTTTTTACATACTC
CGATTTGTGTTCTCTTTGAAGATGATTGATAGGCCCTCTTGGCCATCGATTATATCTCATACTAAGGCAAA
GAGACCACCTTATTATTTGGTTGTTGTGGCGGCCTTCATAACTTCCTTGAAATAAAATATTTGAAACACCG
TTAA

**Linkage Group 24 Scaffold 15**
>ECAMCT208G_542960
GAATTCCATTACAATGATTGTCAACAGAAACATGGAACCACCTATACCACTACATTATTATTTTTCTTTTG
AAGACACCAAATGCACAACAGAGCAGCCAGTTTATGCCACCAATCTAGAGCAAAATGACACGTTACTCTAC
AGGCTCGTCATATGCTCATAAAAACTTCACTTACAGCGTAGTTAA
>EACMGT123G_800056
GAATTCACATGTGACCCCCAAAACCTATTTCATTCCCATGCCGGGCAGATGTTCAAATCTGCTTCAGTATG
TTTGAATACGGCACTCTGGTTCTCGACTTAA
>EACTGA117G_800056
GAATTCACATGTGACCCCCAAAACCTATTTCATTCCCATGCCGGGCAGATGTTCAAATCTGCTTCAGTATG
TTTGAATACGGCACTCTGGTTCTCGA
>EAGMCT313G_1343826
TTAACTTCGACCGCAATCGTAGTTACAAAATATTAGTGCATTCTACTTGTGATAGACCTCGACTGCAGCTC
CAATGATAAAACACTTATTTGGAATGGTATAGATCTGTGGTGAAAGAATCAGCCATTTCGGAATGGAGGGT
AAAGGATCTCACACATCAATACAACAGTGCAGTTTGCAGACAATAACGGTTCGTGGAGTCTGATTCCTGAT
GCTCCAGGTGGTATTTGCGTGACCTTGCTTGATCTGGTTATACATATCGGTGAACCCCGCGCACGAAGGCT
GAATTC
>EAGTCA162G_2809181
TCGACACGTTCTTGAGCAAGAAAATACGTAATATGACACCGGAACATCGTCCTTGTGATTGCTCAATTGAC
GTTCAAGAAGGAGCACAACCGCCATTCGTACCAATCTATAGTTTATCACAAAATGAATTGGCTGAATTC
>EACMGC332G_1122455
GAATTCACACATCTGAAAGGAATAAGGTACGGTGCTCCTCTAGGCCAGTGAGACATGGAATCTTTCAAAAC
ACGAGCATCGGCCATCAGTTCCCACCGTATGCTCATATGTCAACAAATGATGCTGAGGTTGTGGAAGAACT
TGGGCGATGCAAAGCAAAGAGGCCTGAAAGCAACTCCCAGGTTCGGAACCTAACATATTTGAAGGGATACC
TTGATTTATCTTCAAGGGAGAATGATTTGCCGATTGGGCCGTTACACGCACATGAAGAGCAGATAGGAGTG
GCGGAGTGATTCCTTGATTGCTTAA
>EACMGG165G_1562252
TTAAGGATTTTTTTAGTGTTTCTTTTTTTAAAATTATTTTTATTTCTTTGATATTTTTTATGAATTATTTA
TTGACTCTTTTAAAATTTCTTTAGGATTTATTTTAATATTTTTATTTTTATTTCTTTGATATTTTTTGTGA
ATTC

**Linkage Group 25 Scaffold 278**
>EACMCC454G_406588
GAATTCACGTCGATCCAATTGATTGAACCATAGTAAGAAGTAGTAGGGGAGCTCTTGACAGTCATCATGTT
TTCTTTTGATGGCAAACATGATGTCTGAGAAAGCAAGAATGGAGACCAATTGGAAGGAACGGCAAGAACGG
TTGCAGGTAGAGGTTGCGATGTTGCAGACTAAACTAGCTGCATTAGTAAAGAAGGCCAATAATTTAGTATG
GACCAATACGCCACTTTCAACGACAACACCTTCGGAGATATCTTTGAAACCTACAGAAGAATGACATAAGA
TGTTGGAGGAATGTGACAACCAAACTGCCCGACTTGAGTCTCAAAGACGGAAATTGGATTTATTGAATGAA
GATCTGACAACGCGTTTGGATGGAGAACCATCGAACACTGAGGCAGACAACGAAGATGTAGTAGAAGAAAA
GGTTAA

**Linkage Group 28 Scaffold 53**
>ECAMCC222G_21194

GAATTCCAGGGGAAAGTTATTCTCAGATCCTCACTAACCGCAATCGACCTCTACTCGAGACGCTTGAGGCA
TGCTTAGCCGATACGTACTTGAATGTATACTATGCTTTCCCTTGAAGCGGATGCTGGTAATTGAAAAGTAG
CTATCCAACACGATGGACTGGGAAGCAGAGTAAGATATTCTCTGAAAATGAAGGTTAA
>EACMCG386G_900752
GAATTCACGAAATACATCATTCATGAGATGTTGAAAAGCAACTGGTGCATTCGTGAGTCCAAAGGGCATGA
CATTATATTCAAAAAGTCCATACCTGGTTCGAAACGCAGTCTTCCATTCATCTCCTTCTTTGATCCGTACT
AGATTATATGCTCCCCTTAGATCTATTTTTGTATAGATCTTAGCTTGATTCAACTGGTCAAGGAGTCATGA
TATCAAAGGTAATGGATACTTATTCTTGATTGTGATCTTGTTGAGTCCTCCATAATCAACACACATTCGTA
AAGATCCATCTTTTTTCTTTACGAATAGAATTGGAGCTCCTGCTGGAGATTTGAATGGCATATGAAATTCT
TCGTTAA

**Linkage Group 28 Scaffold 103**
>EGAMGC49G_117556
GAATTCGAAAATGGAAAAGTGCAAGGCTTAA
>EACTGA145G_880510
TCGAGATCATCTCCGTAACTTCATTGAGATGATATGATGAGCAAGGCATCTATCTCTTGACTATCCATATC
ATATAAAATAGACAAGATCAAAAGAATATAAATAAGCTATAGTCGTGAATTC
>EACTGA224G_662349
GAATTCACCTACTAGACCGAATTTTTCTTGCATCATAAATCCGCTTTTGCTTGGACTTATAATAATTTAAA
AGATAATTCCGTTGAAGTCTGTGAGCATCATATCATTTTAGAAGAGAACGCTAAACCGGTACGGCAGAGGC
AACACCGGCTTAACCCAAAATATTCCCTTTTAGTAAAAGAAGAATTGGATAAACTTCTCGA

**Linkage Group 29 Scaffold 203**
>EACMGA159G_804951
GAATTCACCCAACTCATTTTGTGATAAATTGTAGATGGTCCAAATGGCGATTGTGCTTCTTTCTAAAGGTC
AATTGGGCAGTCATAGGAACGATGCTCTGGAAGAGTATCCGCATTTTTTTTCTCAAATACATCTTAA
>EAGMGG224G_520903
TTAAGGCTTTAAAACTTAGTTTTAATAAAAATAAACTTATTTTAGTTAAATTATATATTTGATTTATATAA
CATGTATCGAATATGAGAAGCTTACATGCCATTACTAATTGTTTCCTTTAAGATTTGAAAGAAAGGAGAAG
AGGAAAAAATAAGATGGAAAGAATTTATGTATAGAAATCAAATGAAGCCCTGAATTC

**Linkage Group 30 Scaffold 7: see also LG11**
>EACMTC399G_2230657
GAATTCACGCCTCTTAGAAGATCTTTCAACGGAAAGGACATTTCTTGAAATTGTGTAGAGAATGGAACAGC
TTGATACGGTGTCGTATTACATGCTTTCTTGCTCGAGAATGCAACAGGCGTCTGGCTGTAGAAGGTAGTAT
GGAGATTCGCTAATATTCAAGCAGTCTATCCGTTTTGGCTGTTGGAATATAAATAATTGTAAGAATATACC
ATAAATCTATACGGATGTCCTTCGATTGTTTGCCGAAATCCTTTTTGCCCCAGTCGGTAGGTCTCTAGACG
TTACAGAAAACCCCAAATAATCCAATCTGCTAATCTCCGATACCGCTTTATACTCACGACTTTGTATCCAC
TGACGGTCTTGTTAGATTAA
>EAGMCT160G_1857229
GAATTCAGGTTTGGCCGAAGGTCTTTGGCTAACGCACTGGACTGCACCACAGTTCGTGTGACATACCTGAC
AGTGTTTGCCACCTATTGCTCCAAACTTGTAATTACTTTTCCATTCTAGTTCCGTACTAGCTAGTTAA
>EACMGG174G_1215334
GAATTCACGGTGTACTGGGTATAAAGGGTCATGAATACTAATTCACTTGTTTTTTCTTGCTAACTGAAAAA
AGAAAAATACATCATTCTTTTTTAGAATCCTAGGGAACAATTTGGAACACGACACTGAGAATGACAGAGAT
ATTGTCCTTAA
>ECGMCC451G_655410
GAATTCCGCGATCCTGCCATTGACGGGGCAGCACATGAATCCCGGATCTCTAGCCCGCACGAAGAGCTCCT
CGGGAGCGGTTGCTGTGGACATGGCCATCCGGAACGTTGATGTGTACTCGCCAATTTTGAAAGGGTCGAAA
TTGGAGCCTCGGTCTCAACACAGCGTGCACTGCATCCCAGTGTTGATCGTCGTCGTGCTATTTCTTCTGTG
GGTCGGATCCAAAGGTGCGAAATCCTCTCTACACCTTCTCAGCATTGATCCAATTCTGCTAATGACGGTAT
TGTTCAGTTGATTCAGGTGGCGTTTGGAAATTCAAGTGGGGTCTTAGCTTCAGGGATTTTCTGCAGTGTGT
CTTTGGTTTCTTCCTTTTTTATGGGGATTTCTGGCCCTTGGGATCGATTGAAAGATAATCGACTGGTTAA

**Linkage Group 31 Scaffold 124**
>ECGTGA563G_548934
TCGAGAAATCTAGTCTATTTGAGGGTTGTATTTTTTTAACCACGATAAACCTAAAATGACTGGATTAGAGG
GAGATCTAATGATATTAAATACTACAAAACTATTATGATCGTTGATTATTACTTCTATAGGAAGGGTTTCG
TGAGTAATATCTCCAGAAGACAATGGACGACCATCTATGACTTCTATATGAACAGGTTTAGTTTTCTTTAC
TAAAGATATGCTATGTCTTTTAGTAAACTATAGTAGTTGGACAAACTCGTTTTCCGACATATTATCTTCTT
TCACAGCCATACTCCTCATTTTGTGATTGAGTCGTTTCTTTGGATAATGTTGAGCAGTGTGCTTTGGTTCT
CCACAATATAAACATAATCCTTCTTGACGACGACGAGTCTTTTCTTGCTCAGTAAGAGGTTTGAAACGTAT
TACATCAATTTCCATATCCTCTACTTCCAAATGTATGTGTTGAAGAGATGTTGGTGTTGCCATTGAATTAT
AGTAATATTGAGGTGTACTTGTACGATTTTCAGATCGGAATTC
>ECGTAC198G_1111484
TCGAACAACGGTGGCATTTCCCATTCTCAGACGTCAGCATCCACAACCGTCAAAAGAACAACCTTAACTTA
CAAAATCAGATTAATCCTGAGCAAGCTCAAATTCCAGGTTACAGGGACTTTAGGCATACTCAAGTGGTTCA
CAGGTTAACCAAAAATTGCAAACACGGAATTC
>EACMGG181G_1111479
GAATTCACGAATGTACAGATAACGTCAAGGTTCTGAAGATGGAAGACATGACAGACAGAGAGCCAAAACAG
TGCGTACTTGACGCGAACAGTAATCACTCCATGTCATGAAAGCCCCTCTCAAGGAAATTATCTCAAAACAG
GACAACACGAGAACCTTAA

**Linkage Group 31 Scaffold 83**
>EACMCC400G_129323
TTAACCCTAATAATCATAATCAGAATATACAATCGAGTGTACAAATTGTAGAAGAAAAAAATAAGGGGACA
AAGGAATCACCAGTTCATCGAGTAGATGTAGTTCAAGCTGTCCATACAAAAAGTCAGTAAAAAGGAAAAGG
ACCGATTCAACATTTGGGTGATCCTGAAGCAAAGGACAATTGGACCCAATTATGGGGACATCCAATCTAGA
TCCTGATATGGACTTTTTACTGAGTATAAGACCTGAATCGGTTGTGCATCCCAATGAGGTACCAATTATGG
GGGCTTCGATTCCCTTTCAATCTGTTTTGTTCTCTACACAGTTTCAAGAAACATCCTTTCCATTGAAAGAT
CCTCTCAGAGGCGTGAATTC

**Coalescent Linkage group 20+23 Scaffold 110**
>EAGMGC463G_538035
GAATTCACGAAGGAATTAGTTTAAAAGAGCTACTATTGAGATTTTTTTTATGCTGATAATAGCTTTTATGT
ATTTAGAATCACCTTTATTAAATATTGGTGACTCTTAAAATATTTTTTATATTTTATTTTTGATGTTTAAA
ACACATACTATACAAATCTTATTAAAAAAAGGATTGAAAAACAATCTCACTATCAAAAAGTGTAGTCAGGC
CATCCATATAGATGAATGAAGATAGATGATAGAAATCTTCATTTTCTTCGGCCAAGAAACATGTTTAATAG
ACTATAAGGAGATTATCTAAGGCCTTATTAGAAATTATGATTATTCTTTTTGAATAATTTAAATATCAAAT
TCTAAAGTCAAAGAGATTAAATTTTGGATGTAGTTTTGTATAAAAATTGAGTGATTTAAATTTTTTGTGAA
GGAGATATAGGTGCTTAA

>EACMGC422G_725890
TTAAGCATATAAGTACTACTATAACTTGAGAGAAGTTCTTGATAAAGATTCCATAGTAATTAATAAATCCC
AAAAAGCATTCTACATCTCTTACTGTTTTTGAAATCTTCCAATCCATGATTATATGAACTTTTTTAAGATC
CATCATTAGACTATTGTTTAAAATGATGTATCTTAAGAAGTAAACTTGAATAGTGTGGACTACACACTTTT
TTTTTATCTTGGCATACAATCTTGCATCTCTAAGTTTTTGTAACACATGACGAACATGAACTTTATATTTT
TTTACATTCTTAGAGTAGATTAAAATATCGTCAAAATAACAAATAACAAAATCATCTAGAAACTCATAAAA
AATATCATTCATAAGATGTTGAAAAGCAGTTAGTACATTCGTGAATTC

>ECAMCG256G_1345841
GAATTCCAAAAAAATATCGACTGCTGTTCCAATTCTGAGTCTAGGGTGGAAGAATATCTTATCTCACAGAT
TTAAACTCAAACCAATAAAAAGAAAGAGTGCATTAGAAATTATCCTAAATATAGAACCCTATGATATTTTG
AAGGATCTAGATGCTATTCAACCTTCTATTTCTATGAAGCAACTTCTAGCGGTCGCACCAGAGTGCCATTC
TACATTGAATTCATCGTTAA
