# Supplementary material for: A sequence-anchored genetic linkage map for the moss, Physcomitrella patens
Source: Plant J. 2008;56(5):855–66. doi: 10.1111/j.1365-313X.2008.03637.x (PMC2667646; doi:10.1111/j.1365-313X.2008.03637.x)
Supplement: Supplementary file 3 [file tpj0056-0855-SD3.pdf]

Supplementary Appendix 2: Segregation data

The allelic status of each marker was scored as “a” (Gransden allele) or “b” (Villersexel K3 allele).

Markers are listed by linkage group.

```
LG1:
EGAMGA505V           {0} ; 40
 aaaba aabaa aaaba babaa bbbab aabab aaaba abaaa abbba abaaa
 babba babaa aaaaa aabaa aaaaa abaab abaaa aabaa aaab- -----
 ----- ----- ----- ----- ----- ----- ----- ----- ----- -----
 ----- ----- ----- ----- ----- ----- ----- ---
EGAMGA522V           {0} ; 42
 abbbb aabaa aaaaa bbbba bbbbb babbb bbaba aabba bbaba abbaa
 bbabb aaaaa abbaa bbbab bbbbb aabba abbaa bbbaa bbba- -----
 ----- ----- ----- ----- ----- ----- ----- ----- ----- -----
 ----- ----- ----- ----- ----- ----- ----- ---
ECAMCG105bG          {0} ; 58
 aabba aabaa aaaba babba bbbab aabab aaaba abaaa abbba bbaaa
 babba babaa aaaaa aabaa aaaaa abbbb aaaaa aabaa aaab- -----
 ----- ----- ----- ----- ----- ----- ----- ----- ----- -----
 ----- ----- ----- ----- ----- ----- ----- ---
ECAMCG300V           {0} ; 76
 aaaba aabaa aaaba babaa bbbab aaaab aaaba abaab abbaa aaaaa
 babba bbaaa aaaaa aabaa baaaa abaab ababa abbab aaaa- -----
 ----- ----- ----- ----- ----- ----- ----- ----- ----- -----
 ----- ----- ----- ----- ----- ----- ----- ---
ECAMCG301G           {0} ; 77
 aaaba aabaa aaaba babaa bbbab aaaab aaaba abaab abbaa aaaaa
 babba bbaaa aaaaa aabaa baaaa abaab aaaba abbaa aaaa- -----
 ----- ----- ----- ----- ----- ----- ----- ----- ----- -----
 ----- ----- ----- ----- ----- ----- ----- ---
ECAMCG443G           {0} ; 85
 abbba bbabb aabba aabbb ababb bbbbb bbaaa abbbb bbaaa aabaa
 bbbba bbbab bbaab baabb aaaba bbbaa baaab bbaba bbba- -----
 ----- ----- ----- ----- ----- ----- ----- ----- ----- -----
 ----- ----- ----- ----- ----- ----- ----- ---
ECGMCG403G           {0} ; 97
 abbbb aaaaa babba babab babba baaab aaaab aabba bbaba aabba
 bbaba aaaaa abbaa aaaab bbaab abbaa babba bbbaa bbaa- -----
 ----- ----- ----- ----- ----- ----- ----- ----- ----- -----
 ----- ----- ----- ----- ----- ----- ----- ---
EGAMGC39G            {0} ; 101
 abbba abaab aabba aabbb bbbaa babba ababb abbba baaaa abbba
 bbaba bbbab bbaab bbbab bbaaa bbaaa babab bbaaa bbbbb baaaa
 aaaba abbaa aaaba baaab bbaab bbbba ababb aaaaa aabaa babab
 abaab baaab babba aaaba baabb aabaa babaa abb
EGAMGC67V            {0} ; 103
 baaab babaa bbbbb bbaaa abbbb abaaa abaab bbbbb babaa baaab
 bbabb abbbb babba bbbba aaabb abbaa baaab ababb abbba bbbbb
 abbbb baaba abaaa babba aabaa aabab bbabb abbaa aaabb bbbab
 abbbb aabba aaabb bbbbb abaab bbbbb babaa aab
EGAMGC82G            {0} ; 105
 aabaa aabaa aaaba babaa bbbab aabab baaba ababa abbba abaaa
 babba babaa aaaaa aabaa aaaaa abaab aaaaa aabaa aaabb abbbb
 bbbba aaaaa abaaa baaab bbaab bbbab babbb aaaaa ababa abaaa
```

abbaa aabaa bbbab abaaa baaaa aabba aaaba abb
EGAMGC144V {0} ; 112
babab bbabb aaaba abbab aabbb bbaba baabb aabbb baaab babbb
bbabb ababb babbb babaa aaabb bbaab baaaa abbba bbaba babba
abbba aaaba bbbab babaa abaab bbaba abbbb aaaba babaa bbaab
bbaab baaba aaabb bbbab abaab bbbba aabba abb
EGAMGC266G {0} ; 124
abbba abaab aabba aabbb babaa babba ababb ababa baaaa abbba
bbaba bbbab bbaab bbbab bbaaa bbaaa baabb bbaaa bbabb baaaa
aaaba abbaa aaaaa baaab bbaab bbbba ababb aaaaa aabaa babab
abaab baaab babaa aaaba aaabb aaaaa babaa abb
ECAMCC238V {0} ; 156
bbabb aabaa aabaa aabab baaba babab aaabb baaba bbaba aabba
bbaba abaab aabaa bbaab bbaab abbaa babba bbbaa -bbaa baaba
ababb baabb aaaab bbbbb baabb abbaa abbab aabbb babaa babbb
aabbb bbaab baaba aabbb bbaaa aabba babaa abb
ECGMCC94G {0} ; 180
bbabb aabaa aabaa aabab baaba babab aaaab baaba bbaba aabba
bbaba abaab aabaa bbaab bbaab abbaa babba bbbaa bbbaa baaba
ababb baa-b aaaab bbbbb baabb abbaa abaab aabbb babaa babbb
aabbb bbaab baaba aaaba bbaaa aabba aabaa aaa
EACMCC593V {0} ; 239
abaaa baaaa abaaa bbbaa bbbaa ababb aabbb abbaa aaaba bbbbb
babaa aabaa abbab abbaa ababa abbba aaaaa aaaab aaabb ababb
bbbbb aaaaa abbab aaaab bbaba bbbbb bbaba abaab abaaa baaaa
abbaa abaab bbbbb baabb bbbab bbaab bbbab aab
ECAMGG9IV {0} ; 245
bbbab aabaa aabaa aabab baaba babab aaabb baaba bbaba aabba
bbaba abaab aaabb aabba bbbab bbbaa bbbba bbbaa bbbba baaaa
ababb baabb aaaab bbbbb baabb aabaa abbab aabbb babba babbb
aabbb abaaa baaba aabbb bbaaa aabba babaa aab
ECAMGG128V {0} ; 250
baaaa babaa bbbbb abbaa aabbb abaaa bbabb aabbb babba bbabb
bbabb ababb abbab bbaba aabab bbbab baaaa ababb abbba babba
abbba bbaba abaab babba abbab aaabb baabb ababa aabab bbaab
bbabb babbb aaabb bbbbb abbaa bbbbb aabaa aab
ECAMGG146V {0} ; 251
bbbab aabaa aaaab aabaa baabb babbb babab bbaba bbaba abbba
bbaba babab aaaab abbbb bbaaa abbaa babbb bbaaa bbbaa baaaa
aaabb babbb aaaab babab baabb baaaa abbaa aaaab aabba baaab
aaabb bbaaa baaba baabb ababa aabba babaa abb
ECAMGG147G {0} ; 252
babab aabaa aaaaa aaaaa baabb babbb babab bbaba bbaba abbba
bbaba babab aaaab abbbb bbaaa abbaa babbb baaaa bbbaa baaaa
aaabb babbb aaaab babab baabb baaaa abbab bbbbb bbbba babbb
aaabb bbaaa baaba baaba bbaba aabba babaa abb
ECAMGG156V {0} ; 253
baaaa babaa abbab abbaa aabbb ababa bbabb aabbb baaba baabb
bbabb ababb abbab bbaba aabab bbbab baaaa abbbb abbba babba
abbba ababa abaab babaa abbab baaba bbabb ababa babab abaab
bbaab baabb aaabb abbbb abaaa bbbba aabaa aab
ECAMGG435G {0} ; 287
aaaaa bbbaa abaaa bbbaa bbbaa abaab aabbb abbaa aaaba bbbbb
babaa aabaa abbab aabaa ababa abbba aaaaa aaaab aaaab ababb
bbbbb aaaaa abbab aaaab ababa bbbbb bbbba abaab abaaa baaaa
abbba abaab bbbbb baaba bbbab bbaab bbbab aab
ECAMGG439G {1} ; 288

babbb ababb babbb aaabb aaabb babaa bbaaa baabb bbbab aaaaa
abbbb bbabb baaba bbabb babab baaab bbbbb bbbba bbbba babaa
aaaaa bbbbb baaba bbbba aabab aaaaa aaaab babba babbb abbbb
baabb babba aaaba abbaa aaaba aabba aaaba bba
ECAMGG446G {0} ; 290
abbbb aabaa aabba bbbba babbb babbb bbaab aabba bbabb abbaa
bbabb aaaaa baabb abbba bbbbb bbbba aabaa bbbaa bbbbb baaaa
abbba baaab baabb babab baaab abbbb abbba aabaa aabba babbb
aabba abaaa bbbba aabaa bbaba aabba bbbab bab
ECAMGG497V {0} ; 293
aabbb bbaba baaba abaaa babab baaab ababa abbbb baabb babaa
bbabb aaaaa aabbb babaa bbaba baabb bbbaa ababa abbaa baaaa
bbbbb aaaba bbbbb bbaaa bbaba bbbbb ababa baaaa aabaa bbabb
abbab baaba aabaa bbbab abbbb babba abbaa bbb
ECAMGG498G {0} ; 294
aabbb bbaba baaba abaaa babab baaab ababa abbbb baabb babaa
bbabb aaaaa aabbb babaa bbaba baabb bbbaa ababa abbaa baaaa
bbbbb aaaba bbbbb bbaaa bbaba bbbbb ababa baaaa aabaa bbabb
abbab baaba aabaa bbbaa abbbb babba abbaa bbb
ECGMTA59G {0} ; 299
abaaa babaa ababa bbbaa bbbaa aaabb aabbb abbaa aaaba bbbbb
baaaa aabaa abaab aabaa ababa abbba aaaaa aaaab aaabb ababb
bbbbb aaaaa abbab aaabb bbaba bbbbb bbbba abaab bbaaa bbaaa
abbab bbaab bbbbb baaba bbbab bbbba bbbab aab
ECGMTA92G {0} ; 303
abaaa baaba abaaa bbbaa bbbaa ababb aabab abbaa aaaba bbbbb
babaa aabaa abbab aabaa ababa abaaa aaaaa aaaab aaaba ababb
bbbbb aaaaa abbab aaaab bbaba bbbbb bbbba abaab abaaa baaaa
abbaa abaab bbbbb baaba bbbab bbaab bbbab aab
ECGMTA260V {0} ; 315
abaaa bbbaa aaaba bbbaa bbbaa aaabb aabbb abbaa aaaba bbbbb
baaaa aabaa abaaa aabaa ababa abbba aaaaa aaaab aaabb ababb
bbbbb aaaaa abbab baabb bbaba bbbab bbbba abaab abaaa baaaa
abbaa abaab bbbbb baabb bbaab bbbbb bbbab aab
ECGMTA314V {0} ; 319
ababa aabaa aaaba bbbaa bbbab aaaab aaaba abaaa aabba ababb
baaaa aabaa abaaa aaaaa ababa abaaa aaaaa aabba aaabb ababb
bbbbb aaaaa aaaab baabb bbaba abbab bbbbb abaaa abaaa abaab
abaaa abaab bbbab abaab baaab bbbbb babba aab
ECGMTA387V {0} ; 324
ababa ababa aabab aabaa baaab abbab baaba bbaaa bbbbb abaab
aabba bbabb babaa abaaa baaaa baabb aaaaa abbba aaaba abbbb
bbaba abbba aaaaa ababb aaaaa aabbb babbb aaabb abaaa baaaa
abbab abaab aabab babbb bbbba abaab baaba aab
ECGMTA387G {0} ; 325
ababa ababa aabab aabaa baaab abbab baaba bbaaa bbbbb abaab
aabba bbaaa aabaa abaaa baaaa aaaab aaaaa abbaa aaaba abbbb
bbaba ababa aaaaa ababb aaaaa aabbb babbb aaabb abaaa baaaa
abbab abaab aabab babba bbbba abaab baaba aab
EACMGA121V {0} ; 340
bbbbb babaa bbaba bbbba bbbab babab ababa abbab bbbbb abaaa
bbbbb bbbab abbaa babab bbbba abaab aabaa abbba abbab abbbb
bbbb- baaaa babbb aaaab bbbab abbbb bbbbb babba abbba bbbba
abbbb abbbb bbbab babab bbaba bbbba abbbb bbb
EACMGA290V {0} ; 364
abbaa abaab aabba aabbb babaa bbbbb ababb bbaba bbaaa abbba
bbaba bbbab bbaab babab baaaa bbaaa babab bbaaa bbabb baaaa

aaab- abbaa aaaaa baaab bbaab babaa ababb aaaaa aabba babbb
abaab baaaa baaaa aaabb aabba aabba babab abb
EACMGA336V {0} ; 366
abaaa abaaa aabab babaa babab abbab ababa bbaaa bbbba aaaaa
babba bbaaa aaaba aabaa baaaa abaab aaaaa abbab aaaaa abaab
bbab- abbab aaaaa abaab abbaa bbbab babbb aaabb abaaa ababa
abbbb abbab aabab baabb bbaba abaaa aaaba abb
EACMGA364G {0} ; 371
baabb aabaa bbbab bbaaa abbbb abaaa abaab bbbbb babaa baabb
bbabb ababb babba bbbbb aaabb abbaa baaab aaaba abbaa bbbbb
abbb- baaba abaab babaa aabaa aabab baabb abbaa aaaab bbbab
abbbb aabbb aaabb bbaba abaab bbbbb babaa aab
EACMGA559V {0} ; 390
aaaaa baaaa ababa aabba bbbab aabbb aaaba abaaa bbbab bbaaa
babba babaa aabba aabaa baaab abbba aaaab bbbaa aaabb bbbbb
babb- aaaaa aaaba baaab bbaba abbab babbb abaaa bbaaa bbaaa
bbaaa abbab bbbab bbbab bbbab bbbba abbbb abb
EAGMTA41G {0} ; 391
abaaa babaa abaaa bbbaa bbbaa ababb aabbb abbaa aaaba bbbbb
babaa aabaa baaba b-aaa ababa abbba aaaaa aaaab aaabb ababb
bbbb- aaaaa abbab aaaab bbaba bbbbb bbbba abaab abaaa baaaa
abbaa bbaab bbbbb baaab bbbab bbaab bbbab aab
EAGMTA120G {0} ; 396
baaab bbaaa babba bbaaa aaabb ababa bbaab bbbab baaaa bbaab
babab abbbb bbbba b-aaa aaaab aaaba baaab abbbb aabbb bbbbb
abbb- baaaa baaaa baabb aaaaa abbaa bbaba abaaa aabab babbb
abbbb aabbb aaaba abbaa abbbb bbaab baaab aab
EAGMTA152G {0} ; 397
ababa bbaab aabba aabab bbbba aabba abaab ababa baaba abbaa
bbaba bbbab bbaab b-aab bbaaa bbaaa baaab bbaaa bbabb aaaaa
aaab- abbaa aaaaa babab bbaab bbbba ababb abaaa abbba bbbab
abaab aabaa babab aaaaa ababb baaaa babaa abb
EAGMTA207G {0} ; 402
abbba bbabb aabba aabbb ababb bbbbb bbaaa abbbb bbaaa aabaa
bbbba bbbab bbaab b-abb aaaba bbbaa baaab bbaba bbbab baaab
babb- aaaaa aaabb bbbab baaab bbbaa ababa abaaa abbbb bbbab
bbbab aabaa aabab aaaab ababb bbaaa bbbaa abb
EAGMTA246V {0} ; 408
abbba bbaab aabba aabbb bbbaa aabba abaab ababa baaaa abbba
bbaba bbbab bbaab b-bab bbaaa bbaaa baaab bbaaa bbabb baaaa
aaab- abbaa aaaaa baaab bbaab bbbba ababb abaaa aabba bbbab
abaab aabaa babab aaaba ababb aaaaa babaa abb
EAGMTA406V {0} ; 421
ababa abaaa aabab babaa babab abbab aaaba bbaaa bbbaa aaaaa
babba bbaaa aaaba a-baa baaaa abaab aaaaa abbaa aaaaa abaab
bbab- abaab aabaa abaab abbaa bbbab babbb aaabb abaaa ababa
abbbb abbab aabab baabb aaaba abaaa aaaba abb
ECGMGA235V {0} ; 435
bbbbb babaa bbaaa ababa babab baaab baaba abbab bbbba abaaa
bbabb aaabb abbaa b-bab bbbba abaab aabaa abbba bbbab baaaa
bbbb- baaab babba aaaaa babab ababb abbba babaa aabba bbbba
abbab ababa abbaa aabbb ababa abbba abbab bbb
ECGMGA238G {0} ; 436
bbbbb babaa bbaaa ababa babab baaab baaba abbab bbbba abaaa
bbabb aaabb abbaa b-bab bbbba abaab aabaa abbba abbab baaaa
bbbb- baaab babba aaaaa babab ababb abbba babaa aabba bbbba
abbab ababa abbaa aabab ababa abbba abbab bbb

EACMTC148V {0} ; 463
abbab aabaa aabab aabaa baabb babbb abbbb bbaba bbaba abbba
bbaba babab bbaaa bbbbb bbaa- abbaa babbb bbaaa bbbaa baaaa
aaabb babbb aaaab babab baabb baaaa abbab aabbb babba babbb
aaabb bbaaa baaba baaba bbaba aabba babaa abb
EACMTC202V {0} ; 476
ababb aaaaa babba babab babba baaab aaaab aabba bbaba aabba
bbaba aaaaa abbaa aaaab bbaa- abbaa babba bbbaa bbaab baaba
abbba baaab aaaab bbbab bbaab abbbb abbab aabaa babba babab
aabbb bbaaa bbabb aabbb bbaaa aabba babaa bab
EACMTC207G {0} ; 477
abbbb babaa babba babab babba baaab aaaab aabba bbaba aabba
bbaba aaaaa abbaa aaaab bbaa- abbaa babba bbbaa bbaab baaba
abbba baaab aaaab bbbab bbaab abbbb abbab aabaa babba babbb
aabbb bbaaa bbabb aabab bbaaa aabba babaa bab
EACMTC209G {0} ; 478
abbbb aaaaa babba babab babba baaab aaaab aabba bbaba aabba
bbaba aaaaa abbaa aaaab bbaa- abbaa babba bbbaa bbaab baaba
abbba baaab aaaab bbbab bbaab abbbb abbab aabaa babba babbb
aabbb bbaaa bbabb aabab bbaaa aabba babaa bab
EACMTC216G {0} ; 480
abbba bbabb aabaa aabbb ababb bbbbb bbaaa abbbb bbaaa aabaa
bbbba bbbab bbaab baabb aaab- bbbaa baaab bbaba bbbab baaab
babba aaaaa aaabb bbbab baaab bbbaa ababa abaaa abbbb abbab
bbbab aabaa aabab aaaab ababb bbaaa bbbaa abb
EACMTC417V {0} ; 514
aaaba aabaa aaaba babba bbbab abaab aaaba abaab abbaa aaaaa
babba bbaaa aaaaa aabaa baaa- abaab aaaba abbaa aaaab abbab
abbba abaaa aabaa aaaab bbaab bbbab babbb aaaba abaaa ababa
abbba aabab bbbab aaaba baaba abaab aaaba abb
EACMTC420V {0} ; 516
aaaba aabaa aaaba babaa bbbab abaab aaaba abaab abbaa aaaaa
babba bbaaa aaaaa aabaa baaa- abaab aaaba abbaa aaaab abbab
abbba abaaa aabaa aaaab bbaab bbbab babbb aaaba abaaa ababa
abbba aabab bbbab aaabb baaba abaaa aaaba abb
EACMTC424V {0} ; 517
abbba bbbbb aabba aabbb ababb bbbbb bbbaa abbbb abaaa aabaa
bbbba bbbab bbaab bbabb aaab- bbbaa baaaa bbabb bbbab babab
babba aaaaa ababb bbabb baaab bbbaa ababa abaaa abbbb abbab
bbbab aabaa aabab aaabb ababb bbaab bbbaa abb
EACMTC548G {0} ; 530
baaab babaa bbbbb abbaa aabbb abaaa bbaab aabbb babba bbabb
bbabb ababa aaabb bbbab aaba- bbbab baaaa ababb abbba babba
abbba bbaba abaab babba abbab aaaba aaabb ababa aabab bbaab
bbaba babbb aaabb bbbaa abbaa bbbbb aabaa aaa
EACMTC548V {0} ; 531
baaab babaa bbbbb abbaa aabbb abaaa bbaab aabbb babba bbabb
bbabb ababa aaabb bbbab aaba- bbbab baaaa ababb abbba babba
abbba bbaba abaab babba abbab aaabb aaabb ababa aabab bbaab
bbabb babbb aaabb bbbba abbaa bbbbb aabaa aab
ECAMCT134G {0} ; 553
baaaa aaaaa bbbbb bbaaa abbbb abaaa bbaab babbb babaa baaab
aaabb abbbb babba bbbba aaab- abbaa baaaa ababa ababa bbbbb
abbbb aaaba abaaa babba aabaa aabab bbaba abaaa aaaab abaab
abbab aabbb aaabb bbbaa abaab bbbbb baaaa abb
ECAMCT210V {0} ; 564
abbaa aabaa aaaab aabaa baabb babbb abbab bbaba bbaba abbba

```
 bbaba babab baaaa babbb bbaa- abbaa babbb bbaaa bbbaa baaaa
 aaabb babbb aaaab babab baabb baaaa abbab aabbb babba babbb
 aaabb bbaaa baaba baaba bbaba aabba babaa abb
ECAMCT403V          {0} ; 587
 bbbba aaaaa aaaba bbaba babbb babbb bbaaa aabba bbaba abaaa
 bbabb aaaba abbaa bbbab bbbb- aabbb aabaa abbba bbbab baaaa
 bbbba baaab baabb babab baaab ababb abbba aabaa aabba bbbba
 abbba abaab bbbaa aabbb bbaba abbba bbbab bbb
ECAMCT406G          {0} ; 588
 bbbbb babaa aaaba bbaba babbb babbb bbaaa aabba bbaba abaaa
 bbabb aaaba abbaa bbbab bbbb- aabbb aabaa abbba bbbab baaaa
 bbbba baaab baabb babab baaab ababb abbba aabaa aabba bbbba
 abbba abaaa bbbaa aabab bbaba abbba bbbab bbb
ECGMTT184V          {0} ; 603
 abbba abaab aab-a aabbb bbbaa aabba baaab ababa baaaa abbba
 bbaba bbbab bbaab bbbab bba-- bbaaa baaab bbaaa bbabb baaaa
 aaaba abbaa aaaaa baaab bbaab bbbba ababb abaaa aabba bbbab
 abaab aabaa babaa aabba ababb aaaaa babaa abb
ECGMTT193V          {0} ; 605
 abbbb aabaa aab-a bbbba bbbbb babbb ababb aabba bbaba aabaa
 bbaba aaaaa abbaa bbbab bba-- abbba aabaa bbbba bbbab baaaa
 bbbba baaab baabb babab bbaab abbbb abbba aabaa aabba babbb
 aabbb bbaaa bbbba aabbb bbaba aabba bbbab bab
ECGMTT255V          {0} ; 612
 ababa ababa bab-b aabaa baaab abbab baaba bbaaa bbbbb abaab
 aabba bbaaa aaaba aabaa baa-- aaabb aaaaa abbba aaabb ababb
 bbaba abbba aaaaa ababb aaaaa aabbb babbb aaabb abaaa baaaa
 abbab abaab aabab babba bbbba abaab baaba aab
EACMCG63G           {0} ; 629
 bbbbb babaa abaaa ababa babab babbb baaaa abbba bbbba abaaa
 bbabb aaabb abbaa bbbab bbbbb abaaa aabaa abbba bbbab baaaa
 bbbba bbaab baaba aabab baaab ababb abbba aabaa aabba bbbba
 abbbb abaaa bbbaa aabaa abaaa abbba abbab bbb
EACMCG388V          {0} ; 660
 bbbbb bbbbb aabba aabbb ababb bbbbb bbaaa abbbb bbaab aabaa
 bbbba bbbab bbaab baabb aaaba abbaa baaab bbabb bbbab aaaab
 babba aaaaa aaabb abbab baaab bbbaa ababa abaab abbbb abbab
 bbbab aabaa aabab aaabb aaabb bbaaa bbbaa abb
EACMCG436V          {0} ; 661
 abbbb aaaaa babba babab babba baaab aaabb aabba bbaba aabba
 bbaba aaaaa abbaa aaaab bbaab abbaa babba bbbaa bbaab baaba
 abbba baaab aaaab bbbab bbaab abbbb abbab aabaa babba babbb
 aabbb bbaaa bbabb aabbb bbaaa aabba babaa bab
EAGMGG123G          {0} ; 669
 aabba bbaba bbaaa abaaa baaab baaab baaba abbbb bbabb babaa
 bbabb aaaaa aabba bbaaa bbaba baabb babaa ababa abbaa baaaa
 bbbbb aaaba bbbbb bbaaa bbabb bbbbb ababa aaaaa aabaa bbabb
 abbab baaba aabaa babaa abbbb babba abbaa bbb
EAGMGG146V          {0} ; 672
 baaab babaa bbbbb bbaaa abbbb abaaa abaab bbbbb babaa baaab
 bbabb abbbb babba bbbba aaabb abbaa baaab ababb abbba bbbbb
 abbbb baaba abaaa babba aabaa aabab bbabb abbaa aaaab bbbab
 abbbb aabbb aaabb bbbbb abaab bbbbb babaa aab
EAGMGG172G          {0} ; 677
 ababa aabaa aabba bbbba babbb babbb baaab abbba bbaba aabaa
 bbaba aaaaa abbaa aabab bbabb abbba aabba bbbaa bbaab baaaa
 bbbba baaab baabb babab bbaab abbab aabba aabaa aabba bbbbb
```

```
 aaaab bbaaa bbbba aabba bbaba aabba bbbaa bab
EAGMGG255G          {0} ; 690
 baaab baaaa bbbbb bbaaa abbbb abaaa bbaab bbbbb babaa baaaa
 bbabb abbbb babba bbbba aaabb abbaa baaab ababa abbba bbbbb
 abbbb baaba abaaa babba aabba aabab bbaba abbaa aaaab bbbab
 abbbb aabbb aaabb bbbba abaab bbbbb baaaa abb
EAGMGG371V          {0} ; 696
 abbba aaaab aabba aabab babaa bbbbb abbab bbaba bbaba abbba
 bbaba babab bbaab bbbbb baaaa abbaa babbb bbaaa bbaba baaaa
 aaaba babaa aaaab baaab baabb baaaa abaab aaaab aabba babbb
 aaaab baaaa baaaa baabb baaba aabba babab aba
EAGMGG375G          {0} ; 698
 abbba aaaab aabba aabab babaa bbbbb abbab bbaba bbaba abbba
 bbaba babab bbaab bbbbb baaaa abbaa babbb bbaaa bbaba baaaa
 aaaba babaa aaaab baaab baabb baaaa abaab aaaab aabba babbb
 aaaab baaaa baaaa baaba baaba aabba babab aba
EACMGG84V           {0} ; 737
 abbba bbaba bbaaa abaaa baaab baaab baaba abbbb bbabb b-baa
 bbabb aaaaa aabaa bbaaa bbaba baabb babaa ababa abbab baaaa
 bbbba aaaba bbbbb bbaaa bbabb bbbbb a-aaa aaaaa aabaa bbaab
 abbab baaba aabaa babbb abbbb babba abbab bbb
EACMGG114V          {0} ; 742
 bbbaa aabaa aaaab aabaa baabb babbb abbbb bbaba bbaba a-bba
 bbaba babab aaaaa bbbbb bbaab abbaa babbb bbaaa bbbaa baaaa
 aaabb babbb aaaab babab baabb baaaa a-bab aabbb babba babbb
 aaabb bbaaa baaba baaba bbaaa aabba babaa abb
EACMGG160G          {0} ; 750
 bbbbb babba bbaaa ababa babab baaab ababa abbab babbb a-aaa
 bbabb aaaab abbaa bbaab bbbba abaab aabaa abbba abbab baaaa
 bbbba baaaa babbb abaaa babab ababb a-bba babaa aabbb bbbba
 abbab ababa aabaa babab ababa bbbba abbab bbb
EACMGG232G          {0} ; 763
 abbbb aaaaa babba babab babba baaab aaaab aabba bbaba a-bba
 bbaba aaaaa aabaa aaaab bbaab abbaa babba bbbaa bbaab baaba
 abbba baaab aaaab bbbab bbaab abbbb a-bab aabaa babba babbb
 aabbb bbaaa bbabb aabab bbaaa aabba babaa bab
EACMGG257V          {0} ; 767
 abbaa aabaa aabab aabaa baabb babbb abbbb bbaba bbaba a-bba
 bbaba babab baaaa bbbbb bbaaa abbaa babbb bbaaa bbbaa baaaa
 aaabb babbb aaaab babbb baabb baaaa a-bab aabbb babba babbb
 aaabb bbaaa baaba baaba bbaba aabba babaa abb
EACMGG466G          {0} ; 791
 abbba bbbbb aabba babbb ababb bbbbb bbbaa abbbb abaaa a-baa
 bbbba bbbab bbaab bbabb aaaba bbbaa baaaa bbaba bbbab babab
 babba aaaaa ababb baabb baaab bbbaa a-aba abaaa abbbb abbab
 bbbab aabaa aabab aaaab ababb bbabb bbbaa abb
EACMGG501G          {0} ; 794
 ababa abaaa aabab babaa babab abbab aaaba bbaaa bbbba a-aaa
 babba bbaaa aaaba aabaa baaaa abaab aaaaa abbaa aaaaa abaab
 bbaba abaab aaaaa abaab abbaa bbbab b-bbb aaabb abaaa ababa
 abbbb abbab aabab baaab bbaba abaaa aaaba abb
EAGMCT70G           {0} ; 800
 bbbbb babaa bbaaa ababa babab baaab baaba abbab bbbba a-aaa
 bbabb aaabb abbaa bbbaa bbbba abaab aabaa abbba abbab baaaa
 bbbba baaab babba aaaaa babab ababb a-bba babaa aabba bbbba
 abbab ababa abbaa aabab ababa abbba abbab bbb
EAGMCT229G          {0} ; 820
```

```
 abbba aaaab aabba aabab babaa bbbbb babbb bbaaa bbaba a-bba
 bbaba babab bbaab bbbbb baaaa bbbaa babbb bbaaa bbaba baaaa
 aaaba aabaa aaaab baaab bbaab baaaa a-aab aaaab aabba babbb
 aaaab baaaa babba baaaa baaba aabba babab aba
EAGMCT273G           {0} ; 824
 abbba abaab aabba aabbb babaa babbb baaab ababa bbaaa a-bba
 bbaba bbbab bbaab bbbab baaaa bbaaa aabab bbaaa bbabb baaaa
 aaaba abbaa aaaaa baaab bbaab babaa a-abb aaaaa aabba babbb
 abaab baaaa babaa aaaaa aabbb aabba babab abb
EAGMCT403G           {0} ; 839
 bbbbb aaaaa aabba bbbab baaba baaab aaaab aabba bbaba a-bba
 bbaba abaaa abbaa abaab bbaab abbaa babba bbbaa bbaab baaba
 abbbb baaab aaaab bbbab bbaab abbbb a-bab aabbb babba babbb
 aabbb bbaaa bbabb aabaa bbaaa aabba babaa aab
ECGMGT178G           {0} ; 853
 abbbb bbaab aabba aabbb babba aabba baaab ababa baaaa a-baa
 bbaba bbbab bbaab bbaab bbaaa bbaaa baaab bbaaa bbaab baaaa
 aaaba abaaa aaaaa babab bbaab bbbba a-abb abaaa abbba bbbab
 abaab aabaa babab aaaaa ababb baaaa babaa abb
ECGMGT274G           {0} ; 860
 abbba abaab aabba abbbb babaa babbb baaab ababa bbaaa a-bba
 bbaba bbbab bbaab bbbab baaaa bbaab babab bbaaa bbabb baaaa
 aaaba abbaa aaaaa baaab bbaab babaa a-abb aaaaa aabba babbb
 abaab baaaa babaa aaaaa aabbb aabba babab abb
ECGMGT446G           {0} ; 862
 aaaba bbabb aaaba abaaa aabbb baaba ababa abbbb baaab b-bba
 bbabb aaaaa aabbb babaa aaabb baaab bbbaa abbba bbaba baaba
 abbba aabbb bbbab baaaa bbaba bbbba a-abb baaaa aabba bbabb
 abaab baaba aabba bbbab abbab bbbba aabaa abb
EACMGC149V           {0} ; 880
 aabba bbaba bbaaa abaaa baaab baaab baaba abbbb bbabb babaa
 bbabb aaaaa aabba bbaaa bbaba baabb babaa ababa abbaa baaaa
 bbbbb aaaba bbbbb bbaaa bbabb bbbbb ababa baaaa ababa babbb
 bbabb aabba abbaa abaab babba bbabb aabaa aba
EACMGC277V           {0} ; 895
 abbaa aabaa aaaab aabaa baabb babbb abbbb bbaba bbaba abbba
 bbaba babab baaaa bbbbb bbaaa abbaa babbb bbaaa bbbaa baaaa
 aaabb babbb aaaab babab baabb baaaa abbab aabbb abaab abbab
 aaabb ababa aabbb abbab abbba aabbb baaab aab
EACMGC506V           {0} ; 922
 abbba bbaab aabba aabbb bbbaa aabba abaab ababa baaaa abbba
 bbaba bbbab bbaab bbbab bbaaa bbaaa baaab bbaaa bbabb baaaa
 aaaba abbaa aaaaa baaab bbaab bbbba ababb abaaa baaab aabab
 baaaa ababa aabba abbab bbaba baaba abaab aba
EAGMCA99V            {0} ; 935
 abbba bbaab aabba aabbb bbbba aabba baaab ababa baaaa abbaa
 bbaba bbbab bbaab bbaab bbaaa bbaaa baaab bbaaa bbabb baaaa
 aaaba abbaa aaaaa babab bbaab bbbbb ababb abaaa baaab aabab
 bbaaa ababa aabba bbbab bbaba baaba abaab aba
EAGMCA199V           {0} ; 947
 abbaa aabaa aaaab aabaa baabb babbb babbb bbaba bbaba abbba
 bbaba babab baaaa bbbbb bbaaa abbaa babbb bbaaa bbbaa baaaa
 aaabb babbb aaaab babab baabb baaaa abbab aabbb abaab abbab
 aaabb ababa abbbb abbab abbba aabbb baaab aab
EAGMCA213V           {0} ; 948
 ababb ababa aaabb aabaa baaaa abbab baaba bbbaa bbbba abbab
 aabba bbaaa ababa aaaba baaaa aaaab aaaaa bbbaa aaaba bbbba
```

bbaba ababa aaabb ababb aaaaa aabbb aabbb bbabb bbbbb abbbb
aabaa bbaaa baaba baaab abaaa bbaba baaba aaa
EAGMCA319G {0} ; 963
abbba bbbbb aabba babbb ababb bbbbb bbbaa abbbb abaaa aabaa
bbbba bbbab bbaab bbabb aaaba bbbaa baaaa bbaba bbbab babab
babba aaaaa ababb baabb baaab bbbaa ababa abaaa baaab babab
bbbbb bbbba aabba bbbba bbabb bbaba abaaa aba
EAGMCA357V {0} ; 966
abbba abaab aabba aabbb babaa babba baabb ababa baaaa abbba
bbaba bbbab bbaab bbbab bbaaa bbaaa baaab bbaaa bbabb baaaa
aaaba abbaa aaaaa baaab bbaab bbbba ababb aaaaa aaaab aaaab
baaaa ababa aabba abbab ababa baabb aaaab aba
EACMGT107V {0} ; 1011
aabba bbaba aaaba abaab babbb baaaa baaba abbbb baaab babaa
bbabb aaaaa aabbb bbbaa bbaba baaab bbbaa ababa abbba baaaa
bbbbb aaaba bbbbb bbaaa bbaba bbbbb ababb baaaa aabaa bbabb
abaab baaba aabaa bbbab abbbb bbbba abbaa abb
EACMGT238V {0} ; 1023
bbbab aabaa aabaa babab baaba babab aaabb aaaba bbaba aabba
bbaba abaab abbaa bbaab bbaab abbaa babba bbbaa bbaab baaba
ababb baabb aaaab bbbbb baabb abbaa abbab aabbb babba babbb
aabbb bbaaa bbaba aabbb bbaaa aabba babaa aab
EACMGT332V {0} ; 1031
bbbbb babba bbaba ababa babab baaab ababa abbab babbb aabaa
bbaab aaaab abbaa bbaab bbaba bbaab aabaa abbba abbab baaaa
bbbba baaaa babbb abaaa baaab abbbb abbba baaaa aabbb bbbba
abbab ababa aabaa babab ababb bbbba abbab bba
EACMGT389G {0} ; 1035
baaab babaa aabaa abbaa aabbb ababa bbabb aabbb baaba baabb
bbabb ababb baabb babab aabab abbab baaaa abbba abbba babba
abbba ababa abaab babba abbab bbaba bbabb ababa babab abaab
bbaab baabb aaabb abbba aaaaa bbaaa aabaa bab
EACMGT392V {0} ; 1036
baaab babaa abaab abbaa aabbb ababa bbabb aabba baaba baabb
bbaab ababb baabb babab aabab abbab baaaa abbbb abbba babba
abbba ababa abaab babba abbab baaba bbabb ababa babab abaab
bbaab bbabb aaabb abbbb abaaa bbbba aabaa aaa
EAGMTT95V {0} ; 1044
abbba aabab aabba aabab baaab babbb abbbb bbaba bbaba abbba
bbaba babab baaaa bbbbb baaaa abbaa babbb bbaaa bbbaa baaaa
aaabb babba aaaab babab baabb baaaa abbab aaabb babba babbb
aaaab bbaaa baaba baabb baaba aabba babaa aba
EAGMTT101G {1} ; 1045
baaaa bbbbb aaaab ababa abaab abbba bbbba bbaab aabab bbaab
aabab bbbbb baabb bbbba aabba baaab abaab aaabb aabba abbab
baaab abbba bbbba aaaba aabba baaaa baaba bbabb abaab abaaa
bbaaa aabbb aabaa bbaaa aabbb bbaab ababb aba
EAGMTT114G {0} ; 1048
abbba abaab aabba aabbb babaa babbb ababb ababa bbaaa abbba
bbaba bbbab bbaab bbbab baaaa bbaaa babab bbaaa bbabb baaaa
aaaba abbaa aaaaa baaab bbaab babaa ababb bbbaa babba babbb
abaab baaaa babaa aaaba aabbb aabba babab abb
EAGMTT130V {0} ; 1049
abbba bbabb aabba aabbb ababb bbbbb bbaaa abbbb bbaaa aabaa
bbbba bbbab bbaab baabb aaaba bbbaa baaab bbabb bbbab baaab
babba aaaaa aaabb bbbab baaab bbbaa ababa abaaa abbbb bbbab
bbbab aabaa aabab aaabb ababb bbaaa bbbaa abb

EAGMTT344V {0} ; 1072
baabb bbabb aaaba abbab aabbb bbaaa baaab aabbb baaab babbb
bbabb ababb babbb babaa aaabb bbaab baaaa abbba bbaba bbaba
abbba aaaba abbab babaa abaab bbaba abbbb aaaba babaa bbaab
bbaab baaba aaabb bbbab abaab bbbba aabba abb
EAGMTT401V {0} ; 1076
abaaa baaaa abaaa bbbaa bbbaa ababb aabbb abbaa aaaba bbbbb
babaa aabaa baaba bbaaa ababa abbba aaaaa aaaab aaabb aabbb
bbbbb aaaaa abbab aaaab bbaba bbbbb bbbba abaab bbaaa baaaa
abbaa abaab bbbbb baabb bbbab bbaab bbbab aab
EACTCC104G {0} ; 1121
aabba bbaba aaaba abaab babbb baabb baaba abbbb baaab babaa
bbbab aaaaa aabbb babaa ababb baaab bbbaa ababa abbba baaba
bbabb aaaaa bbbbb bbaaa bbaba bbbbb ababb baaaa aabaa bbabb
bbaab baaba aabaa bbbab abbbb bbbaa aabaa abb
EACTCC377G {0} ; 1142
bbbab aabaa aabaa babab baaba babab aaaab aaaba bbaba aabba
bbbaa abaab abbaa bbaab bbaab abbaa babba bbbaa bbaab baaba
ababb baaab aaaab bbbbb baabb abbaa abbab aabbb babba babbb
aabbb bbaaa bbaba aabba bbaaa aabaa babaa aab
EACTCC506G {0} ; 1152
abbbb aaaaa babba babab babba baaab aaaab aabba bbaba aabba
bbbaa aaaaa abbaa aaaab bbaab abbaa babba bbbaa bbaab baaba
ababb baaab aaaab bbbab bbaab abbbb abbab aabaa babba babbb
aabbb bbaaa bbabb aabbb bbaaa aabaa babaa bab
EAGTTA233G {0} ; 1167
abbba abaab aabba aabbb babaa bbbbb abaab bbaba bbaaa abbba
bbbaa bbbab bbaab bbbab baaaa bbaaa babab bbaaa bbabb baaaa
aaaba abbaa aaaaa baaab bbaab babaa ababb aaaaa aabba babbb
abaab baaaa baaaa aaaba aabba aabaa babab abb
ECGTGT43V {0} ; 1176
abbba bbaab aabba babbb bbbaa aabba abaab ababa baaaa abbba
bbbaa bbbab bbaab bbbab bbaaa bbaaa baaab bbaaa bbbbb baaaa
aabba abaaa aaaaa baaab bbaab bbbaa ababb abaaa aabba bbbab
abaab aabaa babab aaaba ababb aaaaa babaa abb
ECGTGT106V {0} ; 1181
abbba abaab aabba aabbb babaa babba ababb ababa baaaa abbba
bbbaa bbbab bbaab bbbab bbaaa bbaaa baaab bbaaa bbbbb baaaa
aabba abbaa aaaaa baaab bbaab bbbba ababb aaaaa aabba babab
abaab baaaa babaa aaaba aaabb aaaaa babaa abb
ECGTGT119G {0} ; 1186
abbba bbaab aabba aabbb bbbba aabba abaab ababa baaaa abbaa
bbbaa bbbab bbaab bbaab bbaaa baaaa baaab abaaa bbbbb baaaa
aaaba abbaa aaaaa babab bbaab bbbba ababb abaaa abbba bbbab
abaab aabaa babab aaaba ababb baaaa babaa abb
EACTAG78V {0} ; 1224
aaaba aabaa aaaba babaa bbbab aabab aaaba abaaa abbaa abaaa
babba bbbaa aaaaa aabaa baaaa ababa aaaaa aabaa aaaab abbbb
abbba aabaa aaaaa aaaab bbaa- abbab babbb aaaba abaaa abaaa
abbba aabab bbbab baaab baaba abbba aaaba abb
EACTAG81G {0} ; 1225
aaaba aabaa aaaaa babaa bbbab aabab aaaba abaaa abbaa abaaa
babba bbbaa aaaaa aabaa baaaa ababa aaaaa aabaa aaaab abbbb
aabba aaaaa aaaaa aaaab bbaa- abbab babbb aaaba abaaa abaaa
abbba aabab bbbab aaaab baaba abbba aaaba abb
EACTAG211G {0} ; 1246
ababb ababa aabab aabaa baaaa abbab ababa bbbaa bbbbb abbab

```
 aabba bbaaa ababa aabba baaaa aaaba aaaaa bbbaa aaaba bbbba
 baaba ababa aaaab ababb aaaa- aabbb aabbb ababb abaaa babaa
 abbab abaab aabab bbbba bbbba ababb baaab aab
EACTAG231G          {0} ; 1247
 aabba bbaba aaaaa abaab babbb baabb baaba abbbb baaab babaa
 bbbab aaaaa aabbb babaa ababb baaba bbbaa ababa abbba baaba
 babbb aaaba bbbbb bbaaa bbab- bbbbb abbbb baaaa aabaa bbabb
 abaab baaba aabaa bbbab abbbb bbbaa abbaa abb
EACTAG263G          {0} ; 1252
 abbba bbaab aabba aabbb bbbba aabba abaab ababa baaaa abbaa
 bbbaa bbbab bbaab bbaab bbaaa bbaaa baaab bbaaa bbabb baaaa
 aaaba abbaa aaaaa babab bbaa- bbbba ababb abaaa abbba bbbab
 abaab aabaa babab aaaba ababb baaaa babaa abb
EACTAG468V          {0} ; 1279
 abbbb bbabb aabba aabbb ababb bbbbb bbaaa abbbb bbaaa aabaa
 bbbba bbbab bbaab baabb aaaba bbbaa baaab bbaba bbbab baaab
 bbbba aaaaa aaabb bbbab baaa- bbbaa ababa abaaa abbbb bbbab
 bbbab aabaa aabab aaabb ababb bbaaa bbbaa abb
EAGTCA127G          {0} ; 1288
 aaaba aabaa aaaaa babaa bbbab aabab aaaba abaaa abbba abaaa
 babba babaa aaaaa aabaa aaaaa abaab aaaaa aabaa aaabb abbbb
 babba aaaaa aaaaa baaab bbaa- abbab babbb aaaaa abaaa abaaa
 abaaa aabab bbbab abaab baaaa aabba aaaba abb
EAGTCA163G          {0} ; 1292
 abbbb aaaaa babba babab baaba baaab aaaab aabba bbaba aabba
 bbbaa abaaa abbaa aaaab bbaab abbaa babba bbbaa bbaab baaba
 aabbb baaab aaaab bbbab bbaa- abbbb abbab aabbb babba babbb
 aabbb bbaaa bbabb aabba bbaaa aabaa babaa bab
ECGTAC331V          {0} ; 1339
 bbbaa aabaa aaaab aabaa baabb babbb abbab bbaba bbaba abbba
 bbbaa aabab aaaaa bbbbb bbaab abbaa babba bbaaa bbbaa baaaa
 ababb babbb aaaab babab baab- aaaaa abbab aabbb babba babbb
 aaabb bbaaa baaba baaba bbaaa aabaa babaa abb
ECGTAC333G          {0} ; 1340
 bbbaa aabaa aaaab aabaa baabb babbb abbab bbaba bbaba abbba
 bbbaa aabab aaaaa bbbbb bbaab abbaa babba bbaaa bbbaa baaaa
 aaabb babbb aaaab babab baab- aaaaa abbab aabbb babba babbb
 aaabb bbaaa baaba baaba bbaaa aabaa babaa abb
EACTGA50V           {0} ; 1347
 abbaa aabaa aabab aabaa baabb babbb abbbb bbaba bbaba abbba
 bbba- babab baaaa bb-bb bbaaa a-baa babbb bbaaa bbbaa baaab
 aabbb bbbbb aaaab babab baabb bbbaa bbbab aabbb babba bbbbb
 aabbb bbaaa baaba baaba bbaba aabaa bbbaa bbb
EACTGA204V          {0} ; 1361
 baaab babaa bbbbb abbaa abbbb abaaa abaab babbb babaa bbabb
 bbba- abbbb baabb bb-ab aaabb b-baa baaab ababb abbba bbbba
 abbba bbaba abaab babba aabaa aabbb baabb abbaa aabab bbaab
 bbabb babbb aaabb bbbba abbab bbabb babaa aab
EACTGA462V          {0} ; 1380
 bbbab aabaa aabab aabab baaba babab aaabb baaba bbaba aabba
 bbba- abaab aabaa bb-ab bbaab a-baa babba bbbaa bbbaa baaba
 abbbb babbb aaaab bbbbb baabb abbaa abbab aabbb babba babbb
 aabbb bbaaa baaba aabba bbaaa aabaa babaa abb
EACTGA463G          {0} ; 1381
 bbbab aabaa aabab aabab baaba babab aaaab baaba bbaba aabba
 bbba- abaab aabaa bb-ab bbaab a-baa babba bbbaa bbbaa baaba
 ababb babbb aaaab bbbbb baabb abbaa abbab aabbb babba babbb
```

```
 aabbb bbaaa baaba aabba bbaaa aabaa babaa abb
EAGTGC115V          {0} ; 1391
 abaaa aabaa aabbb aabaa baaab babbb abaab bbaaa bbaba abbba
 bbba- babab baaaa bb-bb bbaaa a-baa babbb bbaaa bbbaa babaa
 aabbb babbb aaaab babab baabb baaaa abbab aabbb babba babbb
 aaabb bbaaa baaba baaba bbaba aabaa babaa abb
EAGTGC186V          {0} ; 1396
 abbba bbabb aabba aabbb ababb bbbbb bbaaa abbbb bbaaa aabaa
 bbbb- bbbab bbaab ba-bb aaaba b-baa baaab bbaba bbbab baaab
 babba aaaaa aaabb bbbab baaab bbbaa ababa abaaa abbbb abbab
 bbbab aabaa aabab aaabb ababb bbaaa bbbaa abb
EAGTGC265G          {0} ; 1403
 ababa ababa aabab aabaa baaab abbab ababa bbaaa bbbbb abaab
 aabb- bbaaa aaaba aa-aa baaaa a-aab aaaaa abbaa aaaba abbbb
 bbaba ababa aaaaa ababb aaaaa aabbb babbb aaabb abaaa baaaa
 abbab abaab aabab babba bbbba ababb baaba aab
EAGTGC313G          {0} ; 1405
 abbaa aabaa aaaab aabaa baabb babbb abbab bbaba bbaba abbba
 bbba- babab baaaa bb-bb bbaaa a-baa babbb bbaaa bbbaa baaaa
 aaabb babbb aaaab babab baabb baaaa abbab aabbb babba babbb
 aaabb bbaaa baaba baaba bbaba aabaa babaa abb
EAGTGC388V          {0} ; 1410
 abbaa aabaa aaaab aabaa baabb babbb abbbb bbaba bbaba abbba
 bbba- babab baaaa bb-bb bbaaa a-baa babbb bbaaa bbbaa baaaa
 aabbb babbb aaaab babab baabb baaaa abbab aabbb babba babbb
 aaabb bbaaa baaba baaba bbaba aabaa babaa abb
EACTTC44V           {0} ; 1437
 abbaa aa-aa aabab -abaa baabb babbb abba- bbaba bbaba abbba
 bbbaa baabb baaaa bbbbb bb--a ---aa babbb bbaaa bbbaa baaaa
 aabbb babbb aaaab babab baabb baaaa abbab aabbb babba babbb
 aaabb bbaaa baaba baaba b-aba aabaa babaa abb
EACTTC256V          {0} ; 1461
 abbba ab-ab aabba -abbb babaa bbbbb abab- bbaba bbaaa abbba
 bbbaa bbabb bbaab bbbab ba--a ---aa babab bbaaa bbabb baaaa
 aabba abbaa aaaaa baaab bbaab babaa ababb aaaaa aabba babbb
 abaab baaaa baaaa aaaba a-bba aabaa babab abb
EACTTC272G          {0} ; 1463
 abbbb aa-aa aaaba -bbba babbb babbb bbaa- aabba bbaba abbaa
 bbbab aaaaa abbaa bbbab bb--b ---ba aabaa bbbaa bbbab aaaaa
 bbaba aaaaa baabb babab baaab ababb abbba aabaa aabba babbb
 abbba abaaa bbbaa aabab a-aba aabba bbbab bab
EACTTC418G          {0} ; 1474
 ababa ab-aa aaaba -bbaa bbbab aaaab aaaa- abaaa aabba ababb
 baaaa aaaba baaaa aaaaa ab--a ---aa aaaaa aabaa aaabb ababb
 bbabb aaaaa aabab baabb bbaba bbbab bbbba abaaa abaaa bbaab
 abaaa abaab bbbab abaab b-aab bbbbb babaa aab
EACTTC429G          {0} ; 1476
 baaab ba-aa bbbbb -baaa abbbb ababa bbaa- bbbbb babaa baaab
 bbbab abbbb babba bbbba aa--b ---aa baaab ababb abbaa bbbbb
 ababb baaba abaaa babba aabaa aabab bbaba abbaa aaaab bbbab
 abbbb aabbb aaabb bbbba a-aab bbbbb baaaa abb
EACTTC429V          {0} ; 1477
 baaab ba-aa bbbbb -baaa abbbb ababa bbaa- bbbbb babaa baaab
 bbbab abbbb babba bbbba aa--b ---aa baaab ababb abbaa bbbbb
 abbbb baaba abaaa babba aabaa aabab bbaba abbaa aaaab bbbab
 abbbb aabbb aaabb bbbba a-aab bbbbb baaaa abb
EACTTC451bG         {0} ; 1483
```

```
 bbbbb ba-aa aaaba -baba babbb babbb abaa- aabba bbbba abaaa
 bbbab aabaa abbaa bbbab bb--b ---ab aabaa abbba bbbab aaaaa
 bbaba baaab baaba aabab baaab ababb abbba abbaa aabba bbbba
 abbbb abaaa bbbaa abbbb b-aba abbba bbbab bbb
EACTTC484V           {0} ; 1485
 abbba aa-ab aabba -abab babaa babbb abbb- bbaba bbaba abbba
 bbbaa baabb bbaab bbbbb ba--a ---aa babbb bbaaa bbaba baaaa
 aabba babaa aaaab baaab baabb baaaa abaab aaaab aabba babbb
 aaaab baaaa baaaa baaba b-aba aabaa babab aba
EAGTAA67V            {0} ; 1498
 abbba bb-bb aabba -bbbb abab- bbbbb bbba- abb-b ab-aa a-baa
 -bbba bbbab b-aab bbabb aa--a ----a baa-a -b--- ----b babbb
 babba aaaaa ababb bbabb baaab bbbaa ababa abaaa abbbb abbab
 bbbab aabaa aabab a--b- a-abb bbaab bbbaa abb
ECGTGA187G           {0} ; 1538
 aaaba aa-aa aaaba -abab bbbab aabab aaab- abaaa abbba abaaa
 babba babaa aaaaa aabaa aa--a ---ab aaaaa aabaa aaabb abbbb
 bbaba aaaaa aaaaa baaab bbaab abbab babbb aabaa abaaa abaaa
 abaaa aabab bbbab abaab b-aaa aabba aaaba abb
SSR_39               {0} ; 1576
 ababb ababa aabab aabaa baaaa ab-ab ababa bbbaa bb-ba abbab
 aabba bbaaa ababa aaaba b-bba abaab aa--- -bbaa aaaba bbbba
 bbaba bbaba aaabb ababb aaaaa aabbb aabbb bbabb abaaa babaa
 abbab abaab aaaa- bbbba bbbba ababb baaab aab
SSR_756              {0} ; 1592
 babaa aabba bbaaa ababa aabaa baaab ababa abbab bbbba bbbaa
 bbabb aaaab abbaa bbbab ababa bbaab aabaa ababa aabbb baaaa
 bbbba babba bbaba abaaa bbaba bbbba abbba bbbba aabaa bbbbb
 aaaab aaaaa aaba- babab abbab bbbaa abbaa aa-
SSR_152              {0} ; 1620
 abbbb bbbaa aabba bbbba babbb baabb baaab aabba bbbba abbab
 bbaba abaaa abbaa bbbab bbabb abbba aabaa bbbaa bbbbb baaaa
 bbbba babab baabb babab baaab abaab abbba aabab aabba babbb
 aabba bbbaa bbbb- aabbb bbaba aabaa bbbab bab
SSR_874              {0} ; 1621
 ab-aa a-baa a--bb aabba baabb babbb abbab bbaba bbaba aabbb
 bbbba aabab baaaa bbbaa aaaab abaab babbb -bab- -bb-a baaaa
 aaabb aabbb aaba- babab baabb b---- ----- ----- ---ba babbb
 aaabb bbaaa baab- baaba bba-a aabaa babaa ab-

LG2:
EGAMGA80G            {0} ; 6
 abbab aaaab abbaa bbabb aabab aabba baaaa babbb aaaab bbaba
 babaa baaaa abbba bbaab bbaab babbb baabb baaaa baab- -----
 ----- ----- ----- ----- ----- ----- ----- ----- ----- -----
 ----- ----- ----- ----- ----- ----- ----- ---
EGAMGA215G           {0} ; 22
 bbaab abaab bbbbb abaab bbaba abbab aaabb aabbb aabab aabbb
 bbaaa babba baaab bbaba bbaaa bbbba babbb aaaaa abba- -----
 ----- ----- ----- ----- ----- ----- ----- ----- ----- -----
 ----- ----- ----- ----- ----- ----- ----- ---
EGAMGA299V           {0} ; 30
 abbab aaaab abbba bbabb aabab aabba baaaa babbb aaaab bbaba
 babaa baaaa abbba bbaaa bbaab baabb bbabb baaaa baab- -----
 ----- ----- ----- ----- ----- ----- ----- ----- ----- -----
 ----- ----- ----- ----- ----- ----- ----- ---
EGAMGA300V           {0} ; 31
```

```
 bbaab abbaa babaa aaaab bbaab abbaa aaabb abbba aaaab bbabb
 babbb bbbab abaab bbaaa babaa bbbba bbaba abaab abbb- -----
 ----- ----- ----- ----- ----- ----- ----- ----- ----- -----
 ----- ----- ----- ----- ----- ----- ----- ---
ECAMCG264G           {0} ; 75
 abbab aaaab abbaa bbabb abbab aabba baaba babbb aaaab bbaba
 babaa baaaa abbba bbaaa bbaab baabb baabb baaaa baab- -----
 ----- ----- ----- ----- ----- ----- ----- ----- ----- -----
 ----- ----- ----- ----- ----- ----- ----- ---
EGAMGC204V           {0} ; 119
 abbbb aaaab abbaa bbabb abbab aabba baabb babbb aaaab bbaba
 babaa baaaa abbba bbaaa bbaab baabb baabb baaaa baabb baaba
 aabab aabbb aaabb aabab abbbb babaa abbbb abbba bbaba bbaab
 abbbb babaa abaaa baabb bbbbb abbbb abaaa bbb
EGAMGC205G           {0} ; 120
 abbbb aaaab abbaa bbabb abbab aabba baabb babbb aaaab bbaba
 babaa baaaa abbba bbaaa bbaab baabb baabb baaaa baabb baaba
 aabab aabbb aaabb aabab abbbb babaa abbbb abbba bbaba bbaab
 abbbb babaa abaaa baaba bbbbb abbbb abaaa bbb
ECAMCC161G           {0} ; 147
 bbaab abbaa babba aaaab baaab abbab aaabb abbba aaaab bbbbb
 babbb bbbaa baaab bbaaa babaa bbbba babbb abaab -bbbb bbabb
 babba bbbba baaab abaaa abbba aabbb abbab bbbbb abbaa bbbab
 abbba babaa abbaa baaaa bbbba baaaa abbaa baa
ECAMCC162V           {0} ; 148
 bbabb abbaa babba aaaab baaab abbab aaabb bbbba aabab bbbbb
 babbb bbbaa baaab bbaaa babaa bbbba babbb abaab -bbbb bbabb
 babba bbbba baaab abaaa abbba aabbb abbab bbbbb abbba bbbab
 abbba babaa abbaa baaab bbbba baaaa abbaa baa
ECAMCC447G           {0} ; 172
 abaab aabab abbaa ababb aabab aabba baaba bbbbb aabbb bbaba
 babaa babaa ababa bbaaa abaab bbabb baabb bbaaa -aabb baaba
 aaaab aaaaa aaabb bbabb abbab bbbaa ababb abaaa bbaaa bbaab
 ababb babaa abaaa baaba bbaab abaab abbaa baa
EACMCC499G           {0} ; 236
 bbbab ababb bbabb abaab aabba abbab aaaba aabbb abbab aabbb
 bbaaa bbbaa abbaa baaab baaaa bbbba abbbb abbab abbaa abbbb
 bbbaa abbbb aaaab aabaa abaab aabab bbbab babbb abbaa aaabb
 bbbbb bbbab abbba babaa bbbab baaaa aabaa bba
ECAMGG115G           {0} ; 248
 bbbab abbab bbaba aaaab baaab abbab aaabb aabba aabab babbb
 bbbaa bbbaa abaab babaa bbbaa bbbba babbb abaab abbab babbb
 aabba abbba baaab abbaa abbbb aabbb abbab bbbba abbaa abbbb
 bbbba babab abbab baaaa bbbab baaaa abbaa baa
ECAMGG188V           {0} ; 257
 abaaa aabab abbaa ababb aabab aabba ababa babbb aaabb bbaba
 babaa babaa babab abbaa abaab baabb baabb bbaaa baabb baaba
 aaaab aaaaa aaabb bbabb abbab bbbaa ababb abaaa bbaaa bbaab
 ababb babaa abaaa baabb bbaab abaab abbaa bba
ECAMGG326G           {0} ; 272
 abaaa aaaab abbaa ababb aabab aabba abaaa babbb aaabb bbaba
 babaa babaa babab aabaa abaab bbabb baabb bbaaa baabb baaba
 aaaab aaaaa aaabb bbabb abbab bbbaa ababb abaaa bbaaa bbaab
 ababb babaa abaaa baaba bbaab abaab abbaa bba
ECAMGG358V           {0} ; 277
 abbab aaaab abbaa bbabb abbab aabba bbaba babbb aaaab bbaba
 babaa baaaa babbb aabaa bbaab baabb baabb baaaa baabb baaaa
```

```
 aabab aabbb aaaba aabab abbbb babaa abbbb abbaa bbaaa baaab
 abbbb babab aaaaa baabb bbbbb abbbb abaaa bbb
ECGMTA80V           {0} ; 301
 bbbab baaab abbab bbaab aaaab bbaba aabba bbabb baaaa bbaba
 bbbab bbaaa bbbbb abaaa bbbaa bbaba aaabb babba baabb bbaba
 aabba abbba aaabb abbab bbbba babba ababb abbba bbaaa baaba
 abbab bbbab bbaaa baaab bbbab abaab ababa aba
ECGMTA135G          {0} ; 305
 bbaab bbbaa babba baaab aaaaa aaaba aaabb bbbaa aaaaa bbaab
 aabba bbbab baaaa abaaa aabaa bbaba aaaba bbaaa ababb bbabb
 bbaba bbbba baaab abaab abbaa aabbb abaaa bbbbb abbaa bbabb
 abbbb aabaa bbbaa baaba babbb bbaab bbaab bba
ECGMTA256V          {0} ; 314
 bbbab ababb bbabb abaab abbba abbab aaabb aabbb aabab aabbb
 bbaaa babba abaab babab baaaa bbbba bbbbb bbbbb abbab abbbb
 abbaa abbba baaab aabaa abaab aabbb abbab bbbba abbaa aabbb
 bbbba babab aabbb baaab bbbab baaaa abbaa baa
ECGMTA273G          {0} ; 316
 abbab aaaab abbaa bbabb abbab aabba abaaa babbb aaaab bbaba
 babaa baaaa babbb aabaa bbaab baaab baabb baaaa baabb baaba
 aabab aabbb aaabb aabab abbbb babaa abbbb abbba bbaaa bbaab
 abbbb babab aaaaa baaba bbbbb abbbb abaaa bbb
ECGMTA446V          {0} ; 327
 bbbab aaaab bbbab bbabb aabab bbbba abbba babbb baaaa bbaba
 babab bbaaa bbbbb abbaa bbbab baabb baabb babaa baabb bbaba
 aabab ababa aaaba abbab abaaa babaa aabbb abbaa bbaaa baabb
 abbaa bbbab aabaa baaab aabab abaaa abaaa bba
ECGMTA580G          {0} ; 334
 abbab aaaab abbaa bbabb aabab aabba ababa bbabb aaaab bbaba
 babaa baaaa babbb aabaa bbaab baaab baabb baaaa baabb baaba
 aabab aabbb aaabb aabab abbbb babaa abbbb abbba bbaaa bbaab
 abbbb babab aaaaa baaba bbbbb abbbb abaaa bbb
EACMGA107G          {0} ; 339
 abbbb aabab abbab bbabb aabbb aabba bbaba babbb baabb bbbba
 babab babaa ababa bbaba abaab bbaab baabb bbaaa baabb baaba
 aaaa- aaaaa aaaab bbabb abbab bbbaa ababb abaaa bbaaa bbaab
 ababb babaa abaaa baaba baaab abaab abbaa bba
EACMGA254V          {0} ; 355
 abaaa aaaab abaab bbaab aabab bbaba aaaba bbabb baaaa bbaba
 abbab baaaa bbbba babaa bbbaa bbaba aaabb aabaa baabb bbaba
 aabb- abbba aaabb abbab bbbbb babba abaab aaaba abaaa baaba
 abbab abbab baaaa baaab bbbab bbaab ababa bba
EACMGA257G          {0} ; 356
 abbbb aaaab abbab bbaab aabbb bbaba aabba bbabb baaaa bbbba
 abbab baaaa bbbba babaa bbbaa bbaba aaabb aabaa baabb bbaba
 aabb- abbba aaabb abbab bbbbb babba ababb bbbba bbaaa baaba
 abbab abbab baaaa baaaa bbbab bbaab ababa bba
EACMGA477V          {0} ; 382
 abbbb aaaab abbaa bbabb abbab aabba baaba babbb aaaab bbaba
 babaa baaaa abbba bbaaa bbaab baabb baabb baaaa baabb baaba
 aaba- aabbb aaabb aabab abbbb babaa abbbb abbba bbaaa bbaab
 abbbb babab abaaa baabb bbbbb abbbb abaaa bbb
EACMTC178V          {0} ; 468
 bbbab abbbb bbaba aaaab baaab abaab aaabb aabba aabab babbb
 bbbaa bbbab baaab bbaaa bbba- bbbba babbb abaab abbab babbb
 aabba abbba baaab abbaa abbbb aabbb abbab bbbba abaaa bbbbb
 bbbba babab abbab baabb bbbab baaaa abbaa baa
```

```
EACMTC178G           {0} ; 469
 bbbab abbbb bbaba aaaab baaab abaab aaabb aabba aabab babbb
 bbbaa bbbab baaab bbaaa bbba- bbbba babbb abaab abbab babbb
 aabba abbba baaab abbaa abbbb aabbb abbab bbbba abaaa bbbbb
 bbbba babab abbab baaab bbbab baaaa abbaa baa
EACMTC227G           {0} ; 481
 bbaab bbbaa babba aaaab aaaab bbbba aaabb bbbba aaaaa bbabb
 babbb bbbab baaab bbaaa baba- bbaba baaba bbaaa ababb bbabb
 bbaba bbbba baaab abbaa abbba aabbb abbab bbbbb abbaa bbabb
 abbbb babaa abbaa baaab bbbba baaaa abbab baa
EACMTC418V           {0} ; 515
 bbbab ababb bbabb abaab aabba abaab aaabb aabbb aabab aabbb
 bbaaa babba baaab bbaba baaa- bbbba bbbbb abbab abbaa abbbb
 abbaa abbba baaab aabaa abaab aabbb abbab bbbba abbaa aabbb
 bbbba babab abbbb baaba bbbab baaaa abbaa baa
ECAMCT127G           {0} ; 550
 bbaab bbbaa babba baaab aaaab bbaba aaabb bbbba aaaaa bbabb
 babbb bbbab aaaab abaaa baba- baaba baaba bbaaa ababb bbabb
 bbaba bbbba baaab abaab abbaa babbb abaaa bbbbb abbaa bbabb
 abbbb aabaa bbbaa baaab bbbba aabba abaab bba
ECAMCT128V           {0} ; 551
 bbaab bbbaa babba baaab aaaab bbaba aaabb bbbba aaaaa bbabb
 babbb bbbab aaaab abaaa baba- baaba baaba bbaaa ababb bbabb
 bbaba bbbba baaab abaab abbaa babbb abaaa bbbbb abbaa aaabb
 abbbb aabaa bbbaa baabb bbbba aabba abaab bba
ECAMCT185V           {0} ; 559
 abbab aaaab aabaa bbabb abbab aabba baaba babbb aaaab bbaba
 babaa baaaa abbba bbaaa bbaa- baabb baabb baaaa baabb baaba
 aabab aabbb aaabb aabab abbbb babaa abbbb abbba bbaaa bbaab
 abbbb babab abaaa baabb bbbbb abbbb ababa abb
ECAMCT290V           {0} ; 573
 bbaaa abbaa baaba aaaab baaab abbab aaabb abbba aaaab bbbbb
 babbb bbbaa baaab bbaaa baba- bbbba baaab abaab abbbb bbabb
 babba bbbba baaab abaaa abbba aabbb abbab bbbbb abbaa bbbab
 abbba babaa abbaa baabb bbbba baaaa abbaa baa
ECAMCT309V           {0} ; 577
 bbaaa abaaa babba aaaab aaaab abbab aaabb aabba aaaab bbbbb
 babbb bbbaa baaab bbaaa baba- bbbba babbb abaab abbbb bbabb
 babba bbbba baaab abaaa abbba aabbb abbab bbbbb abbaa bbbab
 abbba babaa abbab baabb abbba baaaa abbaa baa
ECAMCT353G           {0} ; 580
 abbab aaaab abbaa bbabb abbab aabba aaaba bbbab aabab ababa
 babaa baaaa abbba bbaaa bbaa- baabb baabb baaaa baabb baaba
 aabab aabbb aaabb aabab abbbb babaa abbbb abbba bbaaa bbaab
 abbbb babab abaaa baaab bbabb abbbb abaaa bbb
ECGMTT78G            {0} ; 596
 aaaaa baaab aba-a bbabb abbab aabba abaaa aabaa aaaab bbaba
 babaa baaaa abbba bbaaa bba-- baaab baabb baaaa aaabb baaba
 aabab aabbb aaabb aabaa abbbb babaa abbbb abbaa bbaaa bbaab
 abbbb babaa abaaa baabb bbbbb abbbb abaaa bbb
ECGMTT195V           {0} ; 606
 bbbab ababb bba-b ababb aabba abbab aaabb aabbb aabab aabbb
 bbaaa babba baaab bbaba baa-- bbbba bbbbb bbbab abbab abbbb
 abbaa abbba baaab aabaa abaab aabbb abbab bbbba abbaa aabbb
 bbbba babab abbbb babab bbbab baaaa abbaa baa
ECGMTT205G           {0} ; 608
 bbaab babaa aab-a baaab aaaab baaba aaabb bbbba aaaaa bbabb
```

```
 babbb bbbab abaab abaaa bab-- baaaa baaba abaaa aaaba abbbb
 bbaba bbbba aaaab abaab abbaa babbb abaaa bbbbb abbaa bbabb
 abbbb aabaa bbbaa baaab bbbba aabba abaab bba
ECGMTT272V          {0} ; 614
 bbaab bbbaa aab-a baaab aaaab bbaba aaabb bbbba aaaaa bbabb
 babbb bbbab aaaab abaaa bab-- baaba baaba bbaaa ababb bbbbb
 bbaba bbbba babab abaab abbaa babbb abbaa bbbbb abbaa bbabb
 abbbb aabaa bbbaa babab bbbba aabba abaab bba
EAGMGG125G          {0} ; 670
 abbab aaaab abbaa bbabb abbab aabba baaba babbb aaaab bbaba
 babaa baaaa abbba bbaaa bbaab baabb baabb baaaa baabb baaba
 aabab aabbb aaabb aabab abbbb babaa abbab abbba abaaa bbaab
 abbbb babab abaaa baaba bbbbb abbbb abaaa bbb
EAGMGG127V          {0} ; 671
 abbab aaaab abbaa bbabb abbab aabba baaba babbb aaaab bbaba
 babaa baaaa abbba bbaaa bbaab baabb baabb baaaa baabb baaba
 aabab aabbb aaabb aabab abbbb babaa abbab abbba abaaa bbaab
 abbbb babab abaaa baabb bbbbb abbbb abaaa bbb
EAGMGG228G          {0} ; 686
 bbbab baaaa babba baaab aaabb baaba aaabb bbbba baaaa bbabb
 babbb bbbab abaaa abaaa bbbaa baabb baaba bbaaa ababb bbabb
 bbaba bbaba baaab abaaa abbaa babbb abaab bbbbb abaaa bbabb
 abbab aabaa bbbaa baaaa bbbba abbba abaab aba
EAGMGG485G          {0} ; 700
 abaab aaaab abbaa ababb abbab aabba baaaa babbb aaabb bbaba
 babaa babaa ababa bbaaa abaab bbabb baabb bbaaa baabb baaba
 aaaab aaaaa aaabb bbabb abbab bbbaa ababb abaaa bbaaa bbaab
 ababb babaa abaaa baaba bbaab abaab abbaa bba
ECGMGC114V          {0} ; 710
 abbab aaaab abbaa bbaab abbab bbaba abbba baabb baaaa bbaba
 aabab baaaa bbbba bbbaa bbbab baabb aaabb babaa baabb baaba
 aabab aabbb aaabb aabab bbbbb babaa abbbb abbba bbaaa bbaab
 abbab abbab abaaa baabb bbbbb bbbbb ababa bbb
ECGMGC552V          {0} ; 728
 bbbab abbbb bbaba aaaab bbaab abbab aaabb aabba aabab babbb
 bbbaa bbbaa baaab bbaaa bbbaa babba babba abaab aabab babbb
 aabba abbba baaab abbaa abbbb aabbb abbab bbbba abbaa bbbbb
 bbbba babab abbab baaab abbab baaaa abbaa baa
EACMGG144G          {0} ; 747
 bbbab abbbb bbaba aaaab baaab abbab aaabb aabba aabab b-bbb
 bbbaa bbbaa baaab bbaaa bbbaa bbbba babbb abaab abbab babbb
 aabba ababa baaab abbaa abbbb aabbb a-bab bbbba abbaa bbbbb
 bbbba babab abbab baaab bbbab baaaa abbaa baa
EACMGG206V          {0} ; 760
 abbab aaaab abbaa bbabb abbab aabba baaba babbb aaaab b-aba
 babaa baaaa abbba bbaaa bbaab baabb baabb baaaa baabb baaba
 aabab aabbb aaabb aabab abbbb babaa a-bbb abbba bbaaa bbaab
 abbbb babab abaaa baabb bbbbb abbbb abaaa bbb
EACMGG270G          {0} ; 769
 bbbab ababb bbabb abaab aabba abbab aaaba aabbb abbab a-bbb
 bbaaa bbbaa baabb aaaba baaaa bbbba bbbbb abbab abbaa abbbb
 bbbaa abbbb aaaab aabaa abaab aabab b-bab babbb abbaa aaabb
 bbbbb babab abbba babaa bbbab baaaa aabaa bba
EACMGG316V          {0} ; 772
 bbaab bbbaa babba baaab abaab baaba aaabb bbbba aaaaa b-abb
 babbb bbbab abaab abaaa baaaa baaba baaba bbaaa ababb bbabb
 bbaba bbbba baaab abaab abbaa babbb a-aaa bbbbb abbaa bbabb
```

abbbb abbaa bbbaa baabb bbbba aabba abaab bba
EACMGG474G {0} ; 792
abaab aabab abbaa ababb aabab aabba baaaa babbb aabbb b-aba
babaa babaa ababa bbaaa abaab bbabb baabb bbaaa baabb baaba
aaaab aaaaa aaabb bbabb abbab bbbaa a-abb abaaa bbaaa bbaab
ababb babab abaaa baaab bbaab abaab abbaa bba
EAGMCT322V {0} ; 829
abbab aaaab abbaa baabb abbab aabba ababa babbb aaaab b-aba
babaa baaaa abbba bbaaa bbaab baabb baabb baaaa baabb baaba
aabab aabbb aaabb aabab abbbb babaa a-bbb abbba bbaaa bbaab
abbbb babab abaaa baabb bbbbb abbbb abaaa bbb
ECGMGT93G {0} ; 848
abbab aaaab abbaa bbabb aabab aabba ababa babbb aaaab b-aba
babaa baaaa abbba bbaaa bbaab baabb baabb baaaa baabb baaba
aabab aabbb aaabb aabab abbbb babaa a-bbb abbba bbaaa bbbab
abbbb babab abaaa baaab bbbbb abbbb abaaa bbb
EACMGC80V {0} ; 870
abbaa aaaab ababa ababb abbab aabba baaba babbb aaabb bbaba
babaa babaa abbba bbaaa abaab bbabb baabb bbaaa baabb baaba
aaaab aaaba aaabb baabb abbab bbbaa ababb abaaa abaab bbbab
babaa babba abbbb baaab baaba babbb abaaa baa
EACMGC82V {0} ; 871
bbaab aaaab ababb bbaab aabab bbbba aabba bbabb baaaa bbaba
abbab baaaa bbbba babaa bbbaa bbaba aaabb aabaa baabb bbaba
aabba abbba aaabb abbab bbbbb babba abaaa abbba abaaa bbbba
bbbaa babab abbaa aaaab aaaaa bbaba bbabb baa
EACMGC91V {0} ; 874
abbab aaaab ababa bbabb abbab aabba baaba babbb aaaab bbaba
babaa baaaa abbba bbaaa bbaab baabb baabb baaaa baabb baaba
aabab aabbb aaabb aabab abbbb babaa abbbb abbba abaab bbbbb
babbb babaa abbbb baaab baaba bbbbb ababa baa
EAGMCA138G {0} ; 942
bbaab bbbaa babba baaab aaaab baaba aaabb bbbba baaaa bbabb
babbb bbbab abaab abaaa bbbaa baabb baaba bbaaa ababb bbabb
bbaba bbbba baaab abaab abbaa babbb abaaa bbbbb abaaa bbbbb
aabbb aabaa bbbaa bbaaa babba bbbba abaab bba
EAGMCA230V {0} ; 952
abbab aaaab ababa bbaab abbab bbaba aabba baabb baaaa bbaba
aabab baaaa bbbba bbbaa bbbab bbabb aaabb aabaa baabb bbaba
aabbb abbbb aaabb aabab bbbbb babaa ababb abbba abaaa bbbbb
bbbaa babab abbab baaab babaa bbaba bbabb baa
EAGMCA232V {0} ; 953
abbab aaaab ababa bbaab abbab bbaba aabba baabb baaaa bbaba
aabab baaaa bbbba bbbaa bbbab bbabb baabb aabaa baabb bbaba
aabbb abbbb aaabb aabab bbbbb babaa ababb abbba abaaa bbbbb
bbbaa babab abbab baaab babaa bbaba bbabb baa
EAGMCA234G {0} ; 954
abbab aaaab ababa bbaab abbab bbaba aabba baabb baaaa bbaba
aabab baaaa bbbba bbbaa bbbab bbabb aaabb aabaa baabb bbaba
aabbb abbbb aaabb aabab bbbbb babaa ababb abbba abaaa bbbbb
bbbaa babab abbab baaaa babaa bbaba bbabb baa
EAGMCA260G {0} ; 958
abaab aabab ababa ababb aabab aabba ababa babbb aabbb bbaba
babaa babaa ababa bbaaa abaab bbabb baabb bbaaa baabb bbaba
bbbab aaaaa aaabb bbabb abbab bbbaa ababb abaaa abaab bbbaa
babaa babba abbab baaaa baaba babbb abaaa baa
ECGMCA81G {0} ; 978

```
 abaab aabab ababa ababb aabab aabba baaba babbb aabbb bbaba
 babaa babaa ababa bbaaa abaab bbabb baabb bbaaa baabb baaba
 aaaab aaaaa aaabb bbabb abbab bbbaa ababb abaaa abaab bbbaa
 babaa babba abbab baaaa baaba babbb abaaa baa
EACMGT70G           {0} ; 1004
 abaab aabab abbaa ababb aabab aabba baaba babbb aabbb bbaba
 babaa babaa ababa bbaaa abaab bbabb baabb bbaaa baabb baaba
 aaaab aaaaa aaabb bbabb abbab bbbaa ababb abaaa bbaaa bbaab
 ababb babaa abaaa baaba bbaab abaab abbaa bba
EACMGT74V           {0} ; 1005
 bbbab baaaa bbbab bbaab aaaab baaba aabbb bbabb baaaa bbaba
 bbbab bbbaa bbbba bbbaa bbbaa bbaba aaabb babaa baabb bbaba
 aabbb ababa aaabb abbab bbbba babba ababb abbbb bbaaa baabb
 abbab bbbab bbbaa baaab bbbab abaab ababa aba
EACMGT373G          {0} ; 1034
 abbab aaaab abbaa bbaab abbab abbba babaa babbb baaab bbaba
 babab baaaa bbbba bbaaa bbbab baabb aaabb babaa baabb baaba
 aabab aabbb aaabb aabab bbbbb babaa abbbb abbba bbaaa bbaab
 abbab abbab abaaa baaba bbbbb bbbbb ababa bbb
EAGMTT333V          {0} ; 1070
 abbab baaab abbaa bbabb abbab aabaa baaba baabb aabab bbaba
 baaab baaab abbba bbaaa bbaab baabb baaba baaaa baabb baaba
 aabab aabbb aaabb aabab ababb babaa abbbb abbba bbaaa bbaab
 abbbb babab abaaa baabb bbbbb abbab abaaa bbb
ECGMGG556G          {0} ; 1112
 abbab aaaab abbaa bbabb aabab aabba abaaa babbb aaaab bbaba
 babaa baaaa abbba bbaaa bbaab baabb baabb baaaa baabb baa-b
 aabaa aabbb aaabb aabab abbbb babaa abbbb abbba bbaaa bbaab
 abbbb babab abaaa baaba bbbbb abbbb abaaa bbb
EACTCC291V          {0} ; 1135
 bbbab baaaa bbbab bbaab aaaab bbaba aabbb bbabb baaaa bbaba
 bbabb bbbaa bbbba aabaa bbbaa bbaba aaabb babaa baabb bbaba
 aabbb ababa aaabb abbab bbbba babba ababb abbbb bbaaa baabb
 abbab bbbab bbbaa baaab bbbab abaab ababa aba
EACTCC294G          {0} ; 1136
 bbbab baaaa bbbab bbaab aaaab bbaba aabbb bbabb baaaa bbaba
 bbabb bbbaa bbbba aabaa bbbaa bbaba aaabb babaa baabb bbaba
 aaabb ababa aaabb abbab bbbba babba ababb abbbb bbaaa baabb
 abbab bbbab bbbaa baaab bbbab abaab ababa aba
EACTAG151G          {0} ; 1238
 bbaab baaaa abbab bbaab aaaab bbaba aabbb bbabb baaaa bbaba
 bbabb bbbaa bbbba aabaa bbbaa bbaab aaabb babaa baabb bbaba
 aabbb ababa aaabb abbab bbbb- babba ababb abbbb bbaaa baaba
 abbab bbbab bbbaa baaab bbbab abaab ababa aba
EACTAG152V          {0} ; 1239
 bbbab baaaa abbbb bbaab aaaab bbaba aabbb bbabb baaaa bbaba
 bbabb bbbaa bbbba aabaa bbbaa bbaab aaabb babaa baabb bbaba
 abbbb ababa aaabb abbab bbbb- babba ababb abbbb bbaaa baaba
 abbab bbbab bbbaa baaab bbbab abaab ababa aba
EACTAG243G          {0} ; 1249
 bbbab baaaa bbbab bbaab aaaab bbaba aabbb bbabb baaaa bbaba
 bbabb bbbaa bbbba aabaa bbbaa bbaab aaabb babaa baabb bbaba
 aabbb ababa aaabb abbab bbbb- babba ababb abbbb bbaaa baaba
 abbab bbbab bbbaa baaab bbbab abaab ababa aba
EAGTCA252G          {0} ; 1303
 bbaab bbbaa babba baaab aaaab bbaba aaabb bbbba aaaaa bbabb
 babbb bbbab aaaab abaaa babaa baaba baaba bbaaa ababb bbabb
```

```
 baaba bbbba baaab abaab abba- babbb abaaa bbbbb abbaa bbabb
 abbbb aabaa bbbaa baaab bbbba babaa abbab bba
EAGTCA255V          {0} ; 1304
 bbaab bbbaa babba baaab aaaab bbaba aaabb bbbba aaaaa bbabb
 babbb bbbab aaaab abaaa babaa baaba baaba bbaaa ababb bbabb
 bbaba bbbba baaab abaab abba- babbb abaaa bbbbb abbaa bbabb
 abbbb aabaa bbbaa baaab bbbba babaa abbab bba
EAGTGC219V          {0} ; 1398
 abbab aaaab abbaa bbabb abbab aabba baaba babbb aaaab bbaba
 baab- baaaa abbba bb-aa bbaab b-abb baabb baaaa baabb baaba
 aabab aabbb aaabb aabab abbbb bbbaa abbbb abbba bbaaa bbaab
 abbbb babab abaaa baabb bbbbb abbbb abaaa bbb
ECGTCA157V          {0} ; 1422
 bbaab bbbaa babba baaab aaaab bbaba aaabb bbbba aaaaa bbabb
 babb- bbbab aaaab ab-aa babaa b-aba baaba bbaab ababb bbabb
 bbbba bbbba baaab abaab abbaa babbb abaaa bbbbb bbbaa bbabb
 abbbb aabaa bbbaa baaab bbbba babaa abbab bba
ECGTCA160G          {0} ; 1423
 bbaab bbbaa babba baaab aaaab bbaba aaabb bbbba aaaaa bbabb
 babb- bbbab aaaab ab-aa babaa b-aba baaba bbaaa ababb bbabb
 bbaba bbbba baaab abaab abbaa babbb abaaa bbbbb abbaa bbabb
 abbbb aabaa bbbaa baaab bbbba babaa abbab bba
EACTTC241G          {0} ; 1459
 bbbab ab-bb bbabb -aaab baaba abbab aaab- aabbb aabab aabbb
 bbaaa babba baaab bbaba bb--a ---ba babbb abbab abbaa bbbbb
 aaaaa abbba baaab aabaa abaab aabbb abbab b-bba abbaa aabbb
 bbbba babab abbbb baaab b-bab baaaa abbaa baa
EACTTC448V          {0} ; 1481
 bbaab bb-aa babba -baab aaaab baaba aaab- bbbba baaaa bbabb
 babbb bbabb abaab abaaa bb--a ---bb baaba bbaaa ababb bbabb
 bbbba bbbba baaab abaab abbaa babbb abaaa bbbbb abaaa bbaab
 abbbb aabaa bbbaa baaab b-bba abbaa abaab aba
ECGTGA339V          {0} ; 1555
 bbbab ba-ab abbab -baaa aaabb bbaaa aabb- bbabb baaaa bbaba
 bbabb bbaaa bbbba babaa bb--a ---ba aaabb babaa baabb bbaba
 aabba abbba aaabb abbab bbbba babba ababb abbba bbaaa baaba
 abbab bbbab bbaaa baaab b-bab abaab ababa aba
ECGTGA430G          {0} ; 1562
 bbbab ba-aa abbab -baaa aaaab bbaba aabb- bbabb baaaa bbaba
 bbabb bbbaa bbbba aabaa bb--a ---ba aaabb babaa baabb bbaba
 aaabb ababa aaabb abbab bbbba babba ababb abbbb bbaaa baaba
 abbab bbbab bbbaa baaab b-bab abaab ababa aba
SSR_490             {0} ; 1585
 abbbb -bbbb abb-a bbaab abbaa baaaa b---a baabb aaaba bbaba
 babab bab-- ----- ----- ----- ----- ----- ----- ----b baaba
 aaba- ----- ----- aabab abb-b babaa abbbb abbba bbaaa bbaab
 abaaa babab aba-- baaaa ----- ----- ----- ---

LG3:
EGAMGA105V          {0} ; 10
 bbbba bbbba abbaa ababb bbabb baaba ababb abbbb baabb aaaaa
 abaab abbab babaa babaa baabb baaba bbaaa babba baaa- -----
 ----- ----- ----- ----- ----- ----- ----- ----- ----- -----
 ----- ----- ----- ----- ----- ----- ----- ---
EGAMGA161V          {0} ; 16
 bbbba bbbba abbaa ababb baabb baaba ababb abbbb baaba aaaaa
 abaab abbab babaa babaa baabb baaba bbaaa babba baaa- -----
```

```
 ----- ----- ----- ----- ----- ----- ----- ----- ----- -----
 ----- ----- ----- ----- ----- ----- ----- ---
ECAMCG141G          {0} ; 62
 aabba bbbaa bbbab bbabb abaab baaab abbab abbbb baabb aaaaa
 abaab abbaa abbba bbbab baabb bbaba baaaa babba baab- -----
 ----- ----- ----- ----- ----- ----- ----- ----- ----- -----
 ----- ----- ----- ----- ----- ----- ----- ---
ECAMCG166V          {0} ; 65
 bbbba aabba aabaa ababb bbaba aaaaa aaaab aabbb baaaa aaaba
 aaaab aabab babab baaaa bbbbb aaaba bbaaa babbb aaab- -----
 ----- ----- ----- ----- ----- ----- ----- ----- ----- -----
 ----- ----- ----- ----- ----- ----- ----- ---
EGAMGC81G           {0} ; 104
 bbbba bbbba abaaa ababb baabb baaba ababb abbab baaba aaaaa
 abaab abbab babaa baaaa baabb baaba baaaa babba bbbab abbba
 babaa babaa abaaa aaabb babba babba aaaaa baaaa baabb abaaa
 babbb bbaab bbbaa bbbaa babbb baaab aaaab baa
EGAMGC166G          {0} ; 115
 aabaa babab babba abbba bbaab babab aabaa bbaba abaab baabb
 bbaaa ababa abbba bbbba aabab bbaba baaab aaabb abbba bbbaa
 ababa ababb bbbbb aabbb ababb bbaba bbaba baabb abbba ababb
 bbaab aabaa ababa bbaaa baaab baaab abbbb bbb
EACMCC85V           {0} ; 194
 aaaba babab babbb abbba bbaab babab aabba bbaba abaab baaab
 bbaaa ababa babbb abbab aabab bbaba baabb aaabb abbba bbbab
 ababa ababb bbbbb aabbb ababb baaba bbaba aaabb abbaa ababb
 bbaab aabab ababa bbaab baabb baaab aabbb bbb
EACMCC154G          {0} ; 203
 aabba bbbaa bbbab bbabb abaab baaab babab abbbb baabb aaaaa
 abaab abbaa babbb abbba baabb bbaba aaaaa babba baabb abbba
 babaa aaaaa bbaaa aaaab babab baaaa ababa baaaa baabb abbaa
 baabb aaaab abbaa bbbaa babbb baaab babaa bbb
EACMCC234V          {0} ; 216
 aabbb abaab aabbb babab aaaab baaab aabbb ababa aaaba bbbba
 baaaa aaaab bbbba babaa aaaba bbbab aabab aaabb abaaa baabb
 bbaba abbbb bbbba abaab abbbb babba babbb aabab bbaaa abbaa
 babab aaabb baaab abbbb bbbaa baaaa babba bbb
EACMCC235G          {0} ; 217
 aabbb abaab aabbb babab aaaab baaab aabbb ababa aaaba bbbba
 baaaa aaaab bbbba babaa aaaba bbbab aabab aaaba abaaa baabb
 bbaba ababb bbbba abaab abbbb babba babbb aabab bbaaa abbaa
 babab aaabb baaab abbba bbbaa baaaa babba bbb
EACMCC464G          {0} ; 233
 bbbba aabba abbaa ababb bbaba aaaba baaab abbbb baaaa aaaaa
 abaab abbab ababb aaaaa baabb baaba bbaaa babbb baaab abbba
 baaaa babab aaaba aaaab baaba aabba aaaaa bbaaa baabb aaaba
 aaabb bbaab bbaaa aabaa babbb baaab aaaab baa
ECAMGG380G          {0} ; 279
 aaaba bbbaa abbbb babbb abaaa bbbab aabaa bbabb baabb bbaab
 baaaa bbaba aabab bbbbb baabb bbaba baabb aaabb abbba bbbab
 aaaba ababb bbabb aaaaa aaaab baaaa bbaaa baaaa abaaa bbbaa
 abaab aabbb abbaa bbbaa aabbb baaab aabaa baa
ECAMGG490V          {0} ; 292
 bbbbb abbbb abbaa ababb bbaba aaaba aaaab bbbbb aaaaa aaaba
 aaaaa aabab baaba baaaa babab aaaba baaaa aabbb baabb aabbb
 baaaa aaabb aaaba abaab aaaba aabba aabaa bbbaa aaabb aaaba
 ababb bbaab bbaaa aabab babbb babbb aaaab bab
```

```
ECAMGG508G           {0} ; 295
 bbbbb aabbb abbba ababb bbaba aaaba aaaab bbbbb aaaaa aaaba
 aaaaa aabab baaba baaaa babab aaaba baaaa aabbb bbabb aabba
 baaaa aaabb aaaba abaab aaaba aabba aabaa bbbaa aaabb ababa
 ababb bbaab bbbaa aabaa babbb babbb aaaab bab
ECGMTA166G           {0} ; 309
 aaaba aabab babba abbaa bbaab babab abbaa bbaba abaab baabb
 bbaaa ababa babbb abbab aabab bbaaa baaab aaaaa abbba bbbaa
 ababa ababb bbbba aabbb ababb bbbba bbaba baabb abbaa ababb
 bbbab aabab aaaba bbaaa baabb baaab abbbb bbb
EACMGA420V           {0} ; 375
 aaaaa bbbaa abbbb babbb abaaa bbbab aaaaa bbabb baabb bbaab
 baaaa bbaba aaabb babbb baabb bbaba baabb aaabb abbba bbbab
 aaab- ababb bbabb aaaaa aaaab baaaa bbaaa baaaa abaaa bbbaa
 abaab aabbb aabaa bbbab aabbb baaab aabaa baa
EAGMTA202G           {0} ; 400
 babba bbaaa bbbab bbabb abaab baaab abbab abbbb baabb aaaaa
 abaab abbaa abbba b-bab baabb baaba baaaa babba baabb abbba
 baba- aaaaa bbaaa aaaab babab baaaa ababa baaaa baabb abbaa
 baabb aaaab abbaa bbbab babbb baaab babaa bbb
EAGMTA294G           {0} ; 413
 aabba bbbaa abbbb babbb abaaa babab aabaa bbabb baabb bbaab
 baaaa bbaba aaabb b-bbb baabb bbaba baabb aaabb abbba bbbab
 aaab- ababb bbabb aaaaa baaab baaaa bbaaa baaaa abaaa bbbaa
 abaab aabbb abbaa bbbaa aabbb baaab aabaa baa
EAGMTA346G           {0} ; 417
 aabba babab babba abbba bbaab bbbab aabba bbaaa abaab baabb
 bbaaa ababb aaaba b-bba aabab bbaba baaab aaabb abbba bbbab
 abab- ababb bbbbb aabbb abbba bbaba baaba baaba aabaa abaab
 bbaab aabab ababa bbaab baabb baaab abbbb bbb
EACMTC280G           {0} ; 488
 aaaba bbbaa abbbb babbb abaaa bbaab aabaa bbabb baabb abaab
 baaaa bbaba aabbb bbbbb baab- bbaba baabb aaabb abbba bbbab
 baaba ababb bbabb aaaab aaaab baaaa bbaaa baaaa abaaa bbbaa
 abaab aaabb abbaa bbbaa aabbb baaab aabaa baa
EACMTC365G           {0} ; 502
 bbbba aabba abbaa ababb bbaba aabba abaab abbbb baaaa aaaaa
 abaab abbab babaa baaaa baab- baaba bbaaa babbb baaab abbba
 baaaa babab aaaba aaaab baaba aabba aaaaa bbaaa baabb aaaba
 aaabb bbaab bbaaa aabaa babbb baaab aaaab baa
EACMTC384G           {0} ; 509
 aaaba bbbab abbbb aabba bbaaa baaab aabaa bbaba abaab baaab
 bbaaa bbaba ababa bbbbb aaab- bbaba baabb aaabb abbba bbbab
 abaaa ababb bbbbb aaaba aaaab baaaa bbaaa aaaab abbaa bbabb
 abaab aabbb ababa bbbaa baabb baabb aabbb baa
ECAMCT217G           {0} ; 565
 babbb abbab aabbb babab aaaab babab aabbb ababa aaabb babba
 bbaaa aabba bbbba ababa aaab- babbb aabab aaaba abbba baaab
 ababa ababa bbbba abaab bbbbb babba bbbba aabab bbaaa abbaa
 bbbab aaabb baaab bbaab baaba baaaa aabbb bbb
ECAMCT298G           {0} ; 575
 aaabb bbbaa abbbb babbb bbaaa bbbab aabba bbabb babbb bbaab
 baaaa bbaba aaabb bbbbb baab- bbaba baabb aaabb abbba bbbab
 baaba ababb bbabb aaaaa aaaab baaaa bbaaa baaaa abaaa bbbaa
 abaab aabbb abbaa bbbaa babbb baaab aabaa baa
EACMCG225V           {0} ; 643
 aabba bbbaa abbbb bbbbb abaab baaab aabba bbabb baabb abaab
```

```
 abaab bbbaa abbbb bbbab bbabb bbaba baaba aaaba bbbba bbbbb
 baaba aaabb bbbab aaaab babab baaaa bbaba baaaa ababb bbbaa
 baaab aaabb abbaa bbbab aabbb baaab babaa bba
EACMCG361G           {0} ; 655
 aaaba babab babbb abbba bbaab babab aabaa bbaba abaab baaab
 bbaaa ababa abbba bbbba aabab bbaba aaabb aaaba abbba bbbab
 ababa ababb bbbbb aabbb ababb baaba bbaba aaabb abbaa ababb
 bbaab aabab ababa bbaaa baabb baaab aabbb bbb
EACMCG363V           {0} ; 656
 aabba bbbaa bbbab bbbbb abaab baaab babbb bbabb baabb abaaa
 abaab bbbaa abbba bbbab baabb bbaba baaba baaba bbabb bbbba
 baaaa aaaba bbbaa aaaab babab baaaa ababa baaaa ababb bbbaa
 baabb aaaab abbaa bbbab babbb baaab babaa bbb
EACMCG364G           {0} ; 657
 aabba bbbaa bbbab bbbbb abaab baaab babab bbabb baabb abaaa
 abaab bbbaa abbba bbbab baabb bbaba aaaba baaba bbabb bbbba
 baaaa aaaba bbbaa aaaab babab baaaa ababa baaaa ababb bbbaa
 baabb aaaab abbaa bbbaa babbb baaab babaa bbb
ECGMGC316V           {0} ; 718
 aabbb bbbaa bbbab bbabb abaab baaab babab abbbb baabb aaaaa
 abaab abbaa abbba bbbaa baabb aaaba baaaa babba baabb abbba
 babaa aaaaa bbaaa aaaab aabab baaaa ababa baaaa baabb abbaa
 baabb abaab abbaa bbbab babbb baaab babaa bba
ECGMGC392G           {0} ; 724
 bbbab aaabb abbba abaab bbaba aaaba aaaab bbbbb aaaba aaaaa
 aaaaa aaaab abaab ababb babab bbaba baaaa aabbb babbb aabba
 baaaa aaaba aaaba abaab aaaba aaaba aabaa bbbaa aaabb aaaba
 ababb bbaab baaaa aabaa babbb babbb aaaaa bab
ECGMGC448G           {0} ; 725
 baaba aabab aaabb bbbba abaab babaa abbab ababa aaabb aaaba
 bbaaa abbba bbbba ababb baaaa babbb babab aaaba abbba babab
 ababa ababa bbbba abbbb bbbbb babba bbbba babab bbbaa abbaa
 bbbab aabab baaaa bbaba baaba baaab abbbb bbb
ECGMGC493V           {0} ; 726
 babba aabab aaabb bbbbb abaab babab abbbb ababa aaabb aaaba
 bbaaa abbba bbbba ababb baaaa babbb babab aaabb abbaa bbbab
 ababa ababa bbbba abbbb abbbb babaa bbbba babab bbbaa abbaa
 bbbab aabab baaaa bbabb baaba baaab abbbb bbb
EACMGG306G           {0} ; 771
 abbbb aabba abbaa ababb bbaba aaaba abaab abbbb baaab a-aaa
 abaab abbab babaa baaaa baabb baaba bbaaa babbb baaab abbba
 baaaa babab aaaba aaaab baaba aabba a-aaa bbaaa aaabb aaaba
 aaabb bbaab bbaaa aabaa babbb baaab aaaab baa
EACMGG317G           {0} ; 773
 aabba babaa abbbb babbb abaab babab aabaa bbabb baabb a-aab
 aaaab bbbba abbbb bbbab bbabb bbaba baabb aaaba abbba bbbbb
 baaba ababb bbbab aaaab baaab baaaa b-aba baaaa abaaa bbbaa
 baaab aaabb abbaa bbbaa aabbb baaab babaa baa
EACMGG336G           {0} ; 779
 bbbba aabba abbaa ababb bbaba aaaba aaaab bbbbb baaaa a-aba
 aaaab aabab babab baaaa babbb aaaba bbaaa babbb baabb aabba
 baaaa aaabb aaaba abaab aaaba aabba a-baa bbbaa baabb aaaba
 ababb bbaab bbaaa aabaa babbb babbb aaaab bab
EACMGG482V           {0} ; 793
 bbbba aabba abbaa ababb bbaba aaaba abaab abbbb baaba a-aaa
 abaab abbab babaa baaaa baabb baaba bbaaa babbb baaab abbba
 babaa babab aaaba aaaab baaba aabba a-aaa bbaaa baabb aaaba
```

aaabb bbaab bbaaa aabba babbb baaab aaaab baa
EAGMCT226V {0} ; 818
bbbba abbba abbaa ababb bbaba ababa baabb abbbb baaaa a-aaa
abaab abbab babaa baaaa babbb baaba bbaaa babbb baaaa abbba
baaaa baaab aaaba abaab baaba aabba a-aaa bbbaa baabb aaaba
ababb bbaab bbaaa aabba babbb babbb aaaab bab
EAGMCT228G {0} ; 819
bbbba aabba abbaa ababb bbaba ababa baaab abbbb baaaa a-aaa
abaab abbab babaa baaaa babbb baaba bbaaa babbb baaab abbba
baaaa baaab aaaba abaab baaba aabba a-aaa bbbaa baabb aaaba
ababb bbaab bbaaa aabaa babbb babbb aaaab bab
EACMGC173V {0} ; 882
aaaba babab babba abbba bbaab babab aabaa bbaba abaab baabb
bbaaa ababa abbba bbbba aabab bbaba baaab aaabb abbba abbaa
aaaba ababb bbbbb aabbb ababb bbaba bbaba baabb abbaa baaab
bbaaa babbb bbbba bbaab babbb baaba ababa bab
EAGMCA309V {0} ; 961
bbbba aabba ababa ababb baaba aaaba baabb abbbb baaba aaaaa
abaab abbab babaa babaa baabb baaba bbaaa babbb baaab abbba
babaa babab ababa aaaab baaba aabba aaaaa bbaaa ababa ababb
bbaaa baaaa bbaab aabbb aaaab aabbb baabb baa
EAGMCA317G {0} ; 962
bbbba aabba ababa ababb baaba aaaba baaab abbbb baaba aaaaa
abaab abbab babaa babaa baabb baaba bbaaa babbb baaab abbba
babaa babab ababa aaaab baaba aabba aaaaa bbaaa ababa ababb
bbaaa baaaa bbaab aabba aaaab aabbb baabb baa
EAGMCA466G {0} ; 970
baabb aabab aabab bbbba abaab babab abbab ababa aaabb aaaba
bbaaa abbba bbbba ababb baaaa babbb babab aaaba abbba bbbab
ababa ababa bbbba abbbb bbbbb babba bbbba babab abbab bbaab
abaaa babbb bbbbb bbaaa bbaab bbaba ababb aaa
EAGMTT141bV {0} ; 1051
aabbb abaab abbbb babab aaaba baaab aabaa ababa aabba bbbba
baaaa aaaab bbbbb abaaa aaaba bbbab aabab aaabb abaaa aaabb
bbaba abbbb bbbba abaab abbbb aaaba aaabb aabab bbaaa aabaa
babab aaabb bbbab abbbb bbbab baaaa babba bbb
EAGMTT173G {0} ; 1059
babbb aabab aaabb bbbbb abaab babab babab ababa aaabb aabba
bbaaa abbba bbbba ababb aaaba babbb aabab aaabb abbba bbaab
ababa ababa bbbba abbab bbbbb babba bbbba aabab bbbaa abbaa
bbbab aabab baaab bbaba baaba baaaa abbbb bbb
EAGMTT275G {0} ; 1068
baabb aabab aaabb bbbba abaab babab babab baaba aaabb aabba
bbaaa abbba bbbba ababb aaaba babbb babab aaabb abbba bbbab
ababa ababa bbbba abbab bbbbb babba bbbba aabab bbbaa abbaa
bbbab aabab baaab bbaba baaba baaaa abbbb bbb
EAGMTT466V {1} ; 1081
aaabb aaaab baabb babaa abbaa abbbb babaa baaaa abbab bbbbb
babba baaba ababb ababb abbaa aabab aabbb abaab abbba baaab
ababb ababb aabab bbbaa abbab baaab bbbbb abbbb abbaa babbb
bbbaa aabba aabbb ababb abaaa abbba bbbba abb
EACTCC122G {0} ; 1123
aaaba abbaa aaaab babba aaaaa aaaaa aaaaa bbabb aaabb aaaaa
aaaaa aaaba aaaab abaaa baaaa baaaa aaaaa aaaba aabba bbaaa
aaaba abaab baaab aaaaa aaaab aaaaa aaaaa baaaa aaaaa baaaa
abaaa aaaba aabaa aaaaa aabab baaab baaaa aaa
EAGTTA176G {0} ; 1163

```
 aabbb abbab aabbb babab aaaab babab aabbb ababa aaabb bbbba
 bbaaa aaaba bbbbb ababa aaaba babab aabab aaabb abbba baabb
 ababa ababa bbbba abaab abbbb babba bbbba aabab bbaaa abbaa
 bbbab aaabb baaab ababb babaa baaaa aabba bbb
ECGTGT133G          {0} ; 1187
 aabba bbbaa bbbab bbabb abaab baaab abbab abbbb baabb aaaaa
 abaab abbaa abbba bbbab baabb bbaba baaaa babba baabb abbba
 baaaa aaaaa bbaaa aaaab babab baaaa ababa baaaa baabb abbaa
 baabb aaaab abbaa bbbab babbb baabb babaa bbb
EACTAG84V           {0} ; 1226
 aaaba babab babbb abbba bbaab babab aabaa bbaba abaab baaab
 bbaaa ababa abbba bbbba aabab bbaab baaab aaabb abbba bbbab
 ababa ababb bbbbb aabbb abab- bbaba bbaba aaabb abbaa ababb
 bbabb aabab ababa bbaab baabb baabb aabbb bbb
EACTAG247G          {0} ; 1251
 aaaba bbbab abbbb aabba bbaaa babab aabaa bbaba abaab baaab
 bbaaa bbaba ababa babbb aaabb baaab baaab aaaba abbba bbaab
 aaaaa ababb bbabb aaaba aaaa- baaaa bbaaa aaaab abbaa bbabb
 abaab aabbb ababa bbbaa baabb baabb aabbb baa
EACTAG381G          {0} ; 1273
 baabb aabab aaaab bbbba abaab babab babab ababa aaabb aaaba
 bbaaa abbba babba ababb baaaa babab babab aaabb abbba bbbab
 aaaba abaaa bbbba abbbb bbbb- babba bbbba babab bbbaa abbaa
 bbbab aabab baaaa bbabb baaba baabb abbbb bbb
EACTAG383V          {0} ; 1274
 baabb aabab aaabb bbbba abaab babab babbb ababa aaabb aaaba
 bbaaa bbbba babba ababb baaaa babab babab aaabb abbba bbbab
 ababa ababa bbbba abbbb bbbb- babba bbbba babab bbbaa abbaa
 bbbab aabab baaaa bbabb baaba baabb abbbb bbb
EACTAG503V          {0} ; 1282
 aaaba bbbbb bbbbb aabba bbaaa babab aabba bbaba aaaab bbaab
 baaab bbaba aaabb bbbbb aaabb bbaab baabb aaabb abbaa bbbab
 abaaa ababb bbbbb aaabb aaaa- bbaba bbaaa aaaaa abbaa bbabb
 abaab aabbb ababa bbbab bbabb baabb aabba bab
ECGTAC322V          {0} ; 1335
 baabb aabab aaaab bbbba abaab babab babab ababa aaabb aaaba
 bbaaa abbba bbbba ababb baaaa babbb babab aaabb abbba bbbab
 ababa ababa bbbba abbbb bbbb- babba bbbba babab bbbaa abbaa
 bbbab aabab baaaa bbabb baaba baabb abbbb bbb
ECGTAC325G          {0} ; 1337
 baabb aabab aaaab bbbba abaab babab babab ababa aaabb aaaba
 bbaaa abbba bbbba ababb baaaa babbb babab aaabb abbba bbbab
 aaaba ababa bbbba abbbb bbbb- babba bbbba babab bbbaa abbaa
 bbbab aabab baaaa bbabb baaba baabb abbbb bbb
EACTGA212G          {0} ; 1362
 aaaba bbbaa abbbb babbb abaaa bbbab aabaa bbabb baabb bbaab
 baaa- bbaba aaabb bb-bb babbb b-aba baabb aaaba abbba bbbab
 aaaba ababb bbabb aaaaa aaaab baaaa bbbaa babaa bbaba bbbba
 abaab bbbbb abbaa bbbba babbb bbbbb aabaa baa
EACTGA229G          {0} ; 1366
 bbbba bbbba abaaa ababb baabb baaba ababb abbbb baaba aaaaa
 abaa- abbab aabaa ba-aa baabb b-aba baaaa babba baaab abbba
 baaaa babaa abaaa aaabb babba aabba aaaaa baaaa baabb abaaa
 baabb bbaab bbbaa bbbaa babbb baabb aaaab bba
EACTGA229V          {0} ; 1367
 bbbba bbbba abaaa ababb baabb baaba ababb abbbb baaba aaaaa
 abaa- abbab aabaa ba-aa baabb b-aba baaaa babba baaab abbba
```

```
 babaa babaa abaaa aaabb babba aabba aaaaa baaaa baabb abaaa
 baabb bbaab bbbaa bbbaa babbb baabb aaaab bba
EAGTGC457V          {0} ; 1412
 babbb aabab aaabb bbbbb abaab babab babab ababa aaabb aabba
 bbaa- abbba bbbba ab-bb aaaba b-bbb aabab aaabb abbba bbbab
 abbba ababa bbbba abbab abbbb babba bbbba aabab bbbaa abbaa
 bbbab aabab baaab bbabb baaba baaba abbba bbb
EACTTC113G          {0} ; 1446
 aabbb ab-ab aabbb -bbab aaaab baaab aabb- ababa aaaba bbbba
 baaaa aaaab bbbbb abaaa aa--a ---ab aabab aaabb abaaa baabb
 bbaba abbbb bbbba abaab abbbb babba babbb aabab bbaaa abbaa
 babab aaabb baaab abbbb b-baa baaaa babba bbb
EACTTC371V          {0} ; 1471
 bbbba bb-ba bbaaa -aabb bbaab baaaa abab- bbbbb baaba aaaaa
 abaab ababb aabaa babaa ba--b ---ba baaaa babba baaab abbba
 babaa babaa abaaa aaaab babba babba aaaaa baaab baabb abaaa
 baabb bbaab abbaa bbbab b-bbb baabb aaaaa bba
EACTTC425G          {0} ; 1475
 aabba bb-aa bbbab -babb abaab baaab abba- abbbb baabb aaaaa
 abaab ababa abbba bbbab ba--b ---ba baaaa babba baabb abbba
 baaaa aaaaa bbaaa aaaab babab baaaa ababa baaaa baabb abbaa
 baabb aaaab abbaa bbbab b-bbb baabb babaa bbb
EACTTC434G          {0} ; 1478
 aabbb ab-ab abbba -bbab aaaab baabb aaab- ababa aaaba bbbba
 baaaa aaaab bbbbb abaaa aa--a ---ab aabab aaabb abbaa aaabb
 bbaba abbbb bbbba abaab abbbb aaaba babbb aabab bbaaa abbaa
 babab aaabb babab abbbb b-baa baaaa babba bbb
EACTTC434bG         {0} ; 1479
 aabbb ab-ab abbba -bbab aaaab baaab aaab- ababa aaaba bbbba
 baaaa aaaab bbbbb abaaa aa--a ---ab aabab aaabb abaaa aaabb
 bbaba abbbb bbbba abaab abbbb aaaba babbb aabab bbaaa abbaa
 babab aaabb babab abbbb b-baa baaaa babba bbb
EACTTC440V          {0} ; 1480
 aabbb ab-ab abbbb -bbab aaaab babab aaba- ababa aaaba bbbba
 baaaa aaaab bbbbb abaaa aa--a ---ab aaaab aaabb abaaa aaabb
 bbbba abbbb bbbba abaab abbbb aaaba babbb aabab bbaaa abbaa
 babab aaabb babab abbbb b-baa baaaa babba bbb
EAGTAA235G          {0} ; 1514
 aaaba bb-ab abbbb -abba bbaa- babab aaba- bba-a ab-ab b-aab
 -baaa bbaba a-aba bbbbb aa--b ----b baa-b -a--- ----a bbbab
 abaaa ababb bbbbb aaaba aaaab baaaa bbaaa abaab abbaa bbabb
 abaab aabbb ababa b--a- b-abb bbabb aabbb baa
EAGTAA290G          {0} ; 1516
 ----- ----- ----- ----- ----- ----- ----- ----- ----- -----
 ----- ----- ----- ----- ----- ----- ----- ----- ----a bbbab
 baaba ababb bbbab aaaab baaab baaaa bbaba baaaa abaaa bbbaa
 baaab aaabb abbaa b--a- a-bbb bbabb babaa baa
EAGTAA352G          {0} ; 1520
 bbbba aa-bb abbaa -aabb bbab- aaaba aaaa- bbb-b aa-aa a----
 ----- ----- ----- ----- ----- ----- ----- ----- ----b aabaa
 baaaa aaabb aaaba abaab aaaba aabba aabaa bbbaa aaabb aaaba
 ababb bbaab bbaaa a--a- b-bbb bbbab aaaab bab
ECGTGA59G           {0} ; 1529
 aaaba ba-ab babab -bbbb bbaab baaab aaba- baaaa abaab aaaab
 bbaaa ababa abbba bbbba aa--b ---ba baabb aaaba abbba bbbab
 ababa ababb bbbbb aabbb ababb baaba bbaba aaabb abbaa ababb
 bbaab aabab ababa bbaab b-abb baabb aabbb bbb
```

```
ECGTGA109V           {0} ; 1533
 aabba bb-aa bbbab -baba abaab baaab abba- abbbb baabb aaaaa
 abaab abbaa abbba bbbab ba--b ---ba baaab aabba baabb abbba
 babaa aaaaa bbaaa aaaab babab baaaa ababa baaaa baabb abbaa
 baabb aaaab abbaa bbabb b-bbb baabb babaa bbb
SSR_788              {0} ; 1598
 bbbba aabba ab-aa ababb bbaba aaa-a aaaab bbbbb baaaa ababa
 aaaab aabab babab baaab babbb aaa-a --aa- -ab-b baaab aabba
 baaaa aaa-b aaa-a abaab aaaba aabb- -abaa bb--- -aabb aaaba
 ababb bbaab bbaa- aabaa babbb babab aaaab ba-
SSR_875              {0} ; 1603
 aa-aa bbbab abbbb aabba abaab aaaab aabaa bbaba aabbb bbaaa
 ---aa bbaba aaab- bbbba baaba aaaba baaba aaab- -bbba bbbab
 abaaa bbabb bbbbb aaaba aaaab baaaa bbaaa ----- ---aa bbabb
 abaab aabbb abab- bbbaa babbb baabb aabba ba-

LG4:
EGAMGA58V            {0} ; 2
 abbba bbabb aaaaa bbbaa aabbb aabab ababa bbabb ababb aaaaa
 ababa abaab baabb bbbab bbaab abbaa bbabb bbbaa bbbb- -----
 ----- ----- ----- ----- ----- ----- ----- ----- ----- -----
 ----- ----- ----- ----- ----- ----- ----- ---
EGAMGA74G            {0} ; 5
 bbbbb aaaab bbbaa bbbbb aabba abbab bbaaa baaab bbaab bbaaa
 bbbab babbb abaab abbaa aaabb abbbb baabb bbbbb babb- -----
 ----- ----- ----- ----- ----- ----- ----- ----- ----- -----
 ----- ----- ----- ----- ----- ----- ----- ---
EGAMGA207G           {0} ; 21
 abbba bbabb aaaaa bbbaa aabbb aabab ababa bbabb ababb aaaaa
 ababa abaab baabb bbbab bbaab abbaa baabb bbbaa bbbb- -----
 ----- ----- ----- ----- ----- ----- ----- ----- ----- -----
 ----- ----- ----- ----- ----- ----- ----- ---
EGAMGA238G           {0} ; 24
 bbbab aaabb bbbaa bbbbb abbba abbab bbaaa bbaab bbaab baaaa
 bbbab babbb bbaab abbaa baabb aabbb baabb bbbbb babb- -----
 ----- ----- ----- ----- ----- ----- ----- ----- ----- -----
 ----- ----- ----- ----- ----- ----- ----- ---
EGAMGA626G           {0} ; 48
 abbba aaaba aaaaa bbbaa aabab aabab abaaa bbabb ababb aaaaa
 abbbb abaaa baaab bbbab bbaab abaaa aaabb bbbaa abbb- -----
 ----- ----- ----- ----- ----- ----- ----- ----- ----- -----
 ----- ----- ----- ----- ----- ----- ----- ---
ECAMCG62V            {0} ; 52
 abbba aaaba baaaa abbaa aabab abbab ababa bbabb ababb aaaaa
 bbbbb abaaa baaab abbab bbaab abaaa ababb bbbab abbb- -----
 ----- ----- ----- ----- ----- ----- ----- ----- ----- -----
 ----- ----- ----- ----- ----- ----- ----- ---
ECAMCG73V            {0} ; 54
 ababa aaaab bbbaa bbbbb aabba abbab bbaaa baaab bbaab baaaa
 bbbab babbb abaab abbaa aaabb abbbb bbabb bbbbb baab- -----
 ----- ----- ----- ----- ----- ----- ----- ----- ----- -----
 ----- ----- ----- ----- ----- ----- ----- ---
ECAMCG402G           {0} ; 83
 abbaa bbaab bbbaa bbbba aabba babab ababa bbaab abbab aaaaa
 ababa bbabb bbbbb babab bbabb abbaa baabb abbaa bbbb- -----
 ----- ----- ----- ----- ----- ----- ----- ----- ----- -----
 ----- ----- ----- ----- ----- ----- ----- ---
```

```
ECGMCG541G          {0} ; 100
 abaab aaaba baaaa abbaa aabab abbab bbaba bbabb ababb aaaaa
 bbbbb abaaa baaab abbab bbaab abaaa aaabb bbbaa abbb- -----
 ----- ----- ----- ----- ----- ----- ----- ----- ----- -----
 ----- ----- ----- ----- ----- ----- ----- ---
EGAMGC123V          {0} ; 108
 abbba bbabb aaaaa bbbaa aabbb aabab abaaa bbabb ababb aaaaa
 abbba abaaa baabb bbbab bbaab abaaa baabb bbbaa abbba aabaa
 babab baaba aabaa babaa bbbaa abbaa abbba aabaa abaaa bbaaa
 bbbab bbaab babbb abbbb abbab bbaab aaabb bab
ECAMGG71G           {0} ; 243
 babab aaabb bbbba bbbab aabaa abbaa bbaaa bbaab bbaab baaaa
 bbbab babbb bbaaa bbbaa baabb aabbb baabb bbbbb babba aaabb
 aaaba abbaa bbbab baaaa ababa bbbba abbaa abbbb abbaa bbabb
 abbba bbaba bbbba babba aabbb bbbba ababb bab
ECAMGG220G          {0} ; 258
 abbbb aaaba baaaa abbaa aabab abbab baaba bbabb ababb aaaaa
 bbbbb abaaa abaaa bbbba bbaab abaaa aaabb bbbaa abbba babaa
 babab baaba aabaa babab bbbab abbaa abbba babaa bbaba bbaaa
 bbbab bbbba babbb abbba abbab bbbbb aaabb bab
ECAMGG315G          {0} ; 269
 aabbb baaba baaba babaa abbab aabaa bbaaa aaaaa abbbb babba
 bbbba abbaa baaab abbbb aaaab aaaaa ababb aabaa bbbaa aaabb
 bbbba aaaba babaa bbbab ababa abbab abbbb baaba abaab abaaa
 bbabb babab aabaa babaa abbab bbaaa babbb baa
ECAMGG356V          {0} ; 275
 abaaa aaaba baaba aabab aabab abbaa bbaba bbaba ababb babba
 bbbbb abbab bbaaa abbbb baabb bbbba ababb aabab bbbba baaab
 bbbaa baaaa babba bbbab abaab abbab abbbb aabba bbaaa bbaaa
 bbbbb abbab bbbaa ababb bbbab bbbba aabba bba
ECAMGG356G          {0} ; 276
 abaaa aaaba baaba aabab aabab abbaa bbaba bbaba ababb babba
 bbbbb abbab bbaaa abbbb baabb bbbba ababb aabab bbbba baaab
 bbbaa baaaa babba bbbab abaab abbab abbbb aabba bbaaa bbaaa
 bbbbb abbab bbbaa ababa bbbab bbbba aabba bba
ECGMTA589V          {0} ; 335
 bbbbb aaaab bbbaa bbbbb aabba abbab bbaaa baaab bbaaa baaaa
 bbbab babbb baaaa abbaa aaabb abbbb baabb bbbbb babba baaba
 aaaba abbaa bbbbb aaaba bbaaa bbbba aabaa ababb bbbaa bbabb
 abbba ababa baaaa babbb aabbb bbbba ababb bab
EACMGA100V          {0} ; 338
 bbbbb aaabb bbbba bbbbb aabba abbab bbaaa bbaab bbaab baaaa
 bbbab babbb bbaab aabaa baabb aabbb baabb bbbbb babba aaabb
 aaab- abbaa bbbab baaba abaaa bbbba abbaa abbbb abbaa bbabb
 abbba bbaba bbaba babbb aabbb bbbba ababb bab
EACMGA231V          {0} ; 349
 abbaa bbaab bbbba bbbba abbba babab ababa bbaab abbab baaaa
 ababa bbabb bbbbb babab bbabb abbba baabb abbaa bbbbb aabab
 aaaa- bbbba aabaa babba bbbaa babba abbba abbaa abaaa bbbaa
 bbbab abaaa babab abbbb aabaa bbbbb aaaab aab
EACMGA269V          {0} ; 358
 abbaa bbaab bbbaa bbbba aabba babab ababa bbaaa abbab aaaaa
 ababa bbabb bbbbb babab bbabb abbaa baabb abbaa bbbbb aabab
 aaaa- bbaba aabaa babba bbbaa baaba abbba abbaa abaaa bbbaa
 bbbab abaaa babab ababb aabaa bbbbb baaab aab
EAGMTA196V          {0} ; 399
 aabba baaba baaba babaa abbab aabaa bbaaa aaaaa abbbb bbbba
```

```
 babba bbbba abaaa b-bbb aaaab baaaa ababb aabab bbbaa aaaab
 babb- aaaba babbb bbbaa ababa abbab abbbb baaba abaab abaaa
 bbabb aabab aabaa babbb abbab bbbba babbb bba
EAGMTA312G          {0} ; 414
 abaab aaaba baaba aabaa aabab abbaa bbaba baaba ababb babba
 bbbbb abaaa baaab a-bbb bbaab aaaba ababa babaa abbba baaaa
 bbba- bbaaa aabab bbbab bbbab abbaa abbbb bbbaa bbaab bbaaa
 bbbab bbbab bbbbb abbab bbbab bbaaa aabba bab
ECGMGA470G          {0} ; 444
 bbbab aaabb bbbba bbbbb aabba abbab bbaaa bbaab bbaab baaaa
 bbbab babbb bbaab a-baa baabb aabbb baabb bbbbb babba aaabb
 aaab- abbaa bbbab baaba abaaa bbbba abbaa abbab abbaa bbabb
 abbba bbaba bbaba babaa aabbb bbbba ababb bab
EACMTC71V           {0} ; 449
 aabba baaba baaba babaa abbab aaaaa bbaaa aaaaa abbbb bbbba
 babba abbba abaaa bbbbb aaaa- baaaa ababb aabab bbbaa aaaab
 babba aaaba babbb bbbaa ababa abbab abbbb baaba aaaab abaaa
 bbabb aabab aabaa babbb abbab bbbba babbb bba
EACMTC87V           {0} ; 451
 aaaaa aaaab bbbba bbbba aaaba baaaa bbaba bbaab bbbab baaaa
 abaaa bbabb babab babab bbab- abbaa baabb abbba babbb aaaab
 aaaab abaaa aabaa baaaa abbaa aaabb aabba abbaa abaaa bbbaa
 bbbaa bbaaa baaab ababa aabaa bbbbb baaaa bab
ECAMCT78V           {0} ; 539
 baaab aaaab bbbbb bbbbb aaaba abbab bbaba bbaab bbaab bbaaa
 bbaab bbabb bbaab aabab baab- aabba babbb bbbba babba baaab
 aaaaa abaaa aabaa baaaa abbaa babba abbba aabbb abaaa bbbbb
 abbaa bbaba bbabb babba aabbb bbbba aaaba baa
ECAMCT123G          {0} ; 549
 abbba bbabb aaaaa bbbaa aabbb aabab ababa bbabb ababb aaaaa
 ababa abaab baabb bbbab bbaa- abbaa baabb bbbaa bbbba aabaa
 babab bbaba aabaa babaa bbbaa aabaa abbba aabaa ababa bbbaa
 bbbab bbaaa babbb abbaa abbab bbaab aaabb bab
ECAMCT155G          {0} ; 554
 abaab aaaba baaba aabaa aabab abbaa bbaba baaba ababb babba
 bbbbb abbaa baaaa abbbb baaa- aaaba ababa aabaa abbba baaab
 bbbaa baaaa babaa bbbaa abaab abbab abbbb aabba bbaaa bbaaa
 bbbbb abbab babaa abaab abbab bbaaa aabba baa
ECAMCT237V          {0} ; 569
 aabba baaba baaba babaa abbab aabaa bbaaa aaaaa abbbb bbbba
 babba abbba abaaa bbbbb aaaa- baaaa ababb aabab bbbaa aaaab
 babba aaaba babbb bbbaa ababa abbab abbbb baaba abaab abaaa
 bbabb aabab aabaa babbb abbab bbbba babbb bba
ECGMTT217V          {0} ; 609
 abbba bbabb baa-a bbbaa aabbb aabab baaba bbabb ababb aaaaa
 abbba abaaa baabb bbbab bba-- ababa baabb bbbbb abbbb aaaaa
 babab baaba abbaa babaa bbbaa abbaa abbba aabaa ababa bbaaa
 bbbab bbaaa babbb abbba abbab bbaab aaabb bab
EACMCG42G           {0} ; 627
 bbbab aaabb bbbba bbbbb aaaba abbab bbaaa bbaab bbaab baaaa
 bbbab babbb bbaab abbaa baabb aabbb babbb bbbbb babba aaabb
 aaaba abbaa bbbab baaba abaaa bbbba abbaa abbbb abbaa bbbbb
 abbba bbaba bbaba babba aabbb bbbba ababb baa
EACMCG109G          {0} ; 634
 aaaaa aaaab aaaaa bbbaa aaaba aaaaa baaaa bbaab abaab aaaaa
 abaaa abaab baaab babab bbaab abbaa aaabb abbaa babba aaaaa
 aaaab abaaa aabaa baaaa abbaa aaaaa aabba aabaa abaaa bbbaa
```

bbbaa bbaaa baaab ababa aabaa bbaab aaaaa bab
EACMCG265V {0} ; 649
bbbab aaabb bbbba bbbbb aaaba abbab bbaaa bbaab bbaab baaaa
bbbab babbb bbaab abbaa baabb aabbb babbb bbbbb babba aaabb
aaaba abbaa bbbab baaba abaaa bbbba abbaa abbbb abbaa bbbbb
abbaa bbaba bbaba babbb aabbb bbbba ababb baa
EACMCG310G {0} ; 651
aabba baaba baaba aabaa abbab aabaa bbaaa aaaaa abbbb bbbba
babba abbba abaaa bbbbb aaaab baaaa ababb aabaa bbbaa aaaab
babba aaaba babbb bbbaa ababa abbab abbbb baaba abaab abaaa
bbabb aabab aabaa baaaa abbab bbbba babbb bba
EAGMGG149V {0} ; 673
baaab aaaab bbbbb bbbbb aaaba abbaa bbaba bbaab bbaab baaaa
bbaab bbabb bbaab aabab baabb abbba babbb bbbba babbb baaab
aaaaa abaaa aabaa baaaa abbaa bbaba abbba aabab abaaa bbbbb
bbbaa bbaaa bbabb bbbbb aabba babbb aaaba baa
EAGMGG206G {0} ; 680
abaab aaaba baaba aabaa aabab abbaa bbaaa baaba ababb babba
bbbbb abbaa baaaa abbab baaab aaaba ababa aabaa abbba baaab
bbbaa baaaa babaa bbbab abaab abbab abbbb aabba bbaaa bbaaa
bbbab abbaa babaa ababa abbab bbaaa aabba baa
EAGMGG240V {0} ; 687
abbba bbabb aaaaa bbbaa aabbb aabab ababa bbabb ababb aaaaa
ababa abaab baabb bbbab bbaab abbaa baabb bbbaa bbbba babaa
babab bbaba aabaa babaa bbbaa aabaa abbba aabaa ababa bbbaa
bbbab bbaaa babbb abbbb abbab bbaab aaabb bab
EAGMGG336G {0} ; 695
bbbab aaabb bbbba bbbbb aaaba abbab bbaaa bbaab bbaab baaaa
bbbab babbb bbaab abbaa baabb aabbb babbb bbbbb babba aaabb
aaaba abbaa bbbab baaba abaaa bbbba abbaa abbbb abbaa bbbbb
abbaa bbaba bbaba babba aabbb bbbba ababb baa
EACMGG324G {0} ; 775
abaab aaaba baaba aabaa aabab abbaa bbaba baaba ababb b-bba
bbbbb abaaa baaaa abbbb baaab aaaba ababa babaa abbba baaab
bbbaa baaaa aabaa bbbab abaab abbaa a-bbb babba bbaaa bbaaa
bbbbb abbab babab abaab abbab bbaaa aabba baa
EAGMCT104V {0} ; 804
abaab aaaba baaba aabaa aabab abbaa bbaba baaba ababb b-bba
bbbbb abaaa baaaa abbbb baaab aaaba ababa babaa abbba baaab
bbbaa baaaa aabaa bbbab abaab abbaa a-bbb babba bbaaa bbaaa
bbbbb abbab babab ababb abbab bbaaa aabba baa
EAGMCT355V {0} ; 831
abaab aaaba baaba aabaa aabab abbaa bbaba baaba ababb b-bba
bbbbb abbaa baaaa abbbb baaab aaaba ababa aabab abbba baaab
bbbaa baaaa babaa bbbab abaab abbab a-bbb aabba bbaaa bbaaa
bbbbb abbab babaa ababb abbab bbaaa aabba baa
EAGMCT361G {0} ; 832
abbba bbabb aaaaa bbbaa aabbb aabab baaaa bbabb ababb a-aaa
ababa abaab baabb bbbab bbaab abbaa aaabb bbbaa bbbba aabaa
babab bbaba aabaa babaa bbbaa aabaa a-bba aabaa ababa bbbaa
bbbab bbaaa babbb abbaa abbab bbaab aaabb bab
EACMGC60G {0} ; 869
abbba aaaba baaaa abbaa aabab abbab ababa bbabb ababb aaaaa
bbbbb abaaa baaab abbab bbaab abaaa aaabb bbbaa abbba babaa
babab baaba aabaa babab bbbab abbaa abbba babaa babbb aabba
bbbbb baaab bbaba babaa baaab bbabb bbbab abb
EACMGC123G {0} ; 876

abaab aaaba babaa aabaa aabab abbaa bbaba baaba ababb babba
bbbbb abbaa baaaa abbbb baaab aaaba ababa aabaa abbba baaab
bbbaa baaaa babaa bbbab abaab abbab abbbb aabba aabab babba
bbbaa aaabb ababb baaaa baabb bbbba bbabb aba
EACMGC282V {0} ; 896
aabba baaba babaa babaa abbab aabaa bbaaa aaaaa abbbb bbbba
babba abbba abaaa bbbbb aaaab baaaa ababb aabaa bbbaa aaaab
babba aaaba babbb bbbaa ababa abbab abbbb baaba ababa babba
bbbbb ababb bbbaa baabb baaab babba ababa aba
EACMGC296G {0} ; 898
abaab aaaba babaa aabaa aabab abbaa bbaba baaba ababb babba
bbbbb abbaa baaaa abbbb baaab aaaba ababa aabaa abbba baaab
bbbaa baaaa bbbaa bbbab abaab abbab abbbb aabba aabab babba
bbbaa aaabb abaab baaaa baaab bbbba bbabb bba
EAGMCA525V {0} ; 976
abbba bbabb aaaaa bbbaa aabbb aabab baaba bbabb ababb aaaaa
ababa abaab baabb bbbab bbaab abbaa baabb bbbaa bbbba aabaa
babab bbaba aabaa babaa bbbaa aabaa abbba aabaa babbb aabba
bbbaa baaab bbaba babab bbaab bbabb baaab abb
ECGMCA182V {0} ; 982
abbba bbabb aaaaa bbbba aabbb aabab ababb bbbbb ababb aaaaa
ababa abaab baabb bbbab bbaab abbaa baabb bbbaa bbbba babaa
babab abbba aabaa babaa bbbaa aabaa abbba aabaa babbb aabba
bbbaa baaab bbaba babab bbaab bbabb baaab abb
ECGMCA484V {0} ; 998
abbaa bbaab bbaba bbbba aabba babab ababa bbabb abbab aaaaa
ababa bbabb bbbbb babab bbabb abbaa baabb abbaa bbbbb aabab
aaaab bbaba aabaa babba bbbaa babba abbba abbaa babab aaaba
abbbb babaa baaba baaab bbaab bbaba baaab aba
EACMGT360V {0} ; 1032
bbbbb aaaab bbaaa bbbbb aabba abbab bbaaa baaab bbaab baaaa
bbbab babbb abaab abbaa aaabb abbbb baabb abbbb babba aaabb
aaaba abbaa bbbbb aaaba bbaaa bbbba abbaa abbbb bbbaa bbabb
abbba ababa bbaba babbb aabbb bbbba ababb baa
EAGMTT141V {0} ; 1050
abaab aaaba baaba aabaa aabab abbaa bbabb baaba ababb babba
bbbbb abbaa baaaa abbbb baaab aaaba ababa aabaa abbba baaab
bbbaa baaaa babaa bbbab abaab abbab abbbb aabba bbaaa bbaaa
bbbbb abbab babaa ababb abbab bbaaa aabba baa
EAGMTT352G {0} ; 1073
abbba bbabb aaaaa bbbaa aabbb aabab ababa bbabb ababb aaaaa
ababa abaab baabb bbbab bbaab abbaa baabb bbbaa bbbba abaaa
babab bbaba aabaa babaa bbbaa aabaa abbba aabaa ababa bbbaa
bbbab bbaaa babbb abbba abbab bbaab aaabb bab
EACTCC389V {0} ; 1144
aabaa aaaab bbbba bbbbb aaaba baaaa bbbbb bbaab bbbab baaaa
abaaa bbbbb babbb babab bbabb abbaa baabb abbbb babbb aabab
babab abaaa aabaa baaaa abbaa aaabb babba abbba abaaa bbaaa
bbbba bbaaa baaab ababa aabaa bbbaa baaaa baa
EAGTTA47V {0} ; 1155
abbba ababa aaaaa bbbaa aabab aabab ababa bbabb ababb aaaaa
abbbb abaaa baaab bbbab bbaab abaaa aaabb bbbaa abbba aabaa
babab baaba aabaa babab bbbab abbaa abbba aabaa ababa bbaaa
bbbab bbaba babbb abbba abbab bbabb aaabb bab
EAGTTA49G {0} ; 1156
abbba ababa aaaaa bbbaa aabab aabab ababa bbabb ababb aaaaa
abbbb abaaa baaab bbbab bbaab abaaa aaabb bbbaa abbba aabaa

```
 baaab baaba aabaa babab bbbab abbaa abbba aabaa ababa bbaaa
 bbbab bbaba babbb abbba abbab bbabb aaabb bab
EAGTTA190G          {0} ; 1165
 abbba bbabb aaaaa bbbaa aabbb aabab abaaa bbabb ababb aaaaa
 abbaa abaab baabb bbbab bbaab abbaa baabb bbbaa bbbba aabaa
 baaab bbaba aabaa babaa bbbaa aabaa abbba aabaa ababa bbbaa
 bbbab bbaaa babbb abbba abbab bbabb aaabb bab
EAGTTA441G          {0} ; 1171
 aaaab aaaab bbbbb bbbbb aaaba abbaa bbaaa bbaab bbaab baaaa
 bbaaa bbabb baaab aabab baabb abbba babbb bbbba babbb aaaab
 aaaaa abaaa aabaa baaaa abbaa bbaba aabba aabab abaaa bbbbb
 bbbaa bbaaa bbabb bbbba aabba bbbab baaaa baa
EACTAG154V          {0} ; 1240
 bbbab aaabb bbbba bbbbb aaaba abbab bbaaa bbaab bbaab baaaa
 bbabb babbb bbaab abbaa baabb aabbb babbb bbbbb babba aaabb
 ababa abbaa bbbab baaba abaa- bbbba abbaa abbbb abbaa bbbbb
 abbba bbaba bbaba babba aabbb bbbaa ababb baa
ECGTAC354V          {0} ; 1341
 abaab aaaba baaaa aabaa aabab abbaa bbaba baaba ababb babba
 bbbbb abbaa baaaa abbbb baaab aaaba ababa aabaa abbba baaab
 bbbaa baaaa babaa bbbab abaa- abbab abbbb aabba bbaaa bbaaa
 bbbbb abbab babaa ababb abbab bbaba aabba baa
ECGTAC357G          {0} ; 1342
 abaab aaaba baaaa aabaa aabab abbaa bbaba baaba ababb babba
 bbbbb abbaa baaaa abbbb baaab aaaba ababa aabaa abbba baaab
 babaa baaaa babaa bbbab abaa- abbab abbbb aabba bbaaa bbaaa
 bbbbb abbab babaa ababb abbab bbaba aabba baa
ECGTCA144V          {0} ; 1421
 aabba baaba baaba babaa abbab aabaa bbaaa aaaaa abbbb bbbba
 babb- abbba abaaa bb-bb aaaab b-aaa ababb aabab bbbaa aaaab
 babba aaaba babbb bbbaa ababa abbab abbbb baaba abaab abaaa
 bbabb aabab aabaa babab abbab bbbba babbb bba
EACTTC117G          {0} ; 1447
 aabaa aa-ab bbbba -bbba aaaba baaaa bbab- bbaab bbbab baaaa
 abaaa bbbab babab babab bb--b ---aa baabb abbba babbb aaaab
 aaaab abaaa aabaa baaaa abbaa aaabb aabba abbaa abaaa bbbaa
 bbbaa bbaaa baaab ababa a-baa bbbab baaaa bab
EACTTC172G          {0} ; 1452
 abbba aa-ba aaaaa -bbaa aabab aabab abaa- ababb ababb aaaaa
 abbbb abaaa baaab bbbab bb--b ---aa aaabb bbbaa abbba aabaa
 baaab baaba aabaa babab bbbab abbaa abbba aabaa ababa bbaaa
 bbbab bbaba babbb abbba a-bab bbabb aaabb bab
EACTTC580V          {0} ; 1496
 abbba aa-ba aaaaa -bbaa aabbb aabab abab- ababb ababb aaaaa
 abbbb abaaa baaab bbbab bb--b ---aa aaabb bbbab bbbaa aabaa
 babab baaba aabaa babab bbbab abbaa abbba aabaa ababa bbaaa
 bbbab bbaba babbb abbba a-bab bbabb aaabb bab
EAGTAA203G          {0} ; 1511
 abaab aa-ba baaba -abaa aaba- abbaa bbaa- baa-a ab-bb b-bba
 -bbbb abbaa b-aaa abbbb ba--a ----b aba-a -a--- ----a baaab
 bbbaa baaaa babaa bbbab abaab abbab abbbb aabba bbaaa bbaaa
 bbbbb abbab babaa a--b- a-bab bbaba aabba baa
EAGTAA347G          {0} ; 1519
 abbaa bb-ab bbbaa -bbba aabb- bab-b abaa- bba-b ab-ab a----
 ----- ----- ----- ----- ----- ----- ----- ----- ----b aabab
 aaaab bbaba aabaa babba bbbaa babba abbba abbaa abaaa bbbaa
 bbbab abaaa babab a--b- a-baa bbbbb abaab aab
```

```
EAGTAA445V           {0} ; 1523
 ----- ----- ----- ----- ----- ----- ----- ----- ----- -----
 ----- ----- ----- ----- ----- ----- ----- ----- ----a aaabb
 babba aaaba babbb bbbaa ababa abbab abbbb baaba abaab abaaa
 bbabb aabab aabaa b--a- a-bab bbbba babbb bba
EAGTAA467G           {0} ; 1526
 ----- ----- ----- ----- ----- ----- ----- ----- ----- -----
 ----- ----- ----- ----- ----- ----- ----- ----- ----a baaab
 bbbaa baaaa babaa bbbab abaab abbab abbbb aabba bbaaa bbaaa
 bbbbb abbab babaa a--b- a-bab bbaba aabba bab
ECGTGA348G           {0} ; 1556
 bbbab aa-bb bbbba -bbba aabba abbbb bbaa- bbaab bbaab baaaa
 bbabb babbb bbaab abbaa ba--b ---bb baabb bbbbb baaba aaabb
 aaaba abbaa bbbab baaba abaaa bbbba abbaa abbbb abbaa bbabb
 abbba bbaba bbaba babba a-bbb bbbaa ababb bab
SSR_808              {0} ; 1600
 aaaab aaaab bbbbb bbbbb aaaba abaaa bbaba bbaab bbbbb baaaa
 bbaaa bbabb baaab aabaa aaabb abbba babbb bbbba babbb aaaab
 aaaaa abaaa aabaa baaaa abbaa bbaba aabba aabab abaaa bbaaa
 bbbaa bbaaa bbab- baaba aa-ba bbbab baaaa baa

LG5:
EGAMGA102G           {0} ; 9
 bbaba aabba babab aabbb bbabb babbb aaaab aabba baaaa abbbb
 abbbb ababa babaa baaaa baaab aabab babba abbab bbaa- -----
 ----- ----- ----- ----- ----- ----- ----- ----- ----- -----
 ----- ----- ----- ----- ----- ----- ----- ---
EGAMGA452G           {0} ; 39
 ababa babbb babba aabbb bbabb babab aaaab aabba baaaa abaab
 abaaa ababa bbbaa baaaa baaab aaaab babaa abbab abaa- -----
 ----- ----- ----- ----- ----- ----- ----- ----- ----- -----
 ----- ----- ----- ----- ----- ----- ----- ---
EGAMGC183G           {0} ; 117
 aaaba bbbbb ababa bbaaa babba bbbaa baaab babba baaaa baaba
 aabbb aaaba aabab babba baaba abbba aabbb abbaa abbab ababa
 bbaab aaaba bbbbb aaaab abbbb bbaab abaab bbbab babaa baaaa
 aabbb ababb bbbaa bbaba baaab aabba aabaa aaa
EGAMGC235V           {0} ; 122
 bbbaa aabba babbb aabbb baabb babbb aaaab aabba baaaa abbbb
 abbbb ababa babaa baaaa baaab aabab bbbba abbab bbaaa aaabb
 bbaba abbaa baabb ababa babab abaab aabbb babaa baabb bbaab
 aaabb bbbba abaaa bbbbb bbbba bbbba bbbba aba
EGAMGC395G           {0} ; 127
 aabaa babbb babba aabbb bbabb bbaab aaabb abbba baaaa abbab
 abaaa aabba bbbaa baaaa baaab abaab babaa abbab abaaa aaabb
 bbbaa abaaa babbb aaabb aaaaa abbab aaabb babba baaaa aaabb
 abbab ababa abaab baaaa baaaa abaab bbbba aaa
ECGMCC342G           {0} ; 186
 abbaa ababa bbbab aabaa bbaba bbbbb aaaab ababa bbbba bbbab
 abbab ababb babbb babba baaab aaaab abbba abbab bbaaa aaabb
 abaab abb-b ababa bbaaa abbba abaab aabab bbbaa babab baaab
 baabb abbba bbaab baaba bbbba abaaa babba bba
ECGMCC367G           {0} ; 187
 abbba ababa bbbab aabaa bbaba bbbbb aaaab ababa bbbba bbbbb
 abbab ababa babbb babba baaab aaaab abbba abbab bbaaa aaabb
 abaaa abb-b aaaba bbaaa abbba abaab aabab bbbaa babab baaab
 baabb abbba bbaab baaaa bbbba abaaa babba bba
```

```
ECGMCC373V           {0} ; 188
 abbaa ababa bbbab aabaa bbaba bbbbb aaabb ababa bbbba bbbbb
 abbab ababa babbb babba baaab aaaab abbba abbab bbaaa aaabb
 abaaa abb-b aaaba bbaaa abbba abaab aabab bbbaa babbb baaab
 baabb abbba bbaab baaab bbbba abaaa babba bba
ECGMCC453V           {0} ; 190
 aabaa bbbbb aabaa abaab abbbb bbaaa aabbb aabba baaaa babab
 abbaa aabbb bbbaa bbbba baaab abaab babaa abaab abbab aaaba
 bbbab aba-a baaba baabb abaab bbbbb baabb aabbb baaba aaabb
 abbaa ababb abaaa baaab baaab abaaa babba aaa
ECGMCC455G           {0} ; 191
 aabaa bbbbb aabaa abaab abbbb bbaaa aabab aabba baaaa babab
 abbaa aabbb bbbaa bbbba baaab abaab babaa abaab abbab aaaba
 bbbab aba-a baaba baabb abaab bbbbb baabb aabbb baaaa aaabb
 abbaa ababb abaaa baaaa baaab abaaa babba aaa
EACMCC342G           {0} ; 222
 aaaba babbb babba aabbb bbabb bbaab aaabb aabba baaaa abbab
 abaaa aabba bbabb aaaaa baaab abaab babaa abbab abaaa aaabb
 bbbaa abaaa babbb aaabb aaaaa abbab aaabb babba baaaa aaabb
 abbab ababb abaab baaaa baaaa abaab bbbba aaa
ECAMGG74G            {0} ; 244
 aaaba bbbab aabaa abaaa abbbb bbaaa aabbb aabba baaaa babab
 abbaa aabbb bbabb abbab baaab abaab babaa abaab abbab aaaba
 bbbab abaaa baaba baabb abaab bbbbb baabb aabbb baaba aaabb
 abbaa ababa abaaa baaaa baaab abaaa babba aaa
ECGMTA43V            {0} ; 298
 aabaa bbbbb ababa bbaaa abbba bbbaa ababb aabba baaaa babaa
 aabbb aaaba baabb bbbab baabb abaaa babbb bbaaa abbab aaaba
 baaab abaaa baabb baaab abaab bbbbb bbaab bbbab baaba aaaba
 aaaaa ababb aaaaa bbabb baaab aabba aabba aaa
ECGMTA79G            {0} ; 300
 ababa babbb babba aabbb bbabb baaab aaaab aabba baaaa abaab
 abaaa ababa bbabb aaaaa baaab aaaab babaa abbab abaaa aaabb
 bbbaa abaaa babbb aaabb aaaaa abaab aabbb babba baaaa baaab
 ababb ababa aaaab babaa baaba abaab bbbba aba
ECGMTA82G            {0} ; 302
 bbaba aabba babbb aabbb baabb babbb aaaab aabba baaaa abbbb
 abbbb ababa ababb aaaaa aaaab aabab bbbba abbab bbaaa aaabb
 bbaba abbaa baabb ababa babab abaab aabbb babaa baaab bbaab
 aaabb bbbba aaaaa bbbba bbbba bbbba bbbba aba
EACMGA230bG          {0} ; 348
 abaaa aaaba bbbab aabba babbb babbb aaaab ababa bbbba bbbbb
 abbab ababa bbbbb babba baaab aaaab abbba abbab bbaaa aaabb
 bbab- abbab aaaba bbaaa abbba abaab aabab bbbaa baaab babbb
 baabb abbba bbaab bbaaa bbbba abaaa babba bba
EACMGA237V           {0} ; 351
 aaaaa babbb babba aabbb bbabb baaab aaabb aabba baaaa abbab
 abaab aabba bbbaa bbbaa baaab abaab babaa abaab abaaa abaab
 bbba- abaaa babbb aaabb aaaba abbbb aaabb aaaab baaaa aaabb
 aabab ababb abaab baaab baaaa abaaa bbbba aaa
EACMTC170G           {0} ; 465
 babab bbbbb aaaba bbaaa aabba bbaaa baaab baaba baaaa baaba
 aabbb aaaaa aabab babba baab- bbbaa aaabb abaaa abaab ababa
 bbaab aabba bbbbb aaaab abbbb bbaab abaab bbbab bbbba baaaa
 abbbb ababa baaaa bbaaa aaaab abaaa aabaa aba
EACMTC256V           {0} ; 487
 aabaa bbbbb ababa bbaaa abbba bbaaa baabb aabba baaaa babaa
```

```
 aabbb aaaba abbab bbbba baab- abaaa babbb abaaa abbab aaaba
 baaab abaaa baabb baaab abaab bbbbb bbaab bbbab babba aaaba
 aaaaa ababb abaaa bbaba baaab aabaa aabba baa
EACMTC299V          {0} ; 491
 aabaa bbbbb ababa bbaba abbba bbaaa baabb aabba baaaa babaa
 aabbb aaaba abbab bbbba baab- abaaa babbb abaaa abbab aaaba
 baaab abaaa baabb baaab abaab bbbbb bbaab bbbab baaba aaaba
 aaaaa ababb abaaa bbaba baaab aabba aabba aab
EACMTC300G          {0} ; 492
 aabaa bbbbb ababa bbaaa aabba bbaaa baabb aabba baaaa babaa
 aabbb aaaba abbab bbbba baab- abaaa babbb abaaa abbab aaaba
 baaab ababa bbabb baaab abbab bbbbb bbaab bbbab babba aaaba
 aaaba ababa abbaa bbaaa baaab aabba aabaa aab
EACMTC368V          {0} ; 504
 bbaba aabba babbb aabbb baabb babbb aaaab aabba baaaa abbbb
 abbbb ababa babaa baaaa baaa- aabab bbbba abbab bbaaa aaabb
 bbaba abbaa baabb ababa babab abaab aabab bbbaa baaab bbaab
 aaabb bbbbb abaaa bbbba bbbba bbbba bbbba aba
ECGMTT144G          {0} ; 600
 aaaba babbb aab-a aabbb bbabb bbaab aaaab aabba baaaa abbab
 abaaa aabba bbbaa baaaa baa-- abaab babaa abbab abaaa aabbb
 bbbaa abaaa babbb aaabb aaaaa abbab aaabb babba baaaa aaabb
 abbab ababa abaab baaaa baaaa abaab bbbba aaa
ECGMTT515G          {0} ; 624
 aabaa bbbbb aaa-a bbaaa babba bbbaa ababb baaba baaaa baaba
 abbbb aaaba aabab babba baa-- abbaa aabbb abaaa abbab ababa
 bbaab aabba bbabb aaaab abbbb bbaab abbab bbbab babba baaaa
 aabbb ababa bbbaa bbaaa baaab abbba aabaa aba
EACMCG170G          {0} ; 636
 aabaa bbbbb ababa bbaaa babba bbbaa ababb babba baaaa baaba
 aabbb aaaba aabab babba baaba abbaa aabbb abaaa abbab ababa
 bbaab aaaba bbbbb aaaab abbbb bbaab abaab bbbab babba baaaa
 aabbb ababa bbbaa bbaba baaab abbba aabaa aaa
EACMCG245G          {0} ; 644
 aaaaa bbbbb aabaa abaab abbbb bbaaa aaabb aabba baaaa babab
 abbbb aabba bbbaa bbbba baaab abaaa babba abaab abbab aaaba
 bbbab abaaa baaba baabb abaab bbbbb baabb babbb baaba aaabb
 aabaa aaaba abaaa baaba baaab aabba babba aaa
EACMGG59G           {0} ; 733
 abbaa ababa bbaab aabaa bbaba abbbb aaaab abaab bbbba b-bab
 bbbab baabb babbb babba aaaab aaaab abbba abbab bbaaa aaabb
 abaab abbab ababa bbaaa ababa abaaa a-aab bbbaa babab baaaa
 babbb abbba bbaab baaaa abbbb abaab babbb bba
EACMGG157V          {0} ; 749
 aaaba bbbbb aabaa abaab bbbbb bbaab aabbb aabba baaaa a-bab
 abaaa aabbb bbbaa bbbba baaab abaab babaa abaab abbab aaabb
 bbbaa abaaa babba baabb aaaab bbbbb a-abb aabba baaaa aaabb
 abbab ababb abaab baaba baaab abaaa bbbba aaa
EACMGG181V          {0} ; 757
 bbbba aabba babbb aabbb baabb babbb aaaab aabba baaaa a-bbb
 abbbb ababa babaa baaaa baaab aabab bbbba abbab bbaaa aaabb
 bbaba abbaa baabb ababa aabab abaab a-bbb babaa baaab bbaab
 baabb bbbbb abaaa bbbba bbbba bbbba bbbba aba
EAGMCT1187G         {0} ; 815
 aabab bbbbb aaaba bbaaa aabba bbbaa ababb baaba baaaa b-aba
 aabbb aaaaa aabab babba baaba bbbaa aaabb abaaa abbab ababa
 bbaab aabba bbbbb aaaab abbbb bbaab a-aab bbbab bbbba baaaa
```

```
 abbbb ababa bbaaa bbaaa aaaab abbba aabaa aba
EACMGC165V           {0} ; 881
 aabaa bbbbb abbaa bbaaa babba bbbaa baabb aabba baaaa baaba
 aabbb aaaba abbab bbbba baabb abaaa babbb abaaa abbab aaaba
 baaab aaaba bbabb baaab abbab bbbbb bbaab bbbab abbab abaaa
 baabb aaaba aaabb abbab aabaa aabaa babba bba
EACMGC472V           {0} ; 921
 aabaa bbbbb abbaa bbaab abbba bbbaa baabb aabba baaaa babaa
 aabbb aaaba abbab bbbba baabb abaaa babbb abaab abbab aaaba
 baaab abaaa baabb baaab abaab bbbbb bbaab bbbab abbab abaaa
 baabb aaabb aaaab aabab aabaa aaaaa babba baa
EACMGC590G           {0} ; 931
 ababa aabba babbb aabab baabb babbb aaaab aabba baaaa abbbb
 abbbb ababa babaa baaaa baaab aabab bbbba abbab bbaaa aaabb
 bbaba abbaa baabb ababa aabab abaab aabbb babaa abbba abbbb
 abbbb abbbb aabba aaaba baaba aabbb bbbaa baa
EAGMCA132V           {0} ; 939
 aabaa bbbbb abbaa bbaaa abbba bbbaa ababb aabba baaaa babaa
 aabbb aaaba abbab bbbba baabb abaaa babbb abaab abbab aaaba
 baaab abaaa baabb baaab abaab bbbbb bbaab bbbab abbab abaaa
 baabb aaabb aaaab aabab aabaa aaaaa babba baa
EAGMCA134G           {0} ; 940
 aabaa bbbbb abbaa bbaaa abbba bbbaa ababb aabba baaaa babaa
 aabbb aaaba abbab bbbba baabb abaaa babbb abaaa abbab aaaba
 baaab abaaa baabb baaab abaab bbbbb bbaab bbbab abbab abaaa
 baabb aaabb aaaab aabaa aabaa aaaaa babaa baa
EAGMCA149V           {0} ; 943
 aabaa bbbbb abbaa bbaaa babba bbbaa ababb aabba baaaa babaa
 aabbb aaaba abbab bbbba baabb abbaa babbb abaaa abbab aaaba
 baaab ababa bbabb baaab abbab bbbbb bbaab bbbab abbab abaaa
 baabb aaaba aaabb abbab aabaa aabaa babba bba
ECGMCA334V           {0} ; 994
 aabaa bbbbb abbaa bbaaa abbba bbbaa baabb aabba baaaa babaa
 aabbb aaaba abbab bbbba baabb abaaa babbb abaab abbab aaaba
 baaab abaaa baabb baaab abaab bbbbb bbaab bbbab abbab abaaa
 baabb aaabb aaaab aabab aabaa aaaaa babba baa
EACMGT45G            {0} ; 1000
 aabab bbbbb aaaba bbaaa aabba bbbaa baabb baaba baaaa babba
 aabbb aaaaa aabab babba baaba bbbaa aaabb abaaa abbbb ababa
 bbaab aabba bbbbb aaaab abbbb bbaab abaab bbbab bbbba baaaa
 abbbb ababa bbaaa bbaba aaaab abbba aabaa abb
EAGMTT502G           {0} ; 1084
 bbaba aabba babbb aabba baabb bbbbb aaaab aabba babaa abbbb
 abbbb ababa babab baaba baaab aaaab bbbba abbab bbaaa aaabb
 bbaba abbab baabb ababa bbbbb abaab aabbb babaa baaab bbaab
 baabb abbba abaaa bbaaa bbbba bbaaa babba bba
EAGMTT506V           {0} ; 1085
 bbaba aabba babbb aabba baabb bbbbb aaaab aabba babaa abbbb
 abbbb ababa babab baaba baaab aaaab bbbba abbab bbaaa aaabb
 bbaba abbab baabb ababa bbbbb abaab aabbb babaa baaab bbaab
 baabb abbbb abaaa bbaab bbbba bbaaa babba bba
EAGTTA64G            {0} ; 1157
 aaaba babbb babba aabbb bbabb bbaab aaabb aabba baaaa abbab
 abaaa aabba bbbaa baaaa baaab abaab babaa abbab abaaa aaabb
 bbaaa abaaa babbb aaabb aaaaa abbab aaabb babba baaaa aaabb
 abbab ababa abaab baaaa baaaa abaab bbbba aaa
ECGTGT244G           {0} ; 1204
```

aaaba babbb babba aabbb bbabb bbaab aaabb aabba baaaa abbab
abaaa aabba bbbaa baaaa baaab abaab babaa abbab abbaa aaabb
bbaaa abaaa babbb aaabb aaaaa abbab aaabb babba baaaa aaabb
abbab ababa abaab baaaa baaaa abaab bbbba aaa
ECGTGT281G {0} ; 1205
abbaa ababa bbaab aabaa bbaba abbbb aaaab abaab bbbba bbbab
bbabb baabb babbb babba aaaab aaaab abbba abbab bbbaa aaabb
abaab abbab ababa bbaaa ababa abaaa aaaab bbbaa babab baaaa
babbb abbba bbaab baaba abbbb ababb babbb bba
ECGTGT281bG {0} ; 1206
abbaa ababa bbabb aabaa bbaba abbbb aaabb abaab bbbba bbbab
bbabb baabb babbb babba aaaab aaaab abbba abbab bbbaa aaabb
abaab abbab ababa bbaaa ababa abaaa aaaab bbbaa babab baaaa
babbb abbba bbaab baaba abbbb ababb babbb bba
ECGTGT392G {0} ; 1217
aaaaa bbbbb aabaa abaab abbbb bbaaa aabbb aabba baaaa babab
abbba aabba bbbaa bbbba baaab aaaaa babba abaab abbab aaaba
bbaab abaaa baaba baabb abaab bbbbb baabb babbb baaba aaabb
aabaa aaaba abaaa baaba baaab aabba babba aaa
EACTAG90G {0} ; 1228
bbaba aabba babbb aabbb baabb babbb aaaab aabba baaaa abbbb
abbbb ababa babaa baaaa baaab aabba bbbba abbab bbaaa aaabb
baaba abbaa baabb ababa baba- abaab aabbb babaa baaab bbaab
aaabb bbbba abaaa bbbba bbbba bbbba bbbba aba
EACTAG372G {0} ; 1270
aaaba bbbbb aabaa abaab bbbbb bbaab aabab aabba baaaa aabab
abaaa aabbb bbbaa bbbba baaab ababa babaa abaab abbab aaabb
babaa ababa babba baabb aaaa- bbbbb baabb aabba baaaa aaabb
abbab ababa abaaa baaaa baaab abaaa bbbba aaa
EAGTCA248G {0} ; 1302
bbaba aabba babbb aabbb baabb babbb aaaab aabba baaaa abbbb
abbbb ababa babaa baaaa baaab aabab bbbba abbab bbaaa aaabb
baaba abbaa baabb ababa baba- ababb aabbb babaa baaab bbaab
aaabb bbbba abaaa bbbba bbbba bbbba bbbba aba
EAGTGC82V {0} ; 1389
bbaba aabba babbb aabbb baabb babbb aaaab aabba baaaa abbbb
abbb- ababa babaa ba-aa baaab a-bab bbbba abbab bbaaa aaabb
bbbba abbaa baabb ababa bbbab abaab aabbb babaa baaab bbaab
aaabb bbbbb abaaa bbaaa abbba bbbba bbbba aaa
ECGTCA40V {0} ; 1418
abbaa ababa bbbab aabaa bbaba abbbb aaabb abaab bbbba bbbab
bbab- baabb babbb ba-ba aaaab a-aab abbba abbab bbaaa aaabb
abbab abbab ababb bbaaa ababa abaaa aaaab bbbba babab baaaa
babbb abbbb bbaab baaba bbbbb ababb babbb bba
EACTTC67V {0} ; 1439
ababa ba-ba babba -abbb bbabb babab aaaa- babba baaaa abaab
abaab abbaa bbbaa baaaa ba--b ---ab babaa abbab abaaa aaabb
bbbba abbaa babbb aaabb aaaaa abaab aabbb babba baaaa baaab
ababb bbabb abaab babaa b-aba bbbaa bbbba aba
EACTTC89V {0} ; 1444
aabab bb-bb aaaba -baaa aabba bbbaa baab- baaba baaaa baaba
aabbb aaaaa aabab babba ba--a ---aa aaabb abaaa abbab ababa
bbbab aabba bbbbb aaaab abbbb bbaab abaab bbbab bbbba baaaa
abbbb ababb bbaaa bbaba a-aab abaaa aabaa aba
EACTTC496G {0} ; 1488
aabaa bb-bb ababa -baaa babba bbbaa baab- babba baaaa baaba
aabbb aabaa aabab babba ba--a ---aa aabbb abaaa abbab ababa

```
 bbaab aaaba bbbbb aaaab abbbb bbaab abaab bbbab babba baaaa
 aabbb ababa bbbaa bbaba b-aab aabaa aabaa aaa
EACTTC571G           {0} ; 1495
 ababa ba-bb babba -abbb bbabb babab aaaa- aabba baaaa abaab
 abaaa abbaa bbbaa baaaa ba--b ---ab babaa abbab abaaa aaabb
 bbaaa abbaa babbb aaabb aaaaa abaab aabbb babba baaaa baaab
 ababb ababa abaab babaa b-aba bbaaa bbbba aba
EAGTAA355G           {0} ; 1521
 ----- ----- ----- ----- ----- ----- ----- ----- ----- -----
 ----- ----- ----- ----- ----- ----- ----- ----- ----b abaaa
 bbaab aabba bbbbb aaaab abbbb bbaab abaab bbbab bbbba baaaa
 abbbb ababa bbaaa b--b- a-aab aaaaa aabaa aba
EAGTAA385V           {0} ; 1522
 ----- ----- ----- ----- ----- ----- ----- ----- ----- -----
 ----- ----- ----- ----- ----- ----- ----- ----- ----a aaabb
 bbaba abbab baabb ababa bbbbb abaab aabbb babaa baaab bbaab
 baabb abbbb abaaa b--a- b-bba bbaba babba bba
EAGTAA474V           {0} ; 1527
 ----- ----- ----- ----- ----- ----- ----- ----- ----- -----
 ----- ----- ----- ----- ----- ----- ----- ----- ----a aaabb
 abaaa abbab aaaba bbaaa abbba abaab aabab bbbaa baaab baaab
 baabb abbbb bbaab b--a- b-bba aaaba babba bba
EAGTAA478G           {0} ; 1528
 ----- ----- ----- ----- ----- ----- ----- ----- ----- -----
 ----- ----- ----- ----- ----- ----- ----- ----- ----a aaaab
 abaaa abbab aaaba bbaaa abbba abaab aabab bbbaa baaab baaab
 baabb abbba bbaab b--a- b-bba aaaba babba bba
ECGTGA239V           {0} ; 1541
 aabaa bb-bb ababa -baab babba bbbaa baab- aabba baaaa babaa
 aabbb aaaba abbab bbbba ba--b ---aa babbb abaaa abbab aaaba
 babab ababa bbabb baaab abbab bbbbb bbaab babab babba aaaba
 aaaba ababb abbaa bbbaa b-aab aabba aabaa aab
ECGTGA243G           {0} ; 1542
 aabaa bb-bb ababa -baab babba bbbaa baab- aabba baaaa babaa
 aabbb aaaba abbab bbbba ba--b ---aa babbb abaaa abbab aaaba
 baaab ababa bbabb baaab abbab bbbbb bbaab bbbab babba aaaba
 aaaba ababa abbaa bbbaa b-aab aabba aabaa aab
ECGTGA244G           {0} ; 1543
 aabaa bb-bb ababa -baab babba bbbaa baaa- aabba baaaa babaa
 aabbb aaaba abbab bbbba ba--b ---aa babbb abaaa abbab aaaba
 baaab ababa bbabb baaab abbab bbbbb bbaab bbbab babba aaaba
 aaaba ababa abbaa bbbaa b-aab aabba aabaa aab

LG6:
EGAMGA127V           {0} ; 14
 abbaa ababb baaab babba abbbb bbaaa bbaaa aaaaa abaaa aaaab
 babaa abbbb bbbbb aabaa aaabb abbba bbaba bbbba abaa- -----
 ----- ----- ----- ----- ----- ----- ----- ----- ----- -----
 ----- ----- ----- ----- ----- ----- ----- ---
EGAMGA530G           {0} ; 44
 abbaa abaab baaab babba aabbb bbaaa bbaaa aaaaa abaaa aaaab
 babaa abbbb bbbbb aabaa aaabb abbba baaba bbbba abaa- -----
 ----- ----- ----- ----- ----- ----- ----- ----- ----- -----
 ----- ----- ----- ----- ----- ----- ----- ---
ECAMCG345V           {0} ; 79
 aabbb bbbba abbba babba baabb abbaa abbaa bbbbb ababa abaab
 baaab ababa abaaa bbaba aaabb babab bbaab aabba abbb- -----
```

```
 ----- ----- ----- ----- ----- ----- ----- ----- ----- -----
 ----- ----- ----- ----- ----- ----- ----- ---
ECAMCG406V          {0} ; 84
 aabaa ababb abaaa bbbba babbb abaaa baaba abbbb baaab aaaaa
 baaaa abbab bbbbb abbab bbabb abaaa bbaba abbaa bbbb- -----
 ----- ----- ----- ----- ----- ----- ----- ----- ----- -----
 ----- ----- ----- ----- ----- ----- ----- ---
ECGMCG407V          {0} ; 98
 babab ababb abaaa bbbba babbb abaaa baaba abbbb baaab aaaaa
 babab abbab bbbbb abbab bbabb abaaa bbaba abbab bbbb- -----
 ----- ----- ----- ----- ----- ----- ----- ----- ----- -----
 ----- ----- ----- ----- ----- ----- ----- ---
EGAMGC136G          {0} ; 111
 babab bbbbb ababb aabba babbb babab abbaa abbbb bbabb aaabb
 baaab aaaba abaaa baabb aaaab aabbb baaab aabbb ababb abbbb
 baaaa ababb aaaba aabba aabba ababa aabbb babbb bbbaa bbaab
 babba baaaa abaaa babaa bbaba abaaa bbaaa abb
ECAMCC408V          {0} ; 168
 abaaa ababb baabb babaa aabbb bbaaa bbaaa aaaaa abaaa aaaab
 babaa abbbb bbbbb aabaa aaabb abbba baaba bbbba -baab abaab
 abaab abbbb ababa abbab baaaa bbaba bbbbb babaa ababa babab
 aabba bbbba ababb abaab babaa aabba baaaa bbb
ECAMCC409V          {0} ; 169
 abaaa ababb baabb babaa aabbb bbaaa bbaaa aaaaa abaaa aaaab
 babaa abbbb abbbb aabaa aaabb abbba baaba bbbba -baab abaab
 abaab abbbb ababa abbab bbaaa bbaba bbbbb babaa ababa babab
 aabba bbbba ababb abaab babaa aabba baaaa bbb
EACMCC122V          {0} ; 197
 aabbb bbbba abbba babba baabb abbaa babaa bbbbb ababa abaab
 baaab ababa baaab aabab aaabb babab baaab aabbb abbbb abbba
 aabab bbbab aaaaa aabaa aaaaa aaaba baabb baabb bbbaa ababb
 baaba baaaa abaaa baabb babba bbbba baabb bab
ECAMGG92G           {0} ; 246
 babab bbaab abaab bbbab babbb abaaa abaaa abbbb baaaa aaaaa
 babab abbaa bbbba bbbaa bbabb aaaaa baaba abbab bbbbb baaba
 abbba babab abaaa bbabb baaaa aabaa baaab babba baaaa bbaaa
 baaaa bbabb abaab bbaaa baaba aaaaa babaa aab
EACMGA262V          {0} ; 357
 babbb bbaab abaab bbbab babbb abaaa babba abbbb bbaaa aaaaa
 babab abbaa abbbb bbaaa bbabb aaaaa baaba abbab bbbbb baaba
 abbb- babab bbaaa bbaba babaa aabaa baaab babba baaab bbaaa
 baaaa bbabb abaaa bbaab baaba aaaaa babaa aab
EACMTC498G          {0} ; 524
 babab bbaab abaab bbbab babbb abaaa baaaa abbbb baaaa aaaaa
 babab abbaa bbbbb abbaa bbab- aaaaa baaba abbab bbbbb baaba
 abbba babab abaaa bbabb baaaa aabaa baaab babba baaaa bbaaa
 baaaa bbabb abaab bbaaa baaba aaaaa babaa aab
ECAMCT119G          {0} ; 547
 abbab abbbb baaab bbbba babbb abaab bbaaa aabbb aaaab aaaaa
 babab bbbbb bbbbb aabab bbbb- aabaa baaba abbba bbaab aaaba
 abaaa aabbb ababa abbab baaaa ababa abbbb babba aaaaa babab
 aabaa bbbbb abaab bbaab baaaa aabbb aaaaa aab
ECAMCT119V          {0} ; 548
 abbab abbbb baaab bbbaa aabbb baaab abaaa aaabb aaaab aabbb
 babab bbbbb bbbbb aabaa bbbb- abbaa baaba abbba bbaab aaabb
 bbaba aabbb ababa abbab baaaa ababa abbbb babba aaaaa babab
 aabaa bbbbb abaab bbabb baaaa aaaab aaaaa aab
```

```
EAGMGG159G          {0} ; 675
 babbb abbab ababb aabba babab bbbbb babaa abbbb bbabb baabb
 bbaab aaaaa aaaba baabb aaaab aabbb baaab aabbb ababb ababb
 baaaa ababb aaaba aabba abbba ababa aabbb babbb bbbba bbbab
 babba baaaa bbaaa babaa bbaba bbaaa bbaaa abb
EAGMGG196G          {0} ; 679
 baabb abbab ababb aabbb babab bbbbb babaa abbba bbabb baabb
 bbaab aaaaa aaaba baabb aaaab aabbb baaab aabbb ababb ababb
 baaaa ababb aaaba aabba abbba ababa aabbb babab bbbba bbbab
 babba baaaa bbaaa babaa bbaba bbaaa bbaaa abb
ECGMGC501V          {0} ; 727
 abbab abbbb baabb babbb abbbb bbaab baaaa aabba aaaab aaaab
 babab bbbbb bbbbb aabaa baabb aabba baaba bbbba baaab aaaba
 abaaa abbbb ababa abbab aaaaa abaaa bbbbb babba aaaaa babab
 aabba abbbb ababb bbaab aaaaa aabab aaaaa bab
EACMGG73G           {0} ; 735
 abbaa abbbb baaaa babba abbbb baaaa bbaaa aaaaa abaaa a-aab
 babaa abbbb bbbbb aabaa aaabb abbba baaba bbbba abaab abaaa
 abaaa abbbb ababa abbab bbaaa ababa b-bbb babaa aaaaa babab
 aabba abbba ababb abaaa babaa aabbb baaaa bab
EACMGG88G           {0} ; 738
 aabbb bbbbb abbba babba babbb aabaa abbaa bbbbb ababa a-aab
 baaab ababa abaaa bbaaa aaaab aabbb baaaa aabba abbbb abbba
 aabab bbaaa aaaaa aabba aabaa aaaba a-bbb aaabb bbbaa ababb
 baaba baaaa abaab baaab bbabb bbaaa bbabb aab
EAGMCT123G          {0} ; 806
 abbaa ababb baabb babba aabbb bbaaa bbaaa aaaaa abaaa a-aab
 babaa abbbb bbbbb aabaa aaabb abbba aaaba bbbba abaab abaaa
 abaab abbbb ababa abbab bbaaa ababa b-bbb babaa abaaa babab
 aabba bbbbb ababb abaaa babaa aabba baaaa bab
EAGMCT246V          {0} ; 823
 baabb abbab ababb aabbb babab bbbbb abbaa abbba bbabb b-abb
 bbaab aaaaa aaaba baabb aaaab aabbb baaab aabbb ababb ababb
 baaaa ababb aaaba aabba abbba ababa a-bbb babab bbbba bbbab
 babba baaaa bbaaa babba bbaba bbaaa bbaaa abb
ECGMGT84G           {0} ; 846
 abbab aaabb baaab babba aabbb bbaaa bbaaa aaaaa bbaaa a-aab
 aabbb ababb bbbbb aabaa aaaab abbbb baaba bbbba aaaab baaaa
 abaaa babbb bbaba abbbb bbaaa ababa b-bbb aabaa abaaa babab
 aabba bbbba ababb abaaa aabaa aabba baaaa bab
EACMGC84V           {0} ; 872
 babab bbaab abbab bbbab babba abaaa babaa abbbb bbaba aaaaa
 aabab abbaa abbbb bbaaa bbabb aaaaa baaba abbab babbb babba
 ababa bbbab bbaaa bbaba babaa aabaa baaab babba abbaa bbaab
 abaaa ababa aaaab ababb baaab baaab babba baa
EACMGC86G           {0} ; 873
 babab bbaab abbab bbbab babba abaaa babaa abbbb bbaba aaaaa
 aabab abbaa abbbb bbaaa bbabb aaaaa baaba abbab babbb babba
 ababa bbbab bbaaa bbaba babaa aabaa baaab babba abbaa bbaab
 abaaa ababa aaabb ababa baaab baaab babba baa
EACMGC194G          {0} ; 884
 baabb abbab abbab aabbb babab bbbbb babaa abbba bbabb baabb
 bbaab aaaaa aaaba baabb aaaab aabbb baaab aabbb ababb ababb
 baaaa ababb aaaba aabba abbba ababa aabbb babab ababa abbab
 abbaa abbaa aabab bbbaa bbabb abbab aaaab baa
EACMGC387G          {0} ; 914
 aabbb bbbab abbbb bbbbb babba abaaa babbb abbbb bbaba aaaaa
```

```
 aabab baaba abbbb bbaba baabb aaaab baabb abbaa babbb babba
 bbabb bbbab abaaa bbbaa babaa aabaa baaab baaba abbab bbabb
 abaaa ababa abbab ababa baabb baaba babba baa
EAGMCA127G           {0} ; 938
 babab ababb abaaa bbbba babbb abaaa abaaa abbbb baaab aaaaa
 babab abbab bbbbb abbab bbabb abaaa baaba abbab bbbbb aaaba
 ababa babab ababa abbbb baaaa abbba aabab bbbba bbbaa bbaab
 aaaaa abbaa aaabb aaaaa aabaa abaab bbbba baa
ECGMCA243G           {0} ; 987
 abbaa abbbb babab babbb abbbb bbaaa abaaa aabba aaaaa aaaab
 babab bbbbb bbbbb aabaa baabb abbba baaba bbbba bbaab aaaaa
 abaaa abbbb ababa abbab bbaaa ababa bbbbb babaa bbbaa abaaa
 aaabb baaaa ababa aaaaa ababa abbaa bbbba bab
ECGMCA245V           {0} ; 988
 abbaa abbbb babab babbb abbbb bbaaa ababa aabba aaaaa aaaab
 babab bbbbb bbbbb aabaa baabb abbba baaba bbbba bbaab aaaba
 abaaa abbbb ababa abbab bbaaa ababa bbbbb babaa bbbaa abaaa
 aaabb baaaa ababa aaaab ababa abbaa bbbba bab
EACMGT39G            {0} ; 999
 aabbb bbbbb abbba babba babbb aabaa abbaa bbbbb ababa abaab
 baaab ababa abaaa bbaaa aaaab aabbb baaaa aabba abbbb abbba
 aabab bbaaa aaaaa aabba aabaa aaaba aaabb aaabb bbbaa ababb
 baaba baaaa abaab baaba bbabb bbaaa bbabb aab
EACMGT89V            {0} ; 1008
 abbaa ababb baabb babba aabbb bbaaa bbaaa aaaaa abaaa aaaab
 babaa abbbb bbbbb aabaa aaabb abbba baaba bbbba abaab abaaa
 abaab abbbb ababa abbab bbaaa ababa bbbbb babaa abaaa babab
 aabba bbbbb ababb abaab babaa aabba baaaa bab
EACMGT149G           {0} ; 1016
 aabbb bbbaa abbba bbbba baabb abbaa aabbb abbbb ababa aaaab
 aabab ababa abaab bbaba baabb baaab baaab abbba babbb aabba
 aaaab bbbab aaaaa aabaa aaaaa aabba baabb baabb babaa ababb
 baaba bbaab abaaa bbaba babba babba baabb bab
EACMGT400V           {0} ; 1037
 aabbb bbbaa abaaa bbbba baabb abbaa aabbb aabbb bbaba aaaab
 aabab ababa abaab bbabb baabb babab baaab abbba babbb aabba
 aaaab bbbab aaaaa aabaa aaaaa aabba baabb baabb babaa ababb
 baaba bbaab abaaa bbabb babba babba baabb baa
EAGMTT222G           {0} ; 1065
 baaba abbab abaab aabbb babab bbbbb babba abbba bbabb baabb
 bbaab aaaaa aaaba baabb aaaab aabbb baaab aabbb ababb aabbb
 baaaa ababb aaaba aabba abbba ababa aabbb babab bbbba bbbab
 babba baaaa bbaaa babaa bbaba bbaaa bbaaa abb
EAGMTT443G           {0} ; 1080
 aabbb bbbbb abbba babba baabb aabaa abbaa bbbbb ababa abaab
 baaab ababa abaaa bbaba aaabb aabbb baaaa aabba abbbb abbba
 aabab bbaaa aaaaa aabba aaaaa aaaba aaabb baabb bbbaa ababb
 baaba baaaa abaaa baaba babba bbaaa baabb aab
EAGMTT466bV          {0} ; 1082
 baaab abbab ababb aabbb babab bbbbb babaa abbba bbabb baabb
 bbaab aaaaa aaaba baabb aaaab aabbb baaab aabbb ababb aabbb
 baaaa ababb aaaba aabba abbba ababa aabbb babab bbbba bbbab
 babba baaaa bbaaa babab bbaba bbaaa bbaaa abb
ECGMGG544G           {0} ; 1109
 babab ababb abaaa bbbba babbb abaaa ababa abbbb baaab aaaaa
 babab abbab bbbbb abbab bbabb abaaa baaba abbab bbbbb aaa-b
 ababb babab ababa abbbb baaaa abbba aabab bbbba aaaaa aaaaa
```

aabaa bbbbb abaab bbaaa baaba aaaab bbaaa aab
EACTCC123V {0} ; 1124
aabbb bbbba abbba babba babbb aabaa abbaa bbbbb ababa abaab
baaab ababa abaaa bbaaa aaaab aabbb baaaa aabba aabbb abbba
aabab bbaaa aaaaa aabba aabaa aaaba aaabb aaabb bbbaa ababb
baaba baaaa abaab baabb bbabb baaaa bbabb aaa
EAGTTA162G {0} ; 1161
aaaba bbbba abbba babba baabb abbaa abbaa bbbbb ababa abaab
baaab ababa abaaa bbaba aaabb babab baaab aabba abbbb abbba
aaaab bbbab aaaaa aabaa aaaaa aaaba baabb baabb bbbaa aaabb
baaba baaaa aaaaa baabb babba bbbaa baabb bab
EAGTTA428G {0} ; 1169
abbaa abbbb baabb babbb abbbb bbaab abaaa aabba aaaab aaaab
baabb abbbb bbbbb aabba baabb aabba baabb abbba bbbab aaaba
aaaaa abbbb ababb abbab bbaaa ababa bbbbb babba aabab bbbab
aabba abbbb ababb bbaaa bbaaa aabab aaaaa bab
EAGTTA448G {0} ; 1173
aabbb bbbba abbba babba baabb abbaa abbba bbbbb ababa abaab
baaab ababa abaab bbaba aaabb babab baaab aabba abbbb abbba
aaaab bbbab aaaaa aabaa aaaaa aabba baabb baabb bbbaa ababb
baaba baaaa abaaa baabb babba bbbaa baabb bab
EAGTTA459V {0} ; 1174
aabbb bbbba abbba babba baabb abbaa abbba bbbbb ababa abaab
baaab ababa abaab bbaba aaabb babab baaab aabba abbbb abbba
aabab bbbab aaaaa aabaa aaaaa aabba baabb baabb bbbaa ababb
baaba baaaa abaaa baabb babba bbbaa baabb bab
ECGTGT164V {0} ; 1190
aabbb bbbbb abbba babba babbb aabaa abbaa bbbbb ababb abaab
babab ababa abaaa bbabb aaaab abbbb baaaa babba abbbb abbba
aabab bbaaa aaaaa aabba aabaa aaaba aaabb aaabb bbbaa ababb
baaba baaaa ababb baabb bbabb bbaaa bbabb aab
EACTAG129V {0} ; 1235
baabb abbab abaab aabba babab bbbbb babaa abbba bbabb baabb
bbaab aaaaa aaaba baabb aaaab aabbb baaab aabbb ababb ababb
bbaaa ababb aaaba aabba abbb- ababa aabbb babab bbbba bbbab
babba baaaa bbaaa babaa bbaba bbaba bbaaa abb
EACTAG358V {0} ; 1266
abbaa abbbb baabb babba abbbb bbaab abaaa aaaba abaaa aaaab
baabb bbbbb bbbbb aabaa baabb abbab baaba bbbba bbaab aaaaa
abaaa abbbb ababa abbab bbaa- ababa bbbbb babaa aaaaa babab
aabba abbbb ababb bbaaa baaaa aabbb aaaaa bab
EACTAG358G {0} ; 1267
abbaa abbbb baaab babba abbbb bbaaa bbaaa aaaaa abaaa aaaab
baabb bbbbb bbbbb aabaa baabb abbab baaba bbbba bbaab abaaa
aaaaa abbbb ababa abbab bbaa- ababa bbbbb babaa aaaaa babab
aabba abbbb ababb abaaa baaaa aabbb aaaaa bab
EACTAG381V {0} ; 1272
abbab abbbb baabb bbbba aabbb ababb ababa aabba aaaaa aaaaa
baabb bbbbb bbbbb aabaa bbabb abbaa baaba abbba bbaab aaaba
abaaa aabbb ababa abbab baaa- ababa bbabb babba aaaaa babab
aabaa abbbb abaab bbaaa baaaa aabab aaaaa bab
EAGTCA102V {0} ; 1286
aabab bbbab ababb bbbbb babba abaaa babba abbbb bbaba aaaaa
aaabb baaba abbbb bbaba bbabb aaaab baabb abbab babab babba
bbabb bbbab abaaa bbbaa baba- aabaa baaab baaba babab bbaaa
bbaab ababb abaaa bbabb baaba baaaa babaa bab
ECGTAC279G {0} ; 1332

```
 aabbb bbbaa abbba bbbba baabb abbaa aabbb abbbb ababa aaaab
 aaabb ababa abaab bbaba baabb baaab baaab abbba babbb aabba
 aaaab bbbab aaaaa aabaa aaaa- aabba baabb baabb babaa ababb
 baaba bbaab abaaa bbabb babba babaa baabb bab
ECGTAC283V          {0} ; 1334
 aabbb bbbaa abbba bbbba baabb abbaa aabbb abbbb ababa aaaab
 aaabb ababa abaab bbaba baabb bbaab baaab abbba babbb aabba
 abaab bbbab aaaab babaa aaab- aabba baabb baabb babaa ababb
 baaba bbaab abaaa bbabb babba babaa baabb bab
EAGTGC200V          {0} ; 1397
 abbaa abbbb baabb babbb abbbb bbaab ababa aabba aaaaa aaaab
 baab- bbbbb bbbbb aa-aa baabb a-bba baaba bbbba bbaab aaaba
 abbaa abbbb ababa abbab bbaaa ababa bbbbb babaa aaaaa babab
 aabba abbbb ababb bbaaa baaaa aabab aaaaa bab
EAGTGC345G          {0} ; 1407
 aaaba bbbaa abbba bbbba baaab abaaa aaabb aabaa bbaba aaaab
 aaab- ababa abaab bb-ba baabb b-aab baaab abbba babbb aabba
 aaaab bbbab aaaaa aabaa aaaaa aabba baabb baaaa babaa ababb
 baaaa bbaab abaaa bbabb babba babaa baabb bab
EAGTGC346V          {0} ; 1408
 aabbb bbbaa abbba bbbba baabb abbaa aabbb abbbb bbaba aaaab
 aaab- ababa abaab bb-ba baabb b-aab baaab abbba babbb aabba
 aabab bbbab aaaaa aabaa aaaaa aabba baabb baaaa babaa ababb
 baaaa bbaab abaaa bbabb aabba babaa baabb aab
EACTTC213V          {0} ; 1454
 abbaa ab-bb baabb -bbbb abbbb bbaab aba-- aabba aaaab aaaab
 baabb bbbbb bbbbb aabaa ba--b ---ba baaba bbbba bbaab aaaba
 abbaa abbbb ababa abbab bbaaa ababa bbbbb babba aaaaa babab
 aabba abbbb ababb bbaaa b-aaa aabab aaaaa bab
EACTTC395G          {0} ; 1472
 babab bb-ab abaab -bbba babbb ababa baaa- abbbb baabb baaaa
 baabb ababa bbbbb abbab bb--b ---aa baaba abbab bbbbb baaba
 ababa babab abbaa bbabb baaaa abbaa aaaab bbbba aaaaa baaaa
 babaa bbabb abaab bbaab b-aba aaaaa babaa aab
EACTTC451G          {0} ; 1482
 baabb bb-bb ababb -abba babbb babab abba- abbbb bbabb aaabb
 baaab abaaa aaaaa baabb aa--b ---bb baaab aabbb ababb abbab
 baaaa ababb aaaba aabba aabaa ababa aabbb babbb bbbaa bbaab
 babba baaaa abaaa babaa b-aba bbaba bbaaa abb
EACTTC452V          {0} ; 1484
 baabb bb-bb ababb -abba babbb babab abba- abbbb bbabb aaabb
 baaab abbaa abaaa baabb aa--b ---bb baaab aabbb ababb abbbb
 babaa ababb aaaba aabba aabaa ababa aabbb babbb bbbaa bbaab
 babba baaaa abaaa babaa b-aba bbaba bbaaa abb
EACTTC548G          {0} ; 1494
 baabb ab-ab ababb -abbb babab bbbbb baba- abbba bbabb baabb
 bbaab aaaba aaaba baabb aa--b ---bb baaab aabbb ababb ababb
 baaaa ababb aaaba aabba abbba ababa aabbb babab bbbba bbbab
 babba baaaa bbaaa babaa b-aba bbaba bbaaa abb
EAGTAA154V          {0} ; 1504
 babab ab-bb abaaa -bbba babb- abaaa baaa- abb-b ba-ab a-aaa
 -aabb abbab b-bbb abbab bb--b ----a baa-a -a--- ----b aaaba
 ababa babab ababa abbbb baaaa abbba aabab bbbba aaaaa aaaaa
 aabaa bbbbb abaab b--a- b-aba aaaaa bbaaa aab
EAGTAA167G          {0} ; 1506
 babab bb-ab abaab -bbab babb- abaaa baba- abb-b bb-aa a-aaa
 -aabb abbaa a-bbb bbaaa bb--b ----a baa-a -b--- ----b baaaa
```

```
 abbba babab bbaaa bbaba babaa aabaa baaab babba baaab bbaaa
 baaaa bbabb abaaa b--a- b-aba aaaaa babaa aab
EAGTAA224V          {0} ; 1512
 abbaa ab-bb baabb -bbba aabb- bbaaa bbaa- aaa-a ab-aa a-aab
 -aabb abbbb b-bbb aabaa aa--b ----b baa-b -b--- ----b ababa
 abaab abbbb abaaa abbab baaaa ababa bbbbb baaaa abaaa aabab
 aabaa bbbba aaabb a--a- b-baa aabba baaaa aab
ECGTGA435V          {0} ; 1563
 abbaa ab-bb baabb -abba aabbb bbaaa bbab- aaaaa abaaa aaaab
 baaba abbbb bbbbb aabaa aa--b ---ba baaba bbbba abbab abaaa
 abbab abbbb ababa abbab bbaaa ababa bbbbb babaa abaaa babab
 aabba bbbbb ababb abaaa b-baa aabba baaaa bab
SSR_1184            {0} ; 1618
 abbaa aaabb baaab babba aabbb bbaaa bbaaa aaaba bbabb bbbab
 aabbb ababb babbb aabab baaab abbab baaba babba aaaab bbaaa
 abaaa babbb bbaba ababa bbaaa ababa bbbbb aabaa abaaa babab
 aabaa bbbab abab- abbab aabaa aabba baaaa bab

LG7:
EGAMGA86G           {0} ; 7
 babab bbbba bbaba babbb abbaa baaab ababb aabbb aaabb abaaa
 bbbab bbbaa bbabb abbba babaa babbb baaab aaaaa bbbb- -----
 ----- ----- ----- ----- ----- ----- ----- ----- ----- -----
 ----- ----- ----- ----- ----- ----- ----- ---
EGAMGA232G          {0} ; 23
 babab bbbaa bbaba babbb abbaa babab abaab aabbb aaabb abaaa
 bbbab bbbaa bbabb abbba babaa bbbbb baaab aaaaa bbbb- -----
 ----- ----- ----- ----- ----- ----- ----- ----- ----- -----
 ----- ----- ----- ----- ----- ----- ----- ---
EGAMGA520V          {0} ; 41
 baaba abbaa baaba babbb abbab babbb baaab aabbb abbab abbbb
 abbaa abbaa bbaba aaaba aaaaa baabb abbaa aaaab abab- -----
 ----- ----- ----- ----- ----- ----- ----- ----- ----- -----
 ----- ----- ----- ----- ----- ----- ----- ---
ECAMCG56G           {0} ; 49
 babab babbb babba baabb abaaa baaba abbbb ababb aabbb bbaaa
 bbaaa bbabb aabbb abbba baaaa babbb baaab bbbba babb- -----
 ----- ----- ----- ----- ----- ----- ----- ----- ----- -----
 ----- ----- ----- ----- ----- ----- ----- ---
ECAMCG57V           {0} ; 50
 babab babbb babba baabb abaaa baaba abbbb ababb aabbb bbaaa
 bbaaa bbabb aabbb abbba baaaa babbb bbaab bbbba babb- -----
 ----- ----- ----- ----- ----- ----- ----- ----- ----- -----
 ----- ----- ----- ----- ----- ----- ----- ---
ECAMCG60G           {0} ; 51
 baaba abbaa baaba babbb abbab babbb baaab aabbb abbab abbbb
 abbaa abbaa bbaba aaaba aaaaa baabb aabaa aaaaa abab- -----
 ----- ----- ----- ----- ----- ----- ----- ----- ----- -----
 ----- ----- ----- ----- ----- ----- ----- ---
ECGMCG121G          {0} ; 91
 babab aabbb babba baabb abaaa baaba babaa bbabb babaa baaab
 abbba bbaaa abbbb aaaab bbaaa bbabb baaaa bbaba babb- -----
 ----- ----- ----- ----- ----- ----- ----- ----- ----- -----
 ----- ----- ----- ----- ----- ----- ----- ---
ECAMCC93G           {0} ; 139
 ababa aaabb baabb baaba aaaaa aabaa baabb babab abbab bbbbb
 baaba babba bbbaa baabb aaaab baaba baaaa abbab -baaa babab
```

```
 baaab babaa bbbaa bbbab baaaa baaab bbabb bbabb aabba baabb
 bbaba aabaa babab aabaa baaba aaaab bbaab bba
ECGMCC475G           {0} ; 192
 babaa abbaa baaba babbb abbab babbb baaab abbbb abbab abbbb
 abbaa abbaa bbaba aaaba aaaaa baabb aabaa aaaaa bbaba aabab
 abaab aba-a bbbba baaab baaaa abaaa babaa abbbb aabbb baaaa
 aabaa baabb abbab abbaa abbbb aabab babaa bbb
EACMCC149G           {0} ; 202
 babba abbaa ababa babbb abbaa babab abaab aabbb aaaab ababb
 abbaa bbbaa bbbaa abbab babaa babbb aaaaa aaaaa abbba babab
 bbaab abaaa bbbbb baaaa baaaa abaaa babaa ababb babbb bbaab
 bbaaa baaba aabbb abaaa abbbb aaaab aaaaa abb
ECAMGG577V           {0} ; 296
 baaba abbaa baaba babbb abbab babbb abaab aabbb abbab abbbb
 abbaa abbaa bbbaa aaaab aaaaa baabb aabaa aaaaa abaaa aabab
 abaab abbaa bbbba baaab baaaa abaaa babba abbbb aabbb baaaa
 abaaa baabb aabab abaab abbbb aaaab aabaa abb
ECGMTA167G           {0} ; 310
 babba abbaa ababa babbb abbaa babab abaab aabbb aaaab ababb
 abbaa bbbab bbbaa abbab aabaa baaab aaaaa aaaaa abbba babab
 bbaab abaaa bbbbb baaaa baaaa abaaa babaa abbbb babbb bbaab
 bbabb baabb aabbb abaaa abbbb aaaab aaaaa abb
EACMGA358G           {0} ; 370
 babbb aabbb babaa baabb abaaa baaba aabab bbabb aabba abaab
 abbaa bbabb aabbb abbaa aaaaa bbabb babaa bbbba baaba aabbb
 baba- baabb baaab bbaaa aabba abaab babba abbbb bbbba bbaba
 babab babba abbba aabba ababa baaab baaba aba
EAGMTA319G           {0} ; 416
 baaba abbaa baaba babab abbab babbb baaab aabbb abbab abbbb
 abbaa abbaa bbaba a-aba aaaaa baabb aabaa aaaaa ababa aabab
 abaa- abaaa bbbba baaab baaaa abaaa babba abbbb aabbb baaaa
 abaaa baabb aabab abaaa abbbb aaaab aabaa abb
EACMTC107G           {0} ; 453
 aaaaa bbaba bbabb baaba aaaaa baaab aaabb babab bbbab bbbba
 aabaa babaa bbbba baabb aaaa- aabbb aaaaa abbab ababb babab
 babab babbb bbbaa bbbab baaab abaab bbabb bbabb aabaa aaabb
 bbaaa bbbab babbb abaab bbaba baaab ababa bba
EACMTC527V           {0} ; 529
 babba abbaa ababa babbb abbaa baaab baabb aabbb aaaab ababb
 abbaa bbbaa bbaba abbba baba- babbb aaaaa aaaab abbba babab
 bbaab abaaa bbbbb baaaa baaaa abaaa babaa ababb babbb bbaab
 bbaaa baabb aabbb ababa abbbb aaaab aaaaa abb
ECAMCT132V           {0} ; 552
 aaabb abaaa bbaba babbb bbaab baabb babbb aabab abbab abbaa
 abbaa bbbaa bbbba bbabb aaaa- baabb aaaaa abbab bbbbb aabab
 aabab babba babaa bbaab baaaa abaab baabb bbaba aabaa aaaaa
 bbaba bbbbb aabab ababa abbba baaab aabaa aba
ECGMTT278V           {0} ; 616
 babaa abbaa bba-a babbb abbaa babab ababb aabbb aaaab ababb
 bbbaa bbbaa bbaba abbba bab-- babab aaaaa aaaaa bbbba babab
 bbaab abbaa bbbbb baaaa baaba abaaa babaa ababb babbb bbaab
 bbaab babbb babbb abbaa abbbb aaaab aaaaa abb
EACMCG69G            {0} ; 632
 babab bbbaa bbaba babbb abbaa babab baabb aabbb aaabb abaaa
 bbbab bbbaa bbabb abbba babaa babbb bbaab aaaaa bbbba babbb
 bbaab aaaba babab baaaa aaaba abbaa babaa ababb bbbbb bbaab
 babab babba babbb abaaa abaab aaaab baaaa bab
```

EACMCG201G {0} ; 639
babaa aabbb babba babbb abaaa aaabb abbba baabb bbaaa baaab
abbba abbaa aabbb aaaab bbaaa bbabb baaaa bbaba babba aabba
babba baaab abbbb bbaaa abbba abaab babba abbab bbbbb abbba
bbbba bbaba baaba aabba baaba aaaab bbbba aaa
EACMCG202V {0} ; 640
babab aabbb babba babbb abaaa aaabb abbba bbabb bbaaa baaab
abbba abbaa aabbb aaaab bbaaa bbabb baaaa bbabb babba aabba
babba babab abbbb bbaaa abbba abaab babba abbab bbbbb abbba
bbbba bbaba baaba aabbb baaba aaaab bbbba aaa
EACMCG220V {0} ; 642
babaa aabbb babba babbb abaaa aaabb abbba bbabb bbaaa baaab
abbba abbaa aabbb aaaab bbaaa bbabb baaaa bbaba babba aabba
babba baaab abbbb bbaaa abbba abaab babba abbab bbbbb abbba
bbbba bbaba baaba aabbb baaba aaaab bbbba aaa
EACMCG342G {0} ; 654
baaba abbaa baaba babbb abbab babbb abaab aabbb abbab abbbb
abbaa abbaa bbaba aaaba aaaaa baabb aabaa aaaaa ababa aabab
abaab abaaa bbbba baaab baaaa abaaa babba abbbb aabbb baaaa
aaaaa baabb aabab abaaa abbbb aaaab aabaa abb
EAGMGG277G {0} ; 691
babab babbb babba baabb abaaa baaba babba bbabb babba baaab
abbba bbaab aabbb aaaaa bbaaa bbabb baaaa bbbba babaa aabbb
babba babbb aabab bbaaa aabba abaab babba abbbb bbbbb bbaba
babab bbaba baaba aabba ababa baaab ababa aba
EAGMCT99V {0} ; 802
aaaba abaaa bbaba baaba abaaa baabb abbbb babab bbbab b-bba
aabaa bbbab bbbba bbabb aaaab aabbb aaaaa aabab abbbb babab
babab babba babaa bbbab bbaaa abaab b-abb bbaba aaaaa aaaab
bbaba bbbbb babbb ababb abbba baaab aabaa bba
ECGMGT131G {0} ; 850
babba bbbaa baaba babbb abbab babbb abaab aabbb abbab a-bbb
abbaa abbaa bbaba aaaba aaaaa baabb aabaa aaaaa ababa aabab
abaab abbaa bbbba baaab baaaa abaaa b-bba abbbb aabbb baaaa
aaaaa baabb aabab abaaa abbbb aaaab aabaa abb
EACMGC198G {1} ; 885
ababb bbaaa abaab abbaa babbb abbab abaab aabaa ababb abbba
baaab aabbb bbaaa bbbba aabbb aabaa abbbb aabab abaab bbaaa
abaab ababa baaaa aabbb bbaab babba abaab baaba bbbaa ababa
babbb aabba bbbba aaaaa ababa aaaba ababa bba
EACMGC347V {0} ; 909
babab bbbba bbbaa babbb abbaa baaab ababb aabbb aaabb abaaa
bbbab bbbaa bbabb abbba babaa babbb bbaab aaaaa bbbba babbb
bbaab aabba babab baaaa aaaba abbaa babaa ababb babaa aabaa
baaaa abaaa ababb bbbbb baabb ababb abbab aba
EAGMCA283G {0} ; 960
babaa aabbb babba babbb abaaa aaabb abbba bbabb bbaaa baaab
abbba abbaa aabbb aaaab bbaaa bbabb baaaa bbaba babba aabba
babba baaab abbbb bbaaa abbba abaab babba abbab aaabb bbaab
aaaaa bbbbb aaaab bbbba bbbab bbbab babab aab
EAGMCA461V {0} ; 969
aaaaa bbaba bbbab baaba aaaaa babab aaabb babab bbbab bbbba
aabaa babaa bbbba baabb aaaab aabbb aaaaa abbab ababb babab
babab babbb bbbaa bbbab baaab abaab bbabb bbabb babaa bbbab
abaaa babab abbaa abaab aabbb baaab bbabb abb
EAGMCA487G {0} ; 972
aaaaa bbaba bbbab baaba aaaaa babab ababb babab bbbab bbbba

```
 aabaa babaa bbbba baabb baaab aabbb aaaaa abbab abbbb babab
 babab babbb bbbaa bbbab baaab abaab bbabb bbabb babaa bbbab
 abaaa babab abbaa abaaa aabbb baaab bbabb abb
ECGMCA227G          {0} ; 986
 aaaba abaaa bbbaa babbb bbaab baabb babbb aabab abbab abbaa
 abbaa bbbaa abbba bbabb aaaaa bbbbb aaaaa abbab bbbbb aabaa
 aabab babaa babaa bbaab baaaa abaab baabb bbaba babaa aabbb
 abaaa baaba aabaa abaaa aaaab babab bbbba aba
EAGMTT173V          {0} ; 1060
 babaa aabbb babba babbb abaaa aaabb babba bbabb bbaaa baaab
 abbba abbaa aabbb aaaab bbaaa bbabb baaaa bbabb babba ababa
 babba baaab abbbb bbaaa abbba abaab babba abbab bbbbb abbba
 bbbba bbaba baaba aabbb baaba aaaab bbbba aaa
EAGMTT413V          {0} ; 1078
 babba abbaa baaba babbb abbab babbb baaab aabbb abbab abbbb
 abbaa abbaa bbaba aaabb aaaaa baabb aabaa baaab ababa abaab
 abaab abbaa abbba baaab babaa abaaa babaa abbbb aabbb babaa
 aaaaa baabb aabab abaab abbbb baaab aabaa abb
EAGMTT430V          {0} ; 1079
 aaaba ababa bbaaa baaba aaaaa baaab baabb babab bbbab bbbba
 aabaa bbbaa bbbba bbabb aaaab aabbb aaaaa abbab abbbb bbaab
 babab babbb abbaa bbbab baaaa abaab baabb bbaba aabaa aaaab
 bbaaa bbbbb aabbb abaab abbba baaab aabaa bba
EAGTTA125G          {0} ; 1160
 babab aabbb babba babbb abaaa aaabb babba bbabb bbaaa baaab
 abbba abbaa aabbb aaaab bbaaa bbabb baaaa bbaba babba aabba
 baaba baaab abbbb bbaaa abbba abaab babba abbab bbbbb abbba
 bbbba bbaba baaba aabbb baaba aaaab bbbba aaa
EAGTTA336G          {0} ; 1168
 aaaba abbaa bbaba babbb abaab baabb baaab aabab abbab abbbb
 ababa abbaa bbbba baaba aaaaa baabb aabaa abaaa bbbbb aabab
 aaaab abbba babaa baaab baaaa abaaa babba ababb aabaa baaaa
 bbaba baabb aabab abaaa abbbb aaaab aabaa aba
ECGTGT242G          {0} ; 1202
 babaa aabbb babba babbb abaaa aaabb babba bbabb bbaaa baaab
 abbba abbaa aabbb aaaab bbaaa bbabb baaaa bbaba baaba aabba
 baaba baaab abbbb bbaaa abbba abaab babba abbab bbbbb abbba
 bbbba bbaba baaba aabbb baaba aaaab bbbba aaa
ECGTGT243G          {0} ; 1203
 babab aabbb bbbba babbb abaaa aaabb babba bbabb bbaaa baaab
 abbba abbaa aabbb aaaab bbaaa bbabb baaaa bbaba baaba aabba
 baaba baaab abbbb bbaaa abbba bbaab babba abbab bbbbb bbbba
 bbbba bbaba baaba aabbb baaba aaaaa bbbba bab
ECGTGT303G          {0} ; 1211
 baaba abbaa baaba babbb abbab babbb baaab aabbb abbab abbbb
 ababa abbaa bbaba aaaba aaaaa baabb aabaa aaaaa abbba aabab
 abaab abaaa bbbba baaab baaaa abaaa babaa abbbb aabbb baaaa
 aaaaa baabb aabab abaaa abbbb aaaab aabaa abb
ECGTGT305G          {0} ; 1213
 baaba abbaa baaba babbb abbab babbb baaab aabbb abbab abbbb
 ababa abbaa bbaba aaaba aaaaa baabb aabaa baaaa abbba aabab
 abaab abaaa bbbba baaab baaaa abaaa babaa abbbb aabbb baaaa
 aaaaa baabb aabab abaaa abbbb aaaab aabaa abb
EACTAG333G          {0} ; 1261
 abbaa baaba bbaab baaba aaaaa aabab aaabb babab bbbab bbbba
 baaba babba bbbba baabb aaaab aabbb aaaaa aabab abaaa babbb
 babab babbb bbbaa bbbab baaa- aaaab bbabb bbabb aabaa aaabb
```

bbaaa bbbab babab aabab bbaba baaab ababa bba
EACTAG334V {0} ; 1262
abbaa baaba bbaab baaba aaaaa aabab aaabb babab bbbab bbbba
baaba babba bbbba baabb aaaab aabbb aaaaa aabab abaaa babbb
bbbab babbb bbbaa bbbab baaa- aaaab bbabb bbabb aabaa aaabb
bbaaa bbbab babab aabab bbaba baaab ababa bba
EAGTCA96V {0} ; 1285
babba abbaa abaaa babbb abbaa babab baabb aaabb aaaab ababb
bbaba bbbaa bbaba abbba babaa baabb aaaaa aaaaa abbba babab
bbaab abbaa bbbbb baaaa baab- abaaa babaa ababb aabbb bbaab
bbaaa babbb babbb abaaa abbbb aaaab aaaaa abb
EAGTCA292G {0} ; 1307
babba abbaa abaaa babbb abbaa babab baaab aabbb aaaab ababb
ababa bbbaa bbaba abbba babaa baabb aaaaa aaaaa abbba babab
baaab abaaa bbbbb baaaa baaa- abaaa babaa ababb babbb bbaab
bbaaa baaba aabbb abaaa abbbb aaaab aaaaa abb
EAGTCA293V {0} ; 1308
babba abbaa ababa babbb abbaa babab baabb aabbb aaaab ababb
ababa bbbaa bbaba abbba babaa baabb aaaaa aaaab abbba babab
bbaab abaaa bbbbb baaaa baaa- abaaa babaa ababb babbb bbaab
bbaaa baabb aabbb abaaa abbbb aaaab aaaaa abb
EAGTCA496G {0} ; 1319
babab aabbb babba baabb abaaa baaba babaa bbabb babaa baabb
abbba bbaaa aabbb aaaab bbaaa bbabb baaaa bbaba babba aabbb
babba babab abbbb bbaaa aabb- ababb bbbba abbab bbbbb ababa
bbbba bbaba baaba aabbb ababa baaab baaba aaa
ECGTAC209G {0} ; 1329
babba abbaa abaaa babbb abbab babab baabb aabbb aaaab ababb
ababa bbbaa bbaba abbba aabaa baabb aaaaa aaaaa abbba babab
baaab abaaa bbbbb baaaa baaa- abaaa babaa abbbb babbb bbaab
bbaaa baabb aabbb abaaa abbbb aaaab aaaaa abb
EACTGA536V {0} ; 1384
babab bbbba bbaba babbb abbaa baaab ababb aabbb aaabb abaaa
bbab- bbbaa bbabb ab-ba babaa b-bbb bbaab aaaaa bbbba babbb
bbbab aabba babab baaaa aaaba abbaa babaa ababb bbbbb bbaab
babab babba babbb abaaa abaab aaaab baaaa bab
EACTGA537G {0} ; 1385
babab bbbba bbaba babbb abbaa baaab ababb aabbb aaabb abaaa
bbab- bbbaa bbabb ab-ba babaa b-bbb bbaab aaaaa bbbba babbb
bbaab aabba babab baaaa aaaba abbaa babaa ababb bbbbb bbaab
babab babba babbb abaaa abaab aaaab baaaa bab
EAGTGC68G {0} ; 1388
babab bbbbb baaba baabb abbaa baaaa abbbb ababb aaabb bbaaa
bbaa- bbabb aaabb ab-ba babaa b-bbb baaab abbaa babaa babbb
bbaaa aabbb baaab bbaba aabba abaaa babaa abbbb bbbba bbaba
babbb babba babbb aaaba abaaa baaab baabb aba
ECGTCA183V {0} ; 1427
babaa abbaa ababa babbb abbaa babab baabb abbbb aaaab ababb
bbab- bbbaa bbaba ab-ba babaa b-bbb aaaaa aaaaa bbbba babab
bbbab abbaa bbbbb baaaa baaba abbaa babaa abbbb babbb bbaab
bbbab babbb babbb abaaa bbbbb aaaab abaaa abb
ECGTCA236V {0} ; 1429
babab abbaa ababa babbb abbaa babab baabb aabbb aaaab ababb
bbab- bbbaa bbaba ab-ba aabaa b-bbb aaaaa aaaaa bbbba babab
bbbab abbaa bbbbb baaaa baaba aaaaa aabaa ababb babbb bbaab
bbaab aabbb babbb abaaa abbbb aaaab aaaaa abb
EACTTC70V {0} ; 1440

```
 babab bb-ba bbaba -bbbb abbaa baaab abab- babbb aaabb abaaa
 bbabb bbaba bbabb abbba ba--a ---bb bbaab aaaaa bbbba babbb
 bbbab aabba babab baaaa aaaba abbaa babaa ababb bbbbb bbaab
 babbb babba babbb abaaa a-aab aaabb baaaa bab
EACTTC133V           {0} ; 1449
 babaa aa-bb babba -bbbb abaaa aaabb babb- bbabb bbaaa baaab
 abbba ababa aabbb aaaab bb--a ---bb baaaa bbaba babba aabba
 babba baaab abbbb bbaaa abbba abaab babba abbab bbbbb abbba
 bbbba bbaba baaba aabbb b-aba aaaab bbbba aaa
EACTTC133G           {0} ; 1450
 babaa aa-bb babba -bbbb abaaa aaabb babb- bbabb bbaaa baaab
 abbba ababa aabbb aaaab bb--a ---bb baaaa bbaba babba aabba
 baaba baaab abbbb bbaaa abbba abaab babba abbab bbbbb abbba
 bbbba bbaba baaba aabbb b-aba aaaab bbbba aaa
ECGTGA334V           {0} ; 1552
 baaba ab-aa baaba -abbb abbab babab baaa- aabbb abbab abbbb
 ababa abbaa bbaba aaaba aa--a ---bb aabaa aaaaa abbba aabab
 abbab abaaa bbbba baaab baaaa abaaa babaa abbbb aabbb baaaa
 aaaaa baabb aabab abaaa a-bbb aaaab aabaa abb
SSR_709              {0} ; 1588
 bbbba abbaa bbb-- babab a-aab babbb bbbab -bbbb abbba babbb
 abbab bab-- ----- ----- ----- ----- ----- ----- ----b aabab
 abaa- ----- ----- baaab baa-a abaaa bbbba abbbb aabaa baaaa
 bbaaa baabb abba- abaaa abbbb aaa-b a-baa abb
SSR_759              {0} ; 1593
 babaa aabbb babba aaabb aba-b -abbb baaaa bbaaa babba baaab
 aabba bbabb aabbb aaaab bbaaa ababb baaab ----a bbbba aabbb
 ----- ----b babaa aaaaa aabba abaaa aabba abbbb bbbbb bbaba
 bbbab bbb-a baab- bbbbb abbba baaab baabb bbb

LG8:
EGAMGA57G            {0} ; 1
 baaba baaab aaaba bbbbb babba aabbb babaa babbb aaaab aabbb
 baaab aaaba bbaab aaaba abbab abaaa baaab bbaba baba- -----
 ----- ----- ----- ----- ----- ----- ----- ----- ----- -----
 ----- ----- ----- ----- ----- ----- ----- ---
EGAMGA190V           {0} ; 20
 babab aabab abbbb aabbb abbaa babba aabaa abbaa abaaa baaab
 ababb baaab bbbbb babaa baabb aabaa bbbba abbaa bbbb- -----
 ----- ----- ----- ----- ----- ----- ----- ----- ----- -----
 ----- ----- ----- ----- ----- ----- ----- ---
ECAMCG180G           {0} ; 67
 baaba ababa abbbb aabba abbba bbaab aabba bbbba bbaaa baaab
 ababb aabbb baabb bbaba babab aaaba baaab abbbb bbbb- -----
 ----- ----- ----- ----- ----- ----- ----- ----- ----- -----
 ----- ----- ----- ----- ----- ----- ----- ---
ECAMCG361G           {0} ; 81
 baaab aaaba abbbb aabbb abbba babba aabaa abbaa bbaaa baaab
 ababb aaaab bbbbb babaa babbb aaaaa babba abbaa bbbb- -----
 ----- ----- ----- ----- ----- ----- ----- ----- ----- -----
 ----- ----- ----- ----- ----- ----- ----- ---
ECAMCC207G           {0} ; 151
 babaa baaab aabaa bbbbb abbba aabbb babaa babba abbab babbb
 baaaa aabba aaaab aabba abbaa aaaab aaaba bbaba -aaaa abbaa
 baaab ababb aabbb bbaaa ababb abbba bbbab baaba bbbba abbab
 aabba ababa bbaaa bbbba bbbaa abbbb bbbab aaa
ECAMCC467G           {0} ; 174
```

babaa baaab aabaa bbbbb abbaa aabbb babaa babba abbab babbb
baaaa aabba aaaab abbba abbaa aaaab aaaba bbaba -aaaa abaaa
baaab ababb aabba bbaaa ababb abbba bbbab baaba bbbba abbab
aabba ababa bbaaa bbbba bbbaa abbbb bbbab aaa
ECGMCC152V {0} ; 183
babaa baaab aabaa bbbbb bbbba aabbb babaa babbb abaab babbb
baaab aaaba bbaab aaaba abbab abaab aaaba bbaba babaa abbaa
aaaaa aba-b aabbb bbbaa ababb abbbb bbabb babba bbaba bbaab
abbba aaaba bbaaa bbbab bbbba abaab bbaaa abb
EACMCC167G {0} ; 204
baaba baaab aaaaa bbbba bbbba aabbb abbaa babbb aaaab aabbb
babab ababa bbaaa baaab abbbb ababb bbaaa bbaba babaa abbaa
aaaaa ababb aabbb bbbaa bbabb bbbbb bbabb aabba bbaba bbaab
abbba aaaab bbaaa bbbaa bbbbb abaab bbaaa abb
EACMCC276G {0} ; 219
baaba aaaba abbbb aabba abbba bbaab aabaa bbbaa bbaaa baaab
ababb aabab abbab babab babab aaaaa baabb abbba bbbba abbaa
bbbaa bbbab bbbaa aabba babbb abaaa abbaa aaabb baaba abbaa
abbba abaaa aabbb bbaba abbbb aaaaa aabab abb
EACMCC490V {0} ; 234
baaba baaab aabaa bbbbb abbba aabbb abbaa babba abbab babbb
baaaa aabba aaaaa bbaab abbaa aaaab aaaba bbaba baaaa abbaa
baaab ababb aabbb bbaaa ababb abbba bbbab baaba bbbaa abaab
aabba ababb bbaaa bbbbb bbbaa abbbb bbbab aaa
PpLea-1 {0} ; 241
baaba bbbbb abaaa babba bbbba abaab aabba bbbaa baaaa aaaaa
aaabb abbbb ababb bbaba babab aabba bbaab abbbb bbbba abbab
bbaaa aaaba aabaa aaaaa babbb babbb abbbb aaabb baaba abbaa
abbba aaaab aabbb bbaab bbabb aaaba babaa abb
ECAMGG160G {0} ; 255
bbbab aabab abbbb babbb abbab babba aaaaa aabaa aaaba baaaa
bbabb baaab baabb bbaab babbb abbab abbba babaa aaaaa baaba
babab babba bbbab babaa bbaaa baaaa babab aabaa bbbab abbab
aaaab aabba abbbb aabba aabab baaaa babba abb
ECAMGG287V {0} ; 264
baaba baaab aabba bbbbb abbaa aabbb abbaa babba abbab babbb
baaaa aabba aaaaa bbbab abbaa aaaab aaaba bbaba baaaa abaaa
baaab ababb aabba bbaaa ababb abbba bbbab baaba bbbaa abbab
aabba ababb bbaaa bbbbb bbbaa abbbb bbbab aaa
ECAMGG311V {0} ; 267
baaba baaab aabaa bbbbb abbba aabbb abbaa babba abbab babbb
baaaa aabba aaaaa bbaab abbaa aaaab aaaba bbaba baaaa abbaa
baaab ababb aabbb bbaaa ababb abbba bbbab baaba bbbaa abaab
aabba ababb bbaaa bbbbb bbbaa abbbb bbbab aaa
ECAMGG319G {0} ; 270
baaba baaab aabaa bbbbb aabba aabbb abbaa babba abbab babbb
baaaa aabba aaaaa bbaab abbaa aaaab aaaba bbaba baaaa abbaa
baaab ababb aabbb bbaaa ababb abbba bbbab baaba bbbaa abaab
aabba ababb bbaaa bbbba bbbaa abbbb bbbab aaa
EACMGA287V {0} ; 362
baaaa aaaba abbbb aabbb abbba babba aabaa abbaa bbaaa baaab
ababb aaaab bbabb bbbaa babab aaaaa babba abbba bbbba abbaa
bbba- babab bbaaa aabba babab ababa aabaa aaaab baaaa abbbb
ababa abbaa aabbb bbabb aabbb baaaa aabab aba
EACMGA288G {0} ; 363
baaaa aaaba abbbb aabbb abbba babba aabaa abbaa bbaaa baaab
ababb aaaab bbabb bbbaa babab aaaaa babba abbba bbbba abbaa

```
 bbba- babab bbaaa aabba babab ababa aabaa aaaab baaaa abbbb
 ababa abbaa aabbb bbaba aabbb baaaa aabab aba
EAGMTA208G          {0} ; 403
 baaaa aaaba abbbb aabbb abbba baaaa aabaa abbaa bbaaa baaab
 ababb aabab bbabb b-baa aabab aaaaa babba abbbb bbbba abbaa
 bbba- babab bbaaa aabba babab ababa abbaa aaabb baaba abbba
 ababa abaaa aabbb bbaaa aabbb baaaa aabab abb
EAGMTA208V          {0} ; 404
 baaaa aaaba abbbb aabbb abbba baaaa aabaa abbaa bbaaa baaab
 ababb aabab bbabb b-baa aabab aaaaa babba abbbb bbbba abbaa
 bbba- babab bbaaa aabba babab ababa abbaa aaabb baaba abbba
 ababa abaaa aabbb bbaba aabbb baaaa aabab abb
EACMTC350G          {0} ; 500
 baaba aabba abbbb aabba abbba bbaab aabaa bbbaa bbaaa baaab
 ababb aabab baabb bbaba baba- aaaaa baabb abbba bbbba abbaa
 bbbaa bbbab bbbaa aabba babbb ababa abbaa aaabb baaba abbaa
 abbba abaaa aabbb bbaaa abbbb aaaaa aabab abb
ECGMTT274V          {0} ; 615
 baaba baabb bba-a babba bbbba ababb abbba bbbba baaaa aaaaa
 babab ababb ababb aaaba bab-- abbba bbaab bbbbb babaa abaaa
 abaaa abaab abbba baaba bbbbb babbb ababb aaaba bbaba abaaa
 abbaa baaab ababa babaa bbbbb abaab abbaa abb
EACMGC319V          {0} ; 902
 bbbab aabab abbbb aabbb abbaa babba aaaba aabaa aaaba baaaa
 bbabb baaab abbab babba babbb aabaa abbba aabab aabba baaba
 bbbaa babba bbbab babaa bbaaa abaaa babab aabaa baabb aaaba
 bbaaa aaabb aabbb bbabb bbbba aabba abbaa bbb
EACMGC322V          {0} ; 903
 bbbab aabab abbbb aabbb abbaa babba aaaaa aabaa aaaba baaaa
 bbabb baaab abbab babba babbb aabaa abbba aabaa aabba baaba
 bbbaa babba bbbab babaa bbaaa abaaa babab aabaa baabb aaaba
 bbaaa aaabb aabab bbabb bbaba aabba abbaa bbb
EACMGC326G          {0} ; 904
 bbbab aabab abbbb aabbb abbaa babba aaaaa aabaa aaaba baaaa
 bbabb baaab abbab babba babbb aabaa abbba aabaa aabba baaba
 bbbaa babba bbbab babaa bbaaa abaaa babab aabaa baabb aaaba
 bbaaa aaabb aabab bbaba bbaba aabba abbaa bbb
EACMGC421G          {0} ; 917
 baaba baaab aaaba bbbbb abbaa aabbb babaa babba abbab babbb
 baaaa aabba aaaab abbba abbaa aaaab aaaba bbaba baaaa abaaa
 baaab ababb aabba bbaaa ababb abbba bbbab baaba abbbb abbba
 aabbb bbbba baabb bbaaa bbaba abbaa babbb baa
EAGMCA596G          {0} ; 977
 baaba bbabb abaaa babba bbbba ababb aabba bbbba baaaa aaaaa
 baaab ababb ababb baaba babbb aabba bbaab bbbbb bbbaa abbab
 bbaaa aaaab aabaa baaba bbbbb babbb abbbb aaaba abaaa abbbb
 baaaa bbbba aabbb aabaa baaaa bbbab aaaba bbb
EACMGT127G          {0} ; 1013
 baaba baabb abaaa aabba bbbba ababb babba bbbba baaaa aaaaa
 baabb ababb ababb aaaba babbb ababa bbaab bbbbb babaa abbaa
 abaaa abaab aabba baaba bbbbb babbb abbbb ababa bbaba abaaa
 abbaa baaab ababa baaaa bbbbb abaab bbbaa abb
EACMGT136G          {0} ; 1014
 baaba bbabb abaaa babba bbbba ababb aabba bbbba baaaa aaaaa
 aaabb abbbb ababb baaba babbb aabba bbaab bbbba bbbaa abbab
 bbaaa aaaaa aabaa baaba bbbbb babbb abbbb aaaba baaba abaaa
 abbba baaab abbbb bbaaa bbabb aaaab bbbaa abb
```

```
EACMGT203G          {0} ; 1022
 bbbab aabab abbbb aabbb abbaa babba aaaaa aabaa aaaba baaaa
 bbabb baaab abbab babba babbb aabaa abbba aabaa aabba baaba
 bbbaa babba bbbab babaa bbaaa bbaaa babab aabaa bbbab abbab
 aaabb aabba abbbb aabba aabab baaaa aabba aba
EACMGT596G          {0} ; 1041
 babab aabab abbbb aabbb abbaa babba aabaa abbaa aaaba baaaa
 ababb baaab abbab babba babbb aabaa abbba bbbaa aabba aabba
 bbbaa babba bbaab babaa bbaab abaaa babab aabaa bbaab abbab
 aaabb aabba abbbb abbba aabab baaab aabba abb
EAGMTT407V          {0} ; 1077
 baaba aaabb aaaaa bbbba bbbba aabab babaa babbb baaab aaabb
 babab ababa baaab aaaba bbbbb ababa bbaaa bbaba babaa abbaa
 aaaaa ababb aabbb bbbaa bbabb bbbbb bbabb abbba bbaba bbaaa
 abbba baaab baaaa bbaab bbbbb abaab bbaaa abb
ECGMGG281G          {0} ; 1095
 bbbab aabab abbbb aabbb abbaa babba aaaaa aabaa aaaba baaaa
 bbabb baaab abbab babba babbb aabaa abbba aabaa aabba baa-a
 bbbab babba bbbab babaa bbaaa bbaaa babab aabaa bbbab abbab
 aaabb aabba abbbb aabba aabab baaaa aabba aba
ECGMGG420G          {0} ; 1105
 bbbab aabab abbbb babbb bbbab babba aaaaa babaa aaaba babaa
 bbbbb baaab abbab babba babbb abbab abbba baaaa aaaaa baa-a
 aabab baabb abbbb baaaa bbaaa baaaa bbbab aabaa bbbab abbab
 aaaab aabba ababb aabaa aabab baaaa babba abb
ECGMGG421V          {0} ; 1106
 bbbab aabab abbbb babbb bbbab babba aaaaa babaa aaaba babaa
 bbbbb baaab abbab babba babbb abbab abbba baaaa aaaaa baa-a
 aabab baabb abbbb baaaa bbaaa baaaa bbbab aabaa bbbab abbab
 aaaab aabba ababb aabab aabab baaaa babba abb
EACTCC43G           {0} ; 1116
 baaba ababa abbbb aabba abbba bbaab aabba bbbaa bbaaa baaab
 abbab aabbb baabb bbaba babab aaaba baaab abbba bbbba abbaa
 bbaaa bbbaa bbbaa aabba babbb aabba abbaa aaabb babba abbaa
 abbba abaaa aabbb bbaba abbbb aaaba aabab abb
EACTCC47V           {0} ; 1117
 baaba ababa ababb aabba abbba bbaab aabba bbbaa bbaaa aaaab
 abbab aabbb baabb bbaba babab aaaba baaab abbba bbbba abbaa
 bbbaa bbbaa bbbaa aabba babbb aabba abbaa aaabb babba abbaa
 abbba abaaa aabbb bbaba aabbb aaaba aabab aba
EAGTTA173V          {0} ; 1162
 bbbab aabab abbbb babbb bbbab babba aaaaa babaa aaaba babba
 bbbbb baaab abbab babba babbb abbab abbbb baaaa aaaaa baaba
 aabab baabb abbbb baaaa bbaaa baaaa bbbab aabaa bbbbb abbab
 aabab aabba ababb aabaa abbab baaba babba abb
ECGTGT107V          {0} ; 1182
 bbbab aabab abbbb babbb bbbab babba aaaba babaa aaaba babaa
 bbbbb baaab abbab babba babbb abbab abbba baaab aaaaa baaba
 aabab baabb abbbb baaaa bbaaa baaaa bbbab aabaa bbbab abbab
 aaaab aabba ababb aabaa aabab baaba babba abb
ECGTGT109V          {0} ; 1184
 bbbab aabab abbbb babbb bbbab babba aaaaa babaa aaaba babaa
 bbbbb baaab abbab babba babbb abbab abbba baaab aaaaa baaba
 aabab baabb abbbb baaaa bbaaa baaaa bbbab aabaa bbbab abbab
 aaaab aabba ababb aabaa aabab baaba babba abb
ECGTGT113G          {0} ; 1185
 bbbab aabab abbbb babbb bbbab babba aaaaa babaa aaaba babaa
```

```
 bbaab baaab abbab babba babbb abbab abbba aaaaa aaaaa baaba
 aaaab baabb abbbb baaaa bbaaa baaaa bbbab aabaa abbab abbab
 aaaab aabba abaab aabaa aabab babba babba abb
ECGTGT169G          {0} ; 1191
 baaba baaab aaaaa bbbbb bbbba aabbb babaa babbb aaaab aabbb
 baaab aaaba bbaab aaaba abbab abaab bbaaa bbaba baaaa abbaa
 aaaaa ababb aabbb babaa ababb abbbb bbabb babba bbaaa bbaab
 abbba aaabb bbaaa bbbaa bbbba abaab bbaaa abb
ECGTGT170G          {0} ; 1192
 baaba baaab aaaaa bbbbb bbbba aabbb babaa babbb aaaab aabbb
 baaab aaaba bbaab aaaba abbab abaab bbaaa bbaba baaaa abbaa
 aaaaa ababb aabbb bbaaa ababb abbbb bbabb babba bbaaa bbaab
 abbba aaabb bbaaa bbbaa bbbba abaab bbaaa abb
ECGTGT368V          {0} ; 1215
 baaba baaab aaaaa bbbba bbbba aabbb babaa babbb aaaab aabbb
 baabb ababa bbaab aaaba abbbb ababb bbaaa bbaba baaaa abbaa
 aabaa ababb aabbb bbbaa bbabb bbbbb bbabb abbba bbaba bbaab
 abbba aaaab bbaaa bbbaa bbbbb abaab bbaaa abb
EACTAG321V          {0} ; 1259
 baaaa aabbb abbbb aabbb abbba babba aabaa abbaa bbaaa aaaab
 abbab aaaab bbbbb babaa babbb aabaa babba abbaa bbbaa abbaa
 bbbaa babba bbaab aabaa baba- ababa aabaa aaaab bbaaa abbab
 aaaba abbba abbbb abbba aabab baabb aabbb aba
EAGTCA158V          {0} ; 1290
 baaba baaab aaaaa bbbbb bbbba aabbb babaa babbb aaaab aabbb
 baaab aaaba bbaab aaaba abbab abaab bbaaa bbaba babaa abbaa
 abaaa ababb aabbb bbbaa abab- abbbb bbabb babba bbaaa bbaab
 abbba aaabb bbaaa bbbaa bbbba abaab bbaaa abb
EAGTCA176G          {0} ; 1295
 baaba baaab aaaaa bbbbb bbbba aabbb babaa babbb aaaab aabbb
 baaab aaaba bbaab aaaba abbab abaab bbaaa bbaba babaa abbaa
 aaaaa ababb aabbb bbbaa abab- abbbb bbabb babba bbaaa bbaab
 abbba aaabb bbaaa bbbaa bbbba abaab bbaaa abb
EAGTCA411G          {0} ; 1315
 bbbab aabab abbbb babbb bbbab babbb baaaa bbbaa aabba babaa
 bbbbb baaab abbab babba babbb abbab abbba baaaa aaaaa baaba
 aabab baabb abbbb baaaa bbab- baaba bbbab aabaa bbbab abbab
 aaaab aabba ababb aabaa aabab baaba babba abb
EACTGA508G          {0} ; 1382
 baaba bbabb abaaa babba bbbba ababb aabba bbbba baaaa aaaaa
 aaba- abbbb ababb ba-ba babbb a-bba bbaab bbbbb bbbaa abbab
 bbaaa aaaaa aabaa baaba bbbbb babbb abbbb aaaba baaba a-aaa
 a-bb- baaab a-bbb bbaaa ----b ----- bbbab abb
EACTGA510V          {0} ; 1383
 baaba bbabb abaaa babba bbbba ababb aabba bbbba baaaa aaaaa
 aaba- abbbb ababb ba-ba babbb a-bba bbaab bbbbb bbbaa abbab
 bbbaa aaaaa aabaa baaba bbbbb babbb abbbb aaaba baaba a-aaa
 a-bb- baaab a-bbb bbaaa ----b ----- bbbaa abb
EAGTGC349G          {0} ; 1409
 baaba bbabb abaaa babba bbbba ababb aabba bbbba baaaa aaaaa
 aaba- abbbb ababb ba-ba babbb a-bba bbaab bbbbb bbbaa abbab
 bbaaa aaaaa aabaa baaba bbbbb babbb abbbb aaaba baaba abaaa
 abbba baaab abbbb bbaaa bbabb aaabb bbbaa bbb
EAGTAA166V          {0} ; 1505
 baaba ab-ba abbbb -abba abbb- bbaab aabb- bbb-a bb-aa a-aab
 -bbab aabbb b-abb bbaba ba--b ----b baa-b -b--- ----a abbba
 bbbaa bbbaa abbaa aabba babbb aabba abbaa aaabb babba abbaa
```

```
 abbba abaaa aabbb b--b- a-bbb aaaba aabab abb
SSR_765              {0} ; 1594
 babab aabab abbb- ----b abb-- ---ba ----a aabaa abaaa b---a
 ababb baaab bbbbb babab babbb aabaa babba abbaa bbbaa abbaa
 bbbaa babba abaab babaa bbaab abaaa ----- ----- ----- ----b
 aaaba aabba a---- ----- ----- ----- ----- ---
SSR_925              {0} ; 1605
 baaba baaa- aaa-a -bbba bbbba aa--- -a-aa ba--- aaaa- a----
 -a-a- a-a-a b-aa- aaa-a a-bbb a-b-b --aaa bba-a babba a-baa
 aaaaa abb-b aaa-- ---aa --abb -bbb- b-b-- aabba a---a bbaab
 abbba aaaab bbaa- bbbaa bbbb- abaab bbaaa ab-

LG9:
EGAMGA151V           {0} ; 15
 aabab aabaa aabbb babab abaab bbbbb baaab abbab bbbaa babba
 baaab babab bbaba babba ababb babba bbaab aaaab aaaa- -----
 ----- ----- ----- ----- ----- ----- ----- ----- ----- -----
 ----- ----- ----- ----- ----- ----- ----- ---
EGAMGA311V           {0} ; 32
 ababb babbb aabbb babab baaab babbb aaabb abbaa bbbaa babbb
 baaab babbb baaba babab ababa bbbaa abbab baaaa aaaa- -----
 ----- ----- ----- ----- ----- ----- ----- ----- ----- -----
 ----- ----- ----- ----- ----- ----- ----- ---
ECGMCG352G           {0} ; 96
 aabab aabaa aabbb babab abaab bbbbb baaab abbab bbbaa babba
 baaab babab bbaba babba ababb babba baaab aaaab aaaa- -----
 ----- ----- ----- ----- ----- ----- ----- ----- ----- -----
 ----- ----- ----- ----- ----- ----- ----- ---
ECAMCC235G           {0} ; 155
 aaabb aaaaa aabbb babab abaab bbbbb baaab abbbb babaa babba
 baaab babab bbaba babba abaab babaa bbaab abaab -aaaa bbaab
 ababb ababb aabba babbb aaaaa aabaa babbb babaa baaaa abaaa
 aabaa abaab abbaa aabba aabba bbbba ababb abb
EACMCC217V           {0} ; 214
 ababb bbabb aabbb babbb bbaab babbb ababb abbbb bbbab babbb
 baaab babab abbaa ababa abaaa bbbaa aabaa bbaab aaaaa aaaab
 abbab ababb aabba bbbbb abaaa aabab babbb babab baaba aabaa
 abbab abaab aaaba abbbb bbbba babbb abbbb bbb
EACMCC218G           {0} ; 215
 ababb baabb aabbb babbb bbaab babbb ababb abbbb bbbaa babbb
 baaab babab abbaa ababa abaaa bbbaa aabaa bbaab aaaaa aaaab
 abbab ababb aabba bbbbb abaaa aabab babbb babab baaba aabaa
 abbab abaaa aaaba abbba abbba babbb abaab bbb
ECAMGG295G           {0} ; 265
 ababa babbb aabab babbb bbaab babbb aaabb abbaa bbbaa babbb
 baaab babbb abbab ababa ababa bbbaa aabab bbbab aaaaa aabaa
 bbaaa ababb aabbb babbb abaaa babab babbb babab aabba aabaa
 abbab bbbab aaaba aabba aabbb babbb abaab bbb
ECGMTA164G           {0} ; 308
 aabab aabaa aabbb babab abaab bbbbb abaab abbab bbbaa babba
 baaab babab bbbab abaab ababb babaa bbaab aaaab aaaaa bbaab
 ababb ababb aabba babbb aaaaa aabaa aabba babaa baaba abbaa
 aabaa abaaa aabaa aaaaa aabaa bbbba ababb abb
ECGMTA470G           {0} ; 328
 ababb babbb aabab babab bbaab babbb aaabb abbaa bbbaa babbb
 baaab babbb abbab ababa ababa bbbaa aabab bbaaa aaaaa aabaa
 bbaaa ababb aabbb babbb abaaa babab babbb babab aabba aabaa
```

abbab bbbab aaaba aabba abbbb babbb abaab bbb
EACMGA294G {0} ; 365
abaaa aaaab aabbb bbbab baaba bbaab ababb abaab aabba bbbab
bbaaa baaab aaaba bbaab ababa abbba babbb abbaa baaaa abbaa
bbaa- ababb aabbb aabaa babaa aaaaa babab bbbbb aabba aabba
aaaab babaa ababa aaaaa bbbab abaaa aabbb bab
EACMGA538V {0} ; 388
abaaa baabb aabab babab bbaab babbb ababb abbab bbbaa babab
baaab babab baaba baaab aaaaa aabba aaabb bbbaa baaaa aaaaa
baaa- ababb aabbb babab baaaa aabab babbb babab aabba aabba
aabab babab aaaba aaaab bbbbb abbba aaaab bbb
EAGMTA260V {0} ; 409
aabab aabaa aabbb babab abaab bbbbb baaab abbab bbbaa babba
baaab babab bbaba b-bba ababb babba bbaab aaaab aaaaa bbaab
abab- ababb aabba babbb aaaaa aabaa aabba aaaaa aaaba abbaa
aabaa abaab abbaa aaabb aabaa bbbba ababb abb
ECGMGA178G {0} ; 432
abbab bbabb aabbb babbb abaab bbbbb abaab abbbb bbbaa babba
baabb babab bbaba a-bab abaab bbbaa abbaa abaab aaaaa baaab
abba- ababb aabba bbbbb abaaa aabab bbbbb babab baaba abaaa
aaaab abaaa abbaa abbab abbba bbbbb ababb bba
ECGMGA179V {0} ; 433
abbab bbabb aabbb babbb abaab bbbbb abaab abbbb bbbaa babba
baabb babab bbaba a-bab abaab bbbaa abbaa abaab aaaaa baaab
abba- ababb aabba bbbbb abaaa aabab bbbbb babab baaba abaaa
aaaab abaaa abbaa abbbb abbba bbbbb ababb bba
ECGMGA257G {0} ; 437
aabab aabaa aabbb babab abaab abbbb abaab abaab bbbaa babbb
bbaab aabab bbaba b-bba ababb baaba bbaab aaaab aaaaa bbaab
abab- ababb aabba babbb aaaaa aabaa abbba babab baaba abbaa
aabaa abaaa abbaa aaaab aabaa bbbba ababb abb
ECGMGA379V {0} ; 441
ababa baabb aabab babab bbaab babbb baabb abbab bbbaa babab
baaab babab baaba b-aab ababa bbbba aabbb bbbaa baaaa aabaa
bbaa- ababb aabbb babab baaaa aabab babbb babab aabba aabba
abbab babab aaaba aaabb bbbbb abbba aaaab bbb
EACMTC249V {0} ; 486
abbab ababb aabbb babbb abaab bbbab bbaab abbbb bbbaa babbb
baabb babab bbaba aabab abaa- bbbaa abbaa abaab aaaaa baaab
abbab ababb aabba bbbbb abaaa aabab bbbbb babab baaba abaaa
abaab abaaa abbaa abbbb abbba bbbbb ababb bba
EACMTC284V {0} ; 489
aabab aabba aabab babab baaab abbbb bbbab aaaab bbbaa aaabb
abaab aaaab bbbba babba abab- baaaa bbabb aaaab aaaba bbaab
aaabb ababb aabba aabbb aaaab aabaa abbba baaab aaaba abbaa
aabaa abaaa abbaa aaaba aabaa bbbba abaab aba
EACMTC437V {0} ; 520
abbab bbabb aabab babbb abbab bbbbb baaab abbbb bbbaa babba
baabb babab bbaba aabab abaa- bbbaa abbaa abaab aaaaa aaaab
abbab ababb aabba bbbbb abaaa aabab bbbbb babab baaba abaaa
abaab abaaa abbaa abbbb abbba bbbbb ababb bba
EACMTC526G {0} ; 528
aabab aabaa aabbb babab abaab bbbbb baaab abbab bbbaa babba
baaab babab bbaba babba abab- babba bbaab aaaab aaaaa bbaab
ababb ababb aabba babbb aaaaa aabaa aabba babaa baaba abbaa
aabaa abaaa abbaa aaaab aabaa bbbba ababb abb
ECAMCT105V {0} ; 545

aabab babaa aabbb babab abaab bbbbb baaab abbab bbbaa babba
baaab babab bbaba babba abab- babba bbaab aaaab aaaaa bbaab
ababb ababb aabba babbb aaaab aabaa aabba babaa baaba abbaa
aabaa abaaa abbaa aaabb aabaa bbbba ababb abb
ECAMCT361G {0} ; 581
aabab aabaa aabba babab bbaab bbbbb baaab abbab bbbaa babba
baaaa babab bbaba babba abab- babba bbbab aabbb ababa bbaab
ababb ababb aabba babbb aaaaa aabaa babba babba baaba abbaa
aabaa abaab abbaa aaaab aaaaa bbbba ababb abb
ECAMCT442G {0} ; 589
ababb babab aabbb bbbab baaba bbaab ababb aaaab aabba bbbab
bbaaa bbaab aaaba bbaab abab- aabba babbb babaa baaaa abbaa
baaaa ababb aabbb aaaaa babaa aaaaa babab bbbba aabba aabba
aabab baaaa aaaba aaaaa bbbab abaaa aabbb bab
EAGMGG192V {0} ; 678
abbab ababb aabbb babbb abaab bbbbb baabb abbbb bbbaa babba
baabb babab bbaba aabab abaab bbbaa abaaa abaab aaaaa baaab
abbab abbbb aabba bbbbb abaaa aabab babbb babaa baaba abaaa
aaaab abaaa abbaa abbbb abbba bbbbb ababb bba
EAGMGG515V {0} ; 701
ababa baaab aabab babab bbaaa bbabb ababb abaab aabba bbbab
bbaaa baaab aaaba bbaab aaaba bbbba aabbb bbbaa baaaa abbaa
bbaaa ababb aabbb aabaa babaa aabaa babbb babab aabba aabba
aabab babaa aaaba aaaab bbbbb abaaa aabab bab
EAGMGG515G {0} ; 702
ababa baaab aabab babab bbaaa bbabb ababb abaab aabba bbbab
bbaaa baaab aaaba bbaab ababa bbbba aabbb bbbaa baaaa abbaa
bbaaa ababb aabbb aabaa babaa aabaa babbb babab aabba aabba
aabab babaa aaaba aaaaa bbbbb abaaa aabab bab
ECGMGC282V {0} ; 715
ababa babbb aabab babbb bbaab babbb aaabb abbaa babaa babbb
baaab babbb baaba babab ababa babaa aabab bbbab aaaaa aabaa
bbaaa ababb aabbb babbb aaaaa babab babbb babab aabba aabaa
abbab bbbab aaaba aabbb abbbb babab abaab bbb
ECGMGC283V {0} ; 716
ababa babbb aabbb babbb bbaab babbb aaabb abbaa babaa babbb
baaab babbb baaba babab ababa bbbaa aabab bbbab aaaaa aabaa
bbaaa ababb aabbb babbb aaaaa babab babbb babab aabba aabaa
abbab bbbab aaaba aabbb abbbb babab abaab bbb
EACMGG293V {0} ; 770
ababa aaaab aabbb bbbab baaba bbaab ababb abaab aabba b-bab
bbaaa baaab aaaba bbaab ababa abbba babbb bbbab baaaa abbaa
bbaaa ababb aabbb aabaa babaa aaaaa b-bab bbbbb aabba aabba
aaaab babaa aaaba aaaba bbbab abaaa aabbb bab
EAGMCT441V {0} ; 842
aabab aabaa aabab babab abaab ababb abaab abaab abbaa b-abb
bbaab aabab bbaba babba aaabb baaaa bbabb aaaab aaaaa bbaab
aaabb ababb aabba aabbb aaaab aaaaa a-bba babab aaaba abbaa
aabaa abaaa abbaa aaabb aabaa bbbba abaab abb
ECGMGT64G {0} ; 845
ababa babbb aabab babbb bbaab babbb aaabb abbaa bbbaa b-bbb
baaab babbb baaba babab ababa bbbaa aabab bbbab aaaaa aabaa
bbaaa ababb aabbb babbb abaaa babab b-bbb babab aabba aabaa
abbab bbbab aaaba aabab abbbb babbb abaab bbb
ECGMGT164G {0} ; 852
ababa baabb aabab babab bbaab babbb baabb abbab bbbaa a-bab
baaab babab baaba baaab ababa bbbba aabbb bbbaa baaaa abbaa

```
 bbaaa ababb aabbb babab baaaa aabab b-bbb babab aabba aabba
 aabab babab aaaba aaaab bbbbb abbba aaaab bbb
EAGMCA235V          {0} ; 955
 aabab aabaa aabbb babab abaab bbbbb abaab abbab bbbaa babba
 baaab babab bbaba babba ababb babba bbaab aaaab aaaaa bbaab
 ababb ababb aabba babbb aaaaa aabaa aabba babaa aaaaa baaba
 abbbb aabab babbb aabab bbaaa abaaa baaaa bba
EAGMCA240G          {0} ; 956
 aabab aabaa aabbb babab abaab bbbbb abaab abbab bbbaa babba
 baaab babab bbaba babba ababb babba bbaab aaaab aaaaa bbaab
 ababb ababb aabba babbb aaaaa aabaa aabba babaa aaaaa baaba
 abbbb aabab babbb aabaa bbaaa abaaa baaaa bba
EAGMTT157V          {0} ; 1057
 abaab bbabb aabbb babbb abaab bbbbb baaab abbbb bbbaa babba
 baabb babab ababa aabab abaaa bbbaa abbaa bbaab aaaaa aaaab
 abbab ababb aabba bbbbb abaaa aabab babbb babab baaba abbaa
 abbab abaaa abaaa abbbb abbba bbbbb ababb bba
ECGMGG476V          {0} ; 1107
 ababa babbb aabab babbb bbaab babbb aaabb abbaa bbbaa babbb
 baaab babbb baaba babab ababa bbbaa aabab bbbab aaaaa aab-a
 bbaaa ababb aabbb babbb abaaa babab babbb babab aabba aabaa
 abbab bbbab aaaba aabbb abbbb babba abaab bbb
EACTAG412V          {0} ; 1276
 aabab aabaa aabbb babab abaab bbbbb baaab abbab bbbaa babba
 baaab babab bbaba babba ababb babab bbaab aaaab aaaaa bbaab
 ababb ababb aabba babbb aaaa- abbaa aabba babaa baaba abbaa
 aabaa abaaa abbaa aaaab aabaa bbbaa ababb abb
ECGTAC94V           {0} ; 1324
 abaab baabb aabbb babab abaab baabb ababb abbab bbbaa babab
 baaab babab ababa baaab ababa bbbba aabbb bbbaa baaaa aabaa
 bbaaa babba abbbb ababb aaaa- aabab babbb babab aabba aabba
 abbab babab aaaba aaaab bbbbb abbaa aaaab bbb
ECGTAC212V          {0} ; 1330
 ababb babbb aabbb babbb bbaab babbb aaabb abbaa bbbaa babbb
 baaab babbb baaba babab ababa bbbaa aabab bbbab aaaaa aabaa
 bbaaa ababb aabbb babbb abaa- babab babbb babab aabba aabaa
 abbab bbbab aaaba aabbb abbbb babab abaab bbb
EACTGA105G          {0} ; 1349
 aabab aabaa aabab babab abaab ababb baaab aaaab bbbaa baabb
 bbaa- aabab bbaba ba-ba ababb b-aaa bbabb aaaab aaaaa bbaab
 aaabb ababb aabba aabbb aaaab aabaa abbba babab aaaba abbba
 aabaa abaaa abbaa aaaab aabba bbbaa abaab abb
EAGTGC327V          {0} ; 1406
 abbab bbabb aabbb babbb abbab bbbbb baaab abbbb bbbaa babba
 baba- babab baaba aa-ab abaaa b-baa abbaa bbaab aaaaa aaaab
 abbab ababb aabba bbbbb abaaa aabab bbbbb babab baaba abaaa
 abaab abaaa abaaa abbbb abbba bbbab ababb baa
ECGTCA199V          {0} ; 1428
 abbab ababb aabbb babbb abaab bbbbb baaab abbbb bbbaa babba
 baba- babab bbaba aa-ab abaab b-baa abaaa abaab aaaaa baaab
 abbab ababb aabba bbbbb abaaa aabab babbb babaa baaba abaaa
 aaaab abaaa abbaa abbbb abbba bbbab ababb bba
ECGTCA315G          {0} ; 1431
 aabab aabaa aabbb babab abaab bbbbb baaab abbab bbbaa babba
 baaa- babab bbaba ba-ba ababb b-bba bbaab aaaab aaaaa bbaab
 ababb ababb aabba babbb aaaba aabaa aabba babbb baaba abbaa
 aabaa abaaa abbaa aaaab aabaa bbbaa aaabb abb
```

```
EACTTC499G           {0} ; 1489
 abaaa aa-ba aabbb -bbbb abaab bbbbb baaa- abbbb babaa bbbba
 baaab baabb bbaba aabba ab--b ---aa abaab abaab aaaaa bbaab
 abaab ababb aabba bbbbb abaaa aabaa babbb babaa baaba abaaa
 aaaaa abaaa abbaa abbbb a-bba bbbab ababb aba
EAGTAA285V           {0} ; 1515
 abbab bb-bb aabbb -bbbb abaa- bbbbb baab- abb-b bb-aa b-bba
 -abab babab b-aba aabab ab--a ----a abb-a -b--- ----a aaabb
 abbab ababb aabba bbbbb abaaa aabab bbbbb babab bbaba abaaa
 abaab abaaa abbaa a--b- a-bba bbbab ababb bba
EAGTAA446V           {0} ; 1524
 ----- ----- ----- ----- ----- ----- ----- ----- ----- -----
 ----- ----- ----- ----- ----- ----- ----- ----- ----a bbabb
 aaabb ababb aabba aabbb aaaab aabaa abbba babab aaaba abbaa
 aabaa abaaa abbaa a--a- a-baa bbbaa abaab abb
EAGTAA451G           {0} ; 1525
 ----- ----- ----- ----- ----- ----- ----- ----- ----- -----
 ----- ----- ----- ----- ----- ----- ----- ----- ----a bbaab
 aaabb ababb aabba aabbb aaaab aabaa abbba babab aaaba abbaa
 aabaa abaaa abbaa a--a- a-baa bbbaa abaab abb
ECGTGA202V           {0} ; 1539
 abaab bb-bb aabbb -aabb bbaab bbbbb baaa- abbbb bbbaa babba
 babab babab bbaba aabab ab--a ---aa abbaa bbaab aaaaa aaaab
 abbab ababb aabba bbbbb abaaa aabab babbb bbbab baaba abbaa
 abbab abaaa abaaa abbbb a-bba bbbab ababb bba
SSR_1079             {0} ; 1612
 ababa babbb aabab babab bbaab babbb aaabb abbaa bbbaa babbb
 baaab babbb baaba babaa ababa bbbaa aabbb -bbaa baaba aabaa
 bbaaa bbbbb aaabb babab baaaa aabab babbb babab aabba aabaa
 abbab bbb-b aaab- aabab bbbbb aabab abaab b--

LG10:
ECAMCG561G           {0} ; 88
 bbaab aaaaa bbabb aaaaa abbbb ababa babab aabba baaba baabb
 abaab aabbb bbbbb bbaaa bbbba abbaa aabba aaabb bbba- -----
 ----- ----- ----- ----- ----- ----- ----- ----- ----- -----
 ----- ----- ----- ----- ----- ----- ----- ---
ECAMCC403G           {0} ; 167
 abaaa aaabb baaab baaaa baaba babba bbbab aabab ababa ababb
 aaabb baaab bbbbb baabb babaa babba babab abbab -baab bbaba
 babba bbbbb abbaa aabbb bbbaa bbbba aaaab bbbbb baaba aaabb
 aaaaa abbaa bbbba abbba aabab aabba bbabb abb
ECAMCC417G           {0} ; 170
 abbaa aaaba baaab baaaa baaba babba bbbab aabab ababa ababb
 aaabb baaab bbbbb baabb babaa babba babab abbaa -baab bbaba
 babba bbbbb abbaa aabbb bbbaa bbbba baaab bbbbb baaba aaabb
 aaaaa abbaa bbbba abbba aabab aabbb bbabb abb
ECAMCC419V           {0} ; 171
 abbaa aaaba baaab baaaa baaba babba bbbab aabab ababa ababb
 aaabb baaab bbbbb baabb babaa babba babab abbab -baab bbaba
 babba bbbbb abbaa aabbb bbbaa bbbba baaab bbbbb baaba aaabb
 aaaaa abbaa bbbba abbbb aabab aabbb bbabb abb
ECAMGG384G           {0} ; 280
 abaaa baabb baaab bbaab baabb babbb bbbab aabab ababa aaabb
 aaabb bbaab bbbbb baabb babaa babba babab abbaa bbaab bbaba
 bbbba baaab abbaa aabbb bbbba bbbba aaaaa bbbba baaaa aaabb
 aaaaa abbab bbbba abaaa aabab aabba ababb aab
```

```
ECAMGG391V           {0} ; 282
 abaaa baabb baaab bbaab baabb babbb bbbbb aabab ababa aaabb
 aaabb bbaab bbbbb baabb babaa babba babab abbab bbaab bbaba
 bbbba babab abbaa aabbb bbbba bbbba aaaaa bbbba baaaa aaabb
 aaaaa abbab babba abaab aabab aabba ababb aab
ECAMGG392V           {0} ; 283
 abaaa baabb baaab bbaab baabb babbb bbbbb aabab ababa aaabb
 aaabb bbaab bbbbb baabb babaa babba babab abbab bbaab bbaba
 bbbba babab abbaa aabbb bbbba bbbba aaaaa bbbba baaaa aaabb
 aaaaa abbab babba abaab aabab aabba ababb bab
ECGMTA346V           {0} ; 320
 bbaab aaaaa bbabb aaaaa abbbb ababa abbaa aabba baaaa baabb
 abaab aabbb bbbab babaa bbbba abaaa aabba aaabb bbbab bbbbb
 baaaa babaa abbaa baaba bbaaa abbaa babaa ababb babab abbab
 abaab abbab bbbaa bbaab babbb bbaab aaaaa aba
EACMGA533G           {0} ; 386
 bbabb aaaaa bbabb aaaab abbbb ababa babaa aabba baaba baabb
 abaab aabbb bbabb bbaaa bbbba bbaaa aabba aaabb bbbab bbbbb
 bbaa- babaa aabaa baaba bbaaa aabaa babaa ababb babab abbab
 abaab abbab bbbaa bbaaa babbb bbaab aaaaa aba
EAGMTA282V           {0} ; 411
 aaaba aaabb baaab baaba bbaab babba aabbb babab ababa bbbab
 aaabb baaba ababb b-abb aabaa babaa babaa abbab abaab bbaba
 baab- bbbbb abbaa aabbb bbbab babba bbabb bbbbb baaaa aabbb
 aaaaa abbaa aabba abbbb aabab aabbb bbabb aab
EAGMTA313G           {0} ; 415
 baaab aabba bbaab aaaaa ababb abbba bbbaa aabba baaaa baabb
 abaab aabbb bbaab b-baa aabba aaaaa aabbb aaabb bbbab bbbba
 baaa- babaa abbaa aabba bbaba aabaa babaa ababb babab abbab
 ababa abbab bbbba baaab baabb bbaaa aaaaa aba
EACMTC77V            {0} ; 450
 aaabb aaaba abaaa bbbba bbabb bbbba aabab babab ababb bbbbb
 aaabb babba ababb baabb bbba- babaa baabb aabaa bbbab bbbba
 abaaa bbabb abbab aabbb babbb aabab bbaba bbbbb bbaaa abbbb
 aaaaa abbaa babaa abbba baaaa aabba baaba aba
EACMTC191G           {0} ; 472
 abaab aabba bbabb aaabb abbbb bbaab bbaaa aabba baabb aaabb
 bbbab babbb abbab bbbab bbab- baaba aaabb aaabb bbbab bbbba
 baaaa babaa abbaa aabba bbabb aabaa aabaa ababb babaa abbab
 ababa abbbb bbbaa bbaab babbb bbaaa aaaaa bbb
EACMTC192V           {0} ; 473
 abaab aabba bbabb aaabb abbbb bbaab bbaaa aabba baabb aabbb
 bbbab babbb abbab bbbab bbab- baaba aaabb aaabb bbbab bbbba
 baaaa babaa abbaa aabba bbabb aabaa aabaa ababb babaa abbab
 ababa abbbb bbbaa bbabb babbb bbaaa aaaaa bbb
EACMTC229G           {0} ; 483
 abaab aabba bbabb aaabb abbbb bbaab bbaaa aabba baaaa aaabb
 babab babbb abbab bbbab bbab- baaba aaabb aaabb bbbab bbbba
 baaaa babaa abbaa aabba bbabb aabaa aabaa ababb babaa abbab
 ababa abbbb abbaa bbaab babbb bbaaa aaaaa bbb
EACMTC426G           {0} ; 518
 abaaa baaba bbabb bbaab baaab babbb bbaab aaabb ababb aaabb
 ababb bbaab bbbbb baabb baaa- baaba bbbab ababa bbaab bbaba
 baaba babaa abbaa aabbb bbbbb bbbba aaaaa bbbba baaaa abbbb
 aaaba abbab babbb abaab babaa babba abaab abb
ECAMCT166G           {0} ; 556
 baaab aabba ababb aaaaa abbbb abbaa bbbaa aabba baaaa baabb
```

abbab aaabb bbaab bbbab bbbb- aaaba aabbb aaaba bbbab abbba
baaaa babaa abbaa aabba bbaba aabaa babaa ababb babab abbab
ababa abbab bbbaa bbaab babbb bbaaa aabaa aba
ECAMCT297G {0} ; 574
aaabb aaaba abaaa bbbba bbabb bbbba aabab babab ababb bbbba
aaabb babba ababb baabb bbba- babaa baabb aabba bbbab bbbba
abaaa bbabb abbab aabbb babbb aaaab bbaba bbbbb bbaaa abbbb
aaaaa abbaa babaa abbaa baabb abbba baaba aba
EACMGG109V {0} ; 741
aaabb baabb abaaa babba bbabb bbbba aabbb babab ababb b-baa
aaabb babba ababb baabb bbbaa babba baaab aabab bbbab bbaba
bbaaa bbbab abbab aabbb babbb aabab b-aba bbbbb bbaaa abbbb
aaaaa abbaa babaa abbbb aaaaa aabba baaba aba
EACMGG152V {0} ; 748
abaab aabba bbabb aaabb aabab bbbab bbaba aaaba baabb a-abb
bbbbb babab abbab bbbab bbaba baaba aaabb aaabb bbbab bbbba
baaaa babaa abbaa babba bbabb abbaa a-baa ababb babaa abbab
ababa abbbb bbbaa bbabb babba bbaaa aaaaa bbb
EAGMCT107G {0} ; 805
aaabb baabb abaaa baaba bbabb bbbaa aabab babab ababb b-baa
aaabb babba ababa baabb bbbaa babba baaab aabaa bbbab bbaba
bbaaa bbbab abbab aabbb babbb aabab b-aba bbbbb bbaaa abbbb
aaaaa abbaa babaa abbab aaaab aabba baaba aba
EAGMCT138G {0} ; 807
abaab aabba bbabb aaaaa abbbb abbaa bbbaa aabba baaaa b-abb
abbab aabbb bbaab bbbab bbbba aaaba aabbb aaabb bbbab bbbba
baaaa babaa abbaa aabba bbaba aaaaa b-baa ababb babab abbab
ababa abbab bbbaa bbaab babbb bbaaa aaaaa aba
EAGMCT139V {0} ; 808
bbaab aabba bbabb aaaaa abbbb abbaa bbbaa aabba baaaa b-abb
abbab aabbb bbaab bbbab bbbba aaaba aabbb aaabb bbbab bbbba
baaaa babaa abbaa aabba bbaba aabaa b-baa ababb babab abbab
ababa abbab bbbaa bbabb babbb bbaaa aaaaa aba
EACMGC257V {0} ; 890
aaabb baabb aaaaa babba bbabb bbbba aabbb babab ababb bbbaa
aaabb babba ababb baabb bbbaa babba baaab aabab babab bbaba
bbaaa bbbab abbab aabbb babbb aabab baaba bbbbb aabbb baaaa
aaabb abaab aabab baaab bbbba aaaaa bbaab aba
EACMGC258G {0} ; 891
aaabb baabb abaaa babba bbabb bbbba aabab babab ababb bbbaa
aaabb babba ababb baabb bbbaa babba baaab aabaa bbbab bbaba
bbaaa bbbab abbab aabbb babbb aabab baaba bbbbb aabbb baaaa
aaabb abaab aabab baaaa bbbba aaaaa bbaab aba
EACMGC309V {0} ; 900
aaabb aaaba abaaa bbbba bbabb bbbba aabbb babab ababb bbbbb
aaabb babba ababb baabb bbbaa babaa baabb aabab bbbab bbbba
abaaa bbabb abbab aabbb babbb aabab bbaba bbbbb aabbb abaaa
aaabb abaab aabab baaab bbbba aaaaa bbaab aba
EACMGC336G {0} ; 906
aaaab aaaaa abaaa bbbba bbabb bbaba aaaab aabab ababb bbbbb
aaabb babba ababb baabb bbbaa babaa baabb aabaa abaab bbbba
abaaa bbabb abbab aabbb babbb aabab bbaba bbbbb aabbb abaaa
aaabb abaab aabab baaaa bbbba aaaaa bbaab aba
EACMGC560G {0} ; 926
ababa baabb baaab bbaab baabb babbb bbbbb aabab ababa aaabb
aaabb bbaab bbbbb baabb babaa babba babab abbab bbaab bbaba
bbbba babab abbaa aabbb bbbbb bbbba aaaaa bbbba babaa baaba

baabb aabab baabb aaaaa aabba aaaaa bbabb bbb
ECGMCA281G {0} ; 991
bbaab aaaaa bbbab aaaaa abbbb ababb babab aabba baaba baabb
abaab aabbb bbabb bbaab bbbba abbaa aabba ababb bbbab bbbbb
baaaa babaa aabaa baaba bbaaa aabaa babaa ababb abbaa bbabb
bbbaa baaaa aabab ababa bbaba baaba bbabb bba
EACMGT45bG {0} ; 1001
abaab abbba bbabb aaabb aabab bbbab bbaaa aabaa baabb aaabb
bbbbb babbb abbab bbbab bbaba babaa aaabb aaabb bbbbb bbbba
baaaa babaa abbaa babba bbaba abbaa aabaa ababb babaa abbab
ababa abbbb bbbaa bbaaa babbb bbaaa aaaaa bbb
EACMGT105V {0} ; 1010
aaaba aaabb baaab baaba bbaab babba aabab babab ababa bbbab
aaabb baaba ababb baabb aabaa babaa babaa abbab abaab bbaba
baaba bbbbb abbaa aabbb bbbab babaa bbabb abbbb baaaa aabbb
aaaaa abbaa abbba abbbb aabab aabbb bbabb aab
EAGMTT148V {0} ; 1053
bbbaa aaaab bbabb aaaaa abbab ababa babbb aaaba baaba baabb
abaaa aaabb abbbb bbaaa bbbbb aabaa aabaa aaaab bbbab babab
babba abbba aabaa baaba aabaa aabba abbaa ababa babab aabab
abaab abbba bbbaa bbaab aabab bbbbb baaab abb
EAGMTT241G {0} ; 1066
aaabb baabb abaaa baaba bbabb bbbba aabab babab ababb bbbab
aaabb babba ababa baabb bbbaa babba baaab aabaa bbbab babba
bbaaa bbbab abbab aabbb babbb aabab bbaba bbbbb bbaaa abbbb
aaaaa abbaa babaa abbba aaaab aabba baaba aba
EACTCC129V {0} ; 1125
aaaba baabb bbbaa baaba bbabb bbbba aabab babab ababa bbbab
aabab baaba ababa baabb bbbaa babaa babaa aabab bbaab bbaba
babab bbbbb abbab aabbb babab aabaa bbaba bbbbb bbaaa aabbb
aaaaa abbaa aabaa abbbb aaaab aabba baabb aab
EACTCC373V {0} ; 1141
aaaba aaaba abaaa bbbba bbabb babaa aabbb babab ababb bbbbb
aabab babba ababb baabb bbbaa babaa baabb aabab bbbab bbbba
abbaa bbabb abbab aabbb babbb aabab bbaba bbbbb bbaaa abbbb
aaaaa abbaa babaa abbba baaaa aabaa baaba aba
EACTAG106V {0} ; 1229
abaaa baabb bbabb bbaab baaab babbb bbaab aaabb ababb aaabb
abbab bbaab bbbbb baabb babaa baaab bbbab ababb bbaab bbaba
bbaba babaa abbaa aabbb bbbb- bbbba aaaaa bbbba baaaa ababb
aaaaa abbab babbb abaab babaa aabba abaab abb
EACTAG106bV {0} ; 1230
aaabb baabb abaaa babba bbabb bbbba aabab babab ababb bbbaa
aabab babba ababb baabb bbbaa babaa baaab aabaa bbbab bbaba
bbaaa bbbab abbab aabbb babb- aabab baaba bbbbb bbaaa abbbb
aaaaa abbaa babaa abbbb aaaaa aabba baaba aba
EAGTCA126V {0} ; 1287
aaaba aaabb baaab baaba bbaab babba aabab babab ababa bbbab
aabab baaba ababb baabb aabaa babaa babaa abbab abaab bbaba
bbaba bbbbb abbaa aabbb bbba- babaa bbabb bbbbb baaaa aabbb
aaaaa abbaa abbba abbbb aabab aabbb bbabb aab
EACTGA286V {0} ; 1373
bbaab aaaab bbabb aaaaa abbbb ababa babab aabba baaba baabb
abaa- aabbb bbbbb bb-aa bbbba a-baa aabba aaaab bbbab bbbbb
babaa babba aabaa baaba bbbaa aabba abbaa ababb babab aabab
abaab abbab bbbaa bbaab aabab bbaab aaaab aba
EAGTGC42V {0} ; 1386

```
 aaabb aaaba abaaa bbbba bbabb bbbba aabab babab ababb bbbbb
 aaba- babba ababb ba-bb bbbaa b-baa baabb aabaa bbbab bbbba
 abbaa bbabb abbab aabbb babbb aabab bbaba bbbbb bbaaa abbbb
 aaaaa abbaa babaa abbba baaaa aabba baaba aba
ECGTCA394V           {0} ; 1434
 abaaa aabba bbabb bbabb aabab bbbbb bbaaa aaabb bbabb aaabb
 bbba- bbbab abbab ba-bb bbaba b-aba bbabb aaabb bbbab bbaba
 babaa babaa abbaa babbb bbabb abaaa aabaa abbaa babaa bbbbb
 aaabb abbbb babaa aaaab babaa baaaa abaaa aab
EAGTAA69G            {0} ; 1499
 aaabb aa-ba abaaa -bbba bbab- bbbba aaba- bab-b ab-bb b-bbb
 -abab babba a-abb baabb bb--a ----a baa-b -a--- ----b bbbaa
 bbaaa bbabb abbab aabbb babbb aabab bbaba bbbbb bbaaa abbbb
 aaaaa abbaa babaa a--b- b-aaa aabaa baaba aba
EAGTAA195G           {0} ; 1509
 aaabb ba-bb abaaa -bbba bbab- bbbba aaba- bab-b ab-bb b-baa
 -bbab babba a-abb baabb bb--a ----b baa-b -a--- ----b bbaaa
 bbaaa bbbab bbbab aabbb babbb aabab baaba babbb bbaaa abbbb
 aaaaa abbaa babaa a--b- a-aaa aabba baaba aba
ECGTGA252G           {0} ; 1546
 aaaba ba-bb abaaa -aabb bbabb bbbba aaba- babab ababb bbbab
 aabab baaba ababa baabb bb--a ---aa babaa aabaa bbbab bbaba
 bbaaa bbbab abbab aabbb babbb aabaa bbaba bbbbb bbaaa abbbb
 aaaaa abbaa aabaa abbbb a-aab aabba baaba aab
ECGTGA260V           {0} ; 1547
 ababa aa-ba baaab -aaab bbaaa babba baba- babab ababa abbab
 aabab baaba bbabb baabb ba--a ---aa babab abbab abbab bbaba
 babba bbbbb abbaa aabbb bbbaa babba bbabb babbb baaaa aabbb
 aaaaa abbaa abbba abbbb a-bab aabbb bbabb aab
SSR_45               {0} ; 1577
 bbaab aaaab bbabb aaaaa abbba bbaba babab aabba baaba bbabb
 abaaa aabab bbbbb bbaab babba abbaa babba baaab bbaab bbbbb
 aabba aabba aaaaa baaba bbbaa aabba abbab ababb babab aabab
 abaab abbab baba- bbaab aabba aaaab aaaab ab-
SSR_778              {0} ; 1595
 aa-ab bbbbb abbaa babba bbaaa ab--- -aaab babab ab--- --baa
 ba-aa bbbaa ababb baaaa abbaa baaaa aaaab -abaa b-baa bbaba
 bbaaa bbbab abaab aaaab babbb aabab bbaba aaaab bbaba baabb
 aaaaa abb-a aaaa- abbbb aaaa- -abba baaba b--

LG11:
EGAMGA66V            {0} ; 3
 bbabb babab babbb bbbab bbbba aabab abbba babaa aaaba bbabb
 bbbba babba ababb bbaaa aaaab bbbbb abbba bbbbb bbbb- -----
 ----- ----- ----- ----- ----- ----- ----- ----- ----- -----
 ----- ----- ----- ----- ----- ----- ----- ---
ECAMCG98G            {0} ; 56
 aaaba babba bbaab aaaba bbbaa abbaa bbaba abbba abaaa babbb
 bbaaa aabaa babba bbbaa baabb abaaa aabaa aaaaa abab- -----
 ----- ----- ----- ----- ----- ----- ----- ----- ----- -----
 ----- ----- ----- ----- ----- ----- ----- ---
ECGMCG244G           {0} ; 92
 bbbba babbb aaaaa babaa aaabb aaabb baaaa aaaba bbabb babbb
 abaab babba aabbb baaba baaaa bbabb aabaa aabba abba- -----
 ----- ----- ----- ----- ----- ----- ----- ----- ----- -----
 ----- ----- ----- ----- ----- ----- ----- ---
ECGMCG247V           {0} ; 93
```

```
 bbbba babbb aaaaa babaa aaabb aaabb baaaa aaaba bbabb babbb
 ababb babba babbb baaba baaaa bbabb abbaa aabbb abba- -----
 ----- ----- ----- ----- ----- ----- ----- ----- ----- -----
 ----- ----- ----- ----- ----- ----- ----- ---
EGAMGC308G           {0} ; 125
 bbbab baabb aabbb bbbaa babbb aabaa abbba bbbaa abaaa bbaab
 bbbba aabaa aaaba bbaba aaaaa abbba aabba babba abbba aabba
 bbbab abbbb babba bbbab aaaaa bbaab babab bbbbb aabba bbbab
 babab abbaa aabba abbba bbabb abaaa bbbbb abb
EGAMGC404G           {0} ; 128
 aabaa babba bbaab aaaaa bbbaa abbaa bbaba abbba abaaa babbb
 bbaaa aabaa babba bbbaa baabb abaaa abbaa aaaaa ababa babab
 bbaaa abbbb bbaab bbbbb bbbbb baaaa aabab bbbba abbbb bbbaa
 baabb abbba bbaab babba babab aabbb abaab bba
EGAMGC516V           {0} ; 133
 aaaba baabb aaaba baaab aabab aaaab baaba aabba bbaab bbbaa
 aaaaa babbb aabab babab babab abaaa babaa bbaab abbab aaabb
 bbaab aabbb abbba aaabb bbabb aaaab bbaab babaa aabaa bbbaa
 aaabb aabaa abaab bbbbb baaaa aabbb aabaa aaa
EGAMGC537G           {0} ; 135
 aaaba baabb aaaba baaab aabab aaaab baaba aabba bbaab bbbaa
 aaaaa babbb aabab babab babab abaaa babaa bbaab abbab aaabb
 bbaab abbbb abbba aaabb bbabb aaaab bbaab babaa aabaa bbbaa
 aaabb aabaa abaab bbbba baaaa aabbb aabaa aaa
EACMCC302G           {0} ; 221
 aabaa baabb aaaba bbaab aabab aaaab abaaa aabba bbaab bbbaa
 aaaaa babbb aaabb bbaba babab abaaa babaa bbaaa abbab aaabb
 bbaab aabbb abbba aaabb bbabb aaaab bbaab babaa aabaa bbbaa
 aaabb aabaa abaab bbbba baaaa aabbb aabaa aaa
EACMCC352G           {0} ; 223
 baabb babbb abaab aabba babba abbaa bbabb bbbba abbaa baabb
 bbbaa aabaa abbba abbba baabb abaaa aabaa aaaba ababa aabab
 bbbaa abbbb bbabb bbbab bbbbb babaa aabab bbbba abbaa bbbaa
 baaab abbbb bbaab babba babab aabbb abbaa baa
ECAMGG158V           {0} ; 254
 bbabb baaab aabbb bbbab bbbba aaaab baaba babaa aaaba ababb
 bbbba babba babab babaa aaaab bbbbb aabba bbbaa abbba aabba
 bbbab ababb babba bbbab aaaba bbaab bbbab abbba babaa bbbaa
 babab aabab aabbb aabbb bbabb abbba aabbb bbb
ECAMGG241G           {0} ; 260
 aabab baabb aaaba bbaaa aabab aaaab ababa aabba bbaab bbbaa
 aaaaa babbb aaabb bbaba babab abaaa babaa bbaab abbab aaabb
 bbaab aabbb abbba aaabb bbabb aaaab bbaab babaa babaa bbbaa
 aaabb aabaa bbaab bbbba baaaa aabbb aabaa aaa
ECAMGG300G           {0} ; 266
 aabab baabb aaaba bbaab aabab aaaab ababa aabba bbaab bbbba
 babaa babbb aaabb bbaba babaa bbaaa babaa bbaab bbbbb aaabb
 bbaab aabbb abbaa aaabb bbabb aaaab bbaab aabaa baaaa bbbaa
 aaabb babaa bbaab bbbba baaaa aabbb aabaa bab
ECGMTA382V           {0} ; 322
 bbabb baaaa aabbb bbbab bbbba aaaab baaba babaa aabba bbabb
 bbbba babbb aabab babaa aaaab bbbbb aabba bbbaa abbaa aabba
 bbbab ababb bbbba bbbab aaaaa abbab babbb abbbb aabaa bbbaa
 aabaa aabbb aabbb aabbb bbabb abbba aabbb bbb
EACMGA354V           {0} ; 369
 aabaa bbabb aaaba bbaab aabab aaaab baaba aabba bbaab bbaaa
 aaaaa babbb aabab babab babab abaaa baaaa bbaab abbab aaabb
```

bbaa- aabbb abbba aaabb bbabb aaaab bbaab babaa aabaa bbbaa
aaabb aabaa bbaab bbbbb baaaa aabbb aabaa aaa
EACMGA452G {0} ; 380
bbbaa babbb aaaaa babaa aaabb aaabb babaa aaaba bbaab bbbbb
abbab babbb babab baabb baaaa bbabb abbaa aaaaa abbab aabbb
bbaa- aabba abbba aaabb bbbbb aaaaa bbabb babab abbab bbaaa
baabb aaaaa bbabb babba bbaba aabbb abaab bba
EACMGA529G {0} ; 385
aaaaa babba bbaab aaaba bbbaa abbba bbbba abbba abaaa babbb
bbaba aabaa babba bbbaa baabb bbaaa abbba aaaaa abbba babab
bbaa- abbab bbabb bbbbb bbbbb bbaaa aabab bbbba abbab bbbaa
baabb abbbb bbbab babba babab babba abaab bbb
EAGMTA178V {0} ; 398
bbabb baaab aabbb bbbab bbbba aaaab ababa babaa aaaba bbabb
bbbba babba ababb b-aaa aaaab bbbbb aabba bbbab abbba aabba
bbba- abbbb babba bbbab aaaba bbaab bbbab aabbb aabaa bbbaa
babab aabab aaaab aabba bbabb abbba aabbb bbb
EACMTC371G {0} ; 505
aaaba babba bbaab aaaba bbbaa abaaa bbaba abbba abaaa babbb
baaaa aabaa babba bbbaa baab- abaaa abbaa aaaaa ababa babab
bbaaa abbbb bbaab bbbbb bbbbb baaaa aabab bbbba abbab bbbaa
baabb abbbb bbaab babaa babab aabbb abaab bba
EACMTC508V {0} ; 526
abbba babbb baaab babab aaaab aaaba baaaa bbaba aaaba babbb
bbaaa aabaa aabbb bbaba baab- ababb babba aabba abaaa aabbb
bbaaa abbab babaa babba babbb abbaa abbab aabba bbbbb bbaaa
bbabb aaabb baaab baaba bbaab babbb bbaab abb
EACMTC508G {0} ; 527
abbba babbb baaab babab aaaab aaaba baaaa bbaba aaaba babbb
bbaaa aabaa aabbb bbaba baab- ababb babba aabba aaaaa aabbb
bbaaa abbab babaa babba babbb abbaa abbab aaaba bbbbb bbaaa
bbabb aaabb baaab baaaa bbaab babbb bbaab abb
ECAMCT80G {0} ; 541
aaaba babbb abaab aaaba babab abbaa bbaba babba bbaaa baaaa
bbaaa aabab babba bbbaa baab- abbaa bbaba abaab bbaba aaaaa
ababa abbab bbaaa bbbbb bbbaa baaaa aabab bbbba abaab baaaa
babaa abbbb bbaab babab babab aabbb abaaa baa
ECAMCT89V {0} ; 542
aaaba bbbbb abaaa abbaa babba abbab bbbbb bbbba abbab baaaa
bbbaa aabaa babba abbab baab- bbaba aabaa aaaba ababa aabab
bbbab ababa baabb bbbab bbbaa baaaa aabab bbbba aabaa babaa
babab aaaab bbaaa babba bbaab aabaa aabba aba
ECAMCT394V {0} ; 585
bbbaa aaabb aaaaa baaab aaaab aaabb baaba aabba bbaab bbbaa
bbbab babbb babab baabb baaa- ababb abbaa abaab abbbb aabbb
bbaab aabba abbba aaabb bbbbb aaaab bbabb babab aabaa bbbaa
baabb aabaa bbabb babbb bbaaa aabbb aabab bba
ECGMTT112V {0} ; 599
abbba aabbb baa-b babbb abbbb aabba abaaa bbaba aaaaa babbb
bbaaa abbaa bbbbb bbaba baa-- bbabb bbbba abbba abaaa bbbbb
bbaba abbab abbaa babba bbbbb abbaa bbaab aaaab bbbaa abaaa
ababb aabbb bbbaa babab aaaab babbb bbaab aab
EACMCG142V {0} ; 635
aabaa baaab aaaba baaab aabab aaaab ababa aabba bbaab bbbab
aaaaa babbb aabab babbb babab aaaaa baaaa bbaab abbab aaabb
bbaaa aabbb abbaa aaabb bbabb aaaaa bbaab babaa aabaa bbbaa
aaaab abbaa ababb bbbbb baaaa babbb babaa aaa

```
EACMCG384G           {0} ; 658
 babbb baabb abaaa aabba babba abbaa bbbbb bbbba abbaa bbabb
 bbbaa aabaa babba abbab baabb abaaa aabaa aabba ababa aabab
 bbbaa abbbb bbabb bbbab bbbaa babaa aabab bbbba abbaa babaa
 baaab abbbb bbaab babba baaab aabbb abbba baa
EAGMGG93G            {0} ; 667
 bbbaa baabb aaaaa babaa aaaab aaabb baaba aabba bbaab bbbaa
 abaab babbb aabab baabb baaab aaabb abbaa abaab abbab aaabb
 bbaab aabba abbba aaabb bbbbb aaaab bbabb baaab aabaa bbbaa
 baaab aabaa bbabb babba abaaa aabbb aabab bba
ECGMGC352G           {0} ; 720
 abbbb babbb babaa babaa aaaab aaaba abaaa baabb ababa babbb
 abaaa aabba babbb bbbaa baaaa bbabb abbba aabba abaaa aabbb
 bbaaa bbbab babba babba bbbbb aabab ababb aabbb bbbbb bbbaa
 bbabb aaaba bbaab baaba bbaaa babbb bbaab abb
ECGMGC353G           {0} ; 721
 abbbb babbb babaa babaa aaaab aaaba abaaa baaba ababa babbb
 abaaa aabba babbb bbbaa baaaa bbabb abbba aabba abaaa aabbb
 bbaaa bbbab babba babba bbbbb aabaa ababb aabbb bbbbb bbbaa
 bbabb aaaba bbaab baaba bbaaa babbb bbaab abb
ECGMGC357V           {0} ; 722
 abbbb babbb baaaa babaa aaaab aaaba abaaa baaba aaaba babbb
 abaaa aabba babbb bbaba baaaa baabb abbba aabba abaaa aabbb
 bbaaa bbbab babba babba abbbb aabaa ababb aabbb bbbbb bbbaa
 bbabb aaaba bbaab baabb bbaaa babbb bbaab abb
EACMGG79G            {0} ; 736
 abbbb babbb baaba babaa aaaab aaaba baaaa baaba ababa b-bbb
 abaaa aabba babbb bbaba baaaa bbabb abbba aabba abaaa aabbb
 bbaaa bbbab babba babba bbbbb aabaa a-abb aabbb bbbbb bbbaa
 bbabb aaaba bbaab baaaa bbaaa babbb bbaab abb
EACMGG226G           {0} ; 762
 bbbaa baaba aaaba baaab aaaab aaaab baaaa aabba bbaab b-baa
 abbab babbb babab babbb babab ababb abbaa abaab abbbb aabbb
 bbaab aabbb abbba aaabb bbbbb aaaab b-abb babab aabaa bbbaa
 aaabb aabaa bbaab babab baaaa aabbb aabaa aba
EAGMCT47V            {0} ; 799
 baaba babbb abaab aabba babba abbaa bbabb bbbba abbaa b-abb
 bbaaa aabaa babba abbab baabb abaaa aabaa aaaba ababa aabab
 bbbaa abbbb bbabb bbbab bbbbb babaa a-bab bbbba abbaa bbbaa
 baaab abbbb bbaab babba babab aabbb abbaa bab
EAGMCT521G           {0} ; 843
 bbabb baabb aabbb bbbaa babbb aabaa babaa bbbaa abaaa b-aab
 bbbba aabaa aaaba bbaba aaaaa bbbba aabba babba abbba aabba
 bbbab abbbb babba bbbab aaaaa bbaab b-bab bbbbb aabaa bbbaa
 babab abbab aabba abbaa bbabb abaaa aabbb aab
EAGMCT530V           {0} ; 844
 bbabb baabb aabbb babaa babbb aabaa babba bbbaa ababa b-aab
 bbbba aabaa ababa bbaba aaaaa bbbba aabba bbbbb abbba aabba
 bbbab abbbb babba bbbab aaaaa bbaab b-bab bbbbb aabaa bbbaa
 babab abbab aabba abbba bbabb abaaa aabbb abb
EACMGC59V            {0} ; 868
 ababa babbb baaab aaaba bbbaa aabba baaaa abbba abaaa babbb
 bbaba aabaa babba bbbaa baaba bbaab bbbba abbba abbba bbbab
 bbaaa abbab ababb babbb bbbbb bbbaa aabab babba abaab abaaa
 bbabb aabaa bbaba ababb baaab aabba abbbb baa
EACMGC130G           {0} ; 877
 baabb babbb abaaa aabba babba abbaa bbbbb bbbba abbaa bbabb
```

bbbaa aabaa babba abbab baabb aaaaa aabaa aabba ababa aabab
bbbaa abbbb bbabb bbbab bbbaa babaa aabab bbbba bbabb abaaa
baabb babbb abaaa bbaaa abaab aaaba bbbbb baa
EACMGC180G {0} ; 883
aabaa baabb aabaa bbaab aabab aaaab baaba aabba bbaab bbbaa
aaaaa babbb aabab babab babab abaaa babaa bbaab abbab abbbb
bbaab aabbb abbba aaabb bbabb aaaab bbbab babaa bbbbb bbaaa
aaabb baaba aaaaa abaaa bbaaa aabba abaaa bab
EACMGC301V {0} ; 899
bbabb ababb abbba abbaa babbb aabaa abbbb bbbba abbaa bbaab
bbbaa aabaa baaba ababb baaaa abbaa aabaa aabba ababa aabab
bbbab abbbb baabb bbbab bbbaa baaaa aabab bbbba aaabb abbab
babaa aaabb aaaba bbaab abaab ababa bbabb bab
EACMGC565G {0} ; 927
ababa babbb baaab aabbb babab aabba baaaa abbba abaaa babbb
bbaba aabaa bbbba bbaba baaba bbaab bbbba abbba abaaa bbbbb
bbaaa abbab abbaa babbb bbbbb abbaa bbbab babba abaab bbaaa
bbabb abbaa baabb bbaba baaab aabba abbbb baa
EACMGC568V {0} ; 929
ababa babbb baaab aabbb babab aabba baaaa abbba abaaa babbb
bbaba aabaa bbbba bbaba baaba bbaab bbbba abbba abaaa bbbbb
bbaaa abbab abbaa babbb bbbbb abbaa bbbab babba abaab bbaaa
bbabb abbaa baabb bbabb baaab aabba abbbb baa
EAGMCA55V {0} ; 932
baaba babbb abaab aabba babba abbaa bbabb bbbba abbaa baabb
bbaaa aabaa babba abbab baabb abaaa aabaa aaaba ababa aabab
bbbaa abbbb bbabb bbbab bbbbb babaa aabab bbbba bbabb ababa
baabb babba abaaa bbaab bbaab aaaba bbbbb baa
EAGMCA323V {0} ; 964
aabaa baabb aabaa bbaab aabab aaaab ababa aabba bbaab bbbaa
aaaaa babbb aabab babab babab abaaa babaa bbaab abbab aaabb
bbaab abbbb bbbba aaabb bbabb aaaab bbaab babaa bbbbb bbaaa
aaabb baaba aaaaa abaab bbaaa aabba abaaa baa
EAGMCA497V {0} ; 974
aabba babaa baaab babbb aaaab aaaba abaaa bbaba aaaba babbb
bbaaa aabaa babbb bbaba baaba bbaba babba aabaa abaab abbbb
bbaaa abbab babaa babba bbbbb abbaa bbbab aabba bbaab bbbab
bbabb bbbaa babbb bbbbb baaab babba aabbb baa
ECGMCA372G {0} ; 995
aabaa baabb aabaa bbaab aabab aaaab baaba aabba bbaab bbbaa
aaaaa babbb aabab babab babab abaaa babaa bbaab abbab aaabb
bbaab aabbb abbba aaabb bbabb aaaab bbaab babaa bbbbb bbaaa
aaabb baaba aaaaa abaaa bbaaa aabba abaaa baa
ECGMGG48V {0} ; 1088
ababa babbb baaab aabbb babab aabba abaaa abbba abaaa babbb
bbaba aabaa bbbba bbaba baaba bbaab bbbba abbbb abaaa bbb-b
bbaaa abbab abbaa babbb bbbbb abbaa bbbab babba bbbab abaaa
baabb aabbb bbaaa baabb baaab babba bbaab aab
ECGMGG215V {0} ; 1094
ababa babbb baaab aabbb babaa aabba abaaa abbba abaaa babbb
bbaba aabaa babba bbbaa baaba bbaab bbbba abbba abbba bbb-a
bbaaa abbab ababb babbb bbbbb bbbaa aabab bbbba bbbab bbaaa
baabb abbbb bbaaa baabb baaab babba abaab bab
ECGMGG358V {0} ; 1101
aabab baabb aaaba baaab abbab aaaab ababa aabba bbaab bbbaa
aaaaa babbb aabab babab babab abaaa babaa bbaab abbab aba-b
bbaaa aabbb abbba aaabb bbabb aaaab bbaab babaa aabaa bbbaa

aaabb aabaa abaab bbbbb baaaa aabbb aabaa aaa
ECGMGG366G {0} ; 1102
aabab baabb aaaba baaaa aabab aaaab ababa aabba bbaab bbbaa
aaaaa babbb aabab babab babab abaaa babaa bbaab abbbb aaa-b
bbaaa aabbb abbba aaabb bbabb aaaab bbaab babaa aabaa bbbaa
aaabb aabaa abaab bbbba baaaa aabbb aabaa aaa
EACTCC93G {0} ; 1118
ababa babbb baaab aabbb babab aabba baaaa abbba abaaa babbb
bbbaa aabaa bbbba bbaba baaba bbaab bbbba abbba abaaa bbbbb
bbaaa abbab abbaa babbb bbbbb abbaa bbbab babba bbbab abaaa
baabb aabbb bbaaa baabb baaab babba bbaab aab
EAGTTA232V {0} ; 1166
bbabb ababb abbba abbaa babba abbaa bbbbb bbbba abbaa bbaaa
bbaba aabaa babba abaab baaab bbaaa aabaa aabba ababa aabbb
bbbab abbbb baabb bbbab bbbaa baaaa aabab bbbba abbaa babaa
babab abbab bbbba babba bbabb aaaba aabba aab
EACTAG324V {0} ; 1260
aaaba babba bbaab aaaba bbbaa abbba bbaba abbba abaaa babbb
bbbaa aabaa babba bbbaa baabb bbaaa abbba aaaab ababa babab
bbaaa abbab bbabb bbbbb bbbb- bbaaa aabab bbbba abbab bbbaa
baabb abbbb bbaab babba babab babab abaab bba
EACTAG432G {0} ; 1277
bbabb baabb aabbb bbbaa babbb aabaa abbaa bbbaa ababa bbaab
bbbba aabaa ababa bbaba aaaaa bbbab aabba bbbba abbba aabba
babab abbbb babba bbbab aaaa- bbaab babab bbbbb aabaa bbbaa
babab abbab aabba abbba bbabb ababa aabbb abb
EACTAG446V {0} ; 1278
ababa babbb baaab aaaba bbbaa aabaa baaaa abbba abaaa babbb
bbbaa aabaa babba bbbaa baaba bbaba bbbba abbba abbba bbbab
bbaaa abbab ababb babbb bbbb- bbbaa aaaab bbbba bbbab bbaaa
baabb aabbb bbaaa baaba baaab babba abaab bab
ECGTAC82G {0} ; 1322
bbabb baaab aabbb bbbab bbbba aaaab ababa babaa aaaba bbabb
bbbba babba ababb bbaaa aaaab bbbbb aabba bbbaa abbba aabba
babab ababb babba bbbab aaab- bbaab bbbab abbbb aabaa bbbaa
babab aabab aabbb aabba bbabb abbba aabbb bbb
EACTGA113G {0} ; 1350
aaaba babba bbaab aaaba bbbaa abbaa bbaba abbba abaaa babbb
bbaa- aabaa babba bb-aa baabb a-aaa abbaa aaaaa ababa babab
bbaaa abbbb bbaab bbbbb bbbbb baaaa aabab bbbba abbab bbbaa
baabb abbbb bbaab babba babab aabbb aaaab bbb
EACTGA165V {0} ; 1359
aaaba babba abaab aaaba babaa abbaa bbaba bbbba abaaa babbb
bbaa- aabaa babba bb-aa baabb a-aaa abbaa aaaab ababa aabab
bbbaa abbbb bbaab bbbab bbbbb baaaa aabab bbbba abbab bbbaa
baabb abbbb bbaab babba babab aabaa aabaa baa
EACTGA196V {0} ; 1360
ababa babbb baaab aabbb babab aabba baaaa abbba abaaa babbb
bbba- aabaa bbbba bb-ba baaba b-aab bbbba abbba abaaa bbbbb
bbbaa abbab abbaa babbb bbbbb abbaa bbbab babba bbbab abaaa
baabb aabbb bbaaa baabb baaab babba bbaab aab
EACTGA268V {0} ; 1369
ababa babbb baaab aabbb babab aabba baaaa abbba abaaa babbb
bbba- aabab bbbba bb-ba baaba b-aab bbbba abbba abaaa bbbbb
bbbaa abbab abbaa aabbb bbbbb abbaa bbbab babba abbaa bbaaa
baabb aaaab bbaaa baaab baaab babba bbaab bab
EACTTC50V {0} ; 1438

```
 bbbaa ba-bb aaaaa -bbab aaaab aaabb baab- aabba bbaab bbbbb
 ababb babbb babab baabb ba--a ---bb abbaa abaaa abbbb aabbb
 bbbab aabba abbba aaabb bbbbb aaaab bbabb babab aabab bbbaa
 baabb aaaaa bbabb babbb b-aba aabbb aabab bba
ECGTGA144V          {0} ; 1535
 aaaba ba-ba bbaab -aabb bbbaa abbaa bbab- abbba abaaa babbb
 bbaaa aabaa babba bbbaa ba--b ---aa abbaa aaaaa abbba babab
 bbbaa abbbb bbaab bbbbb bbbbb baaaa aabab bbbba abbab bbbaa
 baabb abbbb bbaab babba b-bab aabab abaab bba
ECGTGA151V          {0} ; 1536
 bbabb ba-ab aabbb -bbab bbbba aaaab abab- babaa aaaba bbabb
 bbbba babba ababb bbaaa aa--b ---bb aabba bbbaa abbba aabba
 bbbab ababb babba bbbab aaaba bbaab bbbab abbbb aabaa bbbaa
 babab aabab aabbb aabba b-abb abbba aabbb bbb
ECGTGA389V          {0} ; 1559
 aabaa ba-bb aaaba -baaa aabab aaa-b baab- aabba bbaab bbbaa
 aaaaa babbb aabab babab ba--b ---aa babaa bbaab abbab bbabb
 bbbab aabbb abbba baabb bbabb aaaab bbaab babaa aabaa abaaa
 aaabb aabaa baaab bbbab a-aaa aabbb aaabb bba
ECGTGA395G          {0} ; 1560
 baaba ba-bb abaab -abbb babba abbba bbab- bbbba abbaa baabb
 bbaaa aabaa babba abbab ba--b ---aa aabaa aaaba abbba aabab
 bbaaa abbbb bbabb bbbab bbbbb babaa aabab bbbba abbaa bbbaa
 baaab abbbb bbaab babba b-bab aabab abbaa baa
SSR_752             {0} ; 1591
 bbabb baaab aabbb bbbab bbbba bbbab abbba babaa aaaba bbabb
 bbbba babba ababa bbaaa aaaab bbbbb aabba bbbaa abbba aabba
 bbbab bbbbb ba-aa bbbab aaaba bbaab bbbab abbbb aab-- ----a
 b--ab ab--- ---b- abbba bba-b abbba aabbb a--
SSR_819             {1} ; 1601
 babaa baaab aaaab abaaa bbabb --baa abbbb abbab aabaa abaab
 baaab aba-b aba-a abbaa abbbb aabb- baabb abaab baaaa bbaaa
 aabbb aaa-- ----b abaaa aaaaa bbabb aabaa ababa baaaa aaabb
 abbab bbbaa aabb- abaab aabab abaaa babba bb-
SSR_835             {0} ; 1602
 aaaba babba bbbab aaaba bbbaa abbaa bbaba abbba abaaa bbaaa
 abaaa aabaa babba bbbab baabb abbaa abbaa aaaaa ababa babab
 aaaaa bbbbb bbbaa baabb bbbbb baaaa babab bbbba abbab bbbaa
 baabb abbba aaaa- babba babab aabab abaab bb-

LG12:
EGAMGA353V          {0} ; 35
 baaba baaba abaab abbbb bbaaa ababa aabab bbbba aabaa bbbbb
 ababa babba babab aaaaa babab abaaa ababa baabb aaba- -----
 ----- ----- ----- ----- ----- ----- ----- ----- ----- -----
 ----- ----- ----- ----- ----- ----- ----- ---
ECAMCG108V          {0} ; 59
 baaba baaba ababb abbbb aaaaa abbba aabbb bbbba aabaa bbbbb
 bbaba babaa babab aaaaa babbb abbab ababa aabbb aaba- -----
 ----- ----- ----- ----- ----- ----- ----- ----- ----- -----
 ----- ----- ----- ----- ----- ----- ----- ---
ECAMCG131G          {0} ; 61
 baaba baaba ababb abbbb aaaaa abbba aabab bbbba aabaa bbbbb
 bbaba babaa babab aaaaa babbb abbab aaaba aabbb aaba- -----
 ----- ----- ----- ----- ----- ----- ----- ----- ----- -----
 ----- ----- ----- ----- ----- ----- ----- ---
ECAMCG144G          {0} ; 63
```

```
 baaba baabb bbabb abbba aaaaa abbba aabab bbaaa aaaaa bbbbb
 bbaba baaaa babab aaaaa babbb abbab aaaba aabba bbba- -----
 ----- ----- ----- ----- ----- ----- ----- ----- ----- -----
 ----- ----- ----- ----- ----- ----- ----- ---
EGAMGC197V           {0} ; 118
 bbbaa baaba ababb abbbb babaa ababa babaa bbbbb abbaa babab
 ababa babba babaa aaaaa baaaa abaaa aaaba baabb aabaa aaabb
 aabba aabba baaab ababa bbaba aabbb ababb babaa aabab bbbaa
 abbaa bbaba bbbaa bbabb babba aaaab baaaa aab
ECGMCC85V            {0} ; 179
 babba baabb bbabb abbba aaaaa abbba aabbb bbbba aaaaa babbb
 bbaba baaaa babab aaaab babbb abbab aaaba aabbb abbaa aaaaa
 aabba abb-b aabba ababb abbba aabab ababb aabaa abbab bbbab
 abbba bbbba bbaba bbbab bbaaa aaaaa babaa abb
EACMCC246V           {0} ; 218
 bbaba aaaab bbabb aaaba aaaab abbaa aabbb baaab aaaab babbb
 baaab aaaaa ababa aabaa babbb abbab baaab aabbb babaa aabba
 baaba aabbb abbba abbab abbba bbbab aabba aaaaa bbbab bbaba
 bbbaa babaa ababa bbaab abaaa baaaa aabab aaa
EACMCC533V           {0} ; 237
 baaba baabb bbabb abbba aaaaa abbba aabbb abbba aaaaa bbbbb
 bbaba baaaa ababa baaaa babbb abbab aaaba aabbb abbaa aaaba
 aabba aabbb aabba ababb ababa aabab aaabb aabaa abbab bbbab
 abbba bbbba bbaaa bbaab abaaa aaaaa baaab aab
ECAMGG379G           {0} ; 278
 bbaba babba bbbbb babba aabba ababa abbaa aabbb bbabb babab
 abaab bbbbb aaaaa babba aabba baaba ababb bbbbb babba abbba
 aabbb babba aaaaa abaab bbaaa bbbba ababb abbaa aaaba aaaaa
 bbbaa bbbaa abbba abbaa aabba bbaab aabab baa
ECGMTA222G           {0} ; 312
 bbaba bbaba aabbb abbbb babba abbba bbbaa abbbb abbaa babab
 abaaa babbb ababa baaaa baaaa ababa aaaba abaab aaaba aabbb
 aabbb aabba aaaab ababa bbaaa bbbbb ababb aabaa aabaa babaa
 abbaa bbbba aabba bbbba aabba bbaab babaa aaa
ECGMTA386G           {0} ; 323
 baaba baabb bbabb abbba aaaaa abbba aabbb bbaaa aaaab babbb
 bbaba baabb bbaba baaaa babbb bbbab aaaba aabbb bbbaa aaaba
 aaaaa aabbb abbba abaab abaaa abbab aaabb aabaa abbab bbbab
 abbba babba aaaaa bbaaa abaaa aaaaa baaab aab
EACMGA251G           {0} ; 353
 baaaa baabb bbabb abbba aaaaa abbba aabbb abbba aaaaa bbbbb
 bbaba baaaa babab abaaa babbb abbab aaaba aabbb abbaa aaaba
 aabb- aabbb aabba ababb abaaa aabab aaabb aabaa abbab bbbab
 abbba bbbba bbaaa bbaaa bbaaa aaaaa baaab aab
EACMGA253V           {0} ; 354
 baaaa baabb bbabb abbba aaaaa abbba aabbb abbba aaaaa bbbbb
 bbaba baaaa babab aaaaa babbb abbab aaaba aabbb abbaa aaaba
 aabb- aabbb aabba ababb ababa aabab aaabb aabaa abbab bbbab
 abbba bbbba bbaaa bbaab bbaaa aaaaa baaab aab
EACMGA283G           {0} ; 361
 bbaaa baaba ababb abbbb aabaa ababa baaaa bbbbb abbaa babab
 ababa babba babaa abaaa baaaa abaaa aaaba baabb aabaa aaabb
 abbb- aabba baaab ababa bbaaa aabbb ababb babaa aabab bbbaa
 abbaa bbaba bbbaa bbaba babba aaaab baaaa aab
EAGMTA65G            {0} ; 393
 bbaba baaba ababb abbbb babaa ababa babaa bbbbb abbaa babab
 ababa babba babaa a-aaa baaaa abaaa aaaba baabb aabaa aaabb
```

```
 aabb- aabba baaab ababa bbaaa aabbb ababb babaa aabab bbbaa
 abbaa bbaba bbbaa bbaaa babba aaaab baaaa aab
EACMTC366V          {0} ; 503
 bbabb babba babbb babba aabba bbbba babab aabbb bbabb babab
 abaab bbbab baaab abaab aaab- babba bbabb ababa babab abbba
 babbb babaa bbaba abbab bbaaa bbbba ababa abbaa abbba aaaab
 bbbaa abaaa aabba aabbb aabba bbaab babba bba
EAGMGG95G           {0} ; 668
 bbaba aaabb bbabb aaaba aaaaa abbaa aabbb bbaab abaaa babbb
 baaab aaaaa babab abaaa babbb abbab aaabb aabba babaa aabba
 aaaba aabbb abbba abaab abbaa bbbab aabba aaaaa bbbab bbaaa
 bbbba babaa ababa bbaaa abaaa baaaa aaaab aaa
ECGMGC241G          {0} ; 713
 bbaba bbaba aabab abbbb aabba abbba bbbaa abbbb aabaa babab
 abaaa babbb babab aaaaa aabaa baaba ababa bbabb ababa aabbb
 aabbb aabba aaaab ababb bbaaa bbbbb ababb abbba aaaaa aabaa
 abbaa bbbaa abbba abbba aabba bbaab babaa aaa
EACMGG251V          {0} ; 766
 baaba baabb bbabb abbba aaaaa abbba aabbb abbba aaaaa b-bbb
 bbaba baaaa babab aaaaa babbb abbab aaaba aabbb abbaa aaaba
 aabba aabbb aabba ababb ababa aabab a-abb aabaa abbab bbbab
 abbba bbbba bbaaa bbabb abaaa aaaaa baaab aab
EACMGG326G          {0} ; 777
 abaaa babba bbbbb babbb aabba ababa babab aaaab bbbbb b-bab
 bbabb bbbbb aabab abaab aabba baaba ababb abbbb bbbaa abbaa
 aabbb babba aabaa abbaa bbaaa baaba a-aba abbbb bbbba baaab
 bbbaa abaab abbba abbab aabba abbba ababb bab
EACMGG327V          {0} ; 778
 ababa babba bbbbb babbb aabba ababa babab aaabb bbabb b-bab
 bbaab bbbbb aabab abaab aabba baaba ababb abbbb bbbaa abbaa
 aabbb babba baaaa abbab bbaba bbaba a-aba abbaa bbaba baaab
 bbbaa abaab abbba abbbb aabba bbaab aaabb baa
EAGMCT367G          {0} ; 834
 bbaba aaabb bbabb ababb aaaaa abbaa aabbb bbaaa baaaa b-bbb
 baaab baaaa babab abaaa babbb abbab aaabb aabba bbbaa aabba
 aaaba babbb abbba abaab abbaa bbbab a-bba ababa bbbab bbaaa
 abbba babaa ababa bbaab abaaa baaaa aaaab aaa
ECGMGT240G          {0} ; 856
 bbaba bbaba aabbb abbbb babba abbba bbbaa bbbbb abbaa b-bab
 abaaa babbb babab aaaaa baaaa ababa aaaba bbabb aaaba aabbb
 aabbb aabba aaaab ababa bbaaa bbbbb a-abb aabaa aabba babaa
 abbaa bbbba abbba bbbaa aabba bbaab babaa aaa
ECGMGT248V          {0} ; 858
 bbaba bbaba aabbb abbbb bbbba abbba bbbaa bbbbb abbaa b-bab
 abaaa babbb babab aaaaa baaaa ababa aaaba bbabb aaaba aabbb
 aabbb aabba aaaab ababa bbaba bbbbb a-abb aabaa aabba babaa
 abbaa bbbba abbba bbbba aabba bbaab babaa aaa
ECGMGT252G          {0} ; 859
 bbaba bbaba aabbb abbbb babba abbba bbbaa bbbbb abbaa b-bab
 aaaaa babbb babab aaaaa baaaa ababa aaaba bbabb aaaba aabbb
 aabba aabba baaab ababa bbaaa abbbb a-abb aaaaa aabba babaa
 abbaa bbbba abbba bbbaa aabba bbaab babaa aaa
ECGMGT462V          {0} ; 863
 baaba baabb bbabb abbba aaaaa abbba aabbb bbaaa aaaaa b-bbb
 bbaba baaaa babab aaaaa babbb abbab aaaba aabbb bbbaa aaaba
 aaaba aabbb abbba abaab ababa abbab a-abb aabaa abbbb bbbab
 abbba bbbba abaaa bbabb abaaa aaaaa baaab aab
```

```
EACMGC199G          {0} ; 886
 baaba baabb bbbab abbba aaaaa abbba aabbb abbba aaaaa bbbbb
 bbaba baaaa babab aabaa babbb abbab aaaba aabba abbaa aaaba
 aabba aaabb aabba ababb abaaa aabab aaabb aabaa abbaa babaa
 aaaaa abaaa baaba bbaba bbaaa bbbab bbbab baa
EAGMCA392G          {0} ; 967
 baaba baaba bbbab abbbb aaaaa abbba aabab abbba aabaa bbbbb
 bbaba babaa babab aaaaa babbb abbab aaaba aabba abbaa aaaba
 aabba aabbb aabba ababb bbaaa aabab aaabb babaa abbaa bbaaa
 aaaaa abaaa baaba bbaba bbaaa bbbab bbbab bba
ECGMCA194V          {0} ; 983
 bbaba baaba abbab abbbb babaa ababa babaa bbbbb abbaa babab
 ababa babba babaa aaaaa baaaa abaaa aaaba baabb aabaa aaabb
 aabba aabba baaab ababa bbaba aabbb ababb babaa abbab ababb
 aaaaa bbaaa aaaba ababb bbaaa bbaab babab bba
EACMGT538G          {0} ; 1038
 bbbba bbbba abbbb abbbb bbbaa ababa bbbaa bbbbb abbaa babab
 ababa babba babaa aaaaa baaaa bbaaa aaaba baabb aabba aaabb
 aabba aabba baaab ababa bbaaa aabbb ababb babaa aabab bbbaa
 abbaa bbaba bbbaa bbaba babba aaaab baaaa aab
EAGMTT268G          {0} ; 1067
 bbaba baaba ababb abbbb babaa ababa babaa bbbbb abbaa babab
 ababa babba babaa aaaaa baaaa abaaa aaaba baabb aabaa aaabb
 aabba aabba aaaab ababa bbbaa aabbb ababb babaa aabab bbbaa
 abbaa bbaba bbbaa bbaaa babba aaaab baaaa aab
EACTCC103V          {0} ; 1120
 ababa aabba bbabb babbb aabba ababa babab aaabb bbabb bbbab
 bbaab bbbbb aabab abaab aabba baaba ababb abbbb bbbaa abbaa
 aabbb baaba baaaa aabab bbabb bbaba ababa abbaa bbaba baaab
 bbbab abaab abbba abbab aabba bbabb aaabb baa
EACTCC414V          {0} ; 1147
 bbaba baaba ababb abbbb babaa ababa babaa bbbbb abbaa babab
 abbaa babba babaa aaaaa baaaa abaaa aaaba baabb aabaa aaabb
 aabba aabba baaab ababa bbaba aabbb ababb babaa aabab bbbaa
 abbaa bbaba bbbaa bbaba babba aaaab baaaa aab
EACTAG175G          {0} ; 1243
 baaba baabb bbaab abbba aaaaa abbba aabbb abbba aaaab bbbbb
 bbbaa baaaa babab aaaaa babbb abbba aaaba aabbb abbba aaaba
 aabba aabbb aabba ababb abaa- aabab aabbb aabaa abbab bbbab
 abbbb bbbba bbaaa bbaab bbaaa aaaba aaaab aab
EACTAG490V          {0} ; 1281
 baaba baabb bbabb abbbb aaaaa abbba aabab abbba aabaa bbbbb
 bbbaa baaaa babab aaaaa babbb abbba aaaba aabbb abbaa aaaba
 abbba aaaab aabba ababb bbab- aabab aaabb babaa abbab bbbaa
 abbba bbbba bbbaa bbaab baaaa aaaaa baaab aab
ECGTAC363V          {0} ; 1343
 baaba baaba ababb abbbb baaaa ababa aabbb bbbba aabaa bbbbb
 abbaa babba babab aaaaa babab abaaa aaaba baabb aabaa aaabb
 abbba aabbb baabb ababb bbab- aabbb aaabb babaa abbab bbbaa
 abbaa bbbba bbbaa bbaba babaa aaaab baaaa aab
ECGTAC382G          {0} ; 1345
 baaba baaba abaab abbbb baaaa ababa aabab bbbba aabaa bbbbb
 abbaa babba babab aaaaa babab abaaa aaaba baabb aabaa aaabb
 aabba aabbb baabb ababb bbaa- aabbb aaabb babaa abbab bbbaa
 abbaa bbbba bbbaa bbaba babaa aaaab baaaa aab
EACTGA389V          {0} ; 1379
 baaba baabb bbabb abbba aaaaa abbba aabbb abbba aaaaa bbbbb
```

```
 bbba- baaaa babab aa-aa babbb a-bab aaaba aabbb abbaa aaaba
 aabba aabbb aabba ababb ababa aabab aaabb aabaa abbab bbbab
 abbba bbbba bbaaa bbaab abaaa aaaba baaab aab
ECGTCA178V          {0} ; 1426
 bbaba bbaba aabbb abbbb babba abbba bbbaa abbbb abbaa babab
 abaa- babbb babab aa-aa baaaa a-aba aaaba bbabb aaaba aabba
 aabbb aaaba aaaaa ababa bbaaa baabb ababb aaaaa aabaa babaa
 abbaa abbba abbba bbaba aabba bbaab babaa aaa
ECGTGA457V          {0} ; 1565
 bbaba bb-ba aabbb -bbbb babba abbba bbba- abbbb abbaa babab
 abaaa babbb babab aaaaa ba--a ---ba aaaba bbabb aaaba aabbb
 aabbb aabba aaaab ababa bbaba bbbbb ababb aabaa aabaa babaa
 abbaa bbbba abbba bbbba a-bba bbaab babaa aaa
ECGTGA462G          {0} ; 1567
 bbaba bb-ba aabbb -bbbb babba abbba bbba- abbbb abbaa babab
 abaaa babbb babab aaaaa ba--a ---ba aaaba bbabb aaaba aabbb
 aaabb aabba aaaab ababa bbaaa bbbbb ababb aabaa aabaa babaa
 abbaa bbbba abbba bbbba a-bba bbaab babaa aaa
SSR_76              {0} ; 1578
 baaba baabb bbabb abbbb aaaaa abbba aabab abbba aabaa babbb
 bbaba baaaa babab aaaab babbb abbab aaaba aabbb abbba aaaba
 aabba aaabb aaaba ababb bbaba aabab aaabb babaa abbab bbbaa
 abbba bbbba bbaa- bbaab bbaaa aaaaa baaab aab

LG13:
EGAMGC135G          {0} ; 110
 aaaba bbaba aabab aabab bbaab abbab baaab aabaa bbbba bbbab
 abaab aabab bbaab bbaaa babab aabbb babab aabaa babab babba
 bbabb abbaa aabab abbba bbabb abbaa abbba aaaaa aaaba bbaba
 babba aabaa abaab bbbaa ababa babba abbaa bbb
EGAMGC167V          {0} ; 116
 aaaaa bbaba aabbb abbab aabbb abbab aaaab aabaa babba abbab
 aaaab bbbab abaab bbaaa baaaa aabba babba abbab baaab babba
 bbabb ababa aabba abbba bbabb abaab ababa aaaaa aaaba ababb
 abaab aaaba bbabb babbb bbaba babab abbbb bbb
ECAMCC379G          {0} ; 165
 aaaaa bbaba babaa aaaab bbaab abbab aaaab abbba abbbb abaaa
 abaab aabab bbbab bbaab babab aaabb bbbab babaa -abaa baabb
 ababb abbaa aabab bbaaa bbbba abbab bbbba aaaaa aabaa bbbba
 babba abaaa abaab bbbaa ababa babba abbaa bbb
ECAMCC477G          {0} ; 175
 aaaaa baabb aabab abbab aabab abbab abaab aabaa babba abbab
 aaaaa bbbab abaab bbaaa baaaa aabba babba aabab -aaab babba
 bbabb ababa aabba abbba bbbbb abaab bbaba abaaa aaaaa ababb
 abaab aaaba bbabb baaba bbaba aaaab aabbb baa
ECAMCC606V          {0} ; 176
 aaaaa bbaba aabab abbab aabab abbab aaaab aabaa babba abbab
 aaaab bbbbb abaab bbaaa baaaa aabba babba aabab -aaab babba
 bbabb aaaba aabba abbba bbabb abaab ababa aaaaa aaaba ababb
 abaab aaaba bbabb babbb bbaba baaab abbbb bbb
ECAMCC608G          {0} ; 177
 aaaaa bbaba aabab abbab aabab abbab aaaab aabaa babba abbab
 aaaab bbbbb abaab bbaaa baaaa aabba babba aabab -aaab babba
 bbabb ababa aabba abbba bbabb abaab ababa aaaaa aaaba ababb
 abaab aaaba bbabb babba bbaba baaab abbbb bbb
ECGMCC223V          {0} ; 184
 aabaa bbabb babaa aaaab bbaab abbab aaabb aabba abbbb abaaa
```

```
 abaaa aabbb babab bbaaa babbb ababb bbbab baaaa aabaa baabb
 abbba abb-a aabab bbaaa bbbba abbab bbbba abaab baaaa bbbba
 baaba abaaa aaaab bbbbb ababa babba abbaa bab
ECGMCC307G          {0} ; 185
 aaaaa bbaba babab aaaab bbaab abbab baaab aabba abbbb abaaa
 abaab aabab bbbab bbbab babab aaabb babab babaa aabaa baaba
 bbabb abb-a aabab ababa bbbbb abbab bbbba aaaaa aabba bbaba
 babba abaaa abaab bbbaa ababa babba abbaa bab
EACMCC202V          {0} ; 212
 aaaaa bbaba aabab abbab aabab abbab aaaab aabaa babba abbab
 aaaab bbbab baaab babaa baaaa aabba babba aabab baaab babba
 bbabb ababa aabba abbba bbabb abaab ababa aaaaa aaaaa ababb
 abaab aaabb bbabb babbb bbaba baaab abbbb bbb
EACMCC373G          {0} ; 226
 aaaaa bbaba babab aaaab bbaab abbab abaab aabba abbbb abaaa
 abaab aabab bbabb bbbba babab aaabb babab babaa aabaa baaba
 bbabb abaaa aabab ababa bbbbb abbab bbbba aaaaa aabaa bbaba
 babba abaab abaab bbbaa ababa babba abbaa bab
ECAMGG184G          {0} ; 256
 aaaaa bbaba aabab abbab babab abbaa aaaab aabaa babba bbbab
 aaaab bbbab baaab babaa babaa aabbb babbb aabaa babab babba
 bbabb ababa aabba abbba bbabb abbab abbba aaaaa aaaaa ababb
 babab aabbb ababb babba ababa babbb abbaa bbb
ECGMTA292V          {0} ; 318
 aaaaa bbaba aabab abbab babab abbab aaaab aabaa babba abbab
 aaaab bbbab baaab babaa babaa aabba babba aabbb babab babaa
 bbabb ababa aabba abbba bbaab aabab aaaba aaaaa aaaaa aaabb
 abaab aaabb bbabb aabbb aaaba babba abbba bbb
EACMGA233V          {0} ; 350
 aaaaa bbaba aabab abbab babab abbab aaaab aabaa babba abbab
 aaaab bbbab abaab baaaa babaa aabba babba aabab babab babba
 bbab- ababa aabba abbba bbabb abbab ababa aaaaa aaaaa ababb
 abaab aaabb bbabb babbb ababa babbb abbba bbb
EAGMTA67G           {0} ; 394
 aaabb bbabb baaaa aaaaa bbaba ababb aaaab aabaa bbbbb bbaaa
 ababa aabba babaa b-aaa aabba ababb bbbab baaaa aabaa babbb
 abbb- abbaa aabab bbaaa bbaba bbbab babba bbbbb baaba bbbba
 baabb abaaa aaaab bbbab ababa baaaa abbaa aab
ECGMGA409G          {0} ; 442
 aaaaa bbaba babab aaaab bbaab abbab abaab aabaa bbbbb abaab
 abaab aabab bbbab b-abb babab aaabb babab babaa babab baaba
 bbab- abbaa aabab abbba bbbbb abbab bbbba aaaaa aaaaa bbaba
 babba aabab abaab bbbab ababa babba abbaa bbb
EACMTC332V          {0} ; 497
 aabaa bbaba aabab aabab baaab abaab baaab aabaa babba bbbab
 aaaab aabab bbaab bbaaa baba- aabbb babab aabaa babab babba
 bbabb abbaa aabaa abbba aaabb abbaa abbba aaaaa aaaaa ababa
 babbb aabab abaab bbbbb ababa bbbba abbaa bbb
EACMTC359G          {0} ; 501
 aaaaa bbaba aabab abbab babab abaab aaaab aabaa babba bbbab
 aaaab bbbab abaab bbaaa baba- aabbb babbb aabab babab babba
 bbabb ababa aabba abbba bbabb abbab abbba aaaaa aaaaa ababb
 babab aabab ababb babab ababa babbb abbaa bbb
EACMTC371V          {0} ; 506
 aabaa bbaba babab aaaab bbaab abaab baabb aabba abbbb abaaa
 abaab aabab bbbab bbbab baba- aaabb bbbab babaa aabaa baaba
 bbbbb abbaa aabab ababa bbbbb abbab bbbba aaaaa aabaa bbaba
```

```
 babba abaab ababb bbbbb ababa babba abbaa bab
EACMCG74V            {0} ; 633
 aaaaa bbabb babaa aaaab bbaab abbab aaabb aabba abbbb abaaa
 abaaa aabab babab bbaab babbb aaabb bbbab babaa aabaa baabb
 ababa abbaa aabab bbaaa bbbba bbbab bbbba abaab babaa bbbba
 baaba abaaa abaab bbbab ababa babba abbaa bab
EAGMGG241G           {0} ; 688
 aaaaa baaba aaaab aaaab aabab abbab abaab aabab babbb abbab
 aaaaa bbbab abaaa bbaaa baaaa aabba babba aabaa baabb babba
 bbabb ababa aabba abbba bbbbb bbbab bbaba ababa aaaaa ababb
 abaab aaaba bbabb baaba bbaba aaaab aabbb bba
EAGMCT332G           {0} ; 830
 aaaaa bbaba babab abaab bbaab abbab abaab aabaa bbbbb a-aab
 abaab aabab bbbab bbaab babab aaabb babab babaa babab baaba
 bbabb abbaa aabab abbba bbbbb abbab b-bba aaaaa aaaaa bbaba
 babba abbab abaab bbbab ababa babba abbaa bbb
ECGMGT156G           {0} ; 851
 aaaba bbabb babaa aaaab baaab abbaa aaaab aabba abbbb a-aaa
 abaaa aabbb babab bbaaa babbb ababb bbbab baaaa aabaa baabb
 abbba abbaa aabab bbaaa bbbba abbab b-bba abaab baaaa bbbba
 baaba abaaa aaaab bbbab ababa babba abbaa bab
EACMGC315G           {0} ; 901
 aaaaa bbaba aaabb aabab bbaab abbab baaab aabaa bbbba bbbab
 aaaab aabab bbaab bbaaa babab aabbb babab aabaa babab babba
 bbabb abbaa aabab abbba bbabb abbaa abbba aaaaa bbbba babab
 ababb aabba abbba aaaaa babab abbaa ababa baa
EACMGC364V           {0} ; 911
 aaaba bbabb baaba aaaab bbaab abbab aaabb aabba bbbbb abaaa
 abaaa aabbb babab bbaaa babbb ababb bbbab baaab aabaa baabb
 abbba abbaa aabab bbaaa bbbba abbab bbbba abaab bbbbb babab
 ababb aabba ababb aaaab bbbab aabaa baaaa aaa
ECGMGG495G           {0} ; 1108
 aaaaa bbaba babab aaaab bbaab abbab abaab aabaa bbbbb abaab
 abaab aabab bbaab bbaab babab aaabb babab babaa babbb baa-a
 bbaba abbaa aabab abbba bbbbb abbab bbbba aaaaa aaaaa bbaba
 babba aabab abaab bbbaa ababa babba abbaa bbb
EAGTCA62V            {0} ; 1284
 aaaaa bbaba babab aaaab bbaab abbab baaab aabba abbbb abaaa
 abaab aabab bbbab bbbab babab aaabb bbbab babaa aabaa baaba
 bbabb abbaa aabab ababa bbbb- abbab bbbba aaaaa aabaa bbaba
 babba abaab abaab bbbab ababa babba abbaa bab
EAGTCA187G           {0} ; 1297
 aaaaa bbaba babaa aaaab bbaab abbab baaab aabaa bbabb abaab
 abaab aabab bbbab bbaab babab aaabb babab babaa babab baaba
 baabb abbaa aabab abbba bbbb- abbab babba aaaaa aaaaa bbaba
 babba abbab abaab bbbab ababa babba abbaa bbb
EAGTCA190V           {0} ; 1298
 aaaba bbaba babaa aaaab bbaab abbaa baaab aaaaa bbabb abaab
 abaab aabab bbbab bbaab babab aaabb babab babaa babab baaba
 bbabb abbaa aabab abbba bbbb- abbab babba aaaaa aaaaa bbaba
 babba abbab abaab bbbab ababa babba abbaa bbb
EAGTCA199V           {0} ; 1299
 aaaaa bbaba babbb aaaab bbaab abbab baaab aabaa bbbbb abaab
 abaab aabab bbbab bbaab babab aaabb babab babaa babab baaba
 bbabb abbaa aabab abbba bbbb- abbab bbbba aaaaa aaaaa bbaba
 babba abbab abaab bbbab ababa babba abbaa bbb
EAGTCA408bG          {0} ; 1314
```

```
 aaaaa bbaba babbb aaaab bbaab abbbb baaab abbaa bbbbb abaab
 abaab aabab bbbab bbaab babab aabbb babab babaa bbbab baaba
 baabb abbaa aabab abbba bbbb- abbab bbbba aaaaa aaaaa bbaba
 babba abbab abaab bbbab ababa babba abbaa bbb
EACTGA329G          {0} ; 1376
 aabaa bbaba aabab aabab bbaab abbab baaab aabaa bbbba bbbab
 abaa- aabab bbaab bb-ab babab a-bbb babab babaa babab bbbba
 bbabb abbaa aabab abbba bbabb abbab abbba aaaaa aaaaa bbaba
 babba aabab abaab bbbab ababa babba abbaa bbb
EACTGA344V          {0} ; 1377
 aaaaa bbaba babab aaaab bbaab abbab baabb aabba abbbb abaaa
 abaa- aabab bbbab bb-ab babab a-abb babab babaa aabaa baaba
 bbbbb abbaa aabab ababa bbbbb abbab bbbba aaaaa aabaa bbaba
 babba abaab abaab bbbab ababa babba abbaa bab
EACTGA359G          {0} ; 1378
 aaaaa bbaba babab aaaab bbaab abbab baaab aabba abbbb abaaa
 abaa- aabab bbbab bb-ab babab a-abb babab babaa aabaa baaba
 bbabb abbaa aabab ababa bbbbb abbab bbbba aabba aabaa bbaaa
 babba abaab abaab bbbbb abaaa aaaba aabaa bab
ECGTCA413V          {0} ; 1435
 aaaab baabb aabab bbbab aabbb aabab ababb abaaa babba abaab
 aaaa- bbbbb abaaa ab-aa baaaa a-baa baaba aabab baaab babba
 bbbbb ababa aabba aabaa bbbbb abbbb baaba abaaa aaaab ababb
 abbab aaaba bbabb baabb bbaaa aaabb aaabb bba
ECGTCA416G          {0} ; 1436
 aaaab baabb aabab bbbab aabbb aabab ababb abaaa babba abaab
 aaaa- bbbbb abaaa ab-aa baaaa a-baa baaba aabab baaab babba
 bbabb ababa aabba aabaa bbbbb abbbb baaba abaaa aaaab ababb
 abbab aaaba bbabb baabb bbaaa aaabb aaabb bba
EACTTC210V          {0} ; 1453
 aabaa bb-ba aabab -abab baaab abbab baa-- babaa babba bbbab
 aaaab aaabb bbaab bbaaa ba--b ---bb babbb aabaa babab babba
 bbbbb abaaa aabaa abbba bbabb abbaa abbba aaaaa aaaaa ababa
 babba aabab abaab bbbab a-aba babba abbaa bbb
EACTTC221G          {0} ; 1455
 aabaa bb-ba aabab -abab baaab abbab baaa- aabaa babba bbbab
 aaaab aaabb bbaab bbaaa ba--b ---bb babbb aabaa babab babba
 bbabb abaaa aabaa abbba bbabb abbaa abbba abaaa aaaaa ababa
 babba aabab abaab bbbab a-aba babba abbaa bbb
EAGTAA135V          {0} ; 1501
 aaaaa bb-ba babab -aaab bbaa- abbab baaa- aab-a ab-bb a-aaa
 -baab aabab b-bab bbbab ba--a ----b bab-b -a--- ----a baaba
 bbabb abbaa aabab ababa bbbbb abbab bbbba aaaaa aabaa bbaba
 babba abaab abaab b--a- b-aba bbbba abbaa bab
EAGTAA136G          {0} ; 1502
 aaaaa bb-ba babab -aaab bbaa- abbab baaa- aab-a ab-bb a-aaa
 -baab aabab b-bab bbbab ba--a ----b bab-b -a--- ----a baaaa
 bbabb abbaa aabab ababa bbbbb abbab bbbba aaaaa aabaa bbaba
 babba abaab abaab b--a- b-aba bbbba abbaa bab
ECGTGA168V          {0} ; 1537
 aaaaa bb-ba aabab -bbab babab abbab aaaa- aabaa babba bbbab
 aaaab bbbab abaab bbaaa ba--a ---bb babbb aabab baaab babba
 bbbbb ababa aabba abbba bbabb abbab abbba aaaaa aaaaa ababb
 babab aabbb ababb babbb a-aba babbb abbaa bbb
SSR_229             {0} ; 1582
 aaaaa bbaba babaa aaaab baaaa bbbab aaaab aabba abbbb abaaa
 abaab aabab bbbab bbaab babab aaaba aabab babaa aabaa baabb
```

```
 ababb bbaaa aabab bbaaa babba abbab babba aaaaa aabaa bbbba
 babba abaaa abaa- bbbab ababa aabba abbaa bab

LG14:
EGAMGA312V          {0} ; 33
 aabaa baaab bbaab ababb aaaba baaba bbbaa aabbb babab abbba
 baaaa abaab aabbb baaba bbbbb babbb bbbaa abbbb bbbb- -----
 ----- ----- ----- ----- ----- ----- ----- ----- ----- -----
 ----- ----- ----- ----- ----- ----- ----- ---
ECAMCG254V          {0} ; 73
 babba baaab babbb bbabb bbaab bbbba babbb baabb bbbbb aabab
 ababa aaaab aaabb aabbb babab baabb abaaa bbbbb bbbb- -----
 ----- ----- ----- ----- ----- ----- ----- ----- ----- -----
 ----- ----- ----- ----- ----- ----- ----- ---
ECGMCG88G           {0} ; 90
 abaaa abaaa bbaaa abaab aabba bbbba aabab abbba babab bbaaa
 babaa abbaa bbabb babbb baaba baabb baaab babab abba- -----
 ----- ----- ----- ----- ----- ----- ----- ----- ----- -----
 ----- ----- ----- ----- ----- ----- ----- ---
EGAMGC549G          {0} ; 136
 babbb baaaa bbbab bbaba bbaab babaa bbbbb baabb bbbab aabab
 bbabb baabb abbbb aabbb babba baaab baaba bbaba bbaaa bbabb
 bbbba bbbab aabaa bbbba bbbaa abbba aaaba bbaab bbabb baaab
 babaa ababb bbbbb abbba babab bbbbb ababa baa
ECAMCC462V          {0} ; 173
 baaba baaab aaaab abaab bbaab bbbbb aabbb aaaba bbabb abbbb
 abaaa abaab aabbb bbabb babab baabb aaaaa bbbbb -bbba baabb
 babbb abbab aaaba abaaa bbbab abaab aaabb bbaaa bbabb babaa
 bbaaa bbaba baaaa aabbb bbbba babbb abbba aaa
ECAMGG120G          {0} ; 249
 aaaaa abaaa bbaaa aaaab aabba bbbba aabab abbba babab bbaaa
 babaa abbaa bbbab bbabb baaba baabb baaab babab abbab bbaba
 abaaa babab bbbaa abaaa babbb ababa aabab bbbbb bbaab abbba
 baabb bbaab bbbba aabaa bbaaa bbaab bbbaa aba
ECGMTA118G          {0} ; 304
 abbba aabab bbaba aaaab abbba bbabb bbbab abbba baaaa aabaa
 aaaaa baabb abaab aaaba bbaba aaabb abaab aaaab abaaa bbbbb
 baaaa aaabb aaaaa abbaa baabb babab aaaab bbbba abbbb bbbba
 abaaa bbbab bbabb abbba bbbaa bbaaa babba aab
EACMGA41G           {0} ; 336
 babaa aaaab aabab bbaab bbaab bbbba babbb baabb bbabb aabba
 ababa aaaab aaabb aabbb babbb baaab aaaaa bbbbb bbbba bbabb
 bbbb- abaab ababa bbaaa bbbab abaab aaabb bbbab bbabb babaa
 bbbaa bbabb baaab babba bbbbb bbbbb abbba aba
EACMGA402G          {0} ; 374
 babbb baaaa bbbab bbaba bbaab babaa bbbbb baabb bbbab aabab
 bbabb baabb abbbb aabbb babba baaab baaba bbaba bbaaa bbabb
 bbbb- bbaab aabaa bbbba bbbaa abbba aaaba bbaab bbabb baaab
 babaa ababb bbbbb abbba babab bbbbb ababa baa
EACMTC314V          {0} ; 495
 babaa baaab aaaab abaab abaab bbbbb aabbb aaaba baabb abbbb
 bbaaa abaab aabba bbabb baba- baabb aaaab bbbbb abaaa baabb
 babbb abaab aaaba ababa bbbab abaab ababb bbaaa bbaab baaaa
 bbaaa bbabb baaaa aabbb bbbba babba ababb aba
EACMTC444V          {0} ; 522
 aabaa baabb bbaaa ababb aaaba bbbba bbbaa abbbb babab abbba
 babaa abaab aabbb babba bbbb- babbb babab abbbb abbaa aaabb
```

aabbb aabab aaaba ababa babbb abaab abbbb abbbb abaab aaaba
bbabb bbaab bbabb aabbb bbbbb bbbbb abbab aaa
ECAMCT203V {0} ; 562
babbb baaaa babab bbaba bbbab babaa bbbbb bbabb babab aabab
bbabb baabb bbbbb aabbb baab- baaab bbabb baaba bbaaa bbabb
abbba bbbbb abbba bbbbb bbbbb abbba aaaba bbaab bbabb bbaab
babab ababb bbbbb abbbb babbb abbbb ababa baa
ECGMTT385G {0} ; 623
aabaa baabb aba-a ababb aaaba bbaba bbbaa abbbb babab abbba
babaa abaab aabbb babba bbb-- babbb babab abbbb abbaa aabbb
aabbb aabab aabba ababa babbb abaab ababb abbbb abaab aaaba
bbabb bbaab bbabb aaabb bbbbb bbbbb abbab aaa
ECGMGC597V {0} ; 730
babab baaaa bbbab bbaaa bbaab babab bbbbb aaabb babaa aabab
aaabb baaba aabab aaaba babaa baaab baaba ababa abaaa bbabb
bbbaa bbbaa aabaa bbbba abbaa abbaa aaaba abaab bbabb baaab
babaa ababb bbbbb ababb aabab bbbab ababa baa
EACMGG409V {0} ; 785
babaa baaab aabab bbaab bbaab bbbba babbb baabb bbabb a-bab
ababa aaaab aaabb aabbb babab baabb aaaaa bbbbb bbbba bbabb
bbbbb abbab ababa bbaaa babab abaab a-abb bbbab bbabb babaa
bbbaa bbabb baaab babbb bbbbb bbbbb abbba aba
EACMGG559G {0} ; 797
babaa baaab bbaab ababb aaaab bbabb bbbaa aabba babab a-bbb
baaaa abaab aabba baaba bbbbb babbb babab bbbbb abbaa baabb
babbb abaab aaaba ababa bbbbb abaab a-abb bbaaa bbaaa baaab
bbaab bbaab bbaab aabab bbbbb bbbbb abbab aba
EAGMCT163G {0} ; 810
abbaa ababa bbaaa ababb aabba bbaba aabaa abbbb babab a-aaa
babaa abbaa abbbb babba baabb bbbbb babab babbb abbaa baaab
aaaba babab abbab ababa aabbb ababa a-bbb bbbbb abaab ababa
bbaab bbaab bbbba aabab bbaaa bbbbb abbaa aaa
EACMGC249V {0} ; 888
aabaa baaab bbaab ababb aaaba bbabb bbbaa abbbb babab abbbb
babaa abaab aabbb baaba bbbbb babbb babab abbbb abbaa aaabb
babbb aabab aaaba ababa babbb abaab ababb abbaa baaaa bbbbb
bbbbb babba baaab baabb aabbb babbb baabb baa
EACMGC250G {0} ; 889
aabaa baaab bbaab ababb aaaba bbabb bbbaa abbbb babab abbbb
babaa abaab aabbb baaba bbbbb babbb babab abbbb abbaa aaabb
babbb aabab aaaba ababa babbb abaab ababb abbaa baaaa bbbbb
bbbbb babba baaab baaba aabbb babbb baabb baa
EACMGC271V {0} ; 893
abbaa ababa bbaaa abaab aabaa baaba aabaa abbba babab baaaa
babaa abbaa ababb babba baabb bbbbb babab babbb abbaa baabb
aaaba babab bbbab ababa aabbb ababa aabbb bbbbb aaaba bbbaa
abbaa bbbbb aaaaa baabb babab aaabb baabb bbb
EACMGC456V {0} ; 919
babbb baaaa bbabb bbaba bbbab babaa bbbbb baabb babab aabab
bbabb baabb bbbbb aabbb baaba baaab bbabb baaba bbaaa bbaab
abbba baaaa aaaaa babba bbbaa abbaa aaaaa bbaaa babbb ababb
babba aabab abaaa babbb aaaab ababa babbb baa
EAGMCA228V {0} ; 951
abbba aaaab bbaaa aaaab abbba bbabb bbbbb aabba baaaa bbbaa
aaaaa baabb baaaa baaab baaba aaaab bbaab aaaab abaaa bbbba
baaaa aabbb abbab abbaa baabb babaa aaaab bbaba aaabb bbbaa
abbaa ababa aaaba abbbb bbbbb baaab bbabb bbb

```
ECGMCA271G            {0} ; 990
 babaa baaab aaaab abaab bbaab bbbbb aabbb aaabb bbabb abbbb
 ababa abaab aaabb babbb babab baabb aaaaa bbbbb abbba baabb
 bbbbb abbab aaaba abaaa bbbab abaab aaabb bbaab baabb bbbbb
 ababb babbb aabab baaba abaab baaab babbb aaa
EAGMTT579G            {0} ; 1087
 babba baaab aabab bbabb bbaab bbbba babbb baabb bbbbb aabab
 ababa aaaab aaabb aabbb babab baabb aaaaa bbbbb bbbba babbb
 bbbbb abbab bbaba bbaaa bbbaa ababb aaabb bbbab bbabb babaa
 babaa ababb babab bbbba bbbbb bbbbb abbba aba
EACTAG64V             {0} ; 1222
 babab baaab aabbb abaab bbaab bbbba aabbb aaabb bbabb abbbb
 abbaa aaaab aaabb babbb babab baabb aaaaa bbbbb abbba baabb
 bbbbb abbab aaaba abaaa bbba- abaab aaabb bbaab bbaab babaa
 bbaaa bbabb baaab aabbb bbbba babbb abbba aba
EACTAG360G            {0} ; 1268
 abbaa abaab bbaab aaaab abbba bbabb bbbbb aabba baaaa bbbba
 aaaaa baabb baaaa baaab baaba aaaba bbaab aaaab abaab bbbba
 baaaa aabbb abbab abbaa bbab- baaba aaaab baaba aabab bbbbb
 bbaaa bbbab bbbba aabbb bbaaa bbaab bbbaa abb
EACTAG364V            {0} ; 1269
 abbaa abaab bbaab aaaab abbba bbabb bbbbb aabba baaaa bbbba
 aaaaa baabb baaaa baaab baaba aaaba bbaab aaaab abaab bbbba
 bbaab aabbb abbab abbaa bbab- baaba aaaab baaba aabab bbbbb
 bbaaa bbbab bbbba aabbb bbaaa bbaab bbbaa abb
EAGTCA408G            {0} ; 1313
 abaab abaaa bbbba abaab aabba bbbba aabbb abbba babab bbbba
 baaba abbab baabb babbb baaba babbb baaab baaab aabab bbbba
 aaaaa babab bbbaa abbaa aaab- ababa abbab bbbba aaaab abbbb
 baabb bbaab bbbba aabab bbaaa bbaab bbbaa aba
EACTGA93V             {0} ; 1348
 abbbb aabab bbaba aaaab abbba bbabb bbbab abbba baaaa bbbaa
 aaaa- baabb baaaa ba-ab bbaba a-abb abaab aaaab abaaa bbbbb
 babaa aaabb abaaa abbaa baabb babab aaaab bbbba abbbb bbbbb
 abaaa bbbab bbabb abbbb bbbaa bbaba babba aab
ECGTCA168G            {0} ; 1424
 babaa baaab aaaab abaab bbaab bbbbb aabbb aaaba bbabb abbbb
 abaa- abaab aabbb bb-bb babab b-abb aaaaa bbbbb abbba baabb
 baabb abaab aaaba abaaa bbbab abaab aaabb bbbaa bbaab babaa
 bbaaa bbabb baaaa aabbb bbbba babbb ababa bba
ECGTCA170V            {0} ; 1425
 babaa baaab aaaab abaab bbaab bbbbb aabbb aaaba bbabb abbbb
 abaa- abaab aabbb bb-bb babab b-abb aaaaa bbbbb abbba baaba
 babbb aaaab aaaba abaaa bbbab aaaab aaabb bbaaa bbaab babaa
 bbaaa ababb baaaa aabbb abbba babbb aaaba aaa
ECGTCA362G            {0} ; 1433
 abaab abaaa bbbaa abaab aabba bbbba aabab abbba babaa bbbba
 baaa- bbbab baabb ba-ab baaba b-abb baaab baaab abbab bbbba
 abaaa babab bbbaa abbaa ababb abbba aabab bbbba aaaab abbbb
 baabb bbaab bbbba aabab bbaaa bbaab bbbba bbb
EACTTC231V            {0} ; 1457
 babba ba-ab aabab -babb bbaab babba bbbb- baabb bbbbb aabab
 abbaa baaab aaabb aabbb ba--a ---bb aaaba bbabb bbbba bbabb
 bbbbb abbab ababa bbaaa bbbaa ababb aaabb babab bbabb babaa
 babaa ababb babab bbbbb b-bbb bbbab abbba aba
EACTTC232G            {0} ; 1458
 babba ba-ab aabab -babb bbaab babba bbbb- baabb bbbbb aabab
```

```
 abbaa baaab aaabb aabbb ba--a ---bb aaaba bbabb bbbba bbabb
 bbabb abbab ababa bbaaa bbbaa ababb aaabb babab bbabb babaa
 babaa ababb babab bbbbb b-bbb bbbab abbba aba
EACTTC407V          {0} ; 1473
 babba ba-ab aabab -babb bbaab bbbaa babb- baabb bbbbb aabab
 abbaa aaaab aaabb aabbb ba--b ---bb aaaaa bbbbb bbbba bbabb
 bbbbb abbab ababa bbaaa bbbaa ababb aaabb bbbab bbabb babaa
 babaa ababb babab bbbbb b-bbb bbbab abbba aba
ECGTGA266V          {0} ; 1548
 abbaa ab-ba bbaaa -baba aabaa baaba aaba- abbba babab bbaaa
 baaba abbaa abbbb babba ba--b ---bb babab babbb abbaa baaaa
 aabba babab bbbab ababa aabbb ababa ababb babbb abaab ababa
 baaab bbaab bbbba aaabb b-aaa bbaab bbbaa aaa
ECGTGA283G          {0} ; 1549
 abbaa ab-ba bbaaa -baba aabaa baaba aaba- abbba babab bbaaa
 baaba abbaa abbbb babba ba--b ---bb babab babbb abbaa baaaa
 aaaba babab bbbab ababa aabbb ababa aabbb bbbbb abaab ababa
 baaab bbaab bbbba aaabb b-aaa bbaab bbbaa aaa
ECGTGA361V          {0} ; 1557
 babbb ba-aa babab -babb bbaab bab-a bbbb- baabb bbbab aabab
 abbab baabb abbbb aabbb ba--a ---bb aaaba bbabb bbbaa bbabb
 bbbbb abbab aabba bbaaa bbbaa abbbb aaaba bbaab bbabb baaaa
 babaa ababb babab bbbbb b-bab bbbab abbba bba
ECGTGA362G          {0} ; 1558
 babbb ba-aa babab -babb bbaab bab-a bbbb- baabb bbbab aabab
 abbab baabb abbbb aabbb ba--a ---bb aaaba bbabb bbbaa bbabb
 bbabb abbab aabba bbaaa bbbaa abbbb aaaba bbaab bbabb baaaa
 babaa ababb babab bbbbb b-bab bbbab abbba bba
ECGTGA501V          {0} ; 1573
 abbaa ab-ba bbaaa -baba aabaa baaba aaba- abbba babab bbaaa
 baaba abbaa abbbb babba ba--b ---bb babab babbb abbaa baaaa
 aabba babab bbbab ababa aabbb ababa aabbb bbbbb abaab ababa
 baaab bbaab bbbba aaabb b-aaa bbaab bbbaa aaa
SSR_86              {1} ; 1579
 ab-ba babbb ---aa babaa aaaa- --aaa bbaaa aaaab bbaab bbaaa
 aabbb babbb bbaaa aabaa ababa abbaa babbb bbbbb bbaab abbaa
 bbaab babba bbaab babbb aaaba babaa bbbaa aabbb aabba ababb
 aabbb aabaa abbb- bbaaa bbbab abaaa baaab bab
SSR_154             {0} ; 1580
 abbbb aabab bbabb bbbab abbab ababb bbbab abbba baaaa bbb--
 -aaaa baabb bbaaa baaab baaba aaabb abaab aaaab ababa bbbaa
 babba aaabb abbab abbaa baabb babaa aaaab bbbba abbaa aaaab
 abaaa bbbab bbaa- abbbb bbaaa aaaba babaa aab

LG15:
EGAMGA94V           {0} ; 8
 baaaa baaba aabba aabbb babaa baaba bbaaa aaaab abaab bbbab
 bbabb bbabb babab ababb babab aabbb bbabb bbbab abbb- -----
 ----- ----- ----- ----- ----- ----- ----- ----- ----- -----
 ----- ----- ----- ----- ----- ----- ----- ---
EGAMGA113G          {0} ; 12
 babaa baaba babba aabbb baaaa baaaa bbbaa babbb aaaba babaa
 bbabb bbabb bbaab ababb aabbb abbbb babbb abbab abab- -----
 ----- ----- ----- ----- ----- ----- ----- ----- ----- -----
 ----- ----- ----- ----- ----- ----- ----- ---
ECAMCG68G           {0} ; 53
 baaaa baaba aabaa aabbb bbbaa baaba bbaaa aaaab aaaab babab
```

```
 bbabb bbabb bbaab ababb aabab aabbb baabb abbab abbb- -----
 ----- ----- ----- ----- ----- ----- ----- ----- ----- -----
 ----- ----- ----- ----- ----- ----- ----- ---
EGAMGC147G           {0} ; 113
 aaaba babab babaa abbba bbbab baaba bbaaa bbaab bbaab aaaab
 bbabb bbabb babbb baaba bbbab aaabb bbbab bbaaa abbbb bbaaa
 babab bbaab aaaaa bbbbb abaaa bbbba baaba aabbb abbbb baaab
 bbbba abaaa bbabb babaa bbaaa baaaa ababb baa
EGAMGC571G           {0} ; 137
 baaaa baaba aabaa aabbb bbbaa baaba bbaaa aaaab aaaab bbbab
 bbabb bbabb baaab ababb aabab aabbb bbabb abbab abbba ababa
 aabab abaab aabba aabbb aabaa babbb baaba aabba bbaab bbaaa
 babaa ababb bbabb aaaaa bbaaa baaaa aaabb bbb
EGAMGC573V           {0} ; 138
 baaaa baaba aabaa aabbb bbbaa baaba bbaaa aaaab aaaab bbbab
 bbabb bbabb baaab ababb aabab aabbb bbabb abbab abbba ababa
 aabab aaaab aabba aabbb aabaa babbb baaba aabba bbaab bbaaa
 babaa ababb bbabb aaaab bbaaa baaaa aaabb bbb
ECAMCC111G           {0} ; 141
 babbb bbaba baaba babaa baaaa bbaab babba aaaaa baaba babaa
 bbabb baabb babab baaab ababa babbb aabbb abbba -baba aabbb
 babaa abaab abbba aaaab bbbbb bbbab babaa baaba babba abbaa
 bbbaa bbabb babbb aabaa bbabb aaaaa baaaa bbb
ECAMCC159V           {0} ; 146
 babbb bbabb baabb babaa baaaa bbaaa aabba aaaaa baaba babaa
 ababb aaaab baaab baaab bbaba bbabb aabbb abbba -bbba abbab
 bbbaa abaaa abbbb abaab bbabb abbab babaa aaaba baaba abbab
 abaab bbabb babbb abbbb bbaab aabba baaaa baa
ECAMCC228V           {0} ; 153
 baaaa aaaba babaa aabbb bbaaa baaba bbbaa aabab aaaaa babab
 bbabb bbabb bbaab ababb aabab abbbb bbbbb abbab -bbba aabba
 aaaaa aabab aabba aabbb aabab babbb ababa aabba bbaab baaaa
 babaa ababb baaba aaabb bbaba baaaa aaaba bbb
ECAMCC229G           {0} ; 154
 baaaa baaba babaa aabbb bbaaa baaba bbbaa aabab aaaaa babab
 bbabb bbabb bbaab ababb aabab abbbb bbbbb abbab -bbba aabba
 aaaaa aabab aabba aabbb aabab babbb ababa aabba bbaab baaaa
 babaa ababb baaba aaaba bbaba baaaa aaaba bbb
ECGMCC576V           {0} ; 193
 baaaa baaba babaa aabbb bbaaa baaba bbbba aabab aaaaa babab
 bbabb bbabb abaab ababb aabab abbbb bbbbb aabab abbba aabba
 aaaaa aab-b aabba babbb aabab babbb ababa aabaa baaab baaaa
 babaa ababb baaba aaabb bbaba baaaa aaaba bbb
EACMCC495V           {0} ; 235
 aabab babab babab ababa bbbab baaaa abaab bbaab bbaab aaaab
 bbabb bbabb abbbb baaab bbbab aaabb bbbab baaab abbbb bbaaa
 babab bbaab aaaaa abbbb abaaa bbbba baaba abbbb abbab aabaa
 bbbba abaab ababb babab bbaaa baaaa abaab baa
EACMGA93G            {0} ; 337
 babaa bbaba baaab babaa baaaa baaab aaaba aaaaa baaaa babaa
 bbabb baabb baaab baaab bbaba baabb aabbb abbba aaaba aabab
 baba- abaab abbba baaab bbabb bbbab babaa baaaa babba abbab
 bbbab bbabb babbb abaaa bbabb aaaaa baaaa bbb
EAGMTA364G           {0} ; 419
 aabaa babab babaa abbba bbbab baaba bbaaa bbaab bbaab aaaab
 bbabb bbabb babbb b-aba bbbab aaabb bbbab bbaaa abbbb bbaaa
 baba- bbaab aaaaa bbbbb abaaa bbbba baaba aabbb abbab baaab
```

```
 bbbba abaaa bbabb babab bbaaa baaaa ababb baa
ECGMGA378G           {0} ; 440
 babbb bbabb baabb babaa baaaa bbaaa aabba aaaaa baaba baaaa
 bbabb aaaab baaab b-abb bbaba baabb aabbb abbba ababa aabab
 bbba- bbaaa abbbb abaab bbabb aabab baaaa baaba baaba abbab
 abaab bbabb babbb aabab baaab aabba baaaa baa
EACMTC144V           {0} ; 461
 babbb bbaba baabb babaa baaaa bbaab aabba aaaaa baaba babaa
 bbabb baabb baaab baaab bbab- baabb aabbb abbba ababb aabab
 babaa abaaa abbba aaaab bbabb bbbab babaa baaba babba abbab
 abaab bbabb babbb ababb bbabb aaaaa baaaa bab
EACMTC333G           {0} ; 498
 babba bbabb babaa aabba abaaa baaaa bbbba aabab aabaa aaaaa
 ababb bbabb abbbb aaaab bbab- abbbb baaab abbaa baaba aabbb
 aaaaa aabab aabaa babba aabaa babbb abbba aabbb abaaa bbbaa
 babaa abaaa baabb aabaa ababa baaaa aaaaa aab
EACMGG124G           {0} ; 745
 baaaa baaba aabaa aabbb bbbaa baaba bbaaa aaaab aaaab b-bab
 bbabb bbabb baaab ababb babab aabbb bbabb bbbab abbba ababa
 aabab aaaab aabaa aabbb abbaa babbb b-aba aabba bbabb bbaaa
 babbb aaaba bbabb aaaab bbaaa baaaa aaabb bbb
EACMGG215G           {0} ; 761
 babbb bbabb baabb babaa baaaa bbaaa aabba aaaaa baaba b-baa
 bbabb aaaab baabb baaab bbaba baabb aabbb abbba ababa aabab
 babaa abaab abbbb abaab bbabb bbbab b-bbb bbbbb bbaba abbbb
 abbbb bbbbb babbb abbab baaab aabba baaaa bab
EACMGG542V           {0} ; 795
 babba baaaa baabb babaa babab bbbaa aabba abaab aaaba b-baa
 aaaab aaaab baaab aaaab bbaba bbbbb abbbb abbba aabba abbab
 bbaaa abaab abbbb bbaab aaaab abbab b-baa aaaba baaaa abbba
 abaab aaabb baaba abbbb bbbab aaaab baaaa aaa
EAGMCT71G            {0} ; 801
 baaaa bbaba babaa aabbb bbaaa baaba bbbaa aabab aaaaa b-bab
 bbabb bbabb bbaab ababb aabab abbbb bbbbb abbab abbba aabba
 aaaaa aabab aabba aabbb aabab babbb a-aba aabba bbabb baaaa
 babaa ababa baaba aaaaa bbaba baaaa aaaba bbb
EAGMCT278G           {0} ; 826
 aabaa baaab babba bbbba bbbab baaba bbaaa bbaab bbaab a-aab
 bbabb bbabb babbb baaba bbbab aaabb bbaab bbaaa abbbb bbaaa
 babab bbaab aaaaa bbbbb abaaa bbbba b-aba aaabb abbab baaab
 bbbaa ababb bbabb babaa bbaaa baaaa ababa aaa
EAGMCT278V           {0} ; 827
 aabaa baaab babba babba bbbab baaba bbaaa bbaab bbaab a-aab
 bbabb bbabb babbb baaba bbbab aaabb bbaab bbaaa abbbb bbaaa
 babab bbaab aaaaa bbbbb abaaa bbbba b-aba aaabb abbab baaab
 bbbaa ababb bbabb babba bbaaa baaaa ababa aaa
ECGMGT119G           {0} ; 849
 baabb bbaba baaaa babaa baaaa bbaaa abbaa aaaaa baaba b-baa
 bbabb baabb baaab baaab bbaba baabb aabbb abbba abaaa aabab
 babaa abaab abbba aaaab bbaab bbbab b-baa baaba babba abbaa
 bbbaa bbabb babbb abaab bbabb aaaaa baaaa bbb
EAGMCA137V           {0} ; 941
 aabaa babab baaba abbba bbbab baaba bbaaa bbaab bbaab aaaab
 bbabb bbabb babbb baaba bbbab aaabb bbbab bbaaa abbbb bbaaa
 babab bbaab aaaaa bbbbb abaaa bbbba baaba aabbb bbaba bbbaa
 abaaa aabab bbaaa bbabb aaabb bbbaa baabb bab
EAGMCA196G           {0} ; 946
```

```
 babbb bbaba babab babaa baaaa bbaab aabba aaaaa baaba babaa
 bbabb baabb baaab baaab bbaba baabb aabbb abbba ababa aabab
 babaa abaab abbba aaaab bbabb bbbab babaa baaba babaa bbbab
 baaaa abaaa abbbb abbaa bbabb bbabb babbb abb
EAGMCA282V          {0} ; 959
 aaaab babab baabb aaabb bbbab baaaa abaab bbaab bbaab aaaab
 bbaaa bbabb bbbbb baaba bbbab ababb bbbab babaa abbbb baaaa
 babab bbaab aaaaa aabbb abaaa bbbba aaaba abbbb bbabb bbbab
 abaaa aabaa aaaaa bbabb abaab aabaa baaba bab
EAGMTT144V          {0} ; 1052
 aabab babab babab aaaba bbbab baaaa baaab bbaab bbaab aaaab
 bbaba bbabb bbbbb baaba bbbab aaabb bbbab baaaa abbbb babaa
 babab bbaab aaaaa abbbb abaaa bbbba baaba abbbb abbab aabaa
 bbbba abaab ababb babab bbaaa baaaa abaab baa
EAGMTT571V          {0} ; 1086
 aabaa baaaa aabaa abbbb bbbab baaaa bbaaa baaab bbaab abaab
 bbabb bbabb babab baaba babab aaabb bbaab bbaab abbbb babba
 babab bbaab aaaaa aabbb abaaa bbbba baaba aabbb abbbb bbaab
 bbbba ababb bbabb baaab bbaaa baaaa ababb bab
ECGMGG294G          {0} ; 1099
 aaaaa babab babab aaabb bbbab baaaa abaab bbaab bbaab aaaab
 bbaaa bbabb bbbbb baaba bbbab bbabb bbbab babaa abbbb bba-a
 babab bbaab aaaaa aabbb abaaa bbbba aaaba abbbb abbab aabaa
 baabb abaab ababb babba bbaba baaaa abaab baa
ECGMGG312V          {0} ; 1100
 babbb baaba babaa aabbb bbaaa baaaa bbbaa aabab aaaaa babaa
 bbabb bbabb bbaab ababb aabbb abbbb babbb abbab ababa aab-b
 baaaa aabab aabba babbb aabab babbb ababa aabbb bbabb babaa
 babaa bbabb baaba aaabb bbaba baaaa baaba bbb
EACTCC136V          {0} ; 1126
 babab baaba aabbb aabbb bbaaa babab bbbba babab aaaaa babab
 bbbab bbabb bbaab ababb aabab abbbb bbbbb abbab bbbba aabba
 aabaa aabab aabba aabbb aabab babbb ababa aabba bbabb baaaa
 babaa ababa baaba aaaba bbaba baaaa aaaba bbb
EACTAG139G          {0} ; 1236
 baaaa baaba babaa aabbb bbaaa baaba bbbaa aabab aaaaa babab
 bbbab bbabb bbaab ababb aabab abbbb bbbbb abbab abbba aabba
 aaaaa aabab aabba babbb aaba- babbb abbba aabba bbabb baaaa
 babaa ababa baaba aaaba bbaba baaaa aaaba bbb
EACTAG336G          {0} ; 1263
 babbb bbabb baaab babaa baaaa bbaaa aabba aaaaa baaba babaa
 bbbab aaaab baaab baaab bbaba bbabb aabbb abbba ababb babab
 babaa abaaa abbbb abaab bbab- abbab babaa baaba baaba abbab
 abaab bbabb babba abbbb bbaab aabaa baaaa bbb
EACTGA121G          {0} ; 1353
 aabaa baaaa aabaa abbbb bbbab baaba bbaaa baaab bbaab abaab
 bbba- bbabb babab ba-ba babab a-abb bbaab bbaab abbbb bbaba
 baaab bbaab aaaaa aabbb abaaa bbbba baaba aabbb abbbb bbaab
 bbbba ababa bbabb baaaa bbaaa baaaa ababb bab
EACTGA123V          {0} ; 1354
 aabaa baaaa aabaa abbbb bbbab baaba bbaaa baaab bbaab abaab
 bbba- bbabb babab ba-ba babab a-abb bbaab bbaab abbbb bbaba
 babab bbaab aaaaa aabbb abaaa bbbba baaba aabbb abbbb bbaab
 bbbba ababb bbabb baaaa bbaaa baaaa ababb bab
EACTTC532G          {0} ; 1492
 babbb bb-ba babba -bbaa baaaa bbaab baba- aaaaa baaba babaa
 bbaab baaab bbaab aaabb ab--a ---bb babbb abbab ababa aabba
```

```
 baaaa abaab abbba baaab bbbbb bbbab bbaba babba bbbba bbbaa
 bbbaa bbabb baabb aabab b-abb baaaa baaba bbb
EACTTC533V          {0} ; 1493
 babbb bb-ba babba -bbaa baaaa bbaab baba- aaaaa baaba babaa
 bbaab baaab bbaab aaabb ab--a ---bb babbb abbab ababa aabba
 babaa abaab abbba baaab bbbbb bbbab bbaba babba bbbba bbbaa
 bbbaa bbabb baabb aabab b-abb baaaa baaba bbb
EAGTAA70V           {0} ; 1500
 babba ba-ba babba -bbbb bbaa- baaaa babb- aab-a ba-ba b-baa
 -bbab bbabb b-aab aaabb aa--b ----b bab-b -b--- ----a aabba
 babaa aabab aabba babbb abbab babbb bbaba babbb bbaba babaa
 babaa bbabb baabb a--a- b-abb bbaaa baaba bbb
EAGTAA231G          {0} ; 1513
 babaa ba-ba babaa -abbb bbaa- baaba bbba- aab-b aa-aa b-bab
 -bbab bbabb b-aab ababb aa--b ----b bbb-b -b--- ----a aabaa
 aaaaa aabab aabba babbb aabab babbb ababa abbba bbabb baaaa
 babaa ababa baaba a--b- b-aba bbaaa aaaba bbb
SSR_1101            {0} ; 1616
 --aaa ababa b--ab ababa ababb babab babba aaabb bbaaa bbbaa
 bbaaa baaaa babab baabb bbaba baaab aabbb abbab abaaa aabba
 baa-- --aab abbba bbaab abaaa bbbab babaa baaba babba abbaa
 abbaa bbaaa aabb- aabab ---bb aaaaa baaaa bbb

LG16:
EGAMGA259G          {0} ; 27
 aaabb abaaa aaaab babab baabb abbaa aaaab abaaa babaa ababb
 bbbbb ababa bbaba aabbb ababa baaab aaaba aaaab aaba- -----
 ----- ----- ----- ----- ----- ----- ----- ----- ----- -----
 ----- ----- ----- ----- ----- ----- ----- ---
EGAMGA259V          {0} ; 28
 aaabb abaaa aaaab babab baabb abbaa aaaab abaaa babaa ababb
 bbbbb ababa bbaba aabbb ababa baaab ababa aaaab aaba- -----
 ----- ----- ----- ----- ----- ----- ----- ----- ----- -----
 ----- ----- ----- ----- ----- ----- ----- ---
EGAMGA331G          {0} ; 34
 aabba abaab bbbaa bbaba bbbbb bbbaa aabab aabbb abaaa bbbab
 abbaa babaa bbbbb bbaab bbbaa bbbbb aaaaa bbabb abba- -----
 ----- ----- ----- ----- ----- ----- ----- ----- ----- -----
 ----- ----- ----- ----- ----- ----- ----- ---
ECAMCC240V          {0} ; 157
 aabab abaaa aaaab babbb babba abbaa baabb abbaa babaa ababb
 bbbbb ababa bbaba abbab ababa baaab ababa aaaab -abab aabab
 ababb aabab bbabb aabab babbb aabaa bbaaa bbbaa babba abbab
 aabab abbab abbaa bbaab aabab abbba bbaaa baa
ECGMGA430G          {0} ; 443
 aaaaa abaab abbba aabba aabbb bbbba aaabb aabaa baaaa abbab
 abbaa babaa bbbbb b-baa bbaab baabb ababa baabb aabab babab
 bbab- aaaab aaabb abbaa babbb aabba abbaa babaa babaa abaaa
 aaabb aabab bbaab abbab aabbb bbaab baabb bbb
EACMTC212V          {0} ; 479
 aabba abaab bbbba bbaba babbb bbbba aabab aabbb abaaa bbbab
 abbaa babaa bbbbb bbaab bbba- bbbbb aaaba bbabb abbab baaba
 bbaba aaabb babbb abbaa babba babba aabab aabaa bbbbb baaab
 aaabb bbbab aaaab abbbb aabbb baaab aabaa bbb
EACMTC241G          {0} ; 484
 aaaba abaab bbbba aaabb babbb bbbba aabbb aabba abaaa bbbab
 abbaa babaa bbbbb bbbab bbba- bbabb ababa bbabb aabab baabb
```

bbabb aaabb baabb abbaa babbb aabba abbab aabaa babaa baaaa
aaabb babab baaab abbab aabbb baaab aabba bbb
ECGMTT370G {0} ; 619
aaaba abaab abb-a aaaba babbb bbbba aabab aabba abaaa bbbab
abbaa babaa bbbbb bbbab bbb-- bbabb ababa bbaaa aabab babba
bbabb aaabb baabb abbab babba aabba abbab aabaa babaa baaab
aaabb babaa baaab abaab aabbb baaab aabba bbb
ECGMTT374V {0} ; 621
aaaba abaab abb-a aaaba babbb bbbba aabbb aabba abaaa bbbab
abbaa babaa bbbbb bbbab bbb-- bbabb ababa bbbbb aabab babba
bbabb aaabb baabb abbab babba aabba abbab aabaa babaa baaab
aaabb babab baaab abbab aabbb baaab aabba bbb
EACMCG254V {0} ; 646
aabaa baaab bbbab bbaba aabaa bbbaa aabbb aabbb ababa bbaaa
bbbaa babaa bbbab baaab baaaa ababb baaba bbaab bbbbb aabba
baaaa aaabb babab aaaaa babba bbaba aabab abbaa ababb bbabb
aaabb bbbab abbab aabab aabab baaab abbaa bba
EACMCG258G {0} ; 647
aabaa baaab bbbab bbaba aabaa bbbaa aabab aabbb ababa bbaaa
bbbaa babaa bbbab baaab baaaa ababb baaba bbaab bbbbb aabba
baaaa aaabb babab aaaaa babba bbaba aabab abbaa ababb bbabb
aaabb bbbab abbab aabaa aabab baaab abbaa bba
EAGMGG151V {0} ; 674
aabaa bbaab bbbab bbaba aabaa bbbaa aabab aabbb ababa bbaaa
bbbaa babaa bbbab baaab baaaa ababb baaba bbaab bbbbb aabba
baaaa aaabb babab abaaa babba bbaba aabab abbaa ababb baabb
aaabb bbbab abbab aabab aabab baaab abbaa bba
EAGMGG277bG {0} ; 692
aaabb abaaa aaaab bbbab bbaab abaaa abaab aaaab bbbaa ababb
abbbb abaaa bbaba babba abaaa baaab ababa aaaab aabab abbab
ababa bbbbb ababb aabab aabbb aabba ababa baaab babaa abbaa
aabaa abbab abbaa babaa aabaa abbba bbaab aba
ECGMGC175V {0} ; 711
babab abaab abbba aabbb abbba bbbaa aaabb abbaa baaab abbab
bbbaa aaaaa bbabb abbaa abaab bbabb ababa aaabb aabbb bbbab
bbabb aabab aabbb babaa babbb aabba abbaa babaa babba abaaa
aabbb aabab bbaab abbab aabbb bbaaa baabb bab
EACMGG170G {0} ; 753
babaa abaaa abbba aabba babba babaa baabb abbaa baaab a-bab
bbbaa aaaaa bbabb abbaa abaab baaab ababa aaabb aabab babab
bbabb aabab bbbbb babaa babbb aabaa b-baa babaa babba abaaa
aabbb abbab bbbaa abbab aabbb abaaa baabb baa
EAGMCT102V {0} ; 803
babaa abaaa abbba abbba babba babaa ababb abbaa baaab a-bab
bbbaa aaaaa bbabb abbaa abaab bbaab bbaba bbabb aabab babab
bbabb aabab bbbbb babaa babbb aabaa b-baa babaa babba abaaa
aabbb abbab bbbaa abbbb aabbb abaaa baabb baa
EACMGC413G {0} ; 915
aabba abaab bbbba bbaba babbb bbbba aabab aabbb abaaa bbbab
abbaa babaa bbbbb bbaab bbbaa bbbbb aaaba bbaba abbab baaba
bbaba aaabb babbb abbaa babba babba aabab aabaa babba baabb
bbaaa baaba abbbb bbbba aaaba aabbb bbaba aaa
EAGMCA255G {0} ; 957
aaabb abaaa aaaab babbb baabb abbaa aaaab aaaaa babaa ababb
bbbbb ababa bbaba abbab ababa baaab ababa aaaab aabab aabab
ababb babab bbabb aabab babbb aabaa abaaa bbbaa abaaa aaaba
babbb abbaa abaab abbaa bbaba ababa bbaaa aba

```
EACMGT65G            {0} ; 1003
 aaaba abaab bbbba aaabb babbb bbbba aabbb aabba abaaa abbab
 abbaa babaa bbbbb bbbab bbbab bbabb ababa bbabb aabab baabb
 bbabb aaabb baabb abbaa babbb aabba abbab aabaa babaa baaaa
 aaabb aabab bbaab abbaa aabbb baaab aaaba bbb
ECGMGG72G            {0} ; 1089
 baaaa abaaa abbba aabba babba babaa ababb bbbaa babab abbab
 bbbaa abaaa bbabb abbaa abaaa baaab ababa aaaba aabab bab-b
 bbaba aabab bbbbb babab babbb aabaa bbaaa babaa babba abaaa
 aabbb abbab bbbaa abbaa aabab abaaa bbabb baa
ECGMGG80V            {0} ; 1090
 aaaba abaaa aaaab bbbab bbaab abaaa baaab aaaab bbbaa ababb
 abbbb abaaa bbaba babba abaaa baaab ababa aaaab aabab abb-b
 ababb bbbbb bbabb aabab aabbb aabba ababa baaab babaa abbaa
 aabaa abbab abbaa babab aabaa abbba bbaab aba
ECGMGG550V           {0} ; 1110
 babab abaab abbba aabba aabba bbbaa aaabb abbaa baaab abbab
 bbbaa aaaaa bbabb abbaa abaab bbabb ababa aaabb aabab bab-b
 bbaba aabab aabbb babaa babbb aabba abbaa babaa babba abaaa
 aabbb aabab bbaab abbab aabbb bbaaa baabb bbb
ECGMGG550G           {0} ; 1111
 babab abaab abbba aabba aabba bbbaa aaabb abbaa baaab abbab
 bbbaa aaaaa bbabb abbaa abaab bbabb ababa aaabb aabab bab-b
 bbaba aabab aabbb babaa babbb aabba abbaa babaa babba abaaa
 aabbb aabab bbaab abbaa aabbb bbaaa baabb bbb
EACTAG88G            {0} ; 1227
 aaabb abaaa aaaab babab baabb abbaa aaaab abaaa babaa ababb
 bbbbb ababa bbaba aabbb ababa baaba ababa aaaab aabab aabab
 aaabb babab bbabb aabab babb- aabaa ababa bbbaa babba abbab
 aabab abbab abbaa baaaa aabab abbba bbaaa baa
EACTAG113V           {0} ; 1231
 aabaa baaab bbbbb bbaba aabaa bbbaa aabbb aabbb ababa bbaaa
 bbaba babaa bbbab baaab baaaa ababb baaba bbaab bbbbb aabba
 bbaaa aaabb babab aaaaa babb- bbaba aabab abbaa ababb bbabb
 aaabb bbbab abbab aabab aabab baabb abbaa bba
EACTAG116G           {0} ; 1232
 aabaa baaab bbbab bbaba aabaa bbbaa aabbb aabbb ababa bbaaa
 bbaba babaa bbbab baaab baaaa ababb baaba bbaab bbbbb aabba
 baaaa aaabb babab aaaaa babb- bbaba aabab abbaa ababb bbabb
 aaabb bbbab abbab aabab aabab baabb abbaa bba
EACTAG311V           {0} ; 1258
 aabba abaab bbbba bbaba babbb bbbba aabab aabbb abaaa bbbab
 ababa babaa bbbbb bbaab bbbaa bbbbb aaaba bbabb abbab baaba
 bbaba aaaab babbb abbaa babb- babba aabab aabaa bbbbb baaab
 aaabb bbbab aaaab abbab aabbb baabb aabaa bbb
EACTAG389V           {0} ; 1275
 aabba abaab bbbba bbaba babbb bbbaa aabab aabbb abaaa bbbab
 ababa babaa bbbbb bbaab bbbaa abbbb aaaba bbabb abbab baaba
 bbaba aaabb babbb abbaa babb- bbbba aaaab aabaa bbbbb baaab
 aaabb bbbab aaaab bbbab aabbb baabb aabaa bbb
EAGTCA314V           {0} ; 1309
 aabba abaab bbbba bbaba babbb bbbaa aabab aabbb abbaa bbbab
 ababa babaa bbbbb bbaab bbbaa bbbbb aaaba bbabb abbab baaba
 bbaba aaabb babbb abbaa babb- babba aabab aabaa bbbbb baaab
 aaabb bbbab aaaab abbab aabbb baabb aabaa bbb
EAGTCA315G           {0} ; 1310
 aabba abaab bbbba bbaba babbb bbbaa aabab aabbb abaaa bbbab
```

ababa babaa bbbbb bbaab bbbaa bbbbb aaaba bbabb abbab baaba
baaba aaabb babbb abbaa babb- babba aabab aabaa bbbbb baaab
aaabb bbbab aaaab abbab aabbb baabb aabaa bbb
EACTGA154G {0} ; 1358
aabaa bbaab bbbbb bbaba aabaa bbbaa aabab aabbb ababa abaaa
bbab- babaa bbbab ba-ab baaaa a-abb baaba bbaab bbbbb aabba
baaaa aaabb babab abaaa babba bbaba aabab abbaa ababb baabb
aaabb bbbab abbab aabab aabab baabb aabaa aba
EAGTGC64G {0} ; 1387
aaabb abaaa aaaab babab baabb abbaa aaaab abaaa babaa ababb
bbbb- ababa bbaba aa-bb ababa b-aab ababa aaaab aabab aabab
ababb babab bbabb aabab babbb aabaa ababa bbbab babba abbab
aabab abbab abbaa baaaa aabab abbba bbaaa baa
EAGTGC183G {0} ; 1395
aaabb abaaa aaaab babbb baabb abbaa aaaab abaaa babaa ababb
bbbb- ababa bbaba ab-ab ababa b-aab ababa aaaab aabab aabab
ababb babab bbabb aabab babbb aabaa abaaa bbbaa babba abbab
aabab abbab abbaa baaaa aabab abbba bbaaa baa
EAGTGC258V {0} ; 1401
aabaa bbaab bbbab bbaba aabaa bbbaa aabab aabbb ababa bbaaa
bbab- babaa bbbab ba-ab baaaa a-abb baaba bbaab bbbbb aabba
babaa aaabb babab abaaa babba bbaba aabab abbaa ababb baabb
aaaba bbbab abaab aabab aabab baaba abbaa aba
EAGTGC263G {0} ; 1402
aabaa bbaab bbbab bbaba aabaa bbbaa aabab aabbb ababa bbaaa
bbab- babaa bbbab ba-ab baaaa a-abb baaba bbaab bbbbb aabba
baaaa aaabb babab abaaa babba bbaba aabab abbaa ababb baabb
aaabb bbbab abbab aabab aabab baabb abbaa bba
ECGTGA249G {0} ; 1545
aaaab ab-aa abbaa -abbb babba babaa baab- bbbaa babaa ababb
bbbbb ababa bbaba abbaa ab--a ---ab ababa aaaab aaaab babab
bbabb aabab bbabb babab babbb aabaa bbaaa babaa babba abaab
aabbb abbab bbbaa abaaa a-bab abaaa bbaaa baa
SSR_732 {0} ; 1590
babaa abaaa abbba aabba babbb aabaa baabb abbaa baaab aabab
bbbaa aaaaa bbabb abbaa abaab baaab ababa aaabb aabab babab
bbabb baaab bbbbb babaa babbb aabaa bbbaa babaa babba abaaa
aabbb abbab bbba- abbaa aabbb abaaa baabb ba-

LG17:
EGAMGA163V {0} ; 17
aabab bbbbb aabbb babbb bbaaa bbbab abaab abaaa aaaab abbab
abbab aabba babaa baaba bbaaa bbaaa abaab abbba abbb- -----
----- ----- ----- ----- ----- ----- ----- ----- ----- -----
----- ----- ----- ----- ----- ----- ----- ---
EGAMGA531G {0} ; 45
abbba baaaa baaab bbaab bbbba abbab baaaa baaaa babbb abaab
babbb aabbb aabbb babaa babba bbaaa baaab babab baaa- -----
----- ----- ----- ----- ----- ----- ----- ----- ----- -----
----- ----- ----- ----- ----- ----- ----- ---
ECAMCG459G {0} ; 86
abbba baaaa baabb bbbab bbbba abbab baaaa baaaa babbb abaab
babbb aabbb aabbb babaa babba bbaaa baaab babab baaa- -----
----- ----- ----- ----- ----- ----- ----- ----- ----- -----
----- ----- ----- ----- ----- ----- ----- ---
EGAMGC117V {0} ; 107
aaaba bbbbb babbb babbb baaaa babaa ababa bbaaa aaaab abbab

```
 babbb aabba aabba babbb bbaaa bbaaa bbaab abbba babba abbaa
 aaaba bbbba baaba babbb ababa bbbbb bbaab aabab baaba baabb
 abbab aabaa aabbb babbb abbaa abaab baaaa bba
ECAMCC95V           {0} ; 140
 aaabb bbbbb aabbb babab abaaa bbbab abaab abaaa aaaab abbab
 abbab aabba babaa baaba bbaaa bbaaa abaab abbba -aabb abbab
 abbba babaa baaba babbb ababa ababb bbaaa aabab bbaba baaab
 abbab aabaa aabbb babbb babba abbba aabaa bbb
ECAMCC266V          {0} ; 159
 aaabb bbbbb aabbb babbb baaaa bbbab abaab abaaa aaaab abbab
 abbab aabba babaa baaba bbaaa bbaaa abaab abbba -bbbb abbab
 abbba babaa baaba babbb ababa ababb bbaaa aabab ababa baaab
 abbab aabaa aabbb babbb babba abbba aabaa bbb
ECAMCC326V          {0} ; 162
 aaaba bbbbb babbb babbb baaaa babab abaaa abaaa aaaab abbab
 aabbb aabba aabba babbb bbaaa bbaaa bbaab abbba -abba abbab
 abbba babba baaba babbb ababa abbbb bbaab aabab baaba baaab
 abbab aabaa aabbb babbb aabba abbbb aaaaa bbb
ECGMCC133G          {0} ; 181
 aaaba bbbbb babbb babbb baaaa babab abaaa abaaa aaaab abbab
 aabbb aabba aabba babbb bbaaa bbaaa bbaab abbba aabba abbab
 abbba bab-a baaba babbb ababa abbbb bbaab aabab baaba baaab
 abbab aabaa aabbb baaba aabba abaab aaaaa bbb
ECAMGG100V          {0} ; 247
 aabab bbbbb aabbb bbbbb baaba abbaa baaaa bbaaa aaaab abbab
 babbb aabbb aabbb ababa bbaaa bbaaa bbaab abbba babba abbbb
 aaaba babba baaba babbb abbba abbbb bbaab aabaa aaaba baabb
 abbab aabaa aabbb babbb abbaa abaab baaba bba
EACMGA167G          {0} ; 346
 aabbb bbbbb aabbb babbb abaaa bbbab abaab abaaa aaaab abbab
 abbab aabba babaa baaba bbaaa bbaaa abaab abbba abbbb abbab
 abbb- babaa baaba babbb ababa ababb bbaaa aabab bbaaa baaab
 abbab aabab aabbb baaba babba abbba aabaa bbb
EACMGA428G          {0} ; 377
 abbaa bbabb baabb bbabb babba abbaa baaaa baaaa babbb abaab
 babab aabbb aabbb bbbaa baaba bbaaa bbaab babba babaa aabbb
 aaab- bbbba bbaaa babbb abbaa abbbb bbaab aabaa aabba babbb
 abbab aaaaa abbbb baaaa abbbb bbaab baaba bba
EACMGA437V          {0} ; 378
 abbaa bbabb baabb bbabb babba abbaa baaaa baaaa babbb abaab
 babbb aabbb aabbb babaa baaba bbaaa bbaab babba babba babbb
 aaab- bbbba bbaaa babbb abbaa abbbb bbaab aabaa aabba babbb
 abbab aaaaa abbbb babab abbbb bbaab baaba bba
EAGMTA524G          {0} ; 426
 aabab bbbbb aabbb babbb bbaaa bbbab abaab abaaa aaaab abbab
 abbab aabba babaa b-aba bbaaa bbaaa abaab abbba abbbb abbab
 abbb- babaa baaba babbb ababa ababb bbaaa aabab bbaaa baaab
 abbab aabab abbbb baaab babba abbba aabaa bbb
ECAMCT452G          {0} ; 590
 aabab bbbbb abbbb babbb baaaa bbbab ababb abaaa aaaab abbab
 abbab aabba babaa baaba bbaa- bbaaa abaab abbba abbbb abbab
 abbba babaa baaba babbb ababa ababb bbaaa aabab bbaaa baaab
 abbab aabab aabbb baaab babba abbba babaa bbb
EACMCG264V          {0} ; 648
 abbba baaaa baabb bbaab babba abbab ababa baaaa babbb abaab
 babbb aabbb aabbb babaa babba bbaaa bbaab babab baaaa aaabb
 aaabb bbbba bbaaa babba abbaa abbbb bbaab aabaa aabba babab
```

aabab aaaab abbba babab bbbbb bbaab ababa bba
EACMCG267G {0} ; 650
abbba baaaa baabb bbaab babba abbab abaaa baaaa babbb abaab
babbb aabbb aabbb babaa babba bbaaa bbaab babab baaaa aaabb
aaabb bbbba bbaaa babba abbaa abbbb bbaab aabaa aabba babab
aabab aaaab abbba baaaa bbbbb bbaab ababa bba
EACMGG96V {0} ; 739
aabaa bbbbb babbb bbbbb baaba abbaa ababa bbaaa aaaab a-bab
babbb aabbb aabba babab bbaaa bbaaa bbaab abbbb babbb abbbb
aaaba bbbba babba bbbbb abbba abbbb b-aab aabaa aaaba baabb
abbab aabaa aabbb babbb abbaa abaab baaba bba
EACMGC286V {0} ; 897
aabbb bbbbb aabbb babbb abaaa bbbab abaab abaaa aaaab abbab
abbab aabbb babaa babba bbaaa bbaaa abaab abbba abbbb abbab
abbba babaa baabb babbb ababa ababb bbaaa aaaab bbabb bbabb
aabbb aaaba abbbb baaab aaaba bbaba ababa abb
EACMGC346V {0} ; 908
abbba baaaa babab abaab aabba abbab baaba baaaa babbb abbaa
babbb aabbb aaabb bbbaa aabba bbbaa bbaab baaab baaaa baabb
aaabb bbaba bbaaa babba ababb aaabb bbaab babba bbaba abbbb
babaa babba aaaaa abbab bbaba ababa aaaba bbb
EACMGC372G {0} ; 912
abbba baaaa babab abaab aabba abbab baaaa baaaa babbb abbaa
babbb aabbb aaabb babaa aabba bbbaa bbaab baaaa baaaa baabb
aaabb bbaba bbaaa babba ababb aaabb bbaab babba bbaaa abbbb
babaa babba aaaaa abbaa bbaba ababa aaaba bbb
EAGMCA513V {0} ; 975
aabab bbbbb aabbb babbb abaaa bbbab baabb abaaa aaaab abbab
abbab aabba babaa baaba bbaaa bbaaa abaab abbba abbbb abbab
abbba babaa baaba babbb ababa ababb bbaaa aabab bbabb bbabb
aabbb aaaba abbbb baaab aaaba bbaba ababa abb
EACMGT104V {0} ; 1009
aabab bbbbb aabbb babbb baaaa bbbab abaab abaaa aaaab abbab
aabab aabba babaa baaba bbaaa bbaaa abaab abbba abbbb abbab
abbba babaa baaba babbb ababa ababb bbaaa aabab abaaa baaaa
abbab aabab aabbb babbb aabba abbba aabaa bbb
EACMGT299V {0} ; 1027
aabab bbbbb aabbb babbb abaaa bbbab abaaa abaaa abaab abbab
abbab aabba aabaa baaba bbaaa bbaaa abaab abbba abbbb abbab
abbba babaa baaba babbb ababa ababb bbaaa aabab bbbaa baaab
abbab aabab aabbb babbb babba abbba aabaa bba
EAGMTT377V {0} ; 1074
abbba baaaa baabb bbaab babba abbab baaaa baaaa babbb abaab
babbb aabbb aabbb babaa babba bbaaa bbaab babab baaaa aaabb
aaabb bbbba abaaa babba abbaa abbbb bbaab aabaa aabba babab
aabab aaaab abbba babab bbbbb bbaab ababa bba
EAGTTA430G {0} ; 1170
aabab bbbbb aabbb babbb abaaa bbbab abaab abaaa aaaab abbab
ababb aabba babaa baaba bbaaa bbaaa abaab abbba abbbb abbab
ababa babaa baaba babbb ababa ababb bbaaa aabab bbaaa baaab
abbab aabab aabbb baabb babba abbba aabaa bbb
EACTAG120G {0} ; 1234
aabab bbbbb aabbb babbb bbaaa bbbab abaab abaaa aaaab abbab
ababb aabba babaa baaba bbaaa bbaaa abaab aabba abbbb abbab
aabba babaa baaba babbb abab- ababb bbaaa aabab bbaaa baaab
abbab aabab aabbb baabb babba abbba aabaa bbb
EACTAG141V {0} ; 1237

```
 aabab bbbbb aabbb babbb abaaa bbbab abaab abaaa aaaab abbab
 ababb aabba babaa baaba bbaaa bbaaa abaab abbba abbbb abbab
 abbba babaa baaba babbb abab- ababb bbaaa aabab bbaaa baaab
 abbab aabab aabbb babbb babba abbba aabaa bbb
EACTAG173G           {0} ; 1242
 aabab bbbbb aabbb babbb abaaa bbbab abaab abaaa aaaab abbab
 ababb aabba babaa baaba bbaaa bbaaa abaab abbba abbbb abbab
 aabba babaa baaba babbb abab- ababb bbaaa aabab bbaaa baaab
 abbab aabab aabbb baabb babba abbba aabaa bbb
EAGTCA479G           {0} ; 1317
 aabab bbbbb aabbb babbb baaaa bbbab abaab abaaa aaaab abbab
 aabbb aabba babaa baaba bbaaa bbaaa abaab abbba abbbb abbab
 aabba babaa baaba babbb abab- ababb bbaaa aabab bbaaa baaab
 abbab aabab aabbb baabb aabba abbba aabaa bbb
EAGTCA505V           {0} ; 1320
 aabab bbbbb aabba babbb baaaa bbbab ababb abaaa aaaab abbab
 aabbb aabba babaa baaba bbaaa bbaaa abaab abbba abbbb abbab
 abbba babaa baaba babbb abab- ababb bbaaa aabab bbaaa baaab
 abbab aabab aabbb babbb aabba abbba aabaa bbb
EACTGA324V           {0} ; 1375
 aabab bbbbb aabbb babbb abaaa bbaab abaab abaaa aaaab abbab
 abab- aabba babaa ba-ba bbaaa a-aaa abaab abbba aaabb abbab
 abbba babaa baaba babbb ababa ababb bbaaa aabab bbaaa baaab
 abbab aabab aabbb babbb babba abbba aabaa bbb
EACTTC76V            {0} ; 1441
 aabab bb-bb aabbb -bbbb baaaa babab abab- bbaaa aaaab abbab
 aabbb aabba babba baabb bb--a ---aa abaab abbba abbba abbab
 abbba babba baaba babbb ababa abbbb bbaaa aabab baaaa baaab
 abbab aabab aabbb babbb a-bba abbba aaaaa bbb
EACTTC325G           {0} ; 1466
 abbba ba-ba baabb -babb babba abbab baaa- aaaaa babbb abaab
 babbb aabbb aabbb babaa ba--a ---aa bbaab babaa baaaa aaabb
 aaabb bbbba bbaaa babba abbaa abbbb bbaab aabaa aabba babbb
 aabab aaaab abbba baaaa b-bbb bbabb ababa bba
EACTTC344bV          {0} ; 1470
 abbba ba-ba baabb -babb babba abbab baab- baaaa babbb abaab
 babbb aabbb aabbb babaa ba--a ---aa bbaab babab baaaa aaabb
 aabbb bbbba bbaaa babba abbab abbbb bbaab aabaa aabba babbb
 aabab aaabb abbba babaa b-bbb bbabb ababa bba
EAGTAA49G            {0} ; 1497
 aabaa bb-bb babbb -bbbb baaa- babab abaa- aba-a aa-ab a-bab
 -abbb aabba a-bba babbb bb--a ----a bba-b -b--- ----a abbab
 abbba babba baaba babbb ababa abbbb bbaab aabab baaaa baaab
 abbab aabab aabbb b--b- a-bba aaabb aaaaa bbb
EAGTAA306G           {0} ; 1517
 ----- ----- ----- ----- ----- ----- ----- ----- ----- -----
 ----- ----- ----- ----- ----- ----- ----- ----- ----b abbab
 abbba babaa baaba babbb ababa ababb bbaaa aabab bbaaa baaab
 abbab aabab aabbb b--b- b-bba aabba aabaa bbb
SSR_680              {0} ; 1587
 aabab bbbbb aabbb babbb baaab abbab abaab abaaa aaaab aabab
 aabbb aabba babaa baabb bbaaa bbaaa abaab abbba abbbb abbab
 abbba baaaa ba-ba babbb ababa ababb bbaaa aabab bbaaa baaab
 abbab aabab aabb- baabb aabba abbba aabaa bb-

LG18:
ECAMCG492V           {0} ; 87
```

```
 aabab bbbab bbaab abbaa bbbba bbbaa ababb aabaa baaba bbbbb
 baaaa abbaa aaaab abaab aaabb bbbba abbaa aabba abba- -----
 ----- ----- ----- ----- ----- ----- ----- ----- ----- -----
 ----- ----- ----- ----- ----- ----- ----- ---
ECAMCC169G            {0} ; 149
 aaaab babbb aaabb aaaaa aabab bbbab aaabb aaaaa ababb baaaa
 baabb bbbbb abbaa bbaab abbbb abbab aaaaa babaa -bbbb baaab
 baaab bbbaa bbbaa abaaa aabab babab bbbba babbb abbab ababa
 babbb aaaab abbab bbbba baaaa baaaa baabb abb
ECAMGG348G            {0} ; 274
 babbb bbaab abbaa aabaa babba bbbaa aaabb aabba abbbb baaab
 baaba abbaa aabbb ababa aaabb abbba aaaab abbaa baaab abbaa
 baabb aabaa bbaba bbaab aabab bbaab bbaaa baaba bbbaa ababa
 bbbba aaaaa abaab bbbba aaaba babba aaabb aaa
ECAMGG420G            {0} ; 286
 aabab bbbab bbbab aabaa aabba bbbaa baabb aabba ababa baaab
 baaba abbaa aabaa ababa aaabb abbba aaaab abbab aaaab abbaa
 baabb aabaa bbaaa bbabb aabab bbaab bbaba babba bbaaa ababa
 bbbbb baaba abaaa bbbba aaaba babbb aaabb aaa
ECGMTA237G            {0} ; 313
 aaaab babbb aaabb aabaa abbab bbbab aaabb aabaa aaabb baaaa
 baaba bbbbb baabb aabba abbbb abaab aaaaa aaaaa aabbb aaaab
 baaab bbbaa bbbaa abaaa aabab babab babba babbb abbbb ababa
 babbb aaaaa aabab bbbba baaaa baaaa aaabb aab
EACMGA230G            {0} ; 347
 aabbb babab bbaab abbaa bbbba bbbaa ababb aabba aaaba baabb
 baaba abbaa aaaab aaaab aaabb bbbba aaabb abbaa abbab abbaa
 baab- aabaa bbaaa bbaba aabab bbaab bbaab baaba bbaaa bbaba
 bbabb babba aaaaa bbbba baaaa aaaab babab bba
EACMGA425V            {0} ; 376
 babaa aaaab aaaba aaabb bbbbb bbbaa abaab aabab ababa babbb
 baaab aabab aabab aabab bbabb bbaba baaaa baaba bbbab baaaa
 baaa- aaaba ababb abbba ababa bbaab bbaab baaab bbbba ababb
 babab baaab bbaba bbaab bbbba abbba abbba baa
EACMGA541V            {0} ; 389
 baabb bbaab aaaab aabaa bbbba bbbaa aaabb abbba abaab baaab
 baabb abbba aabaa babab baabb abbba ababb bbbaa bbbab abaaa
 baab- babab abbba abbab abbbb ababb bbaab babba bbbbb abbba
 babba aabba bbabb bbbbb babba baaaa babbb aba
ECGMGA79G             {0} ; 428
 aabab babbb baaab bbaaa aabab babaa aaaaa ababa abbbb bbbaa
 baabb abbbb abaaa a-aab aabba babab aaaaa babaa aabab aabab
 aaaa- bbbaa abaaa aaaab aabba aaaaa bbbaa babbb bbbbb abbba
 babbb aaaba abbbb bbbab babaa baaaa baabb abb
EACMTC134bV           {0} ; 458
 aabab bbbab bbaab abbba bbbba bbaaa ababb aabaa baaba bbbbb
 baaaa abbaa aaaab abaab aaab- bbbba aabaa aabba abbab bbbba
 baaab abbba bbabb bbbaa aabab ababb abaab bbaaa abaaa bbaba
 bbaba babbb aaaaa bbbba bbbab abbbb babba baa
EACMTC138G            {0} ; 459
 aabaa abbab bbaaa abbaa bbbab baaaa ababb aabaa baaba bbbbb
 baaaa abbaa aaaab abaab aaab- bbbba aabaa aabba aaaab bbaba
 baaab ababa bbabb bbbaa aabab ababb abbbb bbbbb bbaaa bbbba
 bbaba babbb aaaaa bbbaa babab abbbb babba baa
EACMTC144G            {0} ; 462
 aabaa bbbab bbaab abbaa bbbba bbaaa ababb aabba aaaba baabb
 baaba abbaa aaaab aaaab aaab- bbbba aaabb abbaa abaab abbaa
```

```
 baabb aabaa bbaaa bbaba aabab bbaab bbaab baaba bbaaa bbaba
 bbabb babba aaaaa bbbaa baaaa aaaab babab bba
EACMTC439V          {0} ; 521
 aabab bbbab bbaab abbaa bbbba bbaaa ababb aabba baaba bbbbb
 baaaa abbaa aaaab aaaab aaab- bbbba aabba abbab abbab bbbaa
 baabb ababa bbaba bbbaa aabab ababb bbaab bbaba bbaaa bbaba
 bbaba babba aaaaa bbbba babab abaab babbb bba
EACMTC549G          {0} ; 532
 aaaab babbb bbabb aaaaa aabab baaaa aaaaa bbaba abbbb baaaa
 baabb bbbbb abbaa abaab abbb- aabab aaaaa babaa aabbb aaaab
 aaaab bbbaa abbaa abaaa aabab babab bbbaa babbb abbbb ababa
 babbb aaaba abbab bbbab baaaa baaaa baabb abb
ECAMCT402V          {0} ; 586
 aabbb aabab babab aabaa bbbba bbbba ababb abbba ababa baaab
 baaba abbaa aaaba babab baab- abbba aaaab abbab abaab bbbaa
 babbb aabaa bbaab bbabb bbbab bbaab bbbba babba bbaaa ababa
 bbbbb baaba ababa bbbba aaaba babbb aaabb aaa
ECGMTT87V           {0} ; 597
 babbb bbaab bab-a aabaa bbbba bbbaa aaabb aabba abbbb baaab
 baaba abbaa aabba babab aaa-- abbba aaaab abbba baaab abaaa
 baabb aabaa bbaba bbaab aabab bbaab bbaaa baaba bbbaa ababa
 bbbba aaaaa abaab bbbbb aaaba babba aaabb aaa
EACMCG53V           {0} ; 628
 babbb bbaab abbaa aabaa bbbba bbbaa aaabb aabba abbbb baaab
 baaba abbaa aabba babab aaabb abbba aaaab abbaa baaab abbaa
 baabb aabaa bbaba abaab aabab bbaab bbaaa baaba bbbaa ababa
 bbbba aaaaa abaab bbbbb aaaba babba aaabb aaa
EAGMGG217G          {0} ; 683
 aabab bbbab bbaab abbaa bbbba bbbaa aaaba aabba aaaba baabb
 baaba abbaa aaaab aaaab aaabb bbbba aaabb abbab abbab abbaa
 baabb aabaa bbaaa bbaba aabab bbaab bbaab baaba bbaaa bbaba
 bbabb babba aaaaa bbbba baaaa aaaab babab bba
EAGMGG246G          {0} ; 689
 babba abaab aabaa ababb abbbb bbbaa abaab aabab baaba babbb
 baaab abbaa aabab bbbab ababb bbaba aabaa aaaba bbbab babaa
 baaab aaaba ababb bbbaa aabab bbabb abaab baaab abbaa aaabb
 bbaab aaaab ababb bbaaa abaab abbba abbba baa
EACMGG132G          {0} ; 746
 aaaab babbb aaabb aabaa abbab bbbab aaabb aabaa aaabb b-aaa
 baaba bbbbb abbaa bbaab abbbb abbab aaaaa aabaa abbbb aaaab
 baaab bbbaa bbbaa abaaa aabab babab b-bba babab abbbb ababa
 babbb aaaaa abbab bbbab baaaa baaaa aaabb aab
EACMGC349V          {0} ; 910
 babba aaaab aaaba ababb bbbbb bbbaa ababb aabab ababa babbb
 baaab aabab aabab abbab bbabb bbaba aabaa baaba bbbab baaaa
 baaab aaaba ababb abbba abbba bbaab abaab baaab bbbaa bbbbb
 babbb aabbb abaaa bbbab aabbb aaabb baaba bab
EAGMCA110V          {0} ; 936
 babba aaaab aabba ababb bbbbb bbbaa baaab aabab ababa babbb
 baaab aabab aabab abbab bbabb bbaba aabaa baaba bbbab baaaa
 baaab aaaba ababb abbba abbba bbaab abaaa aaaab bbbaa abbbb
 babbb aabbb abaaa bbaab aabbb aaabb aaaaa aab
EAGMCA421V          {0} ; 968
 babba abaab aaaba ababb abbbb bbbaa baaab aabab baaba babbb
 baaab abbaa aabab abbab ababb bbaba aabaa aaaba bbbaa aabaa
 baaab aaaba ababb bbbaa aabab bbabb abaab baaab bbbaa aabaa
 babbb babbb abaaa bbaab aabbb baaba aaaba bab
```

```
ECGMCA168V          {0} ; 981
 babba abaab bbabb abbba abbbb bbbaa ababb aabab baaba babbb
 baaaa abbaa aaaab abaab ababb bbaba aabaa aaaba abbab babba
 baaab aaaba ababb bbbaa aabab bbabb abaab bbaab abbaa aabaa
 babbb babbb abaaa baaab babab baaba ababa aaa
EACMGT329V          {0} ; 1030
 aaaab babbb baabb aaaaa aabab babaa aaaba ababa abbbb baaaa
 baabb bbbbb abbaa abaab abbba aabab aaaaa babab aabbb aaaab
 aaaab bbbaa abbaa abaaa aabab babab bbbaa babbb abbbb ababa
 babbb aaaba abbab bbbbb baaaa baaaa baabb abb
EACMGT562V          {0} ; 1040
 aabab bbbab bbaab abbaa bbbba bbbaa ababb aabba aaaba baabb
 baaaa abbaa aaaab aaaab aaabb bbbba aaabb abbab abbab abbaa
 baabb aabaa bbaaa bbaba aabab bbaab bbaab baaba bbaaa bbaba
 bbabb babba aaaaa bbbbb baaaa aaaab babab baa
ECGMGG116V          {0} ; 1092
 aabab bbbab bbaab abbab bbbba bbbaa baabb aabba aaaba baabb
 baaba abbaa aaaab aaaab aaabb bbbba aaabb abbab abaab abb-a
 baaba aabaa bbaaa bbaba aabab bbaab bbaab baaba bbaaa bbaba
 bbabb babba aaaaa bbbbb baaaa aaaaa baaab bba
ECGMGG580G          {0} ; 1113
 babbb aaaab aabaa ababb bbbbb bbbaa baaab aabab bbaba babbb
 baaab abbab aabab abbab bbabb bbaba aabaa baaba bbbbb baa-a
 baaaa aaaba ababb bbbba abbba bbabb abaab baaab abbba aaabb
 baaab baaab ababb bbaaa ababb abbba abbba baa
EACTCC540G          {0} ; 1153
 aaaab babbb aaabb aaaaa aabab bbbab aaaab aaaaa ababb baaaa
 babab bbbbb abbaa bbaab abbbb abbab aaaaa babaa abbbb baaab
 baaab bbbaa bbbaa abaaa aabab babab bbbba babbb abbbb ababa
 babbb aaaaa abbab bbbbb baaaa baaaa baabb aab
EACTAG276V          {0} ; 1254
 aaaab babbb aaaab aabaa abbab bbbab aaabb aabaa aaabb baaaa
 babaa bbbbb abbaa bbaab abbbb abbba aaaaa aabaa abbbb aaaab
 bbaab bbbaa bbbaa abaaa aaba- babab bbbba babbb abbbb ababa
 babbb aaaaa abbab bbbbb baaaa baaaa aaabb aab
EAGTCA519G          {0} ; 1321
 baabb babab aaaab aabaa bbbba bbbaa aaaab aabaa aaabb baaaa
 babaa bbbbb abbaa bbbab ababb abbab aaaaa aabaa bbbbb aaaaa
 baabb abbaa abbba abaab aaba- abbab bbbba babba abbbb ababa
 babbb aaaaa abbab bbabb baaaa baaba aaabb aaa
EACTGA118V          {0} ; 1352
 aaaab babbb baaab baaaa aabab babaa aaaaa ababa abbbb bbbaa
 baba- abbbb abaaa ab-ab aabba a-bab aaaaa babaa aabbb aaaab
 aabab bbbaa abaaa aaaab aabba aaaaa bbbaa babbb abbbb abbaa
 babbb aaaba abbbb bbbbb babaa aaaaa baabb abb
EACTTC333G          {0} ; 1467
 aabab bb-ab bbaab -abaa bbbba bbbaa abab- aabaa baaba bbbbb
 baaaa ababa aaaab abaab aa--b ---ba aabaa aabba abbab bbbba
 baaab ababa bbabb bbbaa aabab ababb abaab bbaaa abaaa bbaba
 bbaba babbb aaaaa bbbba b-bab abbbb babba baa
SSR_974             {0} ; 1607
 ----b babbb aaa-b aabaa babab abbab aaabb aabaa aaabb bbaaa
 baaba bbbbb abbaa bbaaa ababb abbab aaaaa aabaa abbbb aaaaa
 baaab abbaa bbabb bbaab aabaa aaaab bbbba babba abaab ababa
 babbb aabaa abaa- bbaba aaaaa baaaa aaabb aaa
```

LG20:

```
ECAMCG105G           {0} ; 57
 aaabb aabaa aabba baaaa babbb babab abaab bbbaa abaaa baabb
 babbb abaaa bbabb bbbaa baaab aaaab babbb aabba baaa- -----
 ----- ----- ----- ----- ----- ----- ----- ----- ----- -----
 ----- ----- ----- ----- ----- ----- ----- ---
EGAMGC125V           {0} ; 109
 bbbba bbbba abbbb bbaba bbbab abbba aabab baaba aabab abbbb
 bbbaa babba abbaa bbaba baabb babaa ababb abaaa aabba baaab
 bbbbb baabb bbaab aaaab bbaba abbab ababb aabba abbba aaabb
 abbbb babbb babab abbab abbab abbba aaabb abb
EGAMGC481V           {0} ; 131
 bbbba bbbba ababb bbaba bbaab abbba aaabb babba aabab abbbb
 baaaa babba abaaa bbbaa bbbbb babaa abaaa baaaa bbbbb aaaab
 aabbb baabb baaab aaaab bbaba abbab abbbb abbba bbbba aaaaa
 abaab aaabb aabab bbbbb bbbab abbaa ababa aab
EGAMGC483G           {0} ; 132
 aabbb aabaa aabaa baaaa babbb babaa abaab bbbaa abaaa baabb
 babbb abaaa bbabb bbbaa baaab aaaaa babbb aabba baaab aabab
 bbbaa abbaa bbbaa abbab bbaba ababa abbaa bbaba ababa bbaba
 bbabb abaaa bbabb aaaaa babba baaba bbbab bba
ECGMTA445G           {0} ; 326
 bbbba bbbba abbbb bbaba bbbab abbba aabab baaba aabab abbbb
 bbbba babba baabb aabab bbabb babaa ababb abaaa aabba baabb
 bbbbb babbb bbaab aaaab bbbba abbab ababb aabba abbba aaabb
 ababb aabbb bbbab abbaa bbbab abbbb aaabb aab
ECGMGA155V           {0} ; 431
 abbbb aabaa aabba baaaa babbb baaab baaab bbbab abaaa aaabb
 babab aaaaa bbaba b-baa baaab aaaaa bbbbb aaaaa baaab aabab
 bbba- aabaa bbbaa abbab ababa ababa abbaa bbaba abaaa bbaba
 bbabb abaab bbaba aaaba baaba baaaa bbbab bba
ECGMGA373G           {0} ; 439
 aaaab aaaba abbba babaa bbbbb baabb aaabb abbab bbbbb ababb
 baabb babab aaaba b-bbb bbbbb baaaa bbbba aaaab abbbb bbbba
 abba- babaa abaab aabaa bbbba ababa aabbb bbaba abbab babba
 aaabb bbaab aabab aabab bbbba baaab aaaba bba
ECAMCT116V           {0} ; 546
 bbbba bbbba ababb bbaba bbaab abbba aabbb baaba aabab abbbb
 bbbaa babba abbaa bbaba bbab- babaa ababb bbaaa abbba aaaab
 bbbbb bbabb bbaab abaab bbaba abbab ababb aabba abbba aaaab
 ababb babbb babab abbba bbbab abbba aaabb aab
ECAMCT465G           {0} ; 591
 aaabb baaba abbba babaa bbbbb baabb aaabb abbab babba ababa
 baaab babab aaaba babbb bbbb- aabaa bbbba aaaaa abbbb bbbaa
 abbaa babaa abaab aaaaa bbbba ababa aabbb bbaba aabab baaba
 aaaab ababb abbab aabaa bbbba baaab aaaba bba
ECGMTT179G           {0} ; 602
 aaaaa bbbaa abb-a baaba babab abbba aaaab aaaaa aaaab abbab
 bbbaa babba abbab ababa baa-- babaa abaab abaaa aaaba baaab
 bbbbb baabb baaab aaaab bbaba abbab ababb aabaa abbba aaabb
 abbbb babbb babab bbaaa aabab abbba aaabb abb
ECGMGC298V           {0} ; 717
 baaba baaba ababb babba bbbbb baaaa aaabb abbaa babbb abbba
 baaab babaa aabaa bbbbb bbbba aabaa abbba baaab abbbb bbaab
 aabab bbbaa abaab aaaab bbbba abbbb aabbb ababa baaba aaaaa
 aaaab aaabb aabab babbb bbbbb babba ababa bba
ECGMGC322V           {0} ; 719
 aabab aaaba abbba babaa bbbbb baabb aaabb abbab babba ababb
```

```
 baaab babab aaaba babbb bbbbb aaaaa bbbba aaaab abbbb bbbba
 abbaa babaa abaab aabaa aabba abaaa aabbb bbaba abbab baaba
 aaabb bbaab aabab aabbb bbbba baaab aaaba bba
EACMGG245G           {0} ; 764
 bbbba bbbba abbbb bbaba bbbab abbba aabab baaba aabab a-bbb
 bbbaa babba abbaa bbaba baabb babaa ababb abaaa aabba baaab
 bbbbb baabb bbaab aaaab bbaba abbab a-abb aabba abbba aaabb
 abbbb babbb babab abbaa abbab abbba aaabb abb
EACMGG245V           {0} ; 765
 bbbba bbbba abbbb bbaba bbbab abbba aabab baaba aabab a-bbb
 bbbaa babba abbaa bbaba baabb babaa ababb abaaa aabba baaab
 bbbbb baabb bbaab aaaab bbaba abbab a-abb aabba abbba aaabb
 abbbb babbb babab abbba abbab abbba aaabb abb
EACMGG444V           {0} ; 789
 aabba aabaa aabba bbbbb babbb babaa abaab bbbaa abaaa b-abb
 babbb ababa bbabb bbbaa baaab aaaaa babbb aabba babab babab
 bbbaa aabaa bbbaa abbab bbaba ababa a-baa bbabb abaaa bbaba
 bbaba abaab bbabb aaaba babba baaaa bbbab bba
EAGMCT169G           {0} ; 813
 abaaa baaaa ababb bbaaa bbbab abbba aabab aaaba aabab a-bbb
 bbbaa babba abbaa bbaba baabb babaa ababb abaaa aabba baaab
 abbbb baabb bbaab aaaab bbaba abbab a-abb aabba abbba aaabb
 abbbb babbb babab abbaa abbab abbba aaabb abb
EACMGC273G           {0} ; 894
 abbbb aabaa aabba baaaa babbb baaab abaab bbbab abaaa aaabb
 babab aaaaa bbaba bbbaa baaab aaaaa bbbbb abaaa baabb aabab
 abbaa aabaa bbbaa abbbb ababa ababa abaaa bbaba aaaaa abaab
 abaaa abbba bbbaa baaaa babab babba baabb aab
EACMGC344V           {0} ; 907
 aabba aabaa aabba bbbab babbb babaa abaab bbbaa abaaa baabb
 babbb ababa bbabb bbbaa baaab aaaaa babbb aabba babab babab
 bbbaa aaaaa bbbaa abbab bbaba ababa abaaa bbaba baaaa ababb
 abaaa abbba bbbaa baaab babab babaa baabb bab
EAGMCA493G           {0} ; 973
 bbbba bbbba abbbb bbaba bbbab abbba aabab baaba aabab abbbb
 bbbaa babba abbaa bbaba baabb babaa ababb abaaa aabba baaab
 bbbbb baabb bbaab aaaab bbaba abbab ababb aabba babba aabba
 babbb aaaab babba bbbaa aabba bbbbb abbbb aba
ECGMCA444G           {0} ; 997
 aabaa aaaba abbba babaa bbbbb baabb aaabb abbab bbbba ababb
 baaab babab aaaba babbb bbbbb aaaaa bbbba aaaaa abbbb bbbba
 abbaa babaa abaab aabaa bbbba ababa aabbb bbaba baabb bbbbb
 abaaa baaab abbaa bbaba aabaa aabbb baaba aba
EAGMTT162V           {0} ; 1058
 aabaa aabba abbbb baaaa bbbbb baabb ababb abbab abaaa ababb
 babab aabaa baaba bbaaa babab aaaba bbbaa abaab babbb bbabb
 aabaa babaa bbbab abbaa bbbaa ababa ababa ababa abbab baaba
 aaabb bbaaa aaaba aabab bbaba bbaaa bbbba bba
EACTCC108G           {0} ; 1122
 aabaa aaaba abbba babaa bbbbb baabb aaabb abbab bbbba ababb
 baaab babab aaaba babbb bbbbb aaaaa bbbba aaaaa abbbb bbbba
 abaaa babaa abaab aabaa bbbba ababa aabbb bbaba abbab baaba
 aaabb bbaab aabab aabbb bbbba baaab aaaba bba
ECGTGT182G           {0} ; 1195
 aabba aabaa aabaa bbbab babbb bbbaa abaab bbbaa abaaa babbb
 babbb ababa bbabb bbbaa baaab aaaaa babbb aabba baaab aabab
 bbaaa aabaa bbbaa abbab bbaba abaaa abbaa bbabb bbaaa bbaba
```

```
 bbaba abaaa baabb aaaaa aabba baaba bbaab bba
EACTAG245V          {0} ; 1250
 bbaba bbbba abaab ababa bbbab aabba aaabb babba babab abbba
 baaba babaa aabaa bbbbb bbbab babaa abaab babaa bbbbb aaaab
 abbaa bbabb bbaab aaaab bbaa- abbaa aabaa aaaba abbba aaaaa
 aaaab aaabb aabab babba bbbab bbbaa ababa baa
EACTAG344V          {0} ; 1264
 aabaa aaaba abbba babaa bbbbb baabb aaabb abbab bbbba ababb
 baaab babab aaaba babbb bbbbb aaaaa bbbba aaaaa abbbb bbbba
 abbaa babaa abaab aabaa bbbb- ababa aabbb bbaba abbab baaba
 aaaaa bbaab aabab aabbb bbbba baaab aaaba bba
EACTAG346G          {0} ; 1265
 aabaa aaaba abbba babaa bbbbb baabb aaabb abbab bbbbb baabb
 baaab babab aaaba babbb bbbbb aaaaa bbbba aaaaa abbbb bbbba
 aabaa babaa abaab aabaa bbbb- ababa aabbb bbaba abbab baaba
 aaaab bbaab aabab aabbb bbbba baaab aaaba bba
EAGTCA474G          {0} ; 1316
 aabbb aabaa aabba baaaa babbb babba abaab bbbaa abaaa baabb
 babbb abaaa bbabb bbbaa baaab aaaaa babbb aabba baaab aabab
 babaa aabaa bbbaa abbab bbab- ababa abbaa bbaba abaaa bbaba
 bbabb abaab bbabb aaaaa babba baaba bbbab bba
EAGTGC243V          {0} ; 1400
 aabba aabaa aabba bbbab babbb babaa abaab bbbaa abaaa baabb
 babb- ababa bbabb bb-aa baaab a-aaa babbb aabba babab aabab
 bbbaa aabaa bbbaa abbab bbaba ababa abbaa bbabb abaaa bbaba
 bbaba abaab bbabb aaaaa babba baaba bbbab bba
EAGTGC272V          {0} ; 1404
 abbbb aabba abbab baaaa bbbbb baabb ababb abbbb abaaa ababb
 baab- aabaa baaba bb-aa babab a-aba bbbbb abaaa babbb aabab
 aabab aabaa bbbaa ababa bbbaa ababa abaaa bbaba abbab bbaba
 aabbb abaaa aaaba aabab baaba bbaaa bbbba bba
ECGTGA229V          {0} ; 1540
 baaba ba-ba ababb -abbb bbbbb baaaa aaab- abbaa babbb abbba
 baaab babaa aabaa bbbbb bb--a ---aa abbba baaab abbbb bbaab
 aabab bbbaa abaab aaaab bbbba abbbb aabbb ababa baaba aaaaa
 aaaab aaabb aabab babba b-bbb babaa ababa bba
SSR_1188            {0} ; 1619
 abbbb aabaa aabab baaaa bbbbb baabb ababb abbbb aaaaa ababb
 babab aabaa baaba bbaab babab aaaba bbbbb abaaa babab aabab
 aabab aabaa bbbaa ababa abbaa ababa abaaa bbbba aaaab bbaba
 abbbb abaab baab- aabaa baaba bbaaa bbbba bb-

LG21:
EGAMGA110G          {0} ; 11
 baaab aaabb ababb bbaaa bbbaa aabaa bbaab bbaab aaaaa bbbba
 aaaaa babba babaa bbbaa abbab bbbbb baaab bbbab bbab- -----
 ----- ----- ----- ----- ----- ----- ----- ----- ----- -----
 ----- ----- ----- ----- ----- ----- ----- ---
EGAMGA126G          {0} ; 13
 baaab aaabb ababb bbaaa abbaa aaaba abaab bbaab aaaaa bbbba
 aaaaa baaba bbbaa bbbaa abbab bbbbb baabb bbbab bbab- -----
 ----- ----- ----- ----- ----- ----- ----- ----- ----- -----
 ----- ----- ----- ----- ----- ----- ----- ---
ECAMCG224V          {0} ; 71
 bbaaa baaba baaab abbba bbbaa babaa ababb bbaaa abaab bbaab
 abbab baaba babaa aabaa babbb bbbab abbab bbbaa abbb- -----
 ----- ----- ----- ----- ----- ----- ----- ----- ----- -----
```

```
 ----- ----- ----- ----- ----- ----- ----- ---
ECGMCC73V           {0} ; 178
 ababb ababa ababa aaabb abaaa abbab abbbb bbaab bbaab babaa
 aaaba bbabb bbaab bbbbb baaab abbbb babab bbaaa baaba abbbb
 aaabb bbb-b baaab aabbb abaaa bbbab ababa baaba bbabb babaa
 ababb baaab abaab baabb aaaba abbbb babaa bba
EACMCC371G          {0} ; 225
 bbaab ababb ababb aaabb abaaa aaaab babab bbaab aaaaa abbaa
 aaaba baabb bbaab abbbb bbbab bbbbb bbaba babaa baaba ababb
 ababa abbab baaaa babbb abaab ababb ababa baabb bbabb bbbaa
 aaabb baabb abaab baaba abaaa abbba bbbaa aaa
ECAMGG268V          {0} ; 262
 bbaaa aaabb ababb baaaa abaaa aaaba baaab bbaab aaaaa abbaa
 aaaaa baaba bbabb abbaa bbbab bbbbb baabb babab bbaba ababb
 aaaba abbab aaaaa babbb aaaab ababb ababa bbabb baabb bbbaa
 aaabb baabb bbbab baabb ababa abaaa bbbaa aaa
ECAMGG269G          {0} ; 263
 bbaaa aaabb ababb baaaa abaaa aaaba baaab bbaab aaaaa abbaa
 aaaaa baaba bbabb abbaa bbbab bbbbb baabb babab bbaba ababb
 aaaba abbab aaaaa babbb aaaab ababb ababa bbabb baabb bbbaa
 aaabb baabb bbbab baaba ababa abaaa bbbaa aaa
ECAMGG481V          {0} ; 291
 baaaa aaabb ababb bbaab bbbaa aabba bbaab bbaab aaaaa bbbba
 aaaaa babba ababb abbaa abbab bbbbb baabb bbbab bbabb abbaa
 baaba abbab abaab baabb aaaab abbab abbaa bbabb aaabb bbbbb
 aaabb babba bbbaa baabb babba abaaa bbbaa bab
EAGMTA451V          {0} ; 424
 abbab abbba ababa aaabb abaaa abbab abbbb bbaab bbaab babaa
 aaaba bbabb bbaab b-bbb baaab abbbb ababa bbaaa baaba abbbb
 aaab- bbabb baaab aabbb abaaa bbbab ababa baaba bbabb babaa
 ababb baaab aaaab baabb aaaba abbbb babaa bba
EACMTC55G           {0} ; 447
 baaab aaabb ababb bbaba bbbab aaaaa bbaab abaab aaaaa bbaab
 aaaaa babba babaa bbbaa abba- bbbbb baaaa abbab bbabb abbaa
 baaba abaab aaaaa aaaab aaaab abbaa aabab bbabb baabb bbbbb
 ababb babbb bbbaa bbaaa bbbba abaab bbbba bab
EACMTC377G          {0} ; 507
 aabab abbba ababa aaabb abaaa abaab abbab bbaab bbbbb babaa
 aaaba bbabb bbaab bbabb baab- abbbb ababa bbaaa baaba abbbb
 aaaab bbabb baaab aabbb abaaa bbbbb ababa bbaba bbabb babaa
 ababa baaab abaab baaab aabba abbbb babab bba
EACMTC377V          {0} ; 508
 aabab abbba ababa aaabb abaaa abaab abbab bbaab bbbbb babaa
 aaaba bbabb bbaab bbabb baab- abbbb ababa bbaaa baaba abbbb
 aaaab bbabb baaab aabbb abaaa bbbbb ababa bbaba bbabb babaa
 ababa baaab abaab baabb aabba abbbb babab bba
ECAMCT100V          {0} ; 543
 baaaa baaba baaab abbba bbbaa babaa abaab bbaaa abaab bbaab
 abbab babba babaa aabaa babb- bbbab babab bbbaa abbba babaa
 baaaa abbab aaaaa aabab baaab abbaa babaa aabbb aabbb abbbb
 ababa babba babaa baaba bbaba abaab bbbba bba
ECAMCT198V          {0} ; 560
 bbaab aaaaa aaaab abbba bbbaa babaa abaab bbaaa ababb bbaab
 bbbbb baaaa babaa aabaa babb- babab aabbb bbbaa bbbba babaa
 baaab bbbaa aabaa aabaa baabb abbba babaa aabbb babbb bbbbb
 ababa babba babaa baaba bbaba abaab bbbba bba
ECAMCT199G          {0} ; 561
```

```
 bbaaa baaaa aaaab abbba bbbaa babaa abaab bbaaa ababb bbaab
 abbaa baaaa babaa aabaa aaab- babab aabbb bbbaa bbbba babaa
 baaab bbbaa aaaaa aabaa baabb abbba babaa abbbb babbb bbbbb
 ababa babba babaa baaaa bbaba abaab bbbba bba
ECGMTT60V           {0} ; 595
 bbaab aaabb aba-b baaaa abaaa aaaba baaab bbaab aaaaa abbaa
 aaaaa baaba bbbaa bbbaa bbb-- bbbbb baabb babbb bbaba ababb
 aaaba abaab aaaaa babbb aaaab ababb ababa bbabb baabb bbbaa
 aaabb baabb bbbab babbb ababa abaaa bbaaa aaa
EACMGG168V          {0} ; 752
 bbbab aaabb ababb baaab abaaa aaaaa ababb bbaab aaaaa a-baa
 aaaaa baabb bbbaa bbbbb bbbab bbbbb bbabb babab baaba ababb
 ababa abbab aaaaa babbb abaab ababb a-aba bbabb bbabb bbbaa
 aaabb baabb bbbab baabb abaaa abbba bbbaa aaa
ECGMGT242G          {0} ; 857
 bbaaa aaabb ababb aaaaa abaaa aaaaa baaab bbaab aaaaa a-baa
 aaaaa baabb bbbaa bbbbb bbbab bbbbb bbabb babaa baaba bbbab
 ababa abbab aaaaa babbb abaab ababb a-bba bbbbb bbabb bbbaa
 aaabb baabb bbbab baaab abaaa abbba bbbaa aaa
EACMGC140V          {0} ; 878
 baaab aaabb abbab bbaba bbbab aabaa bbaab abaab aaaaa bbaab
 aaaaa babba babaa bbbaa abbab bbbbb baaab bbbab bbabb abbaa
 baaba abbab aaaaa aaaab aaaab abbab bbbaa bbabb abaab bbabb
 aabaa bbbbb ababa abbbb bbbba aabbb abbab bba
EACMGC142G          {0} ; 879
 baaab aaabb abbab bbaaa bbaab aabaa bbaab abaab aaaaa abaab
 aaaaa babba babaa bbbaa abbab bbbbb baaab babab bbbbb abbaa
 baaba abbab aaaaa aaaab aaaab abbab bbbaa bbabb abaab bbabb
 aabbb bbbbb ababa abbba bbbba aabbb abbbb bba
EAGMCA120G          {0} ; 937
 abbab abbba abbaa aaabb abaaa abbab babab bbaab bbaab babaa
 aaaba bbabb bbaab bbbbb baaab abbbb ababa bbaaa baaba ababb
 aaabb bbabb baaab aabbb abaaa bbbab ababa baabb bbaab baaaa
 aabbb bbaba abaab babba abaaa babbb aaaba baa
EAGMCA190V          {0} ; 944
 abbab abbba abbaa aaabb abaaa abbab babab bbaab bbaab babaa
 aaaba bbabb bbaab bbbbb baaab abbbb ababa bbaaa baaba abbbb
 aaabb bbabb baaab aabbb ababa bbbab ababa baaba bbaab baaab
 aabbb bbaba abbab babbb bbaaa babbb aaaba baa
EAGMCA192V          {0} ; 945
 abbbb abbba abbaa aaabb abaaa abbab babab bbaab bbaab babaa
 aaaba bbabb bbaab bbbbb baaab abbbb ababa bbaaa baaba abbbb
 aaabb bbabb baaab aabbb abaaa bbbab ababa baaba bbaab baaab
 aabbb bbaba abbab babbb abaaa babbb aaaba baa
EACTCC399G          {0} ; 1145
 baaaa baaba baaab abbba bbbaa babaa abaab bbaaa abaab bbaab
 ababb baaaa babaa aabaa babbb babab aabbb bbbaa bbbba babaa
 baaaa abbab aaaaa aabaa baabb aabba babaa aabbb aabbb abbbb
 ababa babba babaa baaba bbaba ababb bbbba bba
EACTCC400V          {0} ; 1146
 baaaa baaba baaab abbba bbbaa babaa abaab bbaaa abaab bbaab
 ababb baaaa babaa aabaa babbb babab aabbb bbbaa bbbba babaa
 babaa abbab aaaaa aabaa bbabb aabba babaa aabbb aabbb abbbb
 ababa babba babaa baaba bbaba ababb bbbba bba
EAGTTA446V          {0} ; 1172
 baaab aaabb ababb bbaaa bbbaa aabaa bbaab bbaab aaaaa bbbaa
 aaaaa babba babaa bbbaa abbab abbbb baaaa bbbab bbabb abbaa
```

```
 babba abbab abaab baabb aaaab abbab abbaa bbabb aaaba bbbbb
 aaabb babba bbbaa baabb babba ababa bbbaa bab
EAGTTA464V           {0} ; 1175
 baaab baaba baaab ababa bbbab babaa bbaab abaab abaab bbaab
 ababb babba babaa abbaa aabab bbbab baaab bbbab bbabb abaaa
 babba abbab aaaaa aaaab aaaab abbab bbbaa bbabb aabbb bbbbb
 aaabb babba babaa baabb babba ababb bbbba bab
ECGTGT312G           {0} ; 1214
 aabab abbaa ababa aaabb abaaa abbab abbaa bbaab bbbba babab
 aabbb bbbbb bbaab aaabb baabb bbabb aaaaa bbbaa baaba abbbb
 aaaab bbabb baaab aabbb abaaa babbb abbbb bbaba bbbaa baaba
 ababa babaa aaaab baabb abbba ababb baaab bba
EACTAG279G           {0} ; 1255
 abbab abbba abaaa aaabb abaaa aabab abbab bbaab bbaab babaa
 aabaa bbabb bbaab bbbbb bbaab abbbb ababa bbaaa baaba ababb
 aaabb bbabb baaab aabbb abaa- bbbab ababa baabb bbabb babaa
 ababb baabb abaab baabb abaaa abbbb babaa aaa
EAGTGC488G           {0} ; 1413
 baaaa baaba baaab abbba bbbab babaa abaab bbaaa abaab bbaab
 abab- babba babaa aa-aa aabbb b-bab baaab bbbaa bbaba bbaaa
 baaba abbab aaaaa aabab baaab abbaa babaa babbb aabbb abbbb
 aaabb babba babaa baaba babba ababb bbbba bba
EAGTGC490V           {0} ; 1414
 baaaa baaba baaab abbba bbbab babaa abaab bbaaa abaab bbaab
 abab- babba babaa aa-aa aabbb b-bab baaab bbbab bbaba bbaaa
 babba abbab aaaaa aabab baaab abbaa babaa babbb aabbb abbbb
 aaabb babba babaa baaba babba ababb bbbba bba
ECGTCA305G           {0} ; 1430
 aabab abbba ababa aaabb abaaa abbab abbaa bbaab bbbbb babab
 aaba- bbabb bbaab ba-bb baabb b-bbb ababa bbaaa babba abbbb
 aaaab bbabb baaab aabbb abaaa babbb ababb bbaba bbaba baaaa
 ababa baaab abaab baabb abbba ababb baaab bba
SSR_927              {0} ; 1606
 bbaaa baaba -aaab abbba bbbab aa-aa abaa- b-aaa a-a-- -aaa-
 a--a- -aaaa babaa aa--a -a-bb babab a-b-- bbbaa bbbba -a-aa
 -aaaa abbab aaaaa aa-aa -aab- abb-a babaa aabbb a---b abbbb
 ababa babba baba- baaba bbab- ababb bbbba bb-

LG22:
EGAMGA406V           {0} ; 38
 aaaaa aabbb bbaab aabbb abbbb abbbb aabba ababa bbabb baaba
 aaaab aabaa bbaba babab aaaab abbba bbbbb aaabb aaaa- -----
 ----- ----- ----- ----- ----- ----- ----- ----- ----- -----
 ----- ----- ----- ----- ----- ----- ----- ---
EGAMGA576V           {0} ; 47
 aaabb babba bbaaa aabab bbbbb abbbb abbaa bbaaa bbabb baaaa
 baaab aaaaa bbaab babaa aaaab babaa abbab aaabb bbba- -----
 ----- ----- ----- ----- ----- ----- ----- ----- ----- -----
 ----- ----- ----- ----- ----- ----- ----- ---
ECAMCG177G           {0} ; 66
 aaaaa abbbb abbab babbb aabbb abbbb aabaa ababa bbbbb baaba
 abaab aabaa baaba babab aaaab ababb baabb aaabb baaa- -----
 ----- ----- ----- ----- ----- ----- ----- ----- ----- -----
 ----- ----- ----- ----- ----- ----- ----- ---
ECAMCG256G           {0} ; 74
 aaabb babba bbaba aabab babbb abbbb abbaa bbaaa bbabb baaaa
 baaab aaaaa bbaab babaa aaaab babaa aabab aaabb abba- -----
```

```
 ----- ----- ----- ----- ----- ----- ----- ----- ----- -----
 ----- ----- ----- ----- ----- ----- ----- ---
EGAMGC390V          {0} ; 126
 aabab babba bbaba aabab babbb aabbb bbbaa bbaaa bbabb aaaaa
 baaab aaaab bbbab babba aaaab babaa abbab baabb abaab baaba
 aaaba babba bbaaa babbb ababb bbbab baaab bbbab aaaaa baaba
 baaaa aaaab bbbba baaab bbaba bbaab babaa aba
EACMCC289V          {0} ; 220
 aaabb babba bbaba aabab babbb abbbb babba bbaaa bbabb baaaa
 baaab aaaaa bbaab bbaaa aaaab babaa abbab aaabb abbab baaab
 aaaba babba bbaaa abbbb ababb bbaab baaab bbbaa aaaba aaaba
 baaaa aaaaa bbaba baaab bbaaa bbaab abbaa abb
ECAMGG232V          {0} ; 259
 aaaaa abbbb abbbb babbb aabbb abbbb aabba ababa bbbbb baabb
 abaab aabaa abbab abaaa aaaaa aaabb bbabb aaabb baaab bbaba
 bbaab babaa baaaa aabbb baaab bbbbb abbab aabaa aabbb baaba
 baaaa baaaa ababa bbbbb aabab baaab babab aab
EACMTC116V          {0} ; 454
 aaaab abbbb abbbb babab aabbb abbbb aabba ababa bbbbb baabb
 abaab aabaa baaba baaaa aaaa- aaabb bbabb aaabb baaab bbaba
 bbaab babaa baaaa abbbb baaab bbbbb abbab babaa bbbbb baaba
 baaaa baaaa ababa bbbbb aabab baaab babab aab
ECAMCT79V           {0} ; 540
 aaaaa aabbb bbabb aabbb aabbb abbbb aabba ababa bbabb baaba
 aaaab aabaa baaba babab aaaa- abbba bbbbb aaabb baaab bbaaa
 aaaaa babba baaaa abbbb bbaaa bbbba aabaa aabaa ababa baaba
 baaaa baaaa bbabb bbbbb bbbbb baaab bbbab aab
ECAMCT104G          {0} ; 544
 aaaab babba bbabb aabbb aabbb abbbb aabba abaaa bbabb baaaa
 baaab aabaa bbaba babaa aaaa- aabba bbbbb aaabb abaab bbaaa
 aaaaa babba baaaa abbab bbaaa bbaba aabaa aabaa ababa ababa
 baaaa baaaa bbabb babab bbaaa baaab bbbab abb
EACMCG458V          {0} ; 662
 aaabb bbbba bbbba aabbb babbb aabbb bbbba bbbaa ababb aaaaa
 babab aaabb bbbab bbbba aaaab babaa abbab baabb abaab aaaba
 aaaba babba bbaaa babbb abbba bbbbb bbaab bbbab aaabb baaba
 baaba baaaa bbbba aaaab bbbba bbaab bbbba aba
EACMCG461G          {0} ; 663
 aaabb bbbba bbbba aabbb babbb aabbb bbbaa bbbaa ababb aaaaa
 babab aaabb bbbab bbbba aaaab babaa abbab baabb abaab aaaba
 aaaba babba bbaaa babbb abbba bbbbb bbaab bbbab aaabb baaba
 baaba baaaa bbbba aaaaa bbbba bbaab bbbba aba
EAGMGG213V          {0} ; 681
 aaaaa abbbb abbab babbb aabbb abbbb aabba ababa bbabb baaba
 abaab aabaa baaba babab aaaab ababb bbabb aaabb baaab bbaba
 abaaa babba baaaa abbbb bbbab bbbba aabaa aabaa abbbb baaba
 baaaa bbaaa bbaba bbbbb aabbb baaab babab aab
EAGMGG215V          {0} ; 682
 aaaaa abbbb abbab babbb aabbb abbbb aabba ababa bbabb baaba
 abaab aabaa baaba babab aaaab ababb bbabb aaabb baaab bbaba
 abaaa babba baaaa abbbb babab bbbba aabaa aabaa abbbb baaba
 baaaa baaaa bbaba bbbbb aabbb baaab babab aab
EAGMGG217bG         {0} ; 684
 aaaaa abbbb abbab babbb aabbb abbbb aabba ababa bbabb baaba
 abaab aabaa baaba babab aaaab ababb bbabb aaabb baaab bbaba
 abaaa babba baaaa abbbb babab bbbba aabaa aabaa abbbb baaba
 baaaa baaaa bbaba bbbba aabbb baaab babab aab
```

EACMGG409bV {0} ; 786
aaabb bbbba bbbba aabab babbb aabbb bbbba bbaaa ababb a-aaa
babab aaaab bbbab bbbba aaaab babaa abbab baabb abaab aaaba
aaaba babba bbaaa babbb abbba bbbbb b-aab bbbab aaabb baaba
baaba baaaa bbbba baabb bbbba bbaab bbbba aba
EACMGC419V {0} ; 916
aaabb bbbba bbbbb abbbb babbb babbb bbaba babaa ababb abaaa
babab aabbb bbbab bbbbb aaaaa babaa abbab baabb aaaab aaaaa
bbaba babba bbaaa babbb abbba abbbb baaab bbbaa aaaaa bbbbb
abbaa abbbb aaaaa aabbb aabab aabaa aaaab baa
EACMGC422G {0} ; 918
aaabb babba bbbba aabbb babbb babbb bbaba babaa ababb abaaa
babab aaabb bbbab bbbbb aaaaa babaa abbab baaba aaaab aaaba
bbaba babba bbaaa babbb abbba bbbbb bbaab bbbab aaaaa bbbbb
abbaa bbbbb aabaa aabba aabab aabab aaaab bbb
EACMGC463G {0} ; 920
aaabb babba bbbab aabbb aabbb abbbb aabba abaaa bbabb baaaa
baaab aabaa bbaab babaa aaaab bbbaa bbbbb aaabb abaab baaab
aaaba babba bbaaa abbbb abaaa bbabb baaaa abbaa ababa bbbaa
abbaa babba babba aabaa babab aaaaa aaaab bab
ECGMCA206V {0} ; 984
aaabb babba bbbaa aabab babbb abbbb abbba bbaaa bbabb baaaa
baaab aaaaa bbaab babaa aaaab babaa abbab aaabb abbab baaab
aaaba babba bbbaa abbbb ababb bbaab baaab bbbaa abaaa bbbaa
abbaa babba aabba aabab aabab abbaa aaabb bab
ECGMCA209G {0} ; 985
aaabb babba bbbaa aabab babbb abbbb abbaa bbaaa bbabb baaaa
baaab aaaaa bbaab babaa aaaab babaa abbab aaabb abbab baaab
aaaba babba bbaaa abbbb ababb bbaab baaab bbbaa abaaa bbbaa
abbaa babba aabba aabaa aabab aaaaa aaaab bab
EAGMTT151V {0} ; 1054
aaaaa aabbb bbabb aabbb aabbb abbbb aabaa ababa bbabb baaba
aaaab aabaa bbaba babab aaaab abbba bbbbb aaabb aaaab babaa
aaaaa babba baaaa abbbb bbaaa bbbba aabaa aabaa ababa aaaba
baaaa baaaa bbabb bbbab bbbbb baaab bbbab aab
EAGMTT151G {0} ; 1055
aaaaa aabbb bbabb aabbb aabbb abbbb aabba ababa bbabb baaba
aaaab aabaa bbaba babab aaaab abbba bbbbb aaabb aaaab babaa
aaaaa babba baaaa abbbb bbaaa bbbba aabaa aabaa ababa aaaba
baaaa baaaa bbabb bbbaa bbbbb baaab bbbab aab
EACTAG241G {0} ; 1248
aabaa aabba bbaaa aabab babbb abbbb abbaa bbaaa bbabb baaaa
baaab aaaaa bbaab babaa aaaab babaa abbab aaabb abbbb baaab
aaaba babba bbaaa abbbb abab- bbaab baabb bbbaa baaba aaaba
baaaa aaaaa bbaba baaab bbaaa bbabb abbaa abb
EACTAG272G {0} ; 1253
aaaaa abbbb abbab babbb aabbb abbbb aabaa ababa bbbbb baaba
abaab aabaa baaba babab aaaab ababb bbabb aaabb baaab bbaba
aaaaa babba baaaa abbbb baba- bbbba aabaa aabaa bbbbb baaba
baaaa baaaa ababa bbbbb aabbb baabb babab aab
EAGTCA132V {0} ; 1289
aaaaa abbbb abbbb babbb aabbb abbbb aabba ababa bbbbb baabb
abaab aabaa baaba babaa aaaaa aaabb bbabb aaabb baaab bbaba
bbaab babaa baaaa aabbb baaa- bbbbb abbab babaa bbbbb baaba
baaaa baaaa ababa bbbbb aabab baabb babab aab
EAGTGC515V {0} ; 1416
aaaaa abbbb abbab babbb aabbb abbbb aabba ababa bbbbb baaba

```
 abaa- aabaa baaba ba-ab aaaaa a-abb bbabb aaabb baaab bbaba
 abbaa babba baaaa abbbb baaab bbbba aabaa aabaa bbbbb baaba
 baaaa baaaa ababa bbbbb aabbb baabb babab aab
EAGTGC518G          {0} ; 1417
 aaaaa abbbb abbab babbb aabbb abbbb aabaa ababa bbbbb baaba
 abaa- aabaa baaba ba-ab aaaaa a-abb bbabb aaabb baaab bbaba
 abaaa babba baaaa abbbb babab bbbba aabaa aabaa bbbbb baaba
 baaaa baaaa ababa bbbbb aabbb baabb babab aab
ECGTGA298V          {0} ; 1550
 abaaa ab-bb abbbb -abba aabab abbbb aabb- ababa bbbbb aaabb
 ababb aabaa baaba babaa aa--a ---bb bbaab baabb bbbbb bbaba
 bbbab bbbaa aaaaa aabbb aaaab babbb abaab babaa bbbbb baaab
 baaaa baaaa aaaba bbbbb a-bab bbaab aaaab aab
ECGTGA300G          {0} ; 1551
 abaaa ab-bb abbbb -abba aabab abbbb aabb- ababa bbbbb aaabb
 ababb aabaa baaba babaa aa--a ---bb bbaab baabb bbbbb bbaba
 bbaab bbbaa aaaaa aabbb aaaab babbb abaab babaa bbbbb baaab
 baaaa baaaa aaaba bbbbb a-bab bbaab aaaab aab
ECGTGA333V          {0} ; 1553
 aaabb ba-ba bbaba -abab babbb aabab abba- bbaaa bbabb baaaa
 baaab aaaab bbaab babaa aa--b ---aa abbab baabb abbab baaab
 aabba babba bbaaa babbb ababb bbaab baaab bbbaa aaaba aaaba
 baaaa aaaaa bbaba baaab b-aaa bbabb babaa aba
ECGTGA335G          {0} ; 1554
 aaabb ba-ba bbaba -abab babbb aabbb abba- bbaaa bbabb baaaa
 baaab aaaab bbaab babaa aa--b ---aa abbab baabb abbab baaab
 aaaba babba bbaaa babbb ababb bbaab baaab bbbaa aaaba aaaba
 baaaa aaaaa bbaba baaab b-aaa bbabb babaa aba
SSR_1073            {0} ; 1611
 aaaaa abbbb abbab aabbb aabba bbbbb aabba ababa bbabb baaba
 aaaab aabaa baaba babaa abbab abbba bbabb aaabb baabb bbaaa
 abaaa baaba baaaa abbbb baaaa bbbba aabaa aabaa abbba baaba
 baaaa baa-b bbab- bbbbb babbb baabb bbbab aab

LG23:
EGAMGA255V          {0} ; 26
 baaba aaaba abaab aaaba baaba abbab babbb babba baabb aabba
 aaaba abaab baabb abbba babba aabba bbabb aaaba baab- -----
 ----- ----- ----- ----- ----- ----- ----- ----- ----- -----
 ----- ----- ----- ----- ----- ----- ----- ---
EGAMGA389V          {0} ; 37
 baaba baabb abaab bbbbb bbaaa ababb aabbb bbbba baaba ababa
 baaaa abbaa bbbbb abbbb baaaa babaa bbabb bbaba baab- -----
 ----- ----- ----- ----- ----- ----- ----- ----- ----- -----
 ----- ----- ----- ----- ----- ----- ----- ---
ECAMCG147G          {0} ; 64
 baabb ababb bbabb babba baaaa ababb aaabb bbbba baaba ababa
 baaaa abbaa bbbbb bbbbb baaaa babaa baabb bbaba baab- -----
 ----- ----- ----- ----- ----- ----- ----- ----- ----- -----
 ----- ----- ----- ----- ----- ----- ----- ---
ECAMCG241G          {0} ; 72
 ababa aaaba abaab baaba baaaa abbba abbbb babba baabb aabbb
 baaba bbaab aaabb ababb babbb babbb babbb bbaba babb- -----
 ----- ----- ----- ----- ----- ----- ----- ----- ----- -----
 ----- ----- ----- ----- ----- ----- ----- ---
ECAMCC389V          {0} ; 166
 babaa aaaba abaab aaaba baaba abbab babbb babba baabb aabba
```

```
 aaaba abaab aaabb abbba babba aabba bbabb ababa -abbb bbabb
 aaaab aabbb baabb babba aabab babbb bbabb baaab baabb bbbba
 bbaba baaaa aaaaa aabab abbab abbba bbaab aaa
ECGMCC145V          {0} ; 182
 bbbab ababb bbaba babbb babaa bbbbb bbaba babba abaab ababa
 aaaaa ababa bbbbb bbbbb abbaa babaa aaaaa ababb aaabb baabb
 bbaba bab-a abbba abbaa aabaa bbbba baaab abaab baaba baaba
 ababa baaaa baabb aaaab babba bbaab ababb bba
EACMGA365G          {0} ; 372
 baabb ababb bbabb babba baaaa ababb aaabb bbbba baaba ababa
 baaaa abbaa bbbbb bbbbb baaaa babaa baabb bbaba baaab baaab
 bbbb- baaaa baabb baaba aaaaa baaba aaaab bbabb babab baaba
 bbaaa baaab bbaaa aabba ababa abaaa babab bba
EACMGA468G          {0} ; 381
 abaaa aaaba abaab baaba baaaa abbba abbbb babba baaab aabbb
 baaaa bbaab aaabb ababb aabbb babbb bbbba bbaba aabbb bbabb
 abab- aabbb baabb babba aabab babbb bbbbb aaaab bbaab abbaa
 bbaba baaab aaaab aaaaa abbab abbbb bbbab bbb
ECGMGA336V          {0} ; 438
 babba ababb bbaba babba abaaa ababb aaabb bbbba baaba bbaba
 baaaa abaaa bbabb b-bbb baaaa babaa baabb bbaba baaab baaab
 bbbb- babaa baabb baaaa aaaaa babba aaaab abaab babab baaba
 bbaaa baaab bbaaa aabba abbba abaab babbb bba
EACMTC227V          {0} ; 482
 bbabb ababb bbaba babbb babaa bbbbb bbaba babba abaab ababa
 aaaaa ababa babbb bbbbb abba- babaa aaaaa ababb aaabb baabb
 bbaba babaa abbba abbaa aabaa bbbba baaab abaab baaaa baaba
 ababa baaab bbabb aabbb babba bbaab ababb bba
EACMCG322G          {0} ; 652
 bbabb abbbb bbbba babbb babab bbbbb bbaba babaa abaab ababa
 aabaa ababb babab bbabb abbaa babaa aabaa bbaba aaaab aaaba
 bbaba babba abbaa abbaa aabaa bbbba bbaab abaab baaaa baaba
 bbaba baaaa bbaba aabaa bbbba abaab abbba baa
EACMGG116G          {0} ; 743
 baaba bbbbb bbabb bbbbb aaaaa ababb aabbb bbbba baaba a-aba
 baaaa abbaa bbbbb abbbb baaaa babaa baabb bbaba baabb baaab
 abbaa aabaa baabb baaba aabaa baaba b-abb bbabb babab baaba
 bbaaa baaab bbaaa babaa ababa abbba babab bba
EACMGG175V          {0} ; 756
 ababa aaaba abaab baaba baaaa abbba abbbb babba baaab a-bbb
 baaab bbaab aaabb ababb aabbb babbb bbbba bbaba aabbb bbabb
 ababb aabbb baabb babba aabab babbb b-bbb aaaab bbaab bbaaa
 bbbba baaab aaaab aaabb abbab abbbb bbaab bbb
EACMGG367G          {0} ; 780
 bbabb ababb bbbba babbb babaa bbbbb bbaba babbb abaab a-bba
 aaaba ababb bbbbb bbabb abbaa babaa abaaa bbabb aaabb aaabb
 bbaba babba abbaa bbbaa aabaa bbbba b-bab abaab baaba bbbba
 bbabb baaab bbabb aabab bbbba abaab ababa baa
EACMGG368V          {0} ; 781
 bbabb ababb bbbbb babbb babaa bbbbb bbaba babbb abbab a-bba
 aaaaa ababb babbb bbabb abbaa babaa aaaaa bbabb aaabb aaabb
 bbaba babba abbaa abbaa aabaa bbbba b-aab abaab baaaa baaba
 bbaba baaab bbabb aabbb bbbba abaab ababa baa
ECGMGT519G          {0} ; 864
 baaba ababb bbaba babba babaa bbbbb baabb bbbba baaab b-aba
 baaaa ababa bbabb bbbba aaaaa babaa aaaaa bbabb aaabb baabb
 bbaba babaa abbba bbbaa aaaaa baaba a-aab abaab babbb baaba
```

abaaa baabb bbabb aabaa babba bbaab aabbb bba
ECGMCA143G {0} ; 980
bbabb ababb bbbba babbb babaa bbbbb bbaba babba abaab ababa
aaaaa ababa babbb bbabb abbaa babaa aaaaa bbaba aaabb baabb
bbaba babaa abbaa abbaa aabaa bbbba baaab abaab baaba bbabb
aabaa babab abbab aaaaa aabaa babab aaaab bab
EACMGT178G {0} ; 1018
aaaaa aaaaa bbaaa aabaa baaaa abbbb babbb babba baabb aaaba
aaaaa abbab babbb abbbb babba aabba bbabb bbaba aaaab bbaab
abaab aabaa baabb babba aabaa baabb bbaab baabb baaab bbbba
bbbaa baaab bbaaa aabaa abbab abbba bbbab bba
EACMGT182V {0} ; 1019
bbaba aaaaa bbabb abbba baaaa abbbb babbb babba baabb aaaba
aaaaa abbab babbb abbbb babba aabba bbabb bbaba baabb bbaab
abaab aabaa baabb babba abbaa baabb bbaab baabb baaab bbbba
bbbbb baaab bbaaa aabab abbab abbba bbbab bba
EACMGT260G {0} ; 1024
baabb ababb bbaaa babba aaaaa ababb ababb bbbbb baaaa bbaba
baaaa abaaa bbabb bbbbb baaaa babaa aaaba bbabb baabb baabb
bbbba babaa aaabb baaaa aaaaa babba aaaab ababb babab baaba
bbaaa baabb bbaba aabba aabba bbaab babbb bba
ECGMGG135G {0} ; 1093
baaba aaaba bbaab abbba baaaa abbbb abbbb babba baabb aaaba
aaaaa abbab bbbbb abbbb baaba aabba bbabb bbaba baaab baa-b
abaaa aabaa baabb babba aabaa baabb bbaab baabb baaab bbbba
bbaaa baaaa bbaaa babaa abaab abbba bbbab bba
ECGMGG289G {0} ; 1096
baaba ababb bbaba babba aaaaa ababb aaabb bbbba baaba bbaba
baaaa abaaa bbabb bbbbb baaaa babaa baabb bbaba baabb baa-b
bbbbb babaa baabb baaaa aaaaa babba aaaab ababb babab baaba
bbaaa baaab bbaaa aabba abbba abaab babbb bba
ECGMGG292V {0} ; 1098
babba ababb bbaba babba abaaa ababb aaabb bbbba baaba bbaba
baaaa abaaa bbabb bbbbb baaaa babaa baabb bbaba baabb baa-b
bbbbb babaa baabb baaaa aaaaa babba aaaab ababb babab baaba
bbaaa baaab bbaaa aabbb abbba abaab babbb bba
ECGTGT487G {0} ; 1221
ababa aaaba abaab baaba baaaa abbba abbbb babba baaab aabbb
baaaa bbabb ababb ababb aabbb bbbbb bbbba bbaba aaabb bbabb
ababb aabbb baabb babba aabab babbb bbbbb aaaab bbaab abbaa
bbaba baaab aaaab aaaab abbab abbab bbbab bbb
EAGTCA165V {0} ; 1293
bbabb abbbb bbbba babbb babab bbbbb bbaba babaa abaab ababa
aaaba ababb babab bbabb abbaa babaa aabaa bbabb aaaab aaaba
bbaba babba abbaa abbaa aaba- bbbba bbaab abaab baaaa baaba
bbaba baaaa bbaba aabab bbbba ababb abbba baa
EAGTCA225G {0} ; 1301
bbabb abbbb bbbba babbb babab bbbbb bbaba babaa abaab ababa
aaaba ababb babab bbabb abbaa babaa aabaa bbabb aaaab aaaba
baaba babba abbaa abbaa aaba- bbbba bbaab abaab baaaa baaba
bbaba baaaa bbaba aabab bbbba ababb abbba baa
EACTTC223G {0} ; 1456
baabb ab-bb bbabb -bbba baaaa ababb aaab- bbbba baaba ababa
baaaa ababa bbbbb bbbbb ba--a ---aa baabb bbaba baabb baaab
bbaba babaa baabb baaba aaaaa baaba aaaab bbabb babab baaba
bbaaa baaab bbaaa aabba a-aba ababa babab bba
EACTTC531V {0} ; 1491

```
 baabb ab-bb bbabb -bbba baaaa ababb aaab- bbbba baaba ababa
 baaaa ababa bbbbb bbbbb ba--a ---aa baabb bbaba baaab baaab
 bbbba babaa baabb baaba aaaaa baaba aaaab bbabb babab baaba
 bbaaa baaab bbaaa aabba a-aba ababa babab bba
ECGTGA446V           {0} ; 1564
 baaba aa-ba abaab -aabb baaba abbab babb- babba baabb aabba
 aabaa abaab baabb abbba ba--a ---ba bbabb aaaba baabb bbabb
 aabab aabba baabb babba aabaa babbb bbabb baabb baaab bbbba
 bbbba baaab aaaaa aaabb a-bab abbaa bbaab aba
SSR_1068             {0} ; 1610
 ababa aaaba abaab baaba baaaa abbba abbaa babba baaab aabbb
 baaaa bbaab aaabb ababa aabbb abbbb bbbba aaaba aabab bbabb
 ababb baabb babbb babba aabab babba aabbb aaaab bbaab bbbaa
 bbbba baaab abaa- aaaab abbab abbab bbaab bbb

LG24:
ECAMCG323V           {0} ; 78
 aaaba bbbaa aaaaa abaaa aabbb bbabb babba abaaa aabab bbbba
 abbbb babbb bbbaa bbaaa abbaa abbab bbbaa ababa bbab- -----
 ----- ----- ----- ----- ----- ----- ----- ----- ----- -----
 ----- ----- ----- ----- ----- ----- ----- ---
EACMGA160V           {1} ; 344
 babbb aaabb abbba bbbbb bbaab aaaaa abbab ababb abbba aabab
 baaaa abaaa aaabb baabb baabb bbaba aaaba bbabb babbb baabb
 abaa- baabb babba ababa baaaa bbbaa aabba ababa baabb aabaa
 aaaaa aabba bbbab abbab babab bbbba ababb bba
EACMTC342G           {0} ; 499
 aaaba bbbaa baaaa abaaa aabbb bbbbb babaa bbbaa babab bbbba
 abbbb babbb bbbaa abaaa abba- abbab bbbab ababa bbaba bbbaa
 babba abbaa abaab bbbbb abbba aaabb bbabb babab abbaa aaabb
 bbaba bbaab aaabb baaab aabba baaab abaaa aab
ECAMCT208G           {0} ; 563
 abaaa bbabb bbabb abbaa babbb aabbb aaaba babab bbaab abbaa
 ababa baaab babbb aabaa bbaa- babbb aaaaa aabbb aaaaa bbbbb
 babbb abbaa aaaab baaab babbb baabb baabb babbb abbbb bbabb
 bbabb aaabb aabba baaab abbaa babbb aaabb bab
ECGMTT97V            {0} ; 598
 abaaa baabb baa-b abbaa babab abaab ababa bbbbb bbaab abbab
 aaaba babab abaab aabbb baa-- bbbbb abaaa abaab bbaab bbbaa
 bbbab bbaaa bbbba aabab babbb bbbbb baaba baaba abbbb baaba
 bbaba aabbb aaaaa babbb bbbba baaab babbb bab
ECGMGC66V            {0} ; 705
 abaaa bbabb bbabb abbaa babab aaaab bbaaa babbb bbaab abbab
 ababa babab bbaab aabab aaabb bbbbb aaaaa aaaab aaaaa bbbab
 bbbbb abaaa baaaa aabab babbb babbb aaabb baaba abbbb baabb
 bbaba aaabb aabaa baabb abbaa baaab babbb bab
ECGMGC66G            {0} ; 706
 abaaa bbabb bbabb abbaa babab aaaab bbaaa babbb bbaab abbab
 ababa babab bbaab aabab aaabb babbb aaaaa aaaab aaaaa bbbab
 bbbbb abaaa baaaa aabab babbb babbb aaabb baaba abbbb baabb
 bbaba aaabb aabaa baaba abbaa baaab babbb bab
ECGMGC103V           {0} ; 707
 aaaba bbbaa aaaaa abaab abbbb bbbbb abbba bbbaa babab bbbba
 abbbb babbb bbbaa abaaa abbaa abbab bbbaa ababa bbaba bbbaa
 babba abbaa abaab bbbbb abbba aaabb ababb babab abbaa aaabb
 bbaaa bbaab aaabb baabb aabba baaab abaaa aab
ECGMGC104G           {0} ; 708
```

aaaba bbbaa aaaaa abaaa aabbb bbbbb abbaa bbbaa babab bbbba
abbbb babbb bbbaa abaaa abbaa abbab bbbaa ababa bbaaa babaa
babba abaaa abaab bbbbb abbba aaabb ababb babab abbaa aaabb
bbaaa bbaab aaabb baaba aabba baaab abaaa aab
EACMGG1211V {0} ; 744
ababa bbbaa baaab aaaaa aabbb bbbbb baaba babaa babab b-aba
abbbb babbb bbbaa abbaa bbbaa aabab bbbab aabba ababa bbbaa
babba abbaa abaab babab abbbb aaabb b-aab babab abbaa bbabb
bbbbb bbaab aaaba baabb abbba aaaab babaa aab
EACMGG165G {1} ; 751
abaaa aaabb bbbab babbb bbaaa aaaaa abaab aabaa bbaba a-aab
baaaa abaaa aabab baabb baabb baaba aaabb babab aabab baabb
aaaab baabb babba aaaaa baaab bbbaa b-baa ababa baabb bbbaa
aabbb babba bbbaa bbbaa bbaab abbba babba bba
EACMGG261V {0} ; 768
aaaba bbbaa aaaaa abaaa aabbb bbbbb babba bbbaa babab b-bba
abbbb babbb bbbaa abaaa abbaa abbab bbbaa ababa bbaba bbbaa
babba abbaa abaab bbbbb abbba aaabb a-abb babab abbaa aaabb
bbaaa bbaab aaabb baabb aabba baaab abaaa aab
EAGMCT275G {0} ; 825
abaaa bbabb bbabb abbaa babab aaaab bbaaa babbb bbaab a-bab
ababa babab bbaab aabab baaab bbbbb aaaaa aabaa aaaaa bbbab
bbbbb abaaa baaaa aabab babbb bbbbb a-abb baaba abbbb baabb
bbaba aaabb aabaa baaab bbbba baaab babbb bab
EAGMCT313G {0} ; 828
ababa bbabb bbabb abaaa aabbb babbb aaaaa babab baaab b-bba
abbba baaab bbbba aabaa bbbaa babbb aabba aabba abaaa bbbab
babba abbab aaaab babab aabbb aaabb b-bab babab abbab bbabb
bbabb baaab aabba baaab abbaa babbb baabb bab
EACMGC332G {0} ; 905
aaaba bbbaa baaab aaaaa aabbb bbbbb babaa babaa babab bbbba
abbbb babbb bbbaa abaaa bbbaa aabab bbbab aabba bbaba bbbaa
babba abbaa abaab bbbbb abbbb aaabb bbabb babab abaab baabb
abaaa bbaaa aaaba bbaaa babbb babab baaba aab
EACMGT123G {0} ; 1012
ababa bbabb bbabb abaaa aabbb aabbb aaaba babab bbaab abbba
ababa baaab bbbbb aabaa bbbaa babbb aabba aabba aaaaa bbbab
babba abbaa aaaab babab aabbb aaabb babbb babab abbbb bbabb
bbabb aaaab aabba baaba abbaa babbb baabb bab
ECGTGT85G {0} ; 1177
ababa bbbaa baaab aaaaa aabbb bbbbb baaba babaa babab bbaba
abbbb babbb bbbaa abbaa bbbaa babab bbbab aabba abbba bbbaa
baaba abbaa abaab babab abbbb aaabb bbaab babab abbaa bbabb
bbbbb bbaab aaaba baabb abbba aaaab babaa aab
ECGTGT87V {0} ; 1178
ababa bbbaa baaab aaaaa aabbb bbbbb baaba babaa babab bbaba
abbbb babbb bbbaa abbaa bbbaa babab bbbab aabba abbba bbbaa
babba abbaa abaab babab abbbb aaabb bbaab babab abbaa bbabb
bbbbb bbaab aaaba baabb abbba aaaab babaa aab
ECGTGT88V {0} ; 1179
ababa bbbaa baaab aaaaa aabbb bbbbb baaba babaa babab bbaba
abbbb babbb bbbaa abbaa bbbaa bbbab bbbab aabba abbba bbbaa
babba abbaa abaab babab abbbb aaabb bbaab babab abbaa bbabb
bbbbb bbaab aaaba baabb abbba aaaab babaa aab
ECGTGT91G {0} ; 1180
ababa bbbaa baaab aaaaa aabbb babbb baaba babaa babab bbaba
abbbb babbb bbbaa abbaa bbbaa babab bbbab aabba abbba bbbaa

```
 baaba abbaa abaab babab abbbb aaabb bbaab babab abbaa bbabb
 bbbbb bbaab aaaba baabb abbba aaaab babaa aab
EAGTCA162G          {0} ; 1291
 ababa bbbaa baaab aaaaa aabbb bbbbb baaaa babaa babab bbaba
 abbbb babbb bbbaa abbaa bbbaa babab bbbab aabba ababa bbbaa
 babba abbaa abaab babab abbb- aaabb bbaab babab abbaa bbabb
 bbbbb bbaab aaaba baabb abbba aaaab babaa aab
EACTGA117G          {0} ; 1351
 ababa bbabb bbabb abaaa aabbb aabbb aaaaa babab bbaab abbba
 abba- baaab bbbbb aa-aa bbbaa b-bbb aabba aabba aaaaa bbbab
 baaba abbaa aaaab babab aabbb aaabb babbb babab abbbb bbabb
 bbabb aaaab aabba baabb abbaa babbb baabb bab
EAGTGC111G          {0} ; 1390
 abaaa bbabb bbabb abbaa babab aaaab bbaaa babbb bbaab abbab
 abba- babab bbaab aa-ab baaab b-bbb aaaaa aabab aaaaa bbbab
 bbabb abaaa baaaa aabab babbb bbbbb aaabb baaba abbbb baabb
 bbaba aaabb aabaa baabb bbbba baaab babbb bab
EACTTC156V          {0} ; 1451
 abaaa bb-bb bbabb -abaa babab aaabb abab- babbb bbaab abbaa
 abbaa baabb bbbab aabaa bb--a ---bb aaaaa aabab aaaaa bbbbb
 babbb abaaa baaab babab babbb baabb aaabb baaba abbbb baabb
 bbabb aaabb aabaa baabb a-baa babbb baabb aab
SSR_781             {0} ; 1596
 ababa bbaaa baaab aaaaa aabbb bbbbb baaba babaa babab baaba
 abbbb baaab bbbaa abbab bbbaa babab abbba aabba abaaa bbbab
 babba abaaa abbab babab abbbb aaabb bbaab babab abbab bbabb
 bbbbb bbaab abab- aaabb abbba aaaab baaaa aab

LG25:
EGAMGA526G          {0} ; 43
 ababa abbbb baaab babbb babaa abbbb bbbaa aabbb bbabb abbaa
 baabb aabba ababa bbabb baaaa abbab babba aabaa abba- -----
 ----- ----- ----- ----- ----- ----- ----- ----- ----- -----
 ----- ----- ----- ----- ----- ----- ----- ---
ECGMCG290G          {0} ; 94
 aaaba abbba baaab babbb baaaa abbbb abbaa babbb bbaba abbaa
 bbabb aabba ababb bbbbb baaaa abbaa babba aabab abaa- -----
 ----- ----- ----- ----- ----- ----- ----- ----- ----- -----
 ----- ----- ----- ----- ----- ----- ----- ---
ECGMCG293V          {0} ; 95
 aaaba abbba baaab babbb baaaa abbbb abbaa babbb bbaba abbaa
 bbabb aabba ababb bbbbb baaaa abbaa bbbba aabab abaa- -----
 ----- ----- ----- ----- ----- ----- ----- ----- ----- -----
 ----- ----- ----- ----- ----- ----- ----- ---
ECAMCC115G          {0} ; 142
 abbba bbaba baaab abbaa babaa abbab ababa bbaaa baabb abbab
 baabb aabbb abaab baaaa abbbb bbbab bbbbb baaab -baaa baabb
 babba bbabb abbbb bbbbb bbbbb abaab bbaab babba aaaba bbbab
 ababb baabb ababb baaba abbbb baaab bbbab aaa
ECAMCC271V          {0} ; 161
 abbaa bbabb baabb abbab aabaa aabab abbba bbaab bbabb abbaa
 aaabb aabbb abaab baaaa aabba abbab bbbbb baaab -bbaa baabb
 babaa abaab abbbb baabb abbbb aaaab bbabb babba aaaba abbab
 aaabb aabba ababb babab abbbb baaab bbbaa aaa
EACMCC89G           {0} ; 195
 ababa abbbb baaab aabab babaa abbbb bbbaa aabbb bbabb abbaa
 baabb aabbb babab aabbb baaaa abbab bbbbb aabab abbaa baabb
```

```
 babbb aabab ababb ababa abbab aaaab babab baaba aaaaa abaaa
 aaaaa aabba bbabb aaaaa abbbb baaab baaaa baa
EACMCC454G          {0} ; 231
 bbbbb bbbbb babbb abbab abbab babab baaba bbaab bbabb abbaa
 aaabb aabbb baaab baaaa aabba abbab bbbbb baaab bbbaa baabb
 babaa bbaab abbbb babba abbbb abaab bbabb babba aaaba abbab
 aaabb babbb ababb baaaa abbbb baaab bbbaa aaa
EACMGA164G          {0} ; 345
 abaaa bbabb baabb abbab aabaa aabab abbba bbabb bbabb abbaa
 aaabb aabbb ababb baaaa aabbb abbab bbbbb baaab bbbaa baabb
 baba- abaab abbbb baaba abbbb aaaab bbabb babba aaaaa abbab
 aaabb aabbb ababb baaaa abbbb baaab bbbaa aaa
EAGMTA104G          {0} ; 395
 ababa abbbb baaab aabab babaa abbbb bbbaa aabbb bbabb abaaa
 baabb aabbb aaaba b-abb baaaa abbab bbbbb aabab abbaa aaabb
 babb- aabab ababb ababa abbab aaaab babab baaba aaaaa abaaa
 aaaaa aabba bbabb aaaaa abbbb baaab baaaa baa
EAGMTA429G          {0} ; 422
 aaaba abbba baaab babbb baaaa abbbb abbaa babbb bbabb abbaa
 bbabb aabba ababb b-bbb baaaa abbaa babba aabab abbaa aaabb
 bbbb- aabaa abaaa abbaa abbaa abaab babab ababb aaaab bbaab
 aaaaa aabab bbbbb aaaaa bbbbb abaaa baaab bba
EAGMTA504G          {0} ; 425
 aaaba abbba baaab babbb baaaa abbbb abbaa babbb bbabb abbaa
 bbabb aabba ababb b-bbb baaaa abbaa babba aabab abbaa aaabb
 bbbb- aabaa abbaa abbaa abbaa abaab aabab ababb aaaab bbaaa
 baaaa aabab bbbbb aaaab bbbbb aaaba bbaab bbb
EACMTC188G          {0} ; 470
 ababa bbabb baabb abbab aabaa aaaab abbaa bbaab bbabb abbaa
 aaabb aabbb abaab baaaa aabb- abbab bbbbb baaaa bbbaa baabb
 babaa abaab abbbb baaba abbbb aaaab bbabb babba aaaaa abbab
 aaabb aabbb ababb baaaa abbbb baaab bbbaa aaa
EACMTC190V          {0} ; 471
 ababa bbabb baabb abbab aabaa aaaab abbba bbaab bbabb abbaa
 aaabb aabbb abaab baaaa aabb- abbab bbbbb baaab bbbaa baabb
 babaa abaab abbbb baaba abbbb aaaab bbabb babba aaaaa abbab
 aaabb aabbb ababb babba abbbb baaab bbbaa aaa
EAGMCT235V          {0} ; 821
 aaaba abbba baabb bbbbb baaaa abbbb babaa babbb bbabb a-baa
 bbabb aabba ababb bbbbb baaaa abbaa babba aabab abbaa aaabb
 babba aabaa abaaa abbaa abbaa abaab a-bab aaabb aaaab abaaa
 aaaaa aabab bbbbb aabba bbbbb aaaaa baaab baa
EAGMCT407V          {0} ; 840
 ababa bbaba baabb aabab aabaa aabab baaba bbaab bbabb a-baa
 aaabb aabbb abaab baaaa aabba abbab bbbbb baaab bbbaa baabb
 babaa bbaab abbbb babba abbbb abaab b-abb babba aaaba abbab
 aaabb babbb aaabb babba abbbb baaab bbbaa aaa
EACTCC450G          {0} ; 1150
 ababa bbbbb baaab aabab babaa abbbb bbbaa aabbb bbabb abbaa
 babab aabbb ababa bbabb baaaa abbab bbbbb aabaa abbaa baabb
 baabb aabab ababb ababa abbab aaaab babab baaba aaaaa abaaa
 aaaaa aabba bbabb aaaaa abbbb baaab baaaa baa
EACTAG119V          {0} ; 1233
 ababa abbbb baaab aabab babaa abbbb bbbaa aabbb bbabb abbaa
 babab aabbb ababa bbabb baaaa abbba bbbbb aabab abbaa baabb
 bbbbb aabab ababb ababa abba- aaaab babab baaba aaaaa abaaa
 aaaaa aabba bbabb aabaa abbbb baaab baaaa baa
```

```
EACTAG284V           {0} ; 1256
 ababa bbabb baabb abbab aabaa aabab abbba bbabb bbabb abbaa
 aabab aabbb abaab baaaa aabbb abbba bbbbb baaab bbbaa baabb
 bbbaa abaab abbbb baaba abbb- aaaab bbabb babba aaaaa abbab
 aaabb aabbb ababb babaa abbbb baaab bbbaa aaa
EAGTCA287G           {0} ; 1306
 aaaba abbbb baaab babbb babaa abbbb bbbaa aabbb bbabb abbaa
 babab aabba ababa bbbbb baaaa abbab bbbba aabab abbaa baabb
 babba aabaa ababa ababa abba- aaaab babab aaaba aaaab abaaa
 aaaaa aabbb bbbbb aaaaa bbbbb aaaab baaaa baa
EACTTC83V            {0} ; 1442
 ababa ab-bb baabb -abab babaa aabbb bbba- abbbb bbabb abbaa
 babab aabbb ababa bbabb ba--b ---ab bbbbb aabab bbbaa baabb
 babab aabab abbbb aaaba abbbb aaaab baaab babba aaaaa abbaa
 aaaba aabbb bbabb aabaa a-bbb baaab bbaaa baa
EACTTC490V           {0} ; 1486
 ababa bb-bb baabb -abab aabaa aabab abbb- bbaab bbabb abbaa
 aabab aabbb abaab baaaa aa--a ---ab bbbbb baaab bbbaa baabb
 babaa abaab abbbb baaba abbbb aaaab bbabb babba aaaaa abbab
 aaabb aabbb ababb babaa a-bbb baaab bbbaa aaa
EACTTC493G           {0} ; 1487
 ababa bb-bb baabb -abab aabaa aabab abbb- abaab bbabb abbaa
 aabab aabbb abaab baaaa aa--a ---ab bbbbb baaab bbbaa baabb
 baaaa abaab abbbb baaba abbbb aaaab bbabb babba aaaaa abbab
 aaabb aabbb ababb baaaa a-bbb baaab bbbaa aaa
SSR_598              {0} ; 1586
 ababa bbaba baabb abbab aabaa aabab ababa bbaab bbabb aabaa
 aaabb aabbb abaab babbb babba abbab bbbbb baaab bbbaa baabb
 babaa abaab abbbb babba abbbb abbbb bbabb babba aaaba abbab
 aaabb babbb abaa- babaa abaab baaab bbbaa aa-

LG26:
ECAMCC140V           {0} ; 144
 bbbbb aabab aabbb bbabb bbbba bbbba aabba babbb bbabb aabbb
 abbaa bbaab bbbab abbaa bbaba baaba abbbb aabab -bbba abaaa
 babbb baaab bbabb abbbb bbbba abaaa abaaa baaaa aaaaa baaaa
 aaabb aabaa abbba aaaab bbbab abbba aaaba baa
EACMGA445G           {0} ; 379
 bbbbb abbbb aabba babab baabb aaabb babba bbaab bbaba abbab
 baaab abbaa bbaba aaaaa ababb aaaaa babbb aabbb babaa baaab
 aabb- bbabb baabb bbabb bbabb bbbaa ababa baaaa babaa babbb
 ababb babab ababa aaaba abbba abaaa babab aab
EACMTC103G           {0} ; 452
 bbbba abbab babba babaa aaaab aabbb abaab aaaaa baaba abaaa
 bbbab abbbb baabb baaaa bbba- babba bbbbb baaba babab baaba
 baaaa baaab baaaa bbaab bbaab abbba bbbaa aabaa babba babab
 bbaba aabaa abaaa aabab ababb abaab baaab aab
ECAMCT54G            {0} ; 536
 bbbba ababb aabba babab baabb aaabb bbaaa bbaaa baaba abbab
 babab abbbb bbaba aaaaa bbaa- aaaba babbb aabbb babab baabb
 aaaaa bbaab baaab baaab aaabb abbaa bbbaa aaaaa babaa babbb
 ababb aabaa ababa aabab abbba abaaa baaab aab
ECAMCT218V           {0} ; 566
 bbbba ababb aabbb babab aaaab aabbb bbaaa bbaaa baaba ababb
 babab abbbb bbaba aaaaa bbaa- aaaba babbb aabba bbbbb baabb
 aaaba abbba baaaa abaab bbabb abbba bbbba aabaa bbbaa babbb
 abbbb aabaa bbaaa babbb bbbba bbaaa baabb bbb
```

```
ECGMTT187V           {0} ; 604
 bbbba ababb aab-a babab bbabb aaabb bbbba bbaab bbaba abbab
 babab abbba bbaba aaaaa bba-- aaaaa babba aabbb babab baaab
 aabaa bbaab baaab baaab aaabb bbbaa bbbaa aaaaa babaa babbb
 ababb aabaa ababa abbbb abbba abaaa baaab aab
EACMCG182G           {0} ; 637
 bbbba aabab aabbb bbaab bbbba aaaba ababa baabb bbbab aabbb
 aaaaa bbaaa bbbab aabaa bbaba aabba abbba aabaa bbbba abaaa
 baaba bbabb baaba ababb bbaaa abaaa ababa baaaa abbba babab
 aaabb abbab abbba aaaba bbbbb abbba babba aba
EACMCG185V           {0} ; 638
 bbbba aabab aabbb bbaab bbbba aaaba ababa baabb bbbab aabbb
 aaaaa bbaaa bbbab aabaa bbaba aabba bbbba aabab bbbba abaaa
 baaba bbabb baaba ababb bbaaa abaaa ababa baaaa abbba babab
 aaabb abbab abbbb aaabb bbbbb abbba babba aba
EACMCG530G           {0} ; 664
 bbbaa aabba babba aaaaa aaaaa aaabb baaab aaaaa baabb abaaa
 bbbab abbbb baabb baaaa bbbab bbaba bbbbb baaba bbbab baaba
 aaaaa baaab baaab bbbab bbabb abbba bbbaa babbb babba babab
 ababb aabaa abaaa aabba ababb abbab baaab bab
EACMGG205G           {0} ; 759
 bbbba ababb aabbb bbaab baabb aaaba babba baabb bbaba a-bbb
 aaaaa abaaa bbabb aaaaa bbaba aaaaa bbbba aabab babaa bbaab
 aaaba bbabb baabb bbabb bbaba abaaa a-aba baaaa bbbba babab
 ababb bbbab abbba aaaab bbbbb abbba babaa abb
EAGMCT179V           {0} ; 814
 bbbba ababb babba babab baaab aaabb bbaaa bbaaa baaba a-aab
 babab abbbb bbaba aaaaa bbaab aaaba babbb aabbb babab baaba
 aaaaa bbaab baaab baaab ababb abbba b-baa aaaaa babaa bbbbb
 ababb aabaa abaaa aabbb abbba abaaa baaab aab
EAGMCT198G           {0} ; 816
 bbbba abbab babba babaa abaab aaabb bbaab babab bbaba a-aaa
 bbbab bbbbb aabbb baaaa bbbbb babba abbbb bbaba aabab baaba
 baaaa baaaa baaaa abaab bbaab abbba b-aaa aabaa aabba bbaab
 bbaba aabaa aaaba abbab aaabb abaaa baaab aab
EAGMCT240G           {0} ; 822
 bbbba ababb aabba bbbab baabb aaabb abbba bbaab bbaba a-bbb
 baaab abbaa bbaba aaaaa bbabb aaaaa aabbb aabba babaa baaab
 aabba bbabb baabb bbabb bbabb bbbaa a-aba baaaa babaa babbb
 ababb bbbab abbba aaaab abbbb abaaa babab aab
EACMGC245G           {0} ; 887
 abbba abbbb baaaa babaa aaaab aaabb abaab aaaaa baaba abaaa
 bbbab abbbb baaba baaaa bbaab babba bbbbb baabb babab baaba
 baaaa baaab baaab bbaab bbabb bbbba bbbba aabaa aaabb babbb
 babaa bbaaa babbb abbaa ababa babba abaaa bab
EAGMTT213V           {0} ; 1063
 bbbba ababb aabbb baabb baabb aaaba babba bbaab bbaba abbbb
 aaaab abaaa bbaba aaaaa bbaba aaaaa bbbba aabbb babaa baaab
 aabba bbabb baabb bbabb bbabb abbaa ababa baaaa bbbaa babab
 ababb bbbab abbba aaabb abbbb abbba babaa abb
EAGMTT214G           {0} ; 1064
 bbbba ababb aabbb baabb baabb aaaba babba bbaab bbaba abbbb
 aaaab abaaa bbaba aaaaa bbaba aaaaa bbbba aabbb babaa baaab
 aabba bbabb baabb bbabb bbabb abbaa ababa baaaa bbbaa babab
 ababb bbbab abbba aaaba abbbb abbba babaa abb
EAGMTT394V           {0} ; 1075
 bbbba ababb aabba babbb baabb aaabb bbbba bbaab bbaba abbab
```

```
 baaab abbba bbaba aaaaa bbabb aaaaa babba aabbb babab baaab
 aabaa bbaab baaab baaab aaabb bbbaa bbbaa baaaa babaa babbb
 ababb babaa ababa abbbb abbba abaaa baaab aab
EACTCC262V          {0} ; 1131
 bbbba abbbb babba babaa aaaab aaabb ababb aaaaa baaba abaaa
 bbabb abbbb baabb baaaa bbaab babba bbbbb baabb babab baaba
 babaa baaab baaaa bbaab bbabb abbba bbbaa aabaa babba babab
 bbabb aabaa abaaa aabbb abbbb abaab baaab aab
EACTGA268bV         {0} ; 1370
 bbbba abbbb babba babaa aaaab aaabb abaab aaaaa baaba abaaa
 bbab- abbba baaba ba-aa bbaab b-bba bbbbb baabb babab baaba
 babaa baaab baaab bbaab bbabb abbba abbaa aabaa baaba babab
 ababb aabaa abaaa aabbb abbbb abaab baaaa aab
EAGTGC168V          {0} ; 1393
 bbbba ababb aabba babab baabb aaabb babba bbaab bbaba abbab
 baaa- abbaa bbaba aa-aa bbabb a-aaa babbb aabbb babaa baaab
 aabba bbabb baabb bbabb bbabb bbbaa ababa baaaa babaa babbb
 ababb bbbab ababa ababb abbba ababa babab aab
EAGTGC426V          {0} ; 1411
 bbbba abbbb babba babab baaab aaaab bbaaa bbaaa baaba abaab
 baab- abbbb bbaba aa-aa bbaab a-aba babbb aabbb babab baaba
 aabaa bbaab baaab baaab ababb abbba bbbaa aaaaa babaa babbb
 ababa aabaa abaaa aabbb abbba abaaa baaab abb
ECGTGA411G          {0} ; 1561
 bbbba ab-bb aabbb -baab baabb aaaba babb- baabb bbaba abbbb
 aaaaa abaaa bbabb aaaaa bb--a ---aa bbbba aabab baaaa bbaab
 aaaba bbabb baabb bbabb bbaba abaaa ababa baaaa bbbba babab
 ababb bbbab abbba aabab b-bbb abbba babaa abb

LG27:
EACMCC354V          {0} ; 224
 aabbb aaaab ababa baaab aabba aaabb abbba aaabb aaaab aabab
 abbbb bbabb aaabb aabaa bbbaa bbbaa aaaab abaab bbaaa babbb
 bbaab aabab babbb bbbba babba baaab babab babba bbaba baaab
 bbbbb aaaba abbba babab abaab babba aabbb baa
ECAMGG338V          {0} ; 273
 babbb aaabb ababa baaab abbba aaaba abbba aaaab aaaab aabab
 abbbb bbabb aaabb aabaa bbbaa bbbaa aaaab abaab bbaaa bbbbb
 bbaab aabab babbb bbbba babba baaab babab baaba bbaba baaab
 bbbbb aaaba abbba babab abaab babba aabbb baa
ECAMGG444V          {0} ; 289
 bbbbb aaabb ababb baaab aabba aaaba abbaa aaaab aaaaa aabab
 aaabb bbabb aaabb aabaa bbbaa abbaa aaaab abaab bbbaa babba
 bbaab aabab babbb bbbba baaba babab babab baaba ababa baaab
 bbbbb aaaaa abbba babab abaab bbbba aabbb baa
ECGMTA288V          {0} ; 317
 aabba aaaba ababa abaaa abaaa ababb abbab aaaab aabab aaaba
 babbb babab aaaab baaba bbaab abbbb babaa abbbb abbbb babaa
 babaa aaaaa abbbb babab aaaab abbba abaab bbbab aabaa aaabb
 bbaaa abaab aaabb bbaab abbbb bbaab babab aba
EACMGA278V          {0} ; 360
 aabbb aaaab ababa baaab aabba aaabb babba aaabb aaaab aabab
 abbbb bbabb aabaa bbaaa bbbaa bbbaa aaaab abaab bbaaa babbb
 bbaa- aabab babbb bbbba babba baaab babab baaba bbaba baaab
 bbbbb aaabb abbba babab abaab babba aabbb baa
EACMGA346G          {0} ; 368
 babbb aaabb aaaaa bbaaa aaabb ababa bbbaa aaaab aaaab aaaaa
```

```
 aaabb bbbbb aabab bbaaa bbaab abaab babab abbab abbaa babbb
 baaa- baaaa babba babba aaabb bbaba baabb bbbbb aabba abaab
 abbbb aaaab abbbb baaaa bbaba bbabb babab bba
EAGMTA289V           {0} ; 412
 babbb aaaba ababa bbaab aaabb ababa bbbba aabab aaaab aaaaa
 aabbb bbbbb aabab b-aaa bbaab ababb babab abbab abbaa babab
 baaa- baaaa babba babba aaabb bbaba baaab bbbab aabba abaab
 abbbb aaaab aaaab baaba bbabb bbbbb babab bbb
EACMTC172V           {0} ; 466
 babbb aaabb ababa bbaab aabbb abbba bbbaa aaaab aaaab aabab
 abbbb bbbbb aabaa bbaaa bbba- bbaab babab abaab abbaa babbb
 baaab aabab babba babba aaaba bbaba babab baaaa aaaba baaab
 abbbb aaaaa abbba baaba ababa bbbbb aabbb bab
EACMTC172G           {0} ; 467
 babbb aaabb ababa baaab aabbb abbba bbbaa aaaab aaaab aabab
 abbbb bbbbb aabaa bbaaa bbba- bbaab babab abaab abbaa babbb
 baaab aabab babba babba aaaba bbaba babab baaaa aaaba baaab
 abbbb aaaaa abbba baaaa ababa bbbbb aabbb bab
EACMTC197V           {0} ; 474
 babbb aaaba ababa bbaab aaabb abbba bbbba aaaab aaaab aaaba
 babbb bbbab aabab bbaaa bbaa- abaab babab abbab abbaa aabbb
 baaaa baaaa babba babba aaabb bbaba baaab bbbab aabba abaab
 abbbb aaaab abbbb baaba bbabb bbbbb babbb abb
EACMTC310G           {0} ; 494
 babbb aaaba ababa bbaab aaabb abbba bbbaa aaaab aaaab aaaba
 babbb bbbab aabab bbaaa bbaa- abaab babab abbaa abbaa aabbb
 baaaa baaaa babba babba aaabb bbaba baaab bbbab aabba abaab
 abbbb aaaab abbbb baaaa bbabb bbbbb babab bbb
ECGMTT226V           {0} ; 610
 babba aaaba baa-a abaaa abaaa ababb abbab aaaab aabab aaaba
 babab babab aaaab babab bba-- abbbb babaa abbbb aaaba aaaab
 baaaa aaaaa ababb babab aaaab abbba abbab babab aabba abaab
 baaba abaab abbbb babab abbbb bbaab babab aba
EAGMCT202V           {0} ; 817
 babbb aaabb ababa baaab aabba aaaba abbaa aaabb aaaab a-bab
 abbbb bbabb aabaa bbaaa bbbaa bbbaa aaaab abaab bbaaa babbb
 bbaab aabab babbb bbbbb babba baaab b-bab baaba bbaba baaab
 bbbbb aaaba abbba babba abaab babba aabbb baa
EAGMCA480G           {0} ; 971
 aabbb aaaab abbaa baaab aabba aaabb abbaa aaabb aaaab aabab
 abbbb bbabb abbaa bbaaa bbbaa bbbaa aaaab abaab bbaaa babbb
 bbaab aabab babbb bbbba babba baaab babab babba ababa aabaa
 bbabb aaabb bbaab babaa aaabb bbbba aabaa bbb
ECGMCA254V           {0} ; 989
 aabbb aaaab abbaa baaab aabba aaabb babaa aaabb aaaab aabab
 abbbb bbabb abbaa bbaaa bbbaa bbbaa aaaab abaab bbaaa babbb
 bbaab aabab babbb bbbba babba baaab babab babba ababa aabaa
 bbabb aaabb bbaab babab aaabb bbbba aabaa bbb
ECGMCA390V           {0} ; 996
 babbb aaabb abbaa baaab aaabb ababa bbbba aaaab aaaab aabaa
 abbbb bbbbb aabab bbaaa bbbab abaab babab abaab abbaa babbb
 baaaa aaaab babba babba aaabb bbaba baaab bbaab abaaa aabab
 abbbb bbabb bbbba aabab baaba bbbba aaaaa bbb
EAGMTT331V           {0} ; 1069
 aabba aaaba aaaaa abaaa abaaa aaaab babab aaaab aabab aaaba
 babbb babab aaaab baaaa bbaab abaab babaa abbbb abbba bbaaa
 babaa aaaaa abbbb babab aaaab abbba abaab bbbab aabaa aaabb
```

```
 bbaaa abaab aaabb bbaab abbbb bbaab babab aba
EAGTTA93G           {0} ; 1158
 babbb aaabb ababa baaab aabbb ababa bbbaa aaaab aaaab aabab
 abbbb bbbbb aabaa bbaaa bbbab bbbab aabab abaab abbaa babbb
 baaab aabab babba bbbba baaba bbabb babab baaba aaaba baaab
 abbbb aaaaa abbba baaaa abaaa bbbbb aabbb bab
EAGTTA95V           {0} ; 1159
 babbb aaabb ababa baaab aabbb ababa bbbba aaaab aaaab aabab
 abbbb bbbbb aabaa bbaaa bbbab bbbab aabab abaab abbaa babbb
 babab aabab babba bbbba baaba bbabb babab baaba aaaba baaab
 abbbb aaaaa abbba baaaa abaaa bbbbb aabbb bab
EAGTCA180G          {0} ; 1296
 babbb aaabb abaaa baaab aabbb ababa bbbaa aaaab aaaab aabaa
 abbbb bbbbb aabab bbaaa bbbab bbaab babab abaab abbaa babbb
 baaaa aabab babba babba aaab- bbaba baaab baaab aaaba bbaab
 abbbb aaaaa abbba baaaa ababa bbbab babbb bab
EAGTAA171V          {0} ; 1507
 babba aa-ba ababa -abaa abab- ababb baba- aaa-b aa-ab a-aba
 -abbb babab a-aab baaab bb--b ----a bab-b -b--- ----a babba
 babaa aaaaa abbbb babab aaabb bbbba abaab bbbab aabba ababb
 bbaba abaab aaabb b--a- a-bbb bbaab babab aba
EAGTAA176G          {0} ; 1508
 babba aa-ba ababa -aaaa abab- abaab baba- aaa-b aa-ab a-aba
 -aaab babaa a-aab baaaa bb--a ----a baa-a -b--- ----a aabab
 baaaa aaaaa abbbb baaab ababb bbbba abaab bbbab aabba abaab
 baaba abaab abbbb b--a- b-bbb bbbbb babab bba

LG28:
ECGMCG38V           {0} ; 89
 abbaa abaaa aabaa aabbb abbab aaaab abaaa bbabb abbaa abaaa
 bbbba bbbbb babba bbbab baaaa baaba abbab bbaab abba- -----
 ----- ----- ----- ----- ----- ----- ----- ----- ----- -----
 ----- ----- ----- ----- ----- ----- ----- ---
EGAMGC49G           {0} ; 102
 ababa abaaa aaaaa abbbb aabbb abaab abbab bbabb abbbb abbba
 baaba ababa babba aaaab babaa baabb aaaab baaab babaa bbbab
 aaaaa baabb aaaba bbbba aaabb ababb babbb baaaa baaab aabba
 bbbba bbaba bbaba aaaaa bbbaa bbbab ababa aaa
EGAMGC265V          {0} ; 123
 baaba abaaa baabb aabbb aabab bbaaa abbab baaab abbbb abbaa
 aaaab aaaba bbbaa aaaaa bbbba bbbbb aaabb aaaaa babba bbbbb
 abaaa bbaab aaabb aaaaa aaaab aabbb bbbbb aaaaa aaabb aabba
 bbaab bbbaa bbbba aabab abbaa aaaab bbaba aaa
ECAMCC222G          {0} ; 152
 ababa abaaa aabaa aabbb abbbb aaaab abaaa bbabb abbba abaaa
 babba abbba babba bbbab baaaa baaba abbab bbaab -abab babab
 aaaaa ababb aaaab babba aaabb ababb babbb baaaa babba abbaa
 ababb bbaba baaba ababa abbaa baaab ababb baa
ECGMTA155G          {0} ; 306
 abbaa abaaa aabaa aabbb abbbb aaaab baaaa bbabb abbaa abaaa
 babba bbbbb abbbb abbba baaaa baaba abbab bbaab abbab babab
 aaaaa ababb aaaab babba aaabb ababb babbb baaba babaa bbbaa
 ababb bbabb bbaba ababa abbaa baaab ababb bba
ECGMTA349V          {0} ; 321
 babaa abaaa bbabb aabbb abbab bbaaa babab aaaab abbbb abbaa
 aaaab aaaba bbaba aaaba bbbba bbbbb aaaab aabba babba bbbbb
 abaaa abaab aaabb aaaaa aaaab aabbb bbbba aaaaa aaaab aabba
```

```
 bbaab bbbbb bbaba aabab abbaa aaaaa bbaba aaa
ECGMTT148V          {0} ; 601
 abbaa abaaa bab-a aabbb abbbb aaaab baaaa bbabb abbaa abaaa
 babba bbbbb babba bbbab baa-- bbaba abbab bbaab abbab baaab
 aaaaa ababb aaaab babba aaabb ababb babbb baaba babaa bbbaa
 ababb bbabb baaba abbba abbaa baaab ababb bba
ECGMTT338G          {0} ; 618
 babaa abaaa baa-b aabbb aabab bbaaa babab baaab abbbb abbaa
 aaaab aaaba bbbaa aaaab bbb-- bbbab aaaab aaaaa babba bbbbb
 abaaa bbaab aaabb aaaaa aaaab aabbb bbbbb aaaaa aaaab aabba
 bbaab bbbab bbbba aaaab abbaa aaaab bbaba aaa
EACMCG386G          {0} ; 659
 bbbaa babab aabaa aabbb abbbb abaab babab baabb abbbb abaaa
 babba abbba babba abaab baaaa baaba aaaab bbaab aaaaa bbbab
 aaaaa bbabb aaabb bbbba aaabb aaabb babab baaaa baaab aabaa
 ababa bbaba baaba ababa abbaa bbaab ababa aaa
EACMGG105V          {0} ; 740
 abbaa abaaa aabaa aabbb abbbb aaaab abaaa bbabb abbaa a-aaa
 babba bbbbb babba bbbab baaaa baaba abbab bbaab abbab babab
 aaaaa ababb aaaab babba aaabb ababb b-bbb baaba babaa bbbaa
 ababb bbabb baaba ababa abbaa baaab ababb bba
EACMGG447V          {0} ; 790
 bbbaa abaaa aaaaa aabbb aabab abaab abbab baabb abbbb a-baa
 aaaba aabba babaa aaaab bbbaa baabb aaaab baaab aabaa bbbab
 aaaaa bbaab aaaba aaaba aaaab abbbb b-bab baaaa aaaab aabba
 bbaab bbbab bbbba aabbb bbbaa bbaab bbaba aaa
EAGMCT400V          {0} ; 838
 abbaa abaaa aabaa aabbb abbab aaaab baaaa bbabb abbaa a-aaa
 bbbba bbbbb babba bbbab baaaa baaba abbab bbaab abbab babab
 aaaaa ababb aaaab babba aaabb ababb b-bbb baaba babaa bbbaa
 ababb bbabb baaba ababa abbaa baaab aaabb bba
EACTCC185G          {0} ; 1127
 abbaa abaaa aabaa aabbb abbab aaaab abaaa bbabb abbaa abaaa
 bbbba bbbbb babba bbbab baaaa baaba abbab bbaab abbab babab
 aaaaa ababb aaaab babba aaabb ababb babbb baaba babaa bbbaa
 ababb bbabb baaba ababa abbaa baaab aaabb bba
EACTCC206V          {0} ; 1129
 abbaa abaaa aaaaa aabbb aabbb abaab abbab baabb abbbb abbaa
 babaa aabba babba aaaab bbbaa baabb aaaab bbaab aabaa bbbab
 aabaa bbaab aaaba aabba aaaab ababb babab baaaa aaaab aabba
 bbaba bbbaa bbbba aaaab bbbaa bbaab bbaba aaa
EAGTCA319V          {0} ; 1311
 abbaa abaaa aabba aabbb abbab aaaab abaaa bbabb abbaa abaaa
 bbbba bbbbb babba bbbab baaaa baaba abbab bbaab abbab babab
 abaaa ababb aaaab babba aaab- ababb babbb baaba babaa bbbaa
 ababb bbabb baaba ababa abbaa baaab aaabb bba
EAGTCA321G          {0} ; 1312
 abbaa abaaa aabba aabbb abbab aaaab abaaa bbabb abbaa abaaa
 bbbba bbbbb babba bbbab baaaa baaba abbab bbaab abbab babab
 aaaaa ababb aaaab babba aaab- ababb babbb baaba babaa bbbaa
 ababb bbabb baaba ababa abbaa baaab aaabb bba
EACTGA145G          {0} ; 1357
 aabaa aaaaa aaaaa aabbb aabbb abaab abbab baabb abbbb abbaa
 baba- aabba babba aa-ab bbbaa b-abb aaaab bbaab aabaa bbbab
 aaaaa bbaab aaaba aabba aaaab ababb babab baaaa aaaab aabba
 bbaba bbbaa bbbba aaaab bbbaa bbaab bbaba aaa
EACTGA224G          {0} ; 1364
```

```
 abbaa abaaa aaaaa aabbb aabbb abaab abbab baabb abbbb abbaa
 baba- aabba babba aa-ab bbbaa b-abb aaaab bbaab aabaa bbbab
 aaaaa bbaab aaaba aabba aaaab ababb babab baaaa aaaab aabbb
 bbaba bbbaa bbbba aaaab bbbaa bbbbb baaba aaa
EACTGA224V           {0} ; 1365
 abbaa abaaa aaaaa aabbb aabbb abaab abbab baabb abbbb abbaa
 baba- aabba babba aa-ab bbbaa b-abb aaaab bbaab aabaa bbbab
 aabaa bbaab aaaba aabba aaaab ababb babab baaaa aaaab aabbb
 bbaba bbbaa bbbba aaaab bbbaa bbbbb baaba aaa
ECGTGA99V            {0} ; 1530
 abbaa ab-aa aabaa -abba abbab aaaab abab- bbabb abbaa abaaa
 bbbba bbbbb babba bbbab ba--a ---ba abbab bbaab abbab babab
 aabaa ababb aaaab babba aaabb ababb babbb babba babaa bbbaa
 ababb bbabb baaba abbaa a-baa baaab aaabb bba
SSR_168              {0} ; 1581
 abbaa abaaa aabaa aabbb abbba bbbab abbab baabb abbbb aaaaa
 babab abbba babba bbaab baaaa baaba aaaab bbaab aabba bbbab
 aaaaa ababb aabbb bbbba aaabb ababb babab baaaa baaab aabaa
 ababa bbaba baab- ababa abbbb bbaab ababa aaa

LG29:
ECGMCC451G           {0} ; 189
 bbaaa ababb bbbab bbaba abbba abbab abaab bbaaa aaaaa babaa
 babaa abbbb ababa abbbb bbabb bbbbb aabab bbaab bbaaa aabab
 bbabb aaa-b bbaba babab bbbbb aabba abaab babbb abbba abbaa
 aabba aabab bbbaa bbbaa baaba abaaa aaabb bab
EACMTC399V           {0} ; 511
 abaaa aaaab babaa bbaba abbba aaaab bbaba abbba aaaab babaa
 babba ababb abaaa aabbb bbba- bbbaa aaabb baaab bbbab aabbb
 ababa aaabb abbba babbb babbb aabba abaab baaba abbaa bbbaa
 aabaa abbaa bbbaa abbbb aaaaa bbaaa abaaa baa
EACMTC399G           {0} ; 512
 abaaa aaaab babaa bbaba abbba aaaab bbaba abbba aaaab babaa
 babba ababb abaaa aabbb bbba- bbbaa aaabb baaab bbbab aabbb
 ababa aaabb abbba babbb babbb aabba abaab baaba abbaa bbbaa
 aabaa abbaa bbbaa abbab aaaaa bbaaa abaaa baa
ECAMCT65V            {0} ; 537
 bbaaa aaaaa baaba baaba aabba aaabb bbaba abbba aaaab babaa
 baaba aaabb aaaab baabb bbba- bbbaa baaba baaab bbbab aabbb
 abaaa aabbb abaaa baabb baabb abbaa bbaab baaba aabaa bbbab
 aaaab abbaa abbba abbbb aaaaa bbaaa bbaaa baa
ECAMCT67G            {0} ; 538
 bbaaa aaaaa baaba baaba aabba aaabb bbaba abbba aaaab babaa
 baaaa aaabb aaaab baabb bbba- babaa baaba baaab bbbab aabbb
 abaaa aabbb abaaa baabb baabb abbaa bbaaa bbaba aabaa bbbab
 aaaab abbaa abbba abbab aaaaa bbaaa bbaaa baa
EACMGG174G           {0} ; 755
 bbaaa abaab bbbaa bbaba abbba abbab bbaab bbbaa aaaaa b-baa
 babaa abbbb ababa aabbb bbabb bbbbb aabab baaaa bbaab aabab
 bbabb ababb ababa babbb bbbbb aabba a-aab baabb abbaa bbbaa
 aaaaa abbaa bbbaa bbaab aaaba abaaa aaabb bab
EAGMCT160G           {0} ; 809
 bbaaa aaaab babaa bbaba abbba aabab bbaab bbbba aaaab b-baa
 babaa abbbb abaaa aabbb bbbab bbbbb aaabb baaaa bbaab aabbb
 ababa ababb ababa babbb babbb aabba a-aab baaba abbaa bbbaa
 aaaaa abbaa bbbaa abaab aaaba abaaa aaaaa bab
EAGMCT168V           {0} ; 812
```

```
 bbaaa aaaab babaa bbaba abbba aabab bbabb bbbba aaaab b-baa
 babba abbbb abaaa aabbb bbbab bbbbb aaabb baaab bbaab aabbb
 ababa ababb ababa babbb babbb aabba a-aab baaba abbaa bbbaa
 aaaaa abbaa bbbaa ababb aaaba abaaa aaaaa bab
EAGMTT199V          {0} ; 1062
 bbbaa abaaa baaba baaba aabba aaabb bbaba abbba aaaab babaa
 baaba aaabb aaaab baabb bbbab bbbaa baaba baaab bbbab bbabb
 abaaa aabbb abbaa baabb baabb abbaa bbaab babba aabaa bbbab
 aaaab abbaa abbba abbab aaaaa bbaaa bbaaa baa
EACTAG210V          {0} ; 1245
 bbaaa ababb bbbbb abbba abbba bbbab abbbb bbaaa aaaba bbbaa
 baaba bbabb ababa abbbb bbabb babbb aabab bbabb baaab aabab
 bbbbb aaaab bbaab aabab bbbb- baaba ababb babbb ababa bbbab
 aabba aabab abbaa bbaab baaba bbaba aaabb bbb
EAGTCA172V          {0} ; 1294
 bbaaa aaaab baabb bbaba aabba aabbb bbaba abbba aabab babaa
 babba aaabb abaaa aaabb bbbab bbbaa aaaba baaab bbbab aabbb
 abaaa baabb abbaa babbb baab- aabba bbaab baaba abbaa bbbaa
 aabab abbaa abbaa abbab aaaaa bbaaa abaaa bba
EACTGA281G          {0} ; 1372
 bbaaa ababb bbbab abbba abbba abbab ababb bbaaa aaaba babaa
 baab- bbabb aaaba ab-bb bbabb b-bbb aabab bbaab baaaa aabab
 bbabb aaabb bbaba aabab bbbbb aaaba ababb babbb ababa bbbab
 aabba aabab abbaa bbaab baaba ababa aaabb bbb
SSR_723             {0} ; 1589
 --aaa aaaaa -aaba -aaba aabba aaa-b --a-a a---a aaaa- -a-aa
 baa-a aaa-- aaaa- -aa-- --bab bbbaa baaba -aaa- ----- aa---
 abaaa babbb abbaa aaabb -aabb a-baa bbaab baaba aabaa ---a-
 aaaa- a---a a--b- bbbab aaa-a baaaa babaa baa
SSR_800             {0} ; 1599
 abaaa abaab bbbbb bbaba abbba ab-ab bbaba bbbaa aa-ab aabaa
 babba aa--- -baba aaa-a b-aba aabba aabaa -aaab bbabb aabab
 bbaaa bbbab abbba baaaa aaabb aabba abaab baabb abaaa bbbaa
 bbaaa abaaa bbba- bbaab aaaba ababa aaabb bab

LG30:
EGAMGC212V          {0} ; 121
 aaaaa babab aabbb bbaab bbbbb aabaa ababb bbaab babbb aaaab
 abbba bbbbb babba abaab aaabb aaaaa baabb aaabb baaab babbb
 ababb baaba babba babaa abaab bbbbb abbba abaab baaba babbb
 bbabb aaaba aaaab bbbbb bbaaa baaab ababb baa
ECAMCC172V          {0} ; 150
 aabaa bbbbb abaab bbaab bbbbb abbaa abbaa bbabb abbbb abbab
 aaaab bbbab aabaa abbab aabba aaaaa aaaba aaabb -aabb baaab
 abaab baaab babab bbbba baaab aabab bbbba bbabb baaba aaaab
 baaab aabba aaaab bbbbb aaaab babbb ababb baa
EACMCC125G          {0} ; 198
 aaaba abbbb abaab bbaab bbbab abbaa babab bbaab abbbb abbab
 aaaab bbbab aaaba abbba aabba aaaaa aaaba baabb baabb baaab
 abaab baaab babab bbbba bbaab aabbb bbbba bbabb baaaa aaaab
 baaab aabbb aaaab bbbba aaaab babbb ababb baa
EACMCC126V          {0} ; 199
 aaaba abbbb abaab bbaab bbbab abbaa babab bbaab abbbb abbab
 aaaab bbbab aaaba abbba aabba aaaaa aaaba baabb baabb baaab
 abaab baaab babab bbbba bbaab aabbb bbbba bbabb baaaa aaaab
 baaab aabbb aaaab bbbbb aaaab babbb ababb baa
EACMGA159G          {0} ; 343
```

```
 aaaaa bbbab abaab bbaab bbbab abbaa abbab bbaab bbbbb aaaab
 aaaaa bbbab babba abbab aaaba aaaaa aaaba aaabb baaab baabb
 abaa- baaba babab bbbba bbaab abbbb abbba ababb baaaa aabbb
 baabb aabbb aaaab bbbba aaaab baaab ababb baa
EACMTC141V          {0} ; 460
 ababa abbbb ababb bbaab bbbab abaaa abbab bbaab abbbb abbab
 aaaab bbbab aabaa abbab aabb- aaaaa aaaba baabb baabb baaab
 abaab baaab baaab bbbbb bbaab aabbb bbbba baabb baaaa aaaab
 baaab aabbb aaaab bbbba aaaab babbb ababb baa
EACMTC504V          {0} ; 525
 aaaba abaab abaab bbaab bbbab abaaa abbab bbaab abbbb abbab
 aaaab bbbab aabaa abbab aabb- aaaaa aaaba baabb baabb baaab
 abaab baaab babab bbbba abaab aabbb bbbba bbabb baaaa aaaab
 baaab aabbb aaaab abbba aaaab babbb ababb baa
EACMCG246G          {0} ; 645
 aaaba bbbab aaaab bbaab bbbab abbaa babab bbaab bbbbb aaaab
 aabba bbbab babba abbab aaaba aaaaa aaaba aaabb baaab baabb
 ababb baaba babab bbbaa abaab bbbbb abbba ababb baaaa babbb
 baabb aabbb aaaab bbbba baaab baaab ababb baa
EAGMGG224G          {0} ; 685
 abaaa abbab bbaab ababb abbab aabab abbab baaab abbaa abaab
 aaaab abbaa aabba abbab aaaba aabab aaaba baabb baaba baaab
 abbbb baaab baaab bbaaa bbaab abbbb bbbba bbabb baaaa babab
 bbaba aabbb aaaba bbaba aabaa babba ababb bab
EACMGG46G           {0} ; 731
 abaaa abbab bbaab ababb abbab aabab abaab baaab abbaa a-aab
 aaaab abbaa aabba abbaa aaaba babbb aaaba baabb baaba baaab
 abbbb baaab baaab bbaaa bbaab abbbb b-baa bbabb bbaaa babab
 bbaba babbb aaaba bbaaa aabaa babbb ababb bab
EACMGG426V          {0} ; 787
 aaaba abbbb abaab bbaab bbbab abbaa abbab bbaab abbbb a-bab
 aaaab bbbab aabaa abbab aabba aaaaa aaaba baabb baabb baaab
 abaab baaab babab bbbba bbaab aabbb b-bba bbabb baaaa aaabb
 bbbbb aabbb aaaab bbbba aaaab babbb ababb baa
EACMGG426G          {0} ; 788
 aaaba abbbb abaab bbaab bbbab abbaa abbab bbaab abbbb a-bab
 aaaab bbbab aabaa abbab aabba aaaaa aaaba baabb baabb baaab
 abaab baaab babab bbbba bbaab aabbb b-bba bbabb baaaa aaabb
 bbbbb aabbb aaaab bbbaa aaaab babbb ababb baa
ECGMGT200G          {0} ; 854
 aaaaa bbaaa aaaab bbaab baaab abbaa babab bbaab bbbbb a-aab
 aaaaa bbbab babba abbab aaaba aaaaa aaaba aaaba baaab aaaab
 aaaaa baaba babab bbbba bbaab abbbb a-bba ababb baaaa aabbb
 baabb aabbb aaaab bbbaa aaaab baaab ababb baa
EAGMCA80G           {0} ; 934
 abaaa abbab bbaab ababb abbab aabab babab baaab abbaa abaab
 aaaab abbaa aabba abbaa aaaba babab aaaba baabb baaba baaab
 abbbb baaab baaab bbaaa bbaab abbbb bbbaa bbabb abbab aaaba
 ababb aabab bbabb aaaaa ababb babaa abbba aab
EACMGT272V          {0} ; 1026
 abaaa abbab bbaab ababb abbab aabab abbab baaab abbaa abaab
 aaaab bbbaa aabba abbaa aaaba babab aaaba baabb baaba baaab
 abbbb baaab baaab bbaaa bbaab abbbb bbbaa bbabb baaaa babab
 bbaba aabbb aaaba bbabb aabaa babba ababb bab
ECGTGT144G          {0} ; 1188
 aaaba abbbb abaab bbaab bbbab abbaa abbab bbaab abbbb abbab
 aaabb bbbab abaaa abbbb aabba abaaa aaaba baabb baabb baaab
```

```
 abaab baaab babab bbbba bbabb aabbb bbbba bbabb baaaa aaaab
 baaab aabbb aaabb bbbba aaaab babab ababb baa
SSR_1092            {0} ; 1613
 aaaaa babab aaaab bbaab bbbaa babaa abbab bbaab babbb abaab
 aabba bbbab babba abbaa aaaba aaaaa aaaba aaabb baaab baabb
 ababb babba babab bbbaa abaab bbbbb abaaa abaab baaaa babbb
 baabb aaabb aaaa- bbbba baaab baaab ababb baa

LG31:
ECAMCG208V          {0} ; 69
 aaaba abbba baaaa aabaa abbaa bbaab babba babaa abbaa aaaaa
 aaabb babab babaa aaaaa bbbbb baaab bbaaa baabb abaa- -----
 ----- ----- ----- ----- ----- ----- ----- ----- ----- -----
 ----- ----- ----- ----- ----- ----- ----- ---
ECAMCG221G          {0} ; 70
 aaaba abbba baaaa aabaa abbaa bbaab babaa babaa abbaa aaaaa
 aaabb babab babaa aaaaa bbbbb baaab baaaa baaba abaa- -----
 ----- ----- ----- ----- ----- ----- ----- ----- ----- -----
 ----- ----- ----- ----- ----- ----- ----- ---
EACMCC393V          {0} ; 227
 baaaa abbba baaaa aaaaa abbaa abbab aabab babaa bbbaa baaaa
 aaaba aabab ababa aaaaa babab abaab bbaaa aaabb bbaab babba
 baaaa abbaa bbaab abbba abbaa aaabb baaaa aaaab bbaba bbaaa
 baaab aaaab bbbaa bbbbb abbbb baaaa babaa baa
EACMCC400G          {0} ; 228
 baaaa abbba baaaa aaaaa abbaa abbab aabab babaa bbbaa baaaa
 aaaba aabab ababa aaaaa babab abaab bbaaa aaaba bbaab babba
 baaaa abbaa bbaab abbba abbaa aaabb baaaa aaaab bbaba bbaaa
 baaab aaaab bbbaa bbbba abbbb baaaa babaa baa
EACMGA535G          {0} ; 387
 baaaa abbba baaaa aaaaa abbaa abbab aabab babaa bbbba baaaa
 aaaba aabab babaa abaaa babab abaab bbbaa aaaba bbaab babba
 bbaa- abbaa bbaab abbba abbaa aaabb baaaa aaaab bbaba bbaaa
 baaab aaaab bbbaa bbbba abbbb baaaa babaa baa
EACMGG181G          {0} ; 758
 baaaa abbba baaaa aaaaa abbaa abbab aabab babaa bbbaa b-aaa
 aaaba aabab babaa aaaaa babab bbaab bbaaa aaaba bbaab babba
 baaaa abbaa bbaab abbba abbaa aaabb b-aaa aaaab bbaba bbaaa
 baaab aaaab bbbaa bbbaa abbbb baaaa babaa baa
EAGMCT43G           {0} ; 798
 aaaba abbaa baaba aabaa abbaa bbaab abbaa bbaba abbab a-aaa
 aaabb babab babaa aaaaa bbbbb babab bbaaa baaba abaaa bbbba
 babba aabaa aaaab bbbaa bbbaa baaab b-aaa baabb bbaba abbab
 aaaab aabaa abbaa abbaa abbbb baaab bbaab bab
ECGTAC198G          {0} ; 1327
 baaaa abbba baaaa aaaaa abbaa abbab aabab babaa bbbaa baaaa
 aabaa aabab babaa aaaaa babab bbaab bbaaa aaaba bbaab babba
 baaaa abbaa bbaab abbba abba- aaabb baaaa aaaab bbaba bbaaa
 baaab aaaab bbbaa bbbba abbbb baaaa babaa baa
EACTGA134V          {0} ; 1355
 aaaba abbba baaaa aaaaa abbaa abbab babab babaa bbbaa baaaa
 aaba- babab babaa aa-aa bbbbb b-aab bbaaa baaba bbaaa bbbba
 babaa abbaa aaaab aabaa bbbaa aaabb baaaa aaabb bbaba abbab
 aaaab aaaaa bbbaa bbbba abbbb baabb babab bab
ECGTGA563G          {0} ; 1574
 aaaba ab-ba baaaa -abaa abbaa bbaab baba- babaa bbbaa baaaa
 aabab babab babaa aaaaa bb--b ---ab bbaaa baaba abbaa bbbba
```

```
 baaba abbaa aaaab abbaa bbbaa aaaab baaaa aaabb bbaba abbab
 aaaab aabaa bbbaa bbbba a-bbb baabb babab bab
SSR_991              {0} ; 1608
 aaaba abbba baaaa aabaa abbab abaab babaa babaa aabaa abbbb
 aaabb babab babaa aaaab baaab baaab bbaaa baaba abaaa bbbba
 aabba abbaa abbbb bbbaa bbbaa baaaa baaaa aabbb bba-- -----
 ----- ----- ----- ----- ----- bbab- baaab bab
```
