# Supplementary material for: A sequence-anchored genetic linkage map for the moss, Physcomitrella patens
Source: Plant J. 2008;56(5):855–66. doi: 10.1111/j.1365-313X.2008.03637.x (PMC2667646; doi:10.1111/j.1365-313X.2008.03637.x)
Supplement: Supplementary file 4 [file tpj0056-0855-SD4.pdf]

SSR	Scaffold	Primer 1	Primer 2	Length
SSR_10	scaffold_169	TGGAGGTTGTTTCTTGGGTC	CTCACATTGCTCCTGCCATA	216
SSR_818	scaffold_169	ACATTTGCTATTATCAAGAATATAGGG	TTCTTGGACCTCACAATCCTTT	199
SSR_974	scaffold_116	attccactcaccctccactg	cttcactttttccggtccac	275
SSR_1166	scaffold_116	gggcagatcataaagttggg	ctcaagcattgcaaaaatgg	194
SSR_39	scaffold_173	CCACCGTAGCACCACCTAAT	GATCGATAAGGGTCTAGCCG	219
SSR_578	scaffold_173	ACGAAGAAGAAGCATCCGAA	AACTCCTCCTCCTCCCTCTG	260
SSR_107	scaffold_98	agtgctgagagcagcatcaa	tgtggagatttcgacccttc	212
SSR_12	scaffold_98	agttttcagcccacaccatc	accagccaaaattggacaag	178
SSR_25	scaffold_117	CTGCGATTTGTCGATTGCCTTG	CCAGAACTTAACCAAACTTCTCCGCA	167
SSR_55	scaffold_117	TGGCTGAAGCGTTTTAGGTT	AAGCACCTTCCATAACGTCG	167
SSR_289	scaffold_103	ttagggtagcgcatttcacc	caactctgctctcccacctc	232
SSR_168	scaffold_53	gggcaagagaggaaggagat	agcagctgaaccaaggaaaa	262
SSR_42	scaffold_83	AGCTGGGTTTCTGTCAAACG	GCCACAACATAAGAGCAGCA	143
SSR_506	scaffold_124	AGAAGGTGATCGGTGGTTTG	GATGCCCACAAAACTTTTCC	276
SSR_991	scaffold_124	agcacatcctgatacagccc	atgcaccgaatggagaaaac	188
SSR_508	scaffold_211	cttctcttaccggtgcaagc	ccacaccttttccctcaaa	207
SSR_1068	scaffold_102	TTCTGCTGCATGCAAACTCT	TAGGGCTTGTGAGTGCAGTG	140
SSR_849	scaffold_247	CATACAGGGGAAATTGTGGC	TCCAATGGTTCCATGGATTT	119
SSR_849	scaffold_102	CATACAGGGGAAATTGTGGC	TCCAATGGTTCCATGGATTT	119
SSR_554	scaffold_110	CTTGACAGAGTCCCGCTTTC	TGTCAATCTGTGGGCTCTTG	247
SSR_554	scaffold_184	CTTGACAGAGTCCCGCTTTC	TGTCAATCTGTGGGCTCTTG	247
SSR_54	scaffold_110	ACGTGTGTGTTCGACAGGAG	CACACCCAAAAATACCGACC	152
SSR_27	scaffold_114	TCACTGGCAAGACATTTAGAAACCCA	GGACATTGTCAGCTAGGGAATGCA	210
SSR_709	scaffold_8	CTTCGATTTCCCCTTTTTCC	ATTCCGATGGCGATAGTGAG	257
SSR_401	scaffold_114	gcggtatgatgtttgcagtaa	aggcattattgaacaagttttga	280
SSR_485	scaffold_114	gcacgtccaggtgaagagtt	ccttggaccaccactctgtt	193
SSR_187	scaffold_184	aacacctttcaccaccttgc	tgccatcgtccaagtctgta	184
SSR_127	scaffold_8	gggcgagtgcaaagaataag	gtgcttcacttcccaagagc	260
SSR_61	scaffold_114	cagagtggtcactggcaaga	tggaagtttgggaccttgag	276
SSR_1167	scaffold_62	gacgacgaagacaacagcaa	gctcaggcgaagaacaaatc	134
SSR_86	scaffold_364	AGGACTAACCGGTTGCAATG	GCAACTGAAGCGAAAGGAAC	186
SSR_538	scaffold_39	gtcaacggtcgaaaccttgt	aagctttccaatgactccaga	216
SSR_266	scaffold_39	ctcgcaatcttactcctccg	caagaagcggccatattcat	177
SSR_90	scaffold_84	TCAACGACCCAACTCACAAA	ACAAGCATTCTTGGCTTTCC	163
SSR_723	scaffold_84	GTTGCACGCAGAAGCTGATA	TCCTTTCGTCCCACAGTAGC	172
SSR_178	scaffold_84	ctagtgggacggctgtcttc	aaaagaaaacttgcggctga	135
SSR_59	scaffold_38	ATGTCGAGCAGCTGAGTGTG	ATCCCTATCACGAAACGCAC	214
SSR_447	scaffold_29	CATTGGCATTGAAATTGTGC	CCCTTGAGAGCGAATGAGAG	254
SSR_517	scaffold_230	ttgccctttctggaaattgt	ggttgggaatgagaaatgga	269
SSR_874	scaffold_139	attacagctgggtcactggg	gcgtcctcattgccatattt	163
SSR_145	scaffold_38	acgagtccatttgagcgact	ccgttttgcaaacaaacaca	114
SSR_72	scaffold_540	tgggttctagggttgattgc	ggaaacaaagatcggtcgaa	234
SSR_76	scaffold_44	GTCGAAAATCACCACTGCAA	TTCACTGCAGCAGCTTGACT	247
SSR_99	scaffold_400	CATCATCGCATCATCGAATC	CCGAATACTCCTCCCTCCTC	271
SSR_391	scaffold_42	GCCTCTCCTCTCCTCTCCTC	TCATTCACACAATTGTCTATTCCA	243
SSR_93	scaffold_75	TAGGGGAGTGGAGTGGACTG	ACCAAGTTCTGGAACAACCG	155
SSR_689	scaffold_42	TAGTCGACACGAGGAGGGTT	ACTCTCCAGCATCACCTGCT	263
SSR_101	scaffold_87	CTGCGGAGCCTAAATTCTTG	TTTCGCCTCATTGGAACTCT	134
SSR_875	scaffold_87	gggaggtggagtgagtgaga	tcgtctgcagagcaaaaaga	280
SSR_762	scaffold_207	ataggcttgtgtcaggtggc	gtgagtgtctgacaggcgag	184
SSR_142	scaffold_42	tccacgtttaggtctgtccc	tccctccctcacacactttc	155
SSR_141	scaffold_69	catcgagaaaattggggaga	cttcatcctcagagccttcg	251
SSR_800	scaffold_7	ACAGGGTACAAGTTCCGGTG	GAATGAATCCGAGCAGAAGC	164
SSR_68	scaffold_7	aaaatgacgagatatgtgccag	aatattttgtgcatgtgaataattg	244
SSR_22	scaffold_172	GCCTGCCCACCCATGTAGACAA	CGTTGAGTACTGGAATGCCGATCATC	198
SSR_672	scaffold_172	ACACTCACTATGGGAACGGC	ACGGCGAAGTTTCTGTCAAG	262
SSR_1017	scaffold_433	CCGCAGCATTACAAACTTGA	TGTCAGGGCTCCTTGAAACT	250
SSR_796	scaffold_254	TGACTCGGGCAAGTCTTTCT	GAATCCAACTTCCGTAGCCA	280
SSR_923	scaffold_20	tgccactactgcaacgtctc	acaacacgtcgcagagcata	277
SSR_194	scaffold_519	gagagacctcgctcgctg	tgctccctctgctaccactt	143
SSR_670	scaffold_414	cttccggagtgagtggtga	cctcctgaaactccaccaga	243
SSR_837	scaffold_296	AGAAGGAGTTGCTGCGACAT	TGTTGTGCAGTTGGGTTCTC	187
SSR_15	scaffold_1	TTTTGATGGTGGGTATGGGT	AAGGGCCTGGCTTAGTGAAT	278
SSR_1079	scaffold_1	GGCATAATAATTTGACGCCG	AAAATTTTGGCGGAGGAGTT	107
SSR_675	scaffold_1	gggagggactggtcatgtta	tcatcatcccctctggtagc	160

SSR_244	scaffold_1	agtggtggaggcttgagaga	cctttaacgtgctggacaca	152
SSR_28	scaffold_138	AAGGCGTGGCCAGGAAGTACCA	GCGACGTCCGTCAATTCCGAA	217
SSR_154	scaffold_138	ggccaggaagtaccagaaca	gtccgtcaattccgaacaac	205
SSR_494	scaffold_256	CCTGGGGACCTGCTATACAA	GCAGGTGAACGCATAACAGA	203
SSR_525	scaffold_149	cccacacaatttgtcactgc	gcccccatgtatcctacctt	207
SSR_60	scaffold_159	gtcctggacgcaagcaag	ctgcaaaattccccaccc	124
SSR_164	scaffold_217	tttgagggctggatttgttc	caactcttcccacctctttcc	113
SSR_1100	scaffold_159	caactccaagcttctctgcc	ctccgatcgctactgagtcc	231
SSR_581	scaffold_335	AAAGATGACAGCACCGAACC	CTTCATGTTCCGAGTTGGCT	239
SSR_45	scaffold_455	GCAAGCTAGAAGTCATGGGC	ACGTCCACGAGGAAGAACAC	230
SSR_1070	scaffold_303	TTCCCGACGTATTAAGTGGC	AGGCCAGCAGCAACAGTTAT	242
SSR_91	scaffold_109	GTGTCCGTGCTATCCACCTT	CCATGTCTCCTGTTCGTCAA	236
SSR_808	scaffold_300	gcagtcaccgcacacttaaa	tgcttccaccctccatttag	116
SSR_697	scaffold_128	TTGCTTGTTTGTCTTCGTGC	AAAGAAGCGGACAGGTCAGA	240
SSR_1069	scaffold_246	TCCCCTCCCATATAACTCCC	GCCGAACGAACGAACTAGAC	262
SSR_948	scaffold_70	GCATTGTGTCAACGATCACC	TTTGGGTGCTCAGAGAGGTT	107
SSR_708	scaffold_56	GGTGTTTAGTGATCGGCGTT	TGATTCAGGCATATGAGAATTTG	205
SSR_582	scaffold_70	GTAGCAGCATCGCACTCCTC	CGGAGAACCTGGAAGAATGA	257
SSR_824	scaffold_352	tcctgctgaccaacctctct	gaatcggagaatttttggca	141
SSR_133	scaffold_246	agccttaacatcaacgccac	actctcccccacacacaaag	168
SSR_207	scaffold_70	cattccttctccccttcaca	tcacactcactctatctctctccc	216
SSR_325	scaffold_91	gacgatgacgagaagcacaa	tgatcatacttggacggcaa	188
SSR_1090	scaffold_70	ccgttatccacctaaaggaaa	gcacatggtgcactgttgta	184
SSR_807	scaffold_18	AGGAACCAAGTCCCTTTGCT	CGGCAACACTTTCACATCAC	159
SSR_826	scaffold_196	ATCGAACCGTTCCTTTTGTG	GGAAGGCAAGCAGGTACAAG	225
SSR_1142	scaffold_387	GACTGGCAAGTCCCAGACAT	AGAGATCAGAGAAGCGTCCG	257
SSR_123	scaffold_73	gatgcggagtttgtagagcc	atacttctcggacgcgacac	237
SSR_806	scaffold_69	CATCGTGATTCTGCACTGCT	TTCCGCAAGACATGATGAAG	229
SSR_1120	scaffold_69	TTGAGGGGTGGAGAAGTTTG	CAGCAGGTGAACACAGGAGA	236
SSR_927	scaffold_69	aaagcaaaataagagatttgacaaaaa	tgagtgaatatattggcccttaaaa	270
SSR_850	scaffold_59	TGAAGCCAGTAGATGACCCC	TTGAACGAGACCAAAGGGAG	225
SSR_152	scaffold_59	ctgaggccagtttccatgat	acacatgcacacaccagcac	182
SSR_634	scaffold_133	GGTAGCTTCAGCGAAAGACG	ACTGCAATGGAAAGGGAGAA	245
SSR_490	scaffold_133	ATTCGGAGAGAAGATCACGC	CGAATGGTAAGGGCAGAAAA	158
SSR_104	scaffold_107	ttcaggcagtaacgtgtcca	ggaatcaaacaaaggcttcc	164
SSR_219	scaffold_305	ggaaggtctgcgaggagat	atgcccttgctacggttc	135
SSR_239	scaffold_158	tctgtggtgttggtggtgat	tagcaattggcaaatacgca	245
SSR_149	scaffold_182	ccggtggacgtgactagagta	catcacatcacacccgctac	242
SSR_172	scaffold_421	tcccaaggaggtgaagattg	aaaacttgtgtatagacatcaaccaaa	126
SSR_773	scaffold_5	caccatctccaccacctctt	cttccagtttgcttgcttcc	269
SSR_150	scaffold_134	CTACTGCTGTTTGGCCCATT	AATAAATCCCCTCCGACACC	160
SSR_759	scaffold_134	gatttagccccacaaaacga	tttgaatttttccgcactcc	184
SSR_775	scaffold_251	TGTGTAGCAGAGAAATGGCG	CCCTCGCGATTGAATAACAT	196
SSR_983	scaffold_275	GCCAGTTTGTGTTTCGGATT	AATATACCGAAGACACGGCG	236
SSR_789	scaffold_162	CCAGCCTGAAACACACTTCA	AACTGGGCAGAATACATCGG	183
SSR_925	scaffold_266	ccaaatctcatgactgctaacaa	ttgcctttttgtgctattcc	213
SSR_159	scaffold_162	ctggaggggctacaattcaa	tgattggaaatggcacgtt	183
SSR_732	scaffold_47	CTCGGATACACCAACACACG	ATGCAAACCACACATCCAGA	111
SSR_153	scaffold_47	gcagggagatggtggagtta	aaagaagcgcagcatcaaat	225
SSR_781	scaffold_15	ATTAAGCGCGGAGAATTCAA	ACTGTTTTCCGCAATCCAAC	167
SSR_938	scaffold_302	ctaaatgcgtgattgcatgg	ccagtggacaaatcggaact	117
SSR_229	scaffold_24	cagcaaattgagcgtagcac	acaaattcttccatgtccgc	153
SSR_181	scaffold_29	ttctactctgctgcgctgg	cagtgtggtggaaaaacacg	233
SSR_182	scaffold_220	tgcggcttcattacttgttg	gcatcaaaacaaaaacacagc	275
SSR_671	scaffold_220	ttcctccacctcacttgctt	catgggggcaacagttattt	259
SSR_117	scaffold_74	atgctgctgctgatttttcc	gaggtccttccttccctgag	256
SSR_203	scaffold_25	atacgctgcagacgttgttg	gtattcccaggaggcaatga	124
SSR_592	scaffold_101	CACACCGCAAGTTCAAAATG	GTCGAGGAGGAGAGGAGAGG	254
SSR_1072	scaffold_101	GCGTCTTGCTTCACCTTTTC	CCAGTTCCCTACGAAAACCA	204
SSR_798	scaffold_101	CGGCATAAAGAGGAAACCAA	GACCCAAATTCAGTTGGCAG	234
SSR_586	scaffold_504	TCTGGAACCCATTCAACCTC	CCCCTCGTCTTCCTTGTTCT	260
SSR_797	scaffold_101	TTTTCATCTCCCCCTCTCCT	GCCACTGTCACCAAAAACCT	239
SSR_787	scaffold_403	GAAGGGAGGAGACGGGATAG	CGTCGTTCTCTTCGCATGTA	184
SSR_915	scaffold_101	GCATGTGTTCGGTGCATTAG	CACCCGGTTCTTCTTCTTCA	183
SSR_183	scaffold_101	ccataccaaaagcgaaggaa	tctctccctccctccttacc	143
SSR_1101	scaffold_177	GCGAGGATCTGAGTTGCTTC	AAGCCATCGACAGTGAAACC	227

SSR_588	scaffold_177	ccccttcattccattttcct	ctcgagatcgagatggatgg	265
SSR_199	scaffold_478	tgattccttccccaagagtg	ggggcccgacttagtaagaa	211
SSR_816	scaffold_14	cggtttccctttcttctcc	aatcccctttcacacacagc	157
SSR_700	scaffold_195	ttgcaagtaccacgcattct	tcaacctccctcctcgac	193
SSR_185	scaffold_177	gtcaacgggttgtctgtcct	agccacacatccaagcaact	163
SSR_809	scaffold_168	CGAGTCTTCCCTTTCTGTGG	CCAGGGCGAGAATATACCAA	249
SSR_499	scaffold_57	CGCTTACTGCTTTCCCAATC	TGAACTGGGTCACAACCAAA	148
SSR_1184	scaffold_129	cactctctcccatgcagtca	tggtgtgaatgtggaaggaa	140
SSR_198	scaffold_57	ctgctcgccctgtatctgtt	gctgcatgtctgaggtgaaa	270
SSR_728	scaffold_57	ctggcacaaccaggattttt	taagcaagtgtgttgcccag	241
SSR_1123	scaffold_121	GAAGGATTTGGAGGAGGAGG	CCCTTCGGAACACGAAATTA	114
SSR_253	scaffold_12	ctgcaattcgctttgttacg	gaaaatcggaagtgcgaaaa	101
SSR_488	scaffold_99	TGCTCACAGGAGACACCAAG	GAAGCAGAAGAAGAAGGCGA	267
SSR_421	scaffold_359	TAGTTGAAGTTGCCGTGTGC	ACGCCTGAAGAAAAACGAAA	176
SSR_641	scaffold_253	ccgaataggctgtgaggatt	tggctgagccctaaaacagt	168
SSR_1061	scaffold_66	gacgacagtgaaacctggct	tgaaattaaaggcgagaagagg	135
SSR_603	scaffold_203	GCACGTGACGATCCTGTAGT	GGTTGCATATTCCCATGGTC	216
SSR_1092	scaffold_314	TGGTAAAGGGACGGACAAAG	TTGTTGAGTGGATCGCTACG	274
SSR_1073	scaffold_273	CGTGGCTATGACCCTCTCTC	TGGTGGTAGATGATGCAGGA	264
SSR_389	scaffold_188	AGCTGAGGAAGGAAGTGCAG	CCATGTAATCCTCATGCCCT	264
SSR_598	scaffold_278	AATCCTGCTGCCATGAGTTT	TTCACGCAGCGATTAGAAGA	234
SSR_431	scaffold_30	GCCCTCTTTGTTGATGGTTC	CCAAGGAATAACTTAGAATGGGG	236
SSR_819	scaffold_358	ACTGATACTTGCTGCCCTGC	ATTGCGACACAAGCAACAAC	259
SSR_788	scaffold_322	GGCATTGAGATCCAGCAAGT	CCACTTTCTCCGTCCCTACA	223
SSR_747	scaffold_141	TTGAGCTGGATCTGCAAGAG	ATGAACCCAGGCATAACTGC	110
SSR_510	scaffold_58	GGGTGCTCTTGACACCTGAT	AAACAAACTCCGCTACCCCT	265
SSR_713	scaffold_58	GTTTTGGTGACCAGGCGTAT	TCACAAATAGCCCATCCCTC	121
SSR_516	scaffold_58	TTGTGTGTTTAGGCCAGTCG	AGCAGCCACAACAACAACAG	167
SSR_678	scaffold_58	CGTGAATTGAGGCTGCAGTA	CTGAACGCCTCCTTGAACTC	244
SSR_609	scaffold_293	tagtgaagggaagggggttt	gcaaatcaagcagggagaag	279
SSR_717	scaffold_58	gcagggtttgtagttggcat	ccctacctttccactgacga	204
SSR_1084	scaffold_141	gcaaggtctggtagggatga	ggctccttatctttgggagg	278
SSR_765	scaffold_71	CATTGAAAAGGCTGGGAGAA	CTCCTCGTTTTCGTCTACGG	271
SSR_513	scaffold_225	ATTGGGTCGTCTTTCAGGTG	ATTTTTGCGAACGATTGGAG	234
SSR_568	scaffold_92	CAAAAAGAATGGGTGGAGGA	CATTCTTGCTTTGCTCACCA	279
SSR_693	scaffold_100	TCAACGCAAACGTCAACTTC	TTGCACGAATGGCTACAGTC	272
SSR_616	scaffold_210	GCATGATCACCAAAACGATG	GGAAGGAACGAAGGAAGAGG	206
SSR_778	scaffold_82	GCATCAAACATGCATCCACT	TGTTTCCCACTCACCATGAA	271
SSR_680	scaffold_401	GTCTGGTTGTGTTGCTCACG	TGTCAGCCTCGCTTACATTG	120
SSR_1060	scaffold_167	aaccgacctggtccctctat	tttctttcccttccggatct	209
SSR_754	scaffold_21	TCTCATTGTCAGCAAACCCA	CTCGTGTCCCATAAGCACCT	235
SSR_756	scaffold_21	catcaaaagcttcaacgggt	gtgcaagtggcacaaatgtc	179
SSR_1067	scaffold_304	TCTTCTTCTCTGGCGGTGTT	TGGGTGAGGAGGTTTGACTC	220
SSR_752	scaffold_76	TTCATCTTTTGCAGGCACAC	TAGCTCCATCCATCGCTCTT	227
SSR_1094	scaffold_90	CAGACAGAAATTGGGACGGT	TTGAAAGCACATTCAATCACA	255
SSR_799	scaffold_90	ttatcgctgatgaggcagtg	gaagggacggagtctgtgag	184
SSR_835	scaffold_119	CCCCCTTATCTCTCTCACCC	CATAGGCCAAGAAAAGAGCG	147
SSR_1001	scaffold_119	tcctcagctcttggttcgtt	atacccggggttttactcca	249
SSR_1095	scaffold_2	CTCACGGAGTAAAGGCCGTA	GATGCCATCTCTTGGCTTTC	185
SSR_831	scaffold_2	GGCCAATCCAAGTGAAGAAA	AGGACCCATCGTAAGCCTCT	257
SSR_1078	scaffold_2	TGTTTCAGGGATCGTGTTGA	TGCTTGCAACCTTCTGATTG	192
SSR_1188	scaffold_2	cacacaaagacggacgctaa	cggaggtatttcacctgagc	268

Supplementary Table 1: SSR marker information

This table identifies the individual SSR loci mapped in this study, their location within the *Physcomitrella* V1.1 genome sequence by sequence scaffold, the nucleotide sequences for each primer pair, and the length of the fragments amplified from the Gransden genotype.
